# Trialkylphosphonium oxoborates as C(sp^3^)–H oxyanion holes and their application in catalytic chemoselective acetalization†

**DOI:** 10.1039/d3sc03081d

**Published:** 2023-10-20

**Authors:** Vincent Ming-Yau Leung, Hong-Chai Fabio Wong, Chun-Man Pook, Ying-Lung Steve Tse, Ying-Yeung Yeung

**Affiliations:** a Department of Chemistry and State Key Laboratory of Synthetic Chemistry, The Chinese University of Hong Kong Shatin, NT Hong Kong China stevetse@cuhk.edu.hk yyyeung@cuhk.edu.hk

## Abstract

The use of trialkylphosphonium oxoborates (TOB) as catalysts is reported. The site-isolated borate counter anion in a TOB catalyst increases the availability of C(sp^3^)–H to interact with electron donor substrates. The catalytic protocol is applicable to a wide range of substrates in the acetalization reaction and provides excellent chemoselectivity in the acetalization over thioacetalization in the presence of alcohols and thiols, which is otherwise hard to achieve using typical acid catalysts. Experimental and computational studies revealed that the TOB catalysts have multiple preorganized C(sp^3^)–Hs that serve as a mimic of oxyanion holes, which can stabilize the oxyanion intermediates *via* multiple C(sp^3^)–H non-classical hydrogen bond interactions.

## Abstract 2

Trialkylphosphonium oxoborates with site-isolated borate counter anions were found to have preorganized C(sp^3^)–Hs that can interact with electron donors. The system can serve as a mimic of oxyanion holes to catalyze chemoselective acetalization.

## Graphical abstract

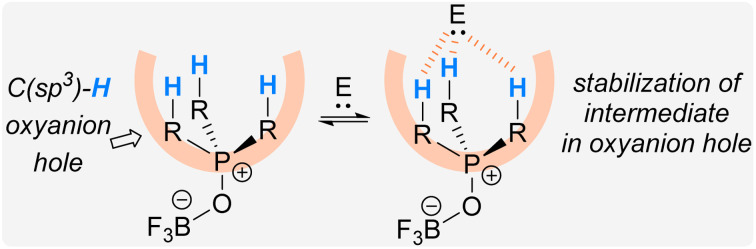

Oxyanion holes are important active sites in enzymes and they can regulate various transformations by stabilizing the high energy oxyanion intermediates in transition states.^1^ Preorganized and structurally well-defined conventional hydrogen bonds (*e.g.* from N–H and O–H) usually serve as the functional groups in typical oxyanion holes. Many biomimetic organocatalysts take advantage of the concept of oxyanion holes for various catalytic chemical transformations.^2^

C(sp^3^)–Hs in hydrocarbons bearing electron-withdrawing groups can form attractive noncovalent interactions with electron donors. The discovery and investigation of this type of molecular interaction can be dated back to the 30 s to 50 s.^3^ Compared with conventional hydrogen bonds that have relatively high energy, these C(sp^3^)–H⋯X (X = electron donor) interactions are weak (usually <4 kcal mol^−1^). In literature publications in recent years, the term “non-classical hydrogen bond (NCHB)” has been used frequently to classify this type of interaction, which plays important roles in structural biology, supramolecular chemistry and crystal engineering.^4^ NCHB is known to consist of multiple types of interactions such as electrostatics, dispersion, induction, and exchange-repulsion. Although NCHB is not universally defined, this type of weak interaction has been labeled as non-conventional hydrogen bonding since 1998.^5^ It is also sometimes simply denoted as C–H⋯X interaction.^6*a*,7*a*–*j*^ For ease of communication and consistency, “NCHB” is used to refer to this kind of weak interaction throughout this manuscript.

In the past few decades, it has been recognized that C(sp^3^)–H can participate in controlling selectivity in organic transformations in a number of studies.^6–8^ Saturated hydrocarbons bearing C(sp^3^)–H NCHB donors were also found to serve as oxyanion holes^8*f*^ and play a pivotal role in stabilizing negatively charged intermediates in catalysis, although the cases are limited.^9^ For instances, alkylonium salts including alkylammonium^10^ and alkylsulfonium^11^ bearing C(sp^3^)–H can activate electron-donor substrates and enhance the reaction rates *via* NCHB interactions. Although the mechanistic evidence is unclear, some phosphonium salts^12^ bearing C(sp^3^)–H could serve as acid catalysts and they are also believed to activate substrates *via* NCHB.^9*c*^ These catalysts are highly stable, non-Brønsted acidic, and easily modifiable. However, typical onium salts such as onium chloride were found to be ineffective catalysts. X-ray crystallographic analysis on these onium salts^10,11^ indicates that the counter-anion interacts with the NCHB donor preferentially. As a result, the relatively basic counter-anions such as chloride (X = Cl) occupy the electropositive C(sp^3^)–Hs and weaken the NCHB catalysts (Scheme 1A). To alleviate this problem, incorporation of a less coordinating counter-anion *via* ion-exchange with a stoichiometric amount of silver salts was employed.

**Scheme 1:**
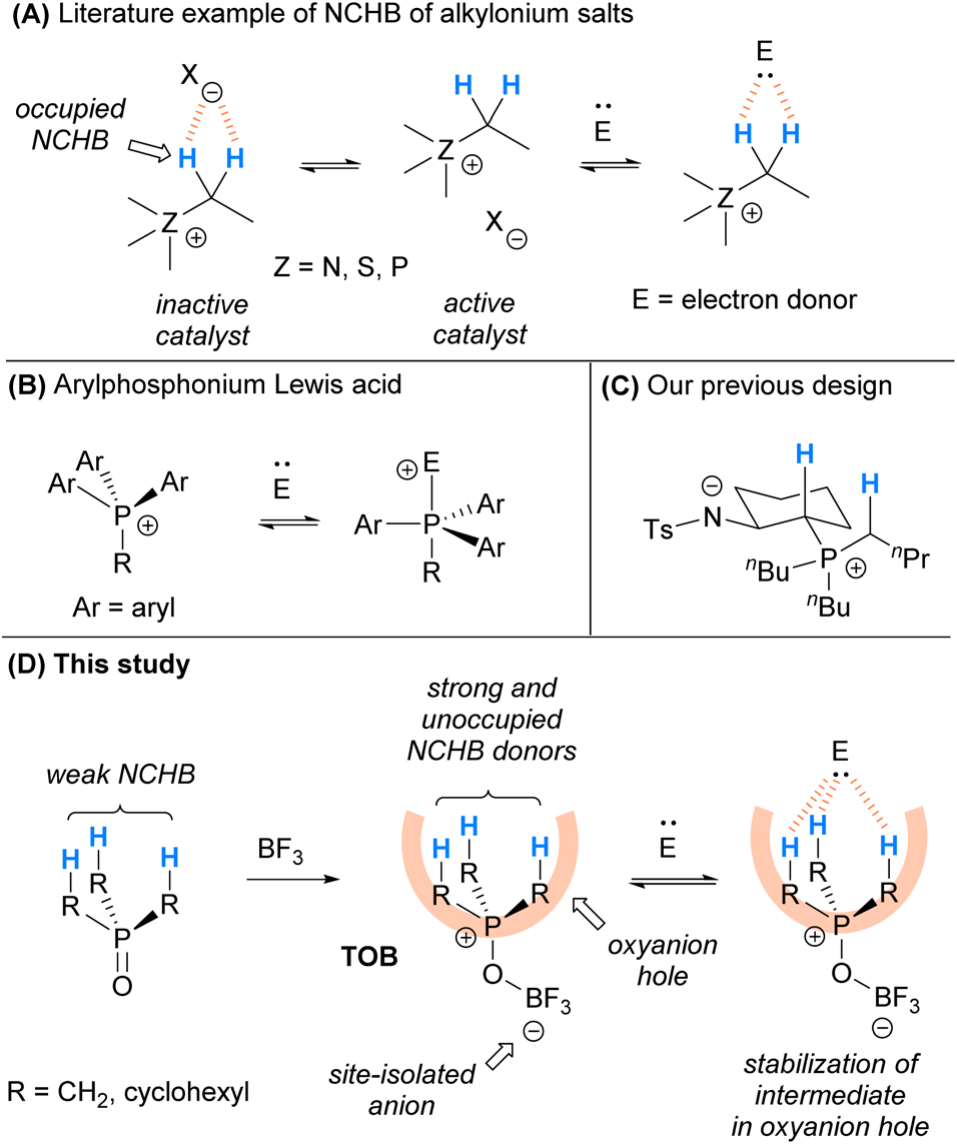
Design of the trialkylphosphonium oxoborate as an oxyanion hole *via* strong C(sp^3^)–H NCHB. (A) Literature reports on using alkylonium salts as NCHB catalysts; (B) Literature reports on using arylphosphonium salts as Lewis acids; (C) Our previous study using amide/phosphonium zwitterions as catalysts; (D) The use of trialkylphosphonium oxoborate as a mimic of oxyanion holes for catalysis in this study.

Arylphosphonium cations are reported to be good Lewis acids for different organocatalytic reactions.^13^ It is believed that electron donors could coordinate to the arylphosphonium cation to give hypervalent phosphine in the transition state (Scheme 1B). Recently, we have designed a series of amide/phosphonium zwitterion catalysts (Scheme 1C) and applied them in some catalytic reactions.^14^ Instead of aryl substituents, phosphonium with alkyl groups was used in the catalyst design. The C(sp^3^)–Hs adjacent to the phosphonium cation were found to be responsible for the activation of substrates *via* NCHB. In these catalysts, the amide anion is site-isolated from the alkylphosphonium cation to achieve satisfactory performance.

Inspired by the seminal work of arylphosphonium Lewis acids and based on our recent experience, herein we report the development of trialkylphosphonium oxoborate (TOB) as a mimic of oxyanion holes for catalytic transformations (Scheme 1D). The TOB catalysts consist of a site-isolated borate anion and so the cationic moiety is not occupied by the counter-anion. Due to the less s-character of alkyl C(sp^3^)–H compared with aryl C(sp^2^)–H, C(sp^3^)–H has a longer bond length and is easier to polarize. In addition, alkyl groups are often bulkier than aryl groups because of the planar structure of arenes. As a result, the C(sp^3^)–Hs in TOB might become the electron-accepting site and can serve as effective oxyanion holes. The catalytic protocol was found to be applicable to a wide range of substrates in the acetalization reaction. Moreover, the catalyst was able to provide excellent chemoselectivity in the acetalization over thioacetalization in the presence of alcohols and thiols, which is otherwise hard to achieve using typical acid catalysts.

The TOB catalysts can be prepared by simply reacting phosphine oxides with boron trifluoride (Scheme 2). For example, trimethylphosphonium oxoborate catalyst TOB-1 (84%) was prepared by mixing trimethylphosphine oxide (1a) and boron trifluoride diethyl etherate in methylene chloride at 23 °C, followed by crystallization. In a similar manner but with the use of tricyclohexylphosphine oxide (1b), TOB-2 was obtained in 91% yield. A single crystal of TOB-2 was successfully obtained and analyzed using X-ray crystallography to confirm the structure. These TOB catalysts were found to be insensitive to moisture and bench-top stable. The stability of these TOB complexes was also studied by treating them with water and no decomposition was observed (also see ESI, Fig. S1†). The high structural stability of these TOB complexes could be attributed to the strong B–O bond.^15^

**Scheme 2:**
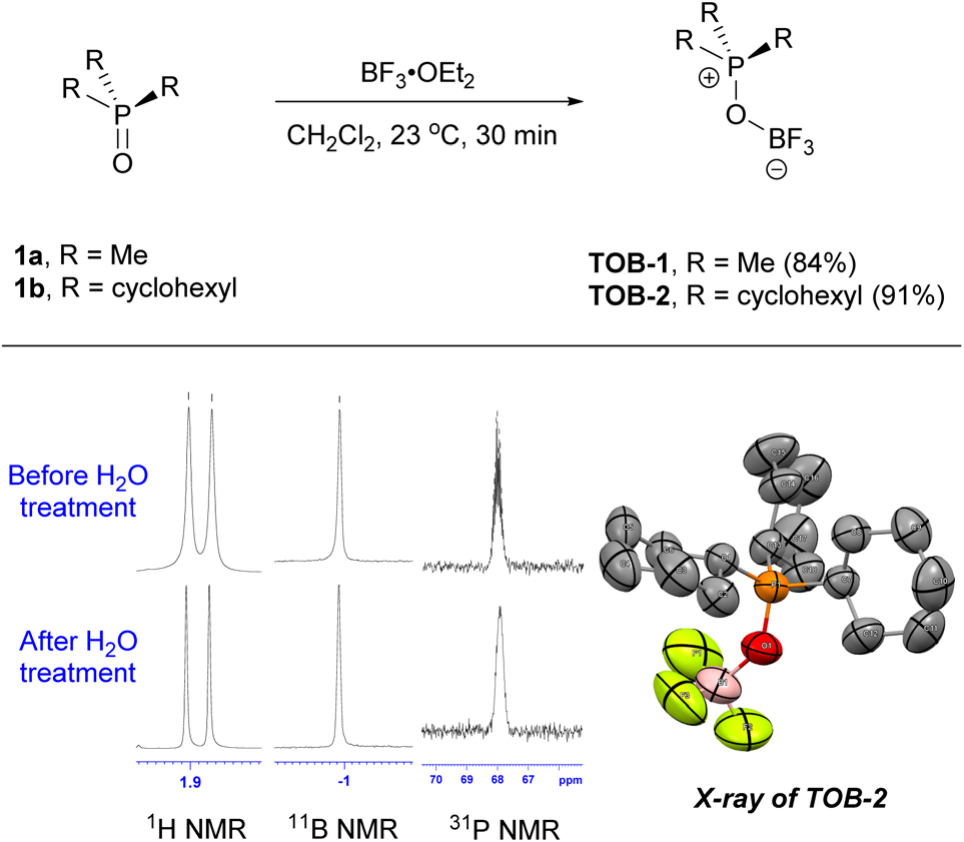
Synthesis of the TOB catalysts.

We began to evaluate the catalytic performance of TOB using acetalization of benzyl alcohol and dihydropyran (2) in the benchmarking study, which typically requires the use of Lewis or Brønsted acids as catalysts and might suffer from problems such as functional group compatibility (Scheme 3). No reaction was observed in the absence of a catalyst. To our delight, good yields of the desired product 3a were observed with 1 mol% of TOB-1 and TOB-2. The reaction could still proceed well even at a 0.1 mol% catalyst loading with elongated reaction time. The performance of TOB-3 that has phenyl instead of alkyl substituents was inferior to that of TOB-1 and TOB-2, indicating that the C(sp^3^)–Hs in the catalysts might be responsible for the high reaction efficiency. In sharp contrast, phosphonium salts P1–P5 bearing different counter anions were found to be ineffective in promoting the reaction. β-Ketophosphonium salt P6 that is believed to be a good C–H NCHB catalyst^12*d*^ was unable to catalyze the reaction at a 1 mol% catalyst loading. These results highlighted the importance of site-isolation of counter-anions in promoting the catalytic performance of the C–H NCHB. We have also examined the zwitterion catalyst P7 that contains a phosphonium cation.^14^ However, no reaction was observed and the starting material was recovered. Although P7 has a site-isolated phosphonium cation, we believed that the Brønsted basic amide anion in P7 might interact with alcohol *via* a hydrogen bond,^14*a*^ leading to diminished Brønsted acidity. A brief survey on different solvents revealed the superior performance of chloroform in the reaction (see ESI, Table S1†).

**Scheme 3:**
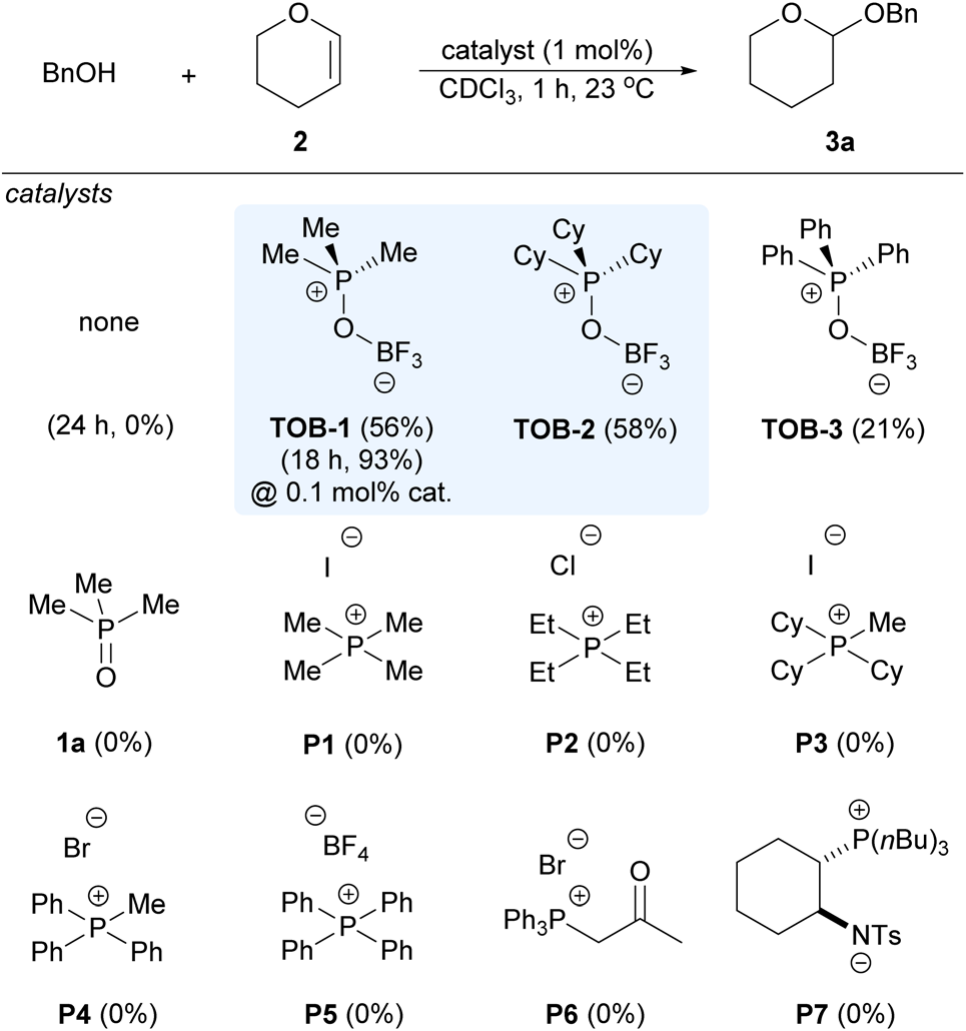
Catalyst comparison in alcohol acetalization. Reaction conditions: reactions were carried out with benzyl alcohols (0.20 mmol), 3,4-dihydro-2*H*-pyran (2) (0.24 mmol) and catalyst in CDCl_3_ (0.4 mL) at 23 °C. The yields were measured by NMR with dibromomethane as the internal standard.

Next, the substrate scope was studied using dihydropyran 2 or dihydrofuran 4 as the reaction partner (Scheme 4A). A number of benzyl alcohols bearing electron-donating and withdrawing substituents were compatible with the catalytic protocols to give the corresponding pyran acetal 3a–3h and furan acetal 5a–5h. Other alcohols were then studied. Various phenols were used in the reaction to give the desired acetals 3i–3t and 5i–5t in excellent yields. A range of alcohols with furanyl, pyridinyl, dibenzofuranyl, aliphatic alkyl, cycloalkyl, olefinic and alkynyl substituents were also examined and the resulting acetals 3u–3ac and 5u–5ac were furnished in good-to-excellent yields. Other than alcohols, we have also briefly examined the use of thiols as the reaction partner in the acetalization (Scheme 4B). After optimization, the desired thioacetals 6a–6f were obtained smoothly when the reaction was carried out with 1 mol% of catalyst at 60 °C. The reaction could also be performed at room temperature with a lower yield (6a, 50% at 23 °C).

**Scheme 4:**
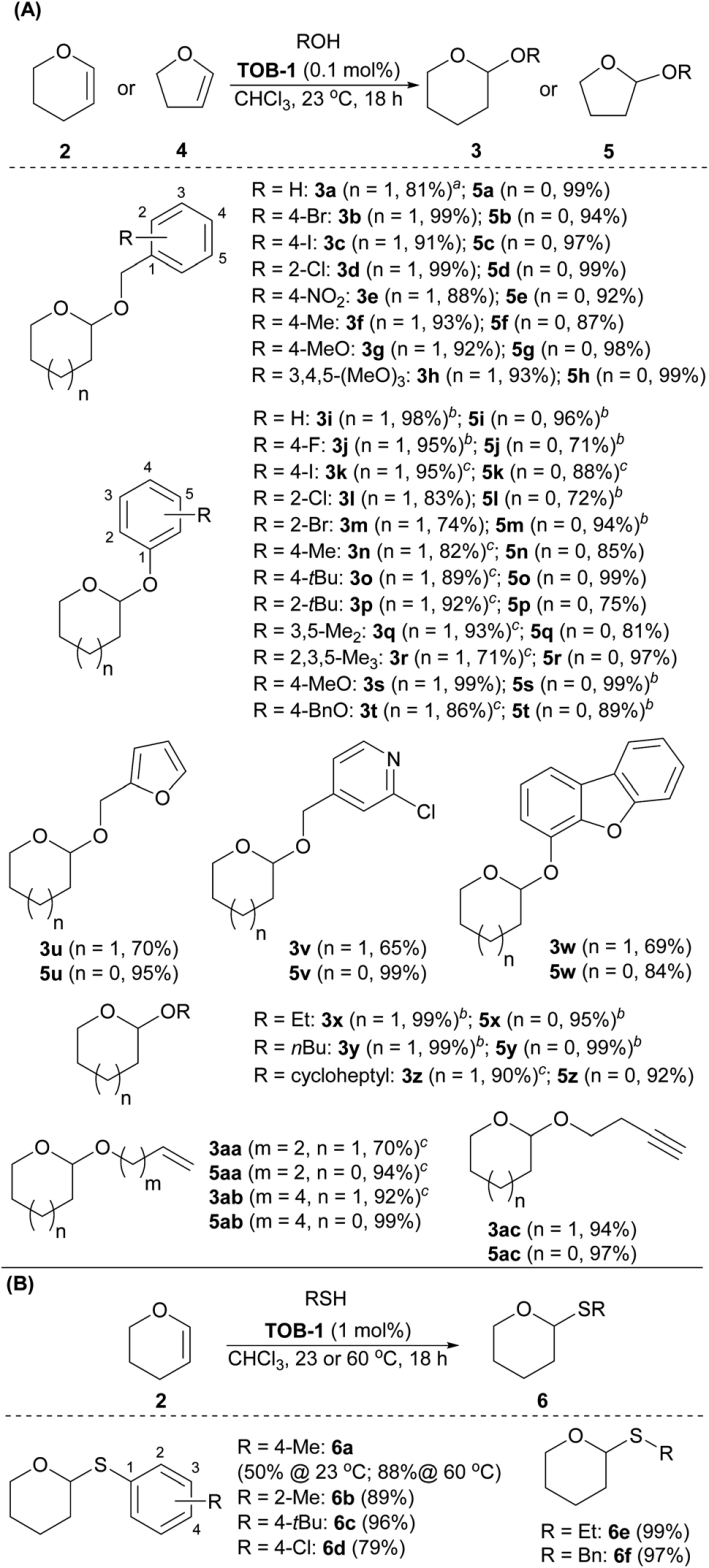
Substrate scope of acetalization. (A) Acetalization of dihydropyrans and dihydrofurans with alcohols; (B) Thioacetalization of dihydropyrans with thiols. Reaction conditions: reactions were carried out with alcohols or thiol (0.20 mmol), 3,4-dihydro-2*H*-pyran (2) (0.24 mmol) or 2,3-dihydrofuran (4) (0.24 mmol), and catalyst TOB-1 in CHCl_3_ (0.4 mL) for 18 h. ^*a*^2.0 mmol scale. ^*b*^The reaction time is 48 h. ^*c*^The reaction time is 72 h.

In a study using equimolar amounts of 4-methylphenol and 4-methylthiophenol together with dihydropyran 2 in chloroform, *S*-acetal 6a was obtained as the dominated product using 0.2 mol% of TOB-1 or BF_3_ as a catalyst (Scheme 5). Interestingly, when the less polar solvent toluene was used, 48% *O*-acetal 3n was obtained and no *S*-acetal 6a was formed when using the catalyst TOB-1. The yield of *O*-acetal 3n was improved by prolonging the reaction time. Satisfactory performance could also be achieved by using 1 mol% of TOB-1 for 24 h. In contrast, a mixture of *O*-acetal 3n and *S*-acetal 6a was obtained when using the Lewis acid catalyst BF_3_ in toluene. The Brønsted acid catalyst camphorsulfonic acid (CSA) was also examined and the results were unsatisfactory. These results clearly show that: (1) the TOB catalyst appears to have different catalytic mechanisms compared with typical Lewis or Brønsted acid catalysts; (2) the catalytic performance of TOB is not due to trace Lewis or Brønsted acid.

**Scheme 5:**
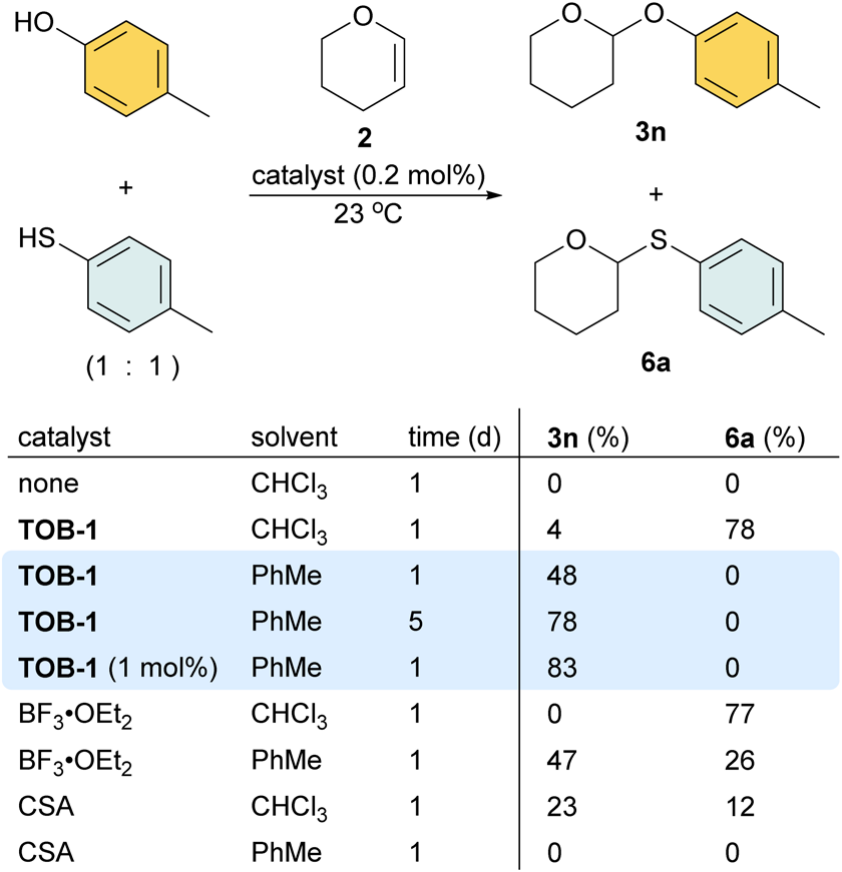
Study on the O/S chemoselectivity in acetalization.

Competition experiments were then performed with different combinations of alcohols and thiols (Scheme 6). To our delight, excellent chemoselectivity was observed in various examples using alcohols/thiols bearing the same substituents (entries 1 and 2). Satisfactory chemoselectivity was also obtained with alcohols and thiols with different substituents (entries 3–10). In particular, in the competition experiment using 2-*tert*-butylphenol and 2-methylthiophenol (entry 5), the less bulky 2-methylthiophenol reacted to give thioacetal 6b (56%) as the sole product using BF_3_ as the catalyst, attributed to the steric effect. In sharp contrast, the inherent preference was overridden with the TOB-1 catalyst; 3p was obtained in 76% yield and no 6b was detected.

**Scheme 6:**
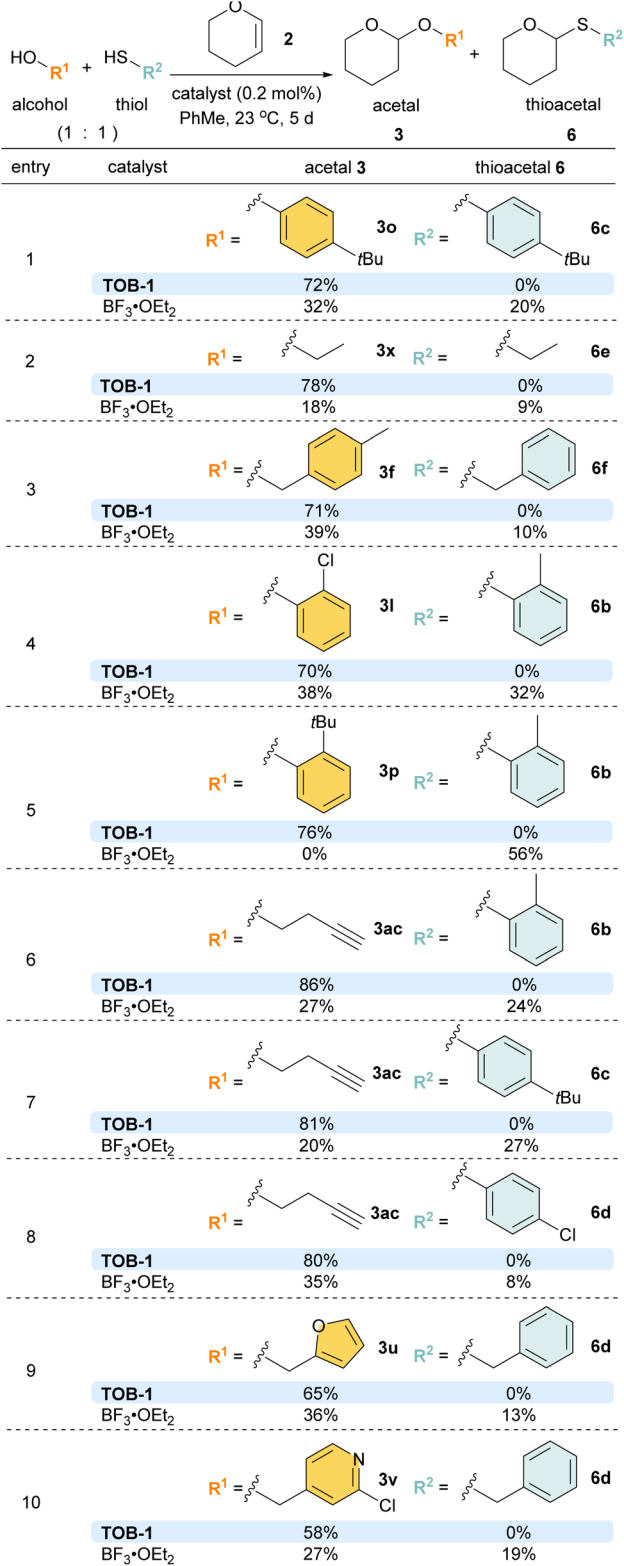
Examples of chemoselectivity acetalization.

Since the reaction mechanism should go through protonation of dihydropyran 2,^16^ we believe that a key component in the mechanistic picture should involve complexation of the alcohol with the TOB catalyst to generate a Brønsted acid. The interaction between an alcohol and TOB-1 catalyst was then investigated by ^1^H NMR titration. Benzyl alcohol was used as the electron-rich titrant and up-field shift of the methyl proton signal of TOB-1 was also observed (Fig. 1A). However, no chemical shift was observed in ^11^B and ^31^P NMR experiments, suggesting that the proton chemical shift is not due to the solvent effect. These results indicate that the electron-rich oxygen atom in benzyl alcohol could interact with the C(sp^3^)–Hs of TOB-1*via* NCHB.

**Fig. 1:**
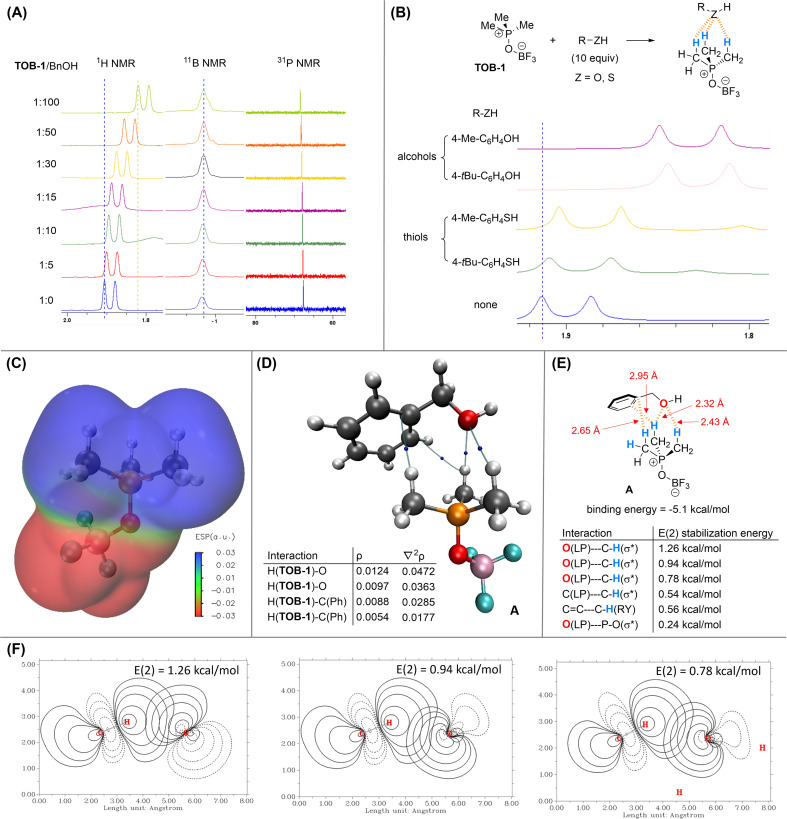
Studies on the interactions between TOB-1 and BnOH. (A) NMR titration experiment with TOB-1 and BnOH; (B) comparison of the TOB-1 C(sp^3^)–H chemical shift in the presence of alcohols and thiols; (C) ESP analysis of TOB-1; (D) AIM analysis on the interactions between TOB-1 and BnOH; (E) NBO analysis on the interactions between TOB-1 and BnOH; (F) selected major E(2) stabilization energies of the C–H⋯O interactions.

It is intriguing that the TOB catalyst shows chemoselectivity in distinguishing the structurally similar alcohol and thiol. Thus, NMR studies were also performed to compare the interactions between TOB-1 and different alcohols/thiols (Fig. 1B). It was found that TOB-1 readily interacts with the alcohols as indicated by the magnitude of TOB-1 C(sp^3^)–H chemical shift. In contrast, the chemical shift was much less significant when the alcohols were replaced with thiols. Based on these results, it appears that the NCHB from TOB-1 C(sp^3^)–H favors the interaction with oxygen over sulfur, which follows the hard-soft acid-base principle.^17^

DFT computational studies at the level of M06-2X/aug-cc-pVTZ///M06-2X/6-311G(d,p)^18^ with Grimme D3 dispersion correction^19^ were performed with Gaussian 16 (ver. C.02)^20^ to gain a deeper insight into the mechanism. Electrostatic potential (ESP) analysis (generated from Multiwfn 3.8)^21^ was conducted on TOB-1 (Fig. 1C). It was found that the positive (blue) region is localized at the C(sp^3^)–Hs while the fluoroborate moiety is largely negative (red). Since the positive region is not occupied by the negative charge, these C(sp^3^)–Hs could serve as an oxyanion hole to stabilize a developing negatively charged oxygen *via* multiple NCHB interactions. Indeed, in the crystal packing from an X-ray analysis of TOB-2 (see ESI, Fig. S4†), it was observed that the fluorine in a TOB molecule is positioned closely to another TOB-2's C(sp^3^)–H. These results agree well with our hypothesis that strong C(sp^3^)–H NCHB donors exist in the TOB compounds.

In the optimized structure of the complex A formed between benzyl alcohol and TOB-1, the TOB-1 catalyst interacts with the benzyl alcohol with multiple NCHBs as shown in Bader's Atoms-in-Molecules (AIM) analysis.^22^ The bond paths from the AIM analysis are shown in Fig. 1D. The electron density (*ρ*) and its Laplacian (∇^2^*ρ*) at the bond critical points are similar to those of typical NCHBs.^23^ Among the identified NCHB interactions, the C–H⋯O interactions were found to be stronger as indicated by the larger *ρ* values. The binding energy of complex A was found to be −5.1 kcal mol^−1^. NBO second-order perturbation [E(2)] energy^24^ analysis was also performed (Fig. 1E and F). The strongest interactions originate from two lone pairs (LP) of O_alcohol_ to two different C–H_TOB-1_(σ*) orbitals, consistent with the conclusion from the AIM analysis. These stabilization energies also correlate well with the corresponding interaction distances, which are generally shorter than the sum of van der Waals' radii. In comparison, no bond paths between the phosphonium cation and the benzyl alcohol were found in the AIM analysis. The NBO E(2) stabilization energy (0.24 kcal mol^−1^) from the interaction between the alcohol oxygen lone-pair of electrons and the phosphonium cation was also found to be very small. These data suggest that no significant interactions exist between the alcohol and the phosphonium cation. Based on these results, we conclude that the active site of the TOB catalysts is at the C(sp^3^)–Hs.

Kinetics studies were conducted (see ESI, Fig. S5–S7†) and the acetalization reaction was found to be of 1st order with respect to each of the reaction components. Thus, the trilateral complex B that is formed from TOB-1, 4-methylphenol, and dihydropyran 2 was studied computationally to unearth the origin of the O/S chemoselectivity in the acetalization. In complex B with a binding energy of −8.0 kcal mol^−1^ (also see ESI, Fig. S10–S11†), multiple C(sp^3^)–Hs preferentially interact with the oxygen atoms in 4-methylphenol and dihydropyran 2*via* NCHB on the basis of AIM analysis (Fig. 2A). It appears that the developing negative charge at the oxygen of alcohol is stabilized in the oxyanion hole consisting of a group of preorganized C(sp^3^)–Hs (also see Fig. 1C), and the reaction partners are aligned in close proximity and ready for the protonation of the olefin of 2. In contrast, NCHBs from TOB-1 interact with the arene instead of the sulfur of 4-methylthiophenol in the trilateral complex C with a binding energy of −5.6 kcal mol^−1^, which is relatively less favorable compared to that of 4-methylphenol (Fig. 2B). Only a weakly bound complex directly between TOB-1 and the sulfur atom of 4-methylthiophenol was found with a binding energy of −4.3 kcal mol^−1^ in the optimized structure (see ESI, Fig. S14†). The weaker binding with the sulfur can be attributed to the size mismatch with the C–Hs in the oxyanion hole of TOB-1 and/or mismatching hard-soft acid-base.^17^ So, the *S*–H might be less acidic and protonation of dihydropyran 2 could be comparably less favored, which is evidenced by the relatively smaller *ρ* (0.0114) and longer distance (2.62 Å) in the S–H⋯C(pyran) interaction of complex C (*vs. ρ* = 0.0170; O–H⋯C(pyran) distance = 2.40 Å in complex B). A similar system was also calculated using BF_3_ instead of TOB-1 (Fig. 2C). Although the Lewis acid BF_3_ can strongly bind with the oxygen/sulfur atom of alcohol/thiol or dihydropyran 2, it can only bind with one substrate at a moment to give the bilateral complexes D and E (see ESI, Fig. S15 and S16†).

**Fig. 2:**
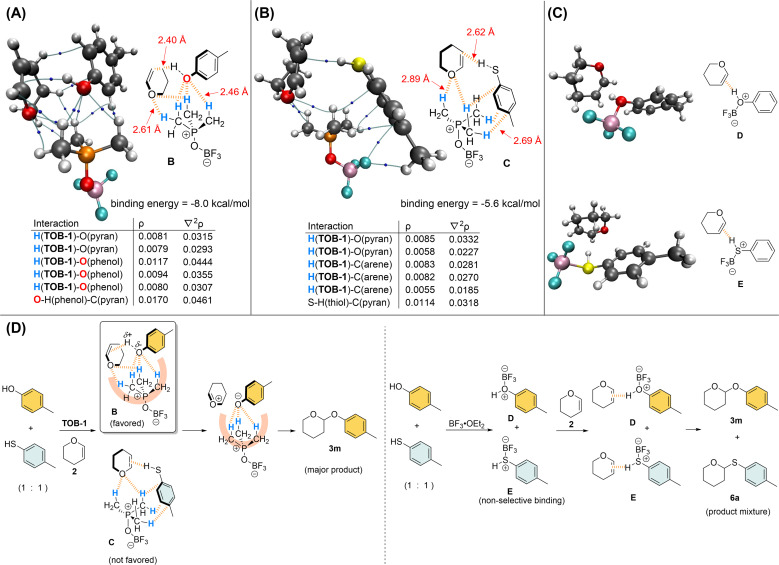
Studies on the mechanism of chemoselective acetalization. (A) AIM analysis on the trilateral complex of TOB-1/4-Me–C_6_H_4_–OH/2; (B) AIM analysis on the trilateral complex of TOB-1/4-Me–C_6_H_4_–SH/2; (C) interactions among BF_3_ and the substrates; (D) plausible mechanism of the chemoselective acetalization.

Based on these results, we believe that the TOB catalyst can effectively promote *O*-acetalization over *S*-acetalization because of the specific alignment of the oxygenated reactants in the oxyanion hole by multiple NCHBs in the favorable trilateral complex B. In contrast, the Lewis acid BF_3_ non-selectively binds with an alcohol and thiol to generate Brønsted acids D and E, which react with dihydropyran 2 to give *O*-acetal and *S*-acetal as a product mixture (Fig. 2D). Further calculations were carried out to study how the different solvents would influence the selectivity based on implicit SMD solvent models.^25^ We discovered a positive correlation between the tendency of the solvent to act as a hydrogen-bond donor and the distance between the OH in 4-methylphenol and C in pyran (see ESI, Fig. S17 and S18†), whereas no such correlation was found between the SH in 4-methylthiophenol and C in pyran. These observations are consistent with the stronger preference of hydrogen bond interaction between chloroform molecules and the hard base oxygen than the softer base sulfur.^26^ A more detailed study is needed to reveal more molecular details of this solvent effect, but we believe that the interactions in the trilateral complex B might be interrupted in chloroform but not in complex C, leading to the preference of thioacetal formation.

## Conclusions

In summary, catalysis using newly designed TOB complexes in acetalization has been developed. The catalyst was found to be able to distinguish alcohols and thiols in the acetalization, which is otherwise difficult to achieve using typical acid catalysts. Mechanistic studies suggest that the C(sp^3^)–H oxyanion hole in the TOB catalysts is crucial for efficient and chemoselective reactions.

## Data availability

Data supporting the findings are provided within the article and in the ESI.† This includes experimental procedures, characterization data of all new compounds, NMR spectra, and data of computational studies. All data are also available from the authors upon request. Crystallographic data for compound TOB-2 was deposited within the Cambridge Structural Database and is freely available *via* the Cambridge Crystallographic Data Centre under CCDC number 2209696.

## Author contributions

V. M.-Y. L. performed the experimental works including catalyst synthesis and scope study, and mechanistic investigation. H.-C. F. W. performed the computational studies. C.-M. P. assisted in the scope study on some entries. Y.-L. S. T. and Y.-Y. Y. supervised the research. V. M.-Y. L., H.-C. F. W., Y.-L. S. T., and Y.-Y. Y. wrote the manuscript.

## Conflicts of interest

There are no conflicts to declare.

## Supplementary Material

SC-014-D3SC03081D-s001

SC-014-D3SC03081D-s002
