# Supplementary material for: Trialkylphosphonium oxoborates as C(sp3)–H oxyanion holes and their application in catalytic chemoselective acetalization
Source: Chem Sci. 2023 Oct 20;14(44):12684–92. doi: 10.1039/d3sc03081d (PMC10646966; doi:10.1039/d3sc03081d)

Electronic Supplementary Material (ESI) for Chemical Science.
This journal is © The Royal Society of Chemistry 2023

Electronic Supplementary Information

Trialkylphosphonium Oxoborates as C(sp^3)-H Oxyanion Hole and Their Application in Catalytic Chemoselective Acetalization

Vincent Ming-Yau Leung, Hong-Chai Fabio Wong, Chun-Man Pook, Ying-Lung Steve Tse,* Ying-Yeung Yeung*

Department of Chemistry and State Key Laboratory of Synthetic Chemistry, The Chinese University of Hong Kong, Shatin, NT, Hong Kong

Table of Contents

S2	General information
S3-S4	Experimental procedures
S5-S26	Physical data
S27	Catalyst stability test
S28	Solvent screening and temperature effect on the chemoselectivity
S29	NMR titration experiment with TOB-1 and benzyl alcohol
S30	Comparison of the chemical shift of alcohols and thiols
S31-S37	X-ray crystal data of TOB-2
S38-S41	Kinetic study of the acetalization reaction
S42-S65	Computational studies
S66	References
S67-S211	NMR spectra

1. General information

Commercially available reagents were used as received. The solvents were dried over a solvent purification system from Innovative Technology. Nuclear magnetic resonance (NMR) spectra were recorded on a Bruker AMX500 (500 MHz) spectrometer or a Bruker AMX400 (400 MHz) spectrometer, in $CDCl_3$ solutions, unless stated otherwise. All chemical shifts were recorded in ppm downfield from tetramethylsilane (stated otherwise. All chemical shifts were recor3 at $\delta = 7.26$) or carbon signals in NMR solvent ($CDCl_3$ at $\delta = 77.16$). Spin-spin coupling constants (J value) recorded in Hz were measured directly from the spectra. High resolution mass spectra were obtained on a Thermo Q Exactive Focus Orbitrap with ionization mode Electrospray Ionization (Positive) or Atmospheric-pressure Chemical Ionization at the Department of Chemistry, The Chinese University of Hong Kong, Hong Kong Special Administrative Region. Analytical thin layer chromatography (TLC) was performed with Merck pre-coated TLC plates, silica gel 60F-254, layer thickness 0.25 mm. Flash chromatography separations were performed on Merck 60 (0.040-0.063 mm) mesh silica gel.

2. Experimental procedures

(i) Preparation of catalyst

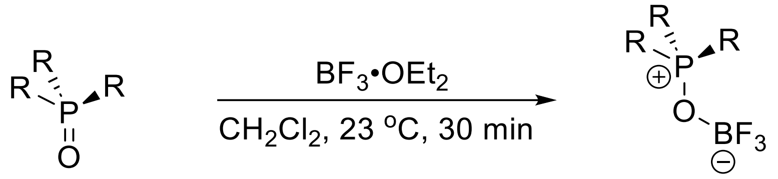

1a, R = Me
1b, R = cyclohexyl
1c, R = Ph

TOB-1, R = Me (84%)
TOB-2, R = cyclohexyl (91%)
TOB-3, R = Ph (95%)

General procedure: To a round bottom flask equipped with a magnetic stirrer bar was added trialkylphosphine oxide **1** (2 mmol) and anhydrous CH_2Cl_2 (1 mL) at 23 °C under inert atmosphere. Boron trifluoride diethyl etherate (0.54 mL, 4 mmol, 2 equiv) was added dropwise using syringe and the resultant mixture was stirred for 30 minutes. Next, diethyl ether was added, and white precipitate was formed. The solution was filtered, and the residue was washed with diethyl ether (5 mL). The residue was dried under high vacuum to give the **TOB** catalyst.

(ii) TOB-catalyzed acetalization reaction

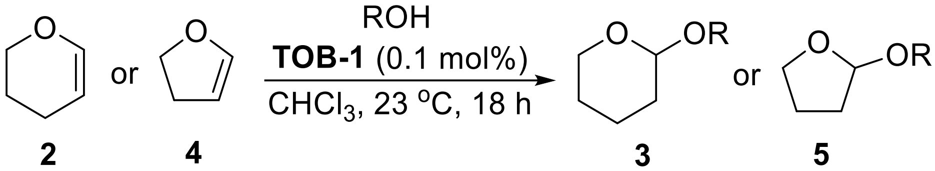

2 **4** **3** **5**

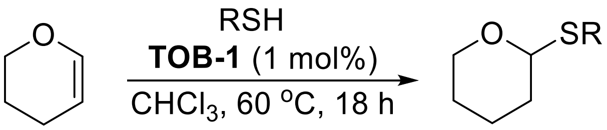

2 **6**

General procedure: To a solution of alcohol or thiol (0.2 mmol, 1 equiv) and **TOB-1** (0.2 µmol, 0.1 mol%) in $CHCl_3$ (0.4 mL) was added 3,4-dihydro-2H-pyran (**2**) (21.9 µL, 0.24 mmol, 1.2 equiv) or 2,3-dihydrofuran (**4**) (18.1 µL, 0.24 mmol, 2 equiv) into a vial (or a resealable tube for reactions at 60 °C). The reaction was stirred at 23 °C (or 60 °C) for 18 hours (or otherwise specified). The solution was concentrated under reduced pressure and the residue was purified by flash column chromatography eluted with hexanes/Et_2O (90:10) to give the desired acetal product.

(iii) Scale-up reaction

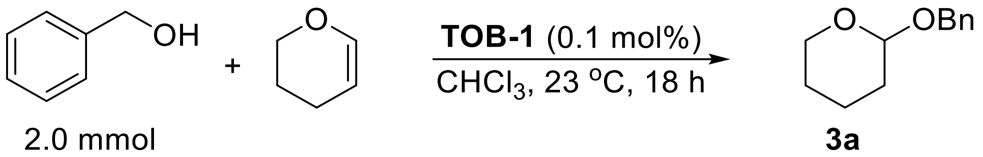

To a solution of benzyl alcohol (207 µL, 2.0 mmol, 1 equiv) and **TOB-1** (2.0 µmol, 0.1 mol%) in $CHCl_3$ (4.0 mL) was added 3,4-dihydropyran **2** (219 µL, 2.4 mmol, 1.2 equiv). The reaction was stirred at 23 °C for 18 hours. The solution was concentrated under reduced pressure and the residue was purified by flash column chromatography eluted with hexanes/Et_2O = 90:10 to give the desired acetal product **3a** (0.31 g, 1.63 mmol, 81% yield).

(iv) O/S Chemoselective acetalization

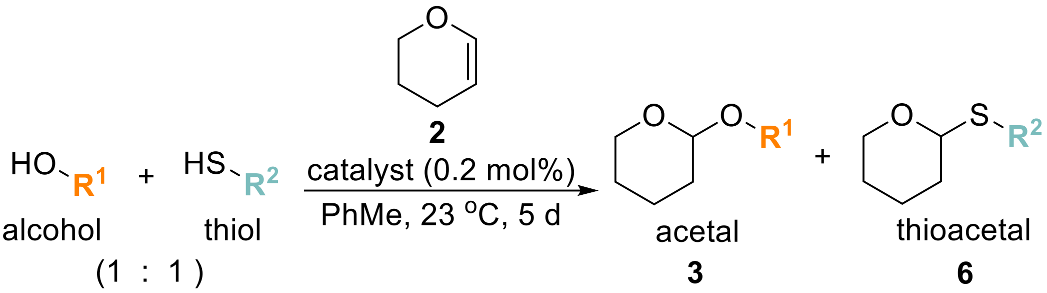

To a solution of alcohol (0.2 mmol, 1 equiv), thiol (0.2 mmol, 1 equiv) and **TOB-1** (0.4 µmol, 0.2 mol%) in toluene (0.4 mL) was added 3,4-dihydro-2H-pyran (**2**) (18.1 µL, 0.2 mmol, 1 equiv) into a vial. The reaction was stirred at 23 °C for 5 days. The reaction mixture was then filtered through a thin plug of silica gel column and the filtrate was concentrated under reduced pressure. The reaction yield and ratio were determined using NMR with dibromomethane (14.0 µL, 0.2 mmol, 1 equiv) as the internal standard.

3. Physical data

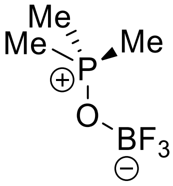

TOB-1

84% yield. White solid. mp 154.7-156.1 °C. ^{1}H NMR (500 MHz, CDCl$_3$): δ 1.89 (d, J = 13.5 Hz, 9H). ^{13}C NMR (125 MHz, CDCl$_3$): δ 14.8 (d, J = 71.3 Hz). ^{11}B NMR (160 MHz, CDCl$_3$): δ -0.93. ^{19}F NMR (470 MHz, CDCl$_3$): δ -146.0 (s, 3F). ^{31}P NMR (200 MHz, CDCl$_3$): δ 67.9 (m). FTICR MS (APCI) calcd for [$C_3H_9BF_3OP$ + Cl]$^-$: 195.01308, found: 195.01285.

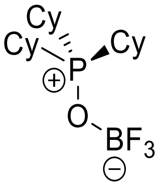

TOB-2

91% yield. White solid. mp 204.2-206.9 °C. ^{1}H NMR (400 MHz, CDCl$_3$): δ 2.23 (qt, J = 12.5 Hz, 2.7 Hz, 3H), 2.00-1.97 (m, 6H), 1.93-1.88 (m, 6H), 1.78 (m, 3H), 1.60-1.51 (m, 6H), 1.36-1.23 (m, 9H). ^{13}C NMR (125 MHz, CDCl$_3$): δ 34.1, 33.6, 26.7 (d, J = 12.5 Hz), 25.8 (d, J = 11.3 Hz). ^{11}B NMR (160 MHz, CDCl$_3$): δ -1.03. ^{19}F NMR (470 MHz, CDCl$_3$): δ -145.9 (s, 3F). ^{31}P NMR (200 MHz, CDCl$_3$): δ 70.1 (m). FTICR MS (APCI) calcd for [$C_{18}H_{33}BF_3OP$ + Cl]$^-$: 399.20115, found: 399.20146.

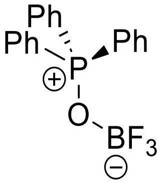

TOB-3[1]

95% yield. White solid. mp 243.5-244.3 °C. ^{1}H NMR (500 MHz, CDCl$_3$): δ 7.77-7.70 (m, 9H), 7.60-7.56 (m, 6H). ^{13}C NMR (125 MHz, CDCl$_3$): δ 134.4 (d, J= 2.7 Hz), 133.2 (d, J= 11.7 Hz), 129.4 (d, J= 13.3 Hz), 124.9 (d, J= 111.6 Hz). ^{11}B NMR (160 MHz, CDCl$_3$): δ -0.46. ^{19}F NMR (470 MHz, CDCl$_3$): δ -144.1 (s, 3F). ^{31}P NMR (200 MHz, CDCl$_3$): δ 44.5 (s).

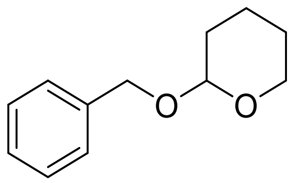

3a

Colorless liquid. 93% Isolated yield. ^{1}H NMR (500 MHz, $CDCl_3$): δ 7.40-7.26 (m, 5H), 4.81 (d, J = 15 Hz, 1H), 4.73 (t, J = 4.4 Hz, 1H), 4.52 (d, J = 15 Hz, 1H), 3.97-3.91 (m, 1H), 3.59-3.54 (m, 1H), 1.93-1.84 (m, 1H), 1.79-1.52 (m, 5H). ^{13}C NMR (125 MHz, $CDCl_3$): δ 138.4, 128.5, 127.9, 127.6, 97.8, 68.9, 62.2, 30.7, 25.6, 19.5. QEFMS calcd for $[C_{12}H_{16}O_2Na]^+$: 215.10425, found: 215.10390.

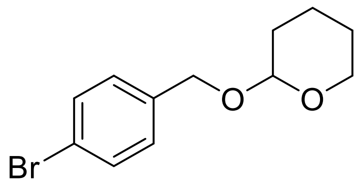

3b

Colorless liquid. 99% Isolated yield. ^{1}H NMR (500 MHz, $CDCl_3$): δ 7.46 (d, J = 8.6 Hz, 2H), 7.24 (d, J = 8.4 Hz, 2H), 4.73 (d, J = 12.3 Hz, 1H), 4.69 (t, J = 3.5 Hz, 1H), 4.45 (d, J = 12.3Hz, 1H), 3.91-3.87 (m, 1H), 3.56-3.52 (m, 1H) 1.89-1.50 (m, 6H). ^{13}C NMR (125 MHz, $CDCl_3$): δ 137.4, 131.1, 129.5, 121.4, 97.9, 68.1, 62.2, 30.6, 25.5, 19.4. QEFMS calcd for $[C_{12}H_{15}BrO_2Na]^+$: 293.01476, found: 293.01405.

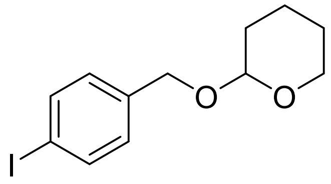

3c

Colorless liquid. 91% Isolated yield. ^{1}H NMR (500 MHz, $CDCl_3$): δ 7.68-7.65 (m, 2H), 7.11-7.09 (m, 2H), 4.71 (d, J = 12.3 Hz, 1H), 4.68 (t, J = 3.6 Hz, 1H), 4.45 (d, J = 12.4 Hz, 1H), 3.90-3.86 (m, 1H), 3.56-3.52 (m, 1H) 1.87-1.51 (m, 6H). ^{13}C NMR (125 MHz, $CDCl_3$): δ 138.1, 137.5, 129.7, 128.7, 97.9, 68.2, 62.3, 30.6, 25.5, 19.4. QEFMS calcd for $[C_{12}H_{15}IO_2Na]^+$: 341.00089, found: 341.00039.

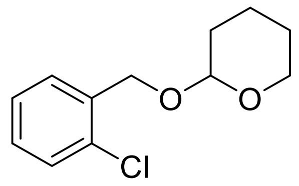

3d

Colorless liquid. 99% Isolated yield. ^{1}H NMR (500 MHz, $CDCl_3$): *δ* 7.52 (d, *J* = 7.5 Hz, 1H), 7.34 (d, J = 7.8 Hz, 1H), 7.24 (dt, *J* = 25.7 Hz, 7.8 Hz, 2H) , 4.86 (d, *J* = 13.2 Hz, 1H), 4.77 (s, 1H), 4.60 (d, *J* = 13.2 Hz, 1H), 3.92 (t, *J* = 10.6 Hz, 1H), 3.57-3.55 (m, 1H), 1.90-1.55 (m, 6H). ^{13}C NMR (125 MHz, $CDCl_3$): *δ* 136.3, 133.0, 129.3, 129.0, 128.6, 126.8, 98.4, 66.4, 62.2, 30.6, 25.5, 19.4. QEFMS calcd for $[C_{12}H_{15}ClO_2Na]^+$: 249.06528, found: 249.06500.

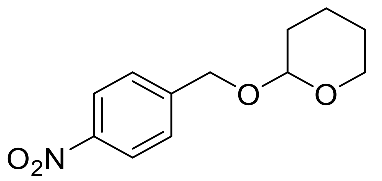

3e

Colorless liquid. 88% Isolated yield. ^{1}H NMR (500 MHz, $CDCl_3$): *δ* 8.19 (d, J = 8.8 Hz, 2H), 7.52 (d, J = 8.8 Hz, 2H), 4.87 (d, *J* = 13.5 Hz, 1H), 4.72 (t, *J* = 3.5 Hz, 1H), 4.59 (d, *J* = 13.5 Hz, 1H), 3.89-3.85 (m, 1H), 3.57-3.53 (m, 1H), 1.89-1.53 (m, 6H). ^{13}C NMR (125 MHz, $CDCl_3$): *δ* 146.2, 127.9, 127.1, 123.7, 98.4, 67.7, 62.4, 30.5, 25.4, 19.4. QEFMS calcd for $[C_{12}H_{15}NO_4Na]^+$: 260.08933, found: 260.08915.

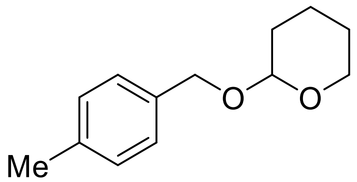

3f

Colorless liquid. 93% Isolated yield. ^{1}H NMR (500 MHz, $CDCl_3$): *δ* 7.27(d, J = 8 Hz, 2H), 7.17 (d, J = 7.8 Hz, 2H), 4.76 (d, *J* = 11.8 Hz, 1H), 4.71 (t, *J* = 3.6 Hz, 1H), 4.48 (d, *J* = 11.8 Hz, 1H), 3.96-3.92 (m, 1H), 3.58-3.54 (m, 1H), 2.36 (s, 3H) 1.92-1.50 (m, 6H). ^{13}C NMR (125 MHz, $CDCl_3$): *δ* 137.3, 135.3, 129.1, 128.1, 97.6, 68.8, 62.2, 30.7, 25.6, 21.3, 19.5. QEFMS calcd for $[C_{13}H_{18}O_2Na]^+$: 229.11990, found: 229.11954

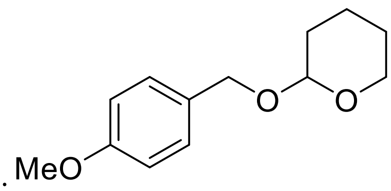

3g

Colorless liquid. 92% Isolated yield. ^{1}H NMR (500 MHz, $CDCl_3$): δ 7.30 (d, J = 8.7 Hz, 2H), 6.88 (d, J = 8.7 Hz, 2H), 4.72 (d, *J* = 11.6 Hz, 1H), 4.69 (t, *J* = 3.6 Hz, 1H), 4.44 (d, *J* = 11.6 Hz, 1H), 3.95-3.90 (m, 1H), 3.8 (s, 3H), 3.55 (m, 1H), 1.88-1.50 (m, 6H). ^{13}C NMR (125 MHz, $CDCl_3$): δ 159.2, 130.4, 129.6, 113.9, 97.6, 68.6, 62.3, 55.4, 30.7, 25.6, 19.5. QEFMS calcd for $[C_{13}H_{18}O_3Na]^+$: 245.11482, found: 229.11456.

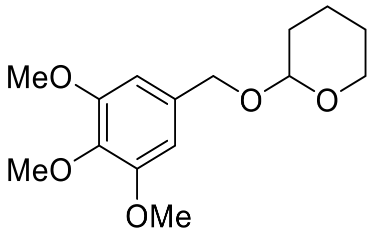

3h

Colorless liquid. 93% Isolated yield. ^{1}H NMR (500 MHz, $CDCl_3$): δ 6.60 (d, J = 3.7 Hz, 2H), 4.72 (d, *J* = 11.9 Hz, 1H), 4.70 (t, *J* = 3.7 Hz, 1H), 4.43 (d, *J* = 10.7 Hz, 1H), 3.96-3.91 (m, 1H), 3.86 (s, 6H), 3.83 (s, 3H), 3.58-3.54 (m, 1H), 1.90-1.52 (m, 6H). ^{13}C NMR (125 MHz, $CDCl_3$): δ 153.4, 137.4, 134.0, 105.0, 97.9, 69.2, 62.5, 61.0, 56.2, 30.8, 25.6, 19.7. QEFMS calcd for $[C_{15}H_{22}O_5Na]^+$: 305.13594, found: 305.13538.

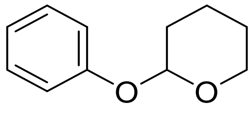

3i

Colorless liquid. 98% Isolated yield. ^{1}H NMR (500 MHz, $CDCl_3$): δ 7.29 (t, *J* = 7.3 Hz, 2H), 7.07 (d, J = 7.9 Hz, 2H), 7.00 (t, *J* = 7.3 Hz, 1H), 5.43 (s, 1H), 3.93 (t, *J* = 11.3 Hz, 1H), 3.63-3.60 (m, 1H), 2.06-1.56 (m, 6H). ^{13}C NMR (125 MHz, $CDCl_3$): δ 157.2, 129.5, 121.7, 116.6, 96.4, 62.1, 30.5, 25.3, 18.9. QEFMS calcd for $[C_{11}H_{14}O_2Na]^+$: 201.08819, found: 201.08858.

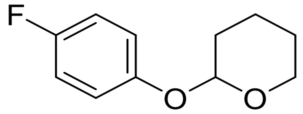

3j

Colorless liquid. 95% Isolated yield. ^{1}H NMR (500 MHz, $CDCl_3$): δ 7.01-6.94 (m, 4H), 5.32 (t, J=3.29 1H), 3.93-3.88 (m, 1H), 3.62-3.58 (m, 1H), 2.07-1.93 (m, 1H), 1.89-1.80 (m, 2H), 1.72-1.58 (m, 3H). ^{13}C NMR (125 MHz, $CDCl_3$): δ 157.9 (d, J_{CF} = 237.5 Hz), 153.3 (d, J_{CF} = 3.75 Hz), 117.9 (d, J_{CF} = 7.5 Hz), 115.9 (d, J_{CF} = 22.5 Hz), 97.2, 62.2, 30.5, 25.3, 18.9. ^{19}F NMR (470 MHz, $CDCl_3$): δ -122.9 (sept, J_{CF} = 4.7 Hz). QEFMS calcd for $[C_{11}H_{13}FO_2Na]^+$: 219.07918, found: 219.07886.

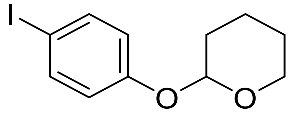

3k

Colorless liquid. 95% Isolated yield. ^{1}H NMR (500 MHz, $CDCl_3$): δ 7.55 (d, J = 7.8 Hz, 2H), 6.83 (d, J =7.8 Hz, 2H), 5.38 (s, 1H), 3.86 (t, J = 11.2 Hz, 1H), 3.60-3.58 (m, 1H), 2.00-1.58 (m, 6H). ^{13}C NMR (125 MHz, $CDCl_3$): δ 157.0, 138.4, 118.9, 96.4, 84.0, 62.1, 30.3, 25.2, 18.7. QEFMS calcd for $[C_{11}H_{13}IO_2Na]^+$: 326.98524, found: 326.98498.

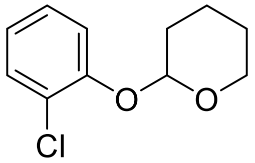

3l

Colorless liquid. 83% Isolated yield. ^{1}H NMR (400 MHz, $CDCl_3$): δ 7.37-7.35 (m, 1H), 7.19-7.18 (m, 2H), 6.95-6.89 (m, 1H), 5.50 (t, J = 2.9 Hz, 1H), 3.96-3.90 (m, 1H), 3.64-3.59 (m, 1H), 2.15-2.04 (m, 1H), 2.00-1.95 (m, 1H), 1.92-1.84 (m, 1H), 1.77-1.61 (m, 3H). ^{13}C NMR (100 MHz, $CDCl_3$): δ 152.7, 130.3, 127.7, 124.0, 122.5, 117.1, 96.9, 62.0, 30.3, 25.3, 18.9. QEFMS calcd for $[C_{11}H_{13}ClO_2Na]^+$: 235.04963, found: 235.04948.

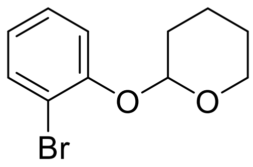

3m

Colorless liquid. 74% Isolated yield. ^{1}H NMR (500 MHz, $CDCl_3$): δ 7.24 (d, J = 12.4 Hz, 1H), 7.13-7.12 (m, 2H), 6.98 (d, J =7.6 Hz, 1H), 5.40 (t, J = 2.8 Hz, 1H), 3.87 (td, J = 10.6 Hz, 2.8Hz, 1H), 3.63-3.59 (m, 1H), 1.99-1.57 (m, 6H). ^{13}C NMR (125 MHz, $CDCl_3$): δ 158.0, 130.6, 124.8, 122.7, 120.0, 115.3, 96.9, 62.1, 30.3, 25.2, 18.7. QEFMS calcd for $[C_{11}H_{13}BrO_2Na]^+$: 278.99911, found: 278.99906.

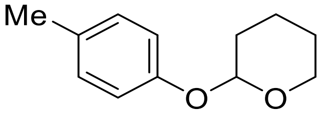

3n

Colorless liquid. 82% Isolated yield. ^{1}H NMR (500 MHz, $CDCl_3$): δ 7.08 (d, J = 8.3 Hz, 2H), 6.95 (d, J = 8.6 Hz, 2H), 5.37 (t, J = 3.3 Hz, 1H), 3.94-3.89 (m, 1H), 3.61-3.57 (m, 1H), 2.28 (s, 3H), 2.05-1.96 (m, 1H), 1.87-1.83 (m, 2H), 1.71-1.57 (m, 3H). ^{13}C NMR (125 MHz, $CDCl_3$): δ 155.0, 131.0, 130.0, 116.5, 96.7, 62.2, 30.6, 25.4, 20.7, 19.0. QEFMS calcd for $[C_{12}H_{16}O_2Na]^+$: 215.10425, found: 215.10387.

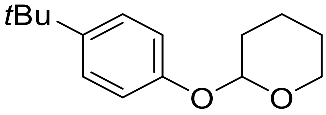

3o

Colorless liquid. 89% Isolated yield. ^{1}H NMR (500 MHz, $CDCl_3$): δ 7.31 (d, J = 8.7 Hz, 2H), 7.0 (d, J = 8.7 Hz, 2H), 5.41 (s, 1H), 3.95 (t, J = 9.9 Hz, 1H), 3.62-3.60 (m, 1H), 2.03-1.60 (m, 6H), 1.31 (s, 9H). ^{13}C NMR (125 MHz, $CDCl_3$): δ 154.9, 144.3, 126.3, 116.0, 96.5, 62.1, 34.2, 31.6, 30.6, 25.3, 19.0. QEFMS calcd for $[C_{15}H_{22}O_2Na]^+$: 257.15120, found: 257.15080.

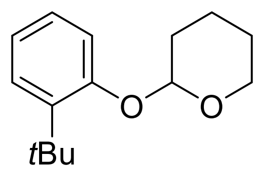

3p

Colorless liquid. 92% Isolated yield. ^{1}H NMR (400 MHz, $CDCl_3$): δ 7.31-7.29 (m, 1H), 7.21-7.14 (m, 2H), 6.94-6.90 (m, 1H), 5.49 (t, J = 2.9 Hz, 1H), 3.95-3.89 (m, 1H), 3.69-3.64 (m, 1H), 2.10-2.01 (m, 1H), 1.95-1.90 (m, 2H), 1.89-1.69 (m, 2H), 1.67-1.61 (m, 1H), 1.43 (s, 9H). ^{13}C NMR (100 MHz, $CDCl_3$): δ 156.2, 138.0, 127.3, 126.7, 121.0, 114.4, 96.2, 61.9, 35.0, 30.7, 30.1, 25.5, 19.1. QEFMS calcd for $[C_{15}H_{22}O_2Na]^+$: 257.15120, found: 257.15090.

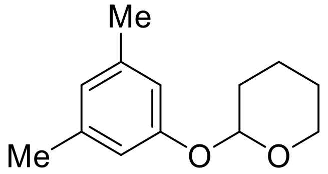

3q

Colorless liquid. 93% Isolated yield. ^{1}H NMR (500 MHz, $CDCl_3$): δ 6.70 (s, 2H), 6.65 (s, 1H), 5.42 (t, J = 3.2 Hz, 1H), 3.94 (td, J = 9.8 Hz, 3.1 Hz, 1H), 3.63-3.60 (m, 1H), 2.30 (s, 6H) 2.04-1.56 (m, 6H). ^{13}C NMR (125 MHz, $CDCl_3$): δ 157.2, 139.2, 123.5, 114.3, 96.3, 62.1, 30.6, 25.4, 21.5, 18.9. QEFMS calcd for $[C_{13}H_{18}O_2Na]^+$: 229.11990, found: 229.11988.

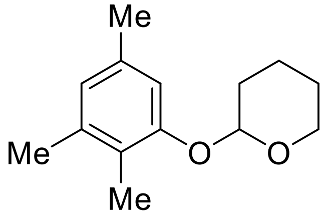

3r

Colorless liquid. 71% Isolated yield. ^{1}H NMR (500 MHz, $CDCl_3$): δ 6.80 (s, 1H), 6.66 (s, 1H), 5.40 (s, 1H), 3.92 (td, J = 9.9 Hz, 2.6 Hz, 1H), 3.64-3.60 (m, 1H), 2.28 (s, 3H), 2.25 (s, 3H), 2.16 (s, 3H), 2.06-2.01 (m, 1H), 1.90-1.88 (m, 2H), 1.74-1.61 (m, 3H). ^{13}C NMR (125 MHz, $CDCl_3$): δ 154.9, 137.6, 135.6, 124.0, 122.7, 112.9, 96.4, 62.1, 30.8, 25.5, 21.4, 20.2, 19.1, 11.6. QEFMS calcd for $[C_{14}H_{20}O_2Na]^+$: 243.13555, found: 243.13525.

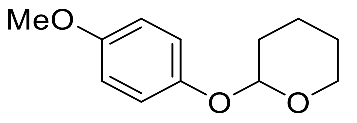

3s

Colorless liquid. 99% Isolated yield. ^{1}H NMR (500 MHz, $CDCl_3$): δ 7.00 (d, J = 7.9 Hz, 2H), 6.82 (d, J = 7.9 Hz, 2H), 5.30 (s, 1H), 3.94 (t, J = 11.1 Hz, 1H), 3.76 (s, 3H), 3.60-3.58 (m, 1H), 2.00-1.55 (m, 6H). ^{13}C NMR (125 MHz, $CDCl_3$): δ 154.6, 151.2, 117.9, 114.6, 97.4, 62.2, 55.7, 30.6, 25.3, 19.0. QEFMS calcd for $[C_{12}H_{16}O_3Na]^+$: 231.09917, found: 231.09923.

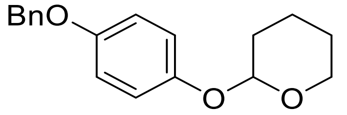

3t

Colorless liquid. 86% Isolated yield. ^{1}H NMR (500 MHz, $CDCl_3$): δ 7.45 (d, J = 7.5 Hz, 2H), 7.40 (t, J = 7.3 Hz, 2H), 7.33 (t, J = 7.2 Hz, 1H), 7.02 (d, J = 9.0 Hz, 2H), 6.92 (d, J = 9.0 Hz, 2H), 5.32 (t, J = 3.1 Hz, 1H), 5.03 (s, 2H) 3.96 (td, J = 12.0 Hz, 2.9 Hz, 1H), 3.63-3.59 (m, 1H), 2.07-1.60 (m, 6H). ^{13}C NMR (125 MHz, $CDCl_3$): δ 153.8, 151.4, 137.4, 128.6, 128.0, 127.6, 117.8, 115.7, 97.4, 70.6, 62.2, 30.6, 25.4, 19.0. QEFMS calcd for $[C_{18}H_{20}O_3Na]^+$: 307.13047, found: 307.13000.

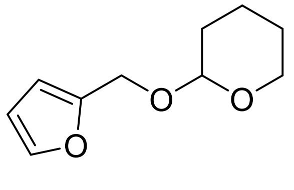

3u

Colorless liquid. 70% Isolated yield. ^{1}H NMR (500 MHz, $CDCl_3$): δ 7.41 (m, 1H), 6.33 (d, J = 2.5 Hz, 2H), 4.71 (m, 1H), 4.67 (d, J = 12.8 Hz, 1H), 4.49 (d, J = 12.8 Hz, 1H) 3.90 (t, J = 10.2 Hz, 1H), 3.55 (m, 1H), 1.87-1.80 (m, 1H), 1.74-1.69 (m, 1H), 1.63-1.59 (m, 2H), 1.54-1.52 (m, 2H) . ^{13}C NMR (125 MHz, $CDCl_3$): δ 151.9, 142.9, 110.4, 109.4, 97.4, 62.1, 60.7, 30.5, 25.5., 19.3. QEFMS calcd for $[C_{10}H_{14}O_3Na]^+$: 205.08352, found: 205.08313.

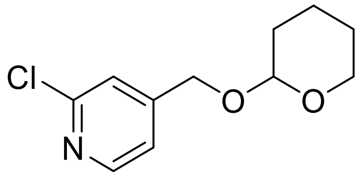

3v

Colorless liquid. 65% Isolated yield. ^{1}H NMR (400 MHz, $CDCl_3$): δ 8.36 (d, J = 2.1 Hz, 1H), 7.66 (dd, J = 8.2, 2.4 Hz, 1H), 7.30 (d, J = 8.1 Hz, 1H), 4.76 (d, J = 12.4 Hz, 1H), 4.67 (t, J = 3.4 Hz, 1H), 4.48 (d, J = 12.4 Hz, 1H), 3.88-3.82 (m, 1H), 3.56-3.51 (m, 1H), 1.87-1.70 (m, 2H), 1.66-1.52 (m, 4H). ^{13}C NMR (100 MHz, $CDCl_3$): δ 150.7, 149.1, 138.5, 132.9, 124.1, 98.3, 65.7, 62.4, 30.5, 25.4, 19.3. QEFMS calcd for $[C_{11}H_{14}NO_2Cl+H]^+$: 228.07858, found: 228.07822.

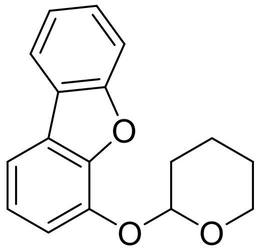

3w

Colorless liquid. 69% Isolated yield. ^{1}H NMR (400 MHz, $CDCl_3$): δ 7.92 (dd, J = 7.6, 0.5 Hz, 1H), 7.62 (d, J = 8.2 Hz, 1H), 7.59 (d, J = 4.4 Hz, 1H), 7.45 (td, J = 7.4, 1.3 Hz, 1H), 7.33 (td, J = 7.7, 0.8 Hz, 1H), 7.25 (d, J = 4.1 Hz, 2H), 5.74 (t, J = 3.1 Hz, 1H), 4.03 (td, J = 11.4, 3.0 Hz, 1H), 3.64 (dtd, J = 11.2, 3.8, 1.1 Hz, 1H), 2.22-2.05 (m, 2H), 2.01-1.93 (m, 1H), 1.79-1.63 (m, 3H). ^{13}C NMR (100 MHz, $CDCl_3$): δ 156.3, 146.2, 142.7, 127.2, 126.2, 124.6, 123.5, 122.8, 120.8, 114.7, 114.0, 112.1, 97.5, 62.2, 30.4, 25.3, 18.7. QEFMS calcd for $[C_{17}H_{16}O_3Na]^+$: 291.09917, found: 291.09847.

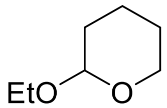

3x

Colorless liquid. 99% Isolated yield. ^{1}H NMR (400 MHz, $CDCl_3$): δ 4.57 (dd, J = 2.7, 4.5 Hz, 1H), 3.89-3.83 (m, 1H), 3.81-3.77 (m, 1H), 3.51-3.40 (m, 2H), 1.88-1.76 (m, 1H), 1.74-1.66 (m, 1H), 1.60-1.48 (m, 4H), 1.21 (t, J = 7.0 Hz, 3H). ^{13}C NMR (100 MHz, $CDCl_3$): δ 98.8, 63.0, 62.6, 30.9, 25.6, 19.9, 15.3. QEFMS calcd for $[C_7H_{14}O_2Na]^+$: 153.08860, found: 183.08840.

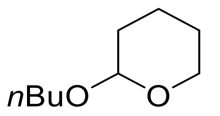

3y

Colorless liquid. 99% Isolated yield. ^{1}H NMR (400 MHz, $CDCl_3$): δ 4.56 (t, J = 2.6 Hz, 1H), 3.88-3.83 (m, 1H), 3.72 (dt, J = 9.6 Hz, 6.8 Hz, 1H), 3.51-3.46 (m, 1H), 3.37 (dt, J = 9.6 Hz, 6.8 Hz, 1H), 1.85-1.77 (m, 1H), 1.73-1.67 (m, 1H), 1.60-1.47 (m, 6H), 1.42-1.33 (m, 2H), 0.91 (t, J = 7.4 Hz, 3H). ^{13}C NMR (100 MHz, $CDCl_3$): δ 98.9, 67.5, 62.4, 32.0, 30.9, 25.6, 19.8, 19.6, 14.0. QEFMS calcd for $[C_9H_{18}O_2Na]^+$: 181.1199, found: 181.1197.

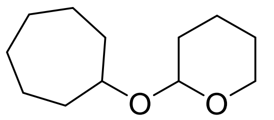

3z

Colorless liquid. 90% Isolated yield. ^{1}H NMR (500 MHz, $CDCl_3$): δ 4.64 (t, J = 3.7 1H), 3.90 (sept, J = 3.6 Hz, 1H), 3.78 (sept, J = 4.2 Hz, 1H), 1.98-1.93 (m, 1H), 1.86-1.81 (m, 2H), 1.72-1.61 (m, 5H), 1.59-1.49 (m, 9H), 1.42-1.34 (m, 2H). ^{13}C NMR (125 MHz, $CDCl_3$): δ 96.9, 76.8, 62.9, 35.8, 33.4, 31.5, 28.4, 28.2, 25.7, 23.3, 23.0, 20.2. QEFMS calcd for $[C_{12}H_{22}O_2Na]^+$: 221.15120, found: 221.15098.

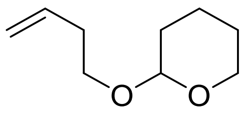

3aa

Colorless liquid. 70% Isolated yield. ^{1}H NMR (500 MHz, $CDCl_3$): δ 5.88-5.80 (m, 1H), 5.10 (d, J = 17.2 Hz, 1H), 5.03 (d, J = 10.3 Hz, 1H), 4.59 (t, J = 3.5 Hz, 1H), 3.86 (t, J = 9.3 Hz, 1H), 3.78 (q, J = 7.1 Hz, 1H), 3.52-3.43 (m, 2H), 5.10 (q, J = 17 Hz, 2H), 1.85-1.79 (m, 1H), 1.74-1.68 (m, 1H), 1.61-1.50 (m, 4H). ^{13}C NMR (125 MHz, $CDCl_3$): δ 135.4, 116.4, 98.9, 66.9, 62.4, 34.3, 30.8, 25.6, 19.7. QEFMS calcd for $[C_9H_{16}O_2Na]^+$: 179.10425, found: 179.10409.

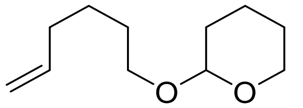

3ab

Colorless liquid. 92% Isolated yield. ^{1}H NMR (500 MHz, $CDCl_3$): δ 5.80 (sext, J = 8.1 Hz, 1H), 4.99 (d, J = 17.2 Hz, 1H), 4.93 (d, J = 10.2 Hz, 1H), 4.56 (s, 1H), 3.85 (t, J = 9.5 Hz, 1H), 3.73 (q, J = 7.7 Hz, 1H), 3.48 (t, J = 5.5 Hz, 1H), 3.37 (q, J = 7.5 Hz, 1H), 2.07 (q, J = 7.1 Hz, 2H), 1.81 (q, J = 8.8 Hz, 1H), 1.69 (t, J = 11.1 Hz, 1H), 1.63-1.45 (m, 9H). ^{13}C NMR (125 MHz, $CDCl_3$): δ 138.9, 114.6, 98.9, 67.5, 62.4, 33.7, 30.9, 29.3, 25.7, 25.6, 19.8. QEFMS calcd for $[C_{11}H_{20}O_2Na]^+$: 207.13555, found: 207.13535.

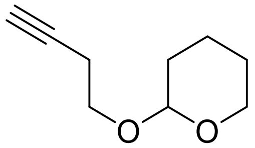

3ac

Colorless liquid. 94% Isolated yield. ^{1}H NMR (500 MHz, $CDCl_3$): δ 4.64 (t, J = 3.4 Hz, 1H), 3.89-3.80 (m, 2H), 3.58-3.48 (m, 2H), 2.48 (td, J = 7 Hz, 2.5 Hz, 2H), 1.97 (t, J = 2.4 Hz, 1H), 1.85-1.50 (m, 6H). ^{13}C NMR (125 MHz, $CDCl_3$): δ 98.9, 81.6, 69.3, 65.6, 62.3, 30.6, 25.5, 20.1, 19.5. QEFMS calcd for $[C_9H_{14}O_2Na]^+$: 177.08860, found: 177.08848.

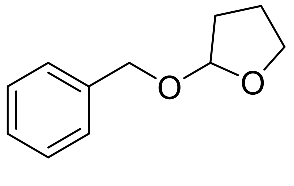

5a

Colorless liquid. 99% Isolated yield. ^{1}H NMR (500 MHz, $CDCl_3$): δ 7.38 (m, 4H), 7.26 (m, 1H), 5.23 (d, J = 4.4 Hz, 1H), 4.73 (d, J = 11.9 Hz, 1H), 4.49 (d, J = 11.9 Hz, 1H), 3.99-3.89 (m, 2H), 2.09-1.82 (m, 4H). ^{13}C NMR (125 MHz, $CDCl_3$): δ 138.4, 128.5, 128.0, 127.6, 103.2, 68.9, 67.1, 32.4, 23.6. QEFMS calcd for $[C_{11}H_{14}O_2Na]^+$: 201.08860, found: 201.08832.

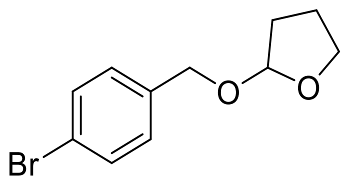

5b

Colorless liquid. 94% Isolated yield. ^{1}H NMR (500 MHz, $CDCl_3$): *δ* 7.45 (d, *J* = 7.6 Hz, 2H), 7.21 (d, *J* = 7.7 Hz, 2H), 5.19 (s, 1H), 4.65 (d, *J* = 12.2 Hz, 1H), 4.45 (d, *J* = 12.1 Hz, 1H), 3.91 (sext, *J* = 7.2 Hz, 2H), 2.05-1.81 (m, 4H). ^{13}C NMR (125 MHz, $CDCl_3$): *δ* 137.5, 131.5, 129.5, 121.4, 103.3, 68.1, 67.2, 32.5, 23.5. QEFMS calcd for $[C_{11}H_{13}BrO_2Na]^+$: 278.99911, found: 278.99890.

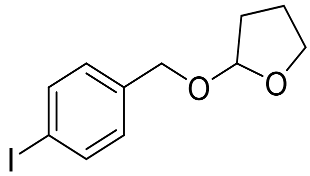

5c

Colorless liquid. 97% Isolated yield. ^{1}H NMR (500 MHz, $CDCl_3$): *δ* 7.66 (d, *J* = 7.5 Hz, 2H), 7.08 (d, *J* = 7.6 Hz, 2H), 5.19 (s, 1H), 4.64 (d, *J* = 12.2 Hz, 1H), 4.41 (d, *J* = 12.2 Hz, 1H), 3.91 (sext, *J* = 7.3 Hz, 2H), 2.05-1.80 (m, 4H). ^{13}C NMR (125 MHz, $CDCl_3$): *δ* 138.2, 137.5, 129.8, 103.3, 93.0, 68.1, 67.2, 32.4, 23.5. QEFMS calcd for $[C_{11}H_{13}IO_2Na]^+$: 326.98524, found: 326.98447.

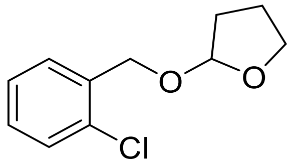

5d

Colorless liquid. 99% Isolated yield. ^{1}H NMR (500 MHz, $CDCl_3$): *δ* 7.46 (d, *J* = 7.4 Hz, 1H), 7.34 (d, J = 7.5 Hz, 1H), 7.28-7.19 (m, 2H), 5.26 (d, *J* = 4.1 Hz, 1H) 4.80 (d, *J* = 13.0 Hz, 1H), 4.57 (d, *J* = 13.0 Hz, 1H), 3.94 (dq, *J* = 5.1 Hz, 7 Hz, 2H), 2.08-1.85 (m, 4H). ^{13}C NMR (125 MHz, $CDCl_3$): *δ* 136.3, 133.2, 129.4, 129.3, 128.7, 126.8, 103.7, 67.3, 66.2, 32.5, 23.5. QEFMS calcd for $[C_{11}H_{13}ClO_2Na]^+$: 235.04963, found: 235.04924.

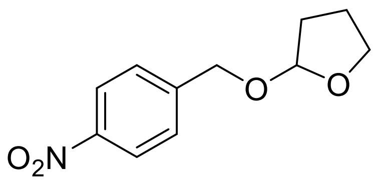

5e

Colorless liquid. 92% Isolated yield. ^{1}H NMR (500 MHz, $CDCl_3$): δ 8.17(d, J = 7.9 Hz, 2H), 7.48 (d, J = 7.9Hz, 2H), 5.21 (s, 1H), 4.79 (d, J = 13.5 Hz, 1H), 4.56 (d, J = 13.4 Hz, 1H), 3.91 (sext, J= 7.5 Hz, 2H), 2.07-1.87 (m, 4H). ^{13}C NMR (125 MHz, $CDCl_3$): δ 147.3, 146.4, 127.9, 123.6, 103.8, 67.6, 67.4, 32.5, 23.5. QEFMS calcd for $[C_{11}H_{13}NO_4Na]^+$: 246.07368, found: 246.07359.

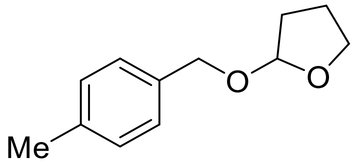

5f

Colorless liquid. 87% Isolated yield. ^{1}H NMR (500 MHz, $CDCl_3$): δ 7.25 (d, J = 7.6 Hz, 2H), 7.16 (d, J = 7.7 Hz, 2H), 5.22 (s, 1H), 4.69 (d, J = 11.7 Hz, 1H), 4.45 (d, J = 11.6 Hz, 1H), 3.91 (dq, J = 26.1 Hz, 7.2 Hz, 2H), 2.35 (s, 3H), 2.08-1.81 (m, 4H). ^{13}C NMR (125 MHz, $CDCl_3$): δ 137.3, 135.3, 129.1, 128.1, 103.0, 68.7, 67.1, 32.4, 23.6, 21.2. QEFMS calcd for $[C_{12}H_{16}O_2Na]^+$: 215.10425, found: 216.10402.

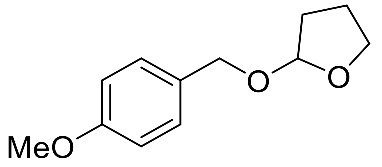

5g

Colorless liquid. 98% Isolated yield. ^{1}H NMR (500 MHz, $CDCl_3$): δ 7.27 (d, J = 8.2 Hz, 2H), 6.88 (d, J = 7.6 Hz, 2H), 5.20 (s, 1H) 4.65 (d, J = 11.4 Hz, 1H), 4.41 (d, J = 11.4 Hz, 1H), 3.92 (sept, J = 7.3 Hz, 2H), 3.8 (s, 3H), 2.07-1.80 (m, 4H). ^{13}C NMR (125 MHz, $CDCl_3$): δ 159.2, 130.5, 129.6, 113.8, 102.9, 68.5, 67.1, 55.4, 32.4, 23.6. QEFMS calcd for $[C_{12}H_{16}O_3Na]^+$: 231.09859, found: 231.09917.

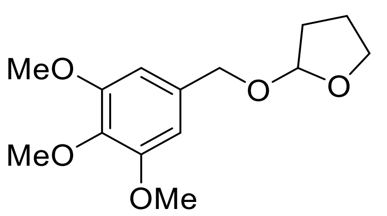

5h

Colorless liquid. 99% Isolated yield. ^{1}H NMR (500 MHz, $CDCl_3$): δ 6.5 (s, 2H), 5.22 (t, J = 3.2 Hz, 1H), 4.63 (d, J = 11.7 Hz, 1H), 4.40 (d, J = 11.7 Hz, 1H), 3.98-3.89 (m, 2H), 3.86 (s, 6H), 3.82 (s, 3H), 2.07-1.82 (m, 4H). ^{13}C NMR (125 MHz, $CDCl_3$): δ 153.4, 137.4, 134.0, 105.0, 103.2, 69.2, 67.2, 60.9, 56.2, 32.5, 23.6. QEFMS calcd for $[C_{14}H_{20}O_5Na]^+$: 291.12029, found: 291.11961.

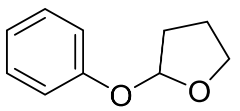

5i

Colorless liquid. 96% Isolated yield. ^{1}H NMR (500 MHz, $CDCl_3$): δ 7.28 (t, J = 7.6 Hz, 2H), 7.03 (d, J = 7.9 Hz, 2H), 6.98 (t, J = 7.4 Hz, 1H), 5.81 (d, J = 4.7 Hz, 1H), 4.07-4.03 (m, 1H), 3.97-3.93 (m, 1H), 2.21-2.10 (m, 3H), 1.99-1.90 (m, 1H). ^{13}C NMR (125 MHz, $CDCl_3$): δ 157.3, 129.5, 121.6, 116.6, 102.4, 68.2, 32.8, 23.6. QEFMS calcd for $[C_{10}H_{12}O_2Na]^+$: 187.07295, found: 187.07261.

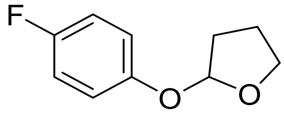

5j

Colorless liquid. 71% Isolated yield. ^{1}H NMR (500 MHz, $CDCl_3$): δ 6.99-6.94 (m, 4H), 4.76 (d, J = 4.8 Hz, 1H), 4.07-4.02 (m, 1H), 3.97-3.92 (m, 1H), 2.21-2.06 (m, 3H), 1.99-1.89 (m, 1H). ^{13}C NMR (125 MHz, $CDCl_3$): δ 158.0 (d, J_{CF} = 237.5 Hz), 153.4 (d, J_{CF} = 1.25 Hz), 118.0 (d, J_{CF} = 8.75 Hz), 115.84 (d, J_{CF} = 22.5 Hz), 103.1, 68.1, 32.8, 23.5. ^{19}F NMR (470 MHz, $CDCl_3$): δ -123.0 (sext, J_{CF} = 4.7 Hz). QEFMS calcd for $[C_{10}H_{11}FO_2Na]^+$: 205.06353, found: 205.06357.

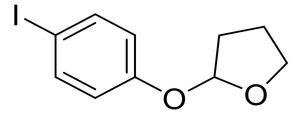

5k

Colorless liquid. 88% Isolated yield. ^{1}H NMR (500 MHz, $CDCl_3$): δ 7.54 (d, J = 8.8 Hz, 2H), 6.80 (d, J =8.8 Hz, 2H), 5.74 (d, J =4.6 Hz, 1H), 4.02 (q, J = 7.6 Hz, 1H), 3.94 (q, J = 7.9 Hz, 1H), 2.19-1.92 (m, 4H). ^{13}C NMR (125 MHz, $CDCl_3$): δ 157.2, 138.2, 119.0, 102.4, 84.0, 68.3, 32.8, 23.5. QEFMS calcd for $[C_{10}H_{11}IO_2Na]^+$: 312.96959, found: 312.96946.

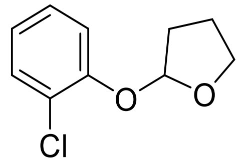

5l

Colorless liquid. 72% Isolated yield. ^{1}H NMR (500 MHz, $CDCl_3$): δ 7.52 (dd, J = 7.9 Hz, 1.5 Hz, 1H), 7.23-7.18 (m, 2H), 6.87 (td, J = 7.9 Hz, 1.7 Hz, 1H), 5.82 (d, J = 4.5 Hz, 1H), 4.10 (dt, J = 8.1 Hz, 5.6 Hz, 1H), 3.96 (q, J =6.7 Hz, 1H), 2.35-1.93 (m, 4H). ^{13}C NMR (125 MHz, $CDCl_3$): δ 153.8, 133.3, 128.5, 123.0, 117.4, 113.7, 103.5, 68.5, 32.9, 23.4. QEFMS calcd for $[C_{10}H_{11}ClO_2Na]^+$: 221.0339, found: 221.0336.

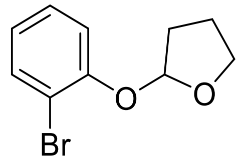

5m

Colorless liquid. 94% Isolated yield. ^{1}H NMR (500 MHz, $CDCl_3$): δ 7.35 (dd, J = 7.9 Hz, 6.5 Hz, 1H), 7.23 (m, 2H), 6.95-6.91 (m, 1H), 5.81 (d, J = 4.5 Hz, 1H), 4.10 (dd, J = 13.8 Hz, 5.8 Hz, 1H), 3.97 (dd, J =14.3 Hz, 7.7 Hz, 1H), 2.34-2.29 (m, 1H), 2.27-2.10 (m, 2H), 2.00-1.93(m, 1H). ^{13}C NMR (125 MHz, $CDCl_3$): δ 152.9, 130.3, 127.7, 124.2, 122.6, 117.6, 103.5, 68.5, 32.9, 23.4. QEFMS calcd for $[C_{10}H_{11}BrO_2Na]^+$: 264.98346, found: 264.98317.

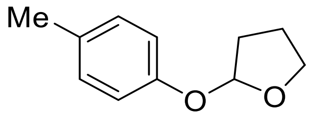

5n

Colorless liquid. 85% Isolated yield. ^{1}H NMR (500 MHz, $CDCl_3$): *δ* 7.22 (d, *J* = 9.0 Hz, 2H), 6.98 (d, *J* = 9.0 Hz, 2H), 5.37 (t, *J* = 3.2 Hz, 1H), 3.90-3.85 (m, 1H), 3.62-3.57 (m, 1H), 2.03-1.95 (m, 1H), 1.87-1.84 (m, 2H), 1.73-1.56 (m, 4H). ^{13}C NMR (125 MHz, $CDCl_3$): *δ* 155.8, 129.4, 126.6, 117.9, 96.7, 62.1, 30.4, 25.3, 18.8. QEFMS calcd for $[C_{11}H_{14}O_2Na]^+$: 201.08860, found: 201.08839.

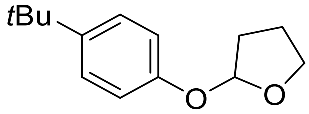

5o

Colorless liquid. 99% Isolated yield, 33% Isolated yield. ^{1}H NMR (500 MHz, $CDCl_3$): *δ* 7.29 (dt, *J* = 8.8 Hz, 2.1 Hz, 2H), 6.96 (dt, *J* = 8.8 Hz, 2.2 Hz, 2H), 5.80 (d, *J* = 4.8 Hz, 1H), 4.06-4.02 (m, 1H), 3.96-3.92 (m, 1H), 2.20-1.89 (m, 4H), 1.29 (s, 9H). ^{13}C NMR (125 MHz, $CDCl_3$): *δ* 154.9, 144.3, 126.3, 116.0, 102.4, 68.1, 34.2, 32.8, 31.6, 23.6. QEFMS calcd for $[C_{14}H_{20}O_2Na]^+$: 243.13555, found: 243.13538.

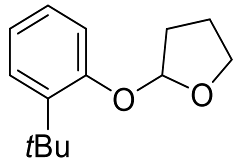

5p

Colorless liquid. 75% Isolated yield. ^{1}H NMR (400 MHz, $CDCl_3$): *δ* 7.30-7.26 (m, 1H), 7.23-7.15 (m, 2H), 6.94-6.90 (m, 1H), 5.87 (d, *J* = 4.6 Hz, 1H), 4.09-4.04 (m, 1H), 4.01-3.96 (m, 1H), 2.30 (m, 3H), 2.05-1.97 (m, 1H), 1.38 (s, 9H). ^{13}C NMR (100 MHz, $CDCl_3$): *δ* 156.0, 138.2, 127.1, 126.7, 121.0, 114.6, 101.8, 68.0, 34.9, 32.9, 30.0, 23.7. QEFMS calcd for $[C_{14}H_{20}O_2Na]^+$: 243.13555, found: 243.13533.

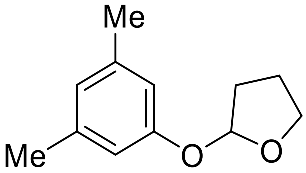

5q

Colorless liquid. 81% Isolated yield. ^{1}H NMR (500 MHz, $CDCl_3$): δ 6.69 (s, 2H), 6.65 (s, 1H), 5.81 (d, J = 4.8 Hz, 1H), 4.09-4.04 (m, 1H), 3.98-3.93 (m, 1H), 2.30 (s, 6H), 2.20-1.92 (m, 4H). ^{13}C NMR (125 MHz, $CDCl_3$): δ 157.2, 139.2, 123.4, 114.3, 102.2, 68.1, 32.8, 23.6, 21.5. QEFMS calcd for $[C_{12}H_{16}O_2Na]^+$: 215.10425, found: 215.10431.

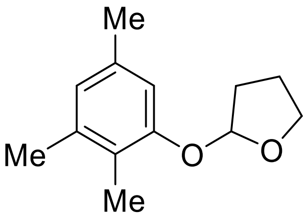

5r

Colorless liquid. 97% Isolated yield, 31% Isolated yield. ^{1}H NMR (500 MHz, $CDCl_3$): δ 6.85 (s, 1H), 6.67 (s, 1H), 5.79 (d, J = 4.7 Hz, 1H), 4.09 (q, J = 6.0 Hz, 1H), 3.97 (q, J = 6.2 Hz, 1H) 2.30 (s, 3H), 2.25-2.11 (m, 9H), 1.99-1.96 (m, 1H). ^{13}C NMR (125 MHz, $CDCl_3$): δ 155.1, 137.7, 135.6, 124.1, 123.0, 113.5, 102.8, 68.0, 32.9, 23.7, 21.4, 20.2, 11.7. QEFMS calcd for $[C_{13}H_{18}O_2Na]^+$: 229.11990, found: 229.11983.

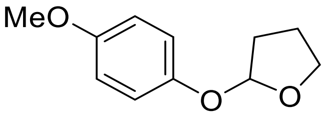

5s

Colorless liquid. 99% Isolated yield. ^{1}H NMR (500 MHz, $CDCl_3$): δ 6.97 (dd, J = 6.7 Hz, 2.3 Hz, 2H), 6.82 (dd, J = 6.7 Hz, 2.3 Hz, 2H), 5.70 (t, J = 4.8 Hz, 1H), 4.07-4.03 (m, 1H), 3.96-3.90 (m, 1H), 3.76 (s, 3H), 2.18-1.90 (m, 4H). ^{13}C NMR (125 MHz, $CDCl_3$): δ 154.7, 151.2, 118.0, 114.7, 103.3, 68.0, 55.8, 32.8, 23.6. QEFMS calcd for $[C_{11}H_{14}O_3Na]^+$: 217.08352, found: 217.08356.

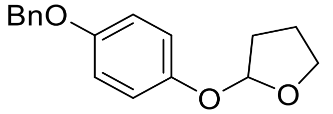

5t

Colorless liquid. 89% Isolated yield. ^{1}H NMR (500 MHz, $CDCl_3$): δ 7.45 (d, J = 7.1 Hz, 2H), 7.40 (t, J = 7.2 Hz, 2H), 7.34 (t, J = 7.2 Hz, 1H), 7.00 (dd, J = 6.7 Hz, 2.35 Hz, 2H), 6.92 (dd, J = 6.7 Hz, 2.35 Hz, 2H), 5.73 (d, J = 4.8 Hz, 1H), 5.03 (s, 2H), 4.10-4.05 (m, 1H), 3.98-3.94 (m, 1H), 2.20-1.91 (m, 4H). ^{13}C NMR (125 MHz, $CDCl_3$): δ 153.8, 151.5, 137.4, 128.6, 128.6, 127.9, 127.6, 118.0, 115.8, 70.6, 67.9, 32.7, 23.5. QEFMS calcd for $[C_{17}H_{18}O_3Na]^+$: 293.11482, found: 293.11440.

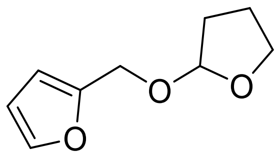

5u

Colorless liquid. 70% Isolated yield. ^{1}H NMR (500 MHz, $CDCl_3$): δ 7.40 (s, 1H), 6.32 (m, 2H), 5.21 (t, J = 3.0 Hz, 1H), 4.60 (d, J = 12.8 Hz, 1H), 4.46 (d, J = 12.8 Hz, 1H), 3.92 (m,2H), 2.05-1.97 (m, 1H), 1.95-1.91 (m, 2H), 1.87-1.79 (m, 1H) . ^{13}C NMR (125 MHz, $CDCl_3$): δ 151.9, 142.9, 110.4, 109.3, 102.8, 67.2, 60.7, 32.4, 25.5. QEFMS calcd for $[C_9H_{12}O_3Na]^+$: 191.06787, found: 191.06778.

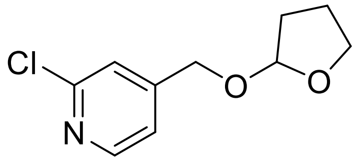

5v

Colorless liquid. 65% Isolated yield. ^{1}H NMR (400 MHz, $CDCl_3$): δ 8.33 (d, J = 1.9 Hz, 1H), 7.61 (dd, J = 8.2, 2.2 Hz, 1H), 7.28 (d, J = 8.1 Hz, 1H), 5.18 (t, J = 2.8 Hz, 1H), 4.67 (d, J = 12.2 Hz, 1H), 4.45 (d, J = 12.2 Hz, 1H), 3.89 (t, J = 6.3 Hz, 2H), 2.05-1.80 (m, 4H). ^{13}C NMR (100 MHz, $CDCl_3$): δ 150.6, 149.1, 138.4, 133.1, 124.1, 103.6, 67.4, 65.5, 32.5, 23.5. QEFMS calcd for $[C_{10}H_{12}NO_2Cl+H]^+$: 214.06293, found: 214.06257.

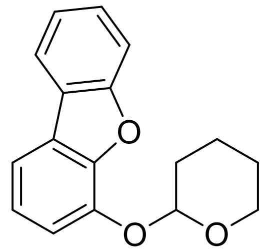

5w

Colorless liquid. 69% Isolated yield. ^{1}H NMR (500 MHz, $CDCl_3$): δ 7.93 (d, J = 7.7 Hz, 1H), 7.63-7.59 (m, 2H), 7.45 (t, J = 8.2 Hz, 1H), 7.34 (t, J = 7.2 Hz, 1H), 7.28-7.26 (m, 2H), 6.10 (d, J = 4.8 Hz, 1H), 4.17 (q, J = 8.0 Hz, 1H), 4.03 (q, J = 7.7 Hz, 1H), 2.49-2.40 (m, 1H), 2.35-2.10 (m, 2H), 2.08-1.96 (m, 1H). ^{13}C NMR (125 MHz, $CDCl_3$): δ 156.2, 146.2, 142.8, 127.2, 126.1, 124.6, 123.5, 122.8, 120.8, 114.6, 114.0, 112.0, 103.6, 68.5, 32.9, 23.5. QEFMS calcd for $[C_{16}H_{14}O_3Na]^+$: 277.08352, found: 277.08289.

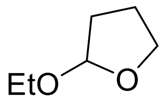

5x

Colorless liquid. 95% Isolated yield. ^{1}H NMR (500 MHz, $CDCl_3$): δ 5.07 (q, J = 3.3 Hz, 1H), 3.87-3.78 (m, 2H), 3.69-3.62 (m, 1H), 3.41-3.35 (m, 1H), 1.99-1.73 (m, 4H), 1.14 (t, J = 7.0 Hz, 3H). ^{13}C NMR (125 MHz, $CDCl_3$): δ 103.8, 67.0, 62.8, 32.5, 23.7, 15.4. QEFMS calcd for $[C_6H_{12}O_2Na]^+$: 139.07295, found: 139.07284.

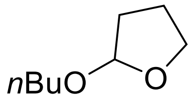

5y

Colorless liquid. 99% Isolated yield. ^{1}H NMR (500 MHz, $CDCl_3$): δ 5.1 (dd, J = 4.8 Hz, 1.6 Hz, 1H), 3.90-3.82 (m, 2H), 3.65 (dt, J = 9.6 Hz, 6.8 Hz, 1H), 3.36 (dt, J = 9.6 Hz, 6.7 Hz, 1H), 2.03-1.77 (m, 4H) , 1.56-1.51, (m, 2H), 1.39-1.31(m, 2H), 0.91 (t, J = 7.4 Hz 3H). ^{13}C NMR (125 MHz, $CDCl_3$): δ 103.9, 67.1, 66.9, 32.5, 32.0, 23.7, 19.5, 14.0. QEFMS calcd for $[C_8H_{16}O_2Na]^+$: 167.10425, found: 167.10419.

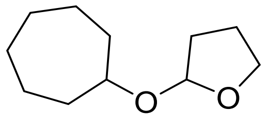

5z

Colorless liquid. 92% Isolated yield. ^{1}H NMR (500 MHz, $CDCl_3$): δ 5.20 (d, J = 4.8 1H), 3.89-3.79 (m, 2H), 3.69 (sept, J = 4.3, 1H), 2.02-1.93 (m, 1H), 1.92-1.74 (m, 5H), 1.64-1.46 (m, 8H), 1.40-1.33 (m, 2H). ^{13}C NMR (125 MHz, $CDCl_3$): δ 102.0, 76.9, 66.6, 35.9, 33.8, 32.7, 28.4, 28.2, 23.7, 23.2, 23.0. QEFMS calcd for $[C_{11}H_{20}O_2Na]^+$: 207.13555, found: 207.13549.

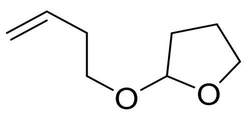

5aa

Colorless liquid. 94% Isolated yield. ^{1}H NMR (500 MHz, $CDCl_3$): δ 5.85-5.77 (m, 1H), 5.12-5.07 (m, 2H), 5.03-5.01 (m, 1H), 3.87 (dq, J = 18.5 Hz, 7.9 Hz, 2H), 3.87 (dt, J = 13.9 Hz, 6.9 Hz, 1H), 3.43 (dt, J = 9.6 Hz, 6.9 Hz, 1H), 2.32 (q, J = 6.8 Hz, 2H), 2.02-1.78 (m, 4H). ^{13}C NMR (125 MHz, $CDCl_3$): δ 135.5, 116.3, 103.9, 67.0, 66.6, 34.3, 32.5, 23.6. QEFMS calcd for $[C_8H_{14}O_2Na]^+$: 165.08860, found: 165.08851.

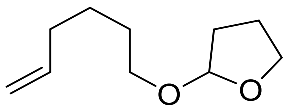

5ab

Colorless liquid. 99% Isolated yield. ^{1}H NMR (500 MHz, $CDCl_3$): δ 5.84-5.75 (m, 1H), 5.09 (dd, J = 1.5 Hz, 4.7 Hz, 1H), 5.01-4.97 (m, 1H), 4.94-4.91 (m, 1H), 3.90-3.82 (m, 2H), 3.64 (dt, J = 6.8 Hz, 9.6 Hz, 1H), 3.36 (dt, J = 6.8 Hz, 9.6 Hz, 1H), 2.06 (q, J = 7.3 Hz, 2H), 2.01-1.81 (m, 4H), 1.57 (quin, J = 6.6 Hz, 2H), 1.42 (quin, J = 7.3 Hz, 2H). ^{13}C NMR (125 MHz, $CDCl_3$): δ 138.9, 114.6, 103.9, 67.1, 66.9, 33.6, 32.4, 29.3, 25.6, 23.6. QEFMS calcd for $[C_{10}H_{18}O_2Na]^+$: 193.11990, found: 193.11995.

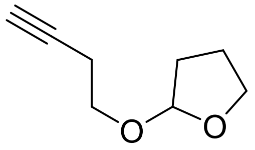

5ac

Colorless liquid. 97% Isolated yield. ^{1}H NMR (500 MHz, $CDCl_3$): δ 5.14 (t, J = 2.8 Hz, 1H), 3.92-3.83 (m, 2H), 3.77-3.72 (m, 1H), 3.56-3.51 (m, 1H), 2.44 (sext, J = 2.5 Hz, 2H), 2.03-1.95 (m, 2H), 1.93-1.89 (m, 2H), 1.85-1.77 (m, 1H). ^{13}C NMR (125 MHz, $CDCl_3$): δ 104.0, 81.6, 69.2, 67.1, 65.3, 32.4, 23.5, 20.1. QEFMS calcd for $[C_8H_{12}O_2Na]^+$: 163.07295, found: 163.07270.

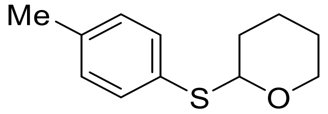

6a

Colorless liquid. 88% Isolated yield. ^{1}H NMR (400 MHz, $CDCl_3$): δ 7.39 (d, J = 8.0 Hz, 2H), 7.11 (d, J = 7.9 Hz, 2H), 5.14 (dd, J = 6.1, 2.1 Hz, 1H), 4.20-4.15(m, 1H), 3.59-3.54 (m, 1H), 2.32 (s, 3H), 2.05-1.98 (m, 1H), 1.90-1.77 (m, 2H), 1.68-1.58 (m, 3H). ^{13}C NMR (100 MHz, $CDCl_3$): δ 137.1, 131.8, 131.5, 129.7, 85.8, 64.7, 31.7, 25.7, 21.8, 21.2. QEFMS calcd for $[C_{12}H_{16}OSNa]^+$: 231.08141, found: 231.08107.

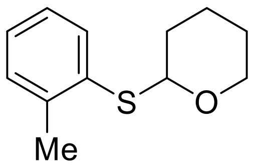

6b

Colorless liquid. 89% Isolated yield. ^{1}H NMR (400 MHz, $CDCl_3$): δ 7.56-7.54 (m, 1H), 7.18-7.10 (m, 3H), 5.21 (dd, J = 5.6 Hz, 1.8 Hz, 1H), 4.20-4.14 (m, 1H), 3.63-3.58 (m, 1H), 2.40 (s, 3H), 2.09-2.02 (m, 1H), 1.95-1.84 (m, 2H), 1.71-1.59 (m, 3H). ^{13}C NMR (100 MHz, $CDCl_3$): δ 138.3, 135.1, 130.4, 130.1, 126.6, 126.5, 84.6, 64.7, 31.9, 25.7, 21.8, 20.9. QEFMS calcd for $[C_{12}H_{16}OSNa]^+$: 231.08141, found: 231.08113.

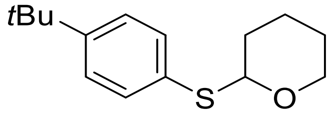

6c

Colorless liquid. 96% Isolated yield. ^{1}H NMR (400 MHz, $CDCl_3$): δ 7.42 (d, J = 8.5 Hz, 2H), 7.31 (d, J = 8.5 Hz, 2H), 5.17 (d, J = 9.6 Hz, 1H), 4.21-4.16 (m, 1H), 3.61-

3.55 (m, 1H), 2.06-1.99 (m, 1H), 1.91-1.79 (m, 2H), 1.68-1.58 (m, 3H), 1.30 (s, 9H). ^{13}C NMR (100 MHz, $CDCl_3$): δ 150.1, 131.8, 131.2, 126.0, 85.7, 64.6, 34.6, 31.7, 31.4, 25.7, 21.8. QEFMS calcd for $[C_{15}H_{22}OSNa]^+$: 273.12836, found: 273.12806.

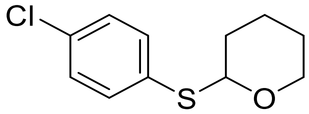

6d

Colorless liquid. 79% Isolated yield. ^{1}H NMR (500 MHz, $CDCl_3$): δ 7.40 (d, J = 8.5 Hz, 2H), 7.25 (d, J = 8.6 Hz, 2H), 5.17 (t, J = 5.8 Hz, 1H), 4.17-4.13 (m, 1H), 3.60-3.56 (m, 1H), 2.05-1.99 (m, 1H), 1.88-1.78 (m, 2H), 1.68-1.60 (m, 3H). ^{13}C NMR (125 MHz, $CDCl_3$): δ 134.0, 132.9, 132.3, 129.0, 85.4, 64.6, 31.6, 25.6, 21.7. QEFMS calcd for $[C_{11}H_{13}ClOSNa]^+$: 251.02678, found: 251.02649.

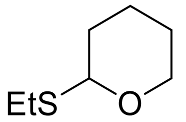

6e

Colorless liquid. 99% Isolated yield. ^{1}H NMR (500 MHz, $CDCl_3$): δ 4.87 (dd, J = 6.7 Hz, 3.1 Hz, 1H), 4.11-4.07 (m, 1H), 3.53-3.48 (m, 1H), 2.72-2.54 (m, 2H), 1.95-1.90 (m, 1H), 1.85-1.80 (m, 1H), 1.71-1.65 (m, 1H), 1.61-1.53 (m, 3H), 1.28 (t, J = 7.4 Hz, 3H). ^{13}C NMR (125 MHz, $CDCl_3$): δ 82.1, 64.9, 31.6, 25.8, 24.5, 22.0, 15.2. QEFMS calcd for $[C_7H_{14}SONa]^+$: 169.06576, found: 169.06565.

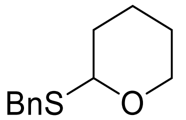

6f

Colorless liquid. 97% Isolated yield. ^{1}H NMR (400 MHz, $CDCl_3$): δ 7.26-7.21 (m, 4H), 7.18-7.14 (m, 1H), 4.63 (dd, J = 6.4 Hz, 2.7 Hz, 1H), 4.07-4.02 (m, 1H), 3.78 (d, J = 13.2 Hz, 1H), 3.64 (d, J = 13.2 Hz, 1H), 3.46-3.40 (m, 1H), 1.82-1.68 (m, 2H), 1.60-1.41 (m, 4H). ^{13}C NMR (100 MHz, $CDCl_3$): δ 138.7, 129.1, 128.6, 126.9, 80.6, 64.4, 34.0, 30.9, 25.8, 21.7. QEFMS calcd for $[C_{12}H_{16}OSNa]^+$: 231.08141, found: 231.08119.

3. Catalyst stability test

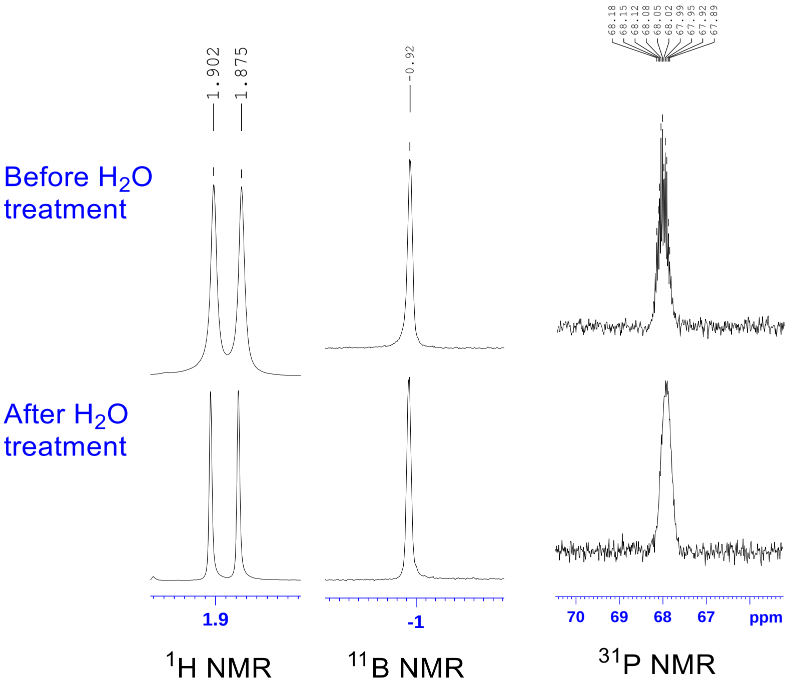

Figure S1. NMR experiments of **TOB-1** before and after treatment with water

*Note: **TOB-1** catalyst (0.1 mmol) was dissolved in H_2O (0.1 mL) and the solution was stirred at 23 °C for 12 h. Water was then removed under reduced pressure and the residue was dissolved in $CDCl_3$ for ^1H, ^{11}B, and ^{31}P NMR experiments.*

4. Solvent screening and temperature effect on the chemoselectivity

Table S1. Solvent screening[a]

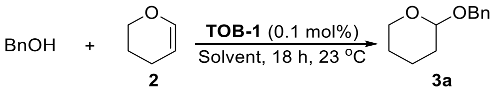

Entry	Solvent	Yield [%]
1	$CHCl_3$ (no catalyst)	0
2	$CHCl_3$	93
3	CH_2Cl_2	40
4	$(CH_2Cl)_2$	50
5	THF	0
6	Acetone	14
7	MeCN	49
8	*n*-Hexane	17
9	PhMe	30

[a]Reaction were carried out with benzyl alcohols (0.20 mmol), 3,4-dihydro-2H-pyran (**2**) (0.24 mmol) and **TOB-1** (0.1 mol%) in solvent (0.4 mL) at 23 °C for 18 h. The yields were measured in NMR with dibromomethane as the internal standard.

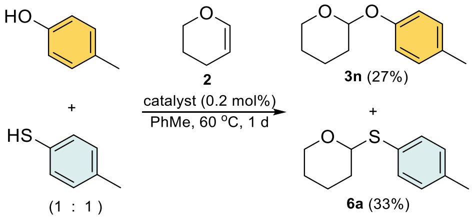

Scheme S1. Study on the temperature effect on the chemoselective acetalization

5. NMR titration experiment with TOB-1 and benzyl alcohol

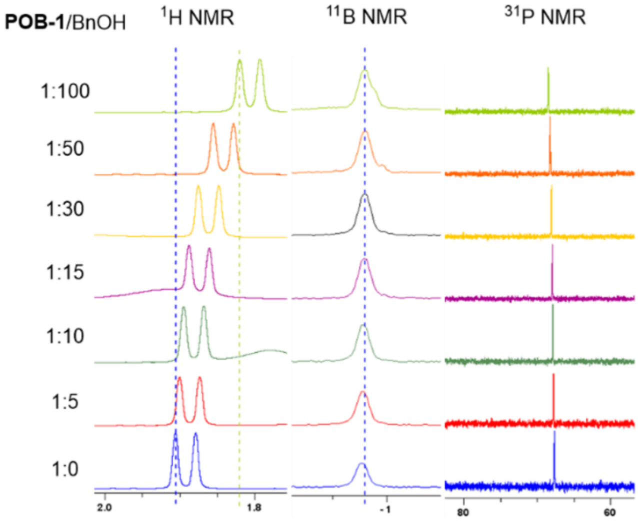

Figure S2. NMR titration experiment with **TOB-1** and benzyl alcohol

*Note: **TOB-1** catalyst (8.0 mg, 0.05 mmol, 1 equiv) was dissolved in $CDCl_3$ (0.4 mL) and the ^{1}H, ^{11}B and ^{31}P NMR spectra were obtained. Different amount of benzyl alcohol was then added and the ^{1}H, ^{11}B and ^{31}P NMR were obtained after each addition.*

6. Comparison of the chemical shift of alcohols and thiols

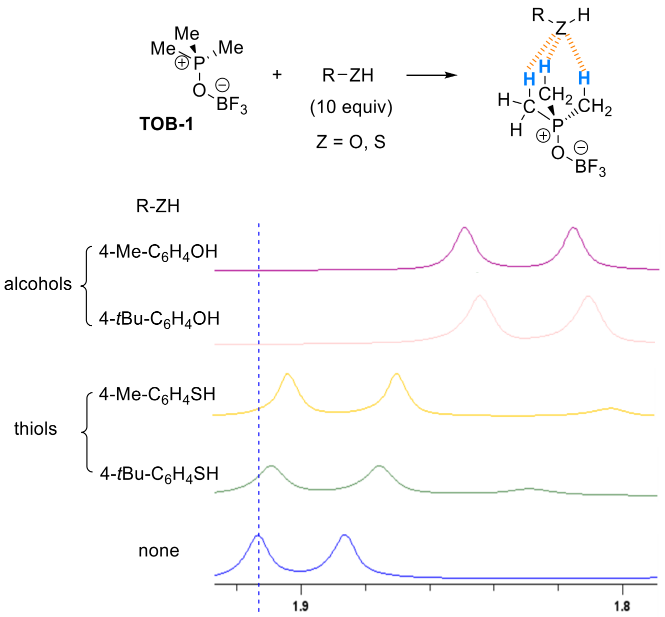

Figure S3. Comparison of the chemical shift of alcohols and thiols in the presence of **TOB-1**

*Note: **TOB-1** catalyst (8.0 mg, 0.05 mmol, 1 equiv) was dissolved in $CDCl_3$ (0.4 mL) and alcohol or thiol (0.5 mmol, 10 equiv) was added. ^1H NMR experiments were then carried out on these samples.*

7. X-ray crystal data of TOB-2

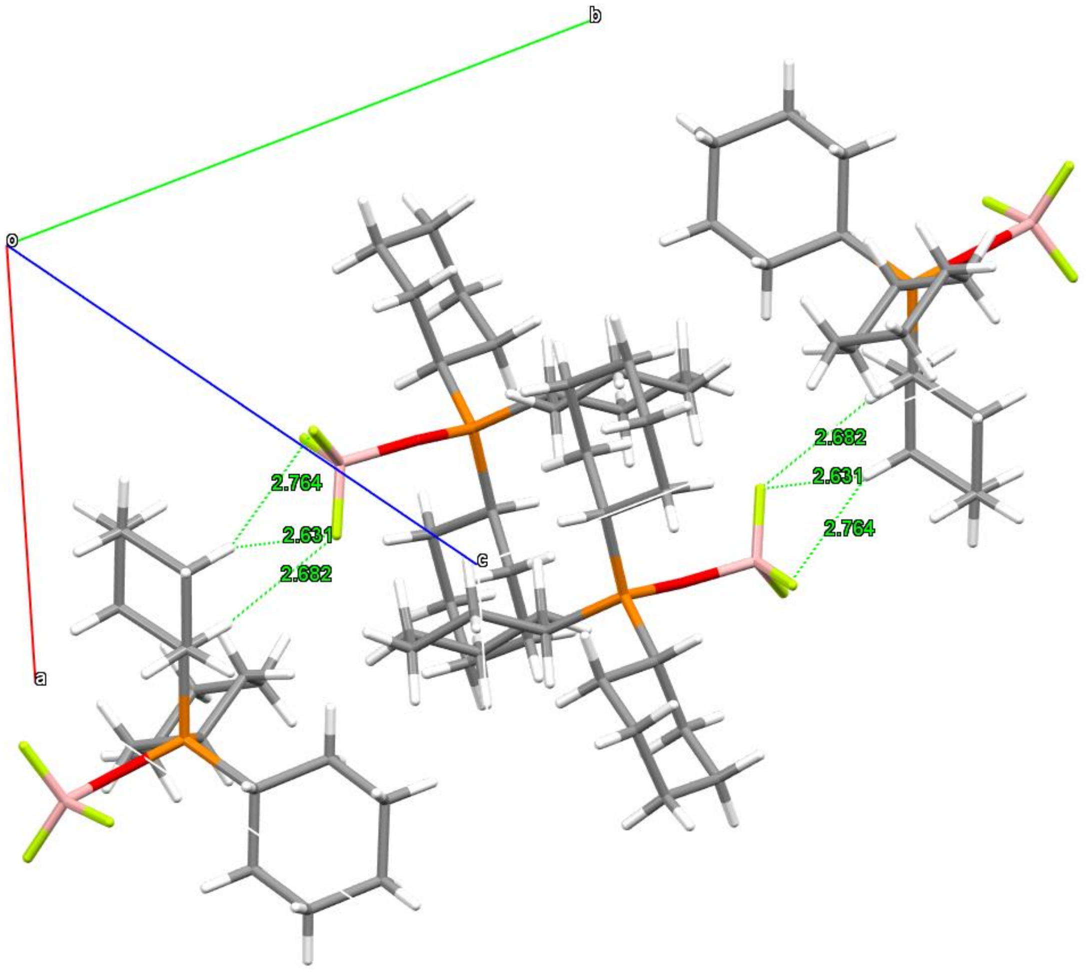

Figure S4. X-ray crystal packing of **TOB-2** (CCDC 2209696)

Crystal data and structure refinement for **TOB-2**.

Identification code	vinn1173pcy3obf3	
Empirical formula	C18 H33 B F3 O P	
Formula weight	364.22	
Temperature	296(2) K	
Wavelength	0.71073 Å	
Crystal system	Monoclinic	
Space group	$P2_1/c$	
Unit cell dimensions	a = 7.9919(7) Å	α= 90°.
	b = 13.6548(12) Å	β= 94.856(3)°.
	c = 18.5379(18) Å	γ = 90°.
Volume	2015.7(3) $Å^3$	
Z	4	
Density (calculated)	1.200 Mg/m^3	
Absorption coefficient	0.165 mm^{-1}	
F(000)	784	
Crystal size	0.400 x 0.300 x 0.200 mm^3	
Theta range for data collection	2.662 to 25.248°.	
Index ranges	-9<=h<=8, -16<=k<=16, -22<=l<=22	
Reflections collected	20181	
Independent reflections	3631 [R(int) = 0.0290]	
Completeness to theta = 25.242°	99.3 %	
Absorption correction	multi-scan	
Max. and min. transmission	0.7456 and 0.6570	
Refinement method	Full-matrix least-squares on F^2	
Data / restraints / parameters	3631 / 0 / 245	
Goodness-of-fit on F^2	1.056	
Final R indices [I>2sigma(I)]	R1 = 0.0469, wR2 = 0.1258	
R indices (all data)	R1 = 0.0610, wR2 = 0.1410	
Extinction coefficient	n/a	
Largest diff. peak and hole	0.374 and -0.189 e.$Å^{-3}$	

Atomic coordinates (x 10^4) and equivalent isotropic displacement parameters ($Å^2$x 10^3) for **TOB-2**. U(eq) is defined as one third of the trace of the orthogonalized U^{ij} tensor.

	x	y	z	U(eq)
B(1)	6916(4)	3907(3)	3335(2)	92(1)
O(1)	5534(2)	4635(1)	3382(1)	64(1)
P(1)	3688(1)	4789(1)	3121(1)	41(1)
C(1)	3164(3)	6029(2)	3379(1)	53(1)
C(2)	4279(4)	6443(2)	3993(2)	83(1)
C(3)	3945(4)	7523(2)	4102(2)	90(1)
C(4)	2090(5)	7714(2)	4178(2)	101(1)
C(5)	983(4)	7291(2)	3588(2)	77(1)
C(6)	1318(3)	6209(2)	3472(1)	59(1)
C(7)	2363(2)	3908(1)	3522(1)	45(1)
C(8)	2498(3)	2874(2)	3215(1)	59(1)
C(9)	1327(3)	2175(2)	3571(2)	74(1)
C(10)	1648(4)	2176(2)	4389(2)	86(1)
C(11)	1546(3)	3193(2)	4695(1)	77(1)
C(12)	2726(3)	3893(2)	4348(1)	63(1)
C(13)	3405(3)	4703(1)	2146(1)	46(1)
C(14)	4599(3)	5383(2)	1778(1)	73(1)
C(15)	4412(4)	5244(2)	961(2)	85(1)
C(16)	2623(5)	5378(2)	656(1)	86(1)
C(17)	1449(4)	4715(2)	1018(1)	80(1)
C(18)	1598(3)	4857(2)	1834(1)	57(1)
F(1)	6354(5)	3245(4)	2780(2)	105(2)
F(2)	8272(6)	4347(5)	3165(6)	154(4)
F(3)	7084(11)	3387(6)	3951(3)	146(4)
F(1')	6682(18)	3102(8)	3440(30)	298(18)
F(2')	7850(20)	4271(15)	2848(12)	205(9)
F(3')	8027(18)	4318(18)	3929(9)	202(8)

Bond lengths [Å] and angles [°] for **TOB-2**.

B(1)-F(1')	1.134(10)
B(1)-F(2)	1.302(6)
B(1)-F(2')	1.317(16)
B(1)-F(3)	1.341(6)
B(1)-F(1)	1.414(6)
B(1)-F(3')	1.466(13)
B(1)-O(1)	1.494(4)
O(1)-P(1)	1.5275(14)
P(1)-C(7)	1.8045(19)
P(1)-C(13)	1.805(2)
P(1)-C(1)	1.819(2)
C(1)-C(2)	1.496(3)
C(1)-C(6)	1.520(3)
C(2)-C(3)	1.515(4)
C(3)-C(4)	1.524(5)
C(4)-C(5)	1.466(4)
C(5)-C(6)	1.520(3)
C(7)-C(8)	1.529(3)
C(7)-C(12)	1.534(3)
C(8)-C(9)	1.526(3)
C(9)-C(10)	1.516(4)
C(10)-C(11)	1.506(4)
C(11)-C(12)	1.522(3)
C(13)-C(18)	1.524(3)
C(13)-C(14)	1.533(3)
C(14)-C(15)	1.520(4)
C(15)-C(16)	1.503(4)
C(16)-C(17)	1.502(4)
C(17)-C(18)	1.521(3)
F(1')-B(1)-F(2')	126.4(18)
F(2)-B(1)-F(3)	115.2(5)
F(2)-B(1)-F(1)	109.8(6)
F(3)-B(1)-F(1)	106.5(5)
F(1')-B(1)-F(3')	110.0(16)
F(2')-B(1)-F(3')	91.6(10)
F(1')-B(1)-O(1)	120.1(7)

F(2)-B(1)-O(1)	110.0(4)
F(2')-B(1)-O(1)	104.6(8)
F(3)-B(1)-O(1)	108.9(4)
F(1)-B(1)-O(1)	106.1(3)
F(3')-B(1)-O(1)	96.0(6)
B(1)-O(1)-P(1)	140.5(2)
O(1)-P(1)-C(7)	111.21(9)
O(1)-P(1)-C(13)	110.18(10)
C(7)-P(1)-C(13)	109.96(9)
O(1)-P(1)-C(1)	106.40(9)
C(7)-P(1)-C(1)	110.83(10)
C(13)-P(1)-C(1)	108.17(10)
C(2)-C(1)-C(6)	112.0(2)
C(2)-C(1)-P(1)	114.62(17)
C(6)-C(1)-P(1)	115.34(15)
C(1)-C(2)-C(3)	111.6(2)
C(2)-C(3)-C(4)	111.3(3)
C(5)-C(4)-C(3)	113.1(3)
C(4)-C(5)-C(6)	112.8(2)
C(5)-C(6)-C(1)	110.9(2)
C(8)-C(7)-C(12)	110.24(17)
C(8)-C(7)-P(1)	113.54(14)
C(12)-C(7)-P(1)	110.66(14)
C(9)-C(8)-C(7)	110.6(2)
C(10)-C(9)-C(8)	112.0(2)
C(11)-C(10)-C(9)	111.5(2)
C(10)-C(11)-C(12)	111.4(2)
C(11)-C(12)-C(7)	111.00(19)
C(18)-C(13)-C(14)	110.66(19)
C(18)-C(13)-P(1)	113.92(14)
C(14)-C(13)-P(1)	112.13(16)
C(15)-C(14)-C(13)	110.8(2)
C(16)-C(15)-C(14)	111.7(2)
C(17)-C(16)-C(15)	111.6(2)
C(16)-C(17)-C(18)	111.6(2)
C(17)-C(18)-C(13)	110.6(2)

Symmetry transformations used to generate equivalent atoms:

Anisotropic displacement parameters ($Å^2$x 10^3) for **TOB-2**. The anisotropic displacement factor exponent takes the form: $-2\pi^2[h^2 a^{*2}U^{11} + ... + 2 h k a^* b^* U^{12}]$

	U^{11}	U^{22}	U^{33}	U^{23}	U^{13}	U^{12}
B(1)	43(2)	115(3)	117(3)	22(3)	8(2)	24(2)
O(1)	33(1)	78(1)	81(1)	3(1)	-3(1)	5(1)
P(1)	31(1)	45(1)	46(1)	1(1)	2(1)	0(1)
C(1)	50(1)	48(1)	61(1)	-6(1)	5(1)	-5(1)
C(2)	76(2)	75(2)	92(2)	-24(2)	-21(2)	-8(1)
C(3)	116(3)	64(2)	86(2)	-25(2)	-11(2)	-20(2)
C(4)	136(3)	72(2)	93(2)	-32(2)	5(2)	12(2)
C(5)	85(2)	62(2)	86(2)	-12(1)	18(2)	16(1)
C(6)	56(1)	60(1)	63(1)	-13(1)	7(1)	9(1)
C(7)	36(1)	49(1)	48(1)	8(1)	1(1)	1(1)
C(8)	58(1)	49(1)	70(1)	4(1)	3(1)	-3(1)
C(9)	65(2)	56(1)	102(2)	17(1)	2(1)	-12(1)
C(10)	69(2)	85(2)	102(2)	46(2)	2(2)	-9(1)
C(11)	68(2)	100(2)	62(2)	31(1)	8(1)	-6(1)
C(12)	64(1)	76(2)	49(1)	14(1)	1(1)	-6(1)
C(13)	50(1)	45(1)	46(1)	1(1)	10(1)	2(1)
C(14)	70(2)	86(2)	67(2)	14(1)	22(1)	-15(1)
C(15)	115(3)	80(2)	66(2)	15(1)	43(2)	-1(2)
C(16)	132(3)	77(2)	48(1)	13(1)	10(2)	12(2)
C(17)	99(2)	88(2)	50(1)	2(1)	-9(1)	-6(2)
C(18)	58(1)	65(1)	47(1)	2(1)	-1(1)	-2(1)
F(1)	89(2)	108(3)	119(3)	-24(2)	16(2)	41(2)
F(2)	31(2)	138(4)	295(11)	28(5)	26(3)	-8(2)
F(3)	147(5)	176(8)	111(3)	55(3)	-11(3)	76(5)
F(1')	116(10)	57(5)	730(50)	71(18)	90(20)	8(5)
F(2')	121(11)	309(19)	200(13)	-16(11)	104(11)	67(12)
F(3')	115(8)	270(20)	203(12)	-2(12)	-84(8)	58(11)

Hydrogen coordinates (x 10^4) and isotropic displacement parameters ($Å^2$x 10 ^{3}) for **TOB-2**.

	x	y	z	U(eq)
H(1A)	3396	6431	2961	63
H(2A)	5443	6352	3897	99
H(2B)	4095	6090	4434	99
H(3A)	4295	7889	3692	108
H(3B)	4602	7750	4533	108
H(4A)	1796	7444	4634	121
H(4B)	1904	8416	4192	121
H(5A)	1136	7643	3144	92
H(5B)	-175	7375	3695	92
H(6A)	644	5982	3045	71
H(6B)	992	5839	3884	71
H(7A)	1198	4123	3417	54
H(8A)	2206	2886	2697	71
H(8B)	3646	2644	3300	71
H(9A)	172	2365	3439	89
H(9B)	1482	1517	3391	89
H(10A)	827	1760	4595	103
H(10B)	2753	1906	4524	103
H(11A)	1836	3173	5213	92
H(11B)	403	3432	4612	92
H(12A)	3879	3691	4471	75
H(12B)	2591	4547	4538	75
H(13A)	3708	4033	2021	56
H(14A)	4355	6058	1893	88
H(14B)	5748	5245	1960	88
H(15A)	5120	5713	738	102
H(15B)	4787	4592	845	102
H(16A)	2291	6054	721	103
H(16B)	2540	5242	141	103
H(17A)	1702	4039	908	96
H(17B)	304	4848	827	96
H(18A)	870	4395	2053	68
H(18B)	1238	5514	1948	68

8. Kinetic Study of the Acetalization Reaction

Four NMR tubes charged with $CDCl_3$ (0.4 mL) and internal standard mesitylene (27.8 µL, 0.2 mmol, 1 equiv). Different amounts of benzyl alcohol, 3,4-dihydropyran **2** and **TOB-1** were added to the four NMR tubes according to the following table.

Entry	Benzyl alcohol	3,4-Dihydropyran	TOB-1
**I**	20.7 µL(1 equiv.)	18.2 µL (1 equiv.)	1 mol %
**II**	10.4 µL (0.5 equiv.)	18.2 µL (1 equiv.)	1 mol %
**III**	20.7 µL (1 equiv.)	9.1 µL (0.5 equiv.)	1 mol %
**IV**	20.7 µL (1 equiv.)	18.2 µL (1 equiv.)	0.5 mol %

The reactions were monitored by ^{1}H NMR experiments in 1.5 hour. The NMR yield of the product were recorded and the curves of the amount of acetal product against time were plotted. A polynomial trendline of the graph was obtained and the initial rates were determined (Figure S5-S7). The rate orders of different reactants are summarized in Table S2.

Table S2. Rate order of different Reactants

**Reactants**	**Rate Order**
Benzyl alcohol	1.048
3,4-Dihydropyran **2**	1.034
**TOB-1**	1.040

(i) Kinetic study of benzyl alcohol

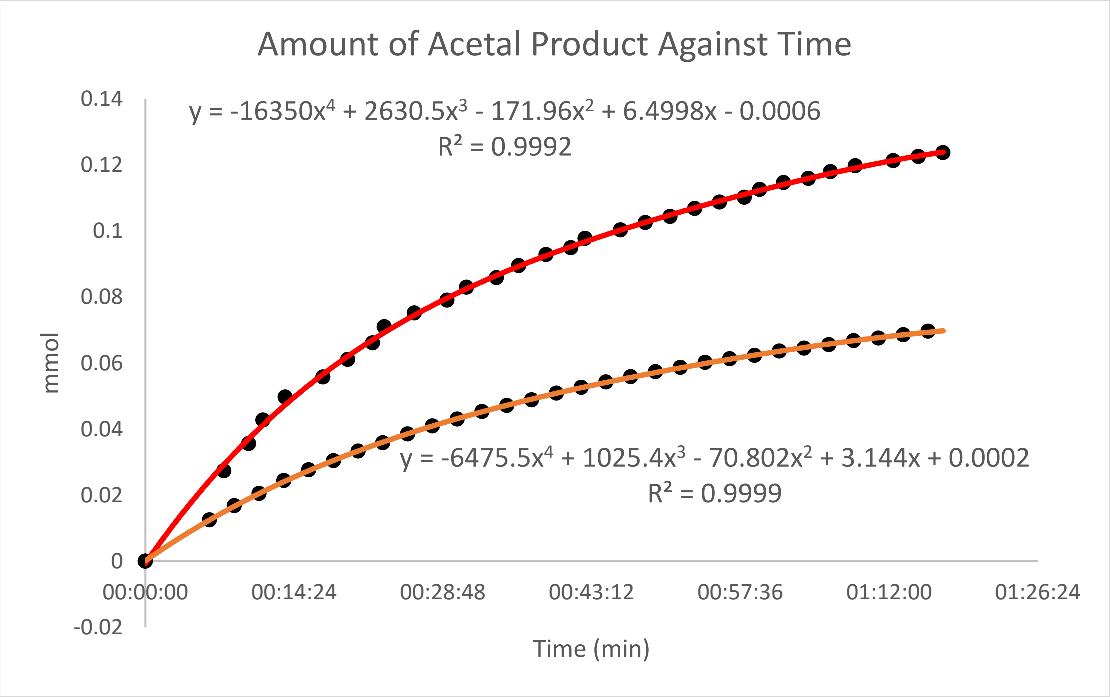

Figure S5. Kinetic study of benzyl alcohol

Rate I $=$ k[BnOH]^x[3,4-Dihydropyran]^y[**TOB-1**]^z

$= \frac{dy}{dt}$ (at t=0)

$=$ 6.4998

Rate II $=$ k$\frac{1}{2^x}$[BnOH]^x[3,4-Dihydropyran]^y[**TOB-1**]^z

$= \frac{dy}{dt}$ (at t=0)

$=$ 3.144

$\frac{Rate\ I}{Rate\ II} = 2^x$

x $=$ 1.048

Rate Order of benzyl alcohol = 1.048

(ii) Kinetic study of 3,4-dihydropyran **2**

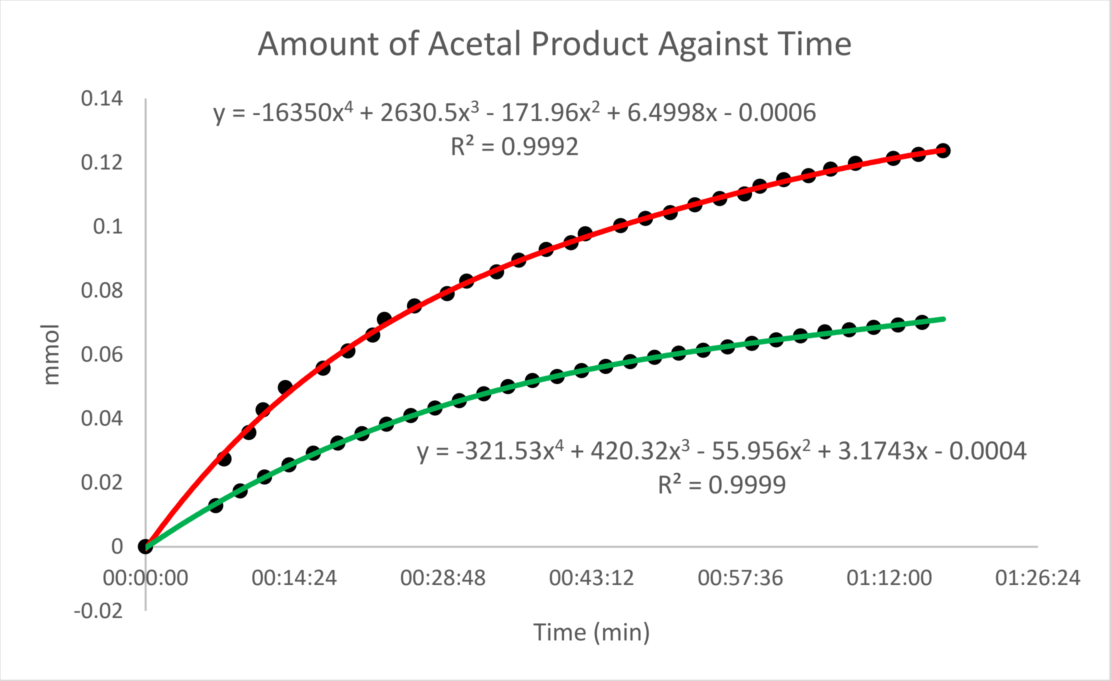

Figure S6. Kinetic study of 3,4-dihydropyran **2**

Rate I = $k[\text{BnOH}]^x[\text{3,4-Dihydropyran}]^y[\textbf{TOB-1}]^z$

= $\frac{dy}{dt}$ (at t=0)

= 6.4998

Rate III = $k[\text{BnOH}]^x \frac{1}{2^y}[\text{3,4-Dihydropyran}]^y[\textbf{TOB-1}.]^z$

= $\frac{dy}{dt}$ (at t=0)

= 3.1743

$\frac{Rate\ I}{Rate\ III}$ = 2^y

y = 1.034

Kinetic Order of 3,4-dihydropyran **2** = 1.034

(iii) Kinetic study of catalyst **TOB-1**

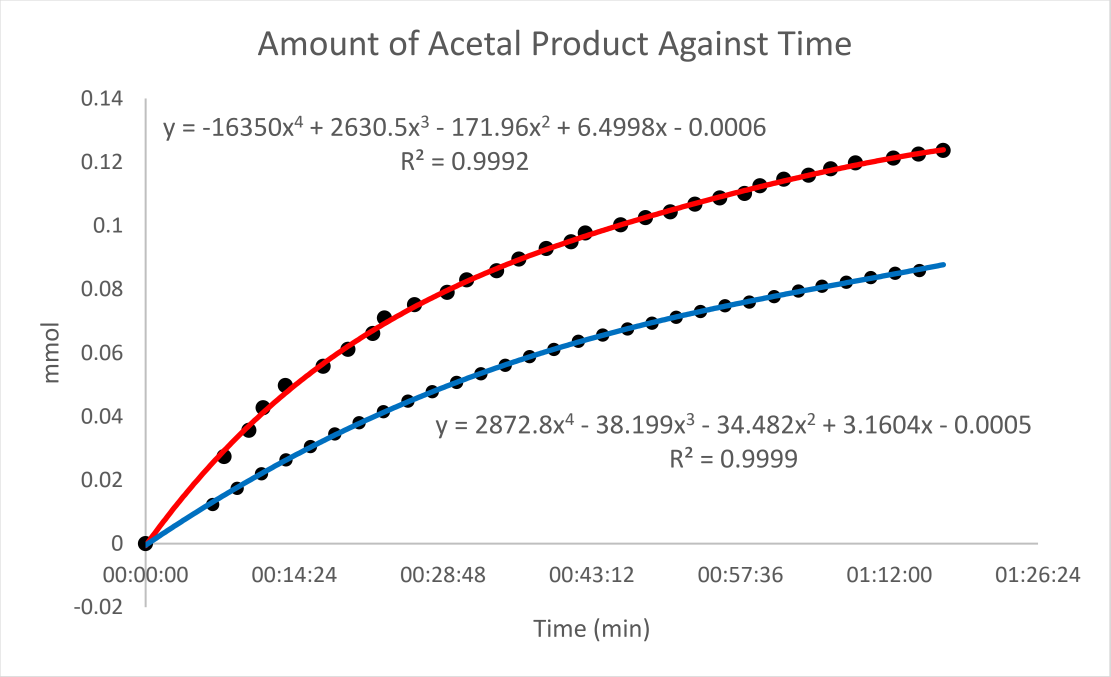

Figure S7. Kinetic study of catalyst **TOB-1**

Rate I = k[BnOH]^x[3,4-Dihydropyran]^y[**TOB-1**]^z

= $\frac{dy}{dt}$ (at t=0)

= 6.4998

Rate IV = k[BnOH]^x[3,4-Dihydropyran]^y $\frac{1}{2^z}$[**TOB-1**]^z

= $\frac{dy}{dt}$ (at t=0)

= 3.1604

$$\frac{Rate\ I}{Rate\ IV} = 2^z$$

z = 1.040

Kinetic Order of **TOB-1** = 1.040

9. Computational studies

All Density Functional Theory (DFT) calculations were carried out using M06-2X hybrid functional[2] with Grimme D3 dispersion correction.[3] These systems were studied in SMD solvents (chloroform and toluene)[4] with Gaussian 16 (ver. C.02).[5] Their geometries were optimized with 6-311G(d) basis set, while the single-point energies, electrostatic potential calculations, and NBO analyses were performed with aug-cc-pVTZ basis set. For binding energy calculations, basis set superposition errors (BSSE) were handled by a counterpoise procedure.[6] Based on the electrostatic potential (ESP) map of **TOB-1**, positive charges are shown to be localized at the methyl groups and phosphonium centre, suggesting that these positions are potential active sites of the catalyst. To investigate the interactions of the trilateral complexes formed between **2**, **TOB-1**, and the alcohol/thiol, configurational searches were performed by initially placing **2** and the alcohol/thiol near the potential active sites at methyl groups and phosphonium centre of **TOB-1**. Several optimized configurations were obtained, and the one with strongest binding between **2** and the complex of the other two species was selected for further study. Additional optimizations of the complexes were done with different implicit SMD solvents to study the solvent effect on the O/S chemoselectivity in the acetalization. The E(2) stabilization energies were computed with second order perturbation theory analysis in NBO basis.[7] Atoms In Molecules (AIM)[8] analysis and electrostatic potential (ESP) map generation was done by Multiwfn 3.8.[9]

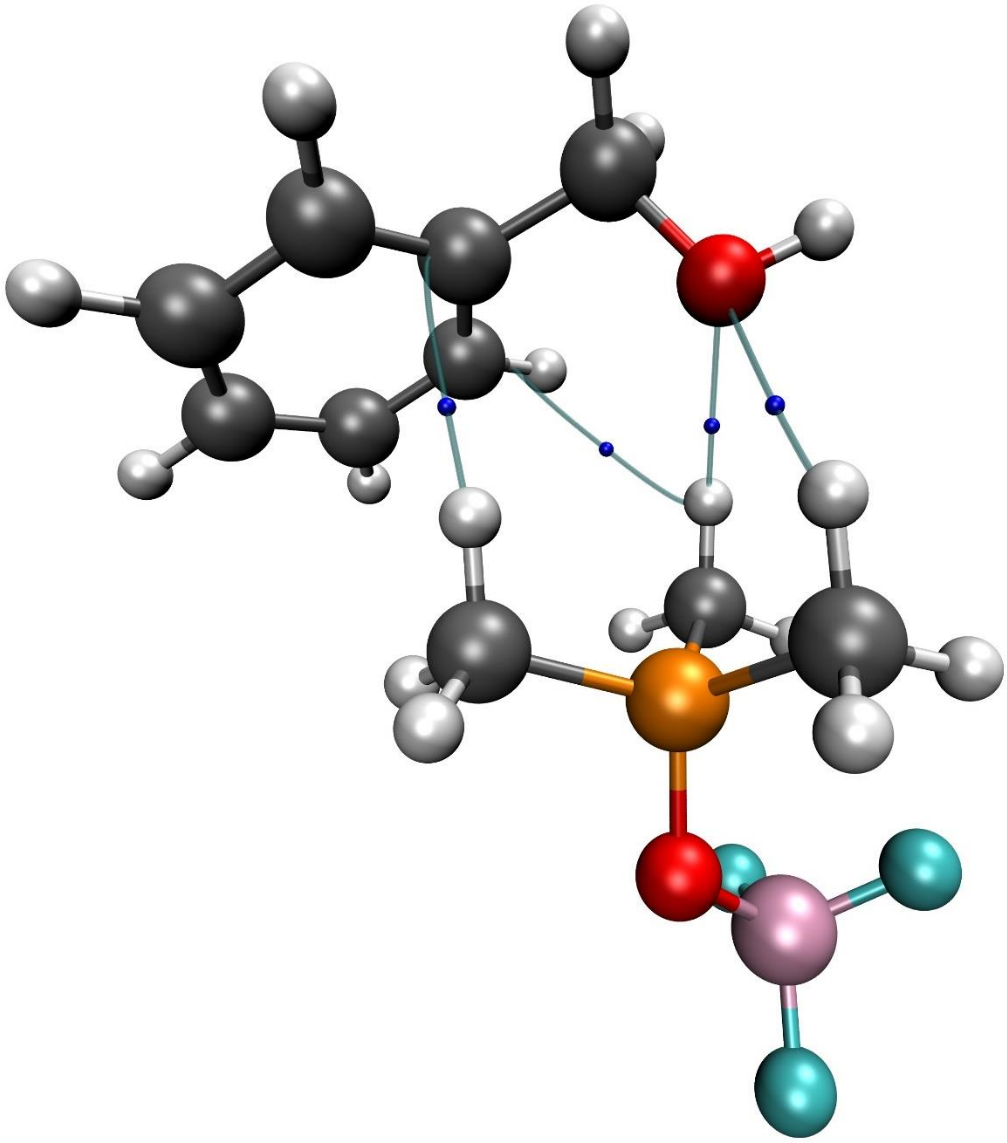

Figure S8. AIM bond paths in complex **A**

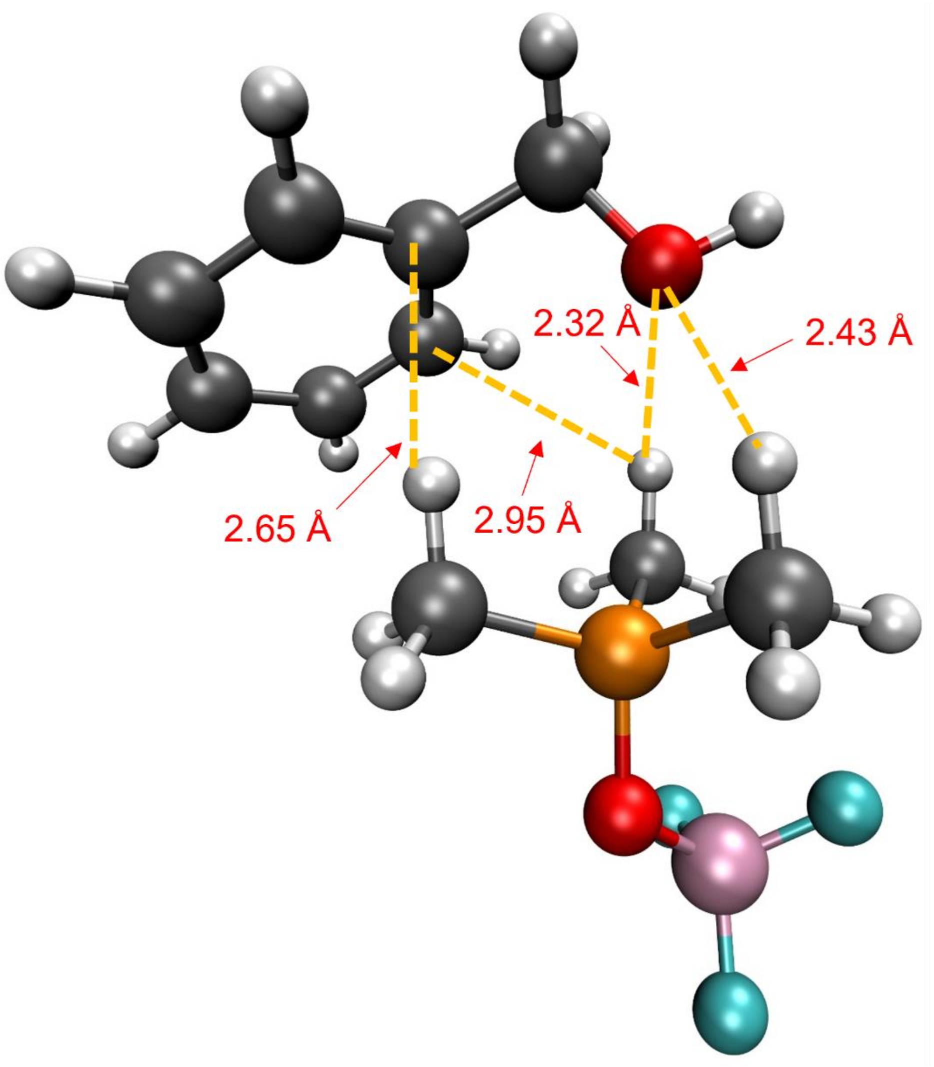

Figure S9. Snapshot of the optimized structure of complex **A**

*Note: Binding energy between **TOB-1** and BnOH (M06-2X/aug-cc-pVTZ, Grimme D3 dispersion, SMD(chloroform)) = -5.12 kcal/mol*

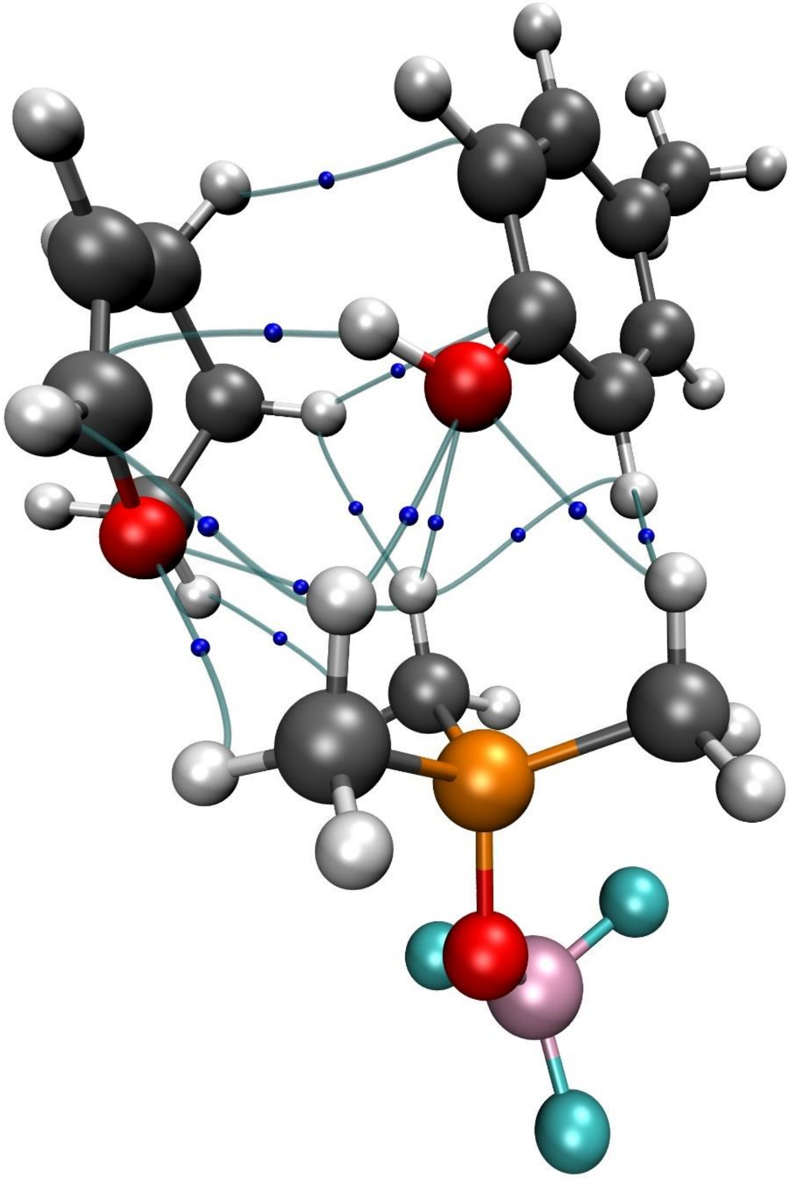

Figure S10. AIM bond paths in complex **B**

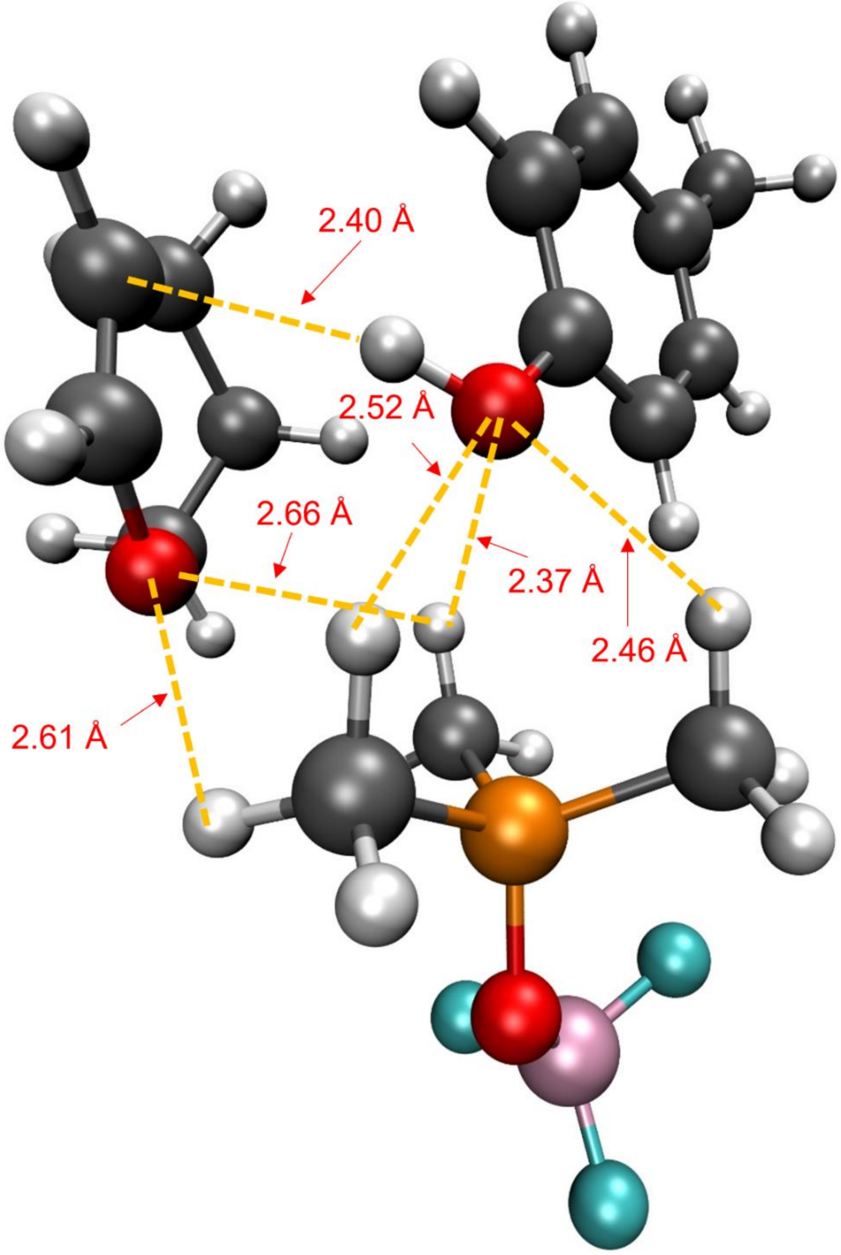

Figure S11. Snapshot of the optimized structure of complex **B**

*Note: Binding energy between **2** and the complex of **TOB-1** and 4-Me-C_6H_4-OH (M06-2X/aug-cc-pVTZ, Grimme D3 dispersion, SMD(toluene)) = -7.97 kcal/mol*

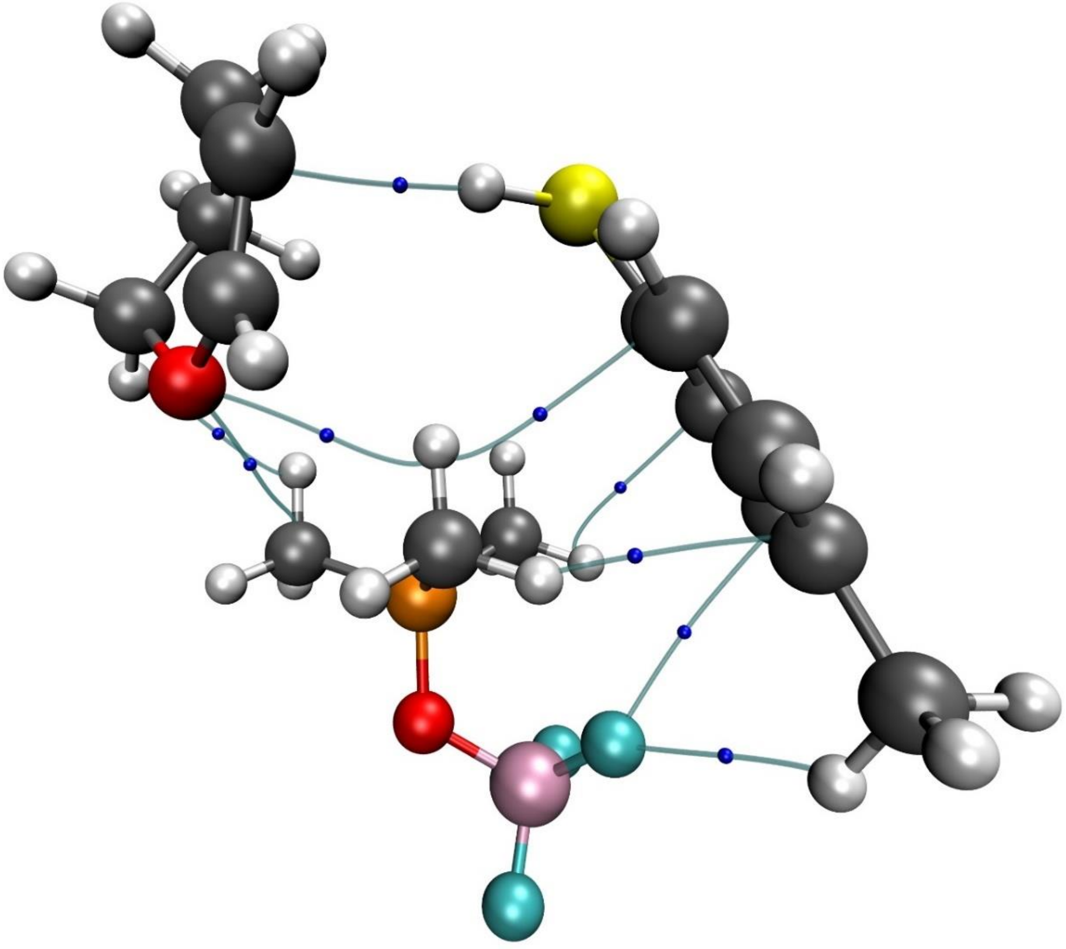

Figure S12. AIM bond paths in complex **C**

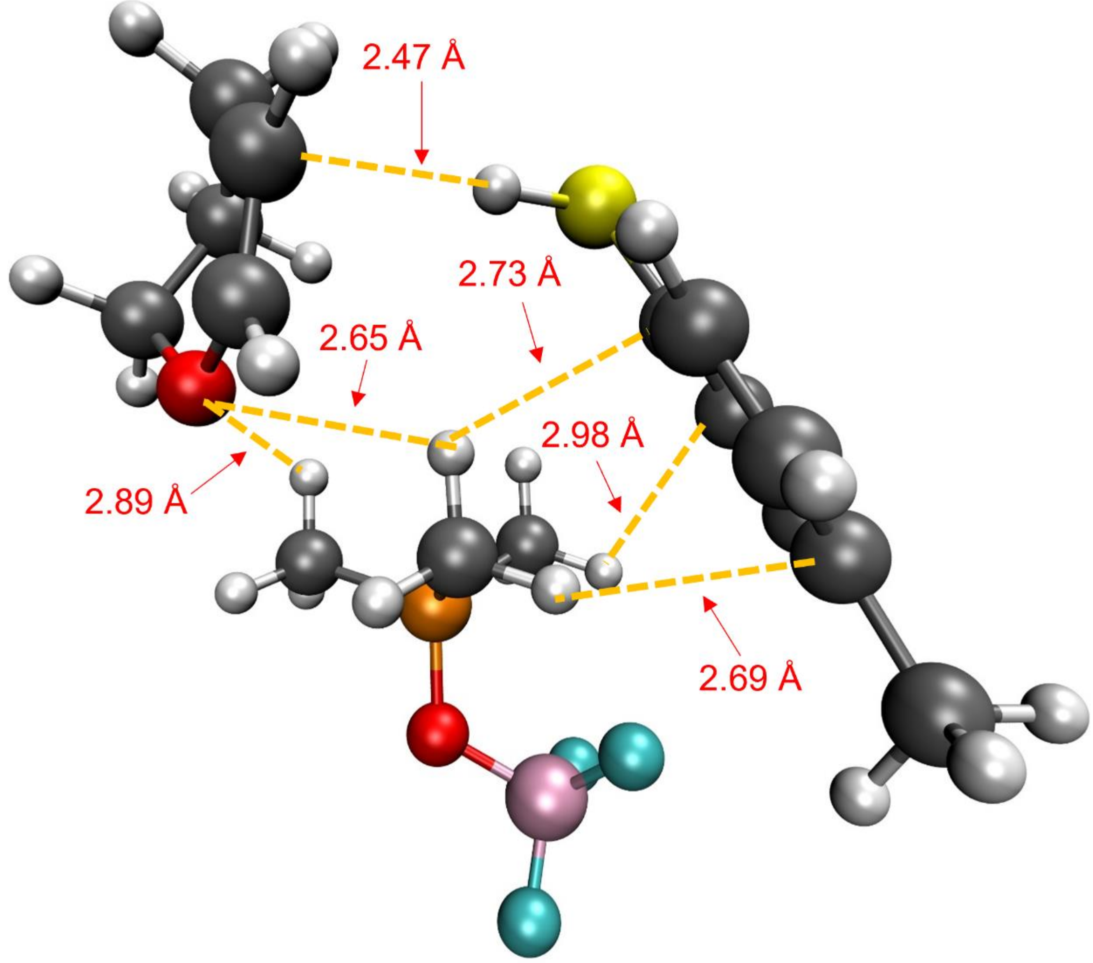

Figure S13. Snapshot of the optimized structure of complex **C**

*Note: Binding energy between **2** and the complex of **TOB-1** and 4-Me-C_6H_4-SH (M06-2X/aug-cc-pVTZ, Grimme D3 dispersion, SMD(toluene)) = -5.64 kcal/mol*

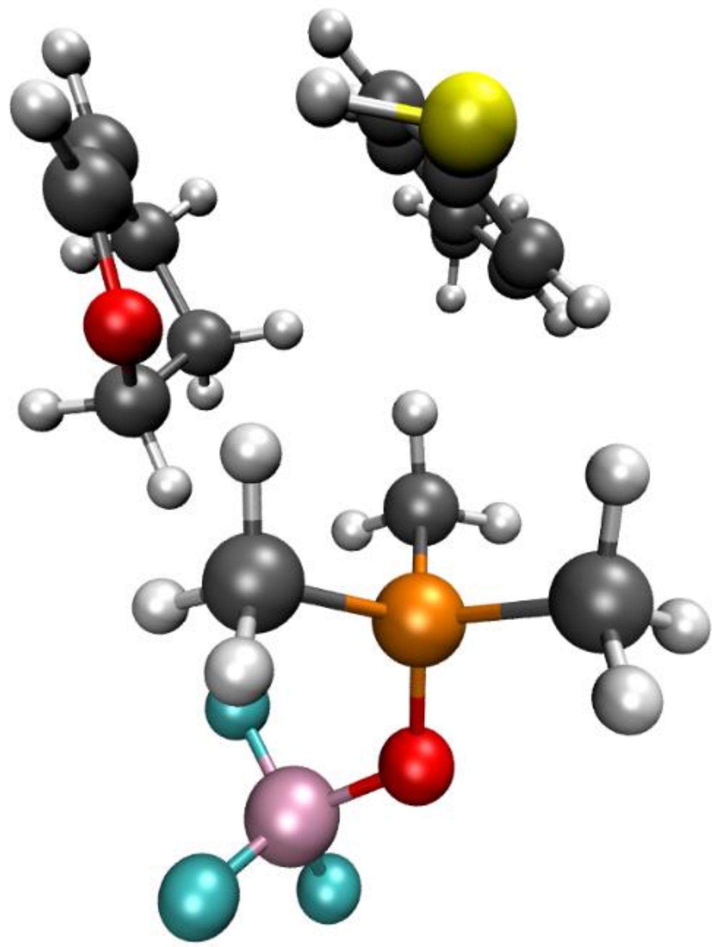

Figure S14. Snapshot of complex **C'** with sulfur positioned close to the C(sp^3)-H

*Note: Binding energy between **2** and the complex of **TOB-1** and 4-Me-C_6H_4-SH (M06-2X/aug-cc-pVTZ, Grimme D3 dispersion, SMD(toluene)) = -4.30 kcal/mol*

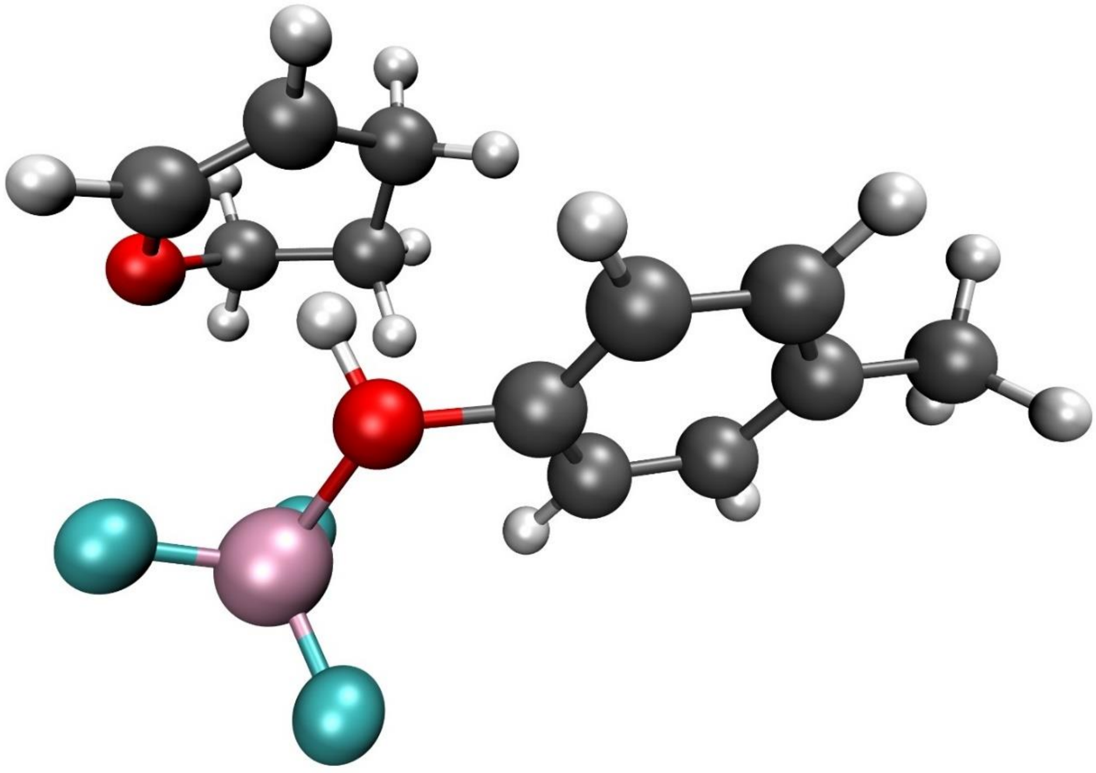

(D)

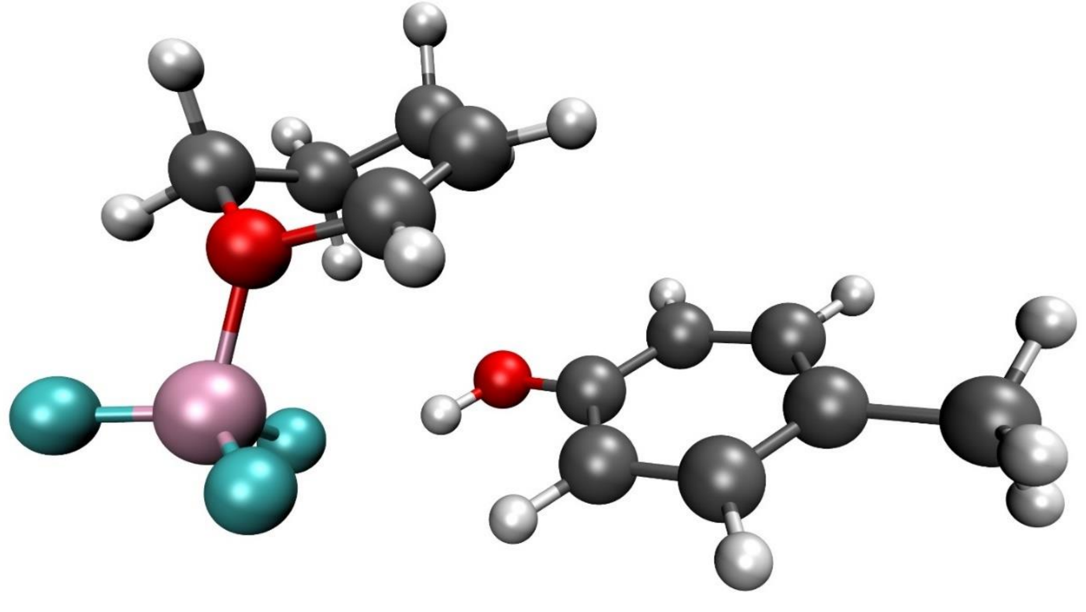

(D')

Figure S15. Snapshots of complexes **D** and **D'**

Note: In the calculation, bilateral complexes ***D*** *(BF_3 complexed with 4-Me-C_6H_4-OH) and* ***D'*** *(BF_3 complexed with* ***2****) were found but no trilateral complex was identified.*

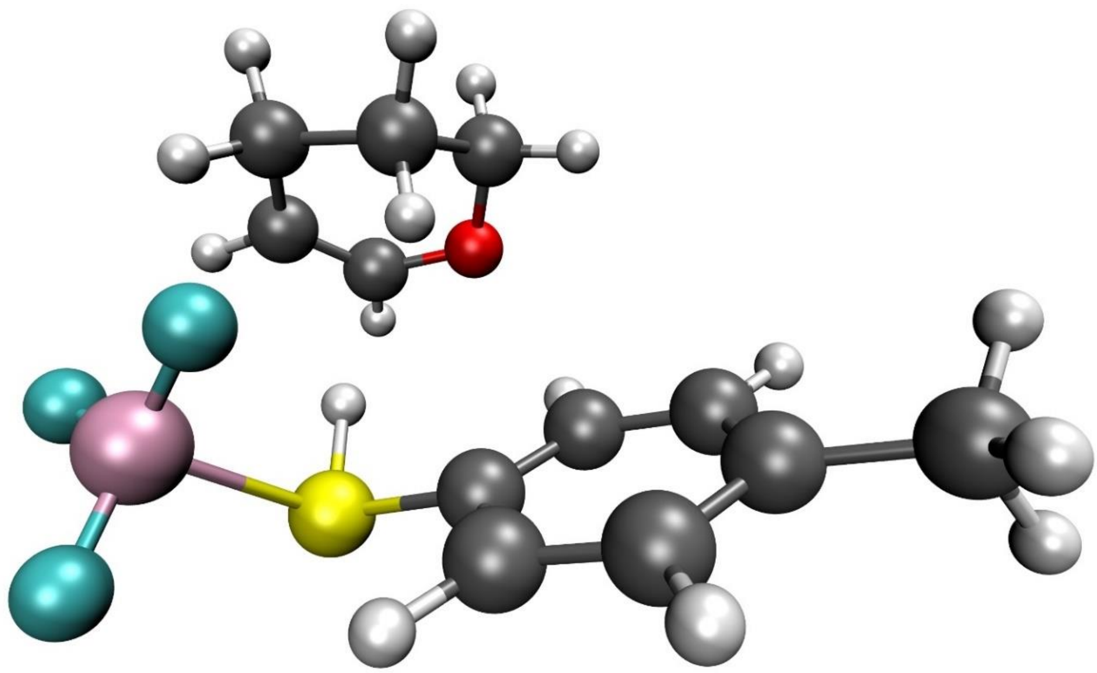

(E)

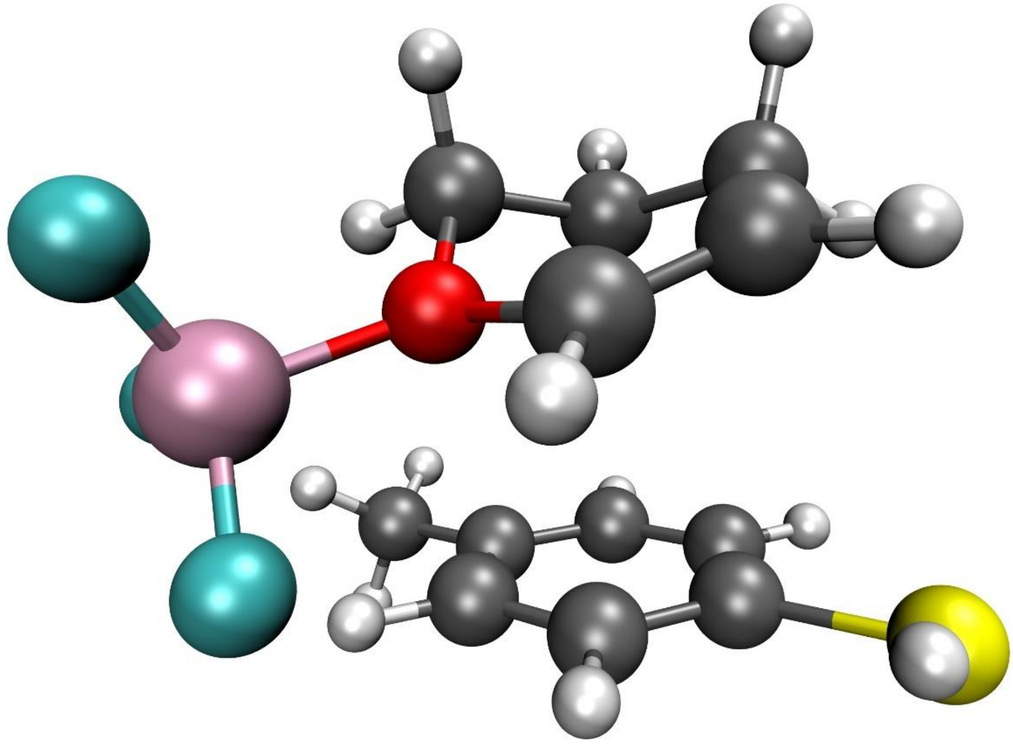

(E')

Figure S16. Snapshots of complexes **E** and **E'**.

*Note: In the calculation, bilateral complexes **E** (BF_3 complexed with 4-Me-C_6H_4-SH) and **E'** (BF_3 complexed with **2**) were found but no trilateral complex was identified.*

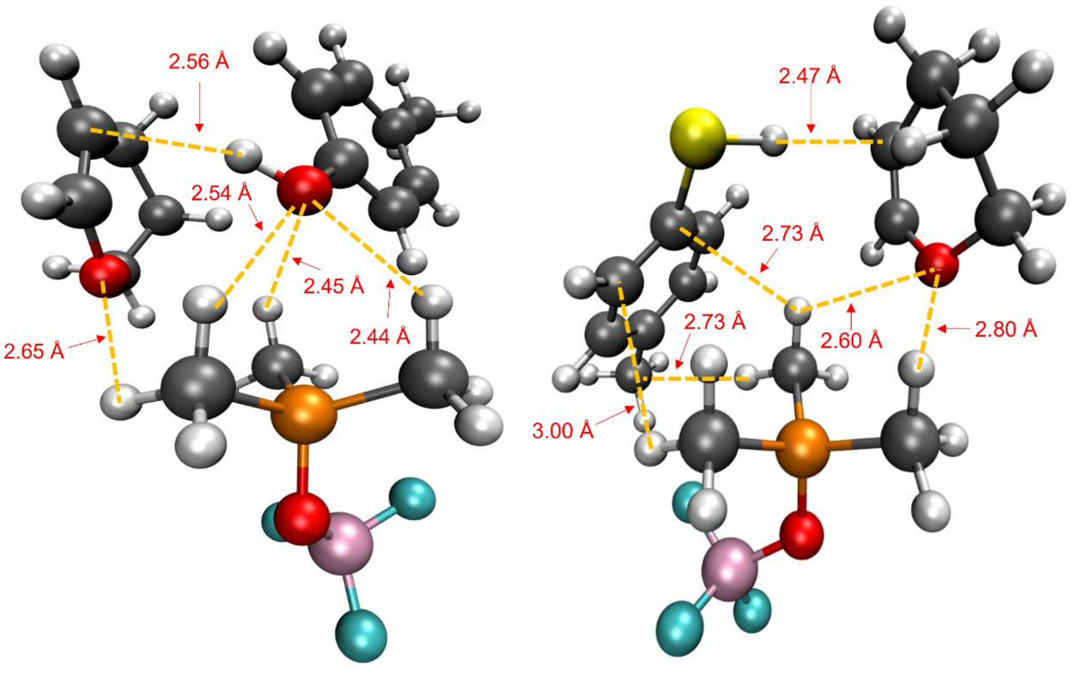

(Complex B in SMD chloroform) **(Complex C in SMD chloroform)**

Figure S17. Snapshots of complexes **B** and **C** in SMD chloroform

*Note: Implicit SMD solvent model investigation was conducted to get a better understanding on the solvent effect. It was observed that the interactions in trilateral complex **B** was considerably interrupted when changing the solvent from toluene to chloroform. In particular, the OH-C(pyran) bond length significantly elongated from 2.40 to 2.56 Å. In contrast, the bond lengths in the trilateral complex **C** are consistent in toluene and chloroform.*

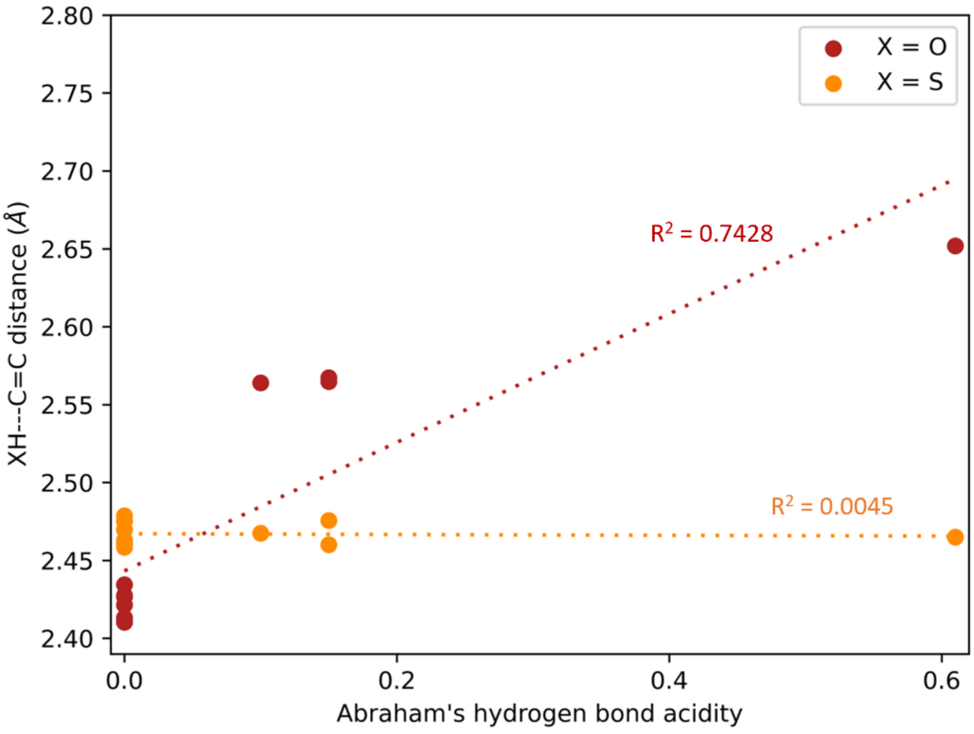

SMD solvent	Abraham's hydrogen bond acidity	OH---C=C (Å)	SH---C=C (Å)
hexane	0.00	2.41	2.48
toluene	0.00	2.41	2.47
chlorobenzene	0.00	2.43	2.46
dibutylether	0.00	2.42	2.46
tetrachloromethane	0.00	2.41	2.48
dichlormethane	0.10	2.56	2.47
chloroform	0.15	2.57	2.48
bromoform	0.15	2.56	2.46
acetic acid	0.61	2.65	2.46

Figure S18. Correlation between Abraham's hydrogen bond acidity[10] of some selected SMD solvents and the intermolecular distances between the 4-Me-C_6H_4-OH/4-Me-C_6H_4-SH and dihydropyran in their trilateral complexes with **TOB-1**

Cartesian coordinates

Complex A

E(M06-2X/aug-cc-pVTZ, Grimme D3 dispersion, SMD(chloroform)) = -1207.79728075

C	3.39670700	-2.23936500	-0.04561900
H	3.44743900	-3.31432400	-0.17469000
C	2.84969000	-1.70157400	1.11866300
H	2.47797000	-2.35806700	1.89723000
C	3.88229800	-1.39445200	-1.03738000
H	4.31231600	-1.80890900	-1.94182100
C	3.81331500	-0.01267900	-0.87048700
H	4.18313100	0.64779400	-1.64822800
C	3.26272900	0.53259700	0.28697200
C	2.78532500	-0.32331500	1.28266100
H	2.36147500	0.09782900	2.18909700
C	3.10253300	2.01919600	0.44314200
H	3.44933500	2.33257100	1.43340600
H	3.69160400	2.54250300	-0.31606300
O	1.71258400	2.32223600	0.29464900
H	1.58479600	3.25131800	0.51846500
P	-1.10850100	0.15685400	-0.45226700
C	-0.75975200	0.37963500	1.28975700
H	-0.61097000	-0.59422400	1.75894900
H	0.14042000	0.99169400	1.37511100
H	-1.60584900	0.88505900	1.75772500
C	0.20006300	-0.78445300	-1.22183500
H	0.26823000	-1.76132300	-0.74114900
H	-0.03348800	-0.91179400	-2.28061700
H	1.14347600	-0.24662000	-1.11147700
C	-1.20577300	1.76417000	-1.23837400
H	-1.48686000	1.62835600	-2.28435400
H	-1.96233400	2.36573600	-0.73391800
H	-0.22794300	2.24501800	-1.17121400
O	-2.42908900	-0.65462100	-0.67227200
B	-3.66866000	-0.41708700	0.14627200
F	-3.78850900	0.96985900	0.35103900
F	-4.74202000	-0.91853400	-0.55966100
F	-3.51310500	-1.05571300	1.37553000

Complex B

E(M06-2X/aug-cc-pVTZ, Grimme D3 dispersion, SMD(toluene)) = -1478.34109403

C	-4.04898080	-2.44064538	0.27616579
C	-2.68236883	-2.71134249	0.38759531
H	-2.35871816	-3.61297490	0.89770295
C	-4.42938244	-1.27396617	-0.38319905
H	-5.48349079	-1.04002328	-0.48918146
C	-3.48506440	-0.40227138	-0.91858805
H	-3.79628141	0.49602654	-1.44312566
C	-2.13114352	-0.68814577	-0.78603593
C	-1.72690419	-1.84925389	-0.13078435
H	-0.66920182	-2.06273834	-0.02653048
C	-5.06758307	-3.39375640	0.84505396
H	-6.07820051	-2.99489355	0.74772147
H	-5.03457663	-4.35627397	0.32775431
H	-4.87911778	-3.58450766	1.90435328
O	-1.16345340	0.14363826	-1.28273085
H	-1.54063311	1.02760783	-1.40918514
P	2.22828799	-0.02266272	-0.74470573
C	1.33536590	0.03699121	0.80922316
H	1.75745313	0.84389304	1.40969570
H	0.28109599	0.22265464	0.60615405
H	1.47862600	-0.90695298	1.33678546
C	1.89690882	1.44814552	-1.71068539
H	2.05708373	2.33191971	-1.09288115
H	2.58072820	1.45868132	-2.56160190
H	0.86673951	1.42049073	-2.06569402
C	1.71426849	-1.45012444	-1.70351962
H	2.33859121	-1.50219154	-2.59806650
H	1.86791917	-2.35401259	-1.11371127
H	0.66746641	-1.34497532	-1.99156422
O	3.76822759	-0.05104644	-0.49870237
B	4.37225649	-0.90405369	0.60232699
F	3.63619585	-2.10490032	0.63640927
F	5.69119115	-1.11798187	0.29139337
F	4.20991660	-0.22586006	1.80565181
C	-0.99247708	3.16010268	-0.69024905
H	-0.48964188	3.44349432	-1.60937898

C	-2.32309788	3.14413920	-0.56368703
H	-2.91638367	3.46730834	-1.41128347
C	-2.99824964	2.72950609	0.71765496
H	-3.84114718	2.06680136	0.50618675
H	-3.41358107	3.61068610	1.21858737
C	-1.98761870	2.02859648	1.62763625
H	-2.35264460	1.97387855	2.65481895
H	-1.82174382	1.00213067	1.28563911
C	-0.67036266	2.78655259	1.60349696
H	0.08674231	2.31677772	2.22958338
H	-0.81778362	3.81850756	1.94172919
O	-0.10858599	2.83370918	0.28429394

Complex C

E(M06-2X/aug-cc-pVTZ, Grimme D3 dispersion, SMD(toluene)) = -1801.30294136

C	-1.64849295	2.99880782	-0.65918862
C	-1.68829436	2.63671442	0.68971572
H	-2.64908257	2.54774245	1.18630468
C	-0.39960635	3.09910150	-1.27276629
H	-0.34105828	3.38837655	-2.31692596
C	0.77447433	2.83234577	-0.57489474
H	1.73390863	2.92343888	-1.07200738
C	0.71299864	2.43457329	0.75805761
C	-0.52560019	2.35230103	1.39357407
H	-0.58134860	2.04876683	2.43318544
C	-2.92627302	3.23285063	-1.41780877
H	-2.72737252	3.53332212	-2.44723202
H	-3.52509276	2.31866448	-1.43333876
H	-3.52643684	4.01298728	-0.94327154
S	2.18175754	2.01236596	1.68299873
H	2.83343839	1.44682472	0.64917196
P	-0.93167475	-1.69229246	0.30436035
C	-1.18650434	-1.04310763	1.95815273
H	-1.56911612	-1.83812787	2.60016477
H	-0.23826314	-0.66855654	2.35105292
H	-1.91847652	-0.23631545	1.91297850
C	0.20687868	-3.07140245	0.40911047
H	-0.25870002	-3.87537597	0.98132347

H	0.44550118	-3.42226275	-0.59585833
H	1.11929669	-2.74163913	0.90903179
C	-0.21066843	-0.42067213	-0.72299208
H	-0.03341825	-0.83600260	-1.71651859
H	-0.91119095	0.41267436	-0.78561307
H	0.73363472	-0.09545418	-0.28459341
O	-2.25885725	-2.23018998	-0.30807589
B	-3.56437558	-1.46470107	-0.16177374
F	-3.25522775	-0.09462837	-0.29664096
F	-4.41582914	-1.89688433	-1.14507247
F	-4.05212360	-1.70339435	1.11745097
C	3.01036762	-0.39039002	-1.64420464
H	2.35795678	0.03474950	-2.39894137
C	4.05930674	0.26011958	-1.13649802
H	4.28618602	1.24400012	-1.53044930
C	4.90571301	-0.32052506	-0.03387048
H	5.08389319	0.43200750	0.74058388
H	5.89189944	-0.60133279	-0.41847420
C	4.19868434	-1.54164616	0.55835529
H	4.88124605	-2.14769917	1.15679400
H	3.37876922	-1.21950844	1.20993370
C	3.62223830	-2.37818620	-0.57274453
H	3.12819843	-3.28136008	-0.21578642
H	4.41517682	-2.66574719	-1.27314779
O	2.62843499	-1.64921982	-1.29898751

Complex C’

E(M06-2X/aug-cc-pVTZ, Grimme D3 dispersion, SMD(toluene)) = -1801.29901922

C	3.95670455	-1.43757971	-1.41485646
C	2.56330351	-1.51431793	-1.46440481
H	2.06980547	-1.68057778	-2.41653748
C	4.56253577	-1.22527045	-0.17525924
H	5.64395034	-1.16540808	-0.11312139
C	3.80413648	-1.09161355	0.98244465
H	4.28890848	-0.93443107	1.93871335
C	2.41436848	-1.15820953	0.91620503
C	1.79651814	-1.38263612	-0.31277883
H	0.71643318	-1.45420552	-0.37266953

C	4.77745104	-1.60447210	-2.66529488
H	5.79423989	-1.23658779	-2.52319677
H	4.83812001	-2.65965401	-2.94630079
H	4.32962642	-1.06655918	-3.50316796
P	-2.61923107	-0.70563886	0.86922269
C	-1.45585550	0.54179785	0.31953071
H	-1.83945337	1.53215237	0.56626608
H	-0.49569236	0.38962955	0.81663381
H	-1.34235558	0.46350355	-0.76287995
C	-2.85239460	-0.54587443	2.63732090
H	-3.22119734	0.45670283	2.85929855
H	-3.58485148	-1.28263192	2.97097923
H	-1.90423837	-0.71366698	3.15177902
C	-1.95492297	-2.33748574	0.52813334
H	-2.68898454	-3.08480622	0.83654900
H	-1.78292211	-2.43310728	-0.54435133
H	-1.02637003	-2.48643201	1.08291893
O	-4.01524166	-0.52185770	0.20502077
B	-4.13476017	-0.17215469	-1.27192655
F	-3.12669560	-0.89673108	-1.93945700
F	-5.38993215	-0.53269163	-1.68614598
F	-3.89141496	1.18919046	-1.40646469
C	1.20756033	2.78874023	1.35537092
H	0.96117684	2.90851258	2.40556133
C	2.39490706	2.35189279	0.93063848
H	3.15187178	2.12922154	1.67421127
C	2.69372732	2.14934620	-0.53019808
H	3.22393452	1.20428647	-0.67431747
H	3.35719007	2.94121203	-0.89557597
C	1.38294321	2.14616160	-1.31701501
H	1.55808771	2.26585002	-2.38822922
H	0.87323545	1.19006645	-1.17089314
C	0.48243498	3.27066525	-0.82897128
H	-0.47665501	3.28206161	-1.34664699
H	0.97545749	4.23919378	-0.97510873
O	0.16624195	3.14275319	0.56151254
S	1.43706346	-1.01275860	2.41496044
H	1.37380930	0.33322912	2.40316142

Complex D

E(M06-2X/aug-cc-pVTZ, Grimme D3 dispersion, SMD(toluene)) = -941.949174058

C	3.62474421	-0.36020727	0.12917177
C	3.07150927	-0.55711869	-1.13886150
H	3.66211643	-1.03639894	-1.91223590
C	2.84681202	0.26842050	1.10018001
H	3.25857624	0.43399265	2.08938170
C	1.54510136	0.68341219	0.83527963
H	0.94063416	1.15901939	1.59660064
C	1.03593858	0.46906968	-0.43246359
C	1.78225870	-0.13522036	-1.43192107
H	1.35654046	-0.26500142	-2.42017307
C	5.03614811	-0.79593871	0.42045108
H	5.25094985	-0.74946374	1.48856285
H	5.20951125	-1.81904184	0.08006181
H	5.75278272	-0.15105290	-0.09528066
O	-0.27717259	0.86044353	-0.74664388
H	-0.84138307	0.08758136	-1.02178053
C	-2.66013274	-0.99755268	-1.05260787
H	-3.15514842	-0.48124151	-1.86855478
C	-1.57244229	-1.76663595	-1.23178047
H	-1.23064981	-1.92957146	-2.24771347
C	-0.87780229	-2.43831294	-0.07290924
H	0.20764389	-2.36382810	-0.18502083
H	-1.11810711	-3.50671326	-0.06269495
C	-1.32459185	-1.77706150	1.23285873
H	-1.05380990	-2.38662106	2.09683119
H	-0.84336338	-0.80356608	1.35254567
C	-2.82983448	-1.57141619	1.21947114
H	-3.17789528	-1.05861093	2.11404660
H	-3.34782819	-2.53333075	1.13185647
O	-3.25380302	-0.74803586	0.12353388
B	-1.12252672	2.00879596	0.03717962
F	-2.23347956	2.10588801	-0.74743078
F	-1.37023057	1.51063533	1.29227991
F	-0.29366574	3.08702986	0.03307725

Complex D'

E(M06-2X/aug-cc-pVTZ, Grimme D3 dispersion, SMD(toluene)) = -941.955591108

C	-3.69058176	-0.43482359	-0.32111001
C	-3.58265415	0.84702026	0.22157844
H	-4.38255396	1.56252797	0.05859876
C	-2.64492871	-1.32841552	-0.08567702
H	-2.69928763	-2.33265616	-0.49393559
C	-1.52939453	-0.96401397	0.65888307
H	-0.71770407	-1.66804126	0.81354316
C	-1.43835972	0.32443748	1.18246870
C	-2.47452884	1.22960377	0.96716030
H	-2.39532573	2.22651223	1.38531653
C	-4.91243926	-0.84942577	-1.09994140
H	-4.67812366	-1.64703890	-1.80733104
H	-5.69611044	-1.22001508	-0.43239839
H	-5.32814849	-0.00966966	-1.66046909
O	-0.35817295	0.74955473	1.88820530
H	0.32255022	0.06370455	1.87531931
C	0.90762774	0.12649352	-1.40929849
H	0.49294775	-0.75488542	-1.87322698
C	0.34818038	1.31930975	-1.32249186
H	-0.62786530	1.42841483	-1.78206471
C	0.95740307	2.48848140	-0.60729405
H	0.21116091	2.91136121	0.06988756
H	1.20150284	3.26757308	-1.33714111
C	2.19875013	2.06673774	0.17892705
H	2.83974580	2.92377893	0.39342337
H	1.90592616	1.62329711	1.13093546
C	3.00345120	1.05601451	-0.60981216
H	3.87672105	0.68552135	-0.07934677
H	3.29584150	1.43195062	-1.59153923
O	2.19190819	-0.13319349	-0.88086158
B	2.32308331	-1.36871150	0.13751660
F	1.53950207	-2.34099308	-0.40661821
F	3.65476269	-1.63743625	0.17798787
F	1.83220983	-0.89581348	1.34682375

Complex E

E(M06-2X/aug-cc-pVTZ, Grimme D3 dispersion, SMD(toluene)) = -1264.89587822

C	-3.56633095	-0.47433782	0.17425216
C	-3.21128550	0.86172740	0.35379508
H	-3.88860303	1.53028770	0.87375792
C	-2.66721984	-1.31507811	-0.48649392
H	-2.92021504	-2.36045329	-0.63054237
C	-1.45424115	-0.84032722	-0.96686036
H	-0.76217098	-1.51039022	-1.46500055
C	-1.12407131	0.49736114	-0.76774871
C	-1.99564332	1.35417659	-0.10735029
H	-1.72754754	2.39103477	0.05967659
C	-4.89073101	-0.99933166	0.65945052
H	-4.78714474	-2.00280168	1.07652643
H	-5.31601059	-0.35022425	1.42584290
H	-5.60632002	-1.05696079	-0.16576935
S	0.41796124	1.16215251	-1.39124798
H	1.19629901	0.08340753	-1.14894771
C	2.45167018	-1.92123203	-1.05112078
H	2.74225523	-1.98264760	-2.09426410
C	3.10978227	-1.18919310	-0.15018725
H	3.99227264	-0.65326337	-0.47839097
C	2.65111766	-1.07007884	1.27836660
H	2.68920454	-0.02454428	1.59236059
H	3.33298655	-1.62005445	1.93615037
C	1.22548480	-1.61016216	1.41049346
H	0.97833381	-1.82248187	2.45261779
H	0.50589818	-0.86603444	1.05831863
C	1.07518302	-2.88162831	0.59083855
H	0.06215615	-3.28079300	0.63797445
H	1.77564008	-3.64784797	0.94390603
O	1.33572183	-2.65718043	-0.80044233
B	1.19119974	2.30505676	0.35681174
F	2.51633296	2.21904264	0.10545176
F	0.73693389	1.58616696	1.40671299
F	0.61134686	3.51035833	0.17457087

Complex E'

E(M06-2X/aug-cc-pVTZ, Grimme D3 dispersion, SMD(toluene)) = -1264.91626130

C	1.30753368	2.31388544	0.14828778
C	2.47310331	1.76522846	0.68562424
H	3.03162798	2.32186559	1.43125698
C	0.61407359	1.56917323	-0.80745108
H	-0.29805363	1.96610171	-1.23968628
C	1.06401791	0.32053444	-1.21604771
H	0.50169085	-0.22786185	-1.96349655
C	2.21721502	-0.22418634	-0.65316924
C	2.92547834	0.50803528	0.29891112
H	3.82169661	0.09369969	0.74754323
C	0.81218737	3.67333194	0.56532558
H	-0.27936268	3.70095005	0.58043738
H	1.18040477	3.94334836	1.55652561
H	1.15068638	4.44130099	-0.13622888
S	2.83600955	-1.82658699	-1.12655154
H	1.68046058	-2.27617003	-1.64086834
C	-1.17835123	-1.78506593	-0.15655149
H	-1.59183857	-2.04755164	-1.11921498
C	-0.39657796	-2.53695548	0.60020982
H	-0.16111333	-3.52768659	0.22987842
C	0.17041803	-2.08882303	1.91645347
H	1.24811674	-2.27369371	1.92371258
H	-0.25791771	-2.70223376	2.71610927
C	-0.10961019	-0.60418438	2.15789638
H	-0.03597313	-0.35717489	3.21856296
H	0.61426183	0.01123413	1.62055050
C	-1.49451135	-0.23501386	1.67426380
H	-1.70845622	0.82506129	1.77648384
H	-2.28179923	-0.83117940	2.14289891
O	-1.56585193	-0.49932949	0.24088253
B	-2.62968340	0.30182718	-0.68127437
F	-2.13000834	0.13654598	-1.94437919
F	-2.57170584	1.58302778	-0.21083249
F	-3.82553144	-0.32011563	-0.46954881

Complex B (in SMD chloroform)

E(M06-2X/aug-cc-pVTZ, Grimme D3 dispersion, SMD(chloroform)) = -1478.35127719

C	-3.88022703	-2.44556144	0.30159579
C	-2.50982491	-2.72332360	0.30080941
H	-2.14801320	-3.61634612	0.80077244
C	-4.31081791	-1.29465713	-0.35557744
H	-5.36956814	-1.05793262	-0.37976022
C	-3.41140414	-0.44057440	-0.98886132
H	-3.76116262	0.44875274	-1.50399202
C	-2.05120080	-0.72706083	-0.95798634
C	-1.59801304	-1.87890488	-0.31682557
H	-0.53652038	-2.09876846	-0.30173683
C	-4.84932828	-3.37058595	0.99114960
H	-5.87495413	-3.01380854	0.88686607
H	-4.79640084	-4.37839414	0.57131409
H	-4.62461259	-3.44861855	2.05812268
O	-1.12455999	0.09450072	-1.54044510
H	-1.53198669	0.95994470	-1.69505960
P	2.23217246	0.07488150	-0.74390444
C	1.25216223	0.01517795	0.75479124
H	1.61313430	0.79839280	1.42305089
H	0.20683000	0.19198647	0.50171093
H	1.38156606	-0.95611325	1.23420588
C	1.90615196	1.58485686	-1.64419642
H	2.00172525	2.43672227	-0.97053269
H	2.63230588	1.66281987	-2.45567781
H	0.89771197	1.54407796	-2.05550789
C	1.85347939	-1.31970669	-1.80506182
H	2.54317703	-1.29807102	-2.65163320
H	1.99683060	-2.24728143	-1.25056205
H	0.82656733	-1.23795302	-2.16337168
O	3.76010852	0.09395558	-0.39814398
B	4.35996604	-0.79607275	0.65492090
F	3.71771613	-2.04616535	0.57693464
F	5.71030593	-0.90512370	0.39530939
F	4.11934305	-0.22874529	1.90527876
C	-1.17029816	3.06137969	-0.62299170

H	-0.66892702	3.37474248	-1.53340784
C	-2.49956923	3.01401431	-0.50042645
H	-3.09810043	3.33674193	-1.34472850
C	-3.16831536	2.54749556	0.76605049
H	-3.99634517	1.87439801	0.53010222
H	-3.60232648	3.40272637	1.29572098
C	-2.14314528	1.84017603	1.65387176
H	-2.50918811	1.73562118	2.67699136
H	-1.94294904	0.83453239	1.26889507
C	-0.84883331	2.63650251	1.66338002
H	-0.08308728	2.17055600	2.28179352
H	-1.02999876	3.65335001	2.03026173
O	-0.28046195	2.73779424	0.35035059

Complex C (in SMD chloroform)

E(M06-2X/aug-cc-pVTZ, Grimme D3 dispersion, SMD(chloroform)) = -1801.31262297

C	-1.46626012	3.12721199	-0.63537426
C	-1.55121837	2.73975233	0.70453702
H	-2.52118495	2.71869159	1.19138067
C	-0.20591828	3.14062609	-1.23406967
H	-0.11193225	3.44531919	-2.27133480
C	0.93571124	2.77246793	-0.52832588
H	1.90519193	2.79946456	-1.01368166
C	0.82749321	2.35794869	0.79708296
C	-0.42243989	2.35302331	1.41598323
H	-0.51577651	2.03342043	2.44818082
C	-2.70927472	3.48973463	-1.40237660
H	-2.46990734	3.79103100	-2.42291667
H	-3.39061410	2.63612356	-1.44618897
H	-3.24258651	4.31120115	-0.91759036
S	2.25069020	1.82546516	1.73542625
H	2.90661814	1.28300543	0.69150334
P	-1.03044199	-1.67406473	0.28262904
C	-1.26165973	-1.02503492	1.93816111
H	-1.66628383	-1.81189230	2.57695881
H	-0.29697965	-0.69074996	2.32701792
H	-1.95956372	-0.18798782	1.90401865

C	0.06753265	-3.08260745	0.36813060
H	-0.40864680	-3.86990444	0.95467728
H	0.27638154	-3.44180357	-0.64055356
H	0.99806579	-2.77217081	0.84647919
C	-0.29537227	-0.41561422	-0.75009872
H	-0.14108189	-0.82897285	-1.74852204
H	-0.96841406	0.44120913	-0.79701780
H	0.66311000	-0.12507169	-0.31894790
O	-2.38336043	-2.17639728	-0.32059513
B	-3.66561038	-1.39764038	-0.17950252
F	-3.35696783	-0.02941133	-0.31600991
F	-4.52652794	-1.82196773	-1.16797750
F	-4.17987407	-1.62889755	1.09412784
C	2.96469532	-0.43955079	-1.60992521
H	2.28288938	0.00269838	-2.32824191
C	4.07252799	0.17042839	-1.18274696
H	4.31297636	1.13958131	-1.60461190
C	4.96491897	-0.43185908	-0.12894061
H	5.22046259	0.32298270	0.62140635
H	5.91266203	-0.75480871	-0.57270237
C	4.25285364	-1.61832123	0.52498419
H	4.95223459	-2.24774203	1.07820395
H	3.49563026	-1.25992343	1.23049840
C	3.56248074	-2.44094793	-0.55054108
H	3.05453184	-3.31517706	-0.14478004
H	4.29022666	-2.77130173	-1.30087435
O	2.55205235	-1.67567131	-1.21706095

10. References

(1) M. Nie, L. Madec, J. Xia, D. S. Hall, J. R. Dahn, *J. Power Sources* 2016, **328**, 433.

(2) Y. Zhao, D. G. Truhlar, *Theor. Chem. Acc*. 2008, **120**, 215–241.

(3) S. Grimme, J. Antony, S. Ehrlich, H. Krieg, *J. Chem. Phys.* 2010, **132**, 154104.

(4) A. V. Marenich, C. J. Cramer, D. G. Truhlar, *J. Phys. Chem. B.* 2009, **113**, 6378–6396.

(5) M. J. Frisch, G. W. Trucks, H. B. Schlegel, G. E. Scuseria, M. A. Robb, J. R. Cheeseman, G. Scalmani, V. Barone, G. A. Petersson, H. Nakatsuji, X. Li, M. Caricato, A. V. Marenich, J. Bloino, B. G. Janesko, R. Gomperts, B. Mennucci, H. P. Hratchian, J. V. Ortiz, A. F. Izmaylov, J. L. W. Sonnenberg, F. Ding, F. Lipparini, F. Egidi, J. Goings, B. Peng, A. Petrone, T. Henderson, D. Ranasinghe, V. G. Zakrzewski, J. Gao, N. Rega, G. Zheng, W. Liang, M. Hada, M. Ehara, K. Toyota, R. Fukuda, J. Hasegawa, M. Ishida, T. Nakajima, Y. Honda, O. Kitao, H. Nakai, T. Vreven, K. Throssell, J. A. Montgomery Jr., J. E. Peralta, F. Ogliaro, M. J. Bearpark, J. J. Heyd, E. N. Brothers, K. N. Kudin, V. N. Staroverov, T. A. Keith, R. Kobayashi, J. Normand, K. Raghavachari, A. P. Rendell, J. C. Burant, S. S. Iyengar, J. Tomasi, M. Cossi, J. M. Millam, M. Klene, C. Adamo, R. Cammi, J. W. Ochterski, R. L. Martin, K. Morokuma, O. Farkas, J. B. Foresman, D. J. Fox, *Gaussian 16*, C.02.; Gaussian, Inc.: Wallingford, CT, 2016.

(6) S. F. Boys, F. J. M. P. Bernardi, *Mol. Phy.* 1970, **19**, 553-566.

(7) a) E. D. Glendening, J, K. Badenhoop, A. E. Reed, J. E. Carpenter, J. A. Bohmann, C. M. Morales, P. Karafiloglou, C. R. Landis, F. Weinhold, NBO 7.0 (University of Wisconsin, 2018), https://nbo7.chem.wisc.edu/biblio_css.htm; b) F. Weinhold, C. R. Landis, E. D. Glendening, *Int. Rev. Phy. Chem.* 2016, **35**, 399–440.

(8) R. F. W. Bader, In Atoms in Molecules: A Quantum Theory; Clarendon Press: Oxford, 1990.

(9) a) T. Lu, F. Chen, *J. Comput. Chem.* 2012, **33**, 580-592; b) Z. Zhang, T. Lu, *Phys. Chem. Chem. Phys.* 2021, **23**, 20323-20328.

(10) M. H. Abraham, *Chem. Soc. Rev.* 1993, **22**, 73-83.

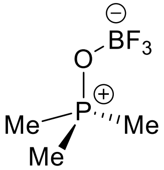

TOB-1

500 MHz ^{1}H NMR

$CDCl_3$

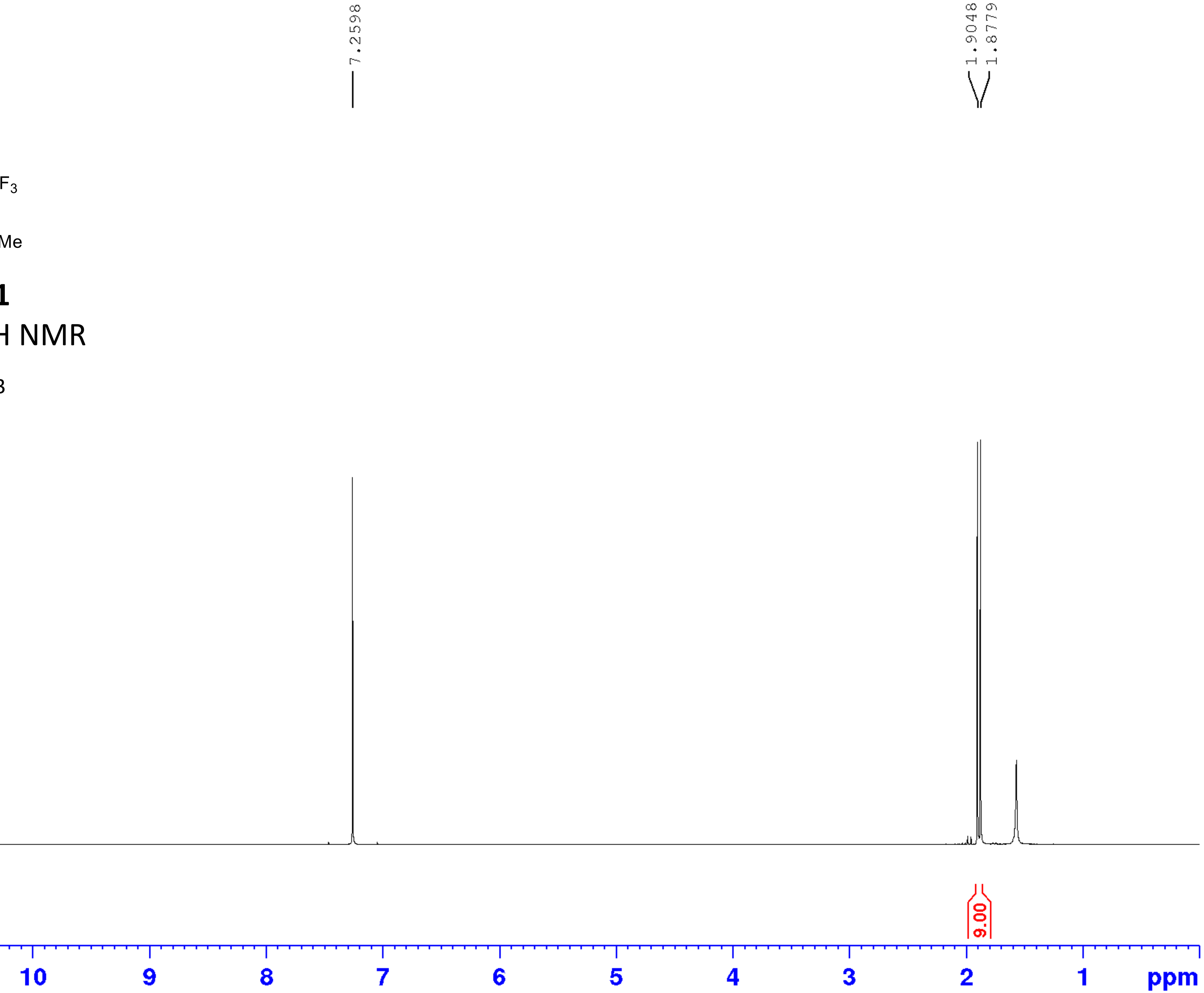


```
Current Data Parameters
NAME      vinn-4-046-2nd-PMe3OBF3-2020101
EXPNO                 1
PROCNO                1

F2 - Acquisition Parameters
Date_          20201015
Time              22.33 h
INSTRUM           spect
PROBHD   Z119470_0283 (
PULPROG            zg30
TD                65536
SOLVENT           CDCl3
NS                   16
DS                    2
SWH           10000.000 Hz
FIDRES         0.305176 Hz
AQ            3.2767999 sec
RG               206.72
DW               50.000 usec
DE                 6.50 usec
TE                295.2 K
D1           1.00000000 sec
TD0                   1
SFO1        500.1330883 MHz
NUC1                 1H
P1                10.91 usec
PLW1        25.00000000 W

F2 - Processing parameters
SI                65536
SF          500.1300124 MHz
WDW                  EM
SSB                   0
LB                 0.30 Hz
GB                    0
PC                 1.00
```

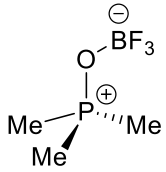

TOB-1

125 MHz ^{13}C NMR

$CDCl_3$

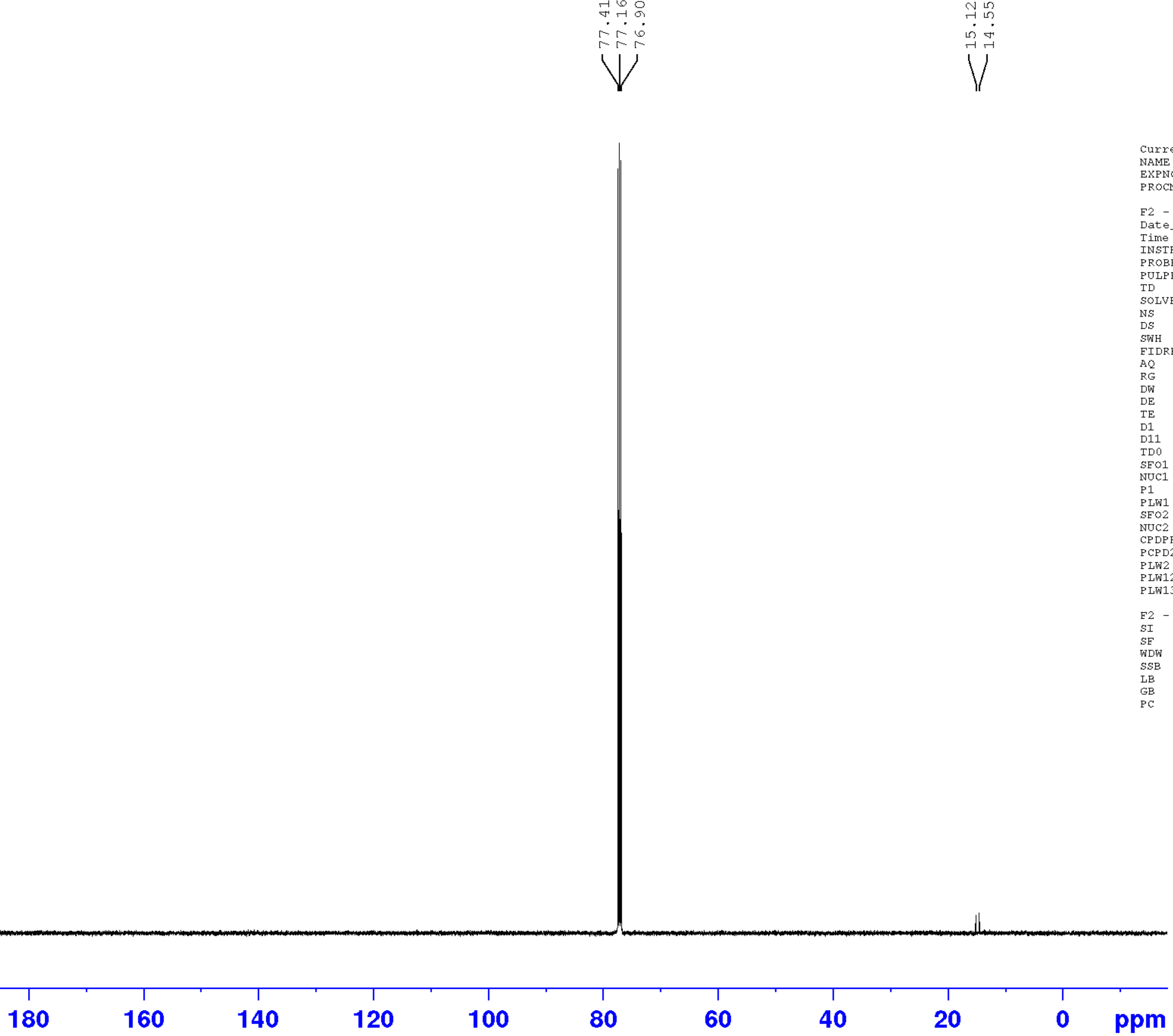


```
Current Data Parameters
NAME       vinn-4-046-2nd-PMe3OBF3-202
EXPNO                    2
PROCNO                   1

F2 - Acquisition Parameters
Date_             20201015
Time                 23.28 h
INSTRUM              spect
PROBHD   Z119470_0283 (
PULPROG             zgpg30
TD                   65536
SOLVENT              CDCl3
NS                    1024
DS                       4
SWH              29761.904 Hz
FIDRES            0.908261 Hz
AQ               1.1010048 sec
RG                  206.72
DW                  16.800 usec
DE                    6.50 usec
TE                   295.1 K
D1              2.00000000 sec
D11             0.03000000 sec
TD0                      1
SFO1           125.7703643 MHz
NUC1                   13C
P1                    9.75 usec
PLW1           94.00000000 W
SFO2           500.1320005 MHz
NUC2                    1H
CPDPRG[2           waltz16
PCPD2                80.00 usec
PLW2           25.00000000 W
PLW12           0.46495000 W
PLW13           0.23387000 W

F2 - Processing parameters
SI                   32768
SF             125.7577726 MHz
WDW                     EM
SSB                      0
LB                    1.00 Hz
GB                       0
PC                    1.40
```

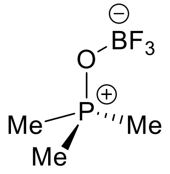

TOB-1

160 MHz ^{11}B NMR

$CDCl_3$

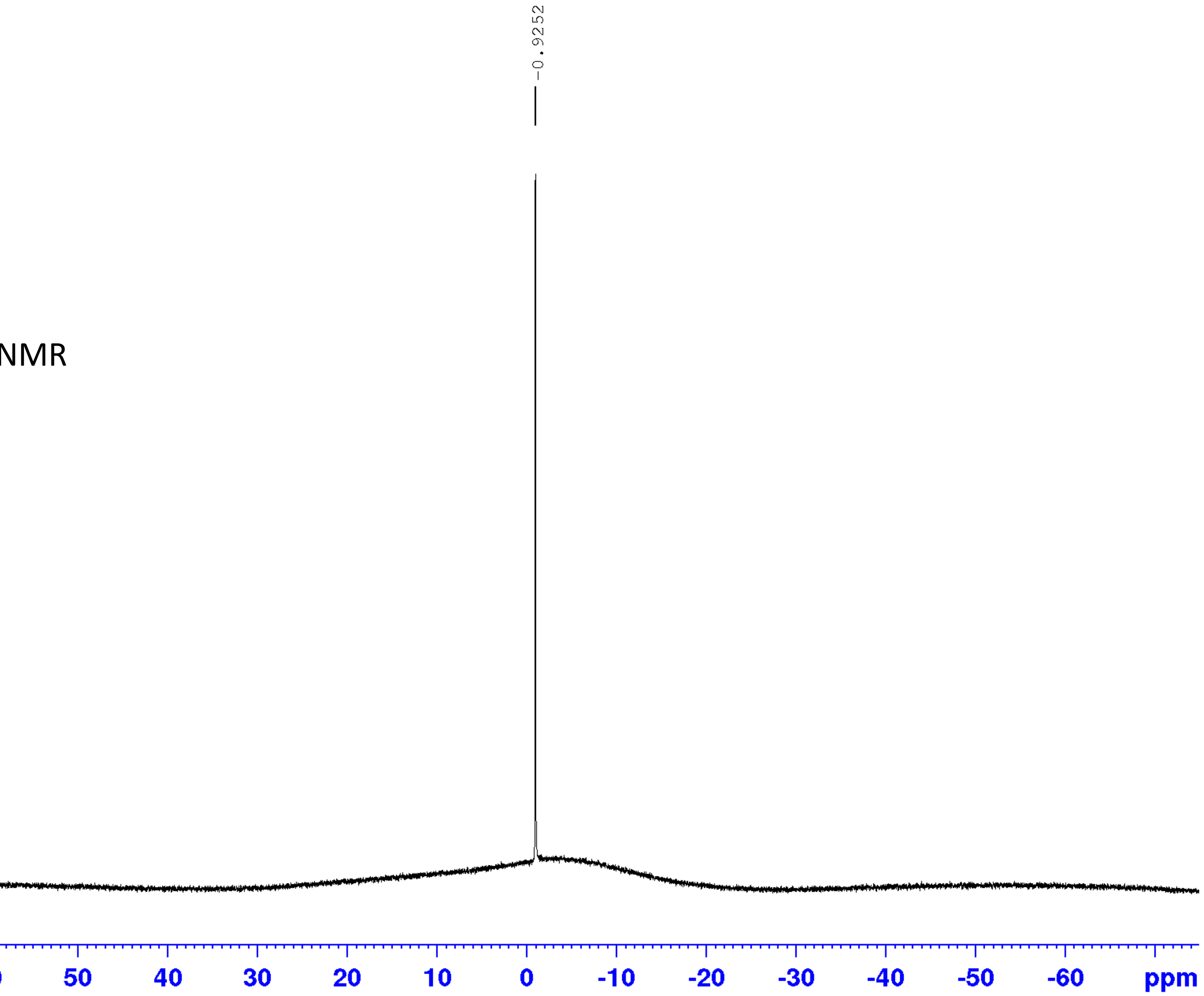


```
Current Data Parameters
NAME      vinn-4-046-2nd-PMe3OBF3-20201015
EXPNO                    3
PROCNO                   1

F2 - Acquisition Parameters
Date_             20201015
Time                 23.29 h
INSTRUM              spect
PROBHD   Z119470_0283 (
PULPROG               zgig
TD                   32768
SOLVENT              CDCl3
NS                      16
DS                       4
SWH              24038.461 Hz
FIDRES            1.467191 Hz
AQ               0.6815744 sec
RG                  206.72
DW                  20.800 usec
DE                    6.50 usec
TE                   295.2 K
D1              1.00000000 sec
D11             0.03000000 sec
TD0                      1
SFO1           160.4615790 MHz
NUC1                   11B
P1                   16.00 usec
PLW1           50.00000000 W
SFO2           500.1320005 MHz
NUC2                    1H
CPDPRG[2          waltz16
PCPD2                80.00 usec
PLW2           25.00000000 W
PLW12           0.46495000 W

F2 - Processing parameters
SI                   16384
SF             160.4615792 MHz
WDW                     EM
SSB                      0
LB                    1.00 Hz
GB                       0
PC                    1.40
```

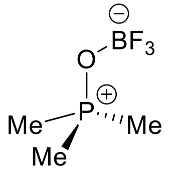

TOB-1

470 MHz ^{19}F NMR

$CDCl_3$

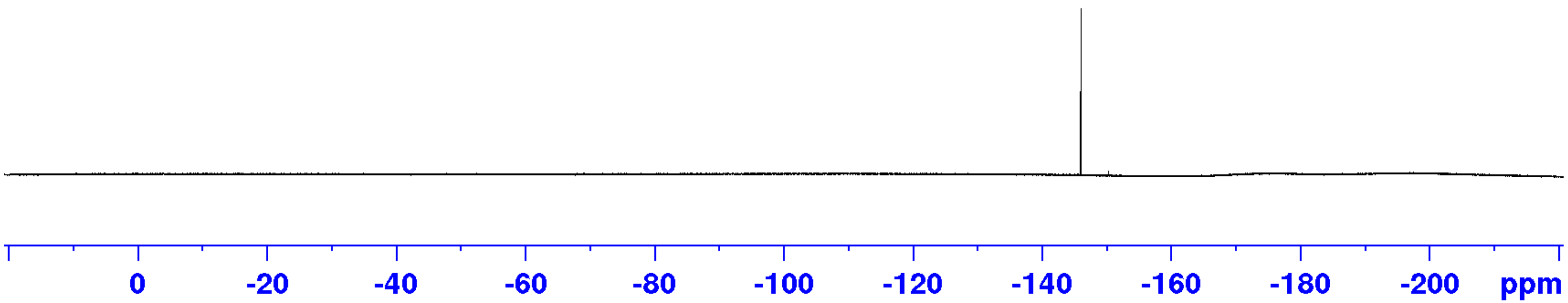


```
Current Data Parameters
NAME      vinn-4-046-2nd-PMe3OBF3-20201015
EXPNO                       4
PROCNO                      1

F2 - Acquisition Parameters
Date_              20201015
Time                  23.31 h
INSTRUM               spect
PROBHD   Z119470_0283 (
PULPROG              zgflqn
TD                   131072
SOLVENT               CDCl3
NS                       32
DS                        4
SWH             113636.367 Hz
FIDRES            1.733953 Hz
AQ               0.5767168 sec
RG                   206.72
DW                    4.400 usec
DE                     6.50 usec
TE                    295.2 K
D1               1.00000000 sec
TD0                       1
SFO1            470.5453180 MHz
NUC1                    19F
P1                    15.00 usec
PLW1            47.23500061 W

F2 - Processing parameters
SI                    65536
SF              470.5923772 MHz
WDW                      EM
SSB                       0
LB                     0.30 Hz
GB                        0
PC                     1.00
```

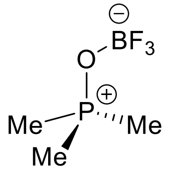

TOB-1

200 MHz ^{31}P NMR

$CDCl_3$

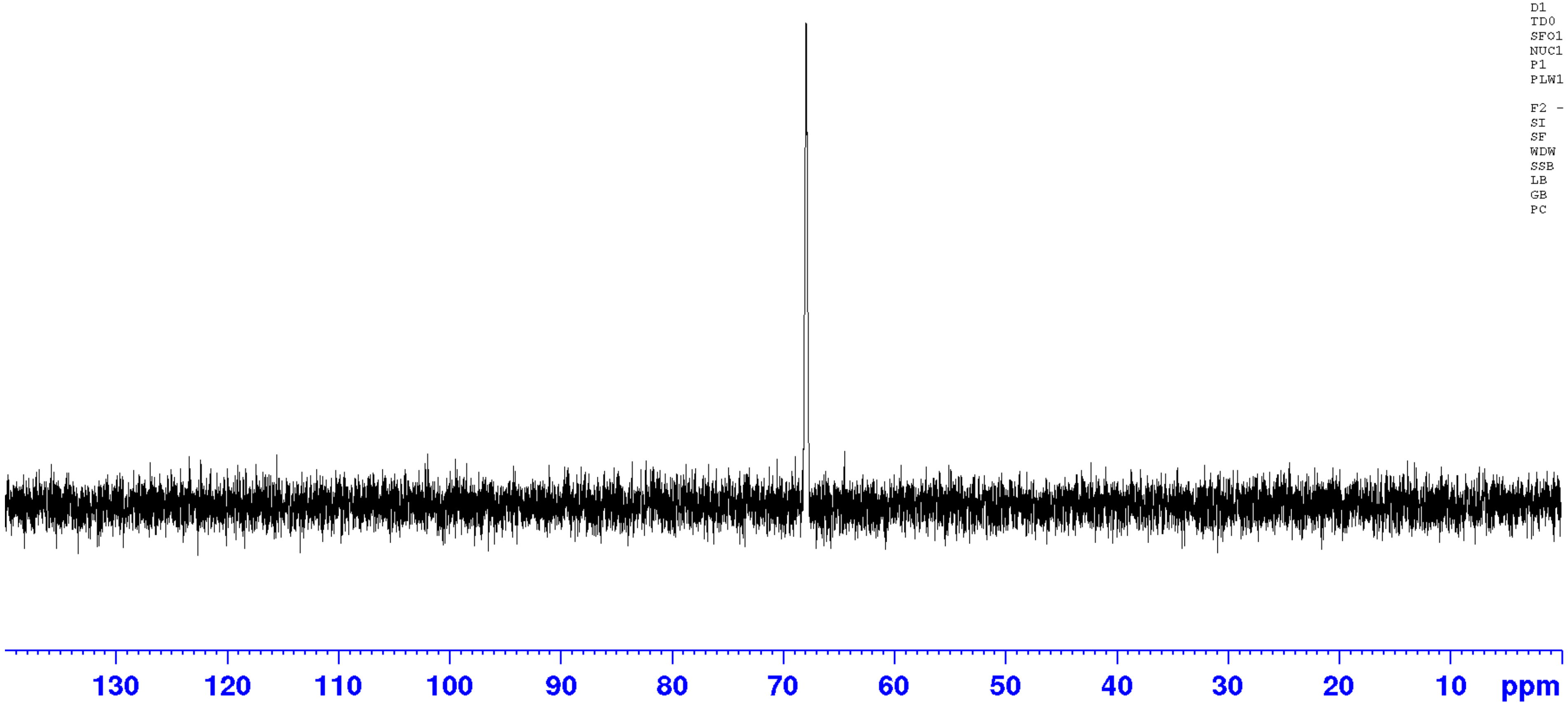


```
Current Data Parameters
NAME       vinn-4-046-2nd-PMe3OBF3-202
EXPNO                     5
PROCNO                    1

F2 - Acquisition Parameters
Date_              20201015
Time                  23.35 h
INSTRUM               spect
PROBHD    Z119470_0283 (
PULPROG                zg30
TD                    65536
SOLVENT               CDCl3
NS                       64
DS                        4
SWH               81521.742 Hz
FIDRES             2.487846 Hz
AQ                0.4019541 sec
RG                   206.72
DW                    6.133 usec
DE                     6.50 usec
TE                    295.1 K
D1               2.00000000 sec
TD0                       1
SFO1            202.4462121 MHz
NUC1                    31P
P1                    14.00 usec
PLW1            54.00000000 W

F2 - Processing parameters
SI                    32768
SF              202.4563350 MHz
WDW                      EM
SSB                       0
LB                     1.00 Hz
GB                        0
PC                     1.40
```

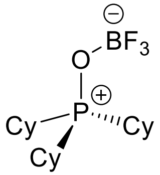

TOB-2

400 MHz ^{1}H NMR

$CDCl_3$

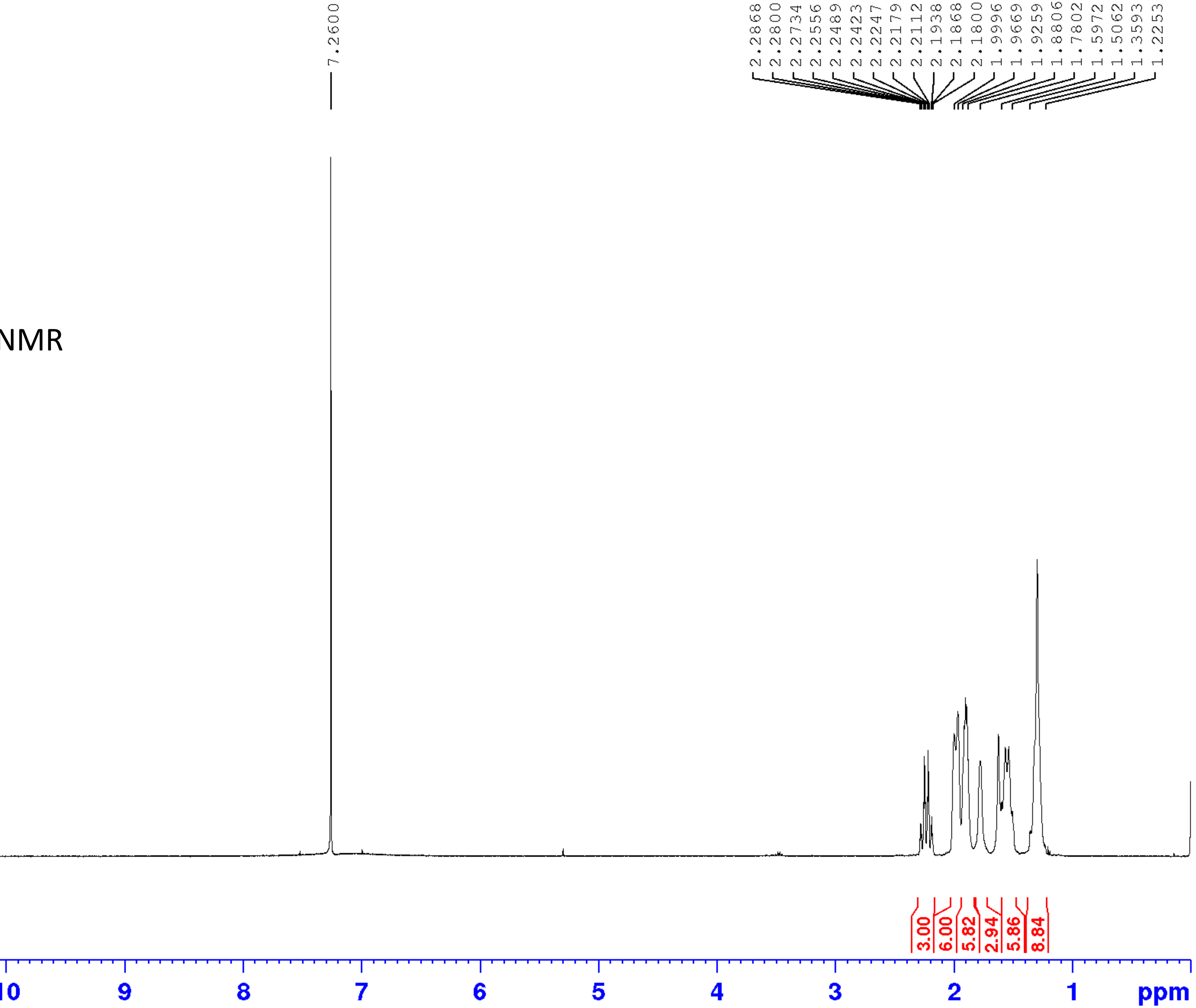


```
Current Data Parameters
NAME      vinn-1-173-PCy3OBF3-20191211
EXPNO                    1
PROCNO                   1

F2 - Acquisition Parameters
Date_             20191211
Time               11.34 h
INSTRUM              spect
PROBHD   Z108618_0257 (
PULPROG               zg30
TD                   65536
SOLVENT              CDCl3
NS                      16
DS                       2
SWH               8012.820 Hz
FIDRES            0.122266 Hz
AQ               4.0894465 sec
RG                     161
DW                  62.400 usec
DE                    6.50 usec
TE                   296.0 K
D1              1.00000000 sec
TD0                      1
SFO1          400.2324714 MHz
NUC1                    1H
P1                   12.80 usec
PLW1           13.56000042 W

F2 - Processing parameters
SI                   65536
SF            400.2300100 MHz
WDW                     EM
SSB                      0
LB                    0.30 Hz
GB                       0
PC                    1.00
```

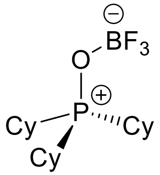

TOB-2

125 MHz ^{13}C NMR

$CDCl_3$

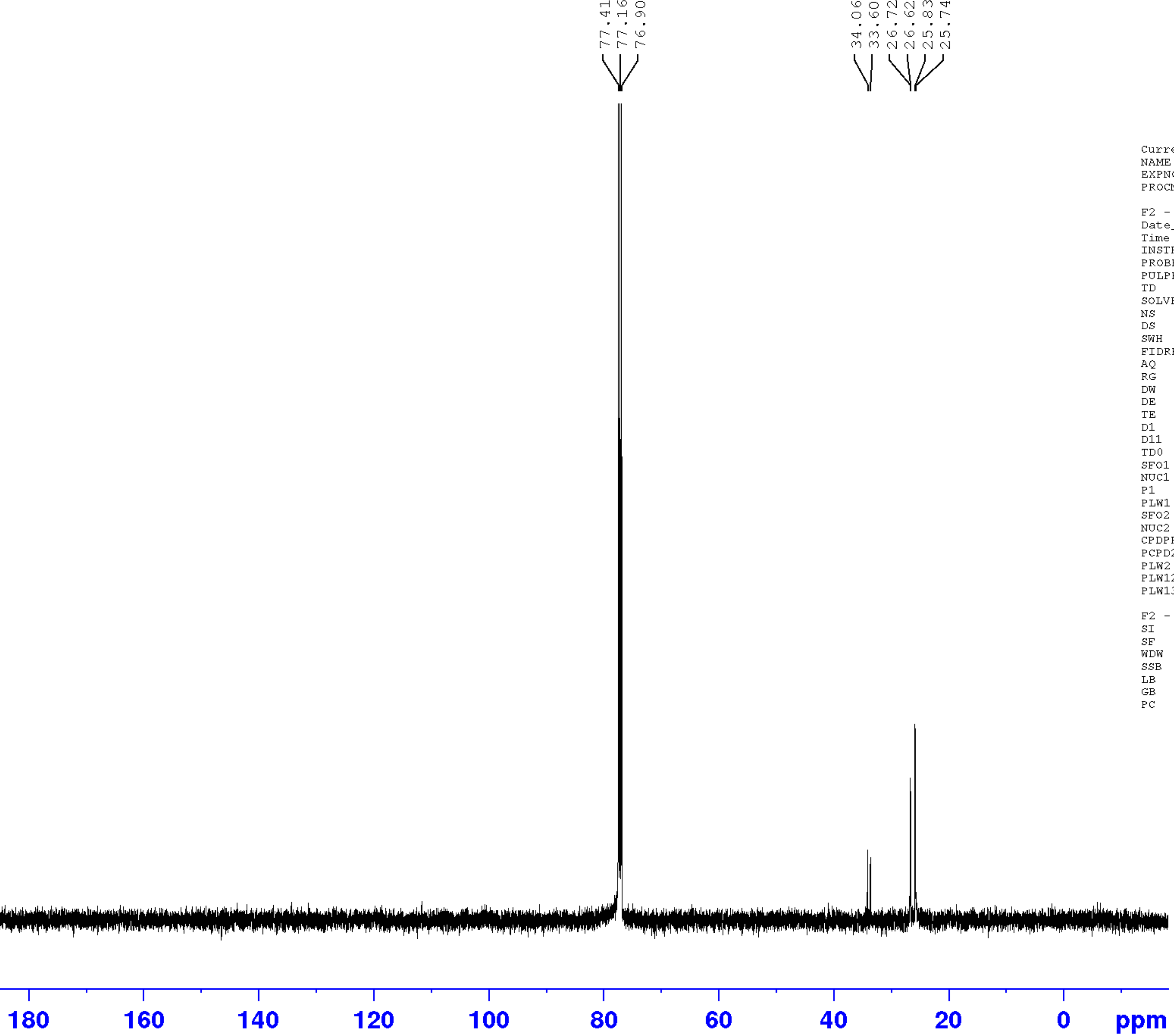


```
Current Data Parameters
NAME      vinn-4-026-PCy3OBF3-2020082
EXPNO                    2
PROCNO                   1

F2 - Acquisition Parameters
Date_             20200823
Time                 18.53 h
INSTRUM              spect
PROBHD   Z119470_0283 (
PULPROG            zgpg30
TD                   65536
SOLVENT              CDCl3
NS                     400
DS                       4
SWH              29761.904 Hz
FIDRES            0.908261 Hz
AQ               1.1010048 sec
RG                  206.72
DW                  16.800 usec
DE                    6.50 usec
TE                   295.2 K
D1              2.00000000 sec
D11             0.03000000 sec
TD0                      1
SFO1           125.7703643 MHz
NUC1                   13C
P1                    9.75 usec
PLW1           94.00000000 W
SFO2           500.1320005 MHz
NUC2                    1H
CPDPRG[2           waltz16
PCPD2                80.00 usec
PLW2           25.00000000 W
PLW12           0.39063001 W
PLW13           0.19648001 W

F2 - Processing parameters
SI                   32768
SF             125.7577735 MHz
WDW                     EM
SSB                      0
LB                    1.00 Hz
GB                       0
PC                    1.40
```

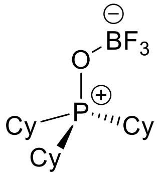

TOB-2

160 MHz ^{11}B NMR

$CDCl_3$

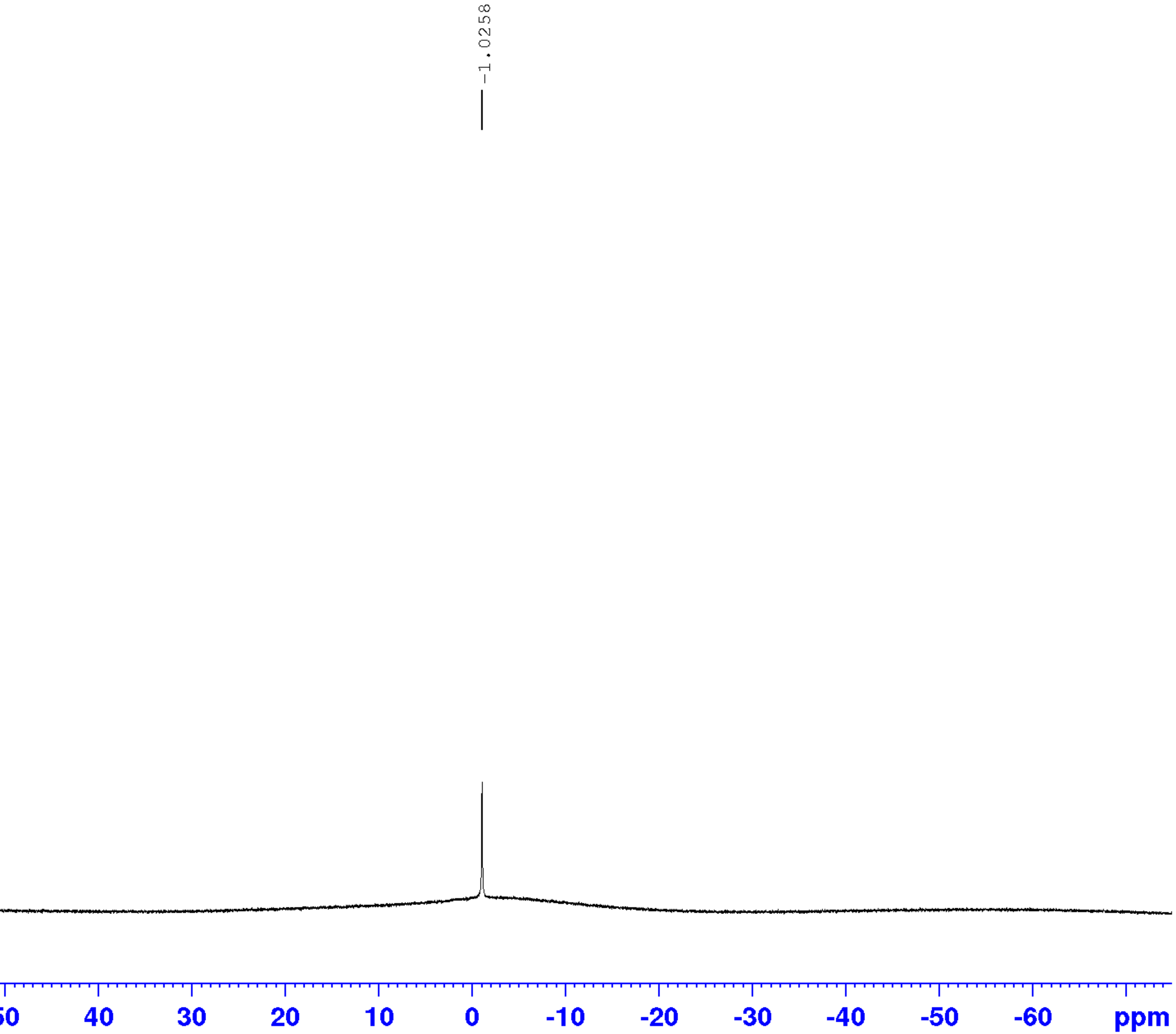


```
Current Data Parameters
NAME      vinn-4-026-PCy3OBF3-20200822
EXPNO                        3
PROCNO                       1

F2 - Acquisition Parameters
Date_             20200823
Time                 18.55 h
INSTRUM              spect
PROBHD   Z119470_0283 (
PULPROG               zgig
TD                   32768
SOLVENT              CDCl3
NS                      16
DS                       4
SWH              24038.461 Hz
FIDRES            1.467191 Hz
AQ               0.6815744 sec
RG                  206.72
DW                  20.800 usec
DE                    6.50 usec
TE                   295.2 K
D1              1.00000000 sec
D11             0.03000000 sec
TD0                      1
SFO1           160.4615790 MHz
NUC1                   11B
P1                   16.00 usec
PLW1           50.00000000 W
SFO2           500.1320005 MHz
NUC2                    1H
CPDPRG[2            waltz16
PCPD2                80.00 usec
PLW2           25.00000000 W
PLW12           0.39063001 W

F2 - Processing parameters
SI                   16384
SF             160.4615792 MHz
WDW                     EM
SSB                      0
LB                    1.00 Hz
GB                       0
PC                    1.40
```

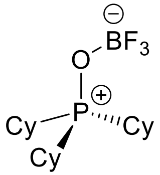

TOB-2

470 MHz ^{19}F NMR

$CDCl_3$

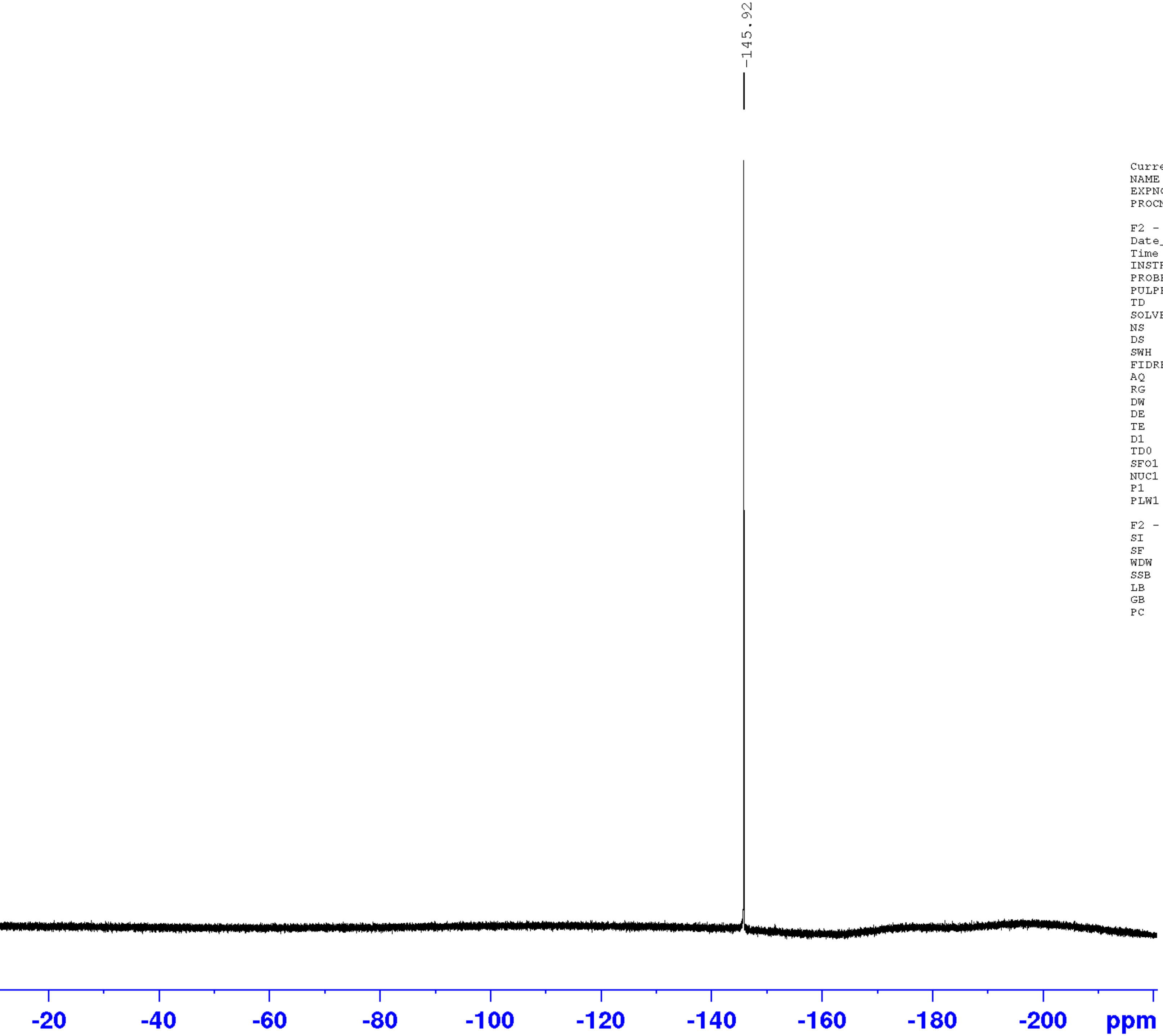


```
Current Data Parameters
NAME      vinn-4-026-PCy3OBF3-2020082
EXPNO                   4
PROCNO                  1

F2 - Acquisition Parameters
Date_            20200823
Time                18.56 h
INSTRUM             spect
PROBHD   Z119470_0283 (
PULPROG            zgflqn
TD                 131072
SOLVENT             CDCl3
NS                     16
DS                      4
SWH           113636.367 Hz
FIDRES          1.733953 Hz
AQ             0.5767168 sec
RG                 206.72
DW                  4.400 usec
DE                   6.50 usec
TE                  295.2 K
D1             1.00000000 sec
TD0                     1
SFO1          470.5453180 MHz
NUC1                  19F
P1                  15.00 usec
PLW1          47.23500061 W

F2 - Processing parameters
SI                  65536
SF            470.5923772 MHz
WDW                    EM
SSB                     0
LB                   0.30 Hz
GB                      0
PC                   1.00
```

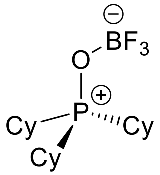

TOB-2

200 MHz ^{31}P NMR

$CDCl_3$

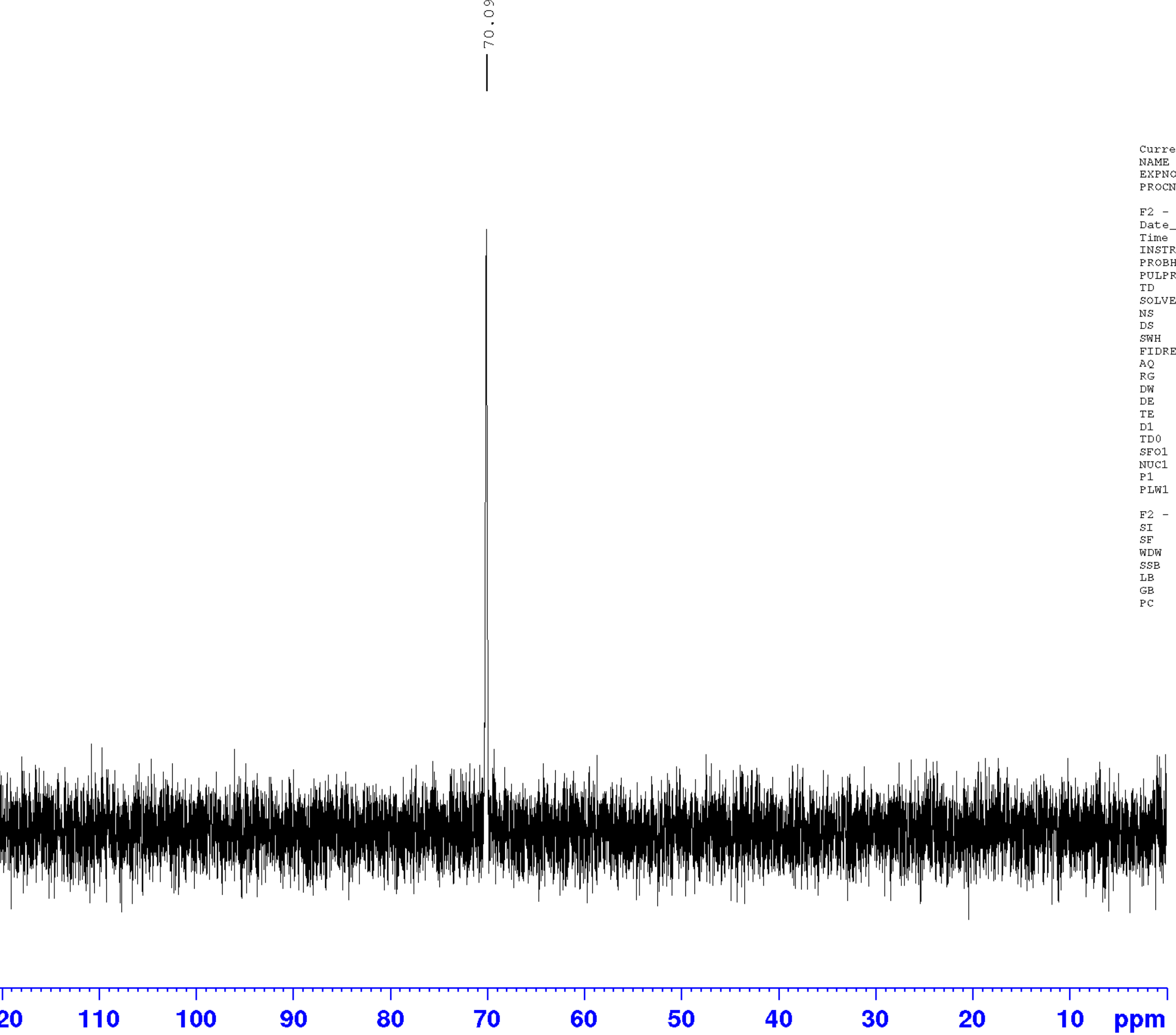


```
Current Data Parameters
NAME       vinn-4-026-PCy3OBF3-2020082
EXPNO                 5
PROCNO                1

F2 - Acquisition Parameters
Date_          20200823
Time              18.58 h
INSTRUM           spect
PROBHD   Z119470_0283 (
PULPROG            zg30
TD                65536
SOLVENT           CDCl3
NS                   32
DS                    4
SWH          81521.742 Hz
FIDRES        2.487846 Hz
AQ           0.4019541 sec
RG               206.72
DW                6.133 usec
DE                 6.50 usec
TE                295.1 K
D1          2.00000000 sec
TD0                   1
SFO1       202.4462121 MHz
NUC1                31P
P1                14.00 usec
PLW1       54.00000000 W

F2 - Processing parameters
SI                32768
SF         202.4563350 MHz
WDW                  EM
SSB                   0
LB                 1.00 Hz
GB                    0
PC                 1.40
```

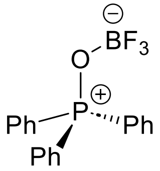

TOB-3

500 MHz ^{1}H NMR

$CDCl_3$

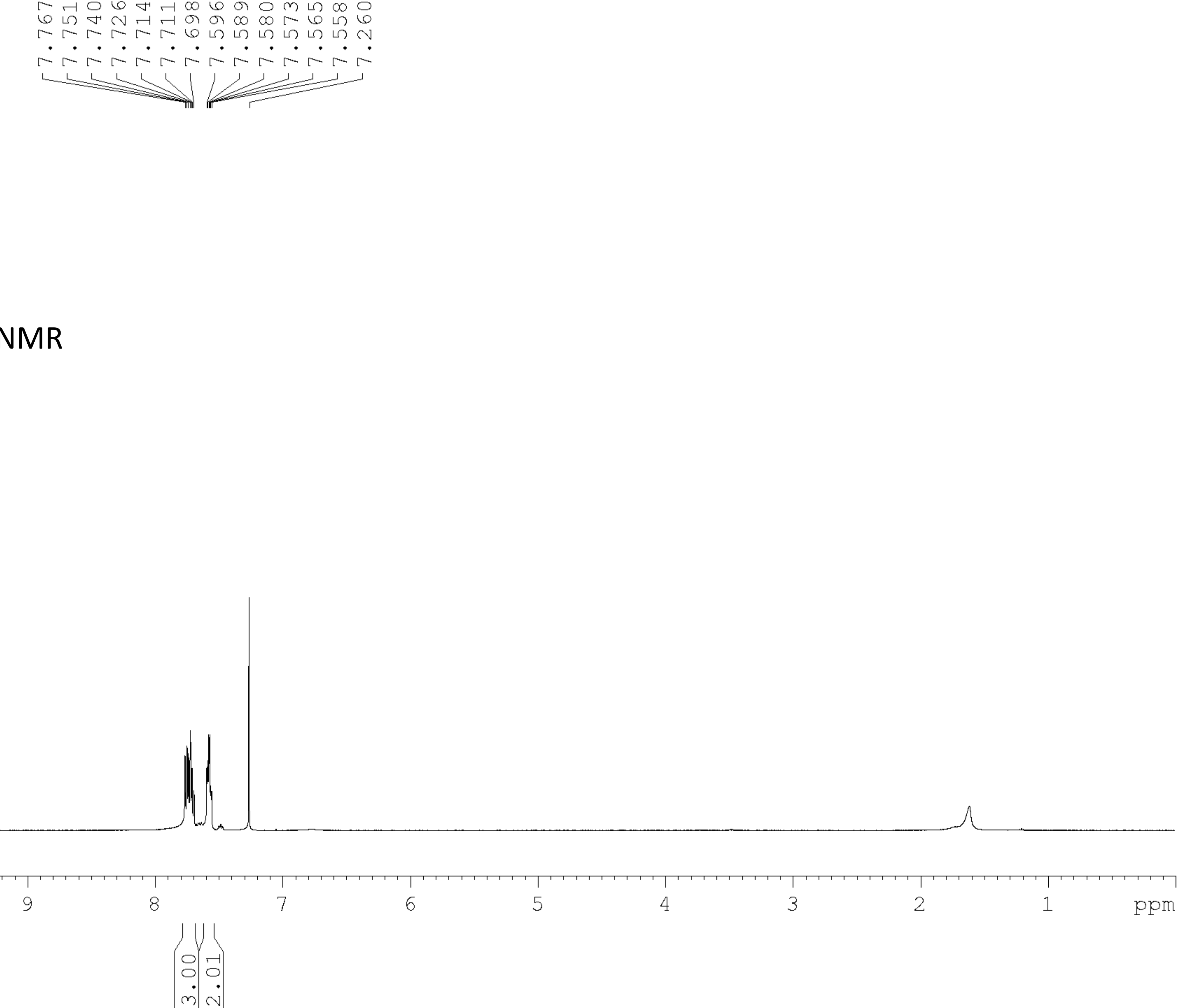


```
Current Data Parameters
NAME      vinn-3-102-PPh3OBF3-CDCl3-2020111
EXPNO              1
PROCNO             1

F2 - Acquisition Parameters
Date_          20201114
Time              12.09 h
INSTRUM           spect
PROBHD   Z119470_0283 (
PULPROG            zg30
TD                65536
SOLVENT           CDCl3
NS                   16
DS                    2
SWH           10000.000 Hz
FIDRES         0.305176 Hz
AQ            3.2767999 sec
RG                142.5
DW               50.000 usec
DE                 6.50 usec
TE                295.2 K
D1           1.00000000 sec
TD0                   1
SFO1        500.1330883 MHz
NUC1                 1H
P1                10.91 usec
PLW1        25.00000000 W

F2 - Processing parameters
SI                65536
SF          500.1300127 MHz
WDW                  EM
SSB                   0
LB                 0.30 Hz
GB                    0
PC                 1.00
```

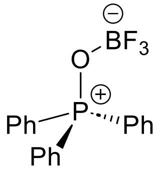

TOB-3

125 MHz ^{13}C NMR

$CDCl_3$

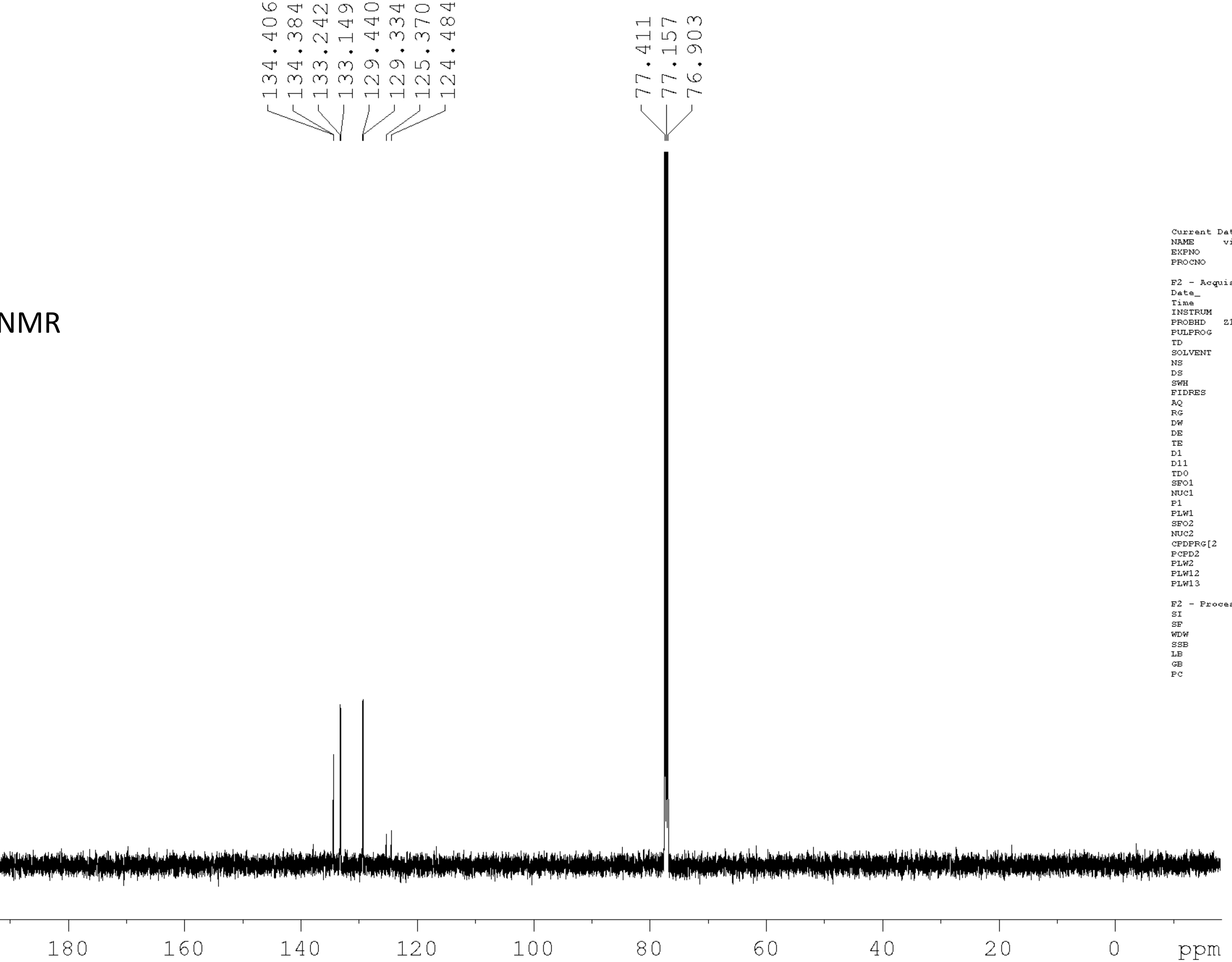


```
Current Data Parameters
NAME       vinn-3-102-PPh3OBF3-CDCl3-20201114
EXPNO                    6
PROCNO                   1

F2 - Acquisition Parameters
Date_             20201114
Time                 12.31 h
INSTRUM              spect
PROBHD   Z119470_0283 (
PULPROG            zgpg30
TD                  65536
SOLVENT             CDCl3
NS                    256
DS                      4
SWH             29761.904 Hz
FIDRES           0.908261 Hz
AQ              1.1010048 sec
RG                 206.72
DW                 16.800 usec
DE                   6.50 usec
TE                  295.1 K
D1             2.00000000 sec
D11            0.03000000 sec
TD0                     1
SFO1          125.7703643 MHz
NUC1                  13C
P1                   9.75 usec
PLW1          94.00000000 W
SFO2          500.1320005 MHz
NUC2                   1H
CPDPRG[2          waltz16
PCPD2               80.00 usec
PLW2          25.00000000 W
PLW12          0.46495000 W
PLW13          0.23387000 W

F2 - Processing parameters
SI                  32768
SF            125.7577729 MHz
WDW                    EM
SSB                     0
LB                   1.00 Hz
GB                      0
PC                   1.40
```

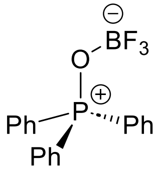

TOB-3

160 MHz ^{11}B NMR

$CDCl_3$

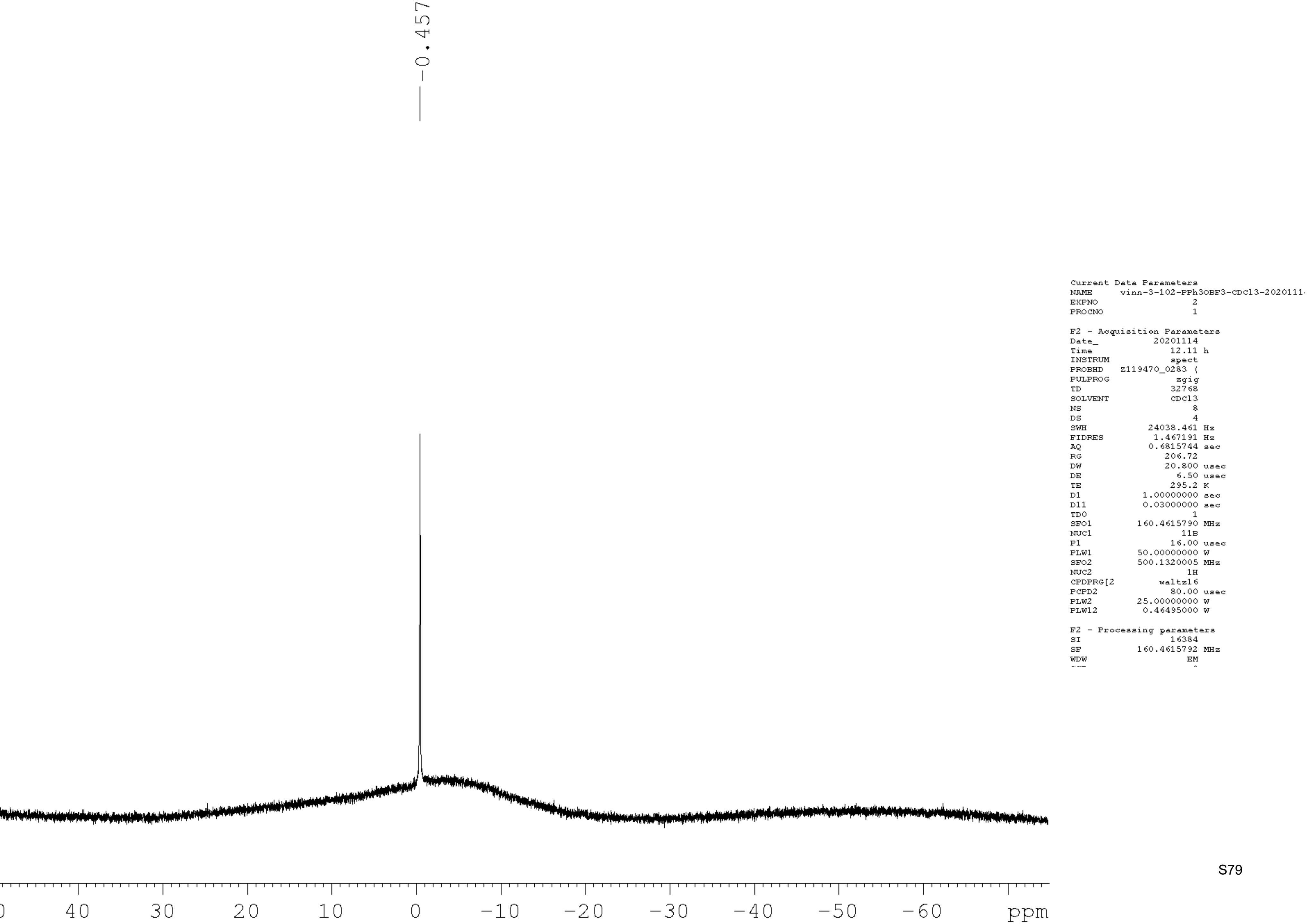

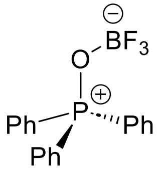

TOB-3

470 MHz ^{19}F NMR

$CDCl_3$

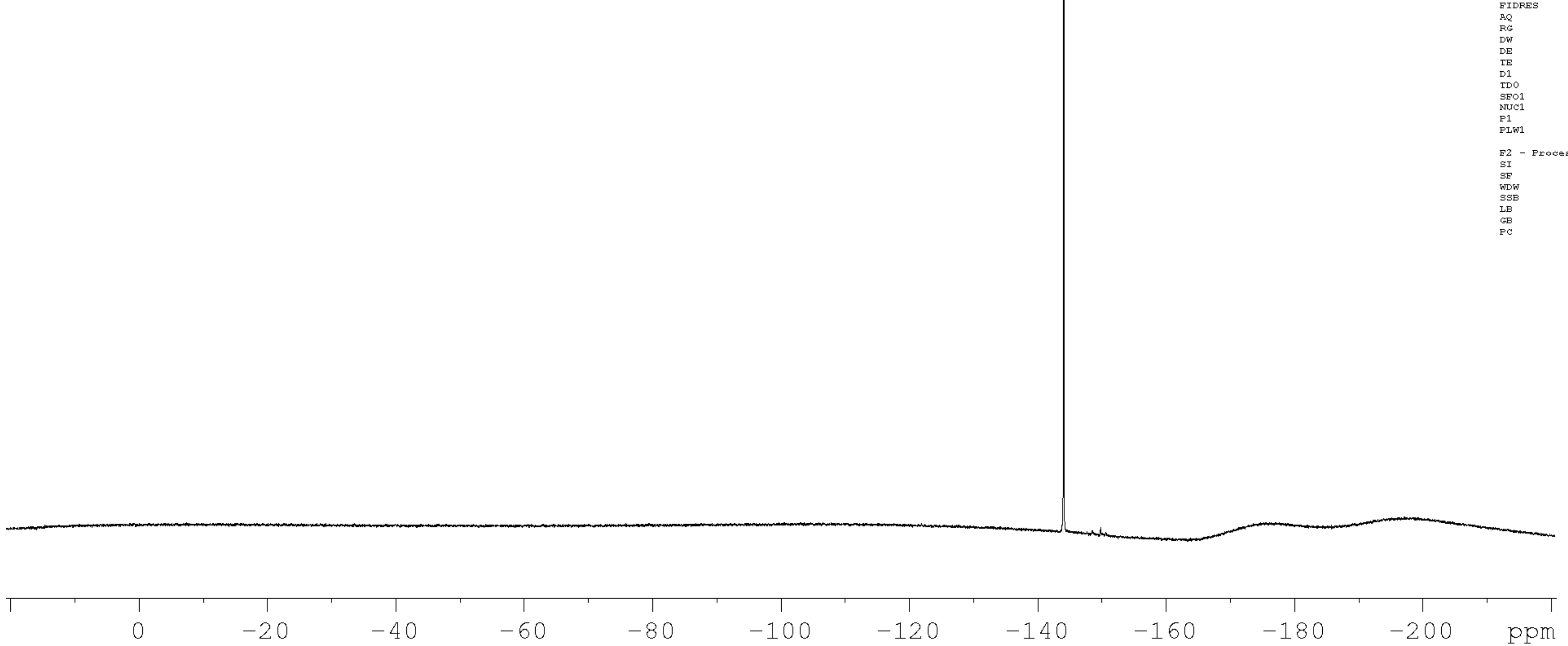


```
Current Data Parameters
NAME     vinn-3-102-PPh3OBF3-CDCl3-20201114
EXPNO                 3
PROCNO                1

F2 - Acquisition Parameters
Date_          20201114
Time              12.12 h
INSTRUM           spect
PROBHD   Z119470_0283 (
PULPROG          zgflqn
TD               131072
SOLVENT           CDCl3
NS                   16
DS                    4
SWH          113636.367 Hz
FIDRES         1.733953 Hz
AQ            0.5767168 sec
RG               206.72
DW                4.400 usec
DE                 6.50 usec
TE                295.2 K
D1           1.00000000 sec
TD0                   1
SFO1        470.5453180 MHz
NUC1                19F
P1                15.00 usec
PLW1        47.23500061 W

F2 - Processing parameters
SI                65536
SF          470.5923772 MHz
WDW                  EM
SSB                   0
LB                10.00 Hz
GB                    0
PC                 1.00
```

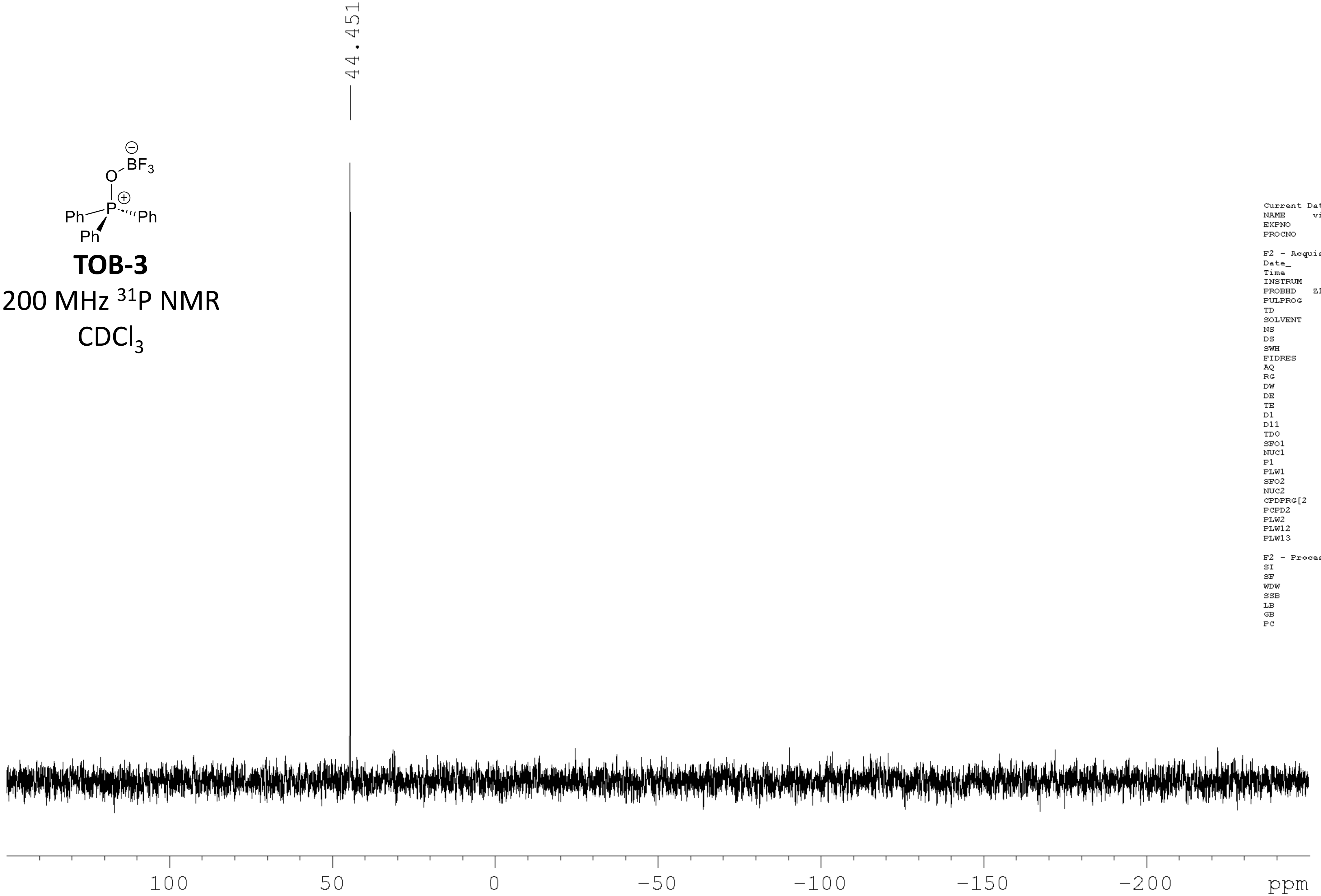

TOB-3

200 MHz ^{31}P NMR

$CDCl_3$

```
Current Data Parameters
NAME      vinn-3-102-PPh3OBF3-CDCl3-20201114
EXPNO                    5
PROCNO                   1

F2 - Acquisition Parameters
Date_             20201114
Time                 12.16 h
INSTRUM              spect
PROBHD   Z119470_0283 (
PULPROG            zgpg30
TD                   65536
SOLVENT              CDCl3
NS                      16
DS                       4
SWH              81521.742 Hz
FIDRES            2.487846 Hz
AQ               0.4019541 sec
RG                  206.72
DW                   6.133 usec
DE                    6.50 usec
TE                   295.2 K
D1              2.00000000 sec
D11             0.03000000 sec
TD0                      1
SFO1           202.4462121 MHz
NUC1                   31P
P1                   14.00 usec
PLW1           54.00000000 W
SFO2           500.1320005 MHz
NUC2                    1H
CPDPRG[2           waltz16
PCPD2                80.00 usec
PLW2           25.00000000 W
PLW12           0.46495000 W
PLW13           0.23387000 W

F2 - Processing parameters
SI                   32768
SF             202.4563350 MHz
WDW                     EM
SSB                      0
LB                   10.00 Hz
GB                       0
PC                    1.40
```

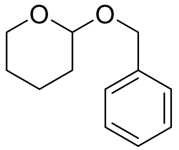

3a

400 MHz ^{1}H NMR

$CDCl_3$

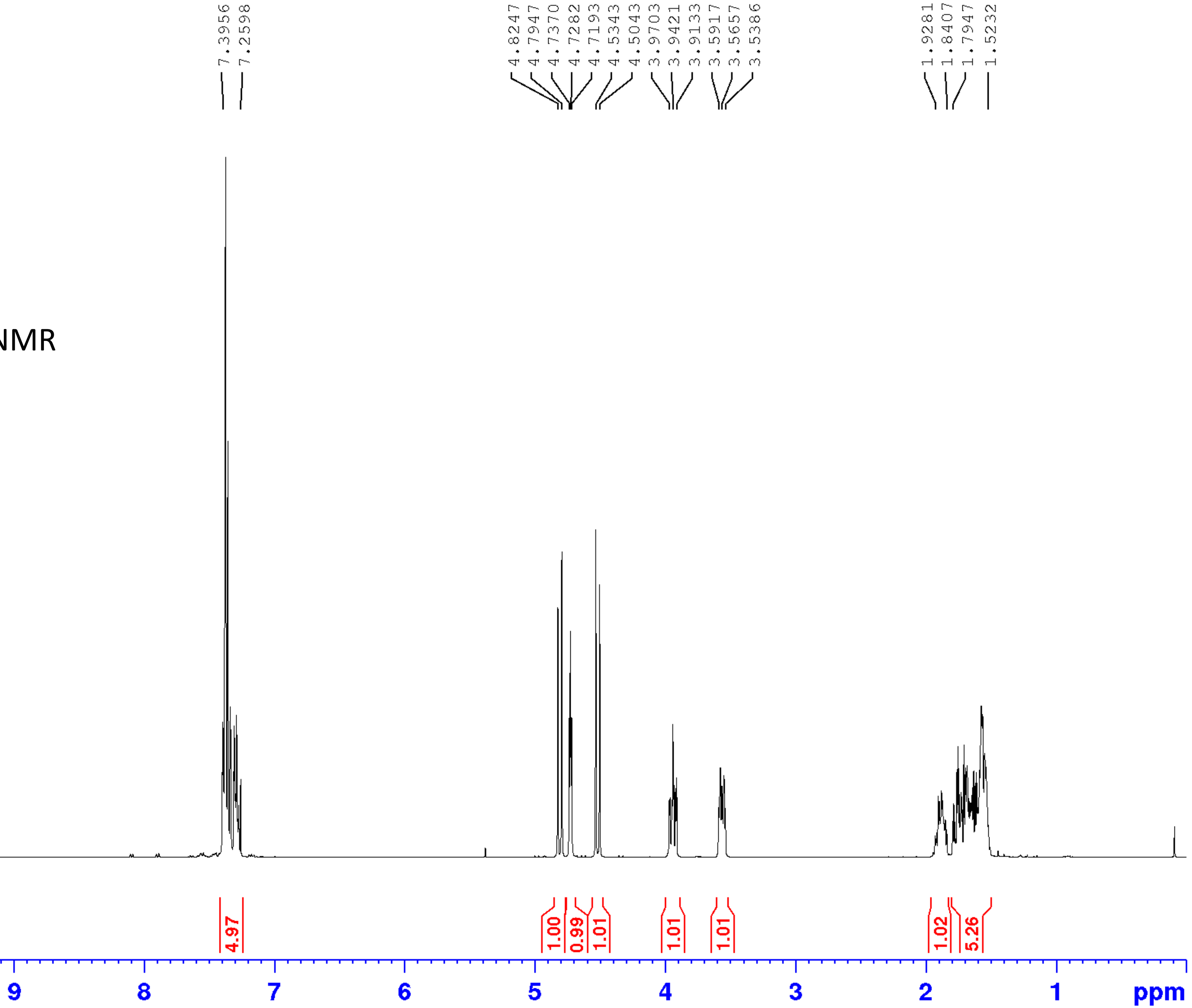


```
Current Data Parameters
NAME     vinn-4-127-islt-20201124
EXPNO                    1
PROCNO                   1

F2 - Acquisition Parameters
Date_              20201124
Time                  12.05 h
INSTRUM               spect
PROBHD    Z820201_0170 (
PULPROG                zg30
TD                    65536
SOLVENT               CDCl3
NS                       16
DS                        2
SWH                8012.820 Hz
FIDRES             0.244532 Hz
AQ                4.0894465 sec
RG                       32
DW                   62.400 usec
DE                     6.50 usec
TE                    295.6 K
D1               1.00000000 sec
TD0                       1
SFO1         400.1324708 MHz
NUC1                     1H
P1                     6.75 usec
PLW1            13.17700005 W

F2 - Processing parameters
SI                    65536
SF           400.1300098 MHz
WDW                      EM
SSB                       0
LB                     0.30 Hz
GB                        0
PC                     1.00
```

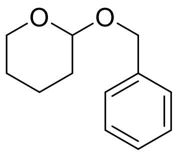

3a

100 MHz ^{13}C NMR

$CDCl_3$

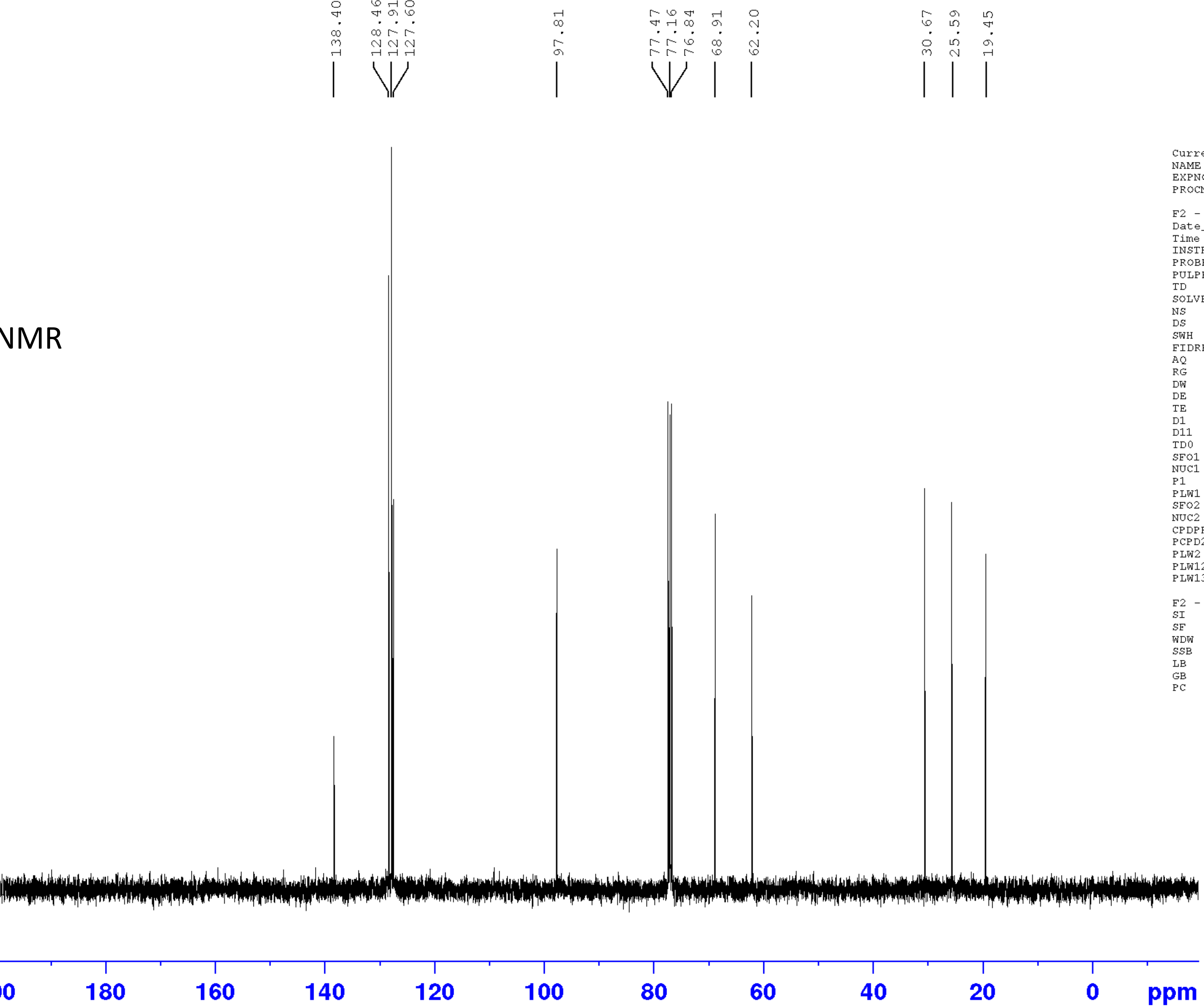


```
Current Data Parameters
NAME      vinn-4-127-islt-20201124
EXPNO                    2
PROCNO                   1

F2 - Acquisition Parameters
Date_             20201124
Time                 12.11 h
INSTRUM              spect
PROBHD    Z820201_0170 (
PULPROG             zgpg30
TD                   65536
SOLVENT              CDCl3
NS                      60
DS                       4
SWH              24038.461 Hz
FIDRES            0.733596 Hz
AQ               1.3631488 sec
RG                     203
DW                  20.800 usec
DE                    6.50 usec
TE                   295.7 K
D1              2.00000000 sec
D11             0.03000000 sec
TD0                      1
SFO1           100.6228298 MHz
NUC1                   13C
P1                   28.00 usec
PLW1           14.80000019 W
SFO2           400.1316005 MHz
NUC2                    1H
CPDPRG[2           waltz16
PCPD2                90.00 usec
PLW2           13.17000008 W
PLW12           0.07408100 W
PLW13           0.03726200 W

F2 - Processing parameters
SI                   32768
SF             100.6127610 MHz
WDW                     EM
SSB                      0
LB                    1.00 Hz
GB                       0
PC                    1.40
```

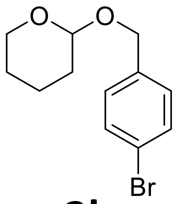

3b

500 MHz ^{1}H NMR

$CDCl_3$

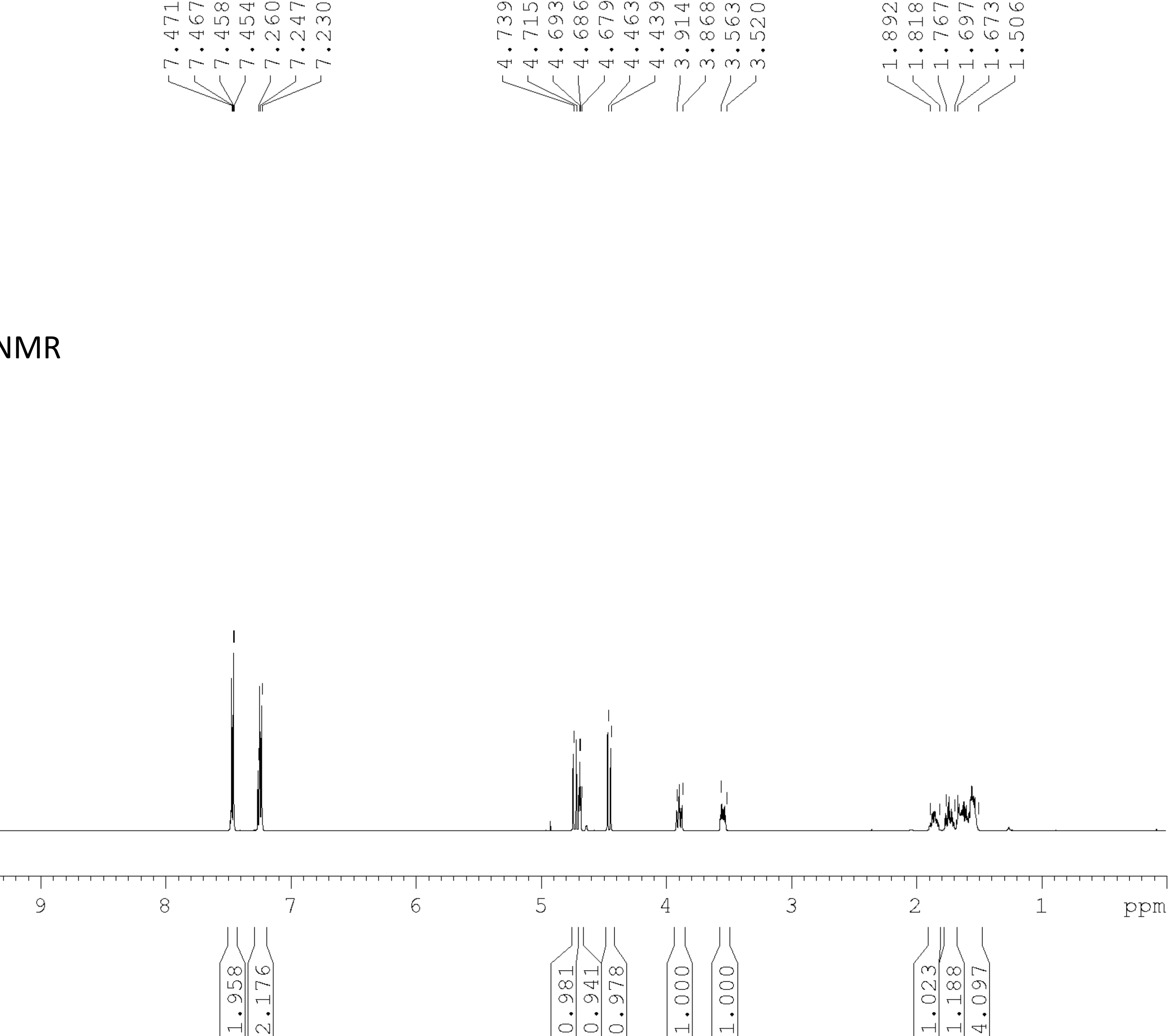


```
Current Data Parameters
NAME        vinn-4-137-1-islt-20201207
EXPNO                    1
PROCNO                   1

F2 - Acquisition Parameters
Date_             20201207
Time                18.39 h
INSTRUM              spect
PROBHD   Z119470_0283 (
PULPROG               zg30
TD                   65536
SOLVENT              CDCl3
NS                      16
DS                       2
SWH              10000.000 Hz
FIDRES            0.305176 Hz
AQ               3.2767999 sec
RG                   30.85
DW                  50.000 usec
DE                    6.50 usec
TE                   295.1 K
D1              1.00000000 sec
TD0                      1
SFO1           500.1330883 MHz
NUC1                    1H
P1                   10.91 usec
PLW1           25.00000000 W

F2 - Processing parameters
SI                   65536
SF             500.1300122 MHz
WDW                     EM
SSB                      0
LB                    0.30 Hz
GB                       0
PC                    1.00
```

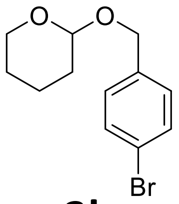

3b

125 MHz ^{13}C NMR

$CDCl_3$

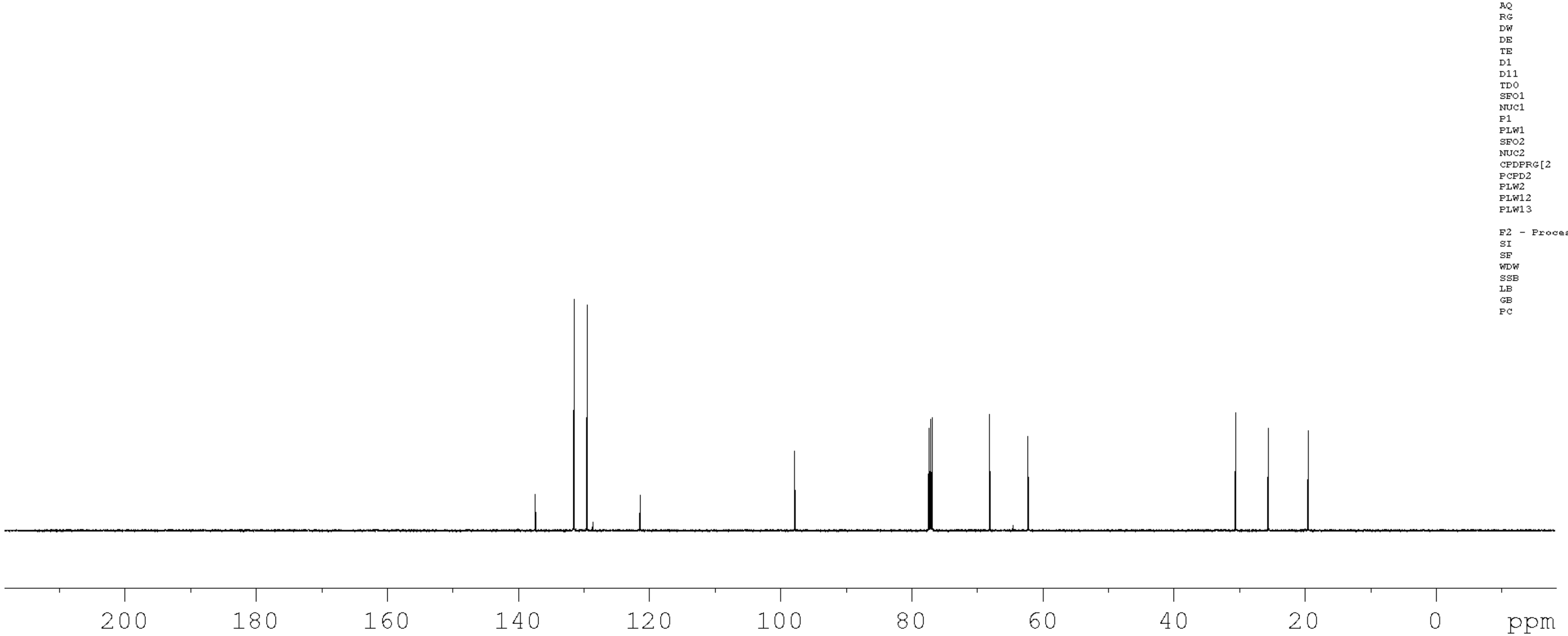


```
Current Data Parameters
NAME     vinn-4-137-1-islt-20201207
EXPNO                    2
PROCNO                   1

F2 - Acquisition Parameters
Date_             20201207
Time              18.45 h
INSTRUM           spect
PROBHD   Z119470_0283 (
PULPROG           zgpg30
TD                65536
SOLVENT           CDCl3
NS                100
DS                4
SWH               29761.904 Hz
FIDRES            0.908261 Hz
AQ                1.1010048 sec
RG                206.72
DW                16.800 usec
DE                6.50 usec
TE                295.1 K
D1                2.00000000 sec
D11               0.03000000 sec
TD0               1
SFO1              125.7703643 MHz
NUC1              13C
P1                9.75 usec
PLW1              94.00000000 W
SFO2              500.1320005 MHz
NUC2              1H
CPDPRG[2          waltz16
PCPD2             80.00 usec
PLW2              25.00000000 W
PLW12             0.46495000 W
PLW13             0.23387000 W

F2 - Processing parameters
SI                32768
SF                125.7577782 MHz
WDW               EM
SSB               0
LB                1.00 Hz
GB                0
PC                1.40
```

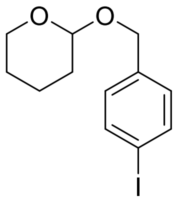

3c

500 MHz ^{1}H NMR

$CDCl_3$

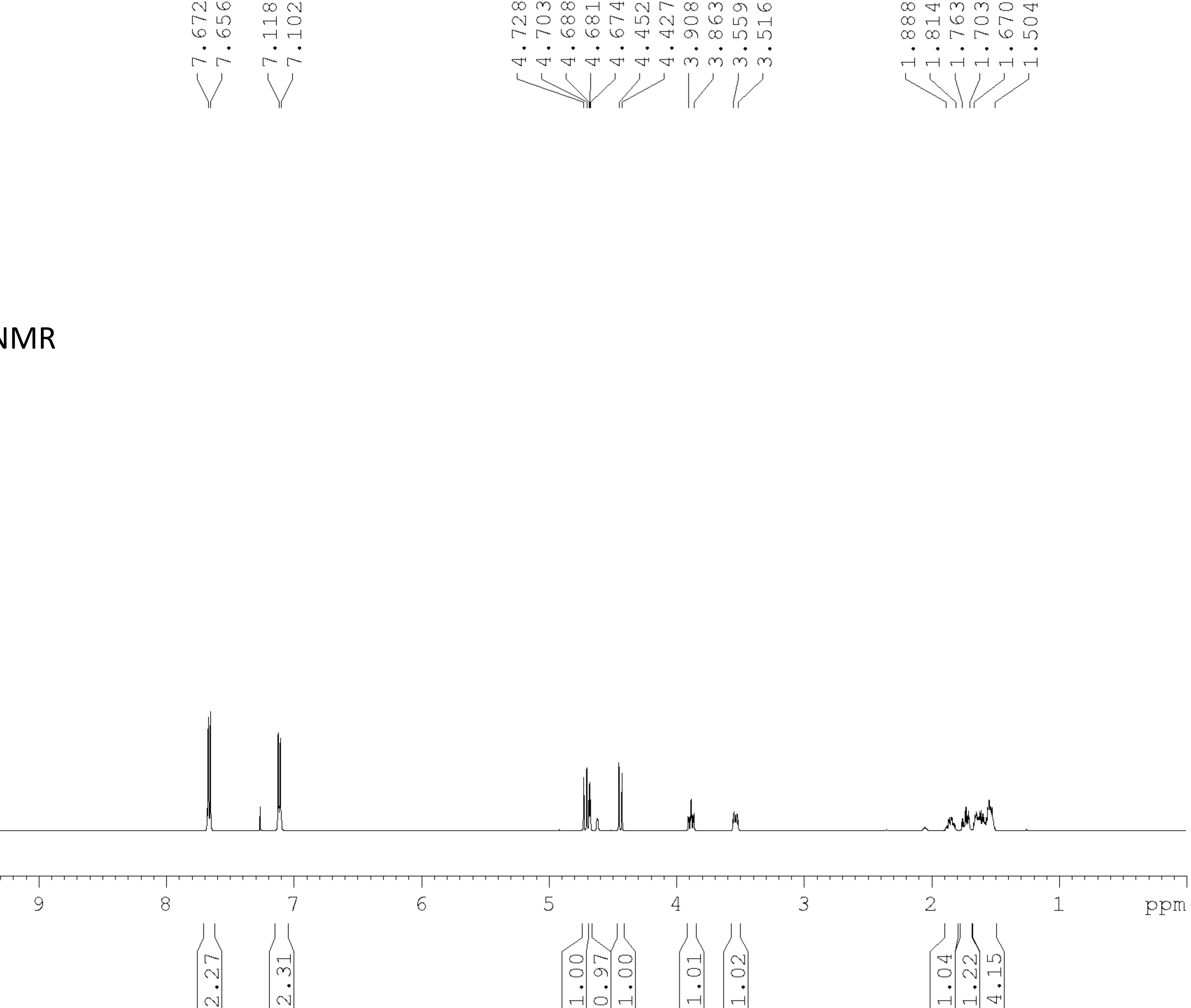


```
Current Data Parameters
NAME      vinn-4-137-2-islt-20201207
EXPNO              1
PROCNO             1

F2 - Acquisition Parameters
Date_          20201207
Time              18.49 h
INSTRUM           spect
PROBHD   Z119470_0283 (
PULPROG            zg30
TD                65536
SOLVENT           CDCl3
NS                   16
DS                    2
SWH           10000.000 Hz
FIDRES         0.305176 Hz
AQ            3.2767999 sec
RG                30.85
DW               50.000 usec
DE                 6.50 usec
TE                295.1 K
D1           1.00000000 sec
TD0                   1
SFO1        500.1330883 MHz
NUC1                 1H
P1                10.91 usec
PLW1        25.00000000 W

F2 - Processing parameters
SI                65536
SF          500.1300122 MHz
WDW                  EM
SSB                   0
LB                 0.30 Hz
GB                    0
PC                 1.00
```

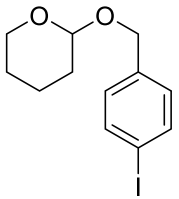

3c

125 MHz ^{13}C NMR

$CDCl_3$

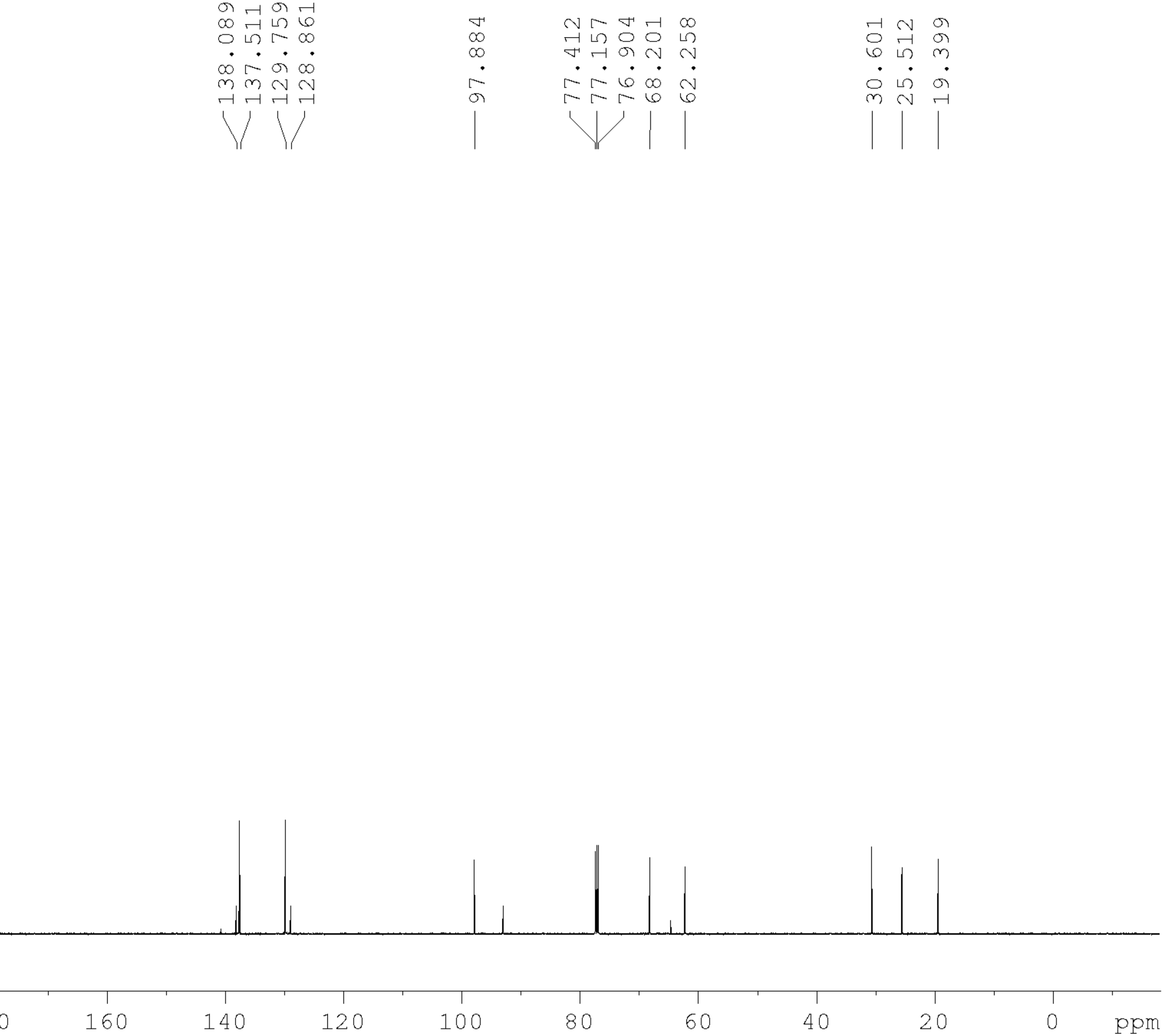


```
Current Data Parameters
NAME     vinn-4-137-2-islt-20201207
EXPNO                  2
PROCNO                 1

F2 - Acquisition Parameters
Date_           20201207
Time               18.55 h
INSTRUM            spect
PROBHD   Z119470_0283 (
PULPROG           zgpg30
TD                 65536
SOLVENT            CDCl3
NS                   100
DS                     4
SWH            29761.904 Hz
FIDRES          0.908261 Hz
AQ             1.1010048 sec
RG                206.72
DW                16.800 usec
DE                  6.50 usec
TE                 295.2 K
D1            2.00000000 sec
D11           0.03000000 sec
TD0                    1
SFO1         125.7703643 MHz
NUC1                 13C
P1                  9.75 usec
PLW1         94.00000000 W
SFO2         500.1320005 MHz
NUC2                  1H
CPDPRG[2         waltz16
PCPD2              80.00 usec
PLW2         25.00000000 W
PLW12         0.46495000 W
PLW13         0.23387000 W

F2 - Processing parameters
SI                 32768
SF           125.7577798 MHz
```

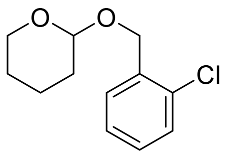

3d

500 MHz ^{1}H NMR

$CDCl_3$

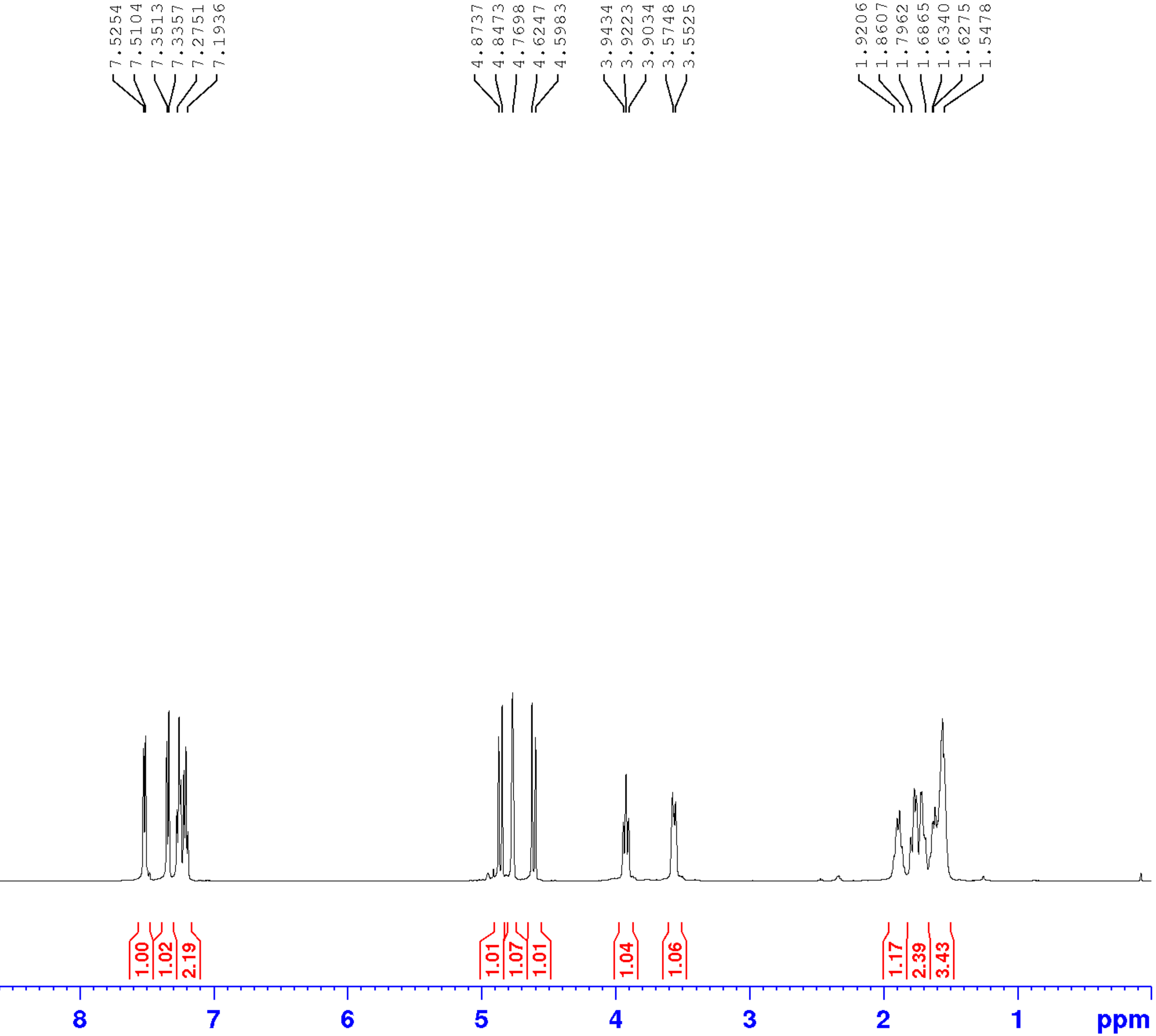


```
Current Data Parameters
NAME      vinn-4-137-16-islt-2020121
EXPNO                    1
PROCNO                   1

F2 - Acquisition Parameters
Date_             20201211
Time                  6.34 h
INSTRUM              spect
PROBHD   Z119470_0283 (
PULPROG               zg30
TD                   65536
SOLVENT              CDCl3
NS                      16
DS                       2
SWH              10000.000 Hz
FIDRES            0.305176 Hz
AQ               3.2767999 sec
RG                   30.85
DW                  50.000 usec
DE                    6.50 usec
TE                   295.1 K
D1              1.00000000 sec
TD0                      1
SFO1           500.1330883 MHz
NUC1                    1H
P1                   10.91 usec
PLW1           25.00000000 W

F2 - Processing parameters
SI                   65536
SF             500.1300150 MHz
WDW                     EM
SSB                      0
LB                    0.30 Hz
GB                       0
PC                    1.00
```

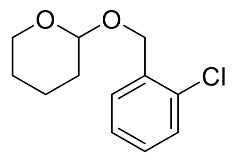

3d

125 MHz ^{13}C NMR

$CDCl_3$

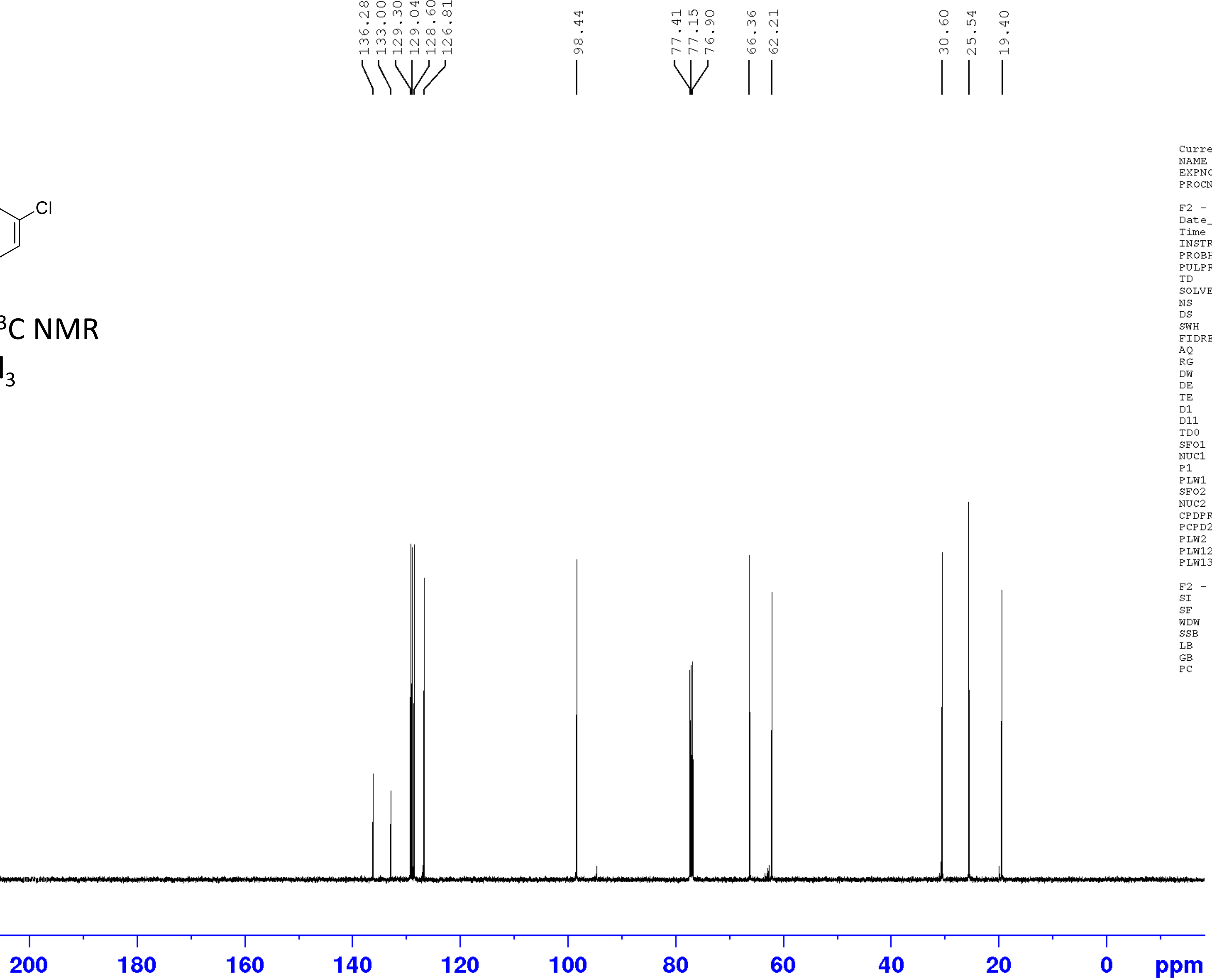


```
Current Data Parameters
NAME      vinn-4-137-16-islt-20201210
EXPNO                    2
PROCNO                   1

F2 - Acquisition Parameters
Date_             20201211
Time                6.39 h
INSTRUM            spect
PROBHD   Z119470_0283 (
PULPROG           zgpg30
TD                 65536
SOLVENT            CDCl3
NS                   100
DS                     4
SWH            29761.904 Hz
FIDRES          0.908261 Hz
AQ             1.1010048 sec
RG                206.72
DW                16.800 usec
DE                  6.50 usec
TE                 295.1 K
D1            2.00000000 sec
D11           0.03000000 sec
TD0                    1
SFO1         125.7703643 MHz
NUC1                 13C
P1                  9.75 usec
PLW1         94.00000000 W
SFO2         500.1320005 MHz
NUC2                  1H
CPDPRG[2         waltz16
PCPD2              80.00 usec
PLW2         25.00000000 W
PLW12         0.46495000 W
PLW13         0.23387000 W

F2 - Processing parameters
SI                 32768
SF           125.7577804 MHz
WDW                   EM
SSB                    0
LB                  1.00 Hz
GB                     0
PC                  1.40
```

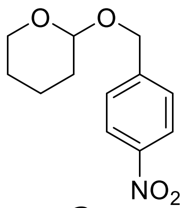

3e

500 MHz ^{1}H NMR

$CDCl_3$

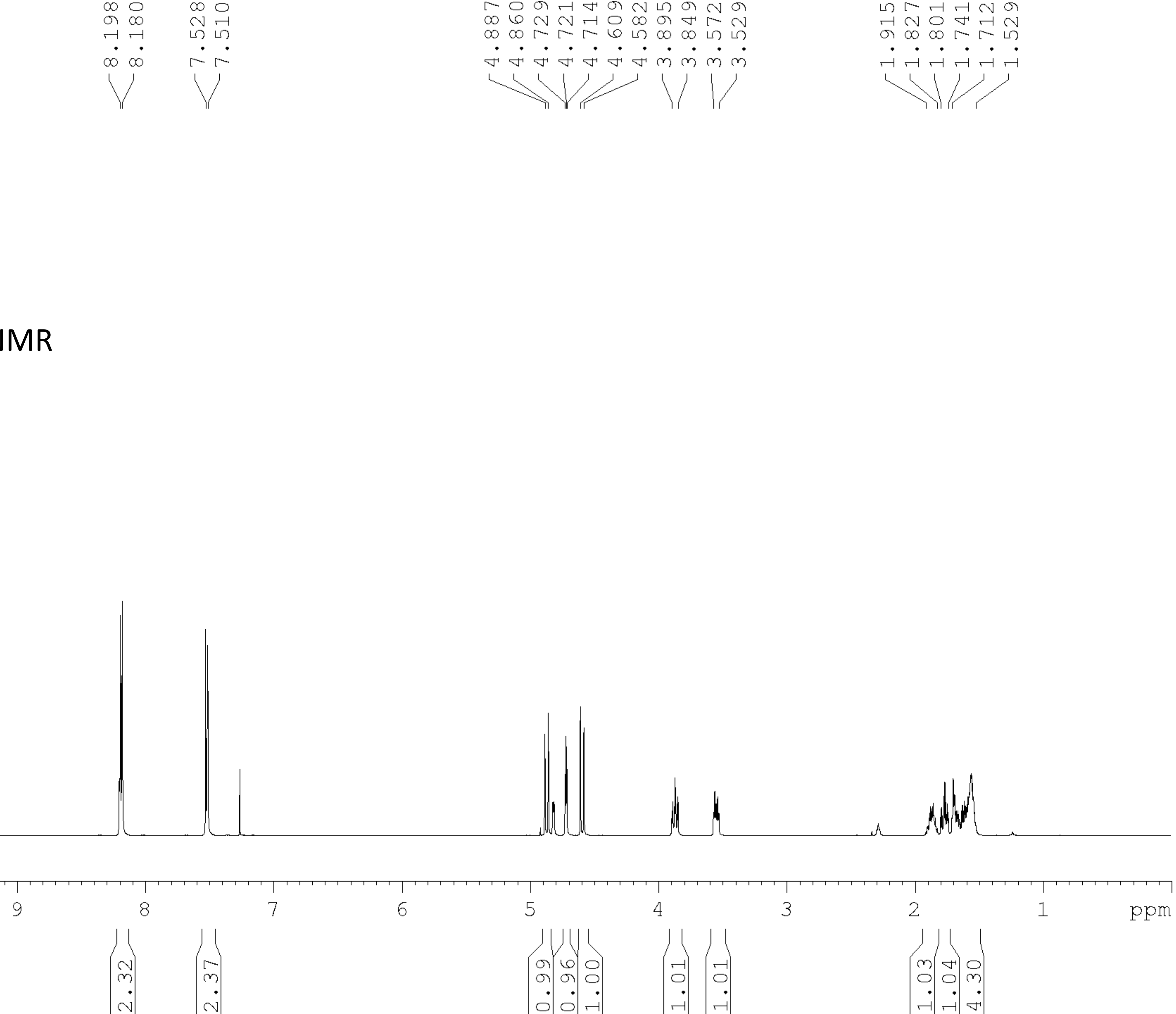


```
Current Data Parameters
NAME     vinn-4-137-4-islt-20201207
EXPNO                 1
PROCNO                1

F2 - Acquisition Parameters
Date_          20201207
Time              19.08 h
INSTRUM           spect
PROBHD   Z119470_0283 (
PULPROG            zg30
TD                65536
SOLVENT           CDCl3
NS                   16
DS                    2
SWH           10000.000 Hz
FIDRES         0.305176 Hz
AQ            3.2767999 sec
RG                 50.6
DW               50.000 usec
DE                 6.50 usec
TE                295.2 K
D1           1.00000000 sec
TD0                   1
SFO1        500.1330883 MHz
NUC1                 1H
P1                10.91 usec
PLW1        25.00000000 W

F2 - Processing parameters
SI                65536
SF          500.1300122 MHz
WDW                  EM
SSB                   0
LB                 0.30 Hz
GB                    0
PC                 1.00
```

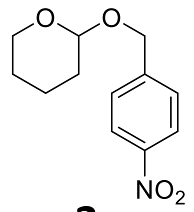

3e

125 MHz ^{13}C NMR

$CDCl_3$

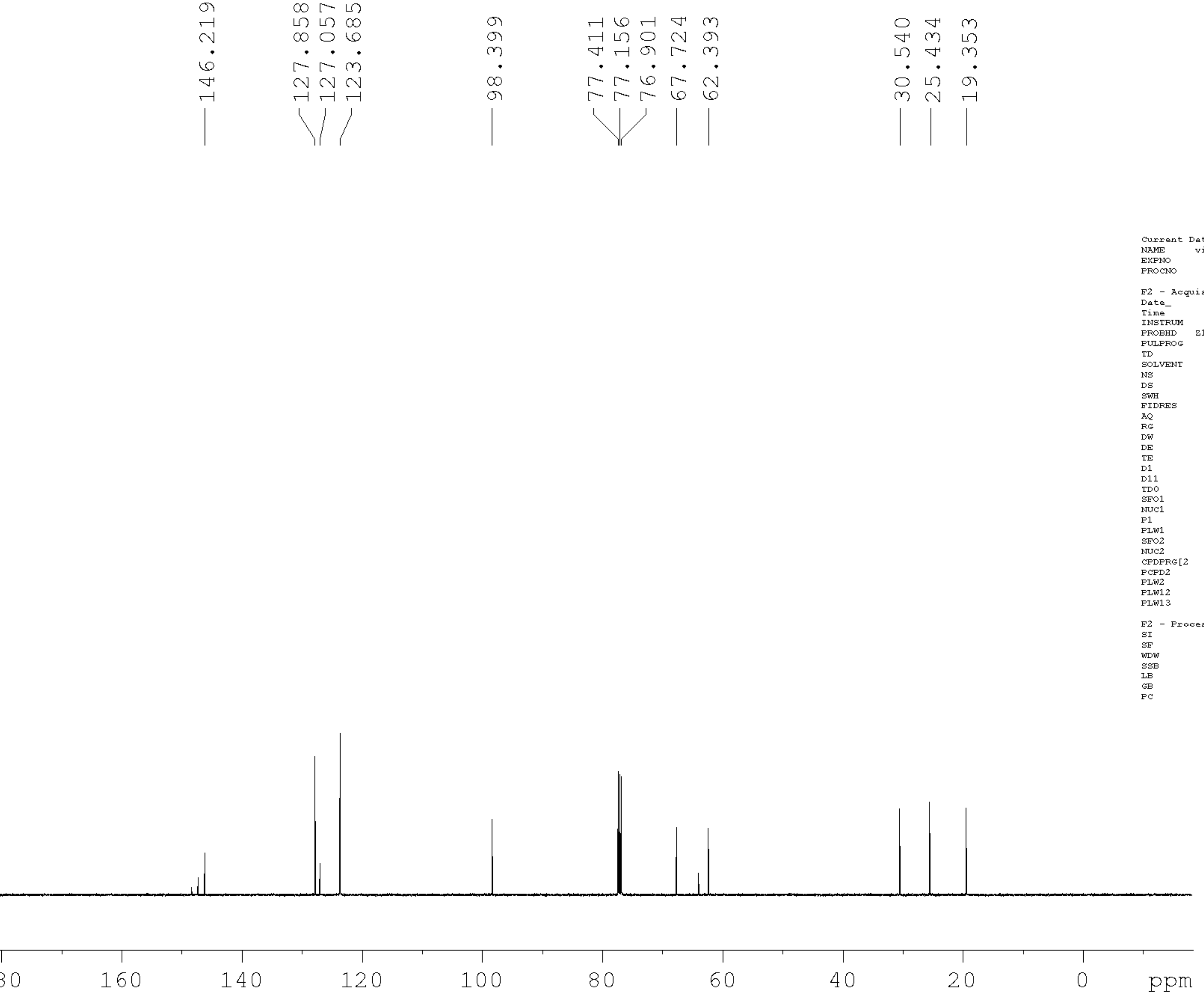

Current Data Parameters

NAME	vinn-4-137-4-islt-20201207
EXPNO	2
PROCNO	1

F2 - Acquisition Parameters

Date_	20201207
Time	19.14 h
INSTRUM	spect
PROBHD	Z119470_0283 (
PULPROG	zgpg30
TD	65536
SOLVENT	CDCl3
NS	100
DS	4
SWH	29761.904 Hz
FIDRES	0.908261 Hz
AQ	1.1010048 sec
RG	206.72
DW	16.800 usec
DE	6.50 usec
TE	295.1 K
D1	2.00000000 sec
D11	0.03000000 sec
TD0	1
SFO1	125.7703643 MHz
NUC1	13C
P1	9.75 usec
PLW1	94.00000000 W
SFO2	500.1320005 MHz
NUC2	1H
CPDPRG[2	waltz16
PCPD2	80.00 usec
PLW2	25.00000000 W
PLW12	0.46495000 W
PLW13	0.23387000 W

F2 - Processing parameters

SI	32768
SF	125.7577777 MHz
WDW	EM
SSB	0
LB	1.00 Hz
GB	0
PC	1.40

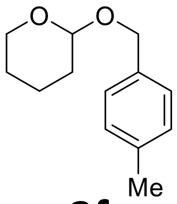

3f

500 MHz ^{1}H NMR

$CDCl_3$

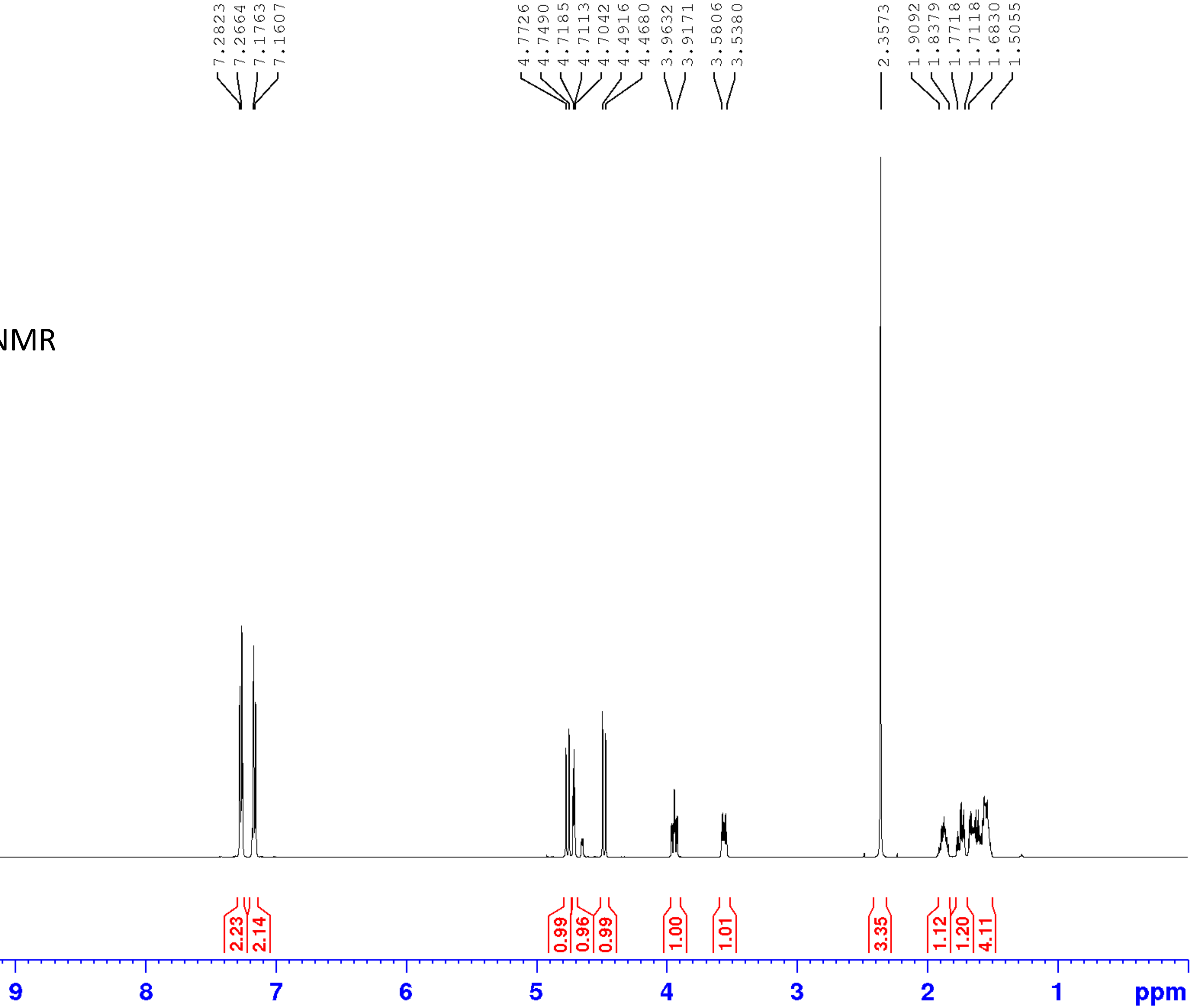

Current Data Parameters
NAME vinn-4-137-3-islt-20201207
EXPNO 1
PROCNO 1

F2 - Acquisition Parameters
Date_ 20201207
Time 18.58 h
INSTRUM spect
PROBHD Z119470_0283 (
PULPROG zg30
TD 65536
SOLVENT CDCl3
NS 16
DS 2
SWH 10000.000 Hz
FIDRES 0.305176 Hz
AQ 3.2767999 sec
RG 30.85
DW 50.000 usec
DE 6.50 usec
TE 295.1 K
D1 1.00000000 sec
TD0 1
SFO1 500.1330883 MHz
NUC1 1H
P1 10.91 usec
PLW1 25.00000000 W

F2 - Processing parameters
SI 65536
SF 500.1300121 MHz
WDW EM
SSB 0
LB 0.30 Hz
GB 0
PC 1.00

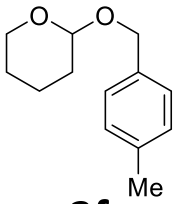

3f

125 MHz ^{13}C NMR

$CDCl_3$

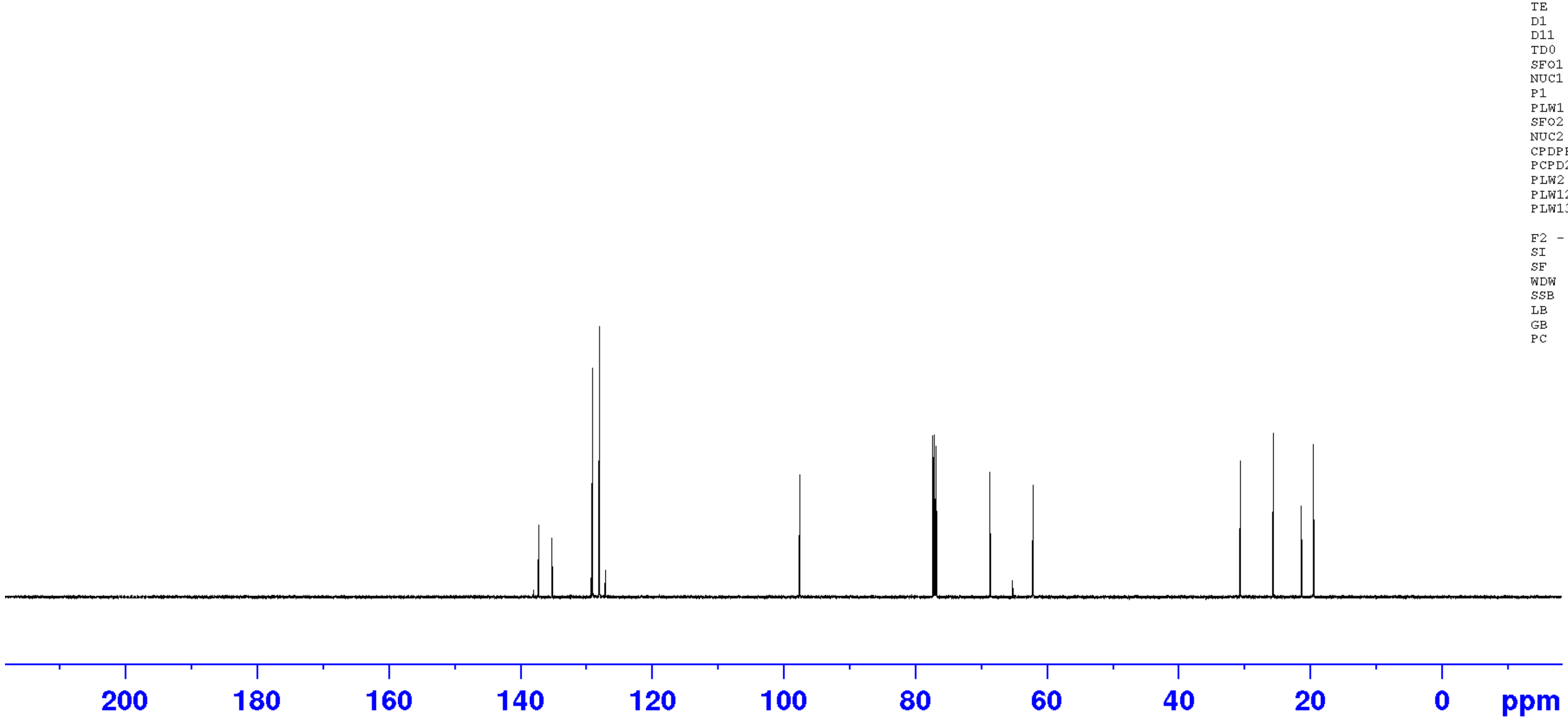


```
Current Data Parameters
NAME      vinn-4-137-3-islt-20201207
EXPNO                    2
PROCNO                   1

F2 - Acquisition Parameters
Date_             20201207
Time                 19.04 h
INSTRUM              spect
PROBHD    Z119470_0283 (
PULPROG             zgpg30
TD                   65536
SOLVENT              CDCl3
NS                     100
DS                       4
SWH              29761.904 Hz
FIDRES            0.908261 Hz
AQ               1.1010048 sec
RG                  206.72
DW                  16.800 usec
DE                    6.50 usec
TE                   295.2 K
D1              2.00000000 sec
D11             0.03000000 sec
TD0                      1
SFO1           125.7703643 MHz
NUC1                   13C
P1                    9.75 usec
PLW1           94.00000000 W
SFO2           500.1320005 MHz
NUC2                    1H
CPDPRG[2           waltz16
PCPD2                80.00 usec
PLW2           25.00000000 W
PLW12           0.46495000 W
PLW13           0.23387000 W

F2 - Processing parameters
SI                   32768
SF             125.7577796 MHz
WDW                     EM
SSB                      0
LB                    1.00 Hz
GB                       0
PC                    1.40
```

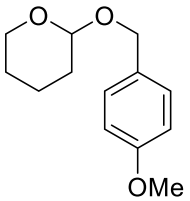

3g

500 MHz ^{1}H NMR

$CDCl_3$

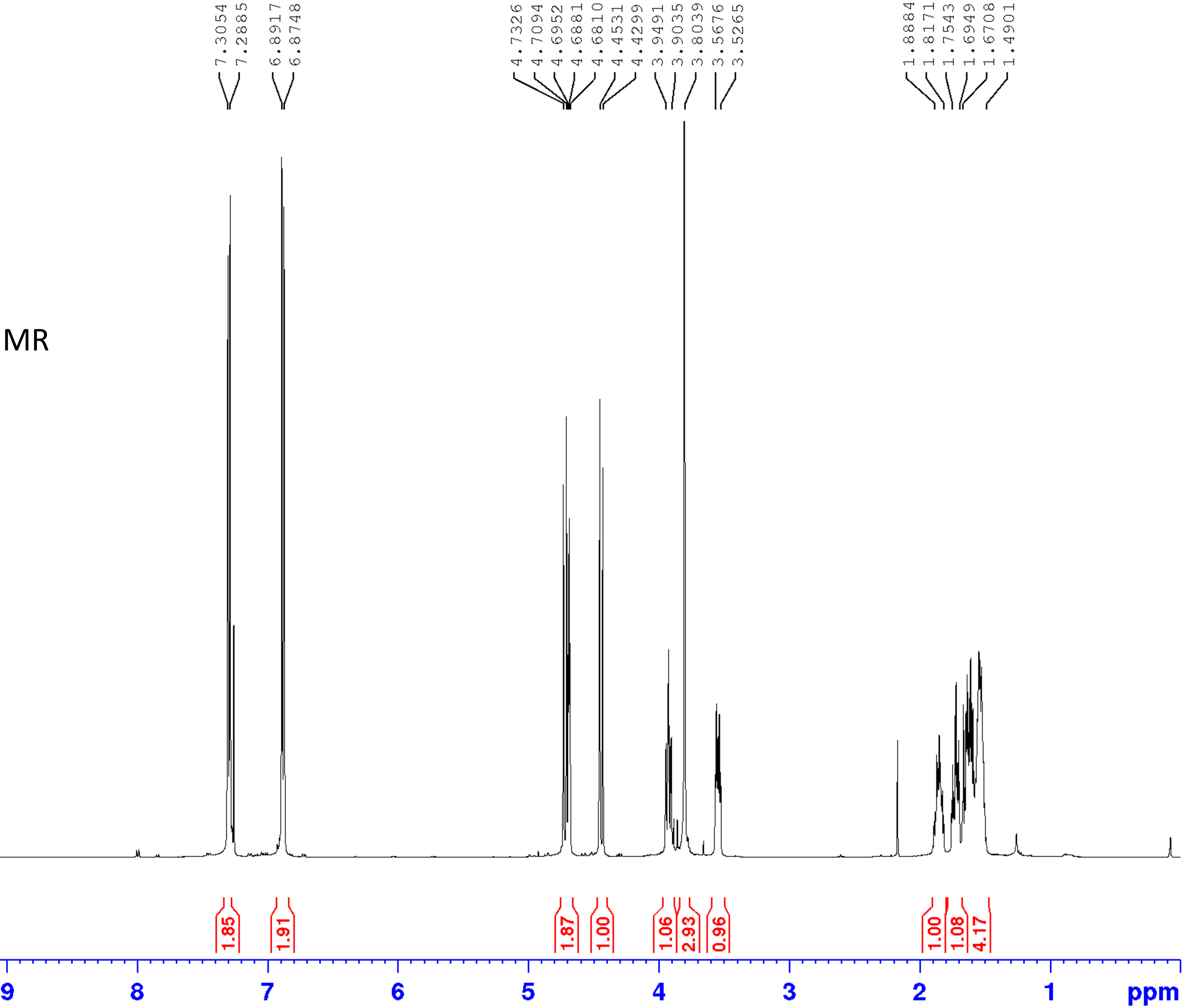

Current Data Parameters
NAME vinn-4-137-7-islt2-2020012
EXPNO 1
PROCNO 1

F2 - Acquisition Parameters
Date_ 20210121
Time 8.38 h
INSTRUM spect
PROBHD Z119470_0283 (
PULPROG zg30
TD 65536
SOLVENT CDCl3
NS 16
DS 2
SWH 10000.000 Hz
FIDRES 0.305176 Hz
AQ 3.2767999 sec
RG 56.83
DW 50.000 usec
DE 6.50 usec
TE 295.2 K
D1 1.00000000 sec
TD0 1
SFO1 500.1330883 MHz
NUC1 1H
P1 10.91 usec
PLW1 25.00000000 W

F2 - Processing parameters
SI 65536
SF 500.1300125 MHz
WDW EM
SSB 0
LB 0.30 Hz
GB 0
PC 1.00

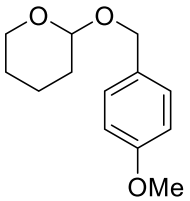

3g

125 MHz ^{13}C NMR

$CDCl_3$

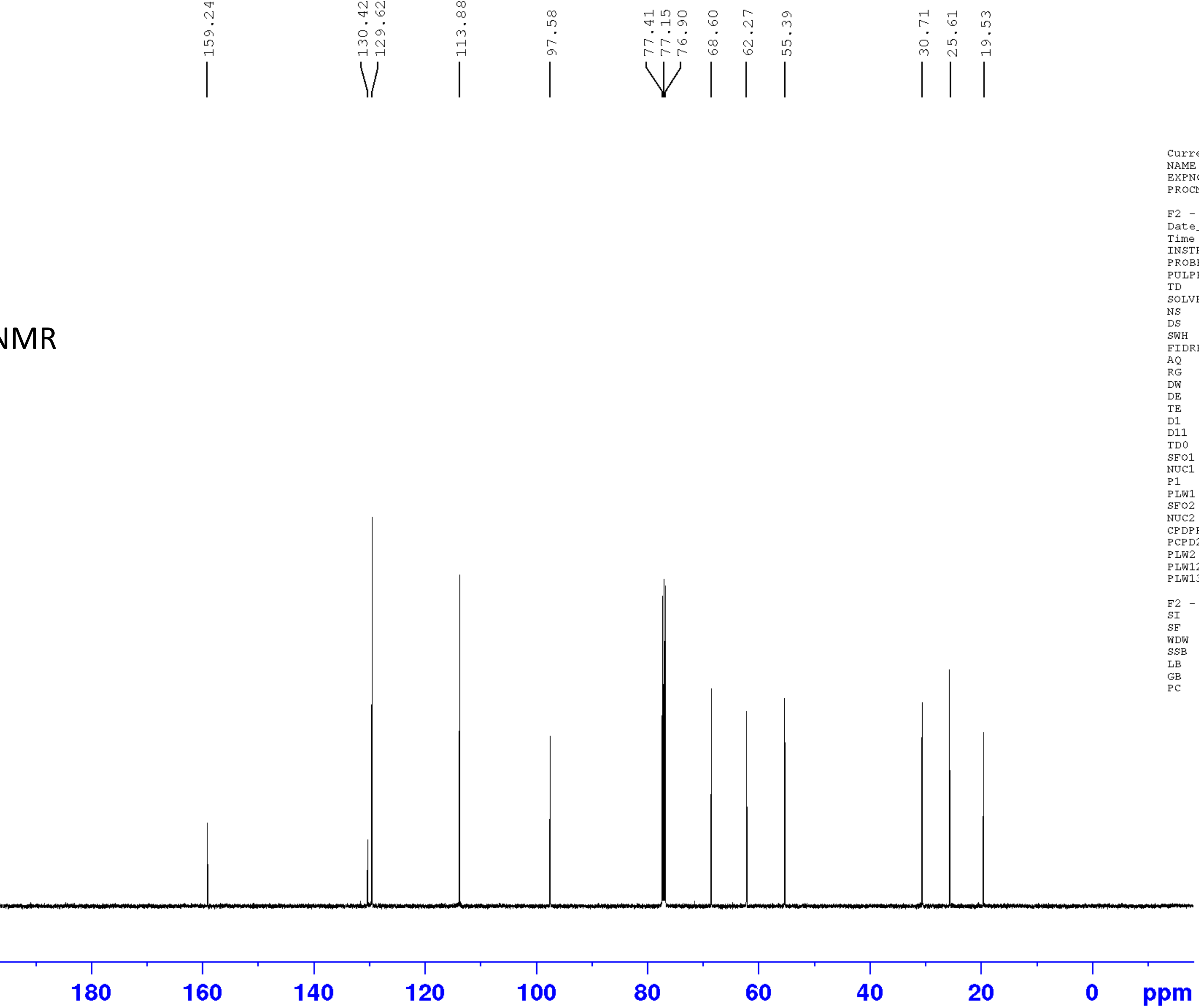


```
Current Data Parameters
NAME      vinn-4-137-7-islt2-20200120
EXPNO                              2
PROCNO                             1

F2 - Acquisition Parameters
Date_              20210121
Time                   8.52 h
INSTRUM               spect
PROBHD    Z119470_0283 (
PULPROG              zgpg30
TD                    65536
SOLVENT               CDCl3
NS                      256
DS                        4
SWH               29761.904 Hz
FIDRES             0.908261 Hz
AQ                1.1010048 sec
RG                   206.72
DW                   16.800 usec
DE                     6.50 usec
TE                    295.2 K
D1               2.00000000 sec
D11              0.03000000 sec
TD0                       1
SFO1            125.7703643 MHz
NUC1                    13C
P1                     9.75 usec
PLW1            94.00000000 W
SFO2            500.1320005 MHz
NUC2                     1H
CPDPRG[2            waltz16
PCPD2                 80.00 usec
PLW2            25.00000000 W
PLW12            0.46495000 W
PLW13            0.23387000 W

F2 - Processing parameters
SI                    32768
SF              125.7577765 MHz
WDW                      EM
SSB                       0
LB                     1.00 Hz
GB                        0
PC                     1.40
```

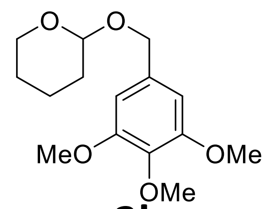

3h

500 MHz 1H NMR

$CDCl_3$

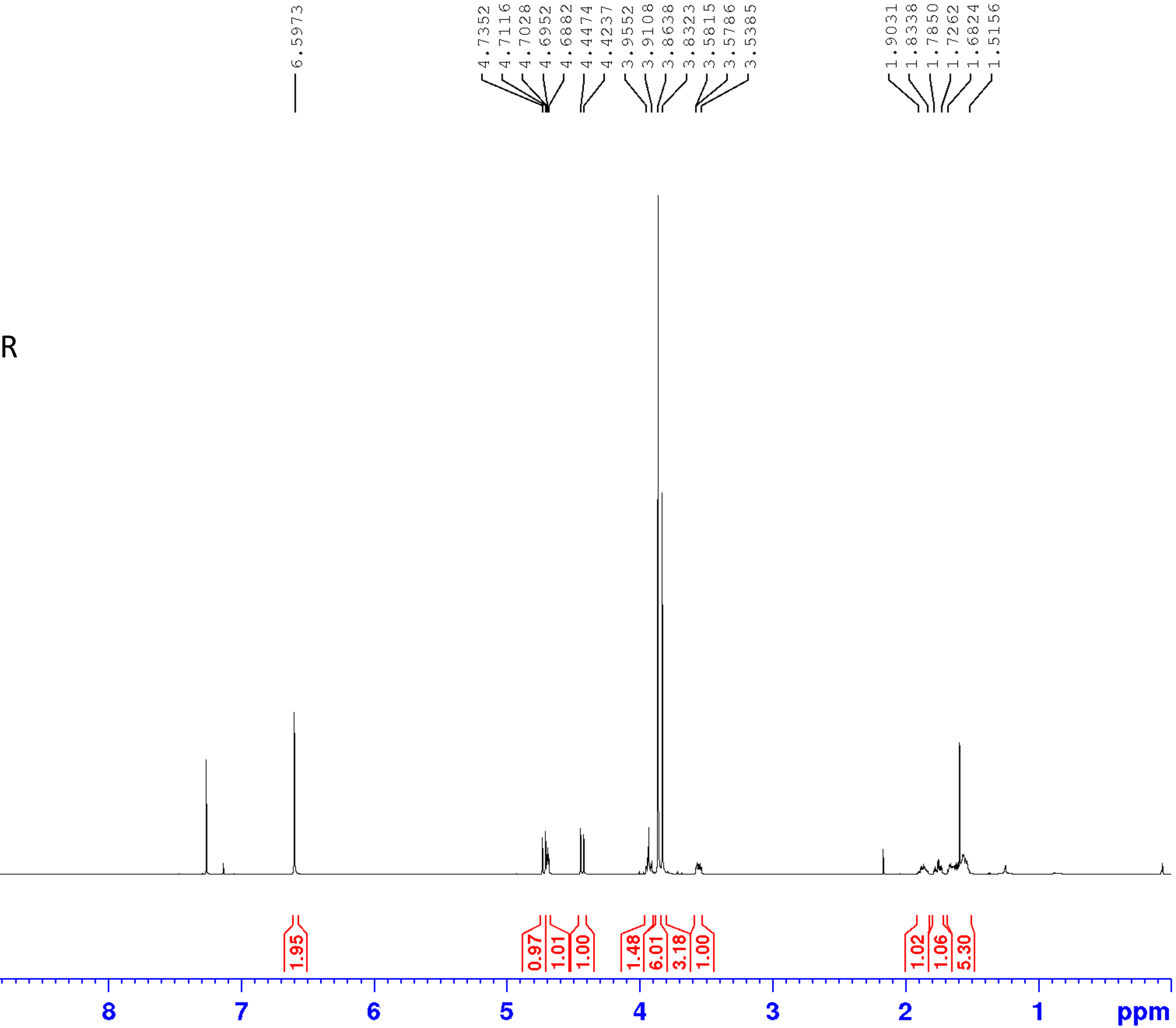


```
Current Data Parameters
NAME     vinn-4-137-6-islt2-2020012
EXPNO                  1
PROCNO                 1

F2 - Acquisition Parameters
Date_          20210121
Time               8.20 h
INSTRUM           spect
PROBHD   Z119470_0283 (
PULPROG            zg30
TD                65536
SOLVENT           CDCl3
NS                   16
DS                    2
SWH           10000.000 Hz
FIDRES         0.305176 Hz
AQ            3.2767999 sec
RG                102.6
DW               50.000 usec
DE                 6.50 usec
TE                295.1 K
D1           1.00000000 sec
TD0                   1
SFO1        500.1330883 MHz
NUC1                 1H
P1                10.91 usec
PLW1        25.00000000 W

F2 - Processing parameters
SI                65536
SF          500.1300123 MHz
WDW                  EM
SSB                   0
LB                 0.30 Hz
GB                    0
PC                 1.00
```

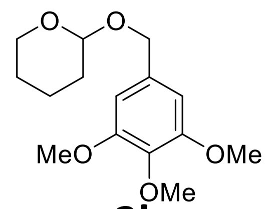

3h

125 MHz ^{13}C NMR

$CDCl_3$

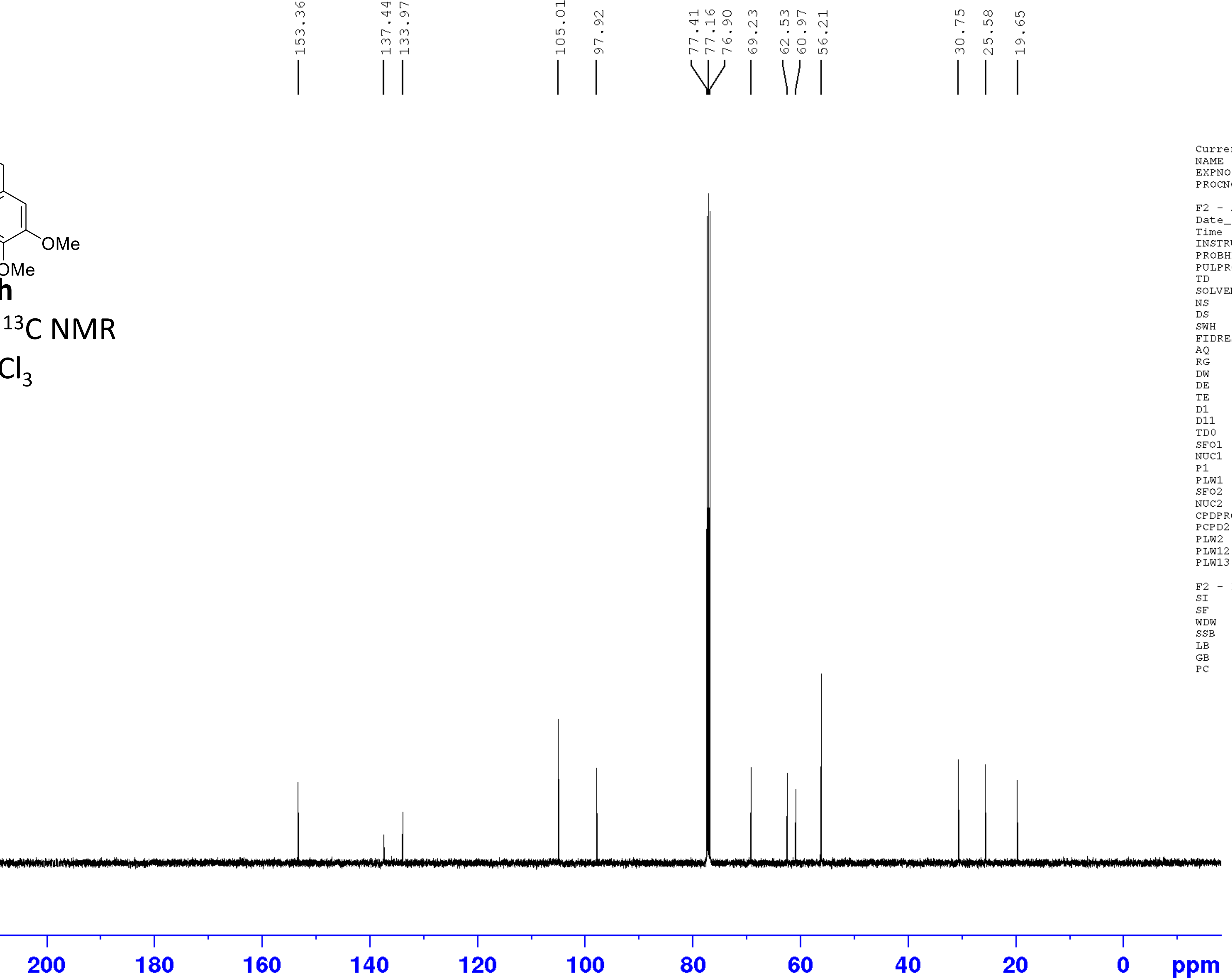


```
Current Data Parameters
NAME      vinn-4-137-6-islt2-20200120
EXPNO                         2
PROCNO                        1

F2 - Acquisition Parameters
Date_               20210121
Time                    8.34 h
INSTRUM                spect
PROBHD    Z119470_0283 (
PULPROG               zgpg30
TD                     65536
SOLVENT                CDCl3
NS                       256
DS                         4
SWH               29761.904 Hz
FIDRES             0.908261 Hz
AQ                1.1010048 sec
RG                   206.72
DW                   16.800 usec
DE                     6.50 usec
TE                    295.2 K
D1               2.00000000 sec
D11              0.03000000 sec
TD0                        1
SFO1            125.7703643 MHz
NUC1                     13C
P1                      9.75 usec
PLW1            94.00000000 W
SFO2            500.1320005 MHz
NUC2                      1H
CPDPRG[2             waltz16
PCPD2                  80.00 usec
PLW2            25.00000000 W
PLW12            0.46495000 W
PLW13            0.23387000 W

F2 - Processing parameters
SI                     32768
SF               125.7577734 MHz
WDW                       EM
SSB                        0
LB                      1.00 Hz
GB                         0
PC                      1.40
```

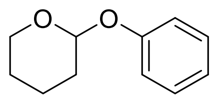

3i

500 MHz ^1H NMR

$CDCl_3$

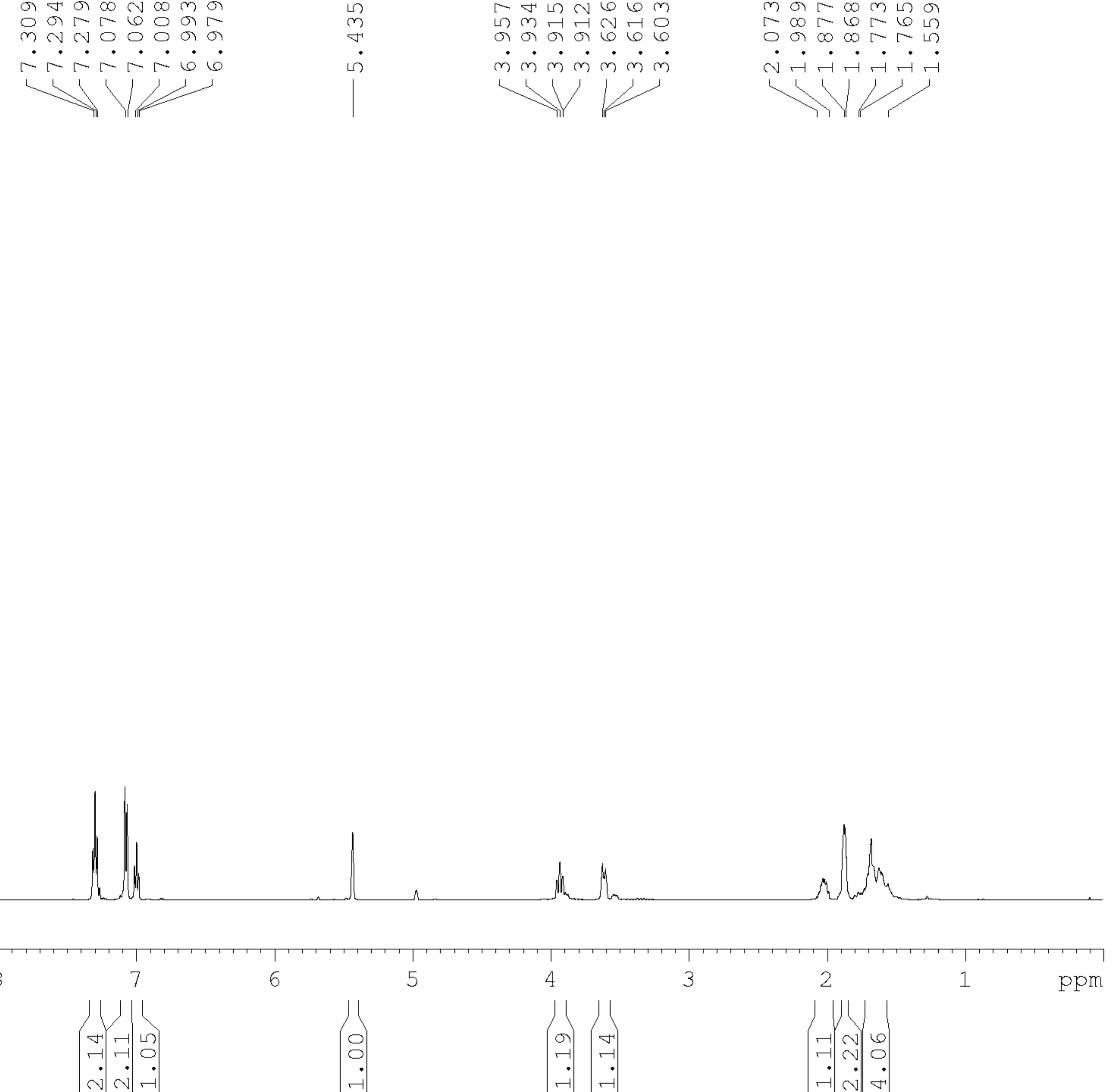

Current Data Parameters
NAME vinn-4-139-1-islt-20201222
EXPNO 1
PROCNO 1

F2 - Acquisition Parameters
Date_ 20201223
Time 8.33 h
INSTRUM spect
PROBHD Z119470_0283 (
PULPROG zg30
TD 65536
SOLVENT CDCl3
NS 16
DS 2
SWH 10000.000 Hz
FIDRES 0.305176 Hz
AQ 3.2767999 sec
RG 30.85
DW 50.000 usec
DE 6.50 usec
TE 295.2 K
D1 1.00000000 sec
TD0 1
SFO1 500.1330883 MHz
NUC1 1H
P1 10.91 usec
PLW1 25.00000000 W

F2 - Processing parameters
SI 65536
SF 500.1300123 MHz
WDW EM
SSB 0
LB 0.30 Hz
GB 0
PC 1.00

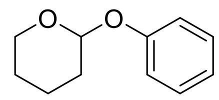

3i
125 MHz ^{13}C NMR
$CDCl_3$

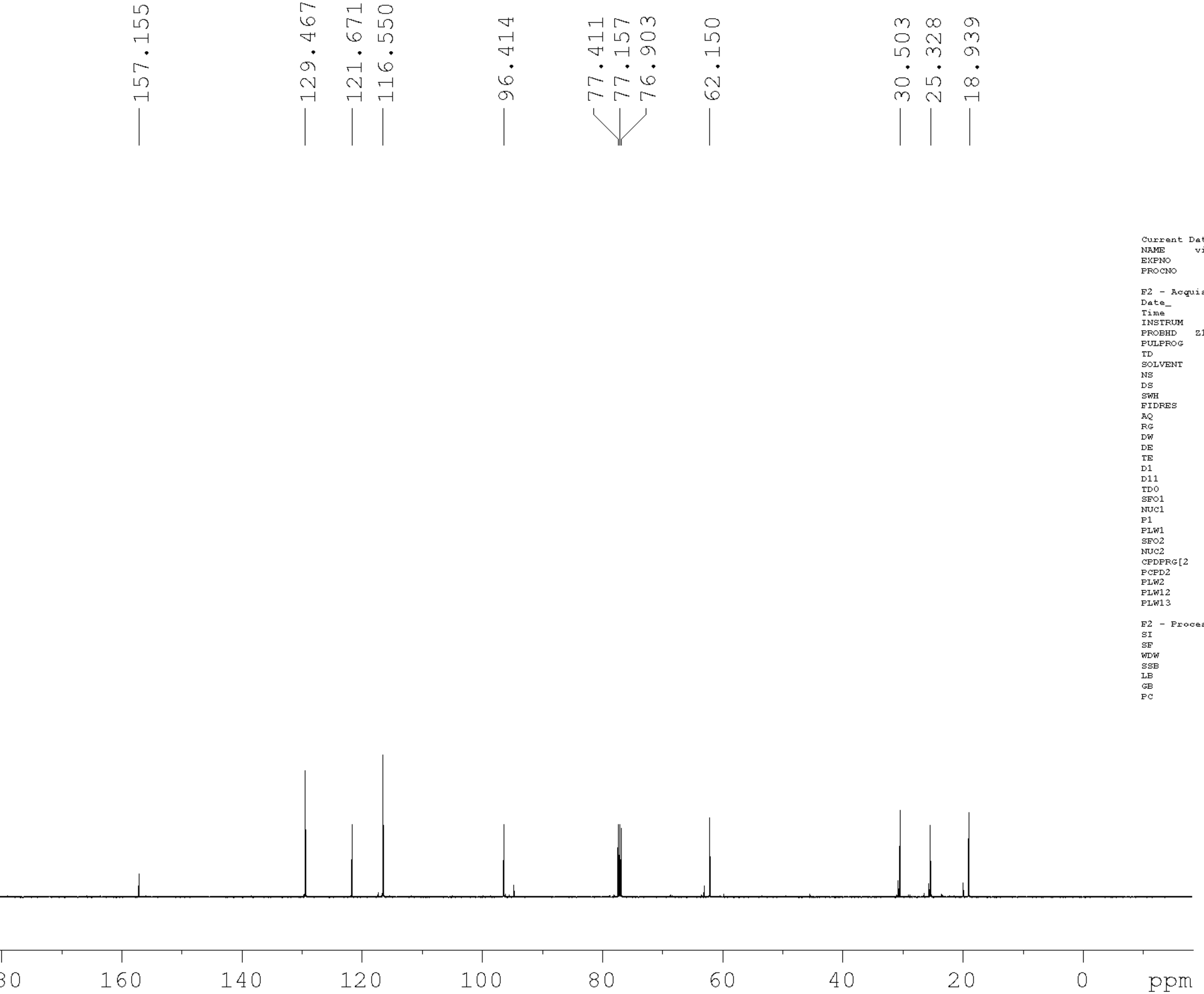

Current Data Parameters

NAME	vinn-4-139-1-islt-20201222
EXPNO	2
PROCNO	1

F2 - Acquisition Parameters

Date_	20201223
Time	8.41 h
INSTRUM	spect
PROBHD	Z119470_0283 (
PULPROG	zgpg30
TD	65536
SOLVENT	CDCl3
NS	128
DS	4
SWH	29761.904 Hz
FIDRES	0.908261 Hz
AQ	1.1010048 sec
RG	206.72
DW	16.800 usec
DE	6.50 usec
TE	295.2 K
D1	2.00000000 sec
D11	0.03000000 sec
TD0	1
SFO1	125.7703643 MHz
NUC1	13C
P1	9.75 usec
PLW1	94.00000000 W
SFO2	500.1320005 MHz
NUC2	1H
CPDPRG[2	waltz16
PCPD2	80.00 usec
PLW2	25.00000000 W
PLW12	0.46495000 W
PLW13	0.23387000 W

F2 - Processing parameters

SI	32768
SF	125.7577796 MHz
WDW	EM
SSB	0
LB	1.00 Hz
GB	0
PC	1.40

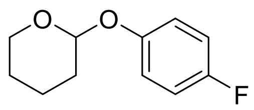

3j

500 MHz ^{1}H NMR

$CDCl_3$

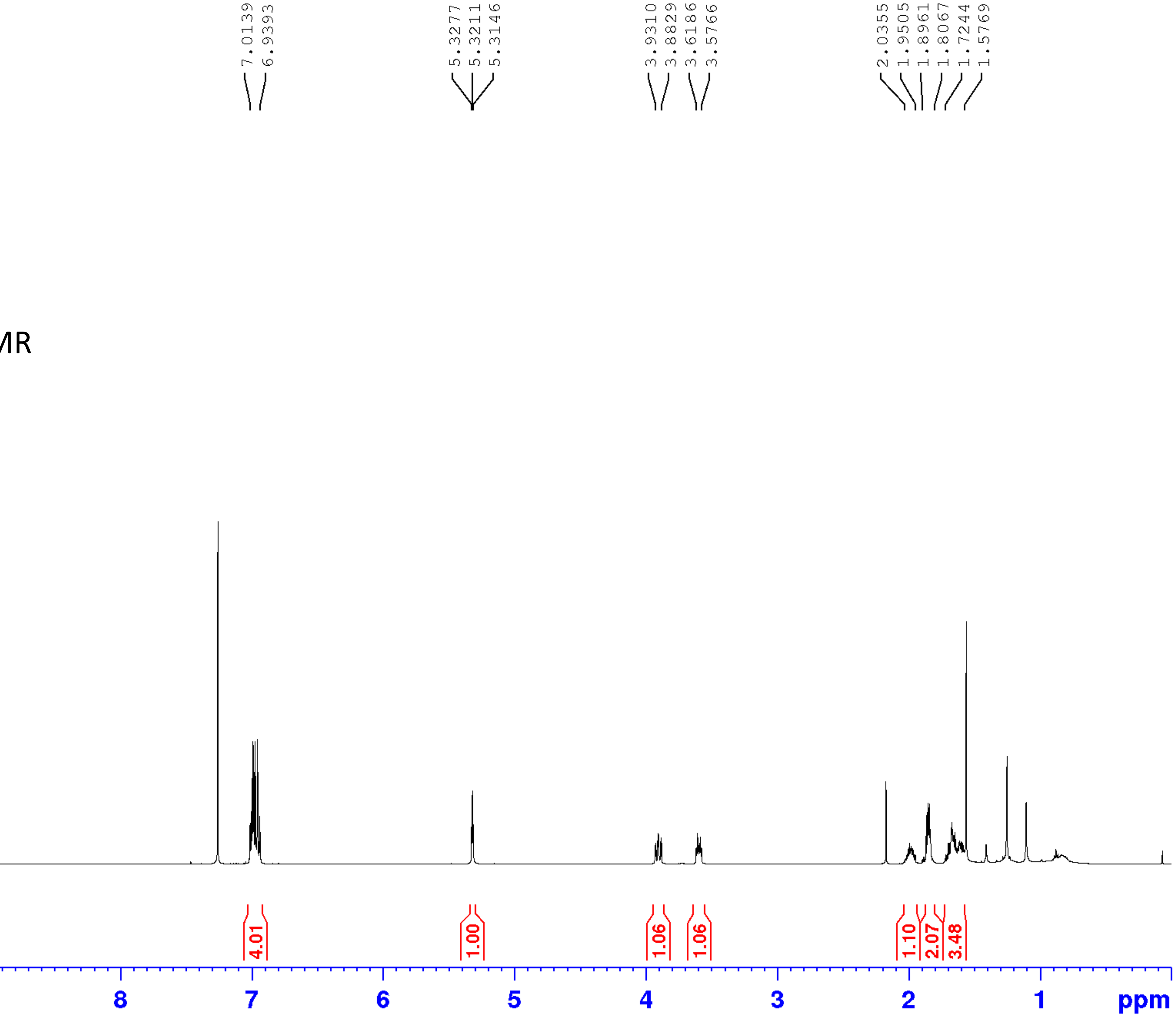


```
Current Data Parameters
NAME     vinn-4-139-4-2-islt2-20200120
EXPNO                    1
PROCNO                   1

F2 - Acquisition Parameters
Date_             20210121
Time                  9.14 h
INSTRUM              spect
PROBHD   Z119470_0283 (
PULPROG               zg30
TD                   65536
SOLVENT              CDCl3
NS                      16
DS                       2
SWH              10000.000 Hz
FIDRES            0.305176 Hz
AQ               3.2767999 sec
RG                  117.01
DW                  50.000 usec
DE                    6.50 usec
TE                   295.1 K
D1              1.00000000 sec
TD0                      1
SFO1           500.1330883 MHz
NUC1                    1H
P1                   10.91 usec
PLW1           25.00000000 W

F2 - Processing parameters
SI                   65536
SF             500.1300124 MHz
WDW                     EM
SSB                      0
LB                    0.30 Hz
GB                       0
PC                    1.00
```

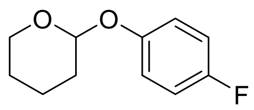

3j

125 MHz ^{13}C NMR

$CDCl_3$

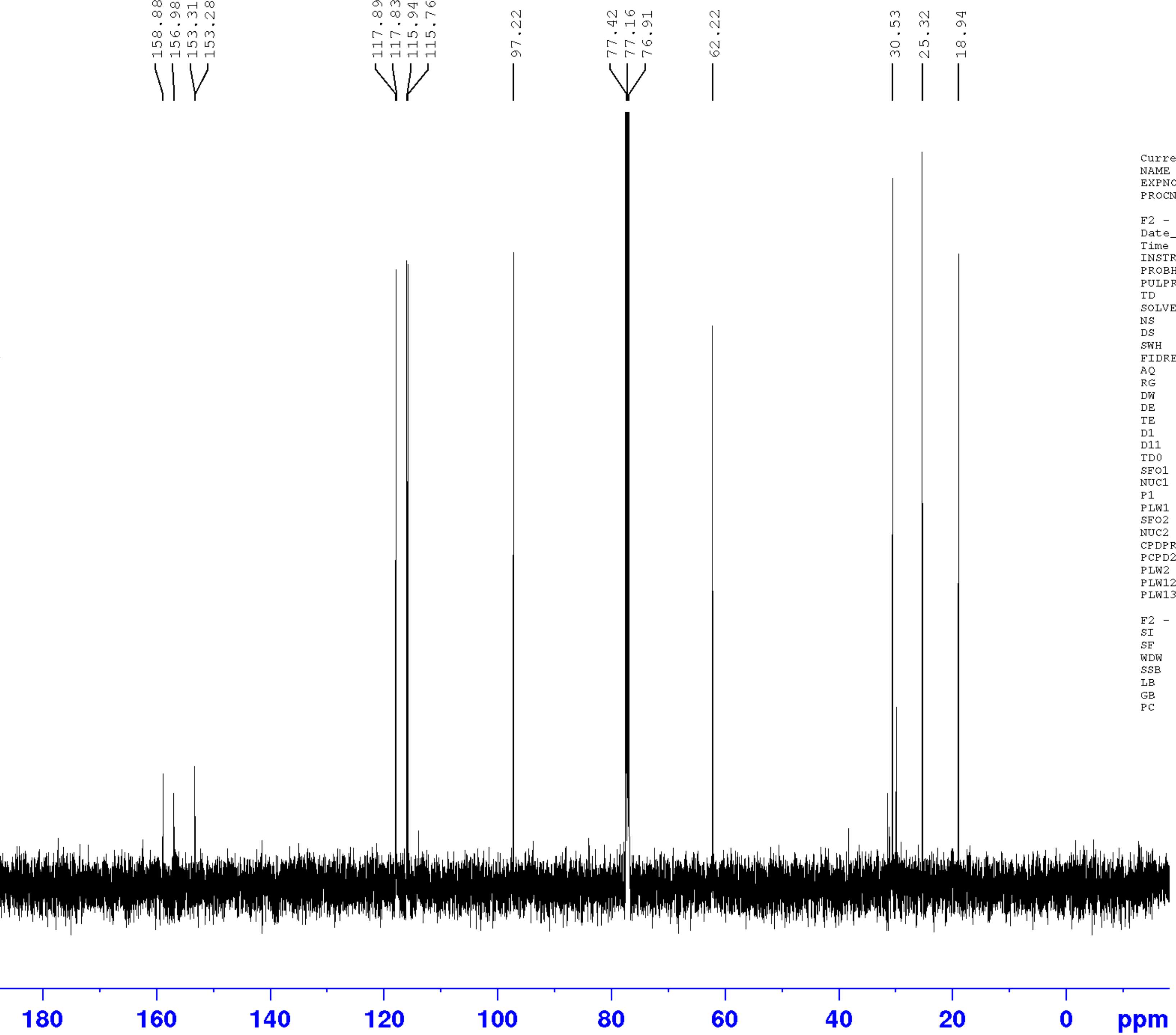

Current Data Parameters
NAME vinn-4-139-4-2-islt2-2020012
EXPNO 2
PROCNO 1

F2 - Acquisition Parameters
Date_ 20210121
Time 9.28 h
INSTRUM spect
PROBHD Z119470_0283 (
PULPROG zgpg30
TD 65536
SOLVENT CDCl3
NS 256
DS 4
SWH 29761.904 Hz
FIDRES 0.908261 Hz
AQ 1.1010048 sec
RG 206.72
DW 16.800 usec
DE 6.50 usec
TE 295.2 K
D1 2.00000000 sec
D11 0.03000000 sec
TD0 1
SFO1 125.7703643 MHz
NUC1 13C
P1 9.75 usec
PLW1 94.00000000 W
SFO2 500.1320005 MHz
NUC2 1H
CPDPRG[2 waltz16
PCPD2 80.00 usec
PLW2 25.00000000 W
PLW12 0.46495000 W
PLW13 0.23387000 W

F2 - Processing parameters
SI 32768
SF 125.7665629 MHz
WDW EM
SSB 0
LB 1.00 Hz
GB 0
PC 1.40

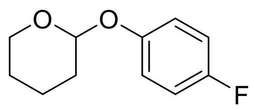

3j

470 MHz ^{19}F NMR

$CDCl_3$

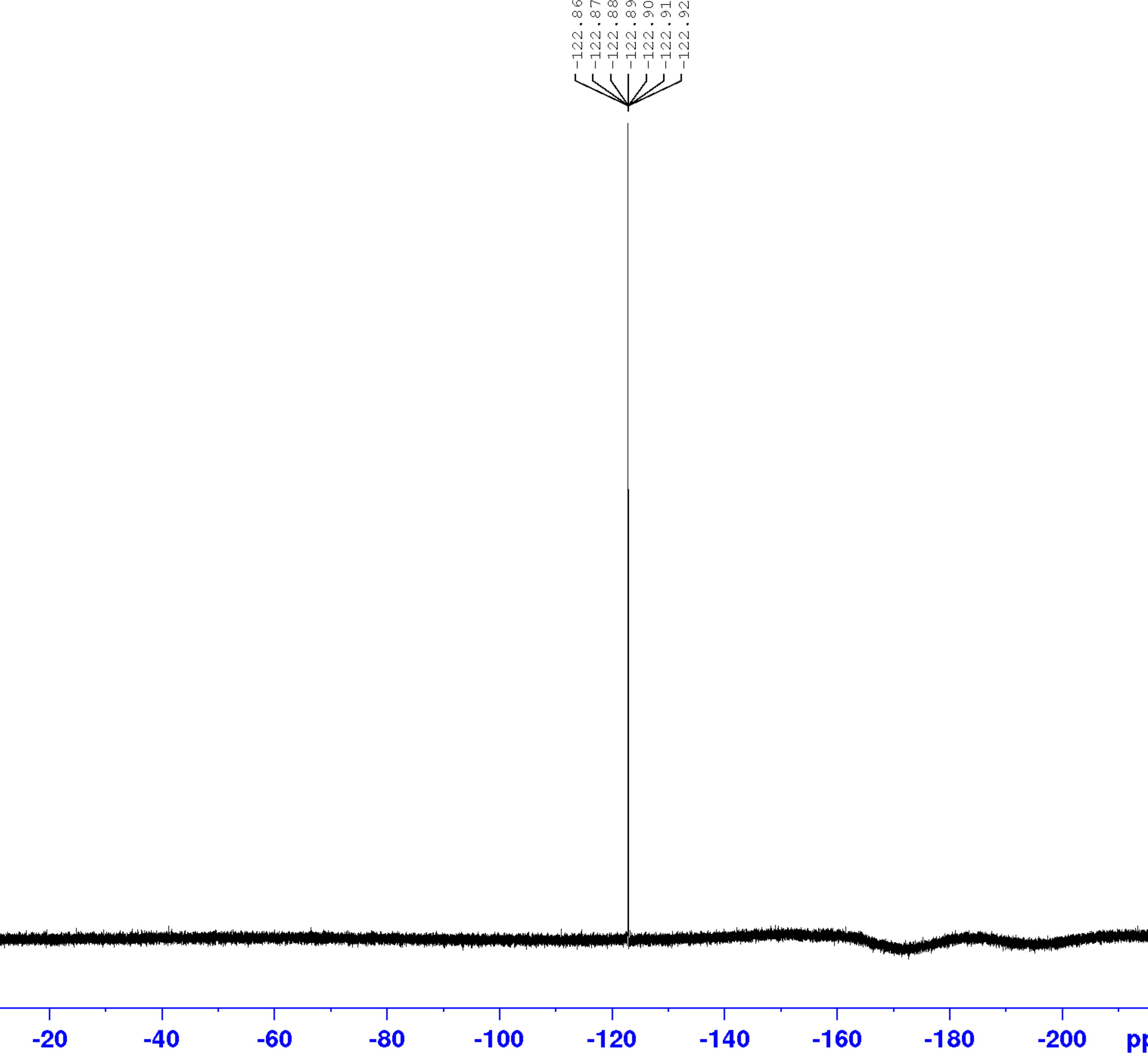


```
Current Data Parameters
NAME      vinn-4-139-4-2-islt2-2020012
EXPNO                     3
PROCNO                    1

F2 - Acquisition Parameters
Date_              20210121
Time                 9.30 h
INSTRUM               spect
PROBHD    Z119470_0283 (
PULPROG              zgflqn
TD                   131072
SOLVENT               CDCl3
NS                       16
DS                        4
SWH           113636.367 Hz
FIDRES          1.733953 Hz
AQ             0.5767168 sec
RG                   206.72
DW                 4.400 usec
DE                  6.50 usec
TE                  295.2 K
D1            1.00000000 sec
TD0                       1
SFO1         470.5453180 MHz
NUC1                    19F
P1                 15.00 usec
PLW1         47.23500061 W

F2 - Processing parameters
SI                    65536
SF           470.5923772 MHz
WDW                      EM
SSB                       0
LB                  0.30 Hz
GB                        0
PC                     1.00
```

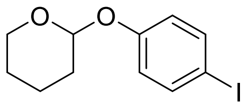

3k

500 MHz ^{1}H NMR

$CDCl_3$

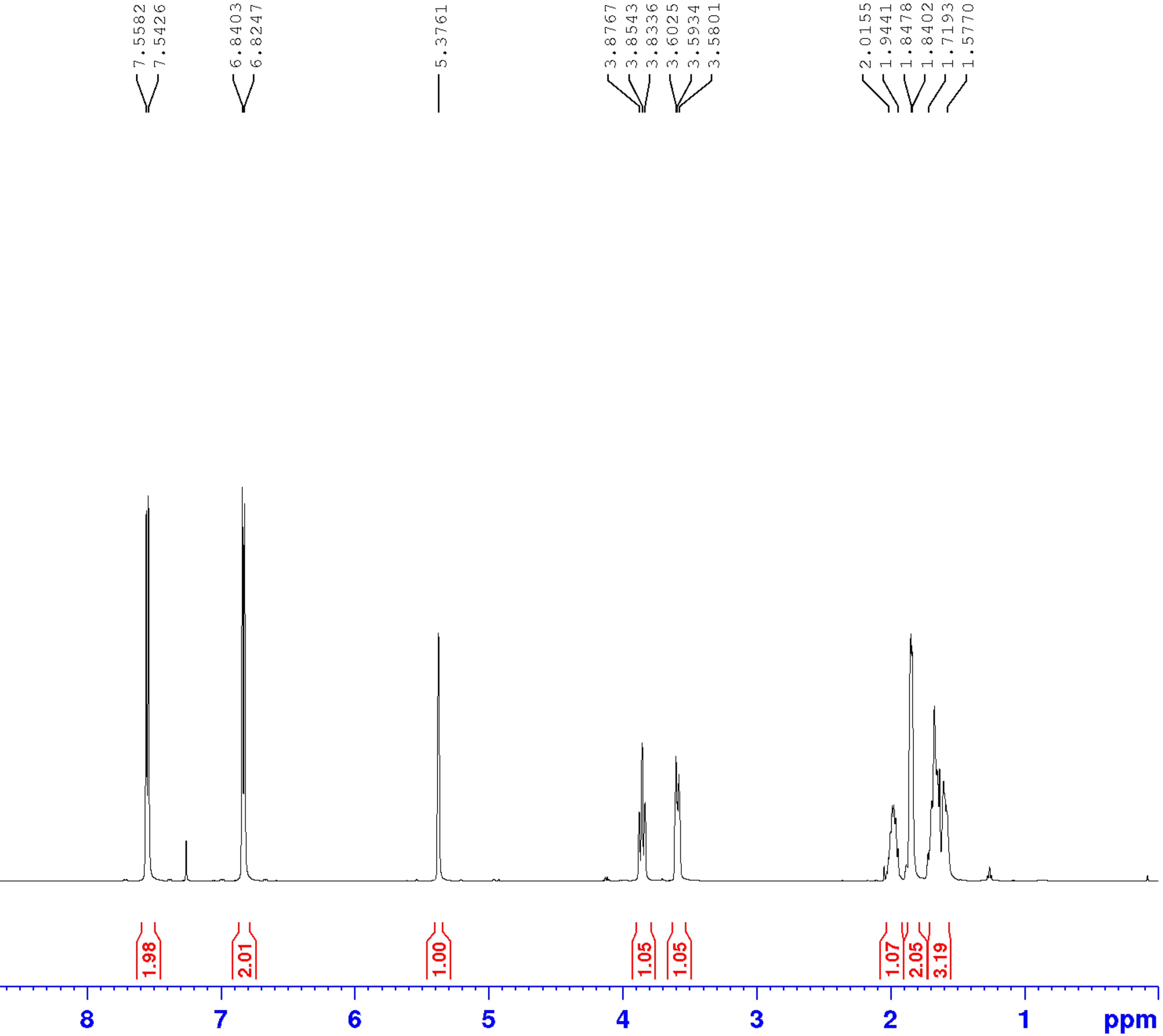


```
Current Data Parameters
NAME      vinn-4-139-3-islt-20201222
EXPNO                    1
PROCNO                   1

F2 - Acquisition Parameters
Date_             20201223
Time                  8.44 h
INSTRUM              spect
PROBHD    Z119470_0283 (
PULPROG               zg30
TD                   65536
SOLVENT              CDCl3
NS                      16
DS                       2
SWH              10000.000 Hz
FIDRES            0.305176 Hz
AQ               3.2767999 sec
RG                   30.85
DW                  50.000 usec
DE                    6.50 usec
TE                   295.2 K
D1              1.00000000 sec
TD0                      1
SFO1           500.1330883 MHz
NUC1                    1H
P1                   10.91 usec
PLW1           25.00000000 W

F2 - Processing parameters
SI                   65536
SF             500.1300120 MHz
WDW                     EM
SSB                      0
LB                    0.30 Hz
GB                       0
PC                    1.00
```

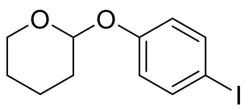

3k

125 MHz ^{13}C NMR

$CDCl_3$

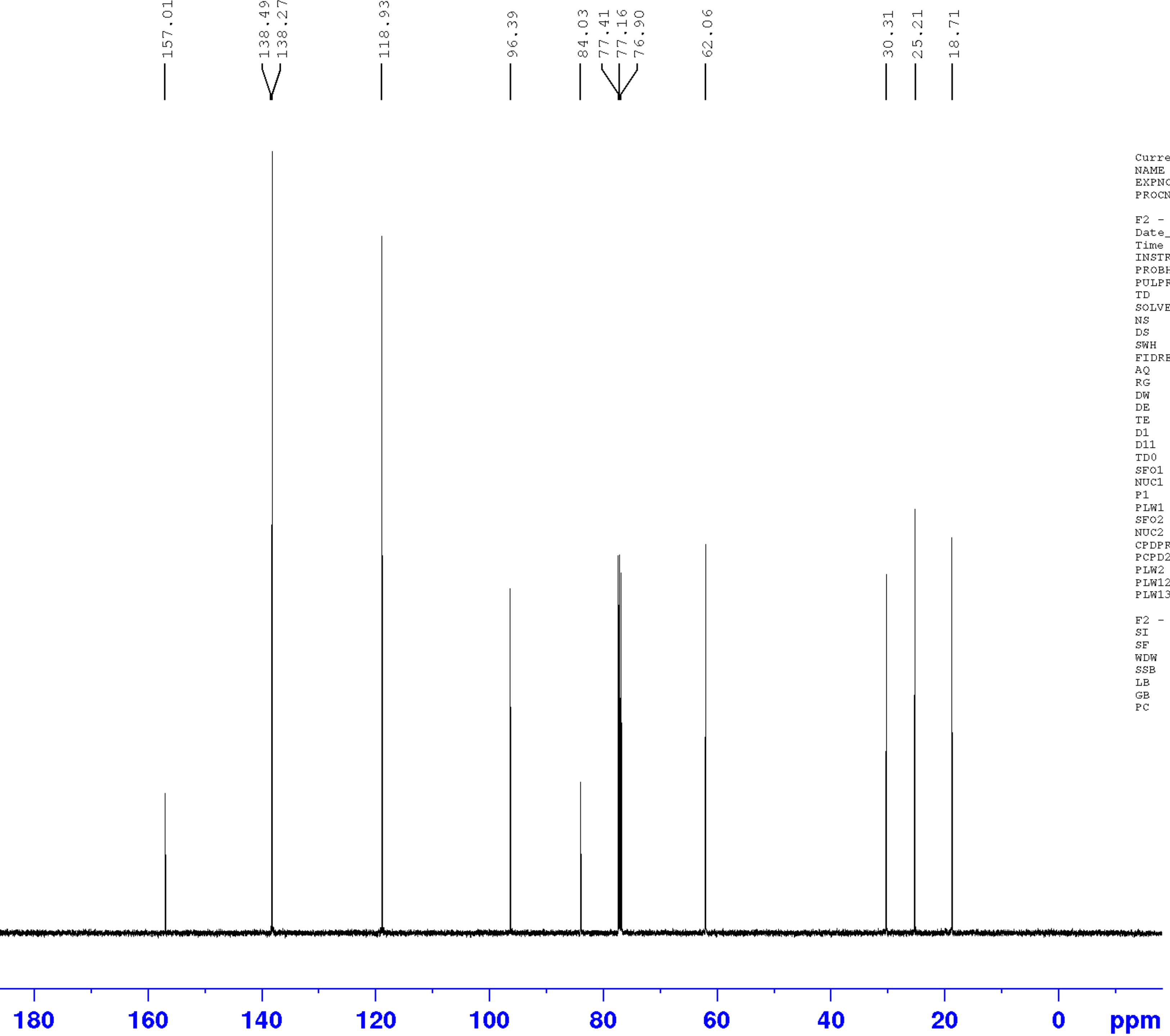


```
Current Data Parameters
NAME     vinn-4-139-3-islt-20201222
EXPNO                    2
PROCNO                   1

F2 - Acquisition Parameters
Date_             20201223
Time                8.51 h
INSTRUM             spect
PROBHD    Z119470_0283 (
PULPROG            zgpg30
TD                  65536
SOLVENT             CDCl3
NS                    128
DS                      4
SWH             29761.904 Hz
FIDRES           0.908261 Hz
AQ              1.1010048 sec
RG                 206.72
DW                 16.800 usec
DE                   6.50 usec
TE                  295.1 K
D1             2.00000000 sec
D11            0.03000000 sec
TD0                     1
SFO1          125.7703643 MHz
NUC1                  13C
P1                   9.75 usec
PLW1          94.00000000 W
SFO2          500.1320005 MHz
NUC2                   1H
CPDPRG[2          waltz16
PCPD2               80.00 usec
PLW2          25.00000000 W
PLW12          0.46495000 W
PLW13          0.23387000 W

F2 - Processing parameters
SI                  32768
SF            125.7577797 MHz
WDW                    EM
SSB                     0
LB                   1.00 Hz
GB                      0
PC                   1.40
```

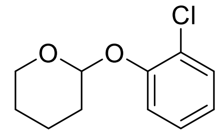

3l

400 MHz ^{1}H NMR

$CDCl_3$

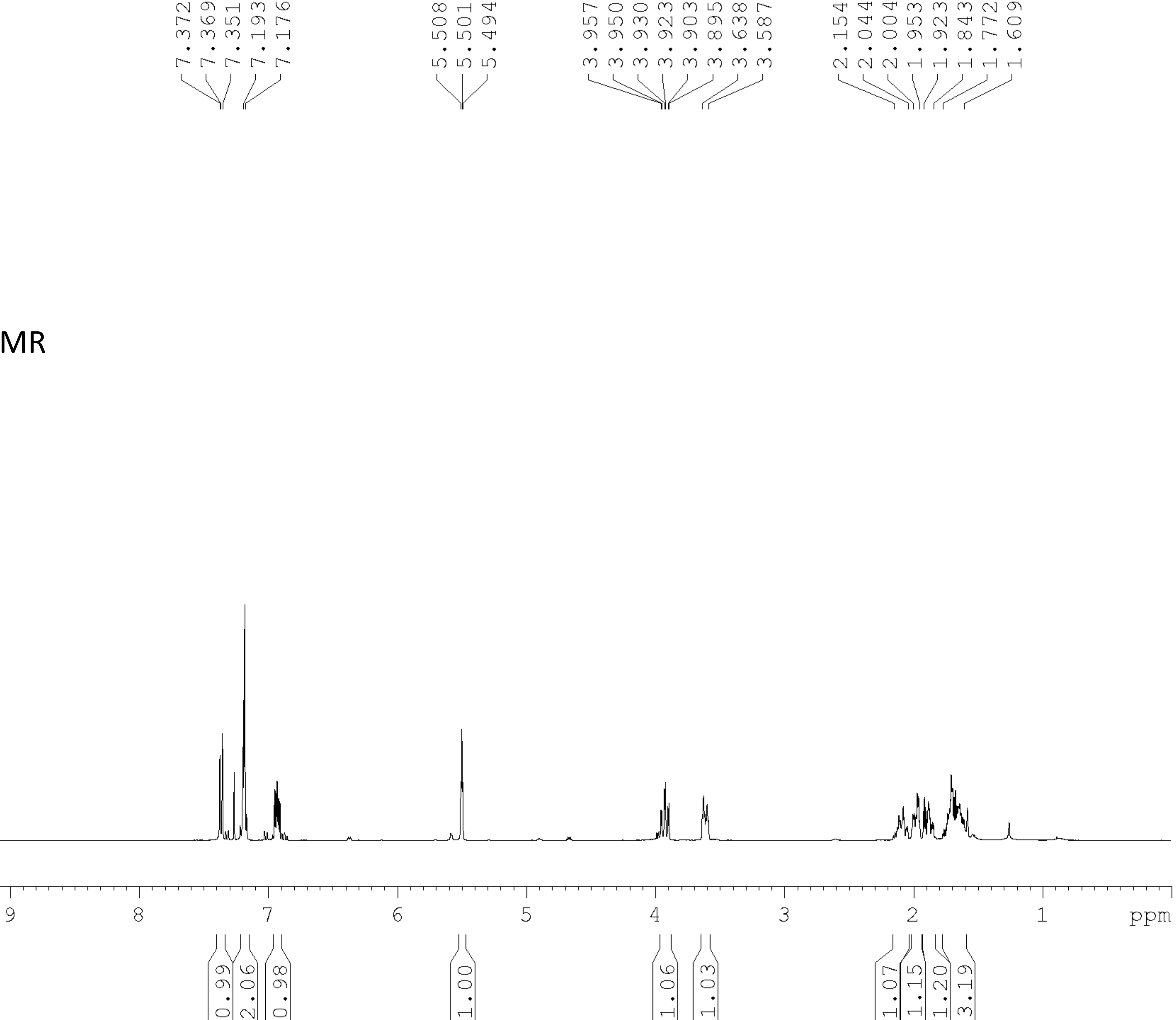


```
Current Data Parameters
NAME      Andy-1-180-3-islt-20220817
EXPNO                    4
PROCNO                   1

F2 - Acquisition Parameters
Date_            20220819
Time                17.55 h
INSTRUM             spect
PROBHD   Z108618_0257 (
PULPROG              zg30
TD                  65536
SOLVENT             CDCl3
NS                     16
DS                      2
SWH              8012.820 Hz
FIDRES           0.244532 Hz
AQ              4.0894465 sec
RG                    203
DW                 62.400 usec
DE                   6.50 usec
TE                  296.2 K
D1             1.00000000 sec
TD0                     1
SFO1          400.1324708 MHz
NUC1                   1H
P1                  15.00 usec
PLW1          12.50000000 W

F2 - Processing parameters
SI                  65536
SF            400.1300098 MHz
WDW                    EM
SSB                     0
LB                   0.30 Hz
GB                      0
PC                   1.00
```

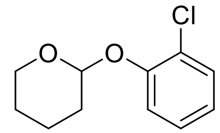

3l

100 MHz ^{13}C NMR

$CDCl_3$

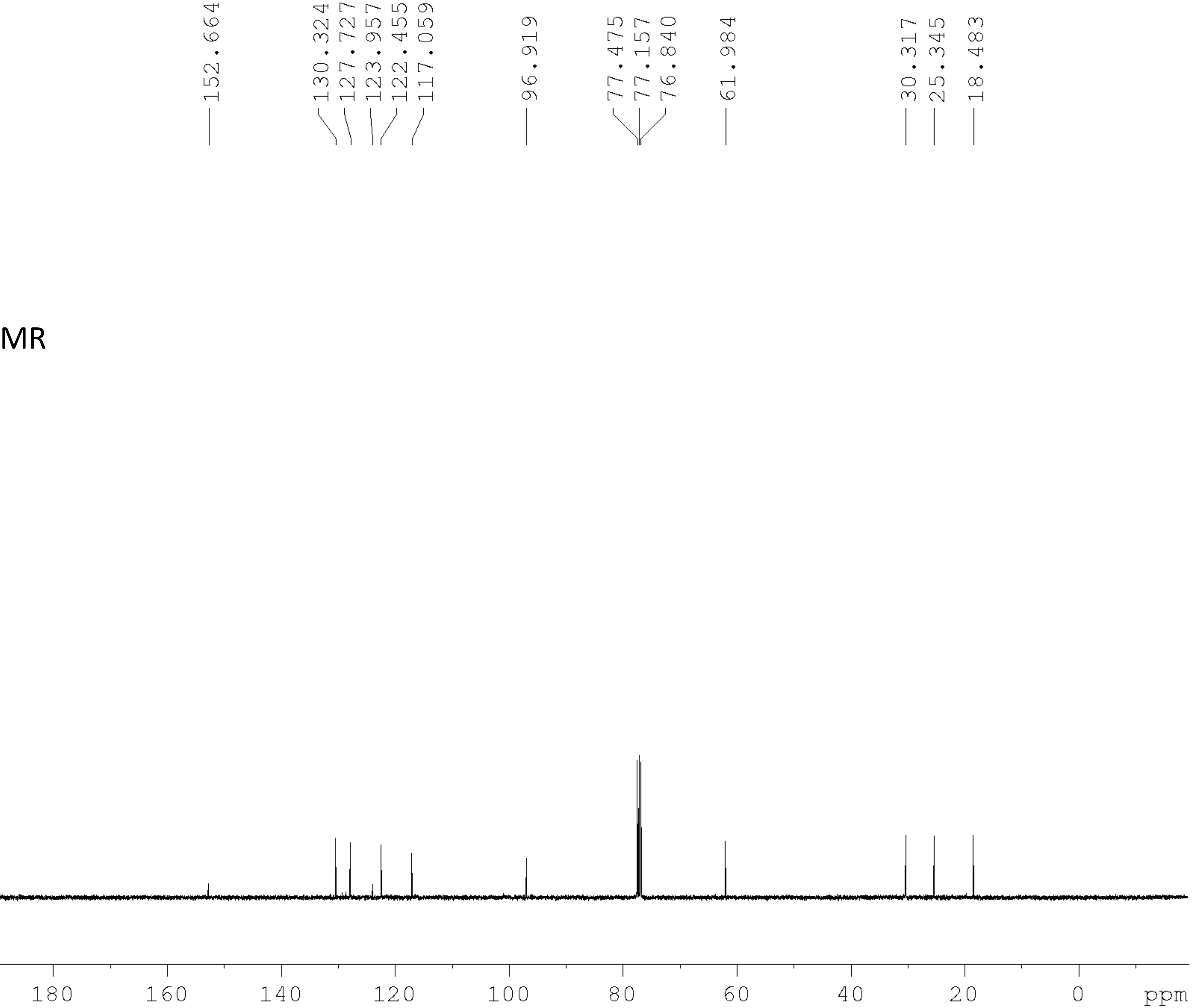


```
Current Data Parameters
NAME      Andy-1-180-3-ialt-20220817
EXPNO                  5
PROCNO                 1

F2 - Acquisition Parameters
Date_           20220819
Time               18.00 h
INSTRUM            spect
PROBHD   Z108618_0257 (
PULPROG          zgpg30
TD                65536
SOLVENT           CDCl3
NS                   62
DS                    4
SWH           24038.461 Hz
FIDRES         0.733596 Hz
AQ            1.3631488 sec
RG                  203
DW               20.800 usec
DE                 6.50 usec
TE                296.6 K
D1           2.00000000 sec
D11          0.03000000 sec
TD0                   1
SFO1        100.6228298 MHz
NUC1                13C
P1                10.00 usec
PLW1        51.00000000 W
SFO2        400.1316005 MHz
NUC2                 1H
CPDPRG[2        waltz16
PCPD2             90.00 usec
PLW2        12.50000000 W
PLW12        0.34722000 W
PLW13        0.17465000 W

F2 - Processing parameters
SI                32768
SF          100.6127565 MHz
```

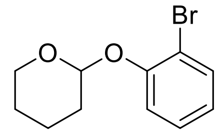

3m

500 MHz ^{1}H NMR

$CDCl_3$

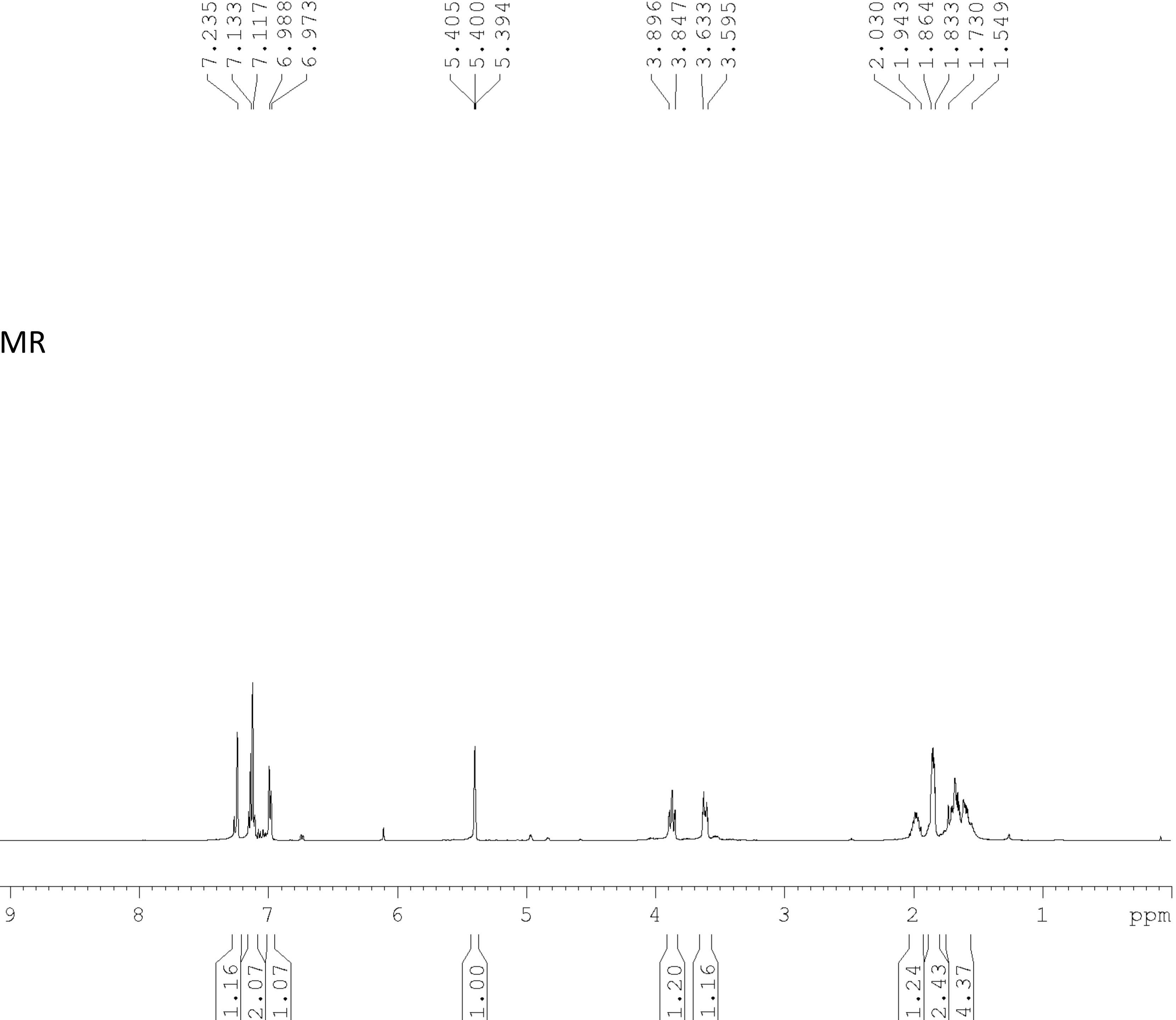

Current Data Parameters
NAME vinn-4-139-2-2-islt2-20210111
EXPNO 1
PROCNO 1

F2 - Acquisition Parameters
Date_ 20210111
Time 18.36 h
INSTRUM spect
PROBHD Z119470_0283 (
PULPROG zg30
TD 65536
SOLVENT CDCl3
NS 16
DS 2
SWH 10000.000 Hz
FIDRES 0.305176 Hz
AQ 3.2767999 sec
RG 30.85
DW 50.000 usec
DE 6.50 usec
TE 295.1 K
D1 1.00000000 sec
TD0 1
SFO1 500.1330883 MHz
NUC1 1H
P1 10.91 usec
PLW1 25.00000000 W

F2 - Processing parameters
SI 65536
SF 500.1300128 MHz
WDW EM
SSB 0
LB 0.30 Hz
GB 0
PC 1.00

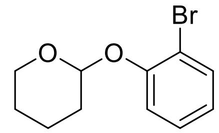

3m

125 MHz ^{13}C NMR

$CDCl_3$

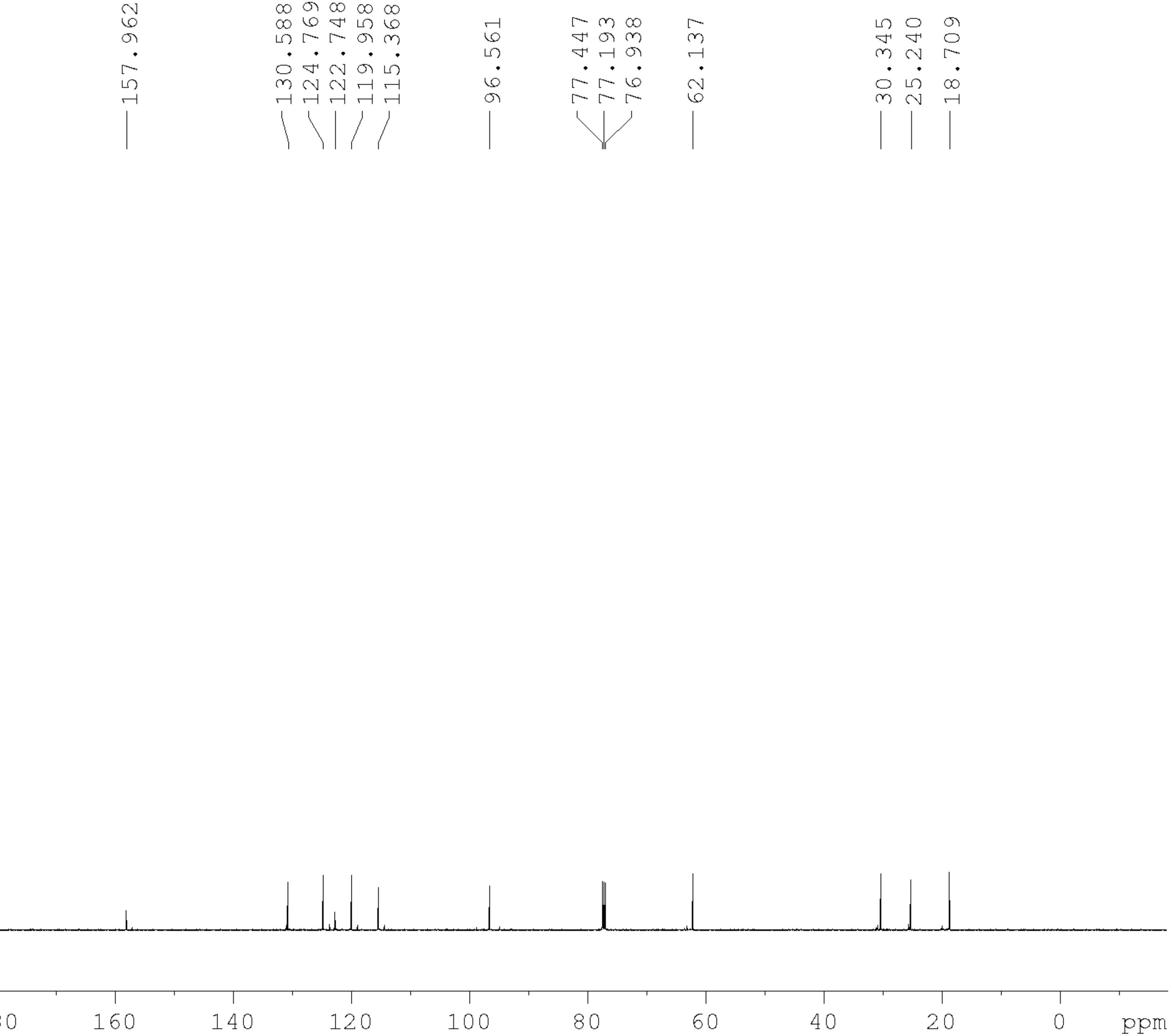


```
Current Data Parameters
NAME     vinn-4-139-2-2-islt2-20210111
EXPNO                 2
PROCNO                1

F2 - Acquisition Parameters
Date_           20210111
Time               18.43 h
INSTRUM            spect
PROBHD   Z119470_0283 (
PULPROG          zgpg30
TD                 65536
SOLVENT            CDCl3
NS                   100
DS                     4
SWH            29761.904 Hz
FIDRES          0.908261 Hz
AQ             1.1010048 sec
RG                117.01
DW                16.800 usec
DE                  6.50 usec
TE                 295.2 K
D1            2.00000000 sec
D11           0.03000000 sec
TD0                    1
SFO1         125.7703643 MHz
NUC1                 13C
P1                  9.75 usec
PLW1         94.00000000 W
SFO2         500.1320005 MHz
NUC2                  1H
CPDPRG[2         waltz16
PCPD2              80.00 usec
PLW2         25.00000000 W
PLW12         0.46495000 W
PLW13         0.23387000 W

F2 - Processing parameters
SI                 32768
SF           125.7577740 MHz
```

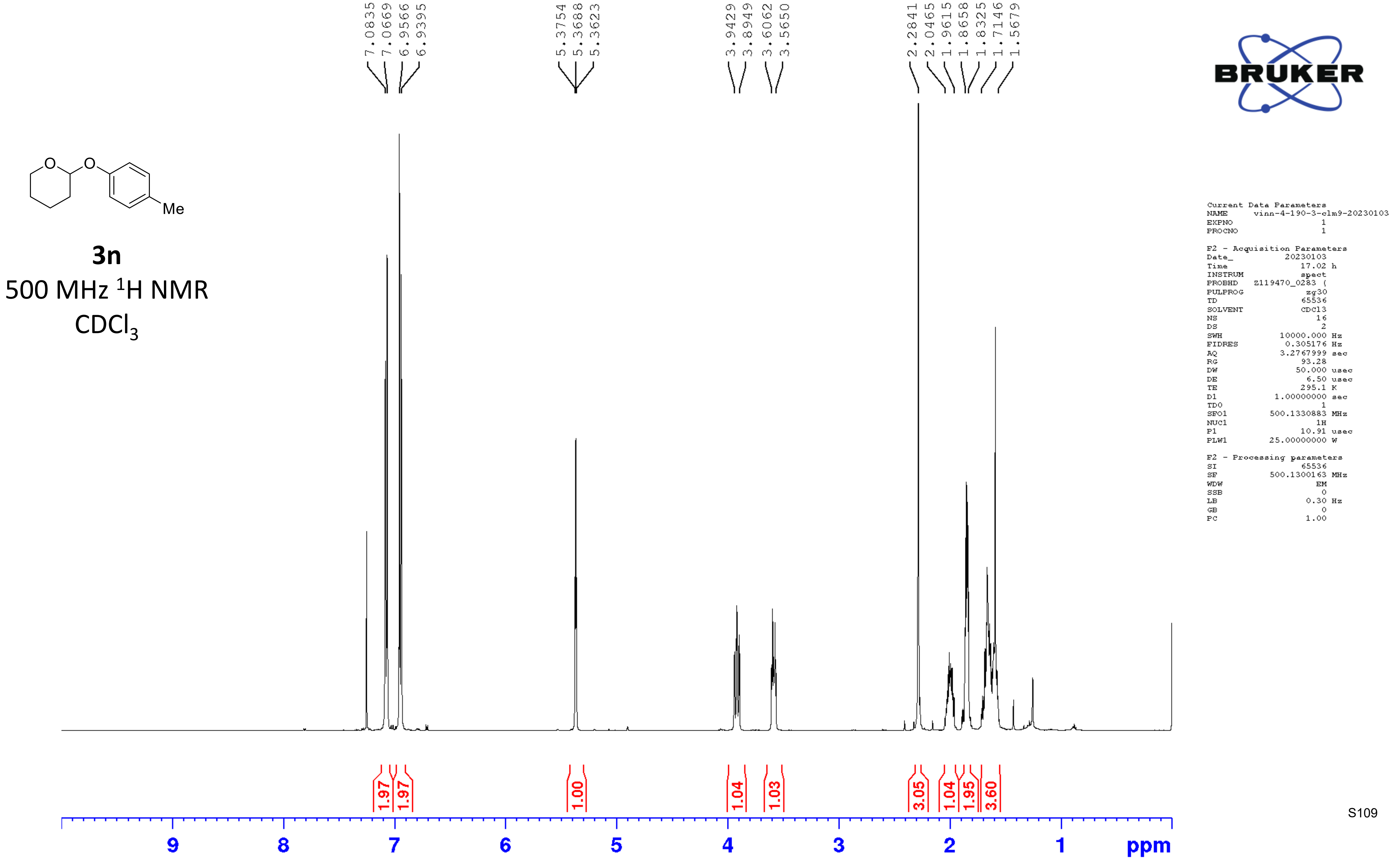
7.0835
7.0669
6.9566
6.9395
5.3754
5.3688
5.3623
3.9429
3.8949
3.6062
3.5650
2.2841
2.0465
1.9615
1.8658
1.8325
1.7146
1.5679
BRUKER
Me
3n
500 MHz 1H NMR
CDCl3
Current Data Parameters
NAME vinn-4-190-3-clm9-20230103
EXPNO 1
PROCNO 1
F2 - Acquisition Parameters
Date_ 20230103
Time 17.02 h
INSTRUM spect
PROBHD Z119470_0283 (
PULPROG zg30
TD 65536
SOLVENT CDCl3
NS 16
DS 2
SWH 10000.000 Hz
FIDRES 0.305176 Hz
AQ 3.2767999 sec
RG 93.28
DW 50.000 usec
DE 6.50 usec
TE 295.1 K
D1 1.00000000 sec
TD0 1
SFO1 500.1330883 MHz
NUC1 1H
P1 10.91 usec
PLW1 25.00000000 W
F2 - Processing parameters
SI 65536
SF 500.1300163 MHz
WDW EM
SSB 0
LB 0.30 Hz
GB 0
PC 1.00
1.97
1.97
1.00
1.04
1.03
3.05
1.04
1.95
3.60
9
8
7
6
5
4
3
2
1
ppm

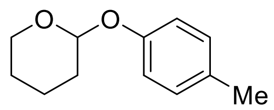

3n

125 MHz ^{13}C NMR

$CDCl_3$

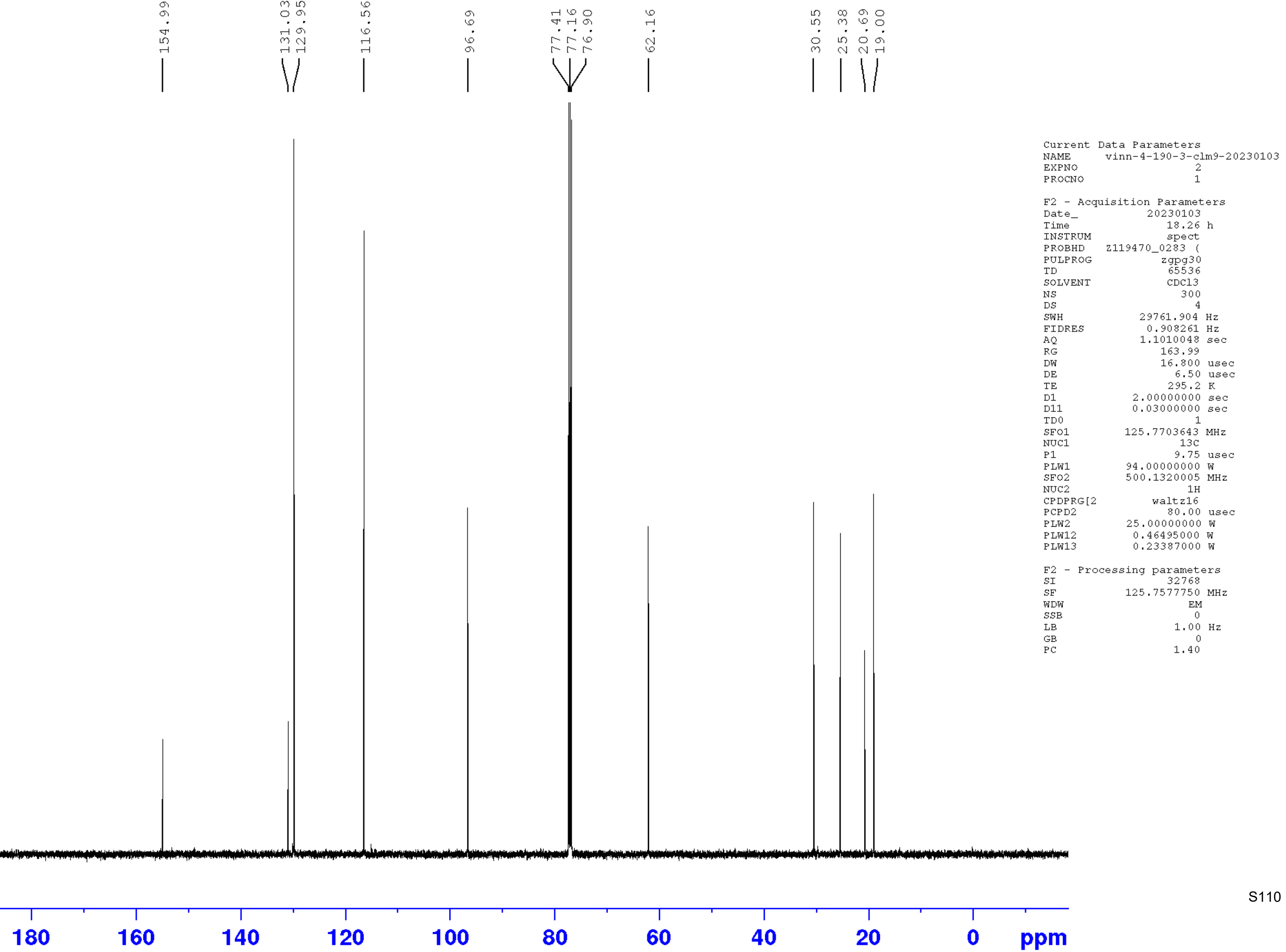

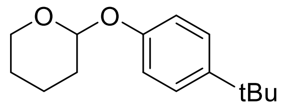

3o
500 MHz ^{1}H NMR
$CDCl_3$

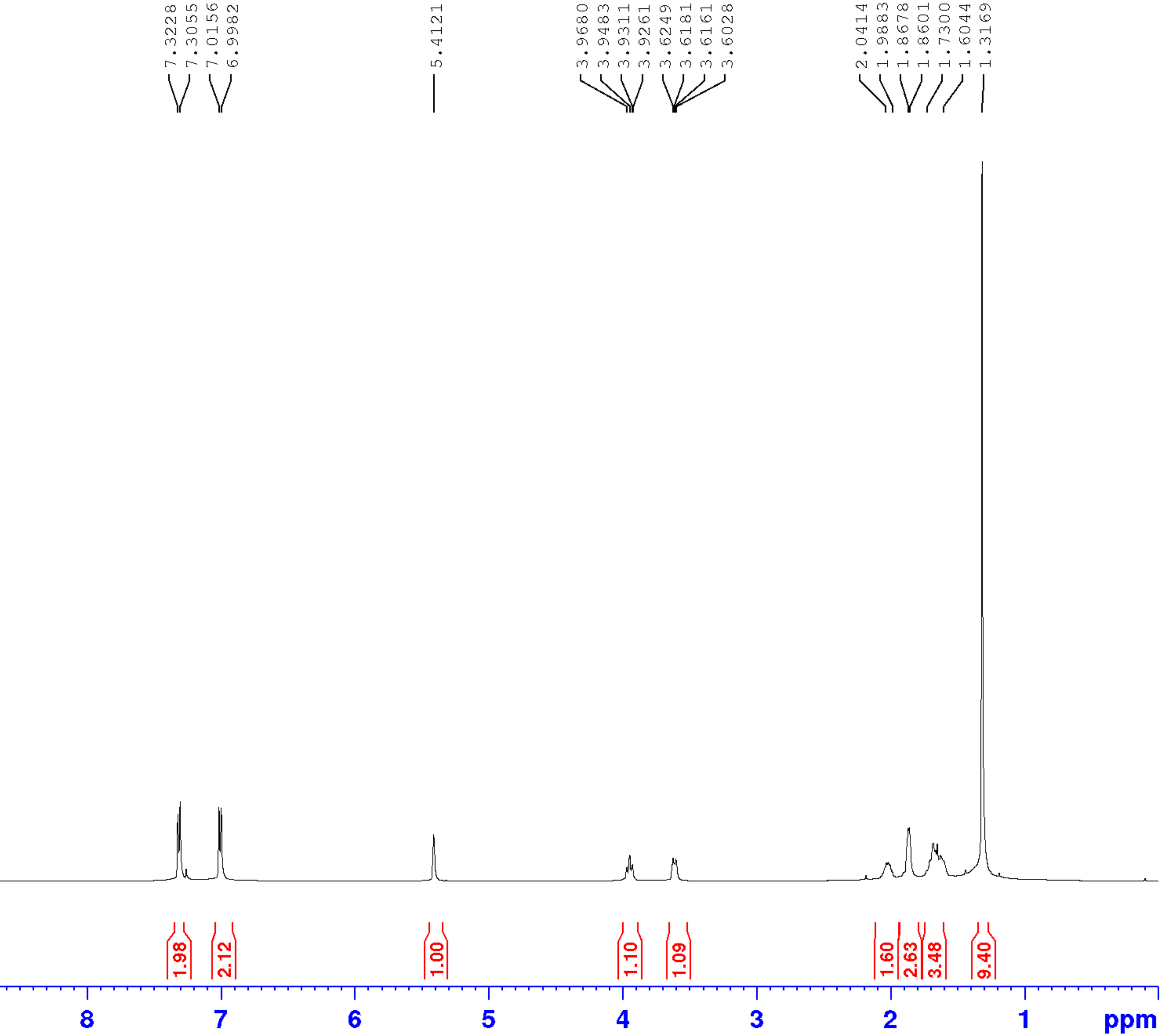


```
Current Data Parameters
NAME      vinn-4-139-8-islt2-2020012
EXPNO                    1
PROCNO                   1

F2 - Acquisition Parameters
Date_             20210121
Time                7.42 h
INSTRUM              spect
PROBHD    Z119470_0283 (
PULPROG               zg30
TD                   65536
SOLVENT              CDCl3
NS                      16
DS                       2
SWH          10000.000 Hz
FIDRES        0.305176 Hz
AQ           3.2767999 sec
RG                   30.85
DW              50.000 usec
DE               6.50 usec
TE               295.2 K
D1          1.00000000 sec
TD0                      1
SFO1       500.1330883 MHz
NUC1                    1H
P1               10.91 usec
PLW1       25.00000000 W

F2 - Processing parameters
SI                   65536
SF         500.1300131 MHz
WDW                     EM
SSB                      0
LB                 0.30 Hz
GB                       0
PC                    1.00
```

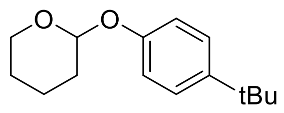

3o

125 MHz ^{13}C NMR

$CDCl_3$

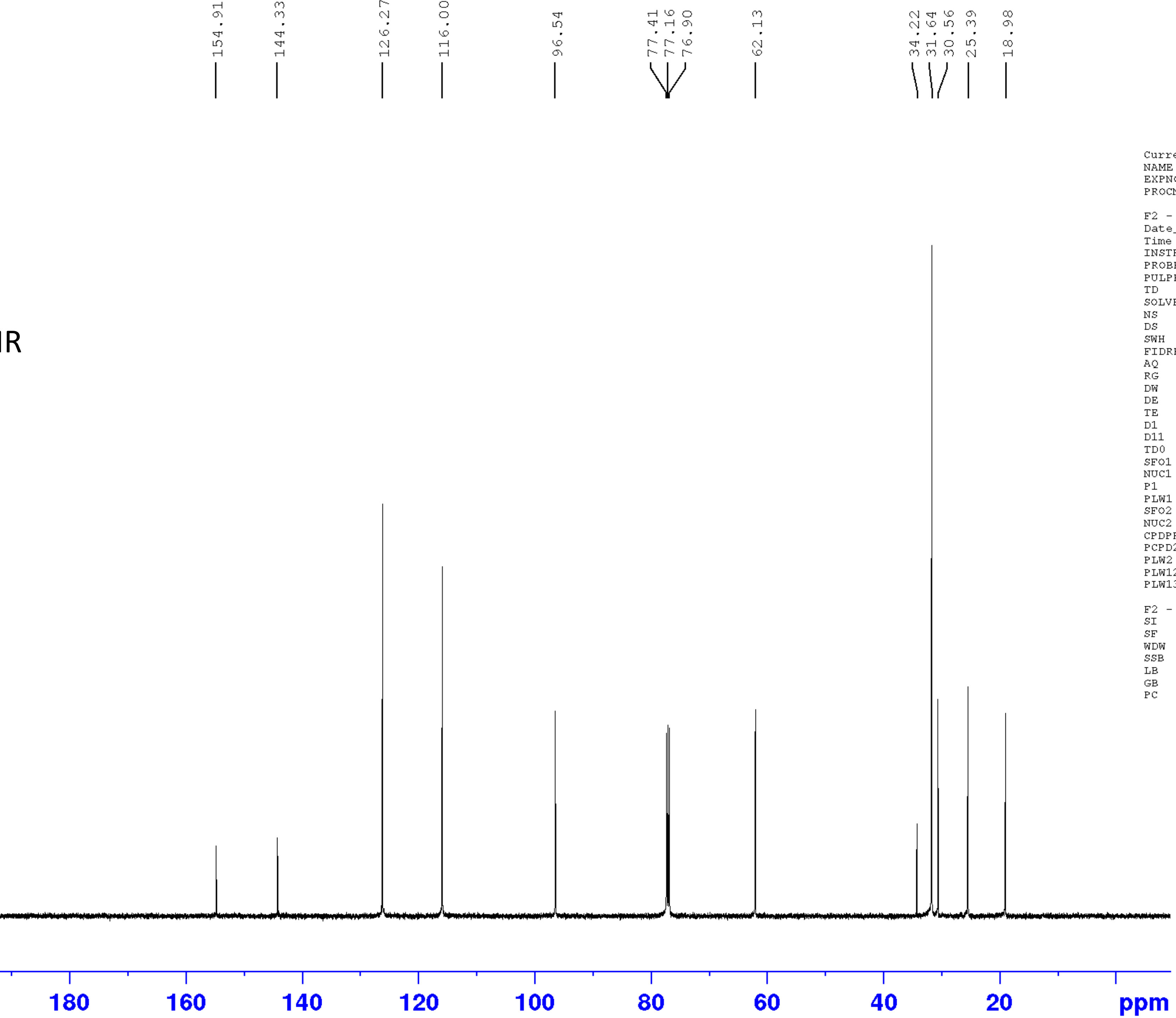


```
Current Data Parameters
NAME      vinn-4-139-8-islt2-20200120
EXPNO                    2
PROCNO                   1

F2 - Acquisition Parameters
Date_             20210121
Time                  7.56 h
INSTRUM              spect
PROBHD    Z119470_0283 (
PULPROG             zgpg30
TD                   65536
SOLVENT              CDCl3
NS                     256
DS                       4
SWH              29761.904 Hz
FIDRES            0.908261 Hz
AQ               1.1010048 sec
RG                  206.72
DW                  16.800 usec
DE                    6.50 usec
TE                   295.1 K
D1              2.00000000 sec
D11             0.03000000 sec
TD0                      1
SFO1           125.7703643 MHz
NUC1                   13C
P1                    9.75 usec
PLW1           94.00000000 W
SFO2           500.1320005 MHz
NUC2                    1H
CPDPRG[2           waltz16
PCPD2                80.00 usec
PLW2           25.00000000 W
PLW12           0.46495000 W
PLW13           0.23387000 W

F2 - Processing parameters
SI                   32768
SF             125.7577780 MHz
WDW                     EM
SSB                      0
LB                    1.00 Hz
GB                       0
PC                    1.40
```

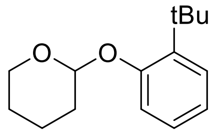

3p

400 MHz ^{1}H NMR

$CDCl_3$

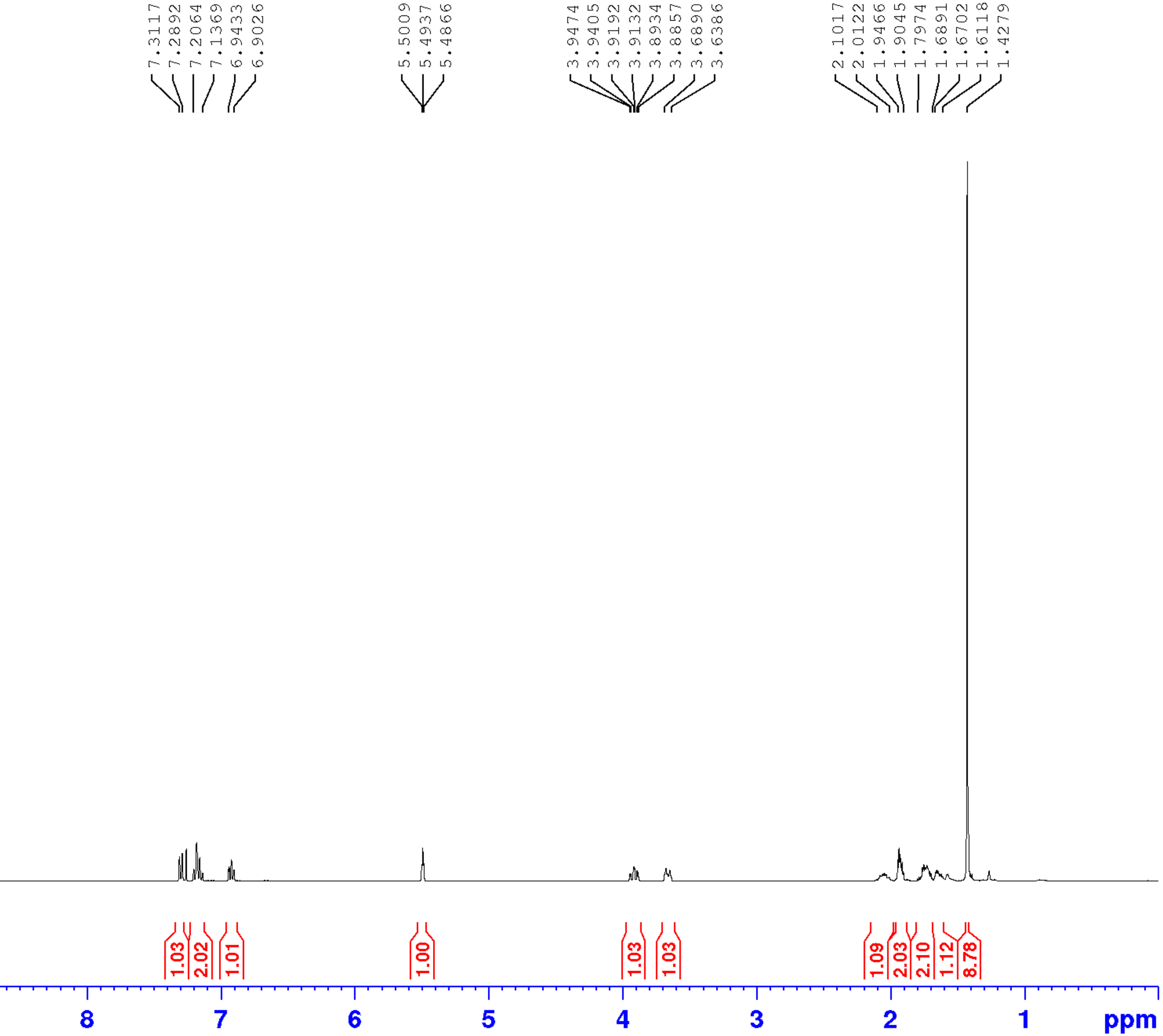

Current Data Parameters
NAME Andy-1-180-2-islt-20220818
EXPNO 2
PROCNO 1

F2 - Acquisition Parameters
Date_ 20220818
Time 14.40 h
INSTRUM spect
PROBHD Z108618_0257 (
PULPROG zg30
TD 65536
SOLVENT CDCl3
NS 16
DS 2
SWH 8012.820 Hz
FIDRES 0.244532 Hz
AQ 4.0894465 sec
RG 203
DW 62.400 usec
DE 6.50 usec
TE 295.9 K
D1 1.00000000 sec
TD0 1
SFO1 400.1324708 MHz
NUC1 1H
P1 15.00 usec
PLW1 12.50000000 W

F2 - Processing parameters
SI 65536
SF 400.1300098 MHz
WDW EM
SSB 0
LB 0.30 Hz
GB 0
PC 1.00

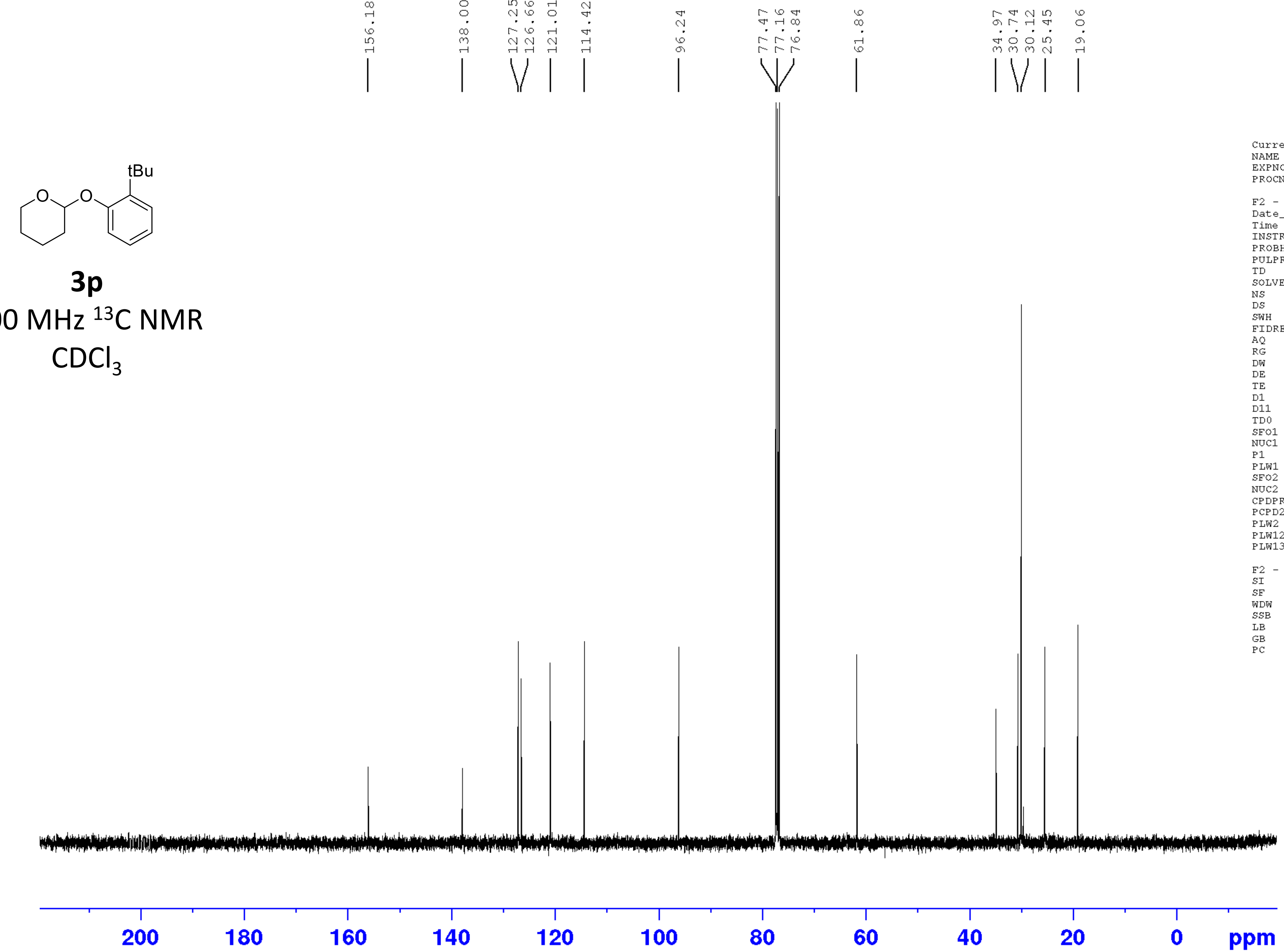

3p

100 MHz ^{13}C NMR

$CDCl_3$

```
Current Data Parameters
NAME      Andy-1-180-2-islt-20220818
EXPNO                      3
PROCNO                     1

F2 - Acquisition Parameters
Date_             20220818
Time                 14.54 h
INSTRUM              spect
PROBHD    Z108618_0257 (
PULPROG             zgpg30
TD                   65536
SOLVENT              CDCl3
NS                     200
DS                       4
SWH              24038.461 Hz
FIDRES            0.733596 Hz
AQ               1.3631488 sec
RG                     203
DW                  20.800 usec
DE                    6.50 usec
TE                   296.6 K
D1              2.00000000 sec
D11             0.03000000 sec
TD0                      1
SFO1           100.6228298 MHz
NUC1                   13C
P1                   10.00 usec
PLW1           51.00000000 W
SFO2           400.1316005 MHz
NUC2                    1H
CPDPRG[2           waltz16
PCPD2                90.00 usec
PLW2           12.50000000 W
PLW12           0.34722000 W
PLW13           0.17465000 W

F2 - Processing parameters
SI                   32768
SF             100.6127560 MHz
WDW                     EM
SSB                      0
LB                    1.00 Hz
GB                       0
PC                    1.40
```

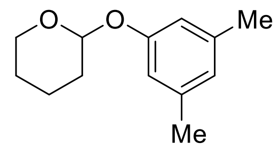

3q

500 MHz ^{1}H NMR

$CDCl_3$

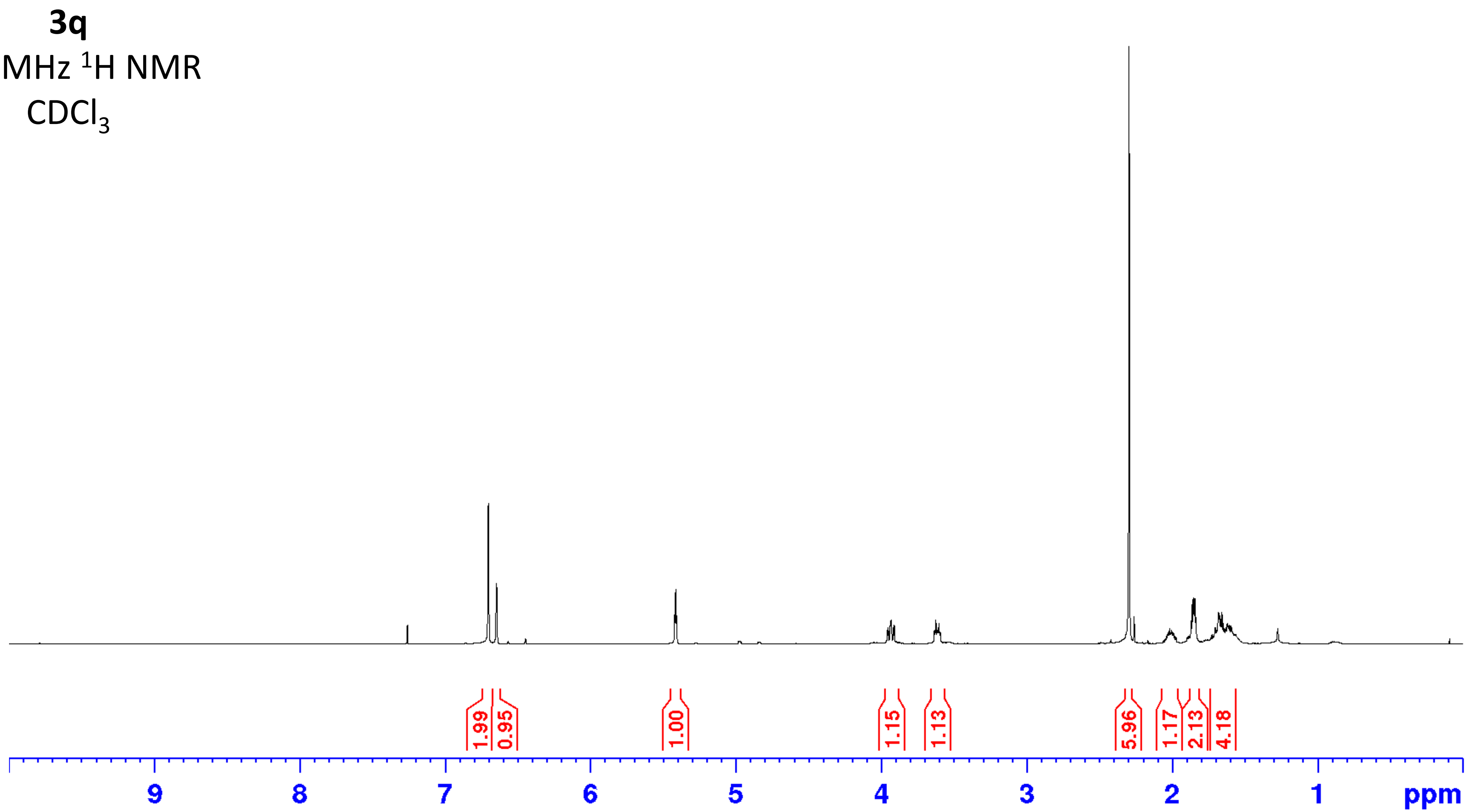


```
Current Data Parameters
NAME      vinn-4-139-9-islt-20210111
EXPNO                    1
PROCNO                   1

F2 - Acquisition Parameters
Date_             20210111
Time                 18.58 h
INSTRUM              spect
PROBHD    Z119470_0283 (
PULPROG               zg30
TD                   65536
SOLVENT              CDCl3
NS                      16
DS                       2
SWH              10000.000 Hz
FIDRES            0.305176 Hz
AQ               3.2767999 sec
RG                   30.85
DW                  50.000 usec
DE                    6.50 usec
TE                   295.1 K
D1              1.00000000 sec
TD0                      1
SFO1           500.1330883 MHz
NUC1                    1H
P1                   10.91 usec
PLW1           25.00000000 W

F2 - Processing parameters
SI                   65536
SF             500.1300125 MHz
WDW                     EM
SSB                      0
LB                    0.30 Hz
GB                       0
PC                    1.00
```

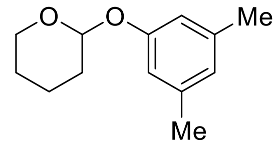

3q

125 MHz ^{13}C NMR

$CDCl_3$

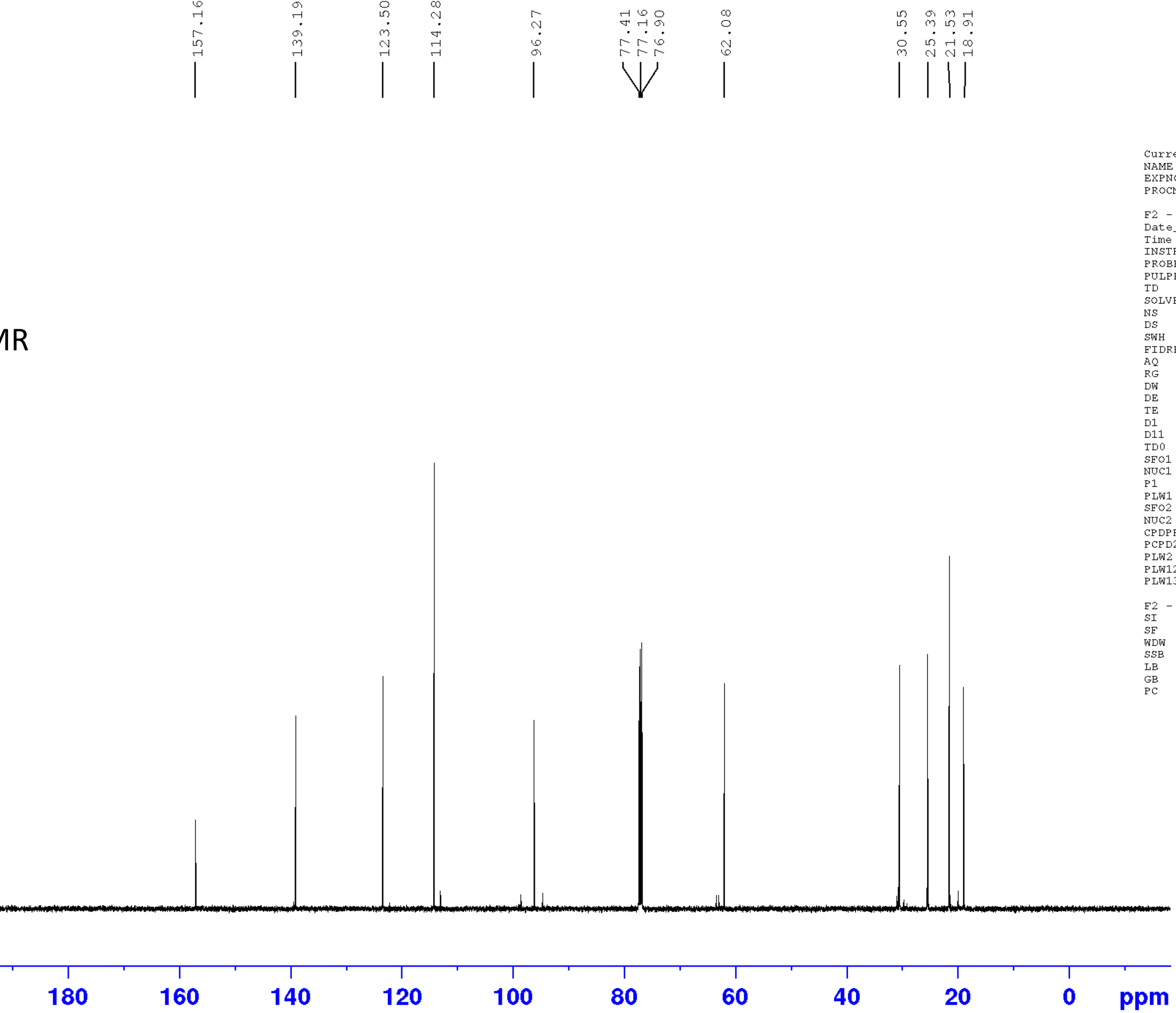

Current Data Parameters
NAME vinn-4-139-9-islt-20210111
EXPNO 2
PROCNO 1

F2 - Acquisition Parameters
Date_ 20210111
Time 19.03 h
INSTRUM spect
PROBHD Z119470_0283 (
PULPROG zgpg30
TD 65536
SOLVENT CDCl3
NS 100
DS 4
SWH 29761.904 Hz
FIDRES 0.908261 Hz
AQ 1.1010048 sec
RG 206.72
DW 16.800 usec
DE 6.50 usec
TE 295.1 K
D1 2.00000000 sec
D11 0.03000000 sec
TD0 1
SFO1 125.7703643 MHz
NUC1 13C
P1 9.75 usec
PLW1 94.00000000 W
SFO2 500.1320005 MHz
NUC2 1H
CPDPRG[2 waltz16
PCPD2 80.00 usec
PLW2 25.00000000 W
PLW12 0.46495000 W
PLW13 0.23387000 W

F2 - Processing parameters
SI 32768
SF 125.7577784 MHz
WDW EM
SSB 0
LB 1.00 Hz
GB 0
PC 1.40

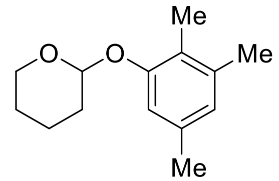

3r

500 MHz ^{1}H NMR

$CDCl_3$

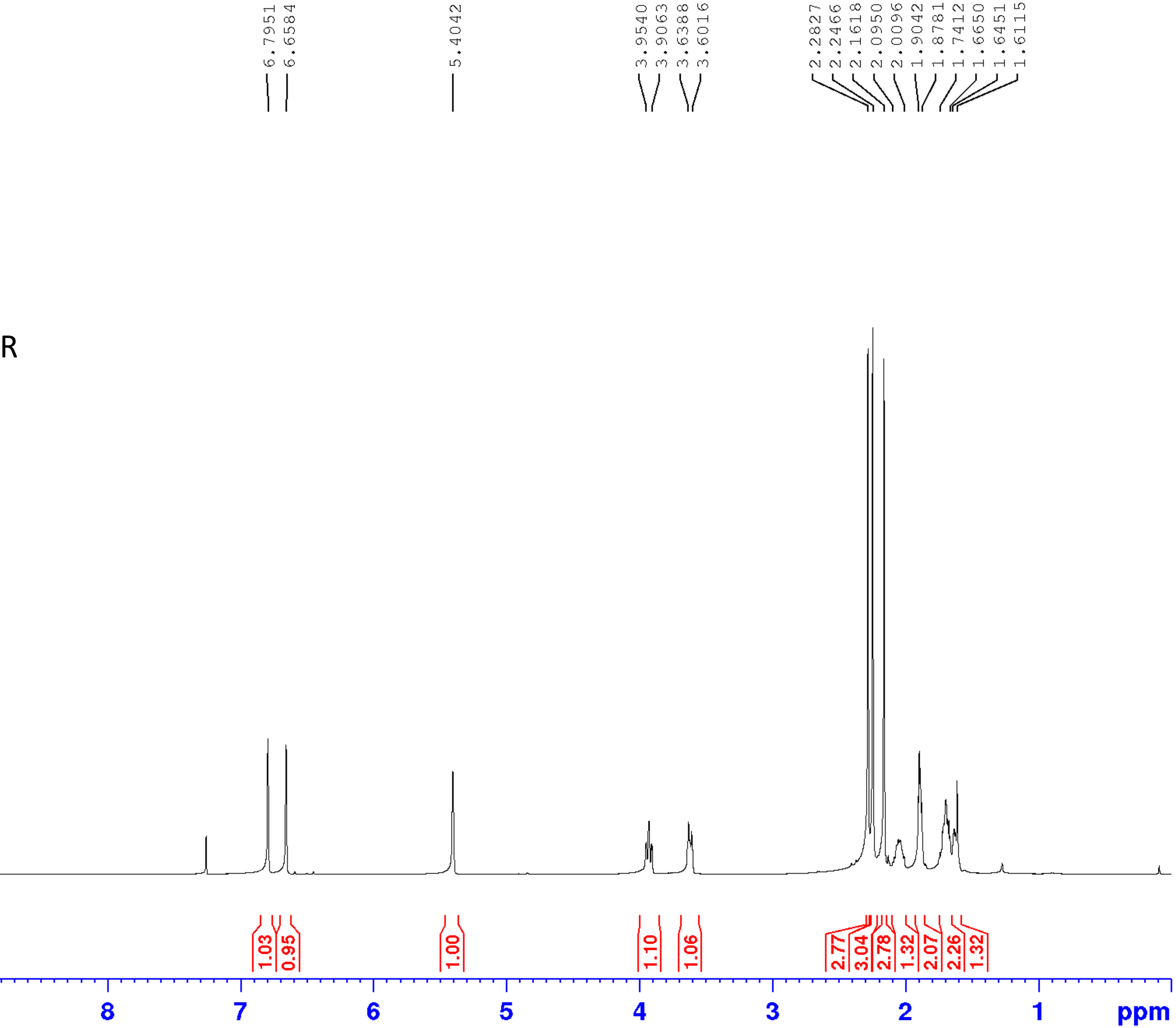

BRUKER

```
Current Data Parameters
NAME      vinn-4-139-10-islt2-20200120
EXPNO                1
PROCNO               1

F2 - Acquisition Parameters
Date_          20210121
Time               8.02 h
INSTRUM           spect
PROBHD   Z119470_0283 (
PULPROG            zg30
TD                65536
SOLVENT           CDCl3
NS                   16
DS                    2
SWH           10000.000 Hz
FIDRES         0.305176 Hz
AQ            3.2767999 sec
RG                 50.6
DW               50.000 usec
DE                 6.50 usec
TE                295.1 K
D1           1.00000000 sec
TD0                   1
SFO1        500.1330883 MHz
NUC1                 1H
P1                10.91 usec
PLW1        25.00000000 W

F2 - Processing parameters
SI                65536
SF          500.1300128 MHz
WDW                  EM
SSB                   0
LB                 0.30 Hz
GB                    0
PC                 1.00
```

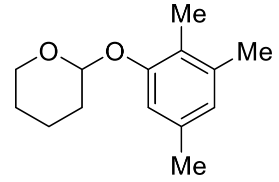

3r

125 MHz ^{13}C NMR

$CDCl_3$

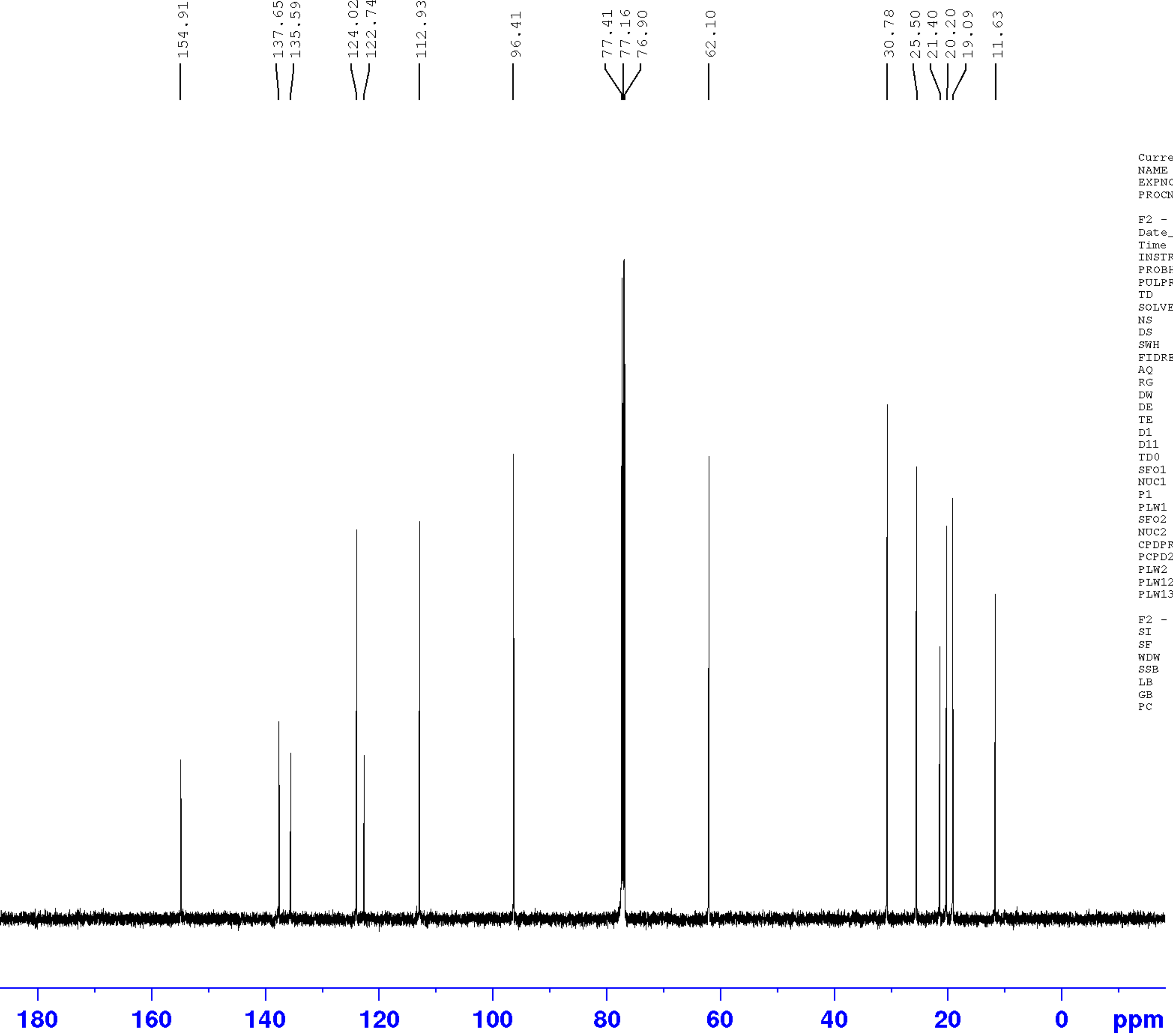


```
Current Data Parameters
NAME        vinn-4-139-10-islt2-2020012(
EXPNO                      2
PROCNO                     1

F2 - Acquisition Parameters
Date_              20210121
Time                   8.16 h
INSTRUM               spect
PROBHD    Z119470_0283 (
PULPROG              zgpg30
TD                    65536
SOLVENT               CDCl3
NS                      256
DS                        4
SWH             29761.904 Hz
FIDRES           0.908261 Hz
AQ              1.1010048 sec
RG                  206.72
DW                  16.800 usec
DE                    6.50 usec
TE                   295.2 K
D1             2.00000000 sec
D11            0.03000000 sec
TD0                       1
SFO1          125.7703643 MHz
NUC1                    13C
P1                     9.75 usec
PLW1          94.00000000 W
SFO2          500.1320005 MHz
NUC2                     1H
CPDPRG[2            waltz16
PCPD2                 80.00 usec
PLW2          25.00000000 W
PLW12          0.46495000 W
PLW13          0.23387000 W

F2 - Processing parameters
SI                    32768
SF            125.7577766 MHz
WDW                      EM
SSB                       0
LB                     1.00 Hz
GB                        0
PC                     1.40
```

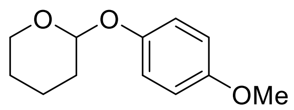

3s

500 MHz ^{1}H NMR

$CDCl_3$

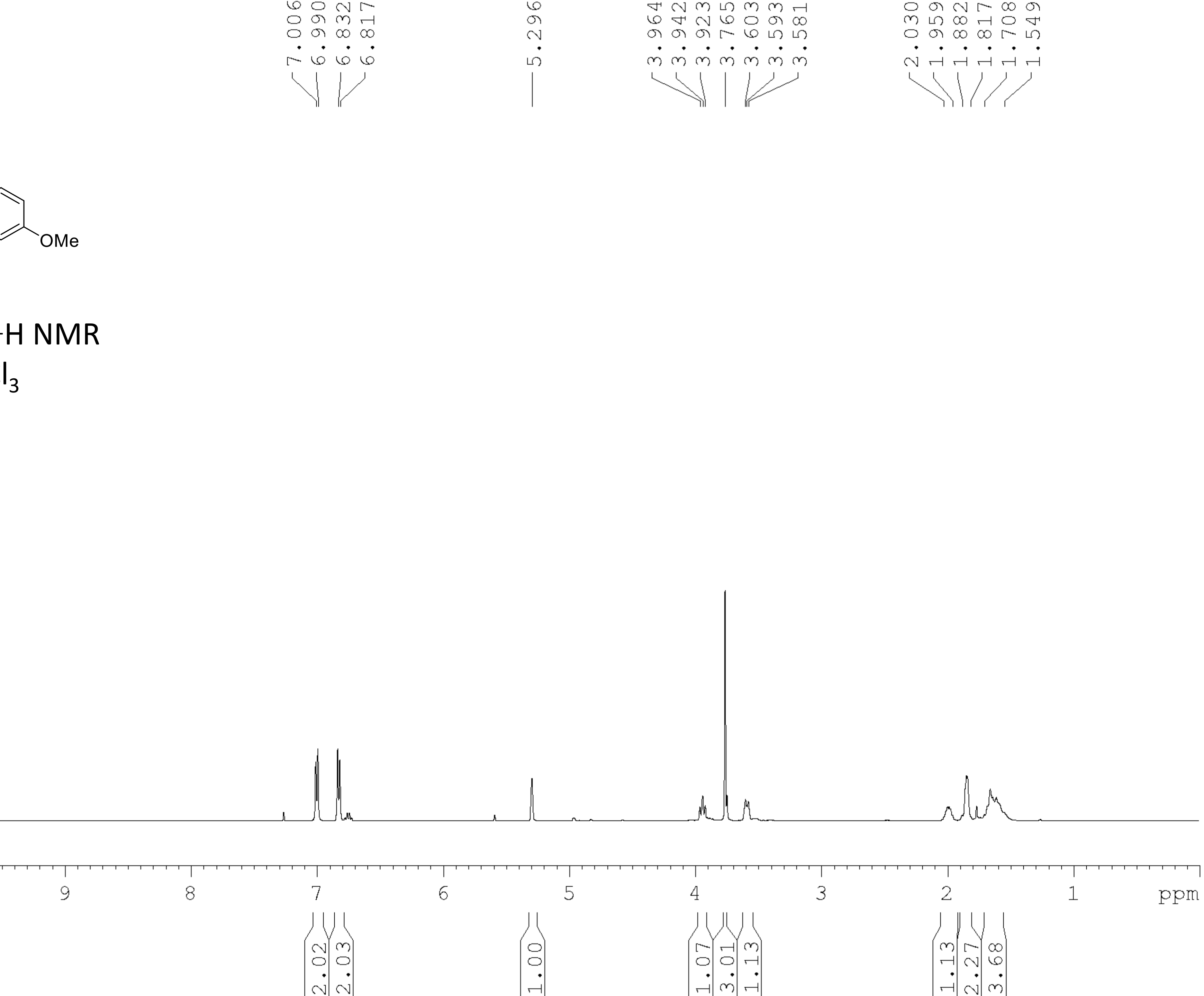


```
Current Data Parameters
NAME      vinn-4-139-5-2-islt-20201216
EXPNO                  1
PROCNO                 1

F2 - Acquisition Parameters
Date_           20201216
Time               19.13 h
INSTRUM            spect
PROBHD   Z119470_0283 (
PULPROG             zg30
TD                 65536
SOLVENT            CDCl3
NS                    16
DS                     2
SWH            10000.000 Hz
FIDRES          0.305176 Hz
AQ             3.2767999 sec
RG                 30.85
DW                50.000 usec
DE                  6.50 usec
TE                 295.1 K
D1            1.00000000 sec
TD0                    1
SFO1         500.1330883 MHz
NUC1                  1H
P1                 10.91 usec
PLW1         25.00000000 W

F2 - Processing parameters
SI                 65536
SF           500.1300121 MHz
WDW                   EM
SSB                    0
LB                  0.30 Hz
GB                     0
PC                  1.00
```

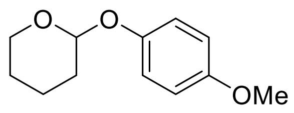

3s
125 MHz ^{13}C NMR
$CDCl_3$

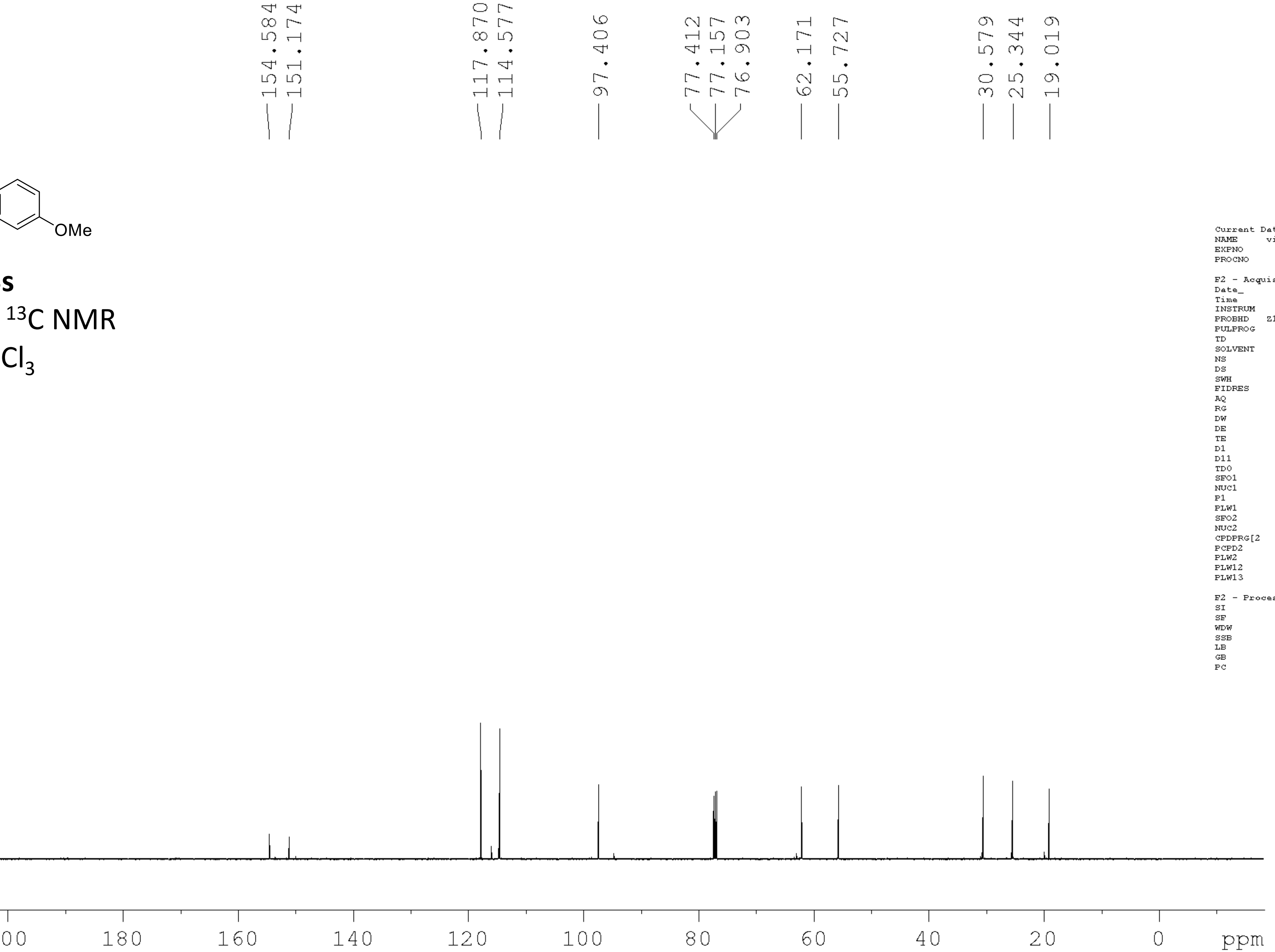

Current Data Parameters
NAME vinn-4-139-5-2-islt-20201216
EXPNO 2
PROCNO 1

F2 - Acquisition Parameters
Date_ 20201216
Time 19.18 h
INSTRUM spect
PROBHD Z119470_0283 (
PULPROG zgpg30
TD 65536
SOLVENT CDCl3
NS 100
DS 4
SWH 29761.904 Hz
FIDRES 0.908261 Hz
AQ 1.1010048 sec
RG 206.72
DW 16.800 usec
DE 6.50 usec
TE 295.2 K
D1 2.00000000 sec
D11 0.03000000 sec
TD0 1
SFO1 125.7703643 MHz
NUC1 13C
P1 9.75 usec
PLW1 94.00000000 W
SFO2 500.1320005 MHz
NUC2 1H
CPDPRG[2 waltz16
PCPD2 80.00 usec
PLW2 25.00000000 W
PLW12 0.46495000 W
PLW13 0.23387000 W

F2 - Processing parameters
SI 32768
SF 125.7577799 MHz
WDW EM
SSB 0
LB 1.00 Hz
GB 0
PC 1.40

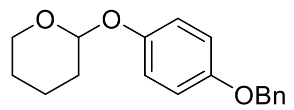

3t

500 MHz ^{1}H NMR

$CDCl_3$

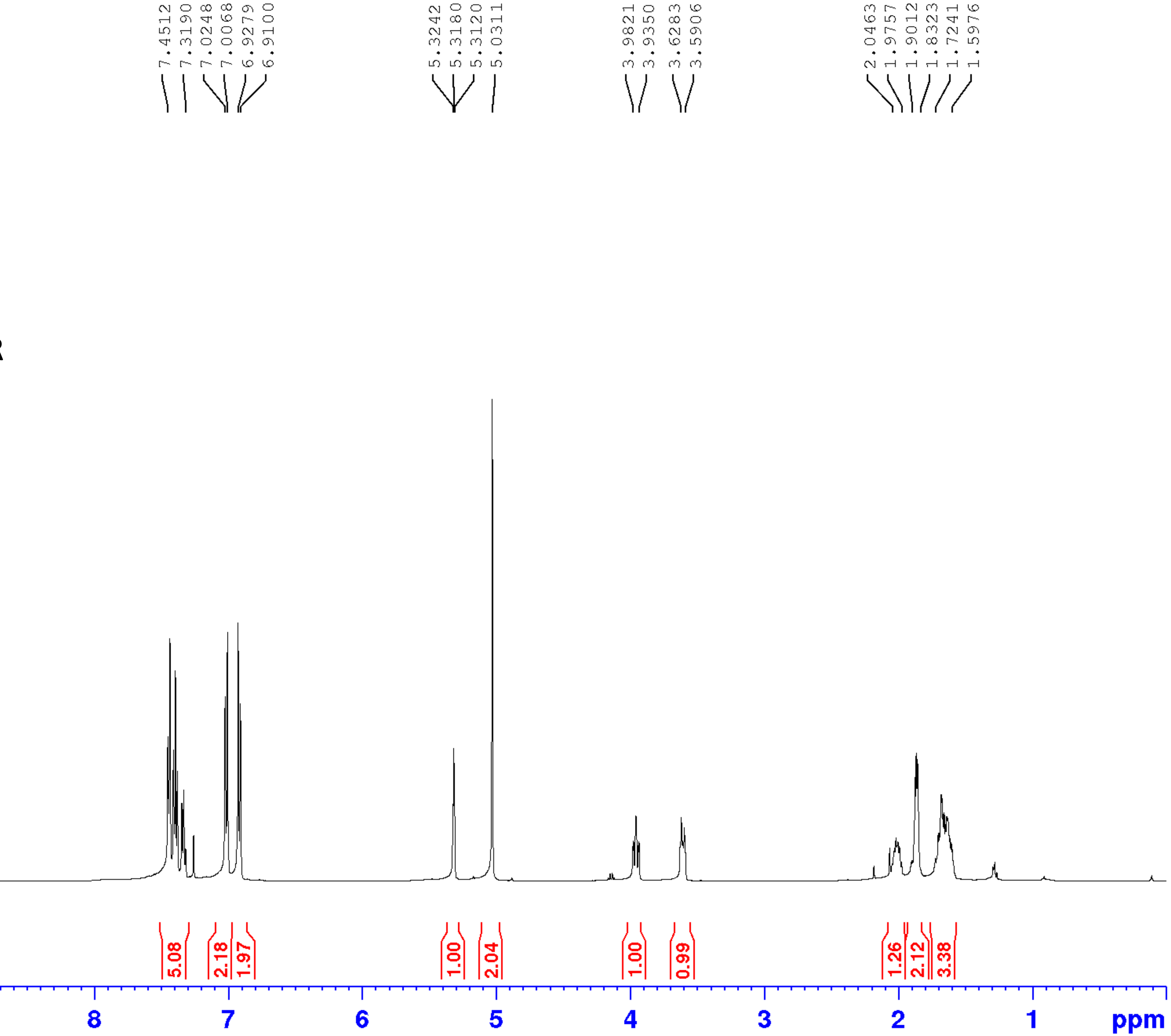

Current Data Parameters
NAME vinn-4-139-6-2-islt2-20200120
EXPNO 1
PROCNO 1

F2 - Acquisition Parameters
Date_ 20210121
Time 9.35 h
INSTRUM spect
PROBHD Z119470_0283 (
PULPROG zg30
TD 65536
SOLVENT CDCl3
NS 16
DS 2
SWH 10000.000 Hz
FIDRES 0.305176 Hz
AQ 3.2767999 sec
RG 30.85
DW 50.000 usec
DE 6.50 usec
TE 295.1 K
D1 1.00000000 sec
TD0 1
SFO1 500.1330883 MHz
NUC1 1H
P1 10.91 usec
PLW1 25.00000000 W

F2 - Processing parameters
SI 65536
SF 500.1300127 MHz
WDW EM
SSB 0
LB 0.30 Hz
GB 0
PC 1.00

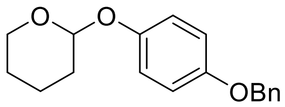

3t
125 MHz ^{13}C NMR
$CDCl_3$

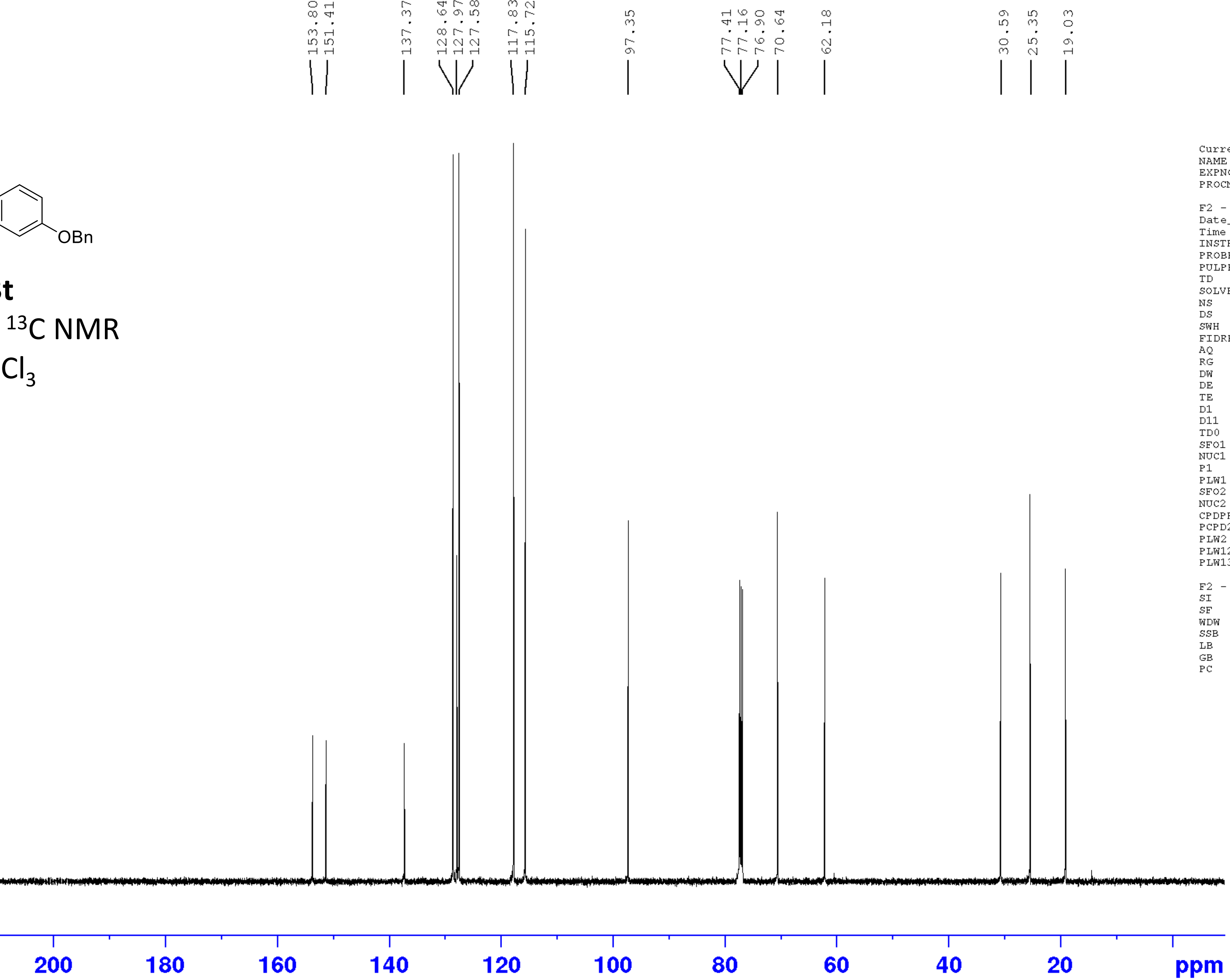

Current Data Parameters
NAME vinn-4-139-6-2-islt2-2020012
EXPNO 2
PROCNO 1

F2 - Acquisition Parameters
Date_ 20210121
Time 9.50 h
INSTRUM spect
PROBHD Z119470_0283 (
PULPROG zgpg30
TD 65536
SOLVENT CDCl3
NS 256
DS 4
SWH 29761.904 Hz
FIDRES 0.908261 Hz
AQ 1.1010048 sec
RG 206.72
DW 16.800 usec
DE 6.50 usec
TE 295.2 K
D1 2.00000000 sec
D11 0.03000000 sec
TD0 1
SFO1 125.7703643 MHz
NUC1 13C
P1 9.75 usec
PLW1 94.00000000 W
SFO2 500.1320005 MHz
NUC2 1H
CPDPRG[2 waltz16
PCPD2 80.00 usec
PLW2 25.00000000 W
PLW12 0.46495000 W
PLW13 0.23387000 W

F2 - Processing parameters
SI 32768
SF 125.7577812 MHz
WDW EM
SSB 0
LB 1.00 Hz
GB 0
PC 1.40

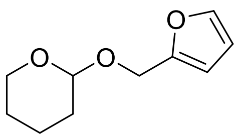

3u

500 MHz ^{1}H NMR

$CDCl_3$

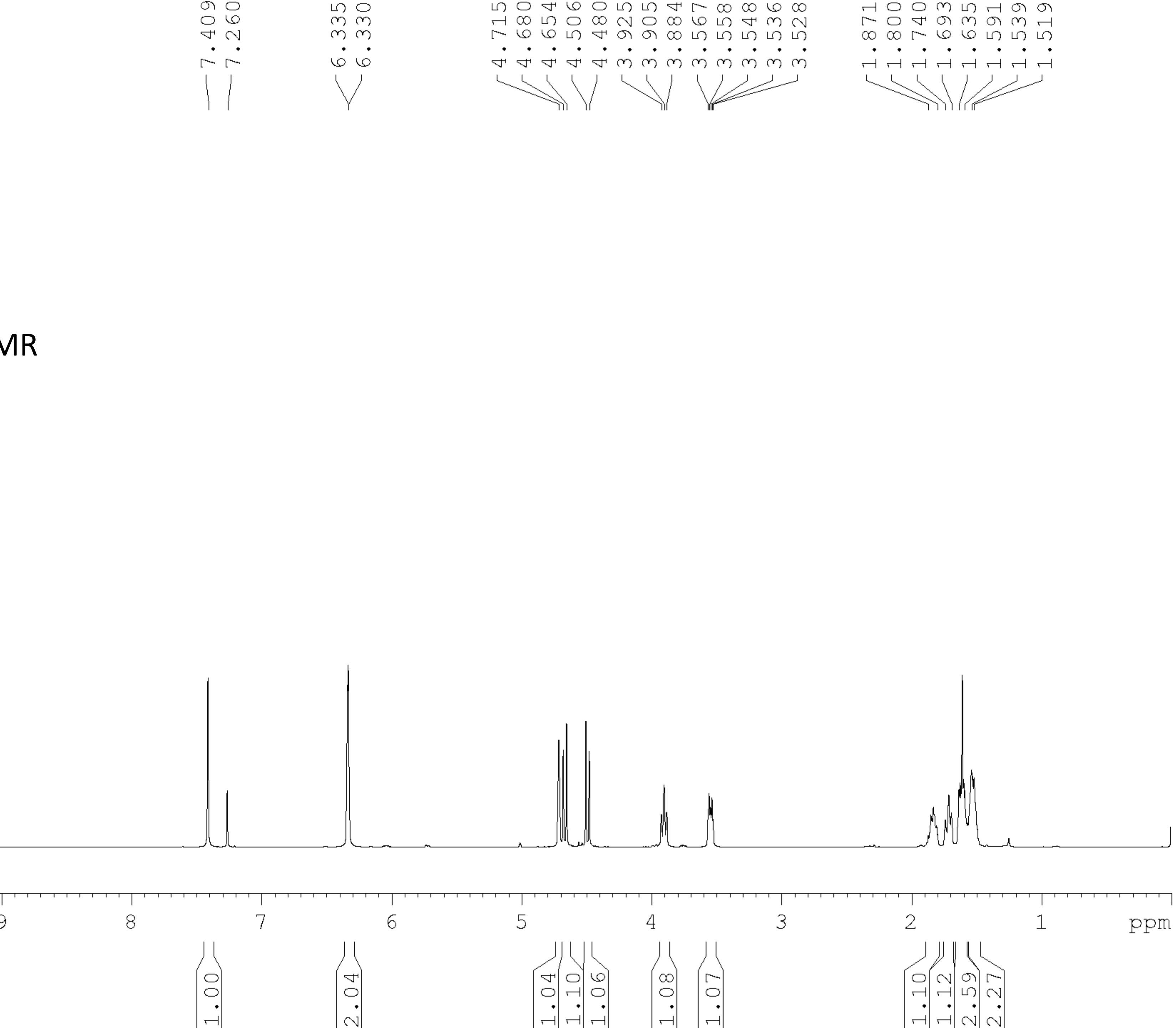


```
Current Data Parameters
NAME     vinn-7-105-1-islt-20230807
EXPNO                  1
PROCNO                 1

F2 - Acquisition Parameters
Date_           20230807
Time               17.34 h
INSTRUM            spect
PROBHD   Z149001_0010 (
PULPROG             zg30
TD                 65536
SOLVENT            CDCl3
NS                    16
DS                     2
SWH            10000.000 Hz
FIDRES          0.305176 Hz
AQ             3.2767999 sec
RG                 30.85
DW                50.000 usec
DE                 10.00 usec
TE                 296.2 K
D1            1.00000000 sec
TD0                    1
SFO1         500.1330883 MHz
NUC1                  1H
P1                 11.25 usec
PLW1         17.35199928 W

F2 - Processing parameters
SI                 65536
SF           500.1300130 MHz
WDW                   EM
SSB                    0
LB                  0.30 Hz
GB                     0
PC                  1.00
```

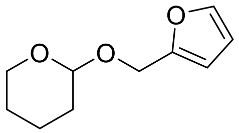

3u

125 MHz ^{13}C NMR

$CDCl_3$

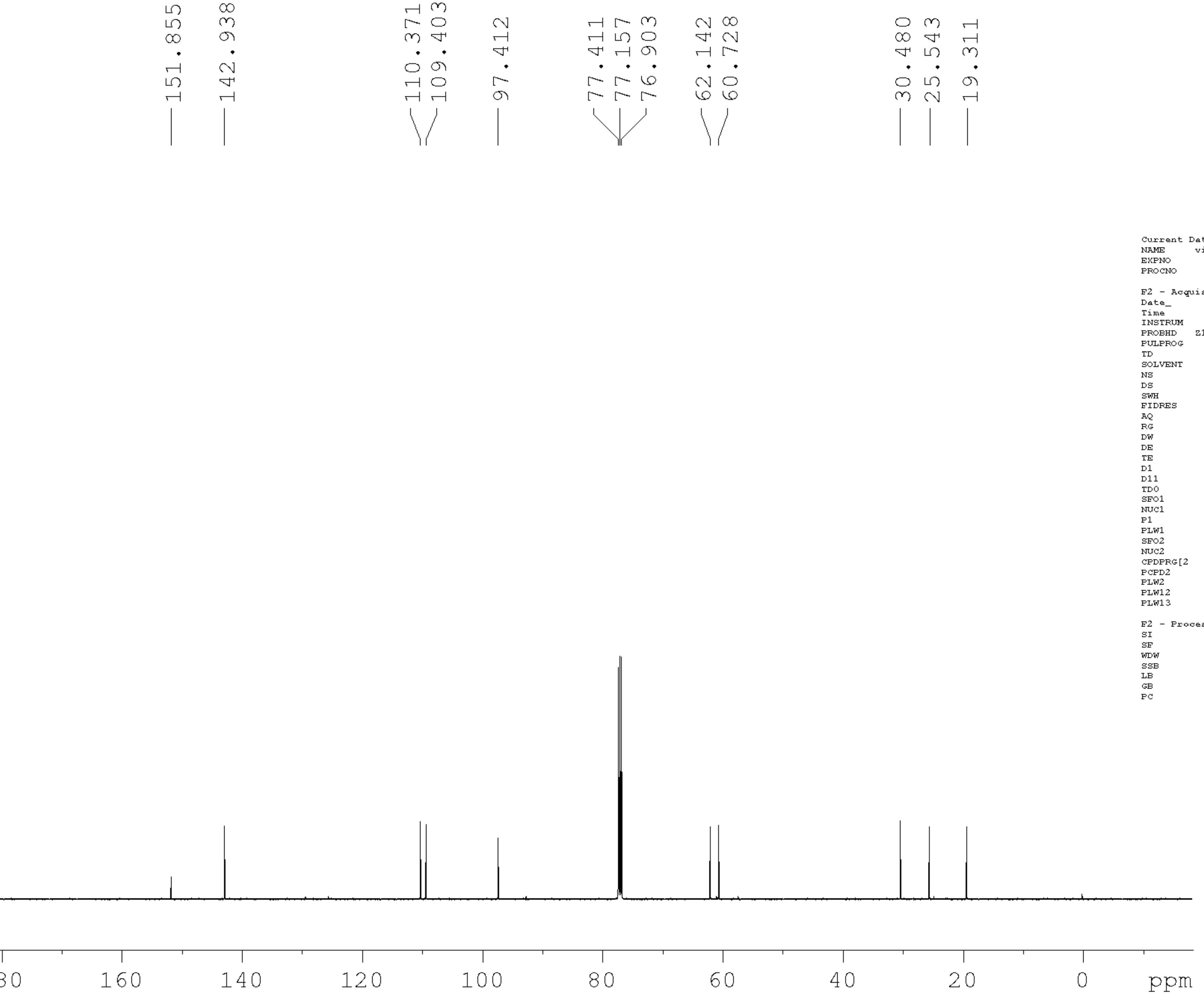


```
Current Data Parameters
NAME      vinn-7-105-1-islt-20230807
EXPNO                     2
PROCNO                    1

F2 - Acquisition Parameters
Date_              20230807
Time                17.46 h
INSTRUM               spect
PROBHD   Z149001_0010 (
PULPROG             zgpg30
TD                   65536
SOLVENT              CDCl3
NS                     200
DS                       4
SWH              29761.904 Hz
FIDRES            0.908261 Hz
AQ               1.1010048 sec
RG                  206.72
DW                  16.800 usec
DE                   18.00 usec
TE                   296.1 K
D1              2.00000000 sec
D11             0.03000000 sec
TD0                      1
SFO1           125.7703643 MHz
NUC1                   13C
P1                   10.00 usec
PLW1           61.00000000 W
SFO2           500.1320005 MHz
NUC2                    1H
CPDPRG[2           waltz16
PCPD2                80.00 usec
PLW2           17.35199928 W
PLW12           0.34314999 W
PLW13           0.17260000 W

F2 - Processing parameters
SI                   32768
SF             125.7577735 MHz
WDW                     EM
SSB                      0
LB                    1.00 Hz
GB                       0
PC                    1.40
```

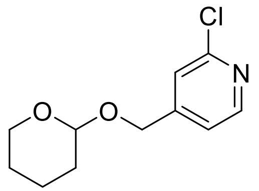

3v

400 MHz ^{1}H NMR

$CDCl_3$

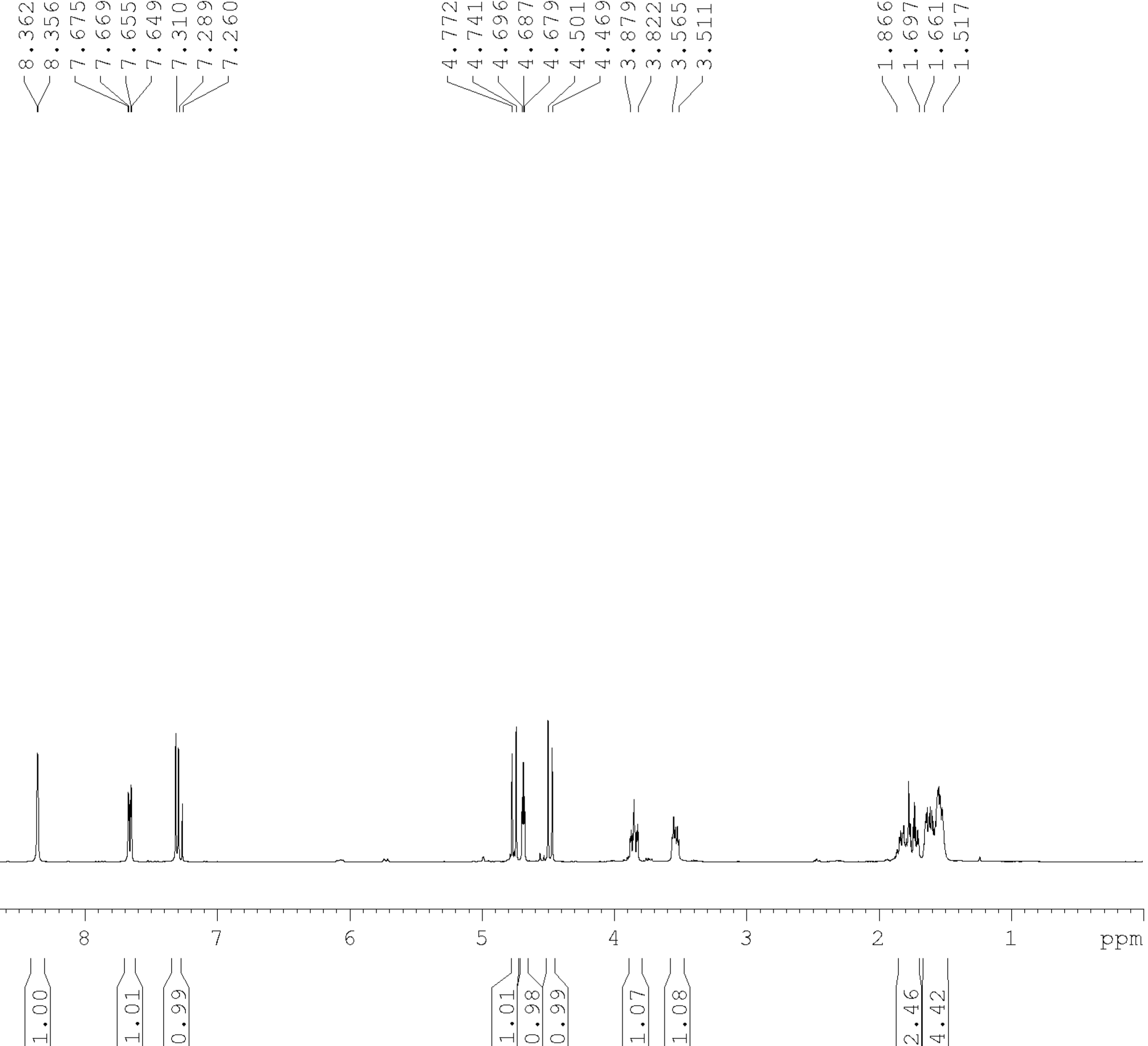


```
Current Data Parameters
NAME     vinn-7-110-1-islt-20230810
EXPNO                     1
PROCNO                    1

F2 - Acquisition Parameters
Date_             20230810
Time                 17.08 h
INSTRUM              spect
PROBHD   Z108618_0257 (
PULPROG               zg30
TD                   65536
SOLVENT              CDCl3
NS                      16
DS                       2
SWH              8012.820 Hz
FIDRES           0.244532 Hz
AQ              4.0894465 sec
RG                     101
DW                  62.400 usec
DE                    6.50 usec
TE                   295.8 K
D1             1.00000000 sec
TD0                      1
SFO1          400.1324708 MHz
NUC1                    1H
P1                   15.00 usec
PLW1          12.50000000 W

F2 - Processing parameters
SI                   65536
SF            400.1300097 MHz
WDW                     EM
SSB                      0
LB                    0.30 Hz
GB                       0
PC                    1.00
```

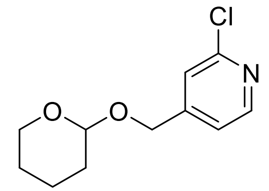

3v

100 MHz ^{13}C NMR

$CDCl_3$

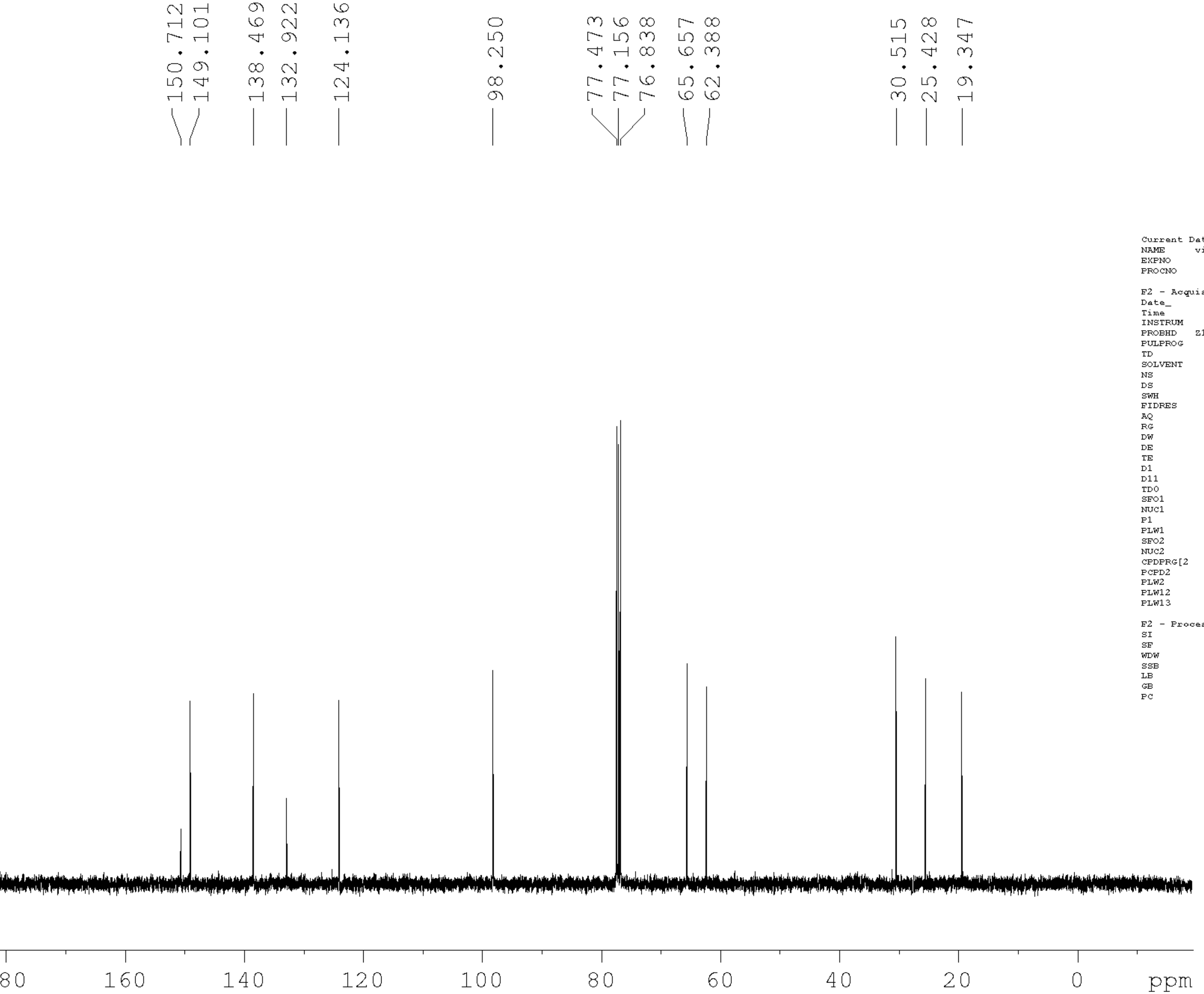


```
Current Data Parameters
NAME      vinn-7-110-1-islt-20230810
EXPNO                  2
PROCNO                 1

F2 - Acquisition Parameters
Date_           20230810
Time               17.12 h
INSTRUM            spect
PROBHD   Z108618_0257 (
PULPROG          zgpg30
TD                65536
SOLVENT           CDCl3
NS                   40
DS                    4
SWH           24038.461 Hz
FIDRES         0.733596 Hz
AQ            1.3631488 sec
RG                  203
DW               20.800 usec
DE                 6.50 usec
TE                296.3 K
D1           2.00000000 sec
D11          0.03000000 sec
TD0                   1
SFO1        100.6228298 MHz
NUC1                13C
P1                10.00 usec
PLW1        51.00000000 W
SFO2        400.1316005 MHz
NUC2                 1H
CPDPRG[2          waltz16
PCPD2             90.00 usec
PLW2        12.50000000 W
PLW12        0.34722000 W
PLW13        0.17465000 W

F2 - Processing parameters
SI                32768
SF          100.6127586 MHz
WDW                  EM
SSB                   0
LB                 1.00 Hz
GB                    0
PC                 1.40
```

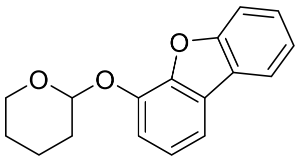

3w

400 MHz ^{1}H NMR

$CDCl_3$

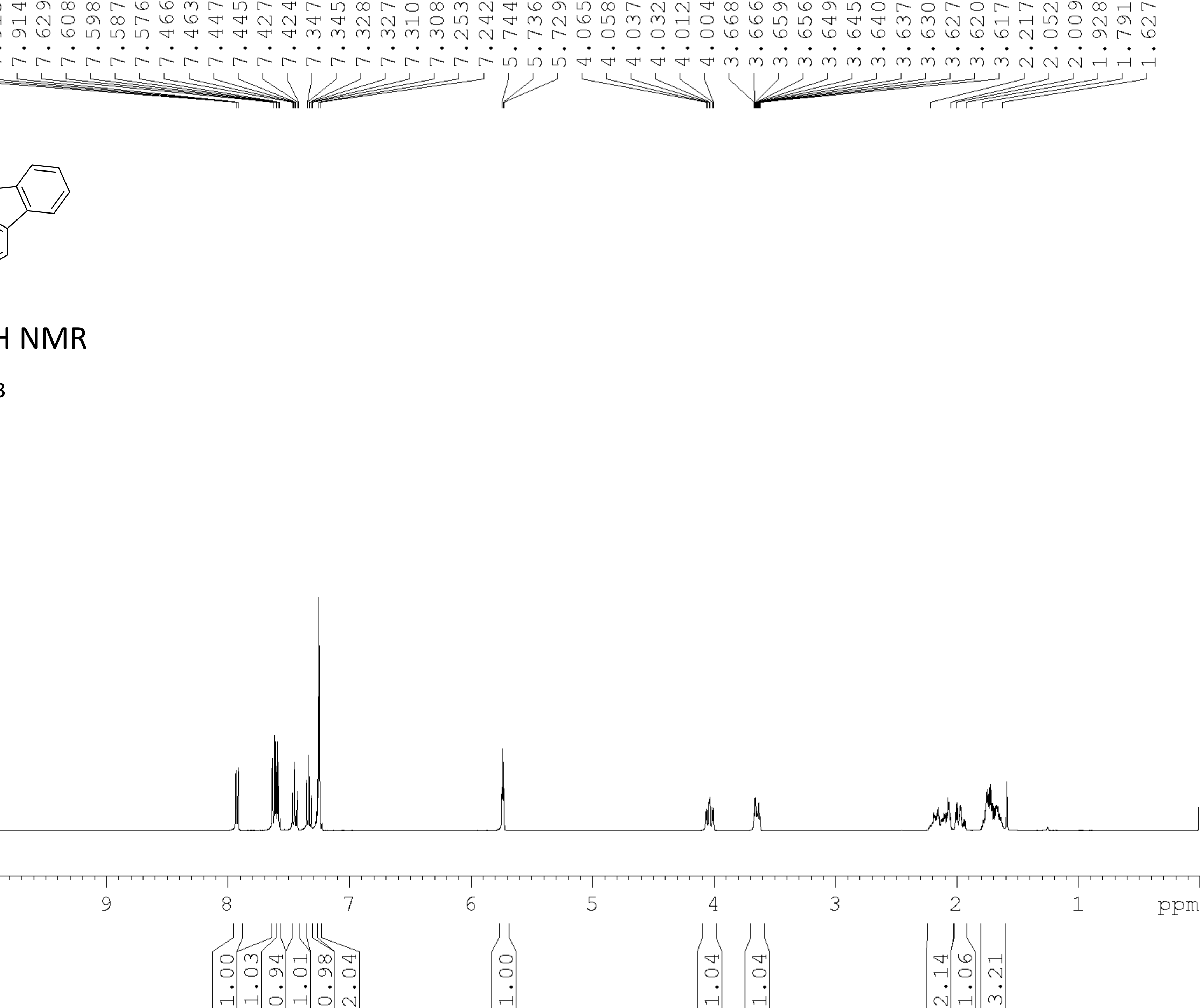


```
Current Data Parameters
NAME     vinn-7-110-3-islt-20230810
EXPNO                 1
PROCNO                1

F2 - Acquisition Parameters
Date_          20230810
Time              17.17 h
INSTRUM           spect
PROBHD   Z108618_0257 (
PULPROG            zg30
TD                65536
SOLVENT           CDCl3
NS                   16
DS                    2
SWH            8012.820 Hz
FIDRES         0.244532 Hz
AQ            4.0894465 sec
RG                 90.5
DW               62.400 usec
DE                 6.50 usec
TE                295.8 K
D1           1.00000000 sec
TD0                   1
SFO1        400.1324708 MHz
NUC1                 1H
P1                15.00 usec
PLW1        12.50000000 W

F2 - Processing parameters
SI                65536
SF          400.1300168 MHz
WDW                  EM
SSB                   0
LB                 0.30 Hz
GB                    0
PC                 1.00
```

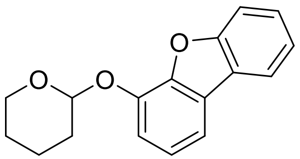

3w

100 MHz ^{13}C NMR

$CDCl_3$

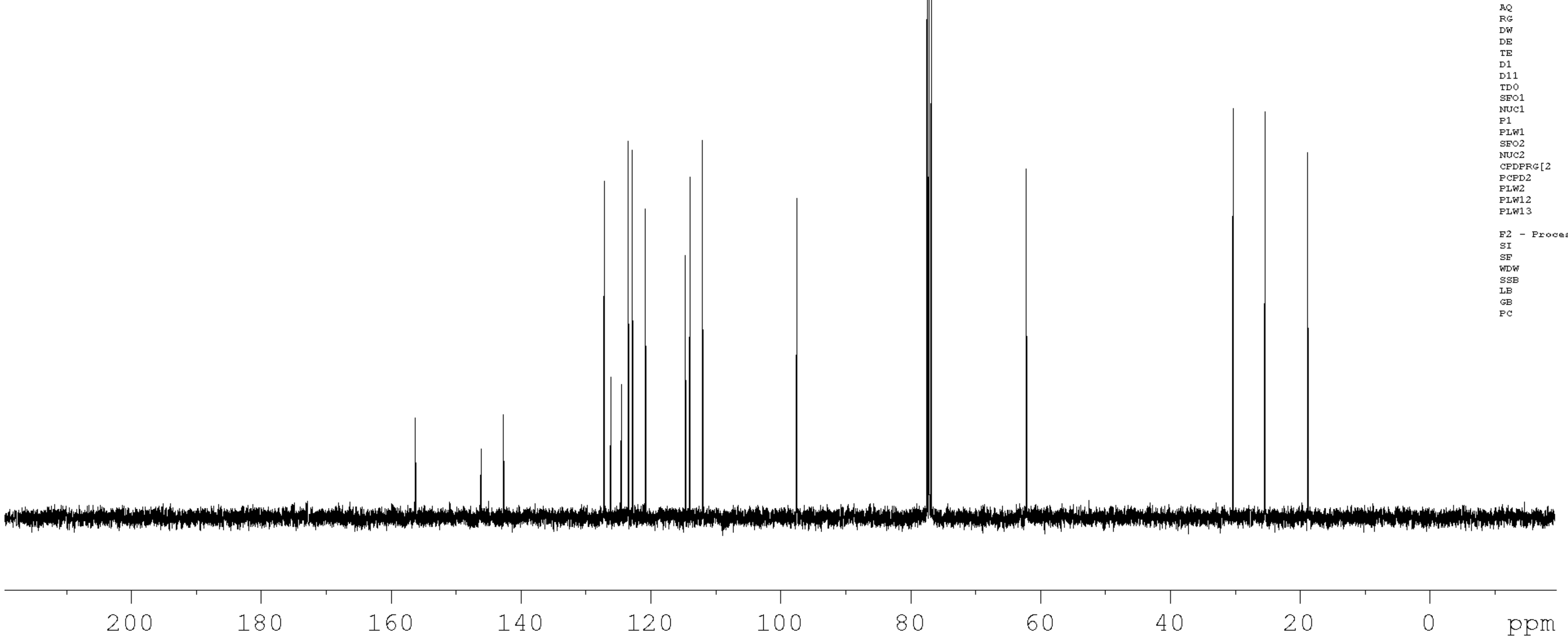


```
Current Data Parameters
NAME     vinn-7-110-3-islt-20230810
EXPNO                     2
PROCNO                    1

F2 - Acquisition Parameters
Date_              20230810
Time                  17.22 h
INSTRUM               spect
PROBHD   Z108618_0257 (
PULPROG             zgpg30
TD                    65536
SOLVENT               CDCl3
NS                       60
DS                        4
SWH               24038.461 Hz
FIDRES             0.733596 Hz
AQ                1.3631488 sec
RG                      203
DW                   20.800 usec
DE                     6.50 usec
TE                    296.1 K
D1               2.00000000 sec
D11              0.03000000 sec
TD0                       1
SFO1            100.6228298 MHz
NUC1                    13C
P1                    10.00 usec
PLW1            51.00000000 W
SFO2            400.1316005 MHz
NUC2                     1H
CPDPRG[2            waltz16
PCPD2                 90.00 usec
PLW2            12.50000000 W
PLW12            0.34722000 W
PLW13            0.17465000 W

F2 - Processing parameters
SI                    32768
SF              100.6127596 MHz
WDW                      EM
SSB                       0
LB                     1.00 Hz
GB                        0
PC                     1.40
```

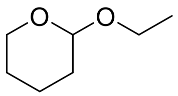

3x

400 MHz ^{1}H NMR

$CDCl_3$

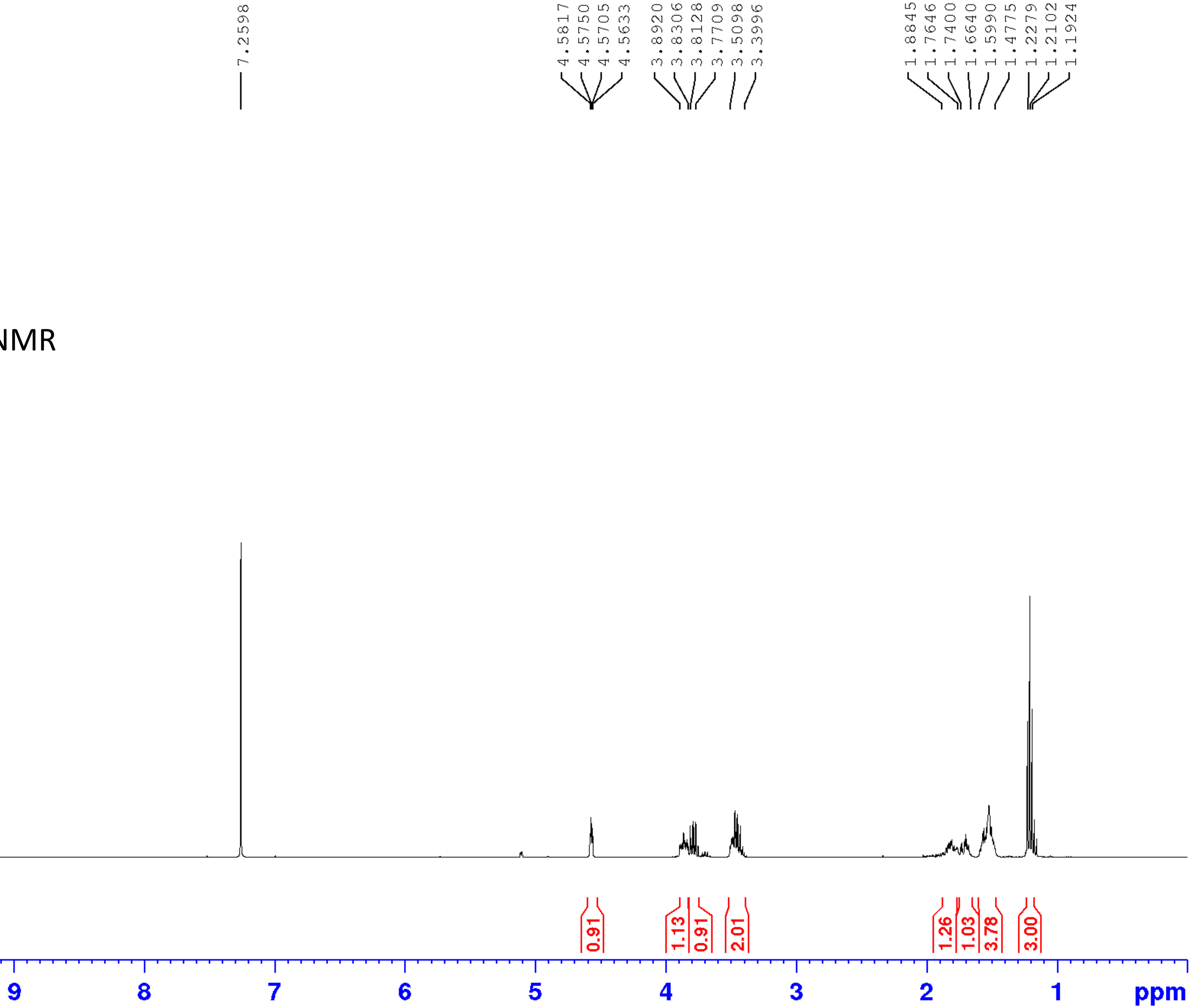


```
Current Data Parameters
NAME     vinn-4-183-5-islt-20230320
EXPNO                     1
PROCNO                    1

F2 - Acquisition Parameters
Date_              20230320
Time                  12.52 h
INSTRUM               spect
PROBHD     Z108618_0257 (
PULPROG                zg30
TD                    65536
SOLVENT               CDCl3
NS                       16
DS                        2
SWH                8012.820 Hz
FIDRES             0.244532 Hz
AQ                4.0894465 sec
RG                     71.8
DW                   62.400 usec
DE                     6.50 usec
TE                    295.4 K
D1               1.00000000 sec
TD0                       1
SFO1            400.1324708 MHz
NUC1                     1H
P1                    15.00 usec
PLW1            12.50000000 W

F2 - Processing parameters
SI                    65536
SF              400.1300101 MHz
WDW                      EM
SSB                       0
LB                     0.30 Hz
GB                        0
PC                     1.00
```

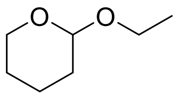

3x

100 MHz ^{13}C NMR

$CDCl_3$

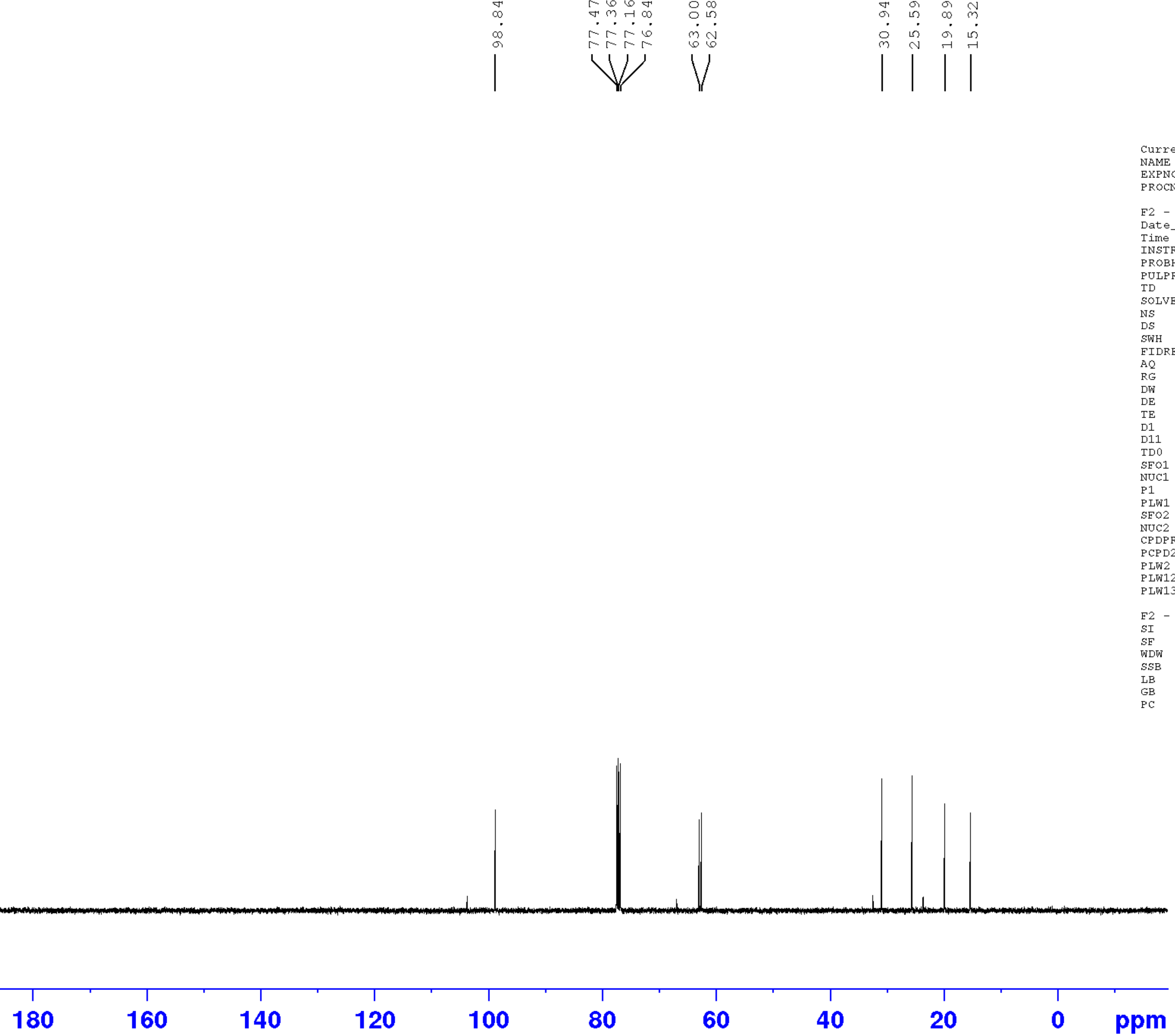


```
Current Data Parameters
NAME      vinn-4-183-5-islt-20230320
EXPNO                   2
PROCNO                  1

F2 - Acquisition Parameters
Date_            20230320
Time                12.56 h
INSTRUM             spect
PROBHD    Z108618_0257 (
PULPROG            zgpg30
TD                  65536
SOLVENT             CDCl3
NS                     37
DS                      4
SWH             24038.461 Hz
FIDRES           0.733596 Hz
AQ              1.3631488 sec
RG                    203
DW                 20.800 usec
DE                   6.50 usec
TE                  296.0 K
D1             2.00000000 sec
D11            0.03000000 sec
TD0                     1
SFO1          100.6228298 MHz
NUC1                  13C
P1                  10.00 usec
PLW1          51.00000000 W
SFO2          400.1316005 MHz
NUC2                   1H
CPDPRG[2          waltz16
PCPD2               90.00 usec
PLW2          12.50000000 W
PLW12          0.34722000 W
PLW13          0.17465000 W

F2 - Processing parameters
SI                  32768
SF            100.6127571 MHz
WDW                    EM
SSB                     0
LB                   1.00 Hz
GB                      0
PC                   1.40
```

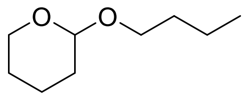

3y

400 MHz ^{1}H NMR

$CDCl_3$

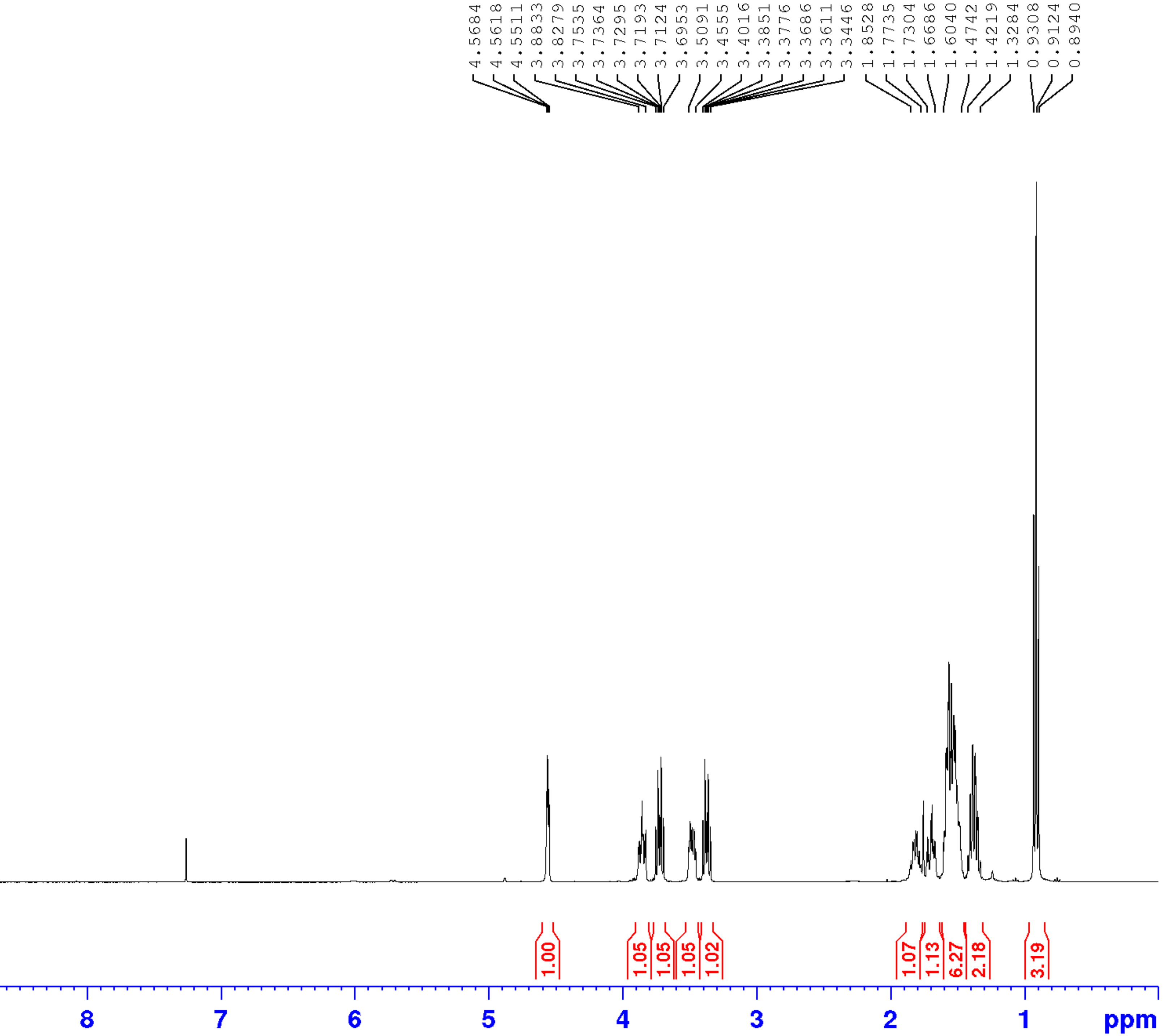

Current Data Parameters
NAME vinn-4-183-13-islt2-20230329
EXPNO 1
PROCNO 1

F2 - Acquisition Parameters
Date_ 20230329
Time 16.24 h
INSTRUM spect
PROBHD Z108618_0257 (
PULPROG zg30
TD 65536
SOLVENT CDCl3
NS 8
DS 2
SWH 8012.820 Hz
FIDRES 0.244532 Hz
AQ 4.0894465 sec
RG 64
DW 62.400 usec
DE 6.50 usec
TE 298.4 K
D1 1.00000000 sec
TD0 1
SFO1 400.1324708 MHz
NUC1 1H
P1 15.00 usec
PLW1 12.50000000 W

F2 - Processing parameters
SI 65536
SF 400.1300096 MHz
WDW EM
SSB 0
LB 0.30 Hz
GB 0
PC 1.00

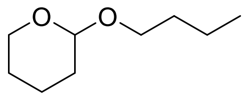

3y

100 MHz ^{13}C NMR

$CDCl_3$

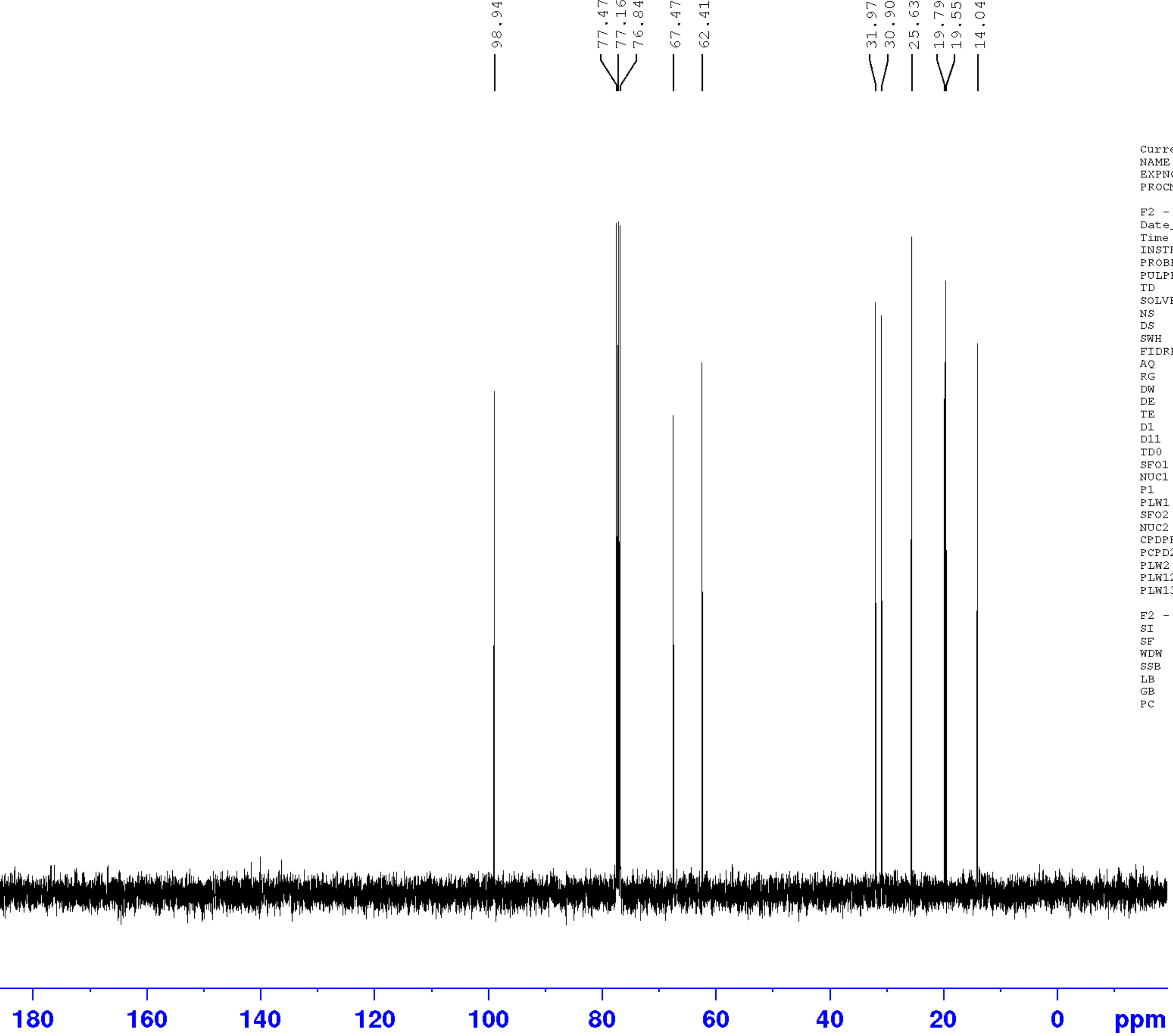


```
Current Data Parameters
NAME      vinn-4-183-13-islt2-2023032[illegible]
EXPNO                        2
PROCNO                       1

F2 - Acquisition Parameters
Date_              20230329
Time                  16.27 h
INSTRUM               spect
PROBHD   Z108618_0257 (
PULPROG              zgpg30
TD                    65536
SOLVENT               CDCl3
NS                       15
DS                        4
SWH               24038.461 Hz
FIDRES             0.733596 Hz
AQ                1.3631488 sec
RG                      203
DW                   20.800 usec
DE                     6.50 usec
TE                    298.3 K
D1               2.00000000 sec
D11              0.03000000 sec
TD0                       1
SFO1            100.6228298 MHz
NUC1                    13C
P1                    10.00 usec
PLW1            51.00000000 W
SFO2            400.1316005 MHz
NUC2                     1H
CPDPRG[2            waltz16
PCPD2                 90.00 usec
PLW2            12.50000000 W
PLW12            0.34722000 W
PLW13            0.17465000 W

F2 - Processing parameters
SI                    32768
SF              100.6127562 MHz
WDW                      EM
SSB                       0
LB                     1.00 Hz
GB                        0
PC                     1.40
```

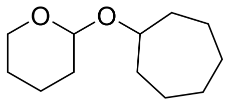

3z

500 MHz ^{1}H NMR

$CDCl_3$

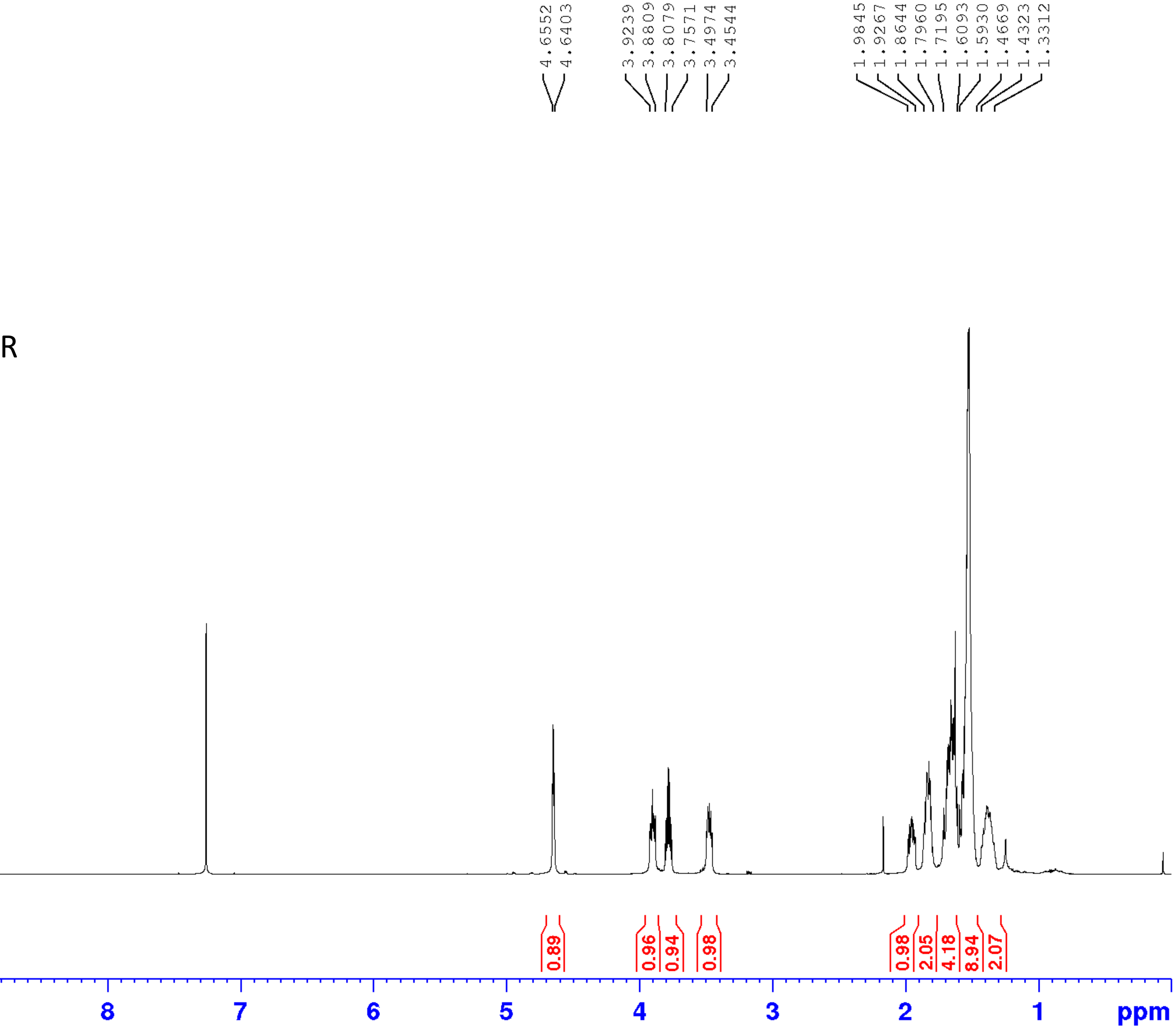

Current Data Parameters
NAME vinn-4-141-6-islt2-2020012
EXPNO 1
PROCNO 1

F2 - Acquisition Parameters
Date_ 20210121
Time 10.14 h
INSTRUM spect
PROBHD Z119470_0283 (
PULPROG zg30
TD 65536
SOLVENT CDCl3
NS 16
DS 2
SWH 10000.000 Hz
FIDRES 0.305176 Hz
AQ 3.2767999 sec
RG 63.76
DW 50.000 usec
DE 6.50 usec
TE 295.1 K
D1 1.00000000 sec
TD0 1
SFO1 500.1330883 MHz
NUC1 1H
P1 10.91 usec
PLW1 25.00000000 W

F2 - Processing parameters
SI 65536
SF 500.1300126 MHz
WDW EM
SSB 0
LB 0.30 Hz
GB 0
PC 1.00

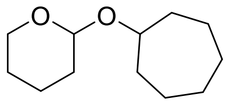

3z

125 MHz ^{13}C NMR

$CDCl_3$

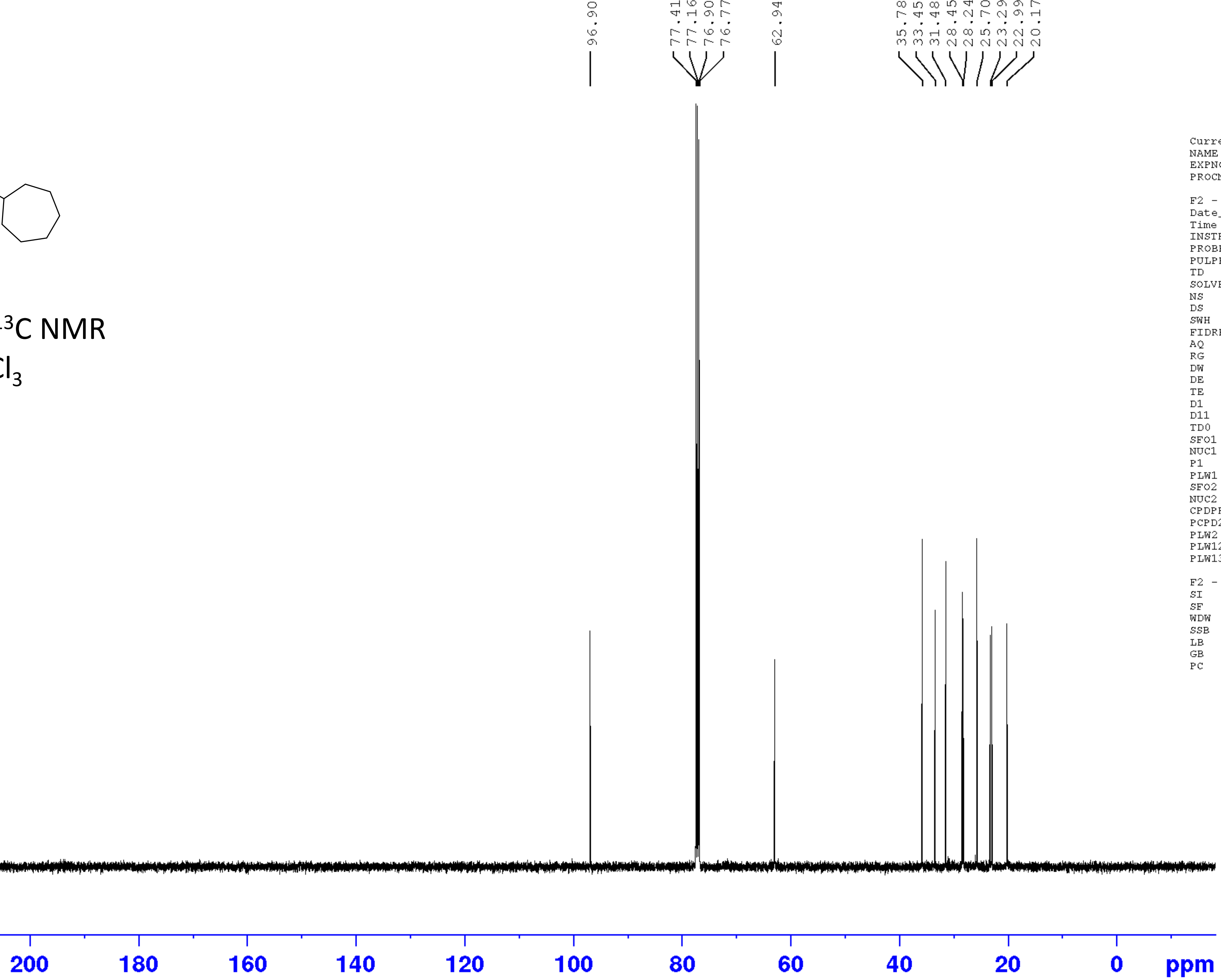


```
Current Data Parameters
NAME       vinn-4-141-6-islt2-20200120
EXPNO                     2
PROCNO                    1

F2 - Acquisition Parameters
Date_              20210121
Time                  10.28 h
INSTRUM               spect
PROBHD   Z119470_0283 (
PULPROG             zgpg30
TD                    65536
SOLVENT               CDCl3
NS                      256
DS                        4
SWH               29761.904 Hz
FIDRES             0.908261 Hz
AQ                1.1010048 sec
RG                   206.72
DW                   16.800 usec
DE                     6.50 usec
TE                    295.2 K
D1               2.00000000 sec
D11              0.03000000 sec
TD0                       1
SFO1            125.7703643 MHz
NUC1                    13C
P1                     9.75 usec
PLW1            94.00000000 W
SFO2            500.1320005 MHz
NUC2                     1H
CPDPRG[2            waltz16
PCPD2                 80.00 usec
PLW2            25.00000000 W
PLW12            0.46495000 W
PLW13            0.23387000 W

F2 - Processing parameters
SI                    32768
SF              125.7577733 MHz
WDW                      EM
SSB                       0
LB                     1.00 Hz
GB                        0
PC                     1.40
```

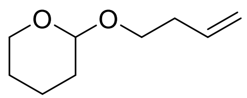

3aa

500 MHz ^{1}H NMR

$CDCl_3$

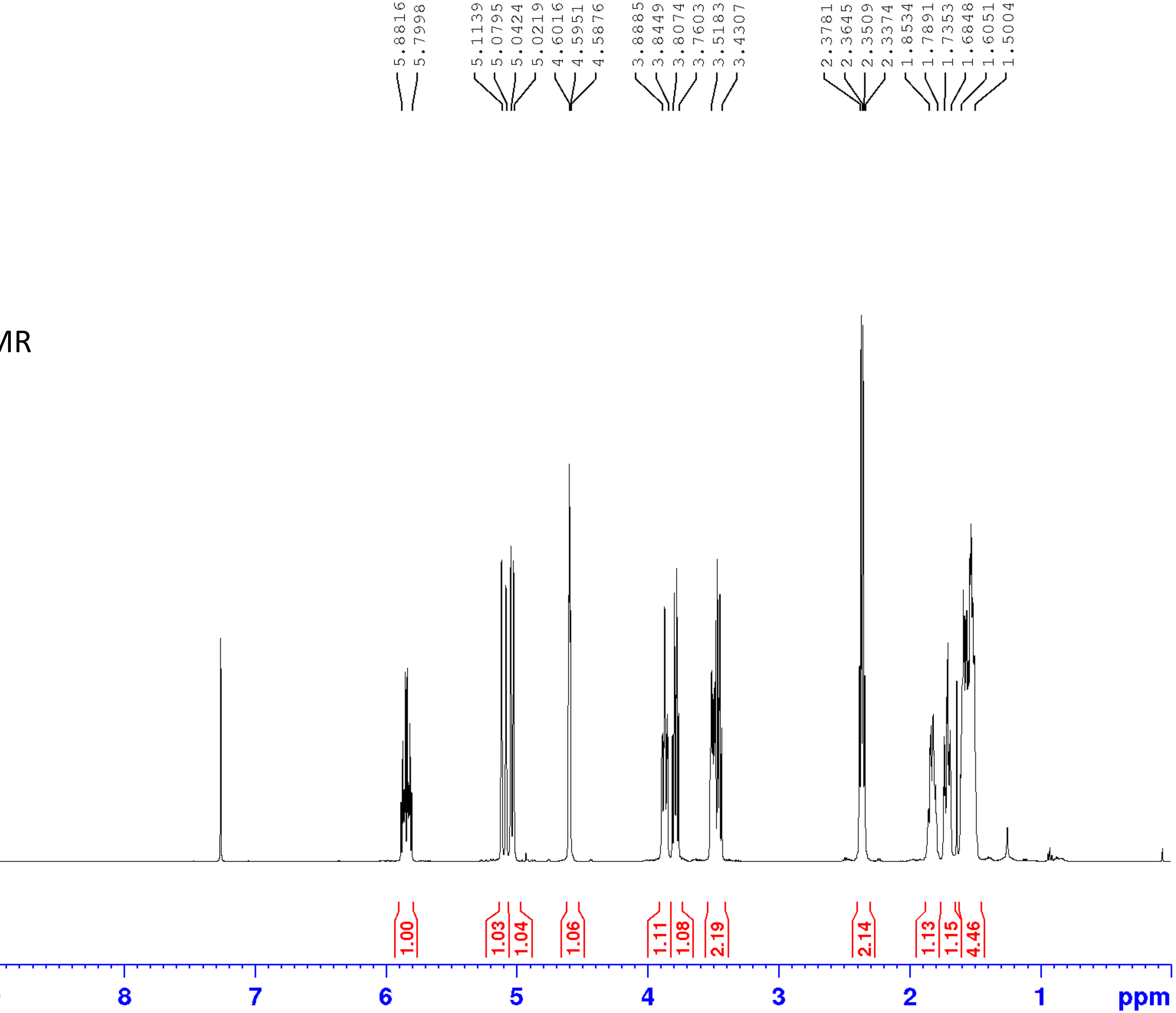

Current Data Parameters
NAME vinn-4-149-12-islt-2020013
EXPNO 1
PROCNO 1

F2 - Acquisition Parameters
Date_ 20210130
Time 18.42 h
INSTRUM spect
PROBHD Z119470_0283 (
PULPROG zg30
TD 65536
SOLVENT CDCl3
NS 16
DS 2
SWH 10000.000 Hz
FIDRES 0.305176 Hz
AQ 3.2767999 sec
RG 63.76
DW 50.000 usec
DE 6.50 usec
TE 295.1 K
D1 1.00000000 sec
TD0 1
SFO1 500.1330883 MHz
NUC1 1H
P1 10.91 usec
PLW1 25.00000000 W

F2 - Processing parameters
SI 65536
SF 500.1300122 MHz
WDW EM
SSB 0
LB 0.30 Hz
GB 0
PC 1.00

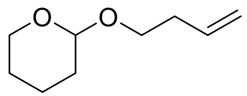

3aa

125 MHz ^{13}C NMR

$CDCl_3$

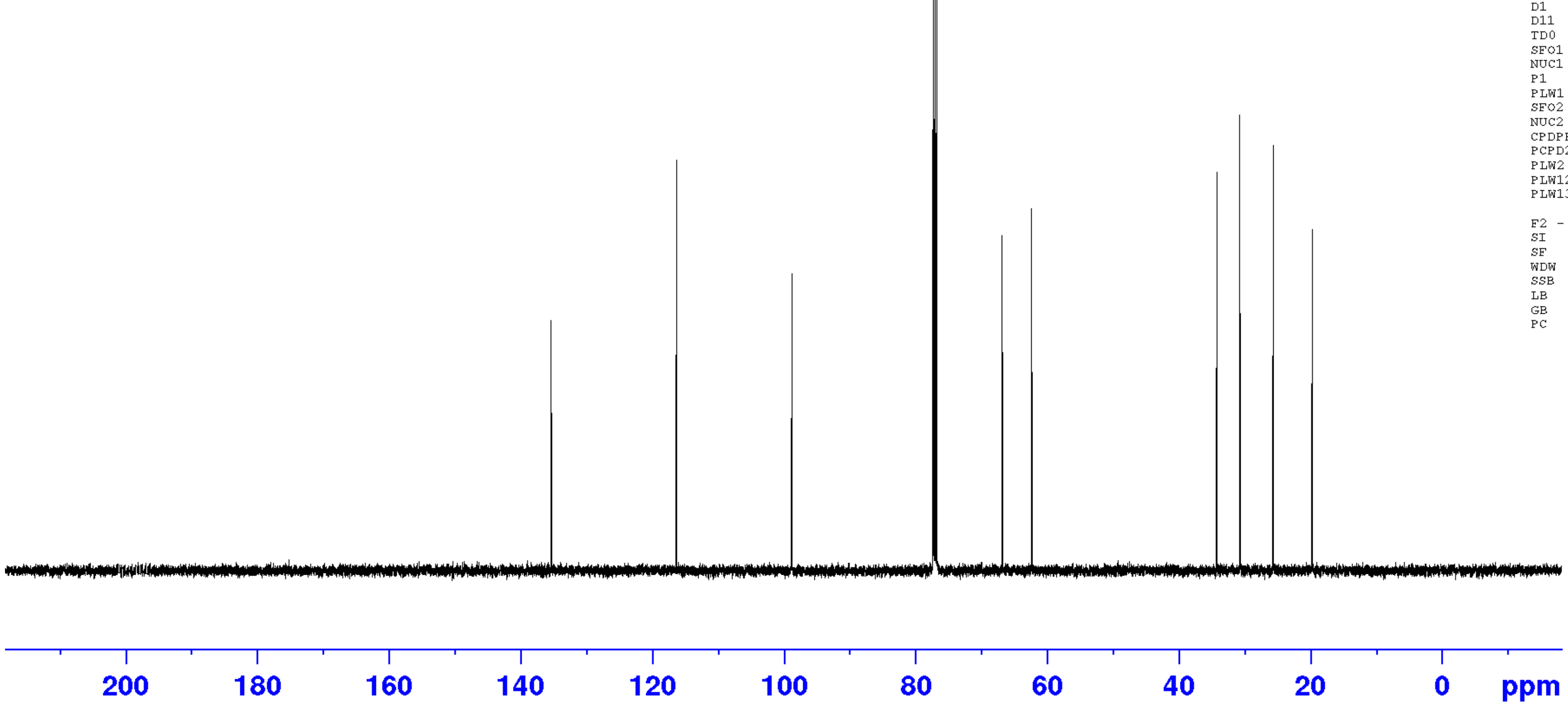


```
Current Data Parameters
NAME     vinn-4-149-12-islt-20200130
EXPNO                    2
PROCNO                   1

F2 - Acquisition Parameters
Date_              20210130
Time                  18.49 h
INSTRUM               spect
PROBHD   Z119470_0283 (
PULPROG              zgpg30
TD                    65536
SOLVENT               CDCl3
NS                      128
DS                        4
SWH              29761.904 Hz
FIDRES            0.908261 Hz
AQ               1.1010048 sec
RG                   206.72
DW                   16.800 usec
DE                     6.50 usec
TE                    295.2 K
D1               2.00000000 sec
D11              0.03000000 sec
TD0                       1
SFO1            125.7703643 MHz
NUC1                    13C
P1                     9.75 usec
PLW1            94.00000000 W
SFO2            500.1320005 MHz
NUC2                     1H
CPDPRG[2            waltz16
PCPD2                 80.00 usec
PLW2            25.00000000 W
PLW12            0.46495000 W
PLW13            0.23387000 W

F2 - Processing parameters
SI                    32768
SF              125.7577732 MHz
WDW                      EM
SSB                       0
LB                     1.00 Hz
GB                        0
PC                     1.40
```

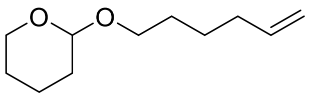

3ab

500 MHz ^{1}H NMR

$CDCl_3$

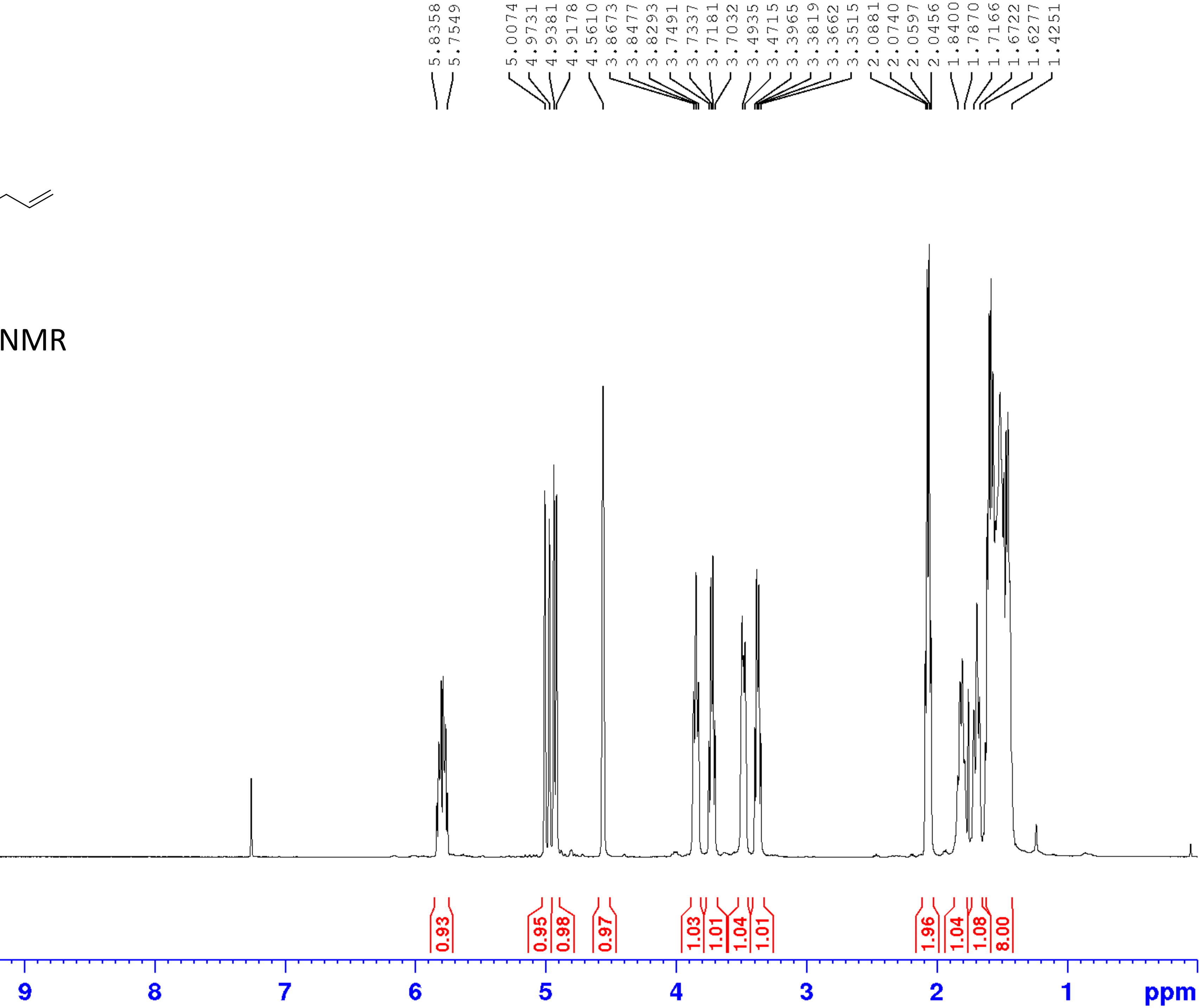


```
Current Data Parameters
NAME      vinn-4-141-7-islt-20201228
EXPNO                    1
PROCNO                   1

F2 - Acquisition Parameters
Date_             20201228
Time                 21.22 h
INSTRUM              spect
PROBHD   Z119470_0283 (
PULPROG               zg30
TD                   65536
SOLVENT              CDCl3
NS                      16
DS                       2
SWH              10000.000 Hz
FIDRES            0.305176 Hz
AQ               3.2767999 sec
RG                   30.85
DW                  50.000 usec
DE                    6.50 usec
TE                   295.2 K
D1              1.00000000 sec
TD0                      1
SFO1           500.1330883 MHz
NUC1                    1H
P1                   10.91 usec
PLW1           25.00000000 W

F2 - Processing parameters
SI                   65536
SF             500.1300123 MHz
WDW                     EM
SSB                      0
LB                    0.30 Hz
GB                       0
PC                    1.00
```

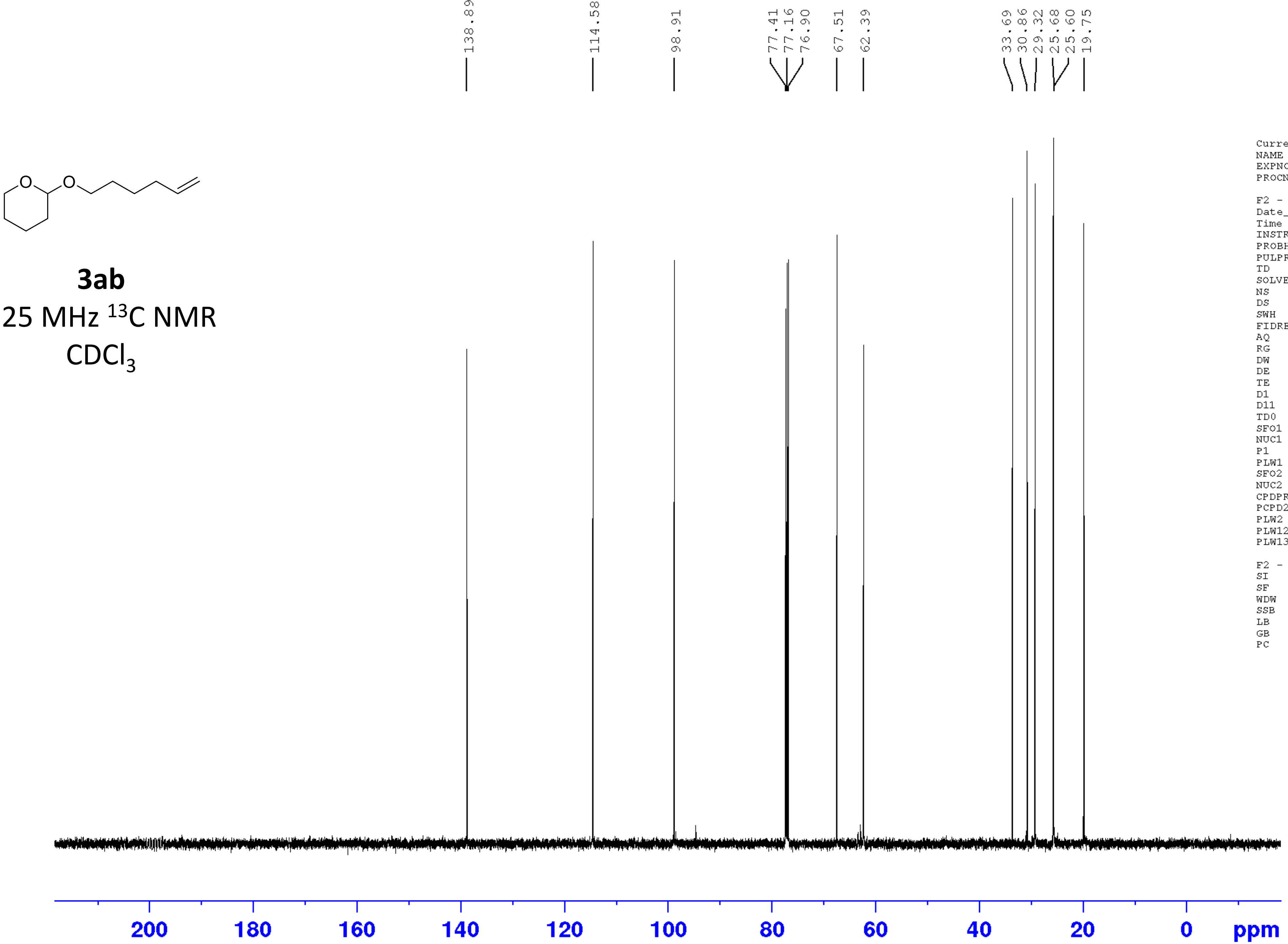

3ab

125 MHz ^{13}C NMR

$CDCl_3$

```
Current Data Parameters
NAME      vinn-4-141-7-islt-20201228
EXPNO                  2
PROCNO                 1

F2 - Acquisition Parameters
Date_           20201228
Time               21.28 h
INSTRUM            spect
PROBHD   Z119470_0283 (
PULPROG          zgpg30
TD                65536
SOLVENT           CDCl3
NS                  100
DS                    4
SWH           29761.904 Hz
FIDRES         0.908261 Hz
AQ            1.1010048 sec
RG               206.72
DW               16.800 usec
DE                 6.50 usec
TE                295.1 K
D1           2.00000000 sec
D11          0.03000000 sec
TD0                   1
SFO1        125.7703643 MHz
NUC1                13C
P1                 9.75 usec
PLW1        94.00000000 W
SFO2        500.1320005 MHz
NUC2                 1H
CPDPRG[2        waltz16
PCPD2             80.00 usec
PLW2        25.00000000 W
PLW12        0.46495000 W
PLW13        0.23387000 W

F2 - Processing parameters
SI                32768
SF          125.7577754 MHz
WDW                  EM
SSB                   0
LB                 1.00 Hz
GB                    0
PC                 1.40
```

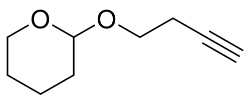

3ac

500 MHz ^{1}H NMR

$CDCl_3$

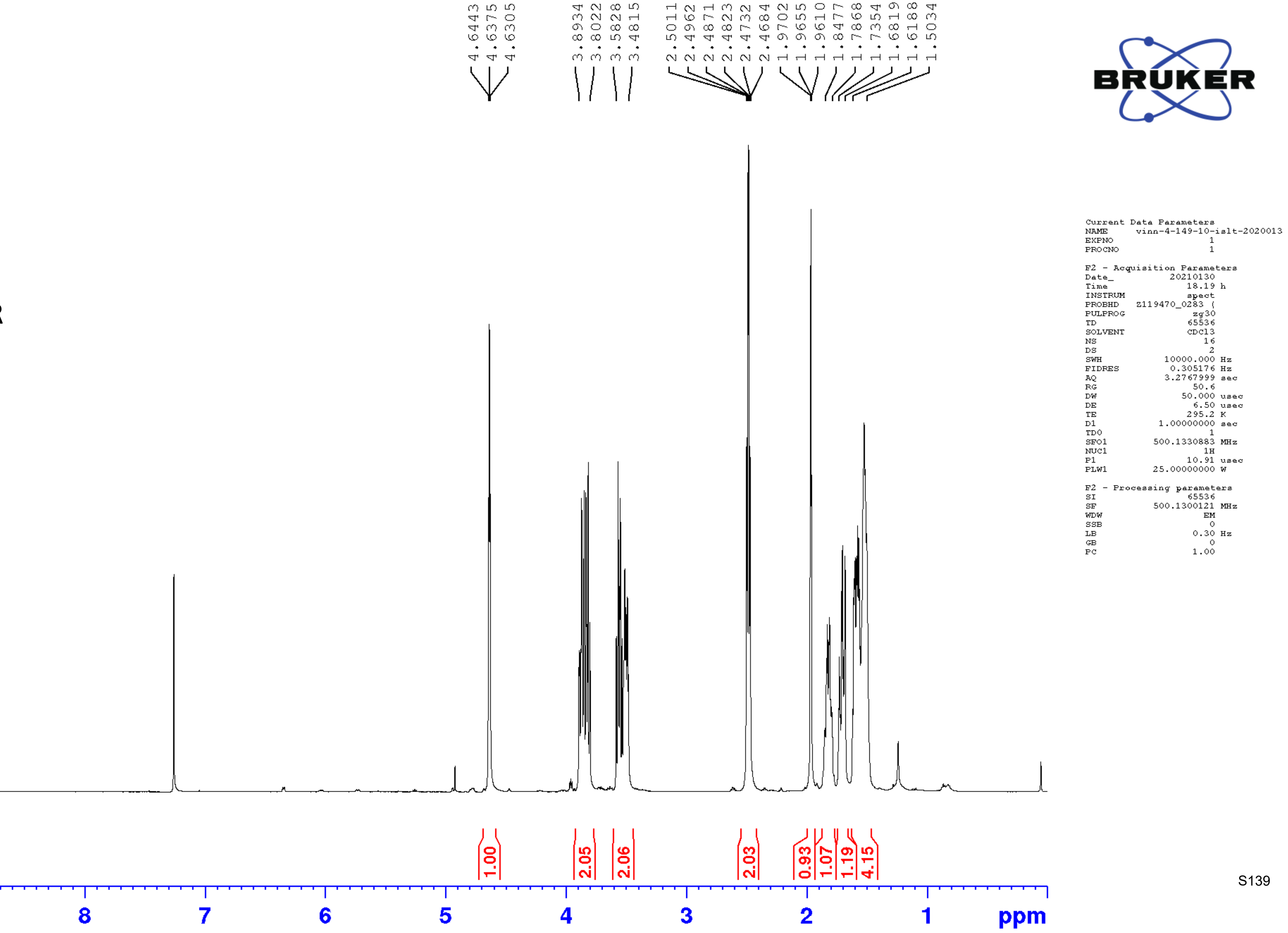

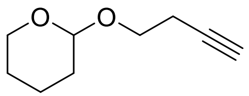

3ac
125 MHz ^{13}C NMR
$CDCl_3$

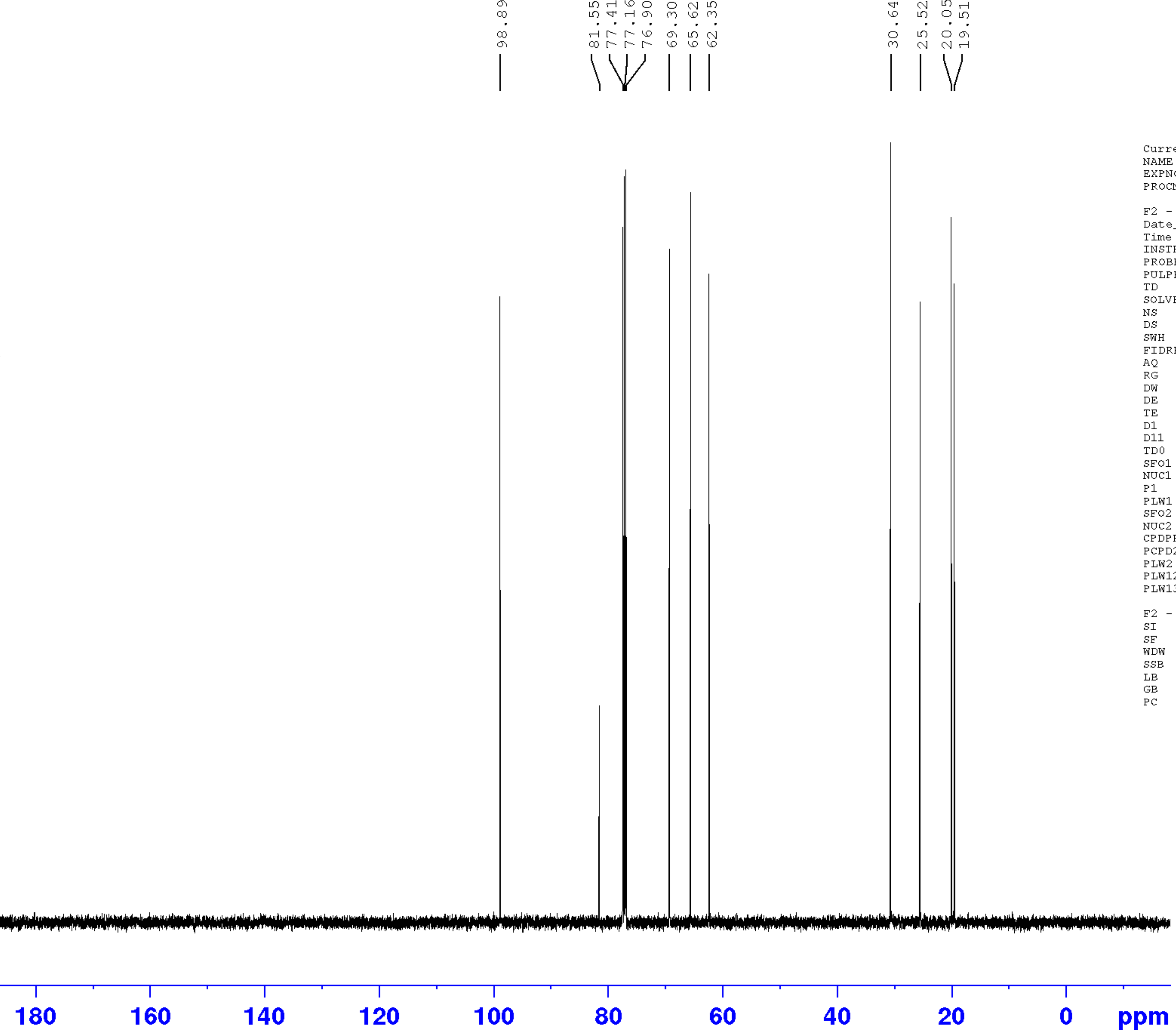


```
Current Data Parameters
NAME      vinn-4-149-10-islt-20200130
EXPNO                    2
PROCNO                   1

F2 - Acquisition Parameters
Date_            20210130
Time                18.27 h
INSTRUM             spect
PROBHD   Z119470_0283 (
PULPROG            zgpg30
TD                  65536
SOLVENT             CDCl3
NS                    128
DS                      4
SWH             29761.904 Hz
FIDRES           0.908261 Hz
AQ              1.1010048 sec
RG                 206.72
DW                 16.800 usec
DE                   6.50 usec
TE                  295.2 K
D1             2.00000000 sec
D11            0.03000000 sec
TD0                     1
SFO1          125.7703643 MHz
NUC1                  13C
P1                   9.75 usec
PLW1          94.00000000 W
SFO2          500.1320005 MHz
NUC2                   1H
CPDPRG[2          waltz16
PCPD2               80.00 usec
PLW2          25.00000000 W
PLW12          0.46495000 W
PLW13          0.23387000 W

F2 - Processing parameters
SI                  32768
SF            125.7577746 MHz
WDW                    EM
SSB                     0
LB                   1.00 Hz
GB                      0
PC                   1.40
```

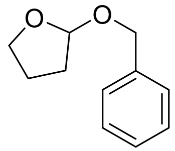

5a

500 MHz ^{1}H NMR

$CDCl_3$

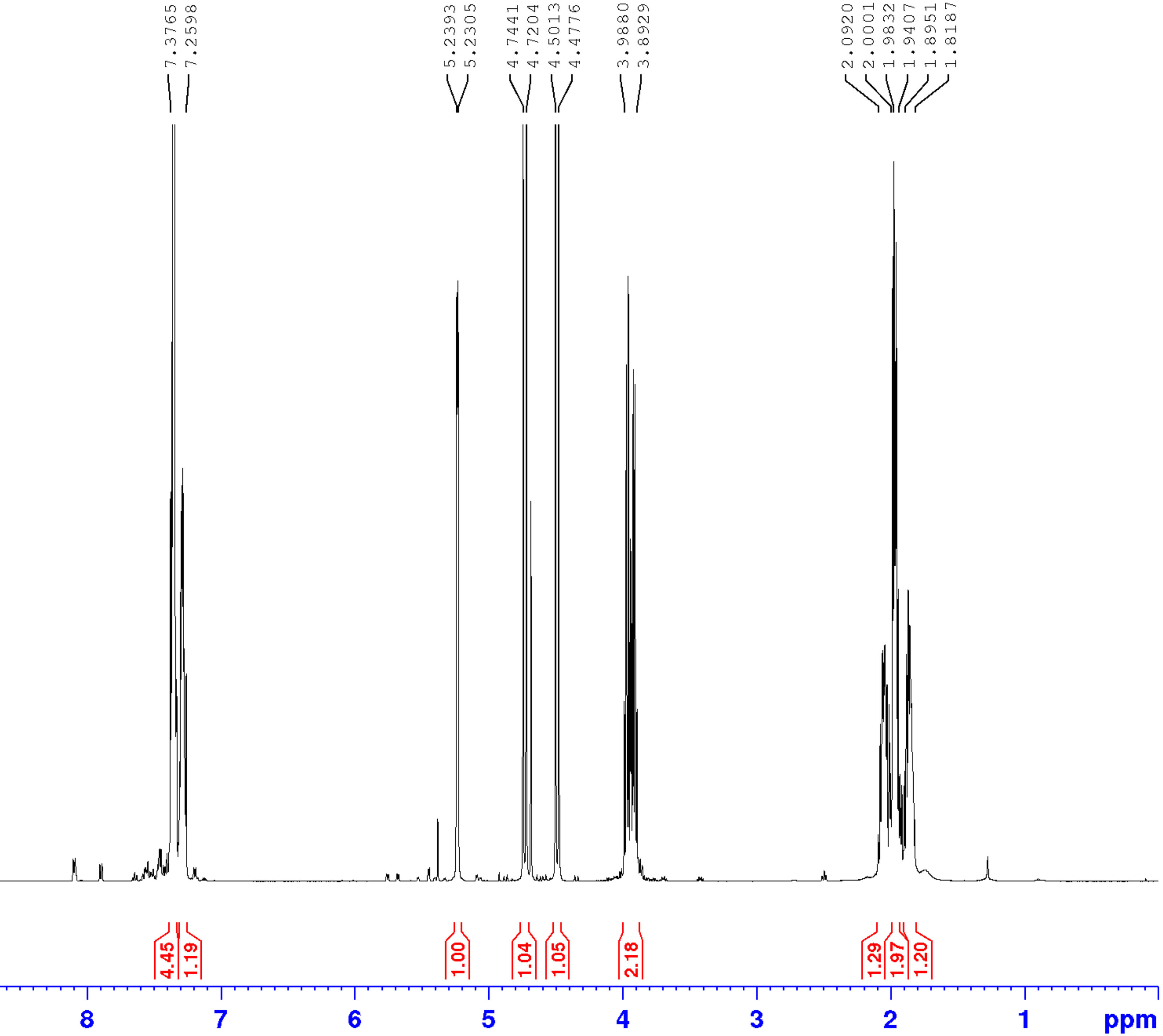


```
Current Data Parameters
NAME      vinn-4-131-2-islt-20201130
EXPNO                     1
PROCNO                    1

F2 - Acquisition Parameters
Date_              20201130
Time                  16.28 h
INSTRUM               spect
PROBHD    Z119470_0283 (
PULPROG                zg30
TD                    65536
SOLVENT               CDCl3
NS                       16
DS                        2
SWH               10000.000 Hz
FIDRES             0.305176 Hz
AQ                3.2767999 sec
RG                    30.85
DW                   50.000 usec
DE                     6.50 usec
TE                    295.1 K
D1               1.00000000 sec
TD0                       1
SFO1            500.1330883 MHz
NUC1                     1H
P1                    10.91 usec
PLW1            25.00000000 W

F2 - Processing parameters
SI                    65536
SF              500.1300121 MHz
WDW                      EM
SSB                       0
LB                     0.30 Hz
GB                        0
PC                     1.00
```

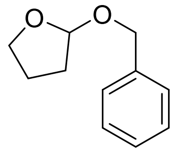

5a

125 MHz ^{13}C NMR

$CDCl_3$

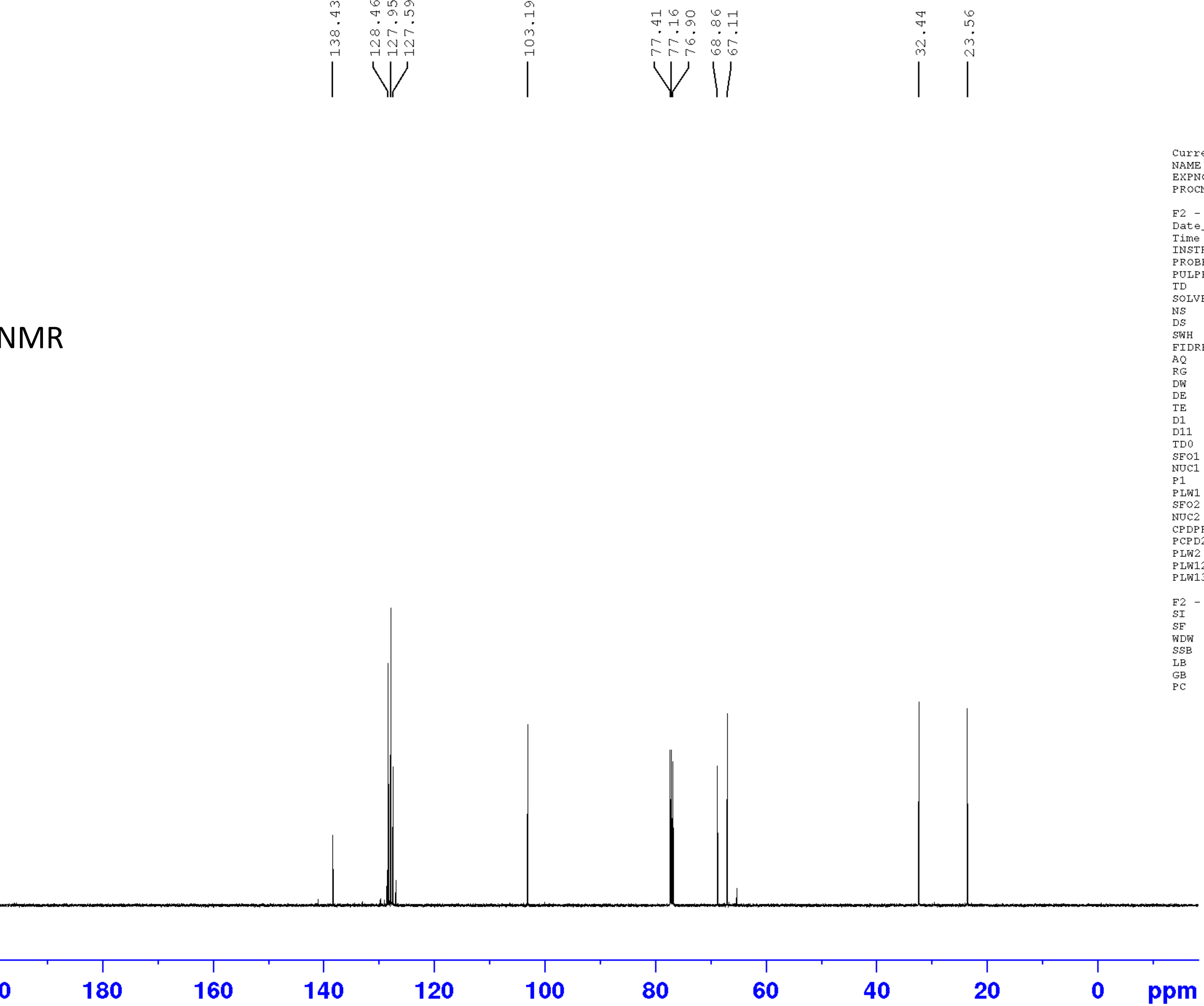

Current Data Parameters
NAME vinn-4-131-2-islt-20201130
EXPNO 2
PROCNO 1

F2 - Acquisition Parameters
Date_ 20201130
Time 16.35 h
INSTRUM spect
PROBHD Z119470_0283 (
PULPROG zgpg30
TD 65536
SOLVENT CDCl3
NS 115
DS 4
SWH 29761.904 Hz
FIDRES 0.908261 Hz
AQ 1.1010048 sec
RG 206.72
DW 16.800 usec
DE 6.50 usec
TE 295.2 K
D1 2.00000000 sec
D11 0.03000000 sec
TD0 1
SFO1 125.7703643 MHz
NUC1 13C
P1 9.75 usec
PLW1 94.00000000 W
SFO2 500.1320005 MHz
NUC2 1H
CPDPRG[2 waltz16
PCPD2 80.00 usec
PLW2 25.00000000 W
PLW12 0.46495000 W
PLW13 0.23387000 W

F2 - Processing parameters
SI 32768
SF 125.7577804 MHz
WDW EM
SSB 0
LB 1.00 Hz
GB 0
PC 1.40

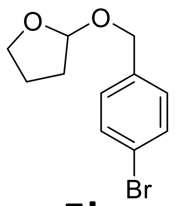

5b

500 MHz ^{1}H NMR

$CDCl_3$

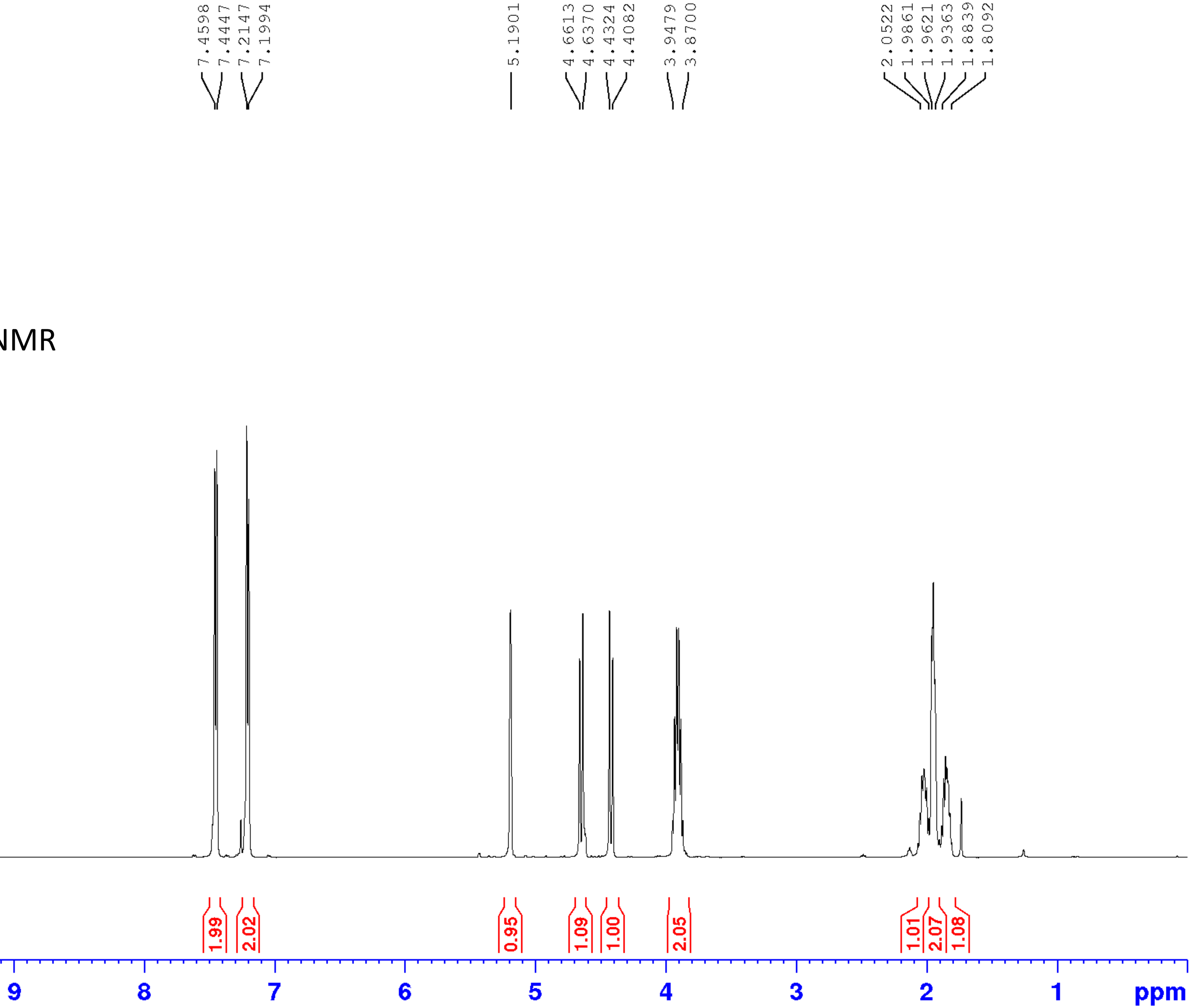


```
Current Data Parameters
NAME      vinn-4-137-8-islt-20201210
EXPNO                      1
PROCNO                     1

F2 - Acquisition Parameters
Date_              20201211
Time                 5.37 h
INSTRUM              spect
PROBHD    Z119470_0283 (
PULPROG              zg30
TD                   65536
SOLVENT              CDCl3
NS                      16
DS                       2
SWH             10000.000 Hz
FIDRES           0.305176 Hz
AQ              3.2767999 sec
RG                  30.85
DW                 50.000 usec
DE                   6.50 usec
TE                  295.1 K
D1             1.00000000 sec
TD0                      1
SFO1          500.1330883 MHz
NUC1                    1H
P1                  10.91 usec
PLW1          25.00000000 W

F2 - Processing parameters
SI                   65536
SF            500.1300122 MHz
WDW                     EM
SSB                      0
LB                    0.30 Hz
GB                       0
PC                    1.00
```

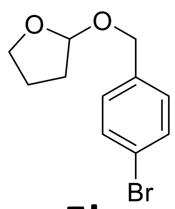

5b

125 MHz ^{13}C NMR

$CDCl_3$

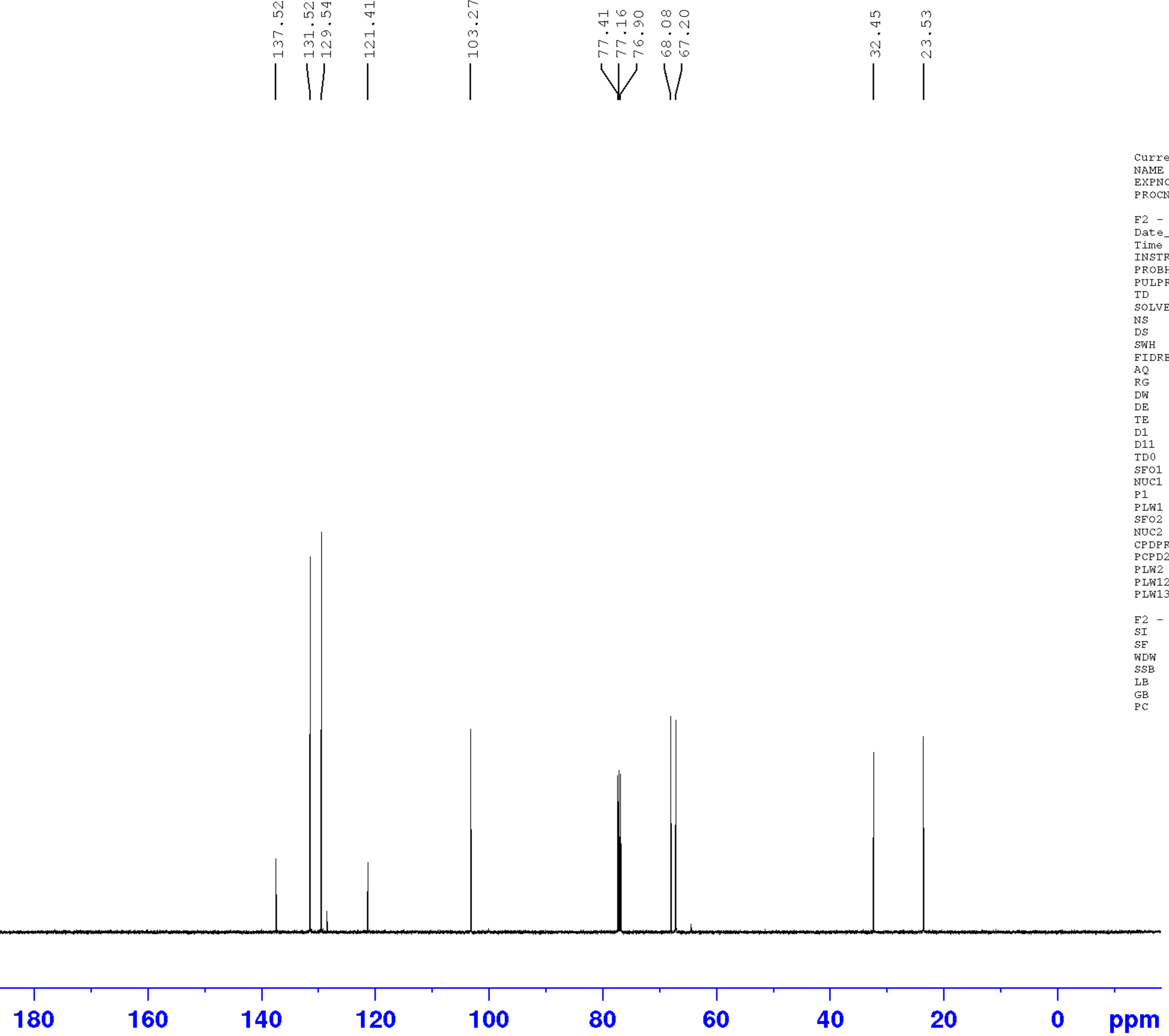


```
Current Data Parameters
NAME      vinn-4-137-8-islt-20201210
EXPNO                     2
PROCNO                    1

F2 - Acquisition Parameters
Date_              20201211
Time                 5.43 h
INSTRUM               spect
PROBHD     Z119470_0283 (
PULPROG             zgpg30
TD                    65536
SOLVENT               CDCl3
NS                      100
DS                        4
SWH           29761.904 Hz
FIDRES         0.908261 Hz
AQ           1.1010048 sec
RG                   206.72
DW              16.800 usec
DE                6.50 usec
TE                  295.1 K
D1          2.00000000 sec
D11         0.03000000 sec
TD0                       1
SFO1        125.7703643 MHz
NUC1                    13C
P1                9.75 usec
PLW1         94.00000000 W
SFO2        500.1320005 MHz
NUC2                     1H
CPDPRG[2            waltz16
PCPD2            80.00 usec
PLW2         25.00000000 W
PLW12         0.46495000 W
PLW13         0.23387000 W

F2 - Processing parameters
SI                    32768
SF          125.7577798 MHz
WDW                      EM
SSB                       0
LB                  1.00 Hz
GB                        0
PC                     1.40
```

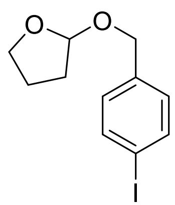

5c

500 MHz ^{1}H NMR

$CDCl_3$

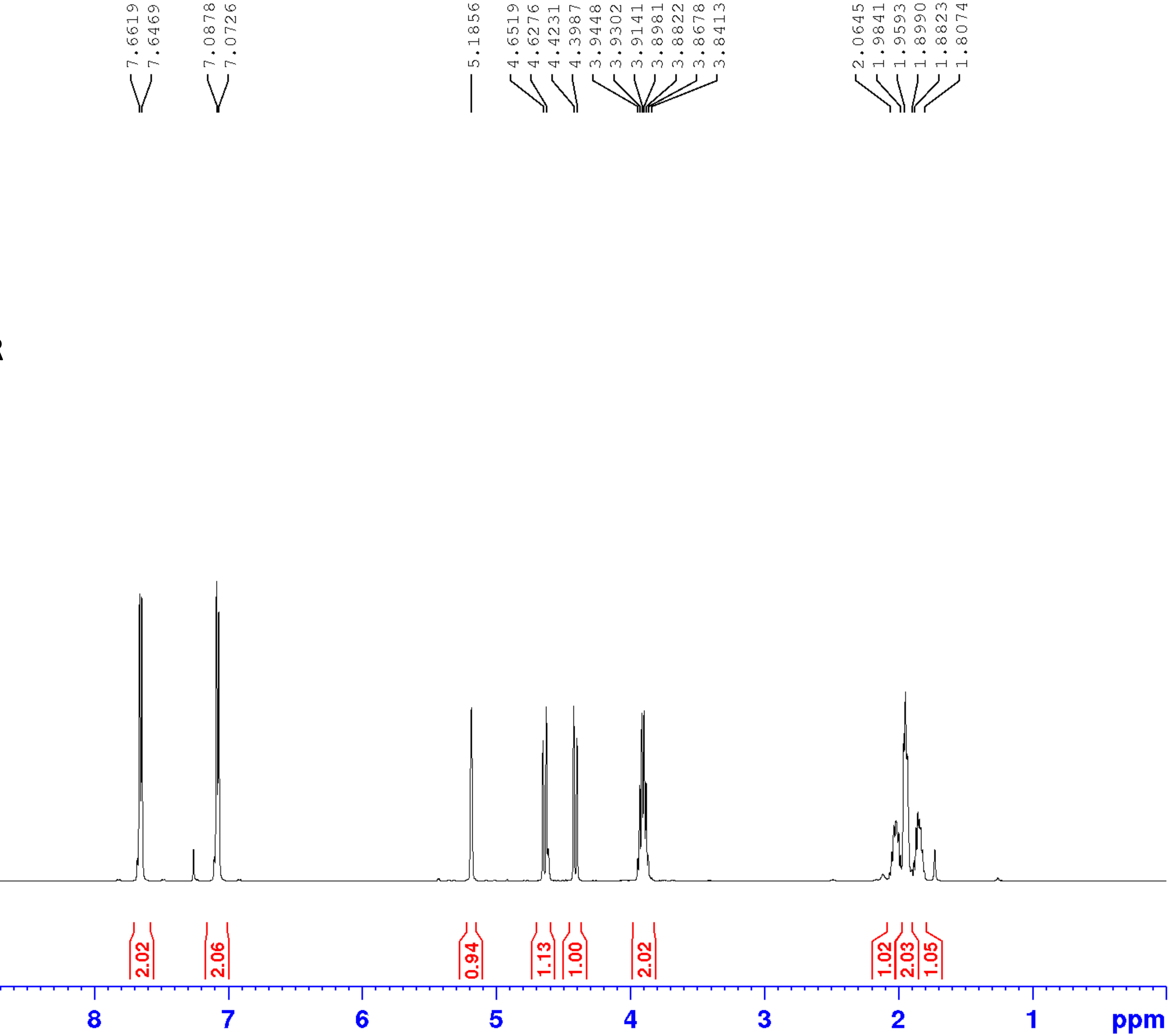

BRUKER

Current Data Parameters
NAME vinn-4-137-9-islt-20201210
EXPNO 1
PROCNO 1

F2 - Acquisition Parameters
Date_ 20201211
Time 5.47 h
INSTRUM spect
PROBHD Z119470_0283 (
PULPROG zg30
TD 65536
SOLVENT CDCl3
NS 16
DS 2
SWH 10000.000 Hz
FIDRES 0.305176 Hz
AQ 3.2767999 sec
RG 30.85
DW 50.000 usec
DE 6.50 usec
TE 295.1 K
D1 1.00000000 sec
TD0 1
SFO1 500.1330883 MHz
NUC1 1H
P1 10.91 usec
PLW1 25.00000000 W

F2 - Processing parameters
SI 65536
SF 500.1300122 MHz
WDW EM
SSB 0
LB 0.30 Hz
GB 0
PC 1.00

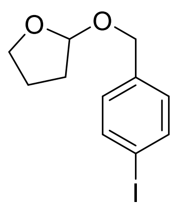

5c

125 MHz ^{13}C NMR

$CDCl_3$

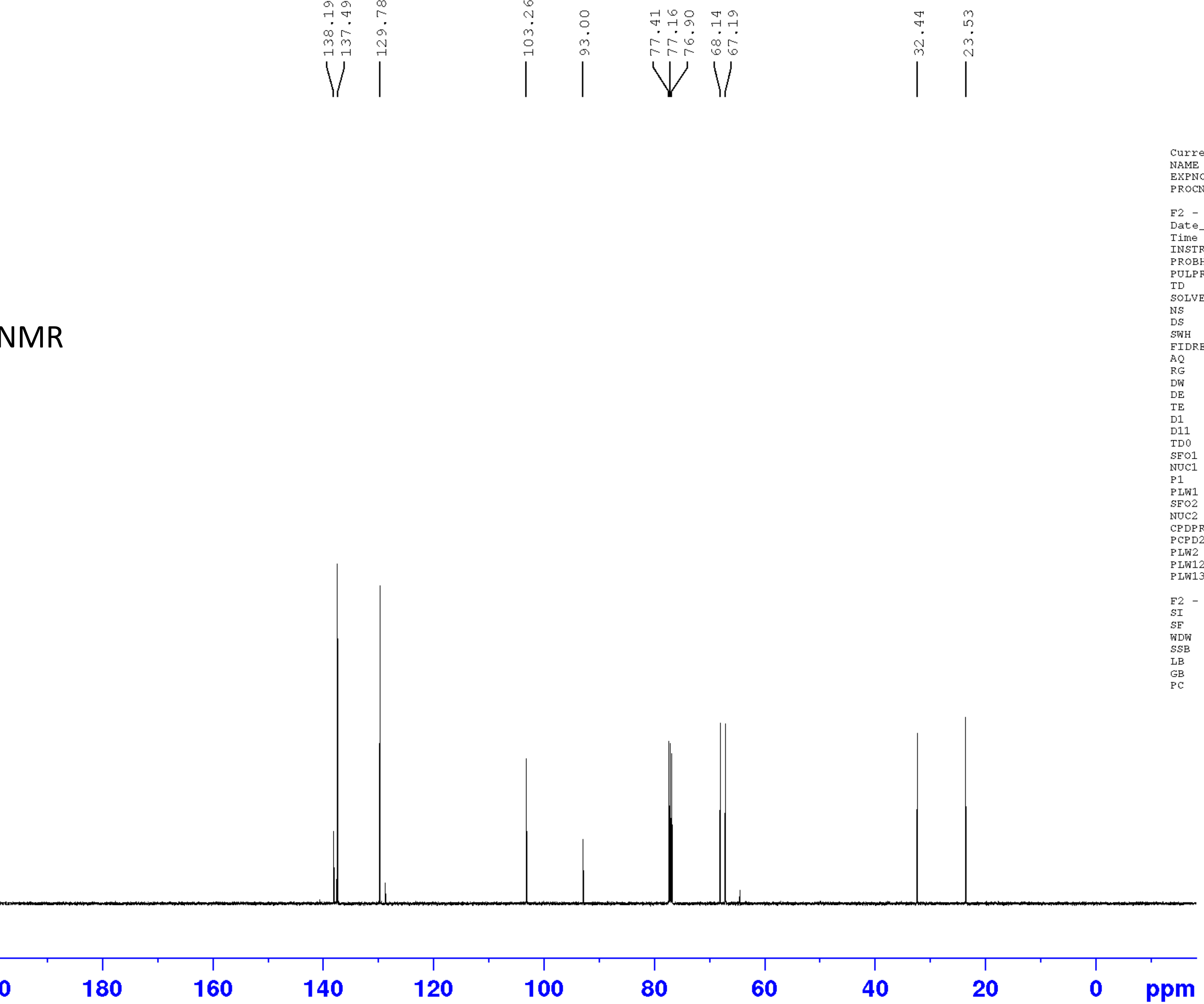


```
Current Data Parameters
NAME      vinn-4-137-9-islt-20201210
EXPNO                    2
PROCNO                   1

F2 - Acquisition Parameters
Date_             20201211
Time                  5.52 h
INSTRUM              spect
PROBHD    Z119470_0283 (
PULPROG            zgpg30
TD                  65536
SOLVENT             CDCl3
NS                    100
DS                      4
SWH             29761.904 Hz
FIDRES           0.908261 Hz
AQ              1.1010048 sec
RG                 206.72
DW                 16.800 usec
DE                   6.50 usec
TE                  295.2 K
D1             2.00000000 sec
D11            0.03000000 sec
TD0                     1
SFO1          125.7703643 MHz
NUC1                  13C
P1                   9.75 usec
PLW1          94.00000000 W
SFO2          500.1320005 MHz
NUC2                   1H
CPDPRG[2          waltz16
PCPD2               80.00 usec
PLW2          25.00000000 W
PLW12          0.46495000 W
PLW13          0.23387000 W

F2 - Processing parameters
SI                  32768
SF            125.7577813 MHz
WDW                    EM
SSB                     0
LB                   1.00 Hz
GB                      0
PC                   1.40
```

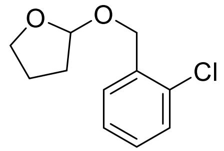

5d

500 MHz ^{1}H NMR

$CDCl_3$

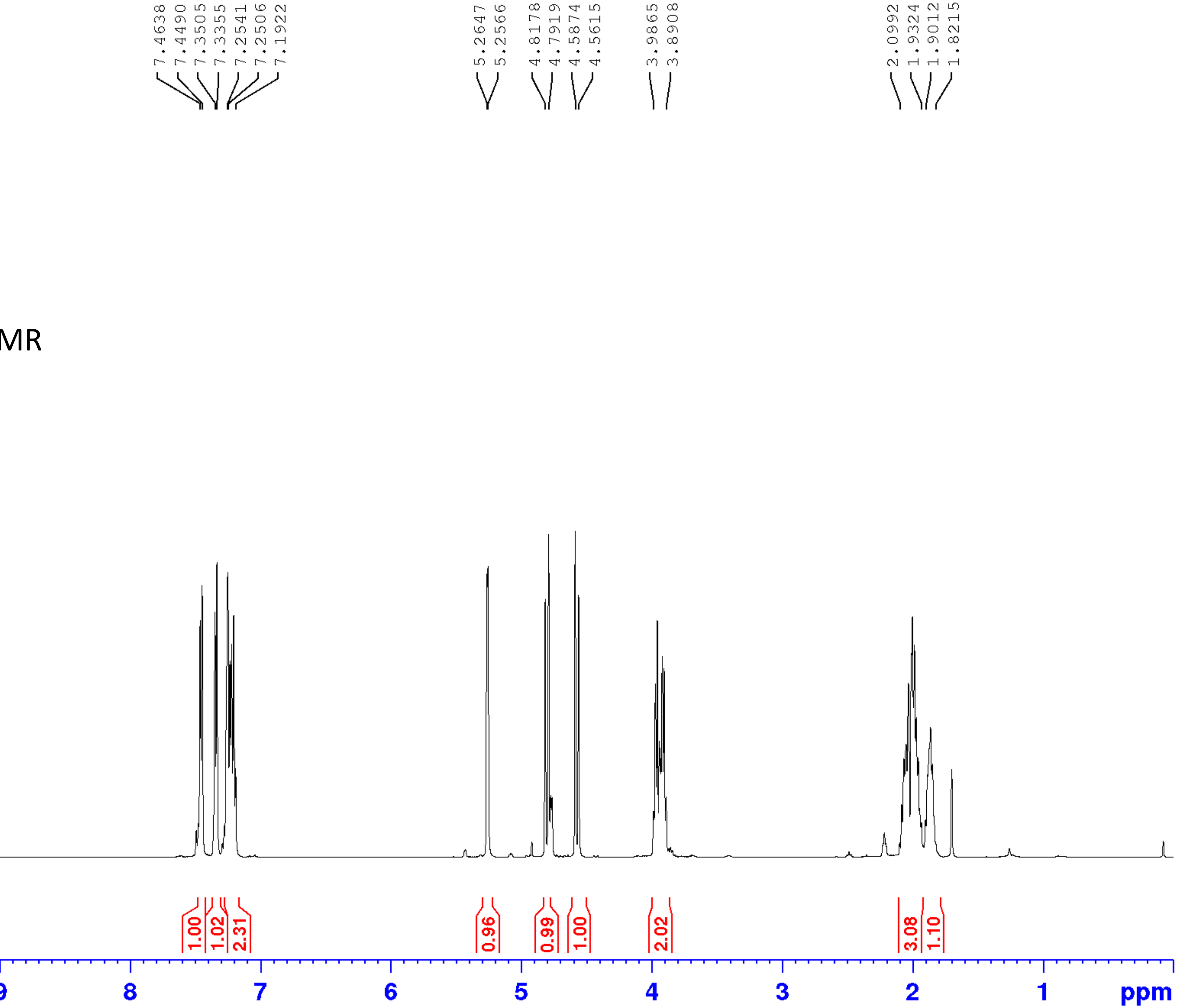


```
Current Data Parameters
NAME     vinn-4-137-17-islt-2020121
EXPNO                   1
PROCNO                  1

F2 - Acquisition Parameters
Date_            20201211
Time                6.43 h
INSTRUM             spect
PROBHD   Z119470_0283 (
PULPROG              zg30
TD                  65536
SOLVENT             CDCl3
NS                     16
DS                      2
SWH             10000.000 Hz
FIDRES           0.305176 Hz
AQ              3.2767999 sec
RG                  30.85
DW                 50.000 usec
DE                   6.50 usec
TE                  295.2 K
D1             1.00000000 sec
TD0                     1
SFO1          500.1330883 MHz
NUC1                   1H
P1                  10.91 usec
PLW1          25.00000000 W

F2 - Processing parameters
SI                  65536
SF            500.1300133 MHz
WDW                    EM
SSB                     0
LB                   0.30 Hz
GB                      0
PC                   1.00
```

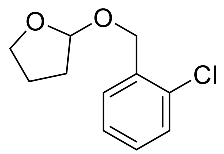

5d

125 MHz ^{13}C NMR

$CDCl_3$

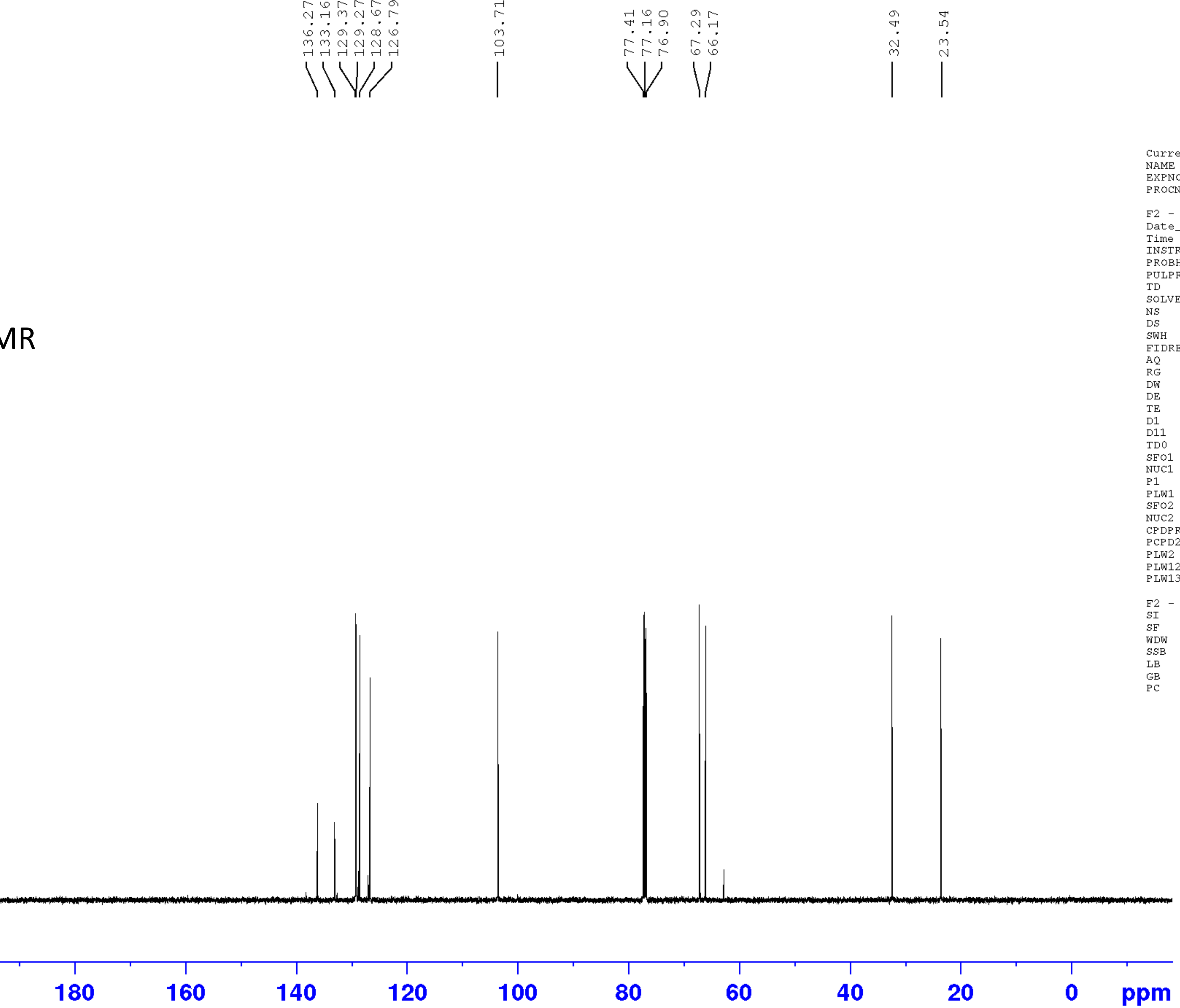


```
Current Data Parameters
NAME      vinn-4-137-17-islt-20201210
EXPNO                    2
PROCNO                   1

F2 - Acquisition Parameters
Date_             20201211
Time                  6.49 h
INSTRUM              spect
PROBHD    Z119470_0283 (
PULPROG            zgpg30
TD                  65536
SOLVENT             CDCl3
NS                    100
DS                      4
SWH             29761.904 Hz
FIDRES           0.908261 Hz
AQ              1.1010048 sec
RG                 206.72
DW                 16.800 usec
DE                   6.50 usec
TE                  295.2 K
D1             2.00000000 sec
D11            0.03000000 sec
TD0                     1
SFO1          125.7703643 MHz
NUC1                  13C
P1                   9.75 usec
PLW1          94.00000000 W
SFO2          500.1320005 MHz
NUC2                   1H
CPDPRG[2          waltz16
PCPD2               80.00 usec
PLW2          25.00000000 W
PLW12          0.46495000 W
PLW13          0.23387000 W

F2 - Processing parameters
SI                  32768
SF            125.7577777 MHz
WDW                    EM
SSB                     0
LB                   1.00 Hz
GB                      0
PC                   1.40
```

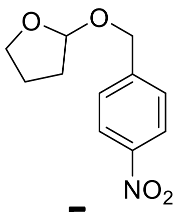

5e

500 MHz ^{1}H NMR

$CDCl_3$

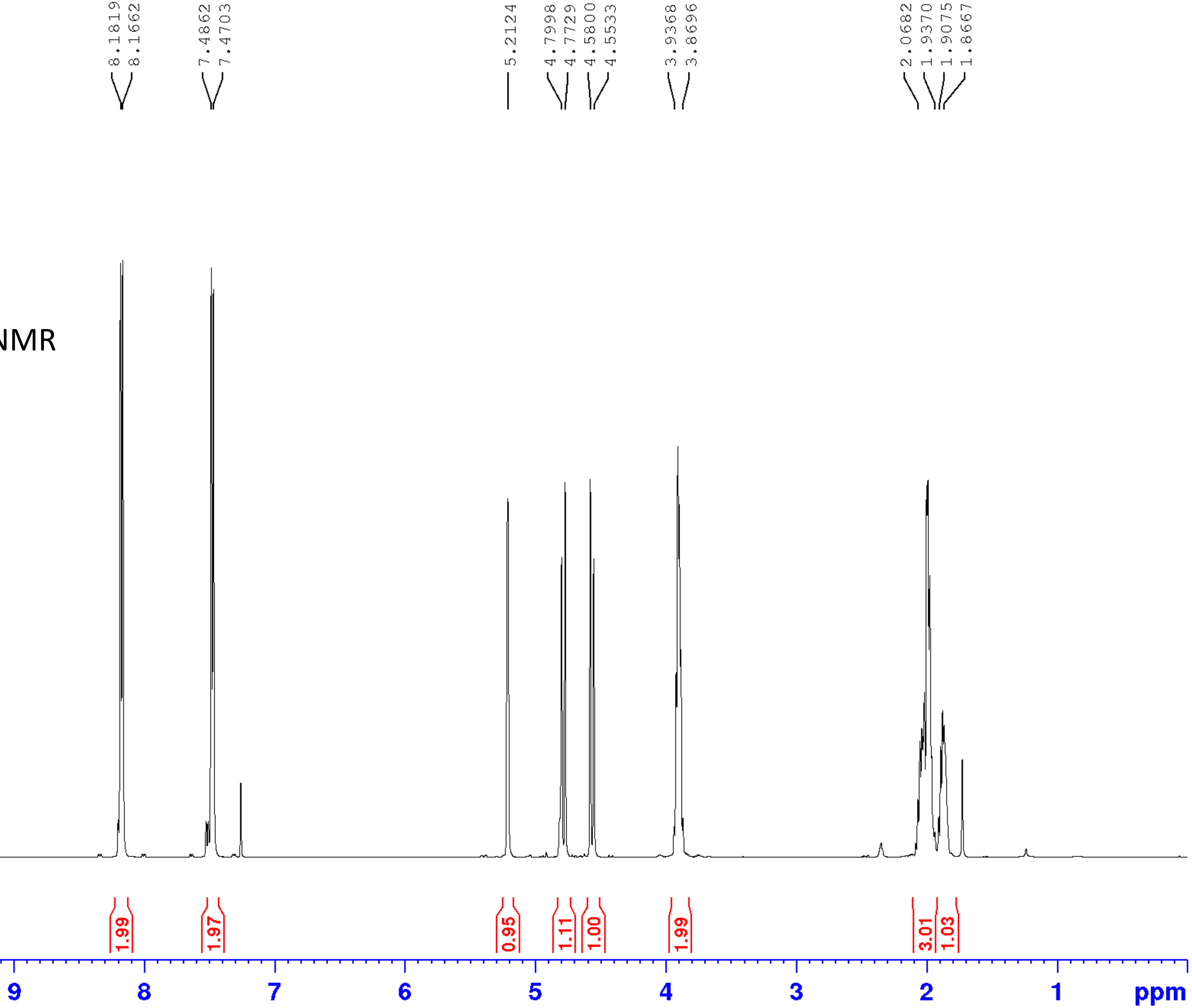


```
Current Data Parameters
NAME      vinn-4-137-11-islt-2020121
EXPNO                     1
PROCNO                    1

F2 - Acquisition Parameters
Date_              20201211
Time                 6.05 h
INSTRUM               spect
PROBHD     Z119470_0283 (
PULPROG                zg30
TD                    65536
SOLVENT               CDCl3
NS                       16
DS                        2
SWH              10000.000 Hz
FIDRES            0.305176 Hz
AQ               3.2767999 sec
RG                    30.85
DW                   50.000 usec
DE                     6.50 usec
TE                    295.1 K
D1               1.00000000 sec
TD0                       1
SFO1            500.1330883 MHz
NUC1                     1H
P1                    10.91 usec
PLW1            25.00000000 W

F2 - Processing parameters
SI                    65536
SF              500.1300121 MHz
WDW                      EM
SSB                       0
LB                     0.30 Hz
GB                        0
PC                     1.00
```

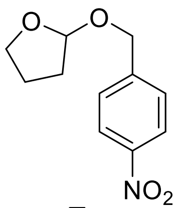

5e

125 MHz ^{13}C NMR

$CDCl_3$

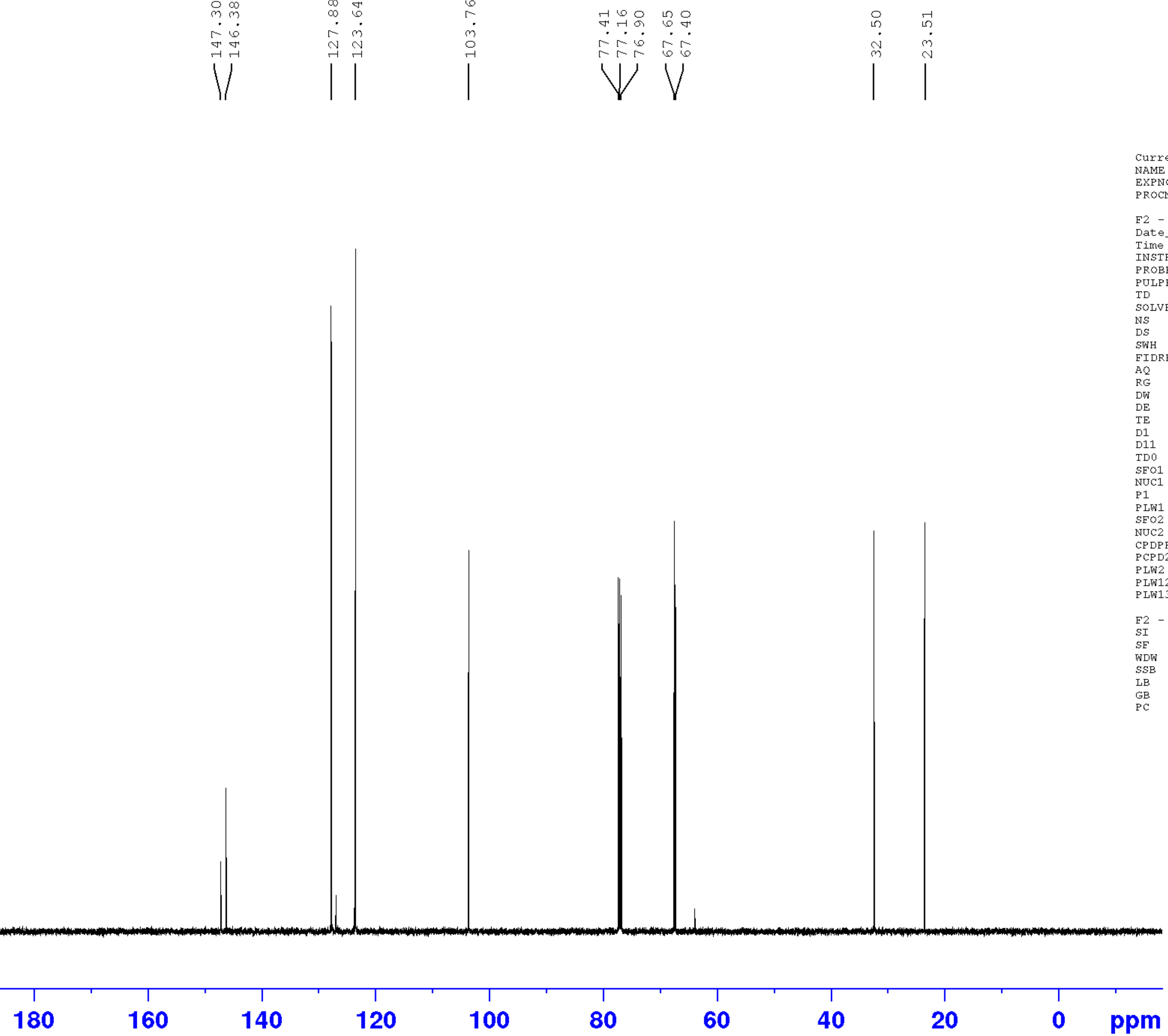


```
Current Data Parameters
NAME      vinn-4-137-11-islt-20201210
EXPNO                     2
PROCNO                    1

F2 - Acquisition Parameters
Date_              20201211
Time                 6.11 h
INSTRUM               spect
PROBHD    Z119470_0283 (
PULPROG             zgpg30
TD                    65536
SOLVENT               CDCl3
NS                      100
DS                        4
SWH              29761.904 Hz
FIDRES            0.908261 Hz
AQ               1.1010048 sec
RG                   206.72
DW                   16.800 usec
DE                     6.50 usec
TE                    295.2 K
D1               2.00000000 sec
D11              0.03000000 sec
TD0                       1
SFO1            125.7703643 MHz
NUC1                    13C
P1                     9.75 usec
PLW1            94.00000000 W
SFO2            500.1320005 MHz
NUC2                     1H
CPDPRG[2            waltz16
PCPD2                 80.00 usec
PLW2            25.00000000 W
PLW12            0.46495000 W
PLW13            0.23387000 W

F2 - Processing parameters
SI                    32768
SF              125.7577786 MHz
WDW                      EM
SSB                       0
LB                     1.00 Hz
GB                        0
PC                     1.40
```

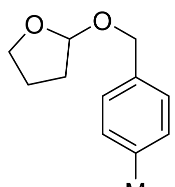

5f

500 MHz ^{1}H NMR

$CDCl_3$

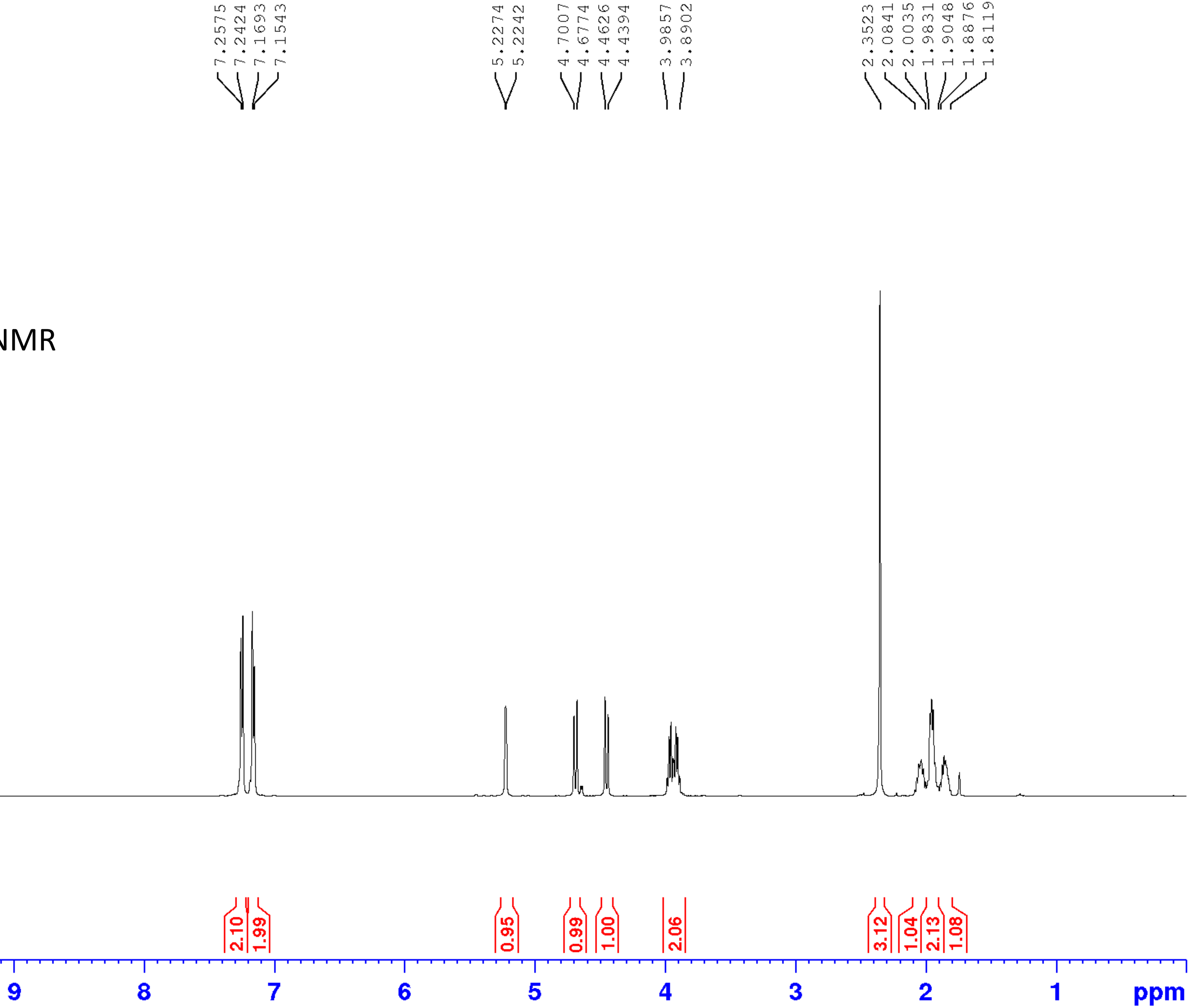

BRUKER

```
Current Data Parameters
NAME      vinn-4-137-10-islt-2020121
EXPNO                   1
PROCNO                  1

F2 - Acquisition Parameters
Date_             20201211
Time                5.56 h
INSTRUM              spect
PROBHD    Z119470_0283 (
PULPROG               zg30
TD                   65536
SOLVENT              CDCl3
NS                      16
DS                       2
SWH            10000.000 Hz
FIDRES          0.305176 Hz
AQ             3.2767999 sec
RG                   30.85
DW                  50.000 usec
DE                   6.50 usec
TE                  295.1 K
D1            1.00000000 sec
TD0                      1
SFO1         500.1330883 MHz
NUC1                    1H
P1                   10.91 usec
PLW1         25.00000000 W

F2 - Processing parameters
SI                   65536
SF           500.1300122 MHz
WDW                     EM
SSB                      0
LB                    0.30 Hz
GB                       0
PC                    1.00
```

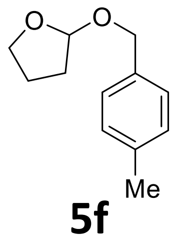

5f

125 MHz ^{13}C NMR

$CDCl_3$

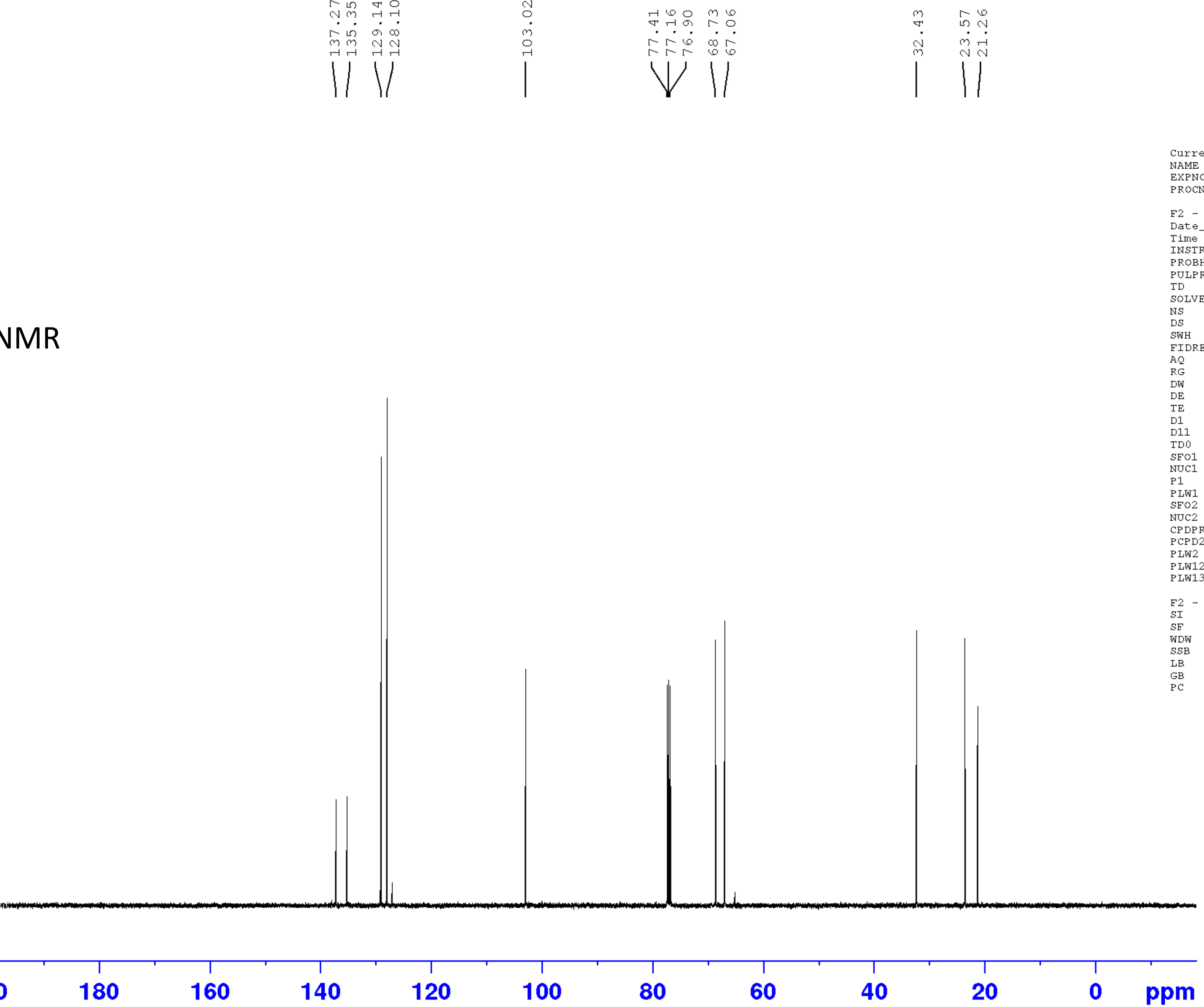


```
Current Data Parameters
NAME     vinn-4-137-10-islt-20201210
EXPNO                    2
PROCNO                   1

F2 - Acquisition Parameters
Date_             20201211
Time                6.02 h
INSTRUM              spect
PROBHD    Z119470_0283 (
PULPROG             zgpg30
TD                   65536
SOLVENT              CDCl3
NS                     100
DS                       4
SWH              29761.904 Hz
FIDRES            0.908261 Hz
AQ               1.1010048 sec
RG                  206.72
DW                  16.800 usec
DE                    6.50 usec
TE                   295.1 K
D1              2.00000000 sec
D11             0.03000000 sec
TD0                      1
SFO1           125.7703643 MHz
NUC1                   13C
P1                    9.75 usec
PLW1           94.00000000 W
SFO2           500.1320005 MHz
NUC2                    1H
CPDPRG[2           waltz16
PCPD2                80.00 usec
PLW2           25.00000000 W
PLW12           0.46495000 W
PLW13           0.23387000 W

F2 - Processing parameters
SI                   32768
SF             125.7577807 MHz
WDW                     EM
SSB                      0
LB                    1.00 Hz
GB                       0
PC                    1.40
```

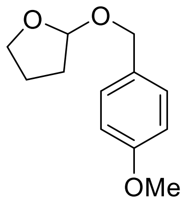

5g

500 MHz ^{1}H NMR

$CDCl_3$

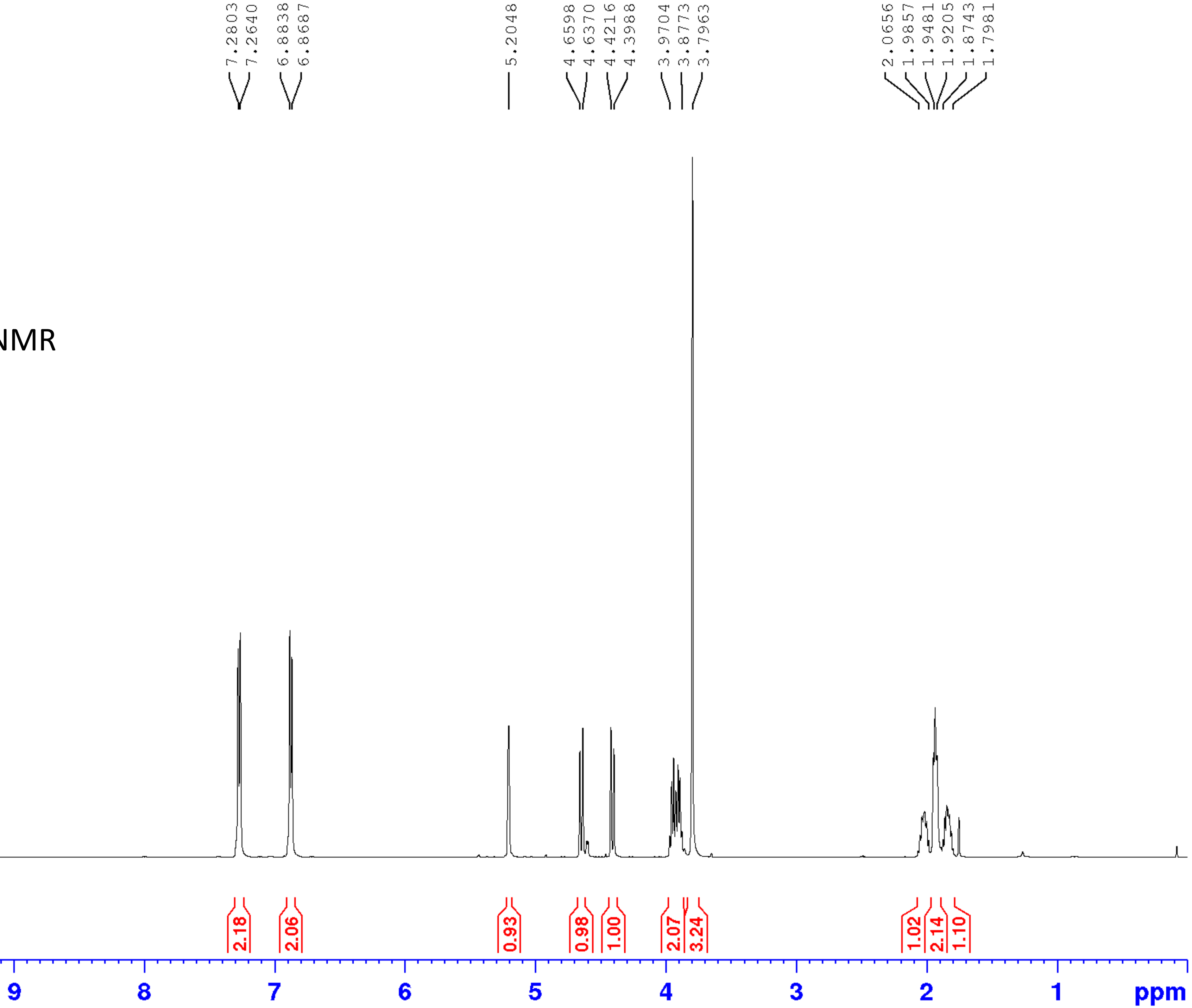


```
Current Data Parameters
NAME      vinn-4-137-13-islt-2020121
EXPNO                      1
PROCNO                     1

F2 - Acquisition Parameters
Date_              20201211
Time                 6.24 h
INSTRUM               spect
PROBHD      Z119470_0283 (
PULPROG                zg30
TD                    65536
SOLVENT               CDCl3
NS                       16
DS                        2
SWH             10000.000 Hz
FIDRES           0.305176 Hz
AQ              3.2767999 sec
RG                   30.85
DW                  50.000 usec
DE                    6.50 usec
TE                   295.1 K
D1             1.00000000 sec
TD0                      1
SFO1          500.1330883 MHz
NUC1                    1H
P1                   10.91 usec
PLW1          25.00000000 W

F2 - Processing parameters
SI                    65536
SF            500.1300121 MHz
WDW                      EM
SSB                       0
LB                     0.30 Hz
GB                        0
PC                     1.00
```

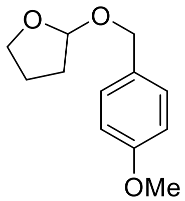

5g

125 MHz ^{13}C NMR

$CDCl_3$

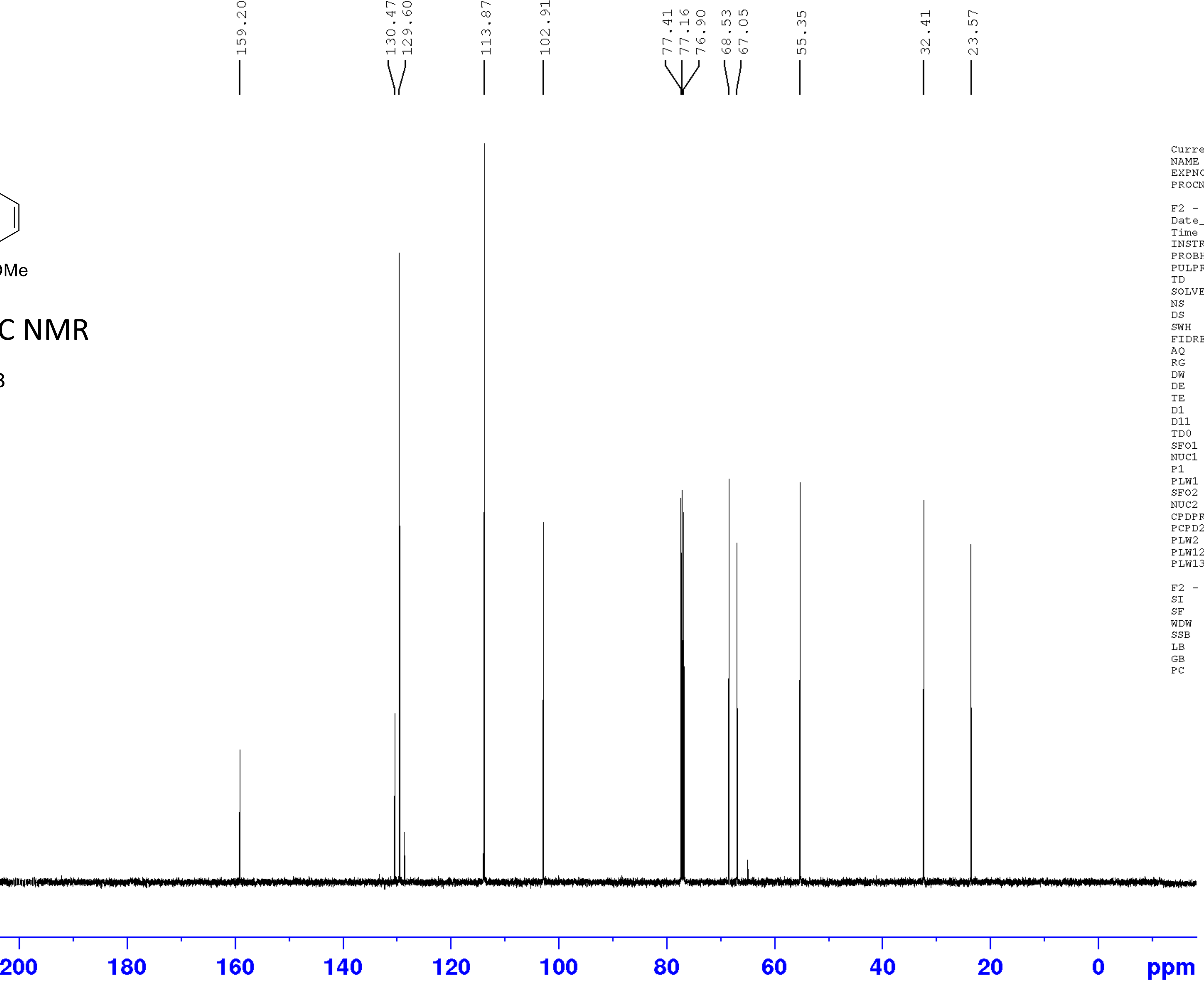

Current Data Parameters
NAME vinn-4-137-13-islt-20201210
EXPNO 2
PROCNO 1

F2 - Acquisition Parameters
Date_ 20201211
Time 6.30 h
INSTRUM spect
PROBHD Z119470_0283 (
PULPROG zgpg30
TD 65536
SOLVENT CDCl3
NS 100
DS 4
SWH 29761.904 Hz
FIDRES 0.908261 Hz
AQ 1.1010048 sec
RG 206.72
DW 16.800 usec
DE 6.50 usec
TE 295.2 K
D1 2.00000000 sec
D11 0.03000000 sec
TD0 1
SFO1 125.7703643 MHz
NUC1 13C
P1 9.75 usec
PLW1 94.00000000 W
SFO2 500.1320005 MHz
NUC2 1H
CPDPRG[2 waltz16
PCPD2 80.00 usec
PLW2 25.00000000 W
PLW12 0.46495000 W
PLW13 0.23387000 W

F2 - Processing parameters
SI 32768
SF 125.7577796 MHz
WDW EM
SSB 0
LB 1.00 Hz
GB 0
PC 1.40

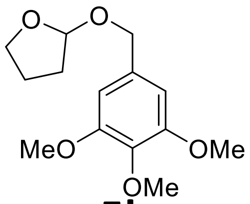

5h

500 MHz ^{1}H NMR

$CDCl_3$

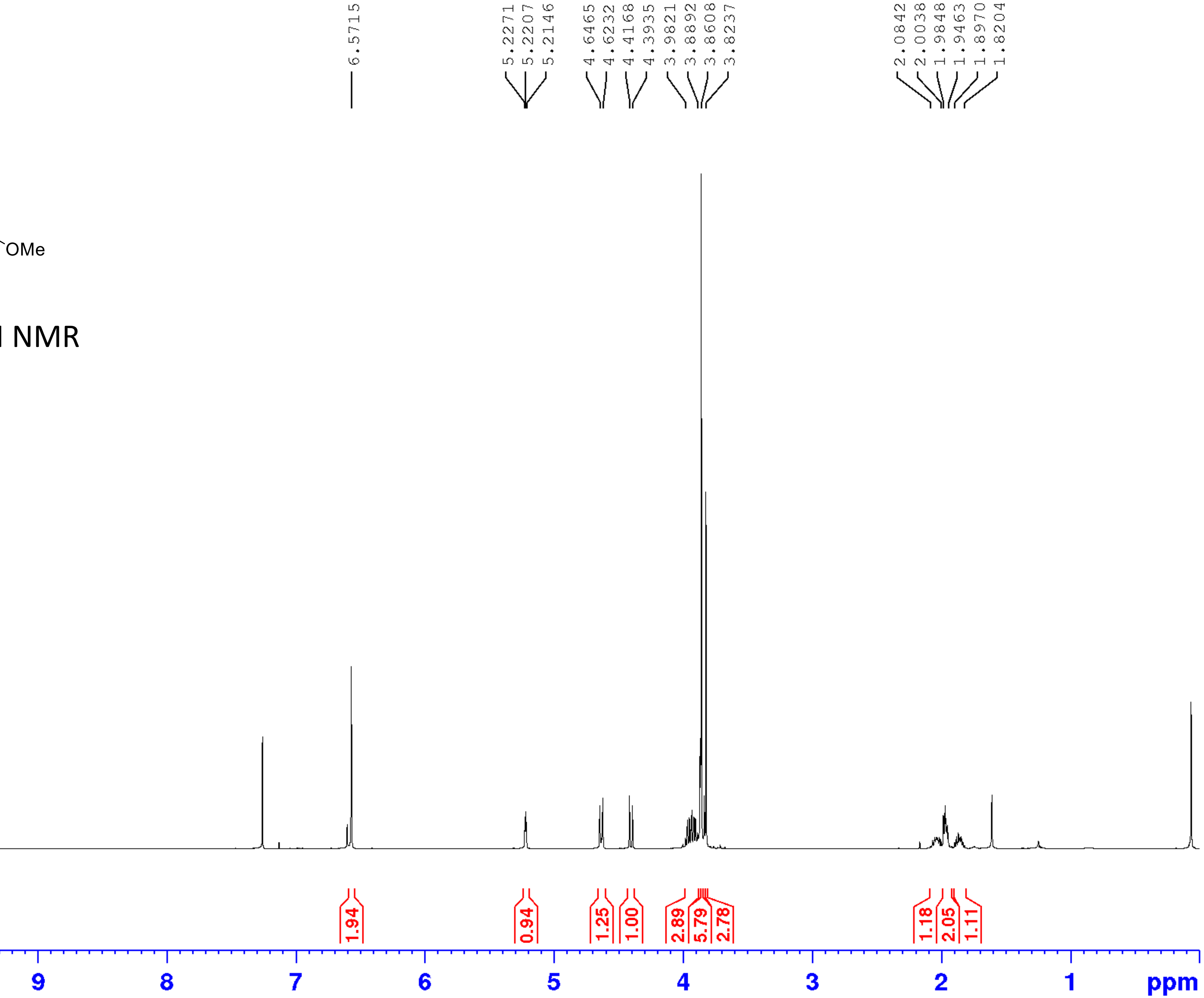

BRUKER

Current Data Parameters
NAME vinn-4-137-12-islt2-20200120
EXPNO 1
PROCNO 1

F2 - Acquisition Parameters
Date_ 20210121
Time 8.57 h
INSTRUM spect
PROBHD Z119470_0283 (
PULPROG zg30
TD 65536
SOLVENT CDCl3
NS 16
DS 2
SWH 10000.000 Hz
FIDRES 0.305176 Hz
AQ 3.2767999 sec
RG 93.28
DW 50.000 usec
DE 6.50 usec
TE 295.1 K
D1 1.00000000 sec
TD0 1
SFO1 500.1330883 MHz
NUC1 1H
P1 10.91 usec
PLW1 25.00000000 W

F2 - Processing parameters
SI 65536
SF 500.1300126 MHz
WDW EM
SSB 0
LB 0.30 Hz
GB 0
PC 1.00

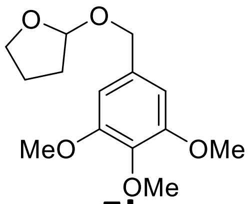

5h

125 MHz ^{13}C NMR

$CDCl_3$

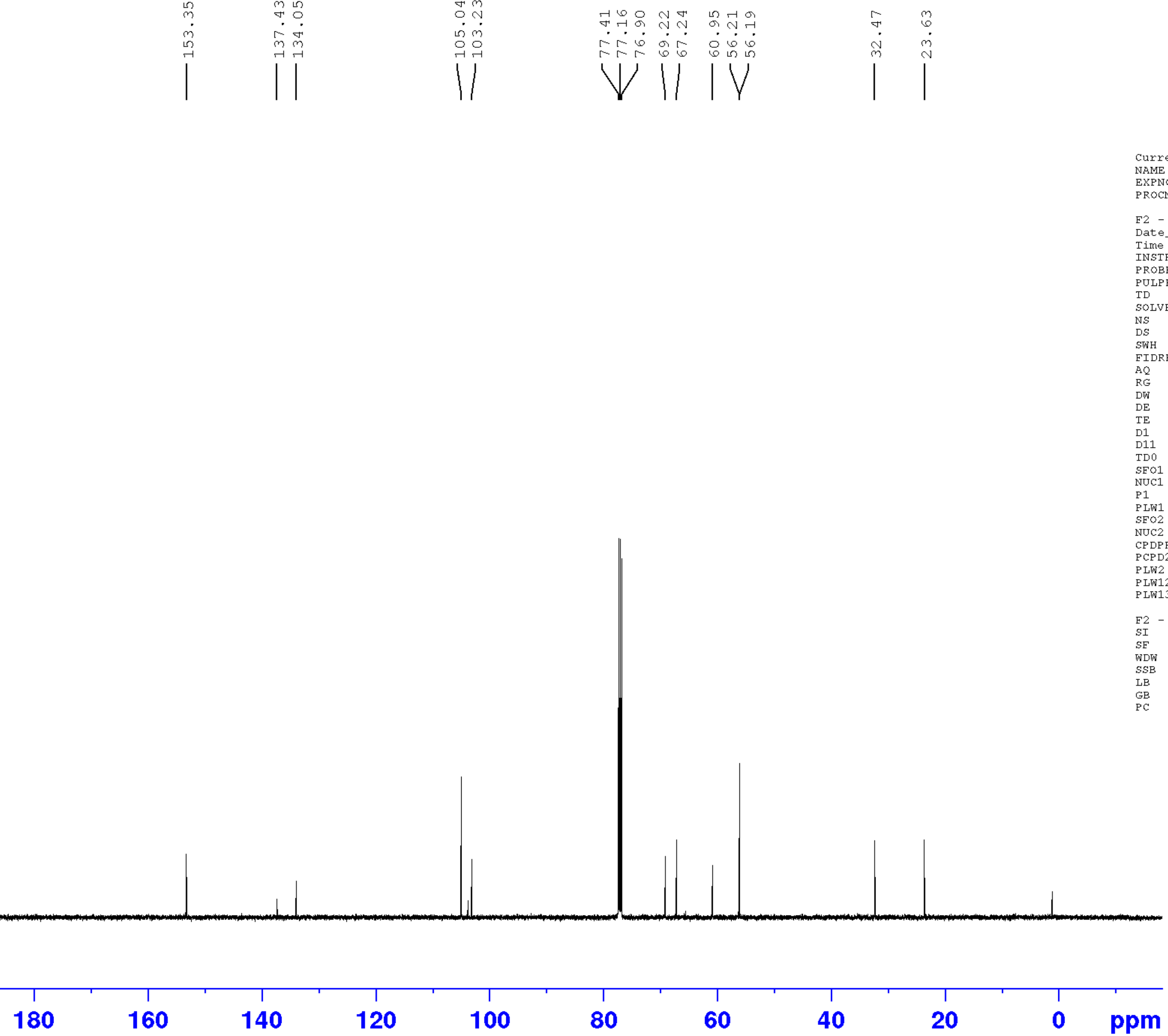


```
Current Data Parameters
NAME      vinn-4-137-12-islt2-2020012(
EXPNO                  2
PROCNO                 1

F2 - Acquisition Parameters
Date_            20210121
Time                 9.10 h
INSTRUM             spect
PROBHD   Z119470_0283 (
PULPROG           zgpg30
TD                 65536
SOLVENT            CDCl3
NS                   256
DS                     4
SWH            29761.904 Hz
FIDRES          0.908261 Hz
AQ             1.1010048 sec
RG                206.72
DW                16.800 usec
DE                  6.50 usec
TE                 295.1 K
D1            2.00000000 sec
D11           0.03000000 sec
TD0                    1
SFO1         125.7703643 MHz
NUC1                 13C
P1                  9.75 usec
PLW1         94.00000000 W
SFO2         500.1320005 MHz
NUC2                  1H
CPDPRG[2         waltz16
PCPD2              80.00 usec
PLW2         25.00000000 W
PLW12         0.46495000 W
PLW13         0.23387000 W

F2 - Processing parameters
SI                 32768
SF           125.7577741 MHz
WDW                   EM
SSB                    0
LB                  1.00 Hz
GB                     0
PC                  1.40
```

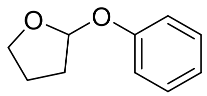

5i

500 MHz ^{1}H NMR

$CDCl_3$

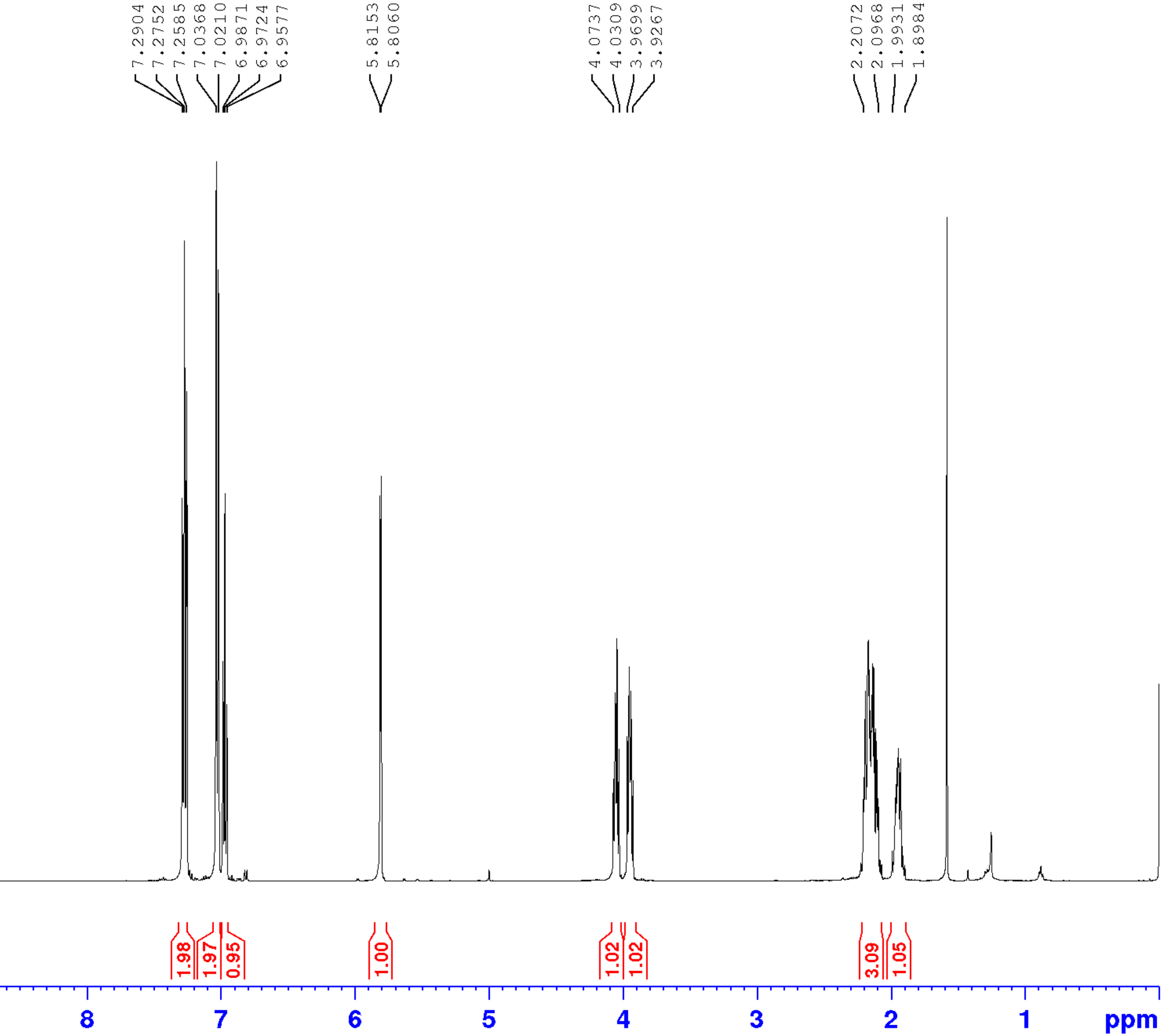


```
Current Data Parameters
NAME     vinn-4-193-islt-20230105
EXPNO                       1
PROCNO                      1

F2 - Acquisition Parameters
Date_            20230105
Time                16.35 h
INSTRUM             spect
PROBHD   Z119470_0283 (
PULPROG              zg30
TD                  65536
SOLVENT             CDCl3
NS                     16
DS                      2
SWH             10000.000 Hz
FIDRES           0.305176 Hz
AQ              3.2767999 sec
RG                 117.01
DW                 50.000 usec
DE                   6.50 usec
TE                  295.2 K
D1             1.00000000 sec
TD0                     1
SFO1          500.1330883 MHz
NUC1                   1H
P1                  10.91 usec
PLW1          25.00000000 W

F2 - Processing parameters
SI                  65536
SF            500.1300150 MHz
WDW                    EM
SSB                     0
LB                   0.30 Hz
GB                      0
PC                   1.00
```

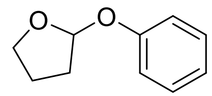

5i

125 MHz ^{13}C NMR

$CDCl_3$

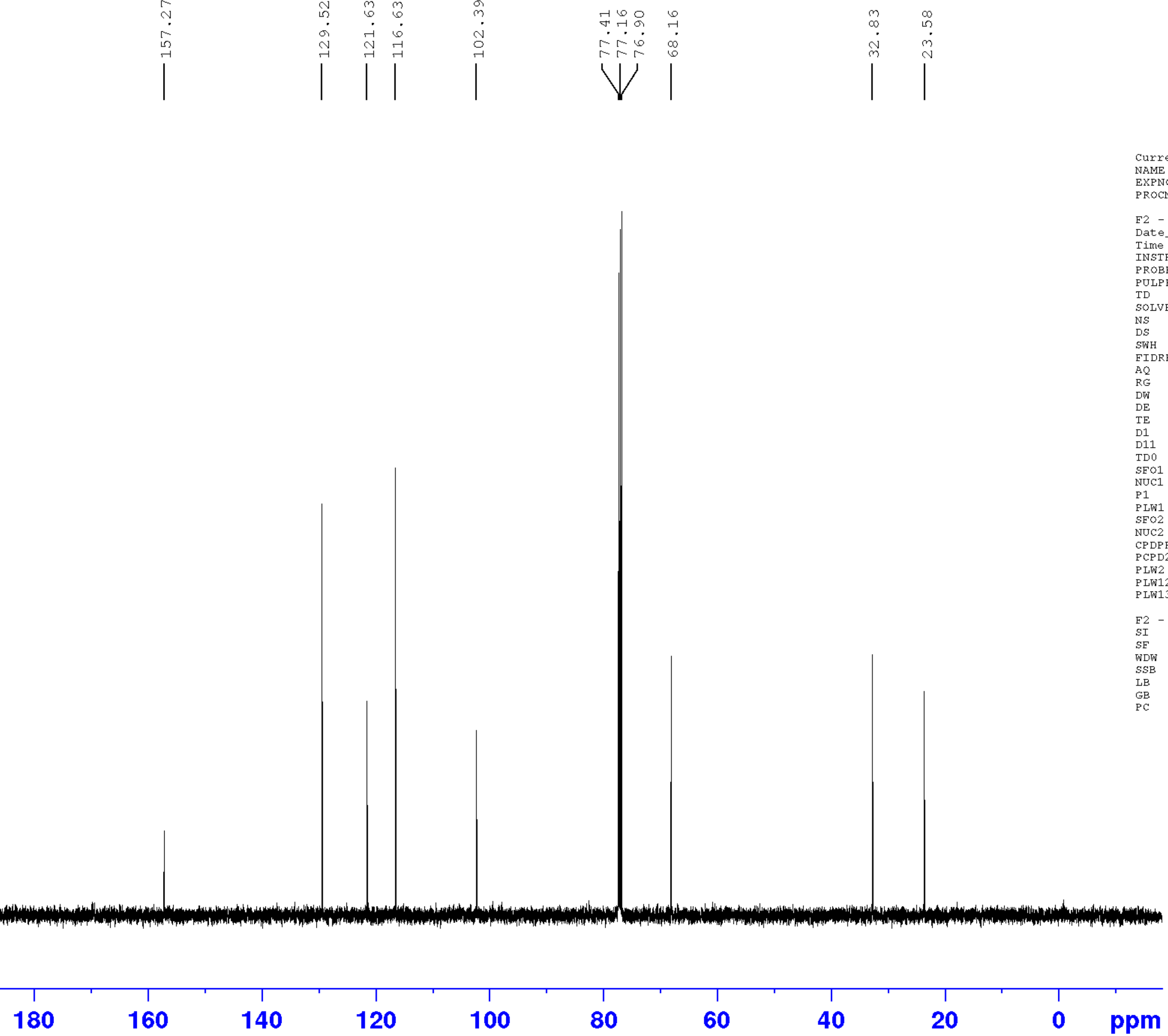


```
Current Data Parameters
NAME      vinn-4-193-islt-20230105
EXPNO                        2
PROCNO                       1

F2 - Acquisition Parameters
Date_              20230105
Time                  16.39 h
INSTRUM               spect
PROBHD   Z119470_0283 (
PULPROG              zgpg30
TD                    65536
SOLVENT               CDCl3
NS                       50
DS                        4
SWH              29761.904 Hz
FIDRES            0.908261 Hz
AQ               1.1010048 sec
RG                   206.72
DW                   16.800 usec
DE                     6.50 usec
TE                    295.1 K
D1               2.00000000 sec
D11              0.03000000 sec
TD0                       1
SFO1            125.7703643 MHz
NUC1                    13C
P1                     9.75 usec
PLW1            94.00000000 W
SFO2            500.1320005 MHz
NUC2                     1H
CPDPRG[2            waltz16
PCPD2                 80.00 usec
PLW2            25.00000000 W
PLW12            0.46495000 W
PLW13            0.23387000 W

F2 - Processing parameters
SI                    32768
SF              125.7577746 MHz
WDW                      EM
SSB                       0
LB                     1.00 Hz
GB                        0
PC                     1.40
```

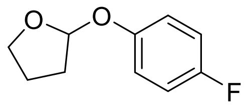

5j
500 MHz ^{1}H NMR
$CDCl_3$

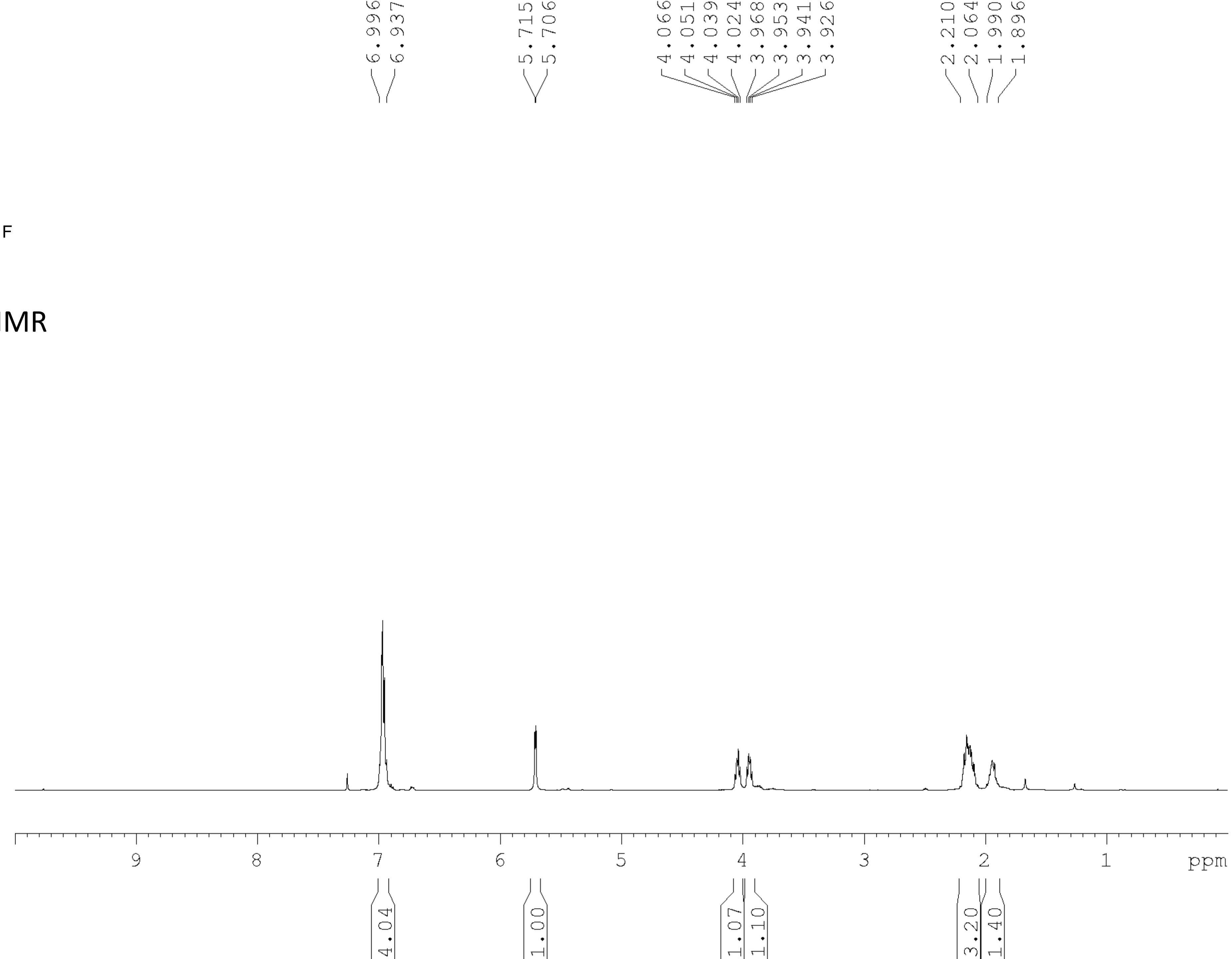

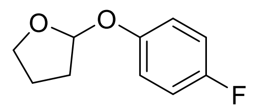

5j
125 MHz ^{13}C NMR
$CDCl_3$

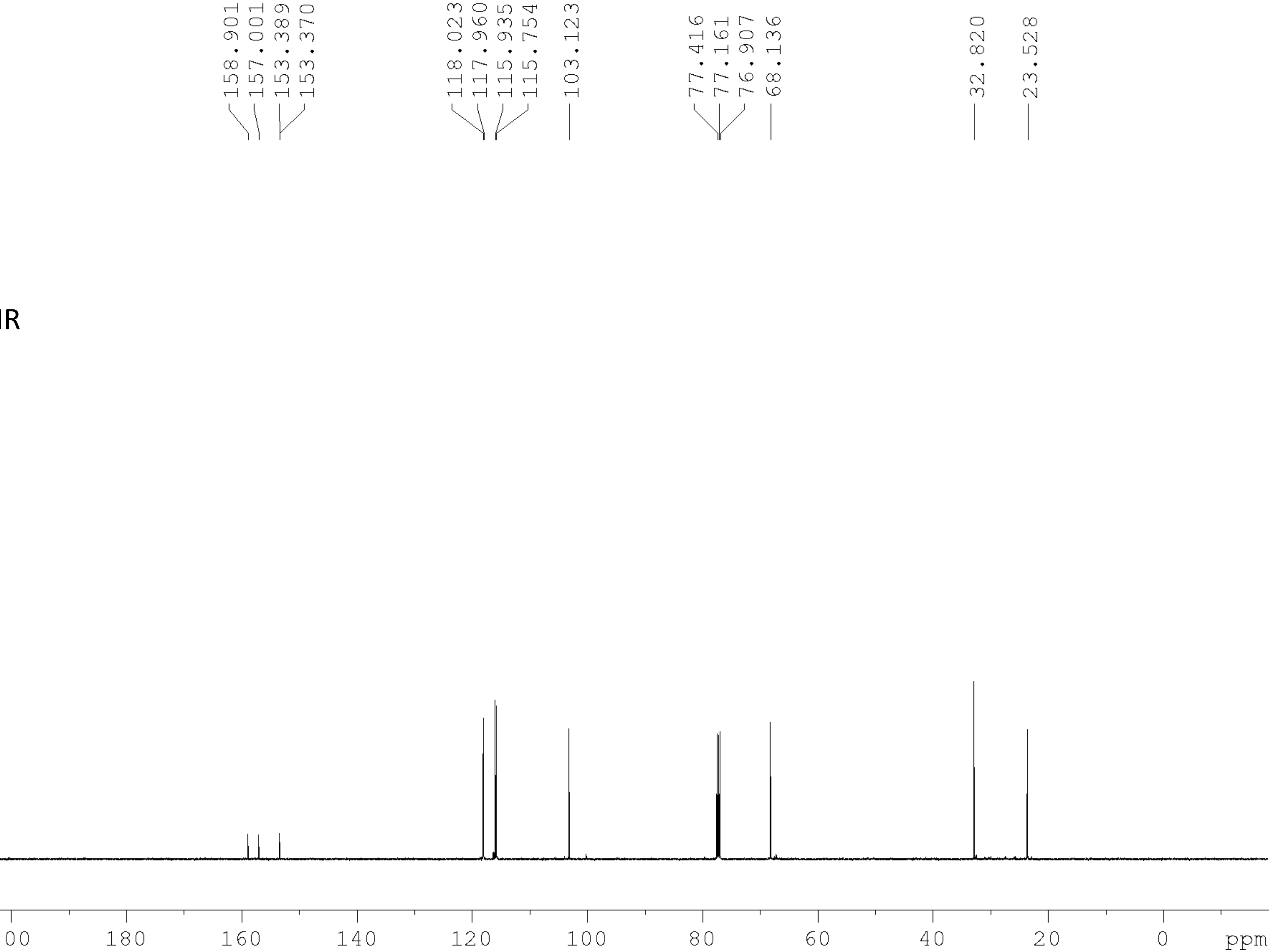

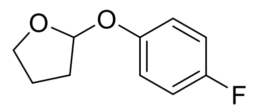

5j

470 MHz ^{1}H NMR

$CDCl_3$

```
Current Data Parameters
NAME      vinn-4-147-3-islt-20200120
EXPNO                    3
PROCNO                   1

F2 - Acquisition Parameters
Date_            20210121
Time              18.34 h
INSTRUM             spect
PROBHD   Z119470_0283 (
PULPROG            zgflqn
TD                 131072
SOLVENT             CDCl3
NS                     16
DS                      4
SWH           113636.367 Hz
FIDRES          1.733953 Hz
AQ             0.5767168 sec
RG                 206.72
DW                  4.400 usec
DE                   6.50 usec
TE                  295.1 K
D1            1.00000000 sec
TD0                     1
SFO1         470.5453180 MHz
NUC1                  19F
P1                  15.00 usec
PLW1          47.23500061 W

F2 - Processing parameters
SI                  65536
SF           470.5923772 MHz
WDW                    EM
SSB                     0
LB                   0.30 Hz
GB                      0
PC                   1.00
```

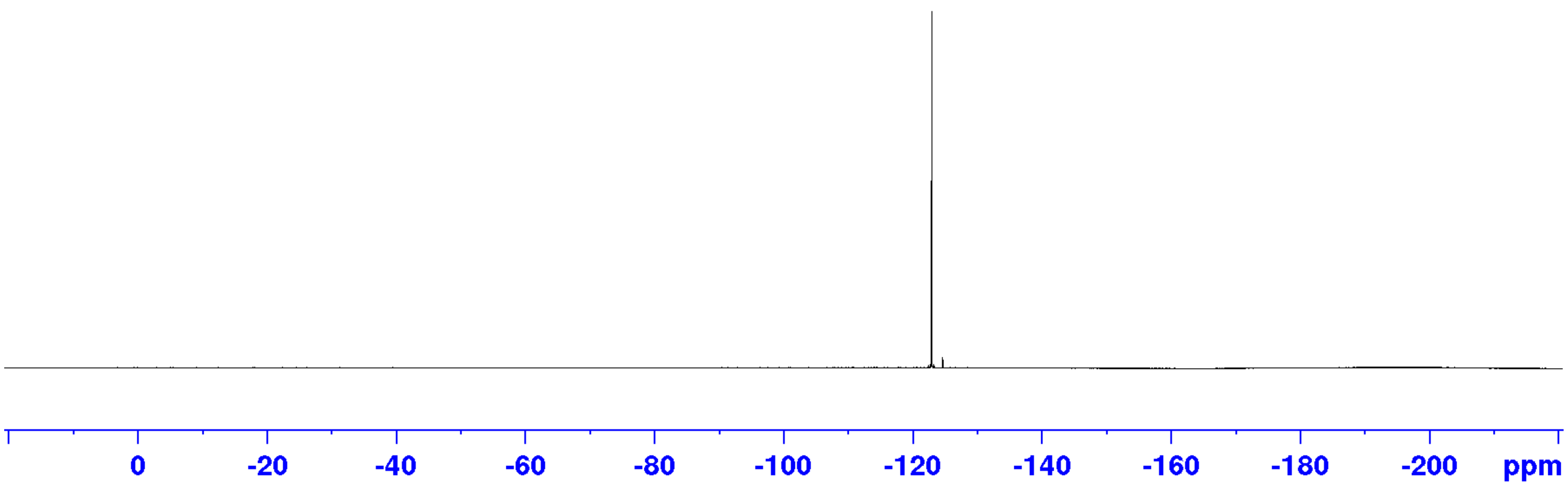

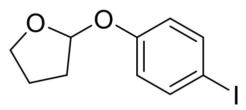

5k

500 MHz ^{1}H NMR

$CDCl_3$

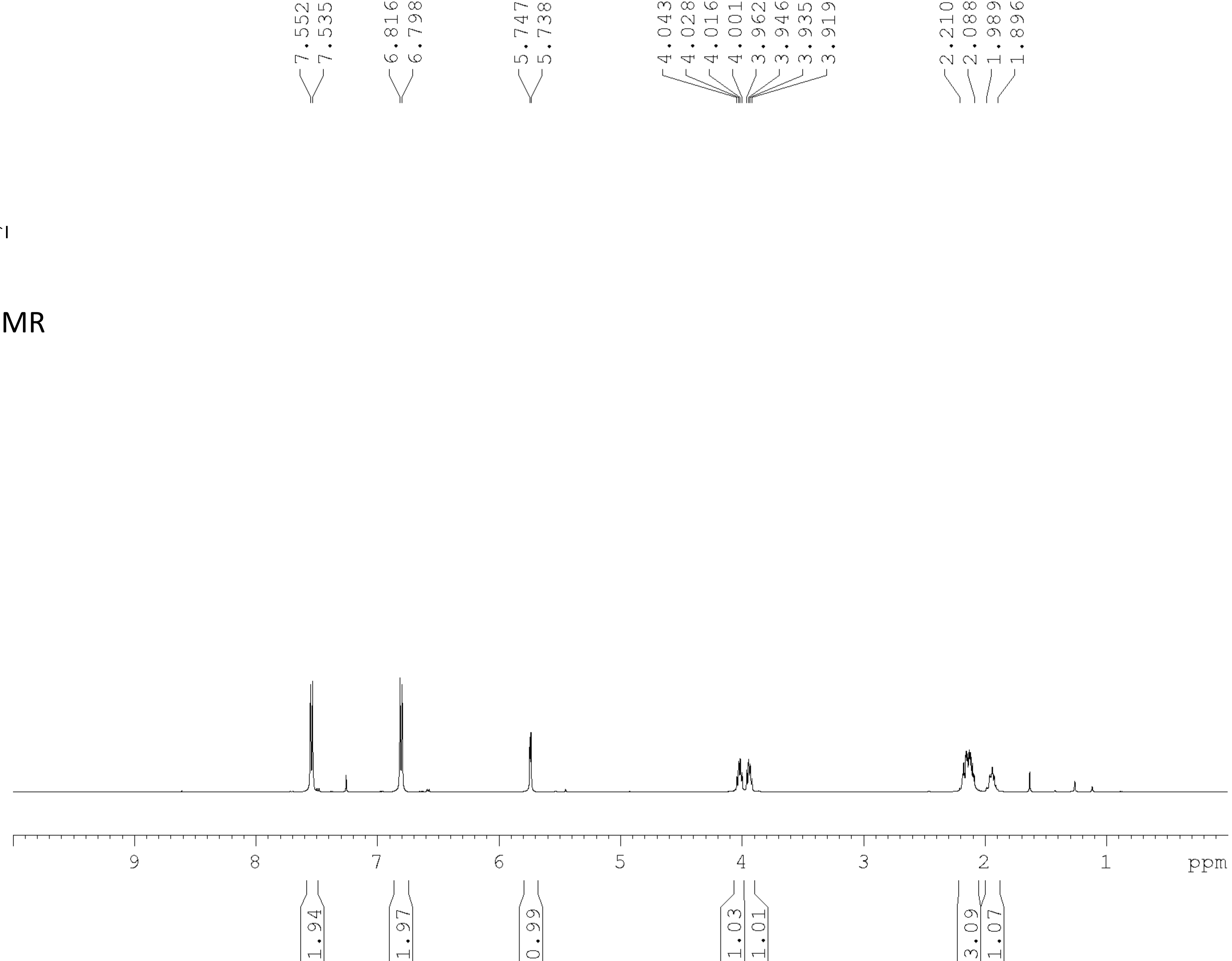

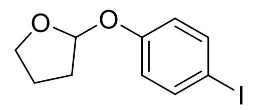

5k
125 MHz ^{13}C NMR
$CDCl_3$

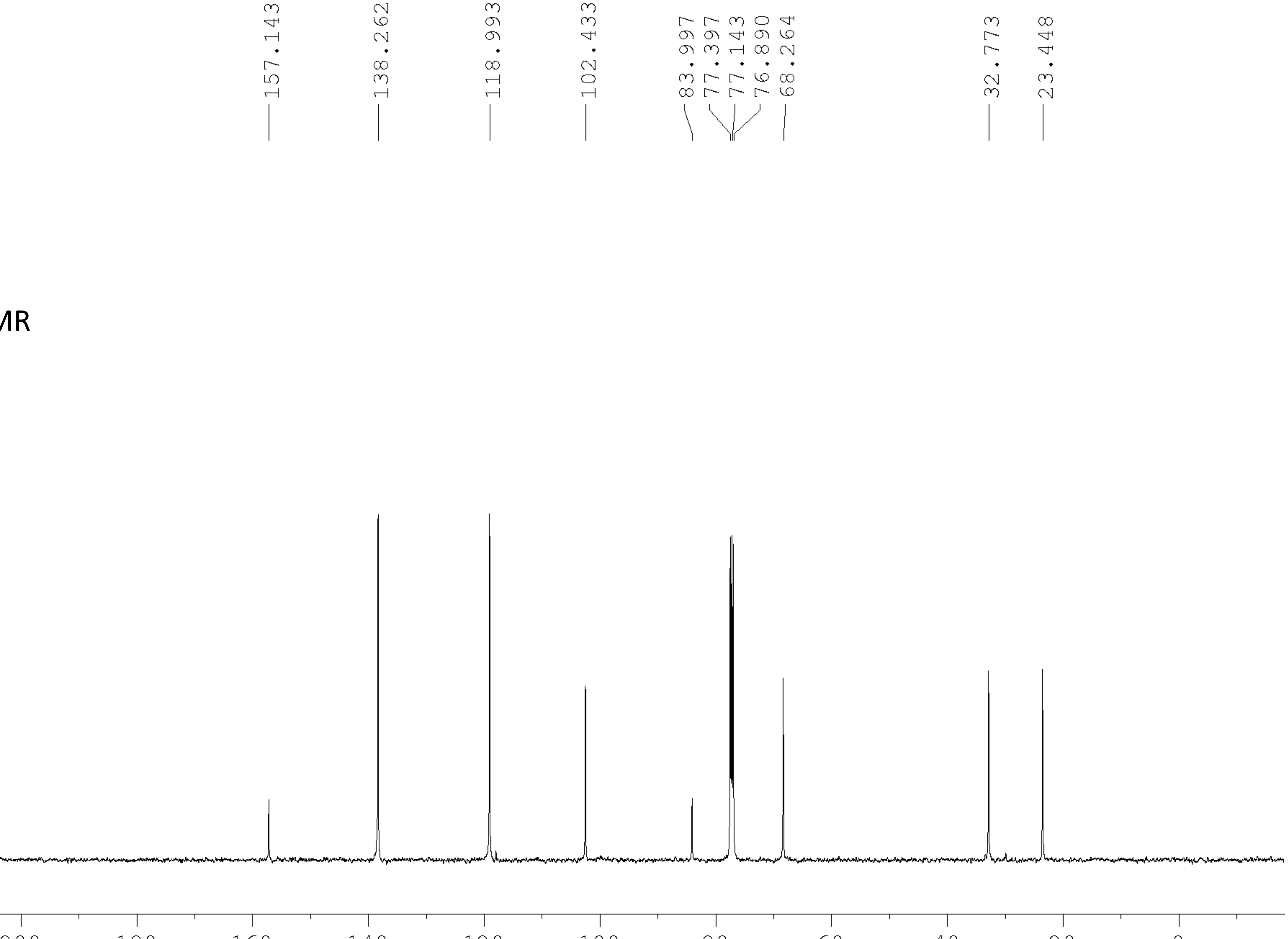

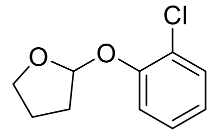

5l

500 MHz ^{1}H NMR

$CDCl_3$

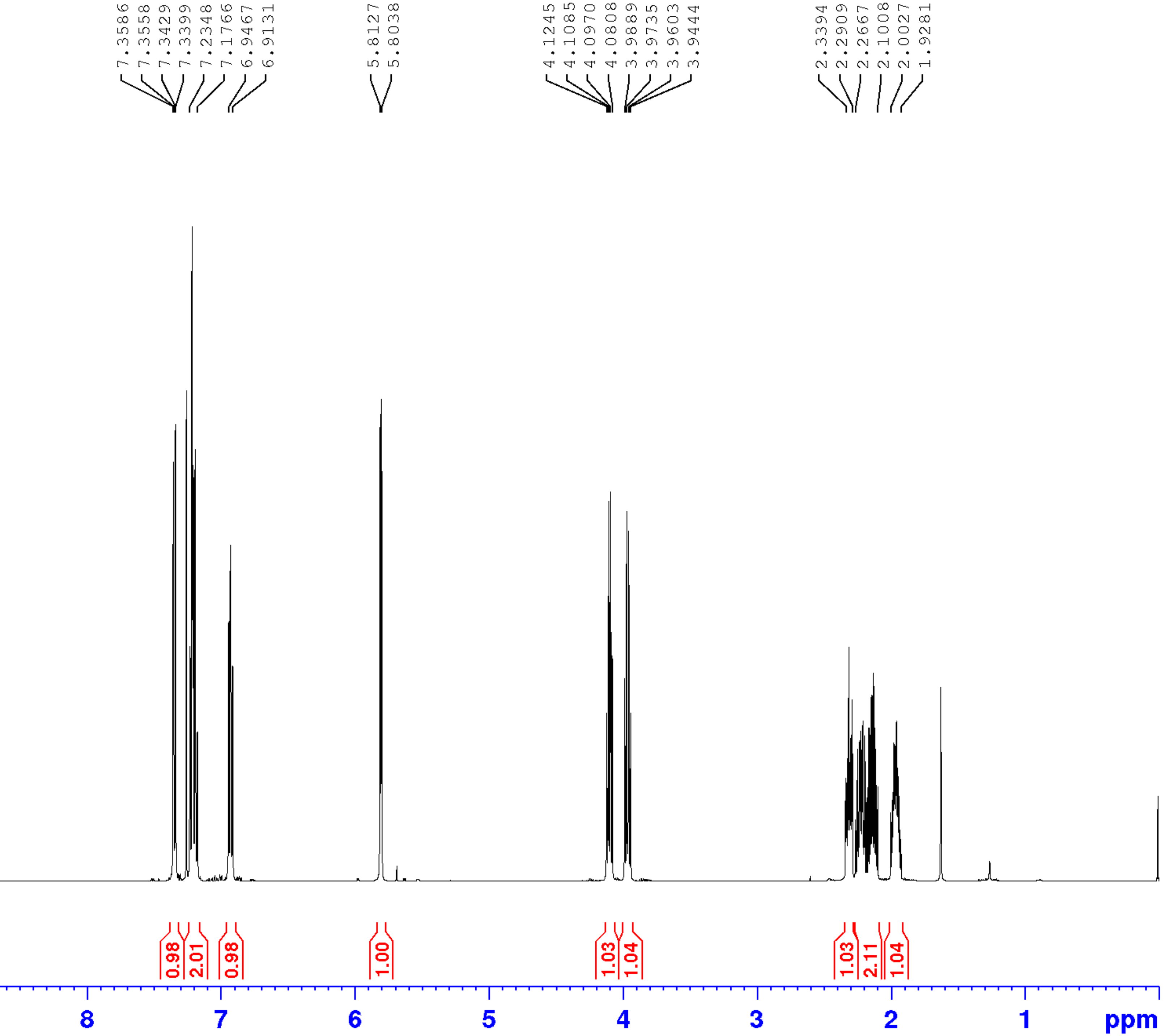


```
Current Data Parameters
NAME     vinn-4-183-3-islt2-2023032
EXPNO                    1
PROCNO                   1

F2 - Acquisition Parameters
Date_             20230321
Time                 15.51 h
INSTRUM              spect
PROBHD   Z149001_0010 (
PULPROG               zg30
TD                   65536
SOLVENT              CDCl3
NS                      16
DS                       2
SWH              10000.000 Hz
FIDRES            0.305176 Hz
AQ               3.2767999 sec
RG                   30.85
DW                  50.000 usec
DE                   10.00 usec
TE                   296.1 K
D1              1.00000000 sec
TD0                      1
SFO1           500.1330883 MHz
NUC1                    1H
P1                   11.25 usec
PLW1           17.35199928 W

F2 - Processing parameters
SI                   65536
SF             500.1300124 MHz
WDW                     EM
SSB                      0
LB                    0.30 Hz
GB                       0
PC                    1.00
```

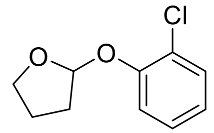

5l

125 MHz ^{13}C NMR

$CDCl_3$

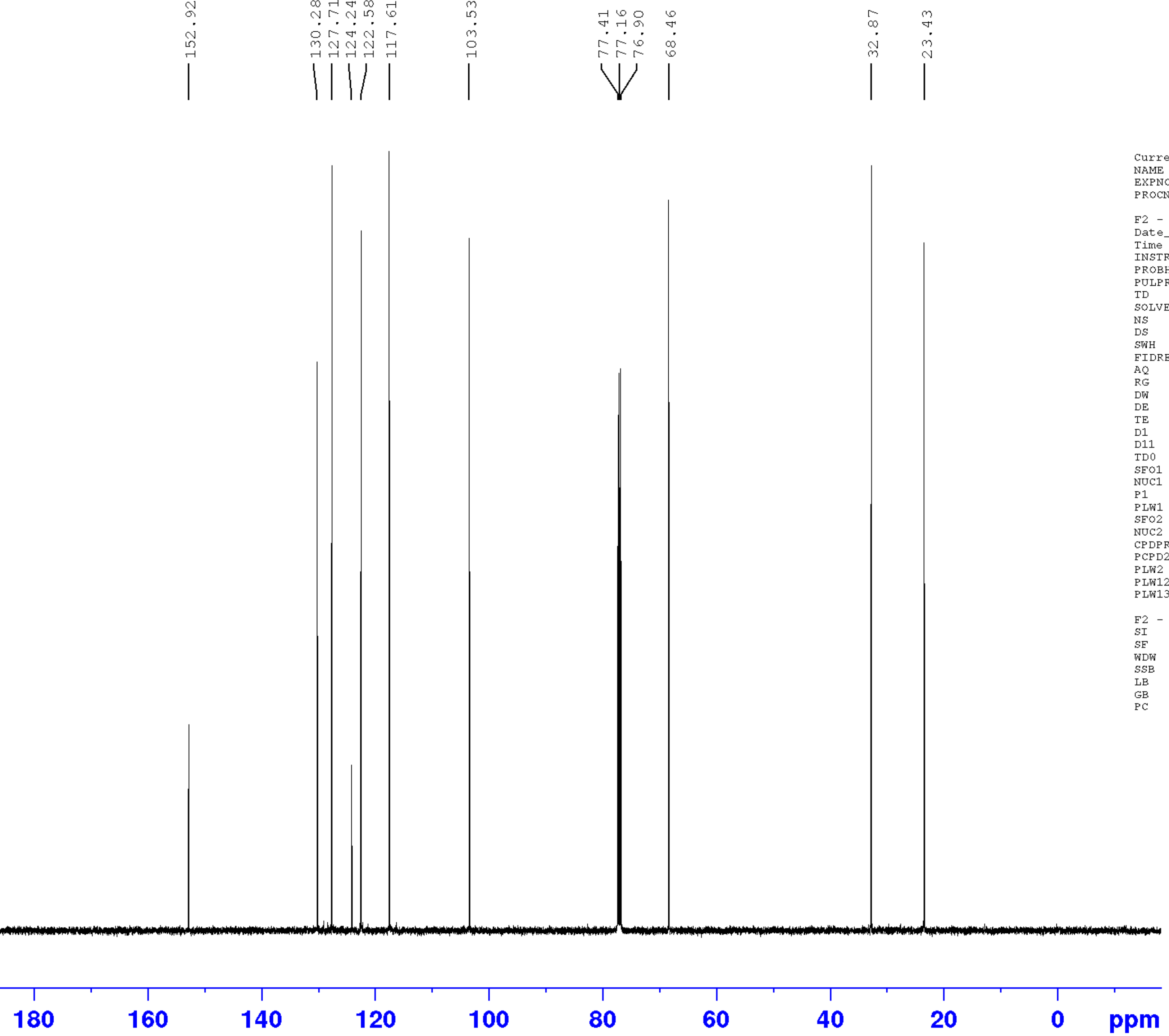


```
Current Data Parameters
NAME     vinn-4-183-3-islt2-20230321
EXPNO                  2
PROCNO                 1

F2 - Acquisition Parameters
Date_            20230321
Time                15.53 h
INSTRUM             spect
PROBHD   Z149001_0010 (
PULPROG            zgpg30
TD                  65536
SOLVENT             CDCl3
NS                     30
DS                      4
SWH             29761.904 Hz
FIDRES           0.908261 Hz
AQ              1.1010048 sec
RG                 206.72
DW                 16.800 usec
DE                  18.00 usec
TE                  296.1 K
D1             2.00000000 sec
D11            0.03000000 sec
TD0                     1
SFO1          125.7703643 MHz
NUC1                  13C
P1                  10.00 usec
PLW1          73.80999756 W
SFO2          500.1320005 MHz
NUC2                   1H
CPDPRG[2          waltz16
PCPD2               80.00 usec
PLW2          17.35199928 W
PLW12          0.34314999 W
PLW13          0.17260000 W

F2 - Processing parameters
SI                  32768
SF            125.7577783 MHz
WDW                    EM
SSB                     0
LB                   1.00 Hz
GB                      0
PC                   1.40
```

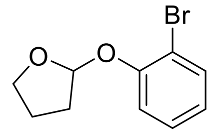

5m

500 MHz ^{1}H NMR

$CDCl_3$

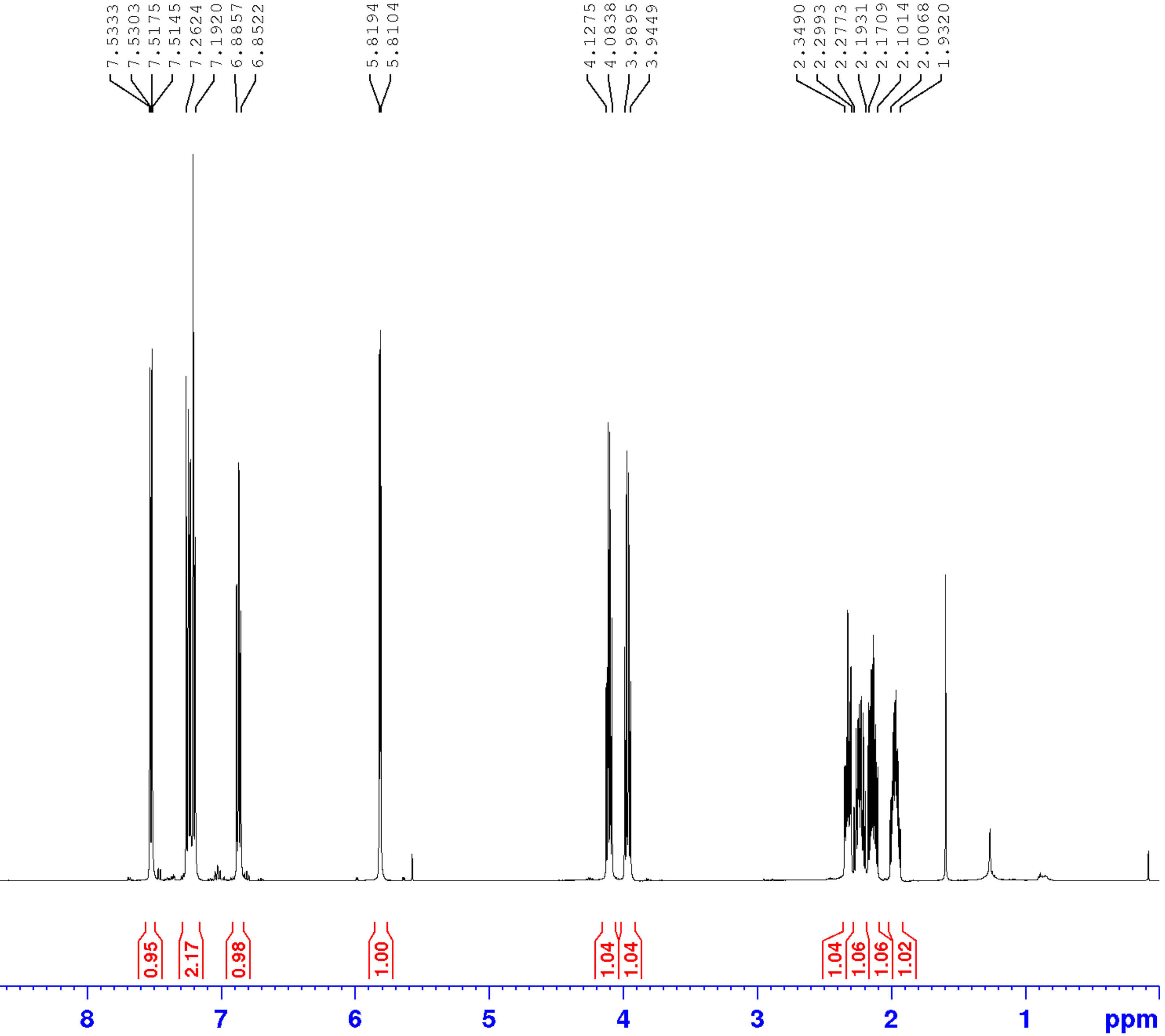


```
Current Data Parameters
NAME      vinn-4-147-1-islt-20200121
EXPNO                    1
PROCNO                   1

F2 - Acquisition Parameters
Date_             20210121
Time               19.07 h
INSTRUM            spect
PROBHD    Z119470_0283 (
PULPROG              zg30
TD                  65536
SOLVENT             CDCl3
NS                     16
DS                      2
SWH          10000.000 Hz
FIDRES        0.305176 Hz
AQ           3.2767999 sec
RG                  63.76
DW              50.000 usec
DE               6.50 usec
TE              295.2 K
D1         1.00000000 sec
TD0                     1
SFO1      500.1330883 MHz
NUC1                   1H
P1              10.91 usec
PLW1       25.00000000 W

F2 - Processing parameters
SI                  65536
SF        500.1300110 MHz
WDW                    EM
SSB                     0
LB                0.30 Hz
GB                      0
PC                   1.00
```

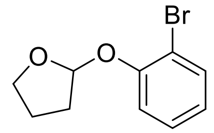

5m

125 MHz ^{13}C NMR

$CDCl_3$

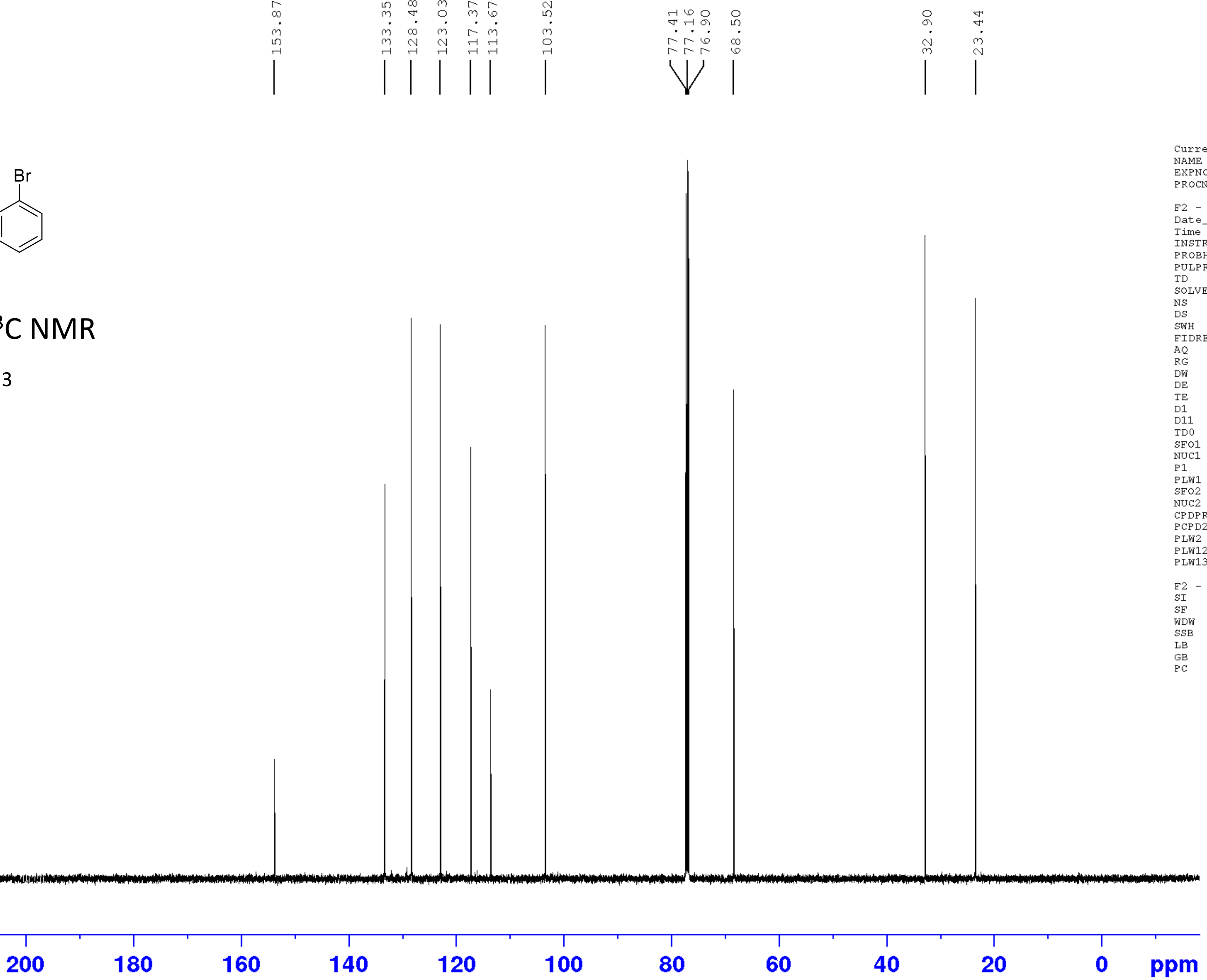


```
Current Data Parameters
NAME      vinn-4-147-1-islt-20200121
EXPNO                    2
PROCNO                   1

F2 - Acquisition Parameters
Date_             20210121
Time                 19.21 h
INSTRUM              spect
PROBHD     Z119470_0283 (
PULPROG             zgpg30
TD                   65536
SOLVENT              CDCl3
NS                     256
DS                       4
SWH              29761.904 Hz
FIDRES            0.908261 Hz
AQ               1.1010048 sec
RG                  206.72
DW                  16.800 usec
DE                    6.50 usec
TE                   295.1 K
D1              2.00000000 sec
D11             0.03000000 sec
TD0                      1
SFO1           125.7703643 MHz
NUC1                   13C
P1                    9.75 usec
PLW1           94.00000000 W
SFO2           500.1320005 MHz
NUC2                    1H
CPDPRG[2           waltz16
PCPD2                80.00 usec
PLW2           25.00000000 W
PLW12           0.46495000 W
PLW13           0.23387000 W

F2 - Processing parameters
SI                   32768
SF             125.7577762 MHz
WDW                     EM
SSB                      0
LB                    1.00 Hz
GB                       0
PC                    1.40
```

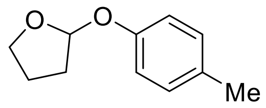

5n

500 MHz ^{1}H NMR

$CDCl_3$

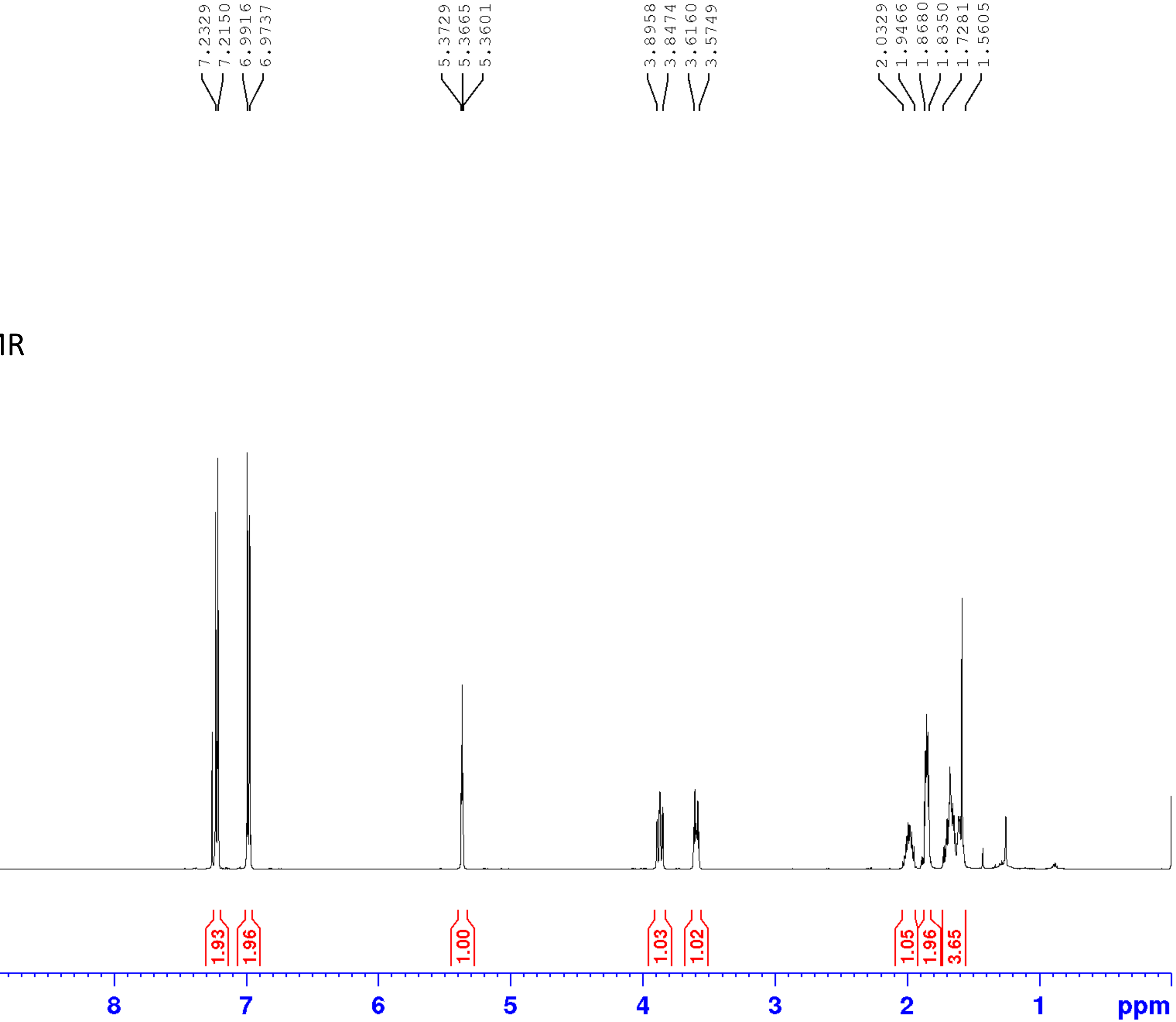


```
Current Data Parameters
NAME     vinn-4-190-2-clm8to9-20230103
EXPNO                    1
PROCNO                   1

F2 - Acquisition Parameters
Date_             20230103
Time                 16.58 h
INSTRUM              spect
PROBHD   Z119470_0283 (
PULPROG               zg30
TD                   65536
SOLVENT              CDCl3
NS                      16
DS                       2
SWH              10000.000 Hz
FIDRES            0.305176 Hz
AQ               3.2767999 sec
RG                   102.6
DW                  50.000 usec
DE                    6.50 usec
TE                   295.1 K
D1              1.00000000 sec
TD0                      1
SFO1           500.1330883 MHz
NUC1                    1H
P1                   10.91 usec
PLW1           25.00000000 W

F2 - Processing parameters
SI                   65536
SF             500.1300128 MHz
WDW                     EM
SSB                      0
LB                    0.30 Hz
GB                       0
PC                    1.00
```

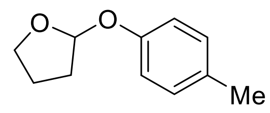

5n

125 MHz ^{13}C NMR

$CDCl_3$

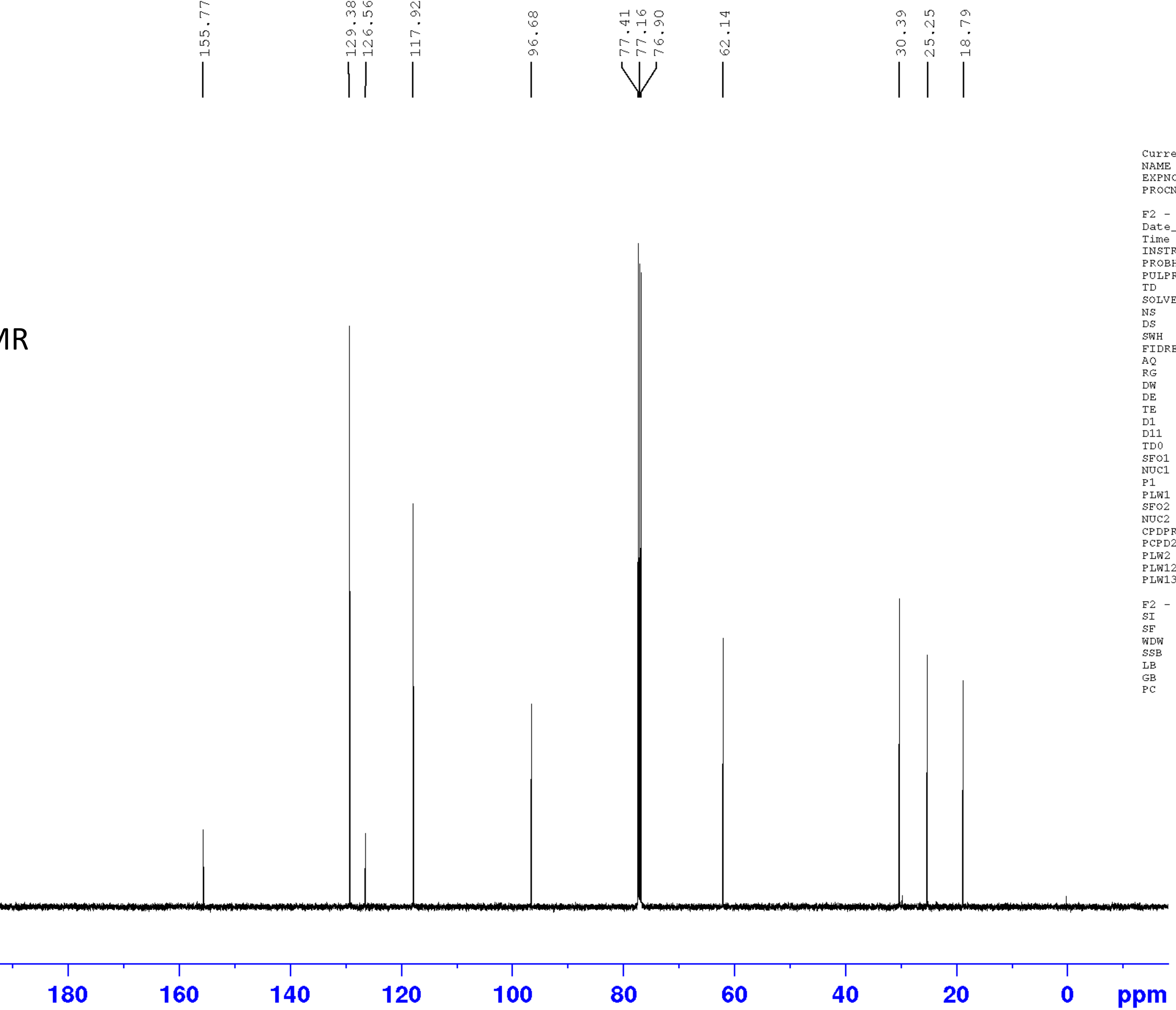


```
Current Data Parameters
NAME     vinn-4-190-2-clm8to9-2023010
EXPNO                  2
PROCNO                 1

F2 - Acquisition Parameters
Date_          20230103
Time              18.07 h
INSTRUM           spect
PROBHD   Z119470_0283 (
PULPROG          zgpg30
TD                65536
SOLVENT           CDCl3
NS                  300
DS                    4
SWH           29761.904 Hz
FIDRES         0.908261 Hz
AQ            1.1010048 sec
RG               206.72
DW               16.800 usec
DE                 6.50 usec
TE                295.2 K
D1           2.00000000 sec
D11          0.03000000 sec
TD0                   1
SFO1        125.7703643 MHz
NUC1                13C
P1                 9.75 usec
PLW1        94.00000000 W
SFO2        500.1320005 MHz
NUC2                 1H
CPDPRG[2        waltz16
PCPD2             80.00 usec
PLW2        25.00000000 W
PLW12        0.46495000 W
PLW13        0.23387000 W

F2 - Processing parameters
SI                32768
SF          125.7577740 MHz
WDW                  EM
SSB                   0
LB                 1.00 Hz
GB                    0
PC                 1.40
```

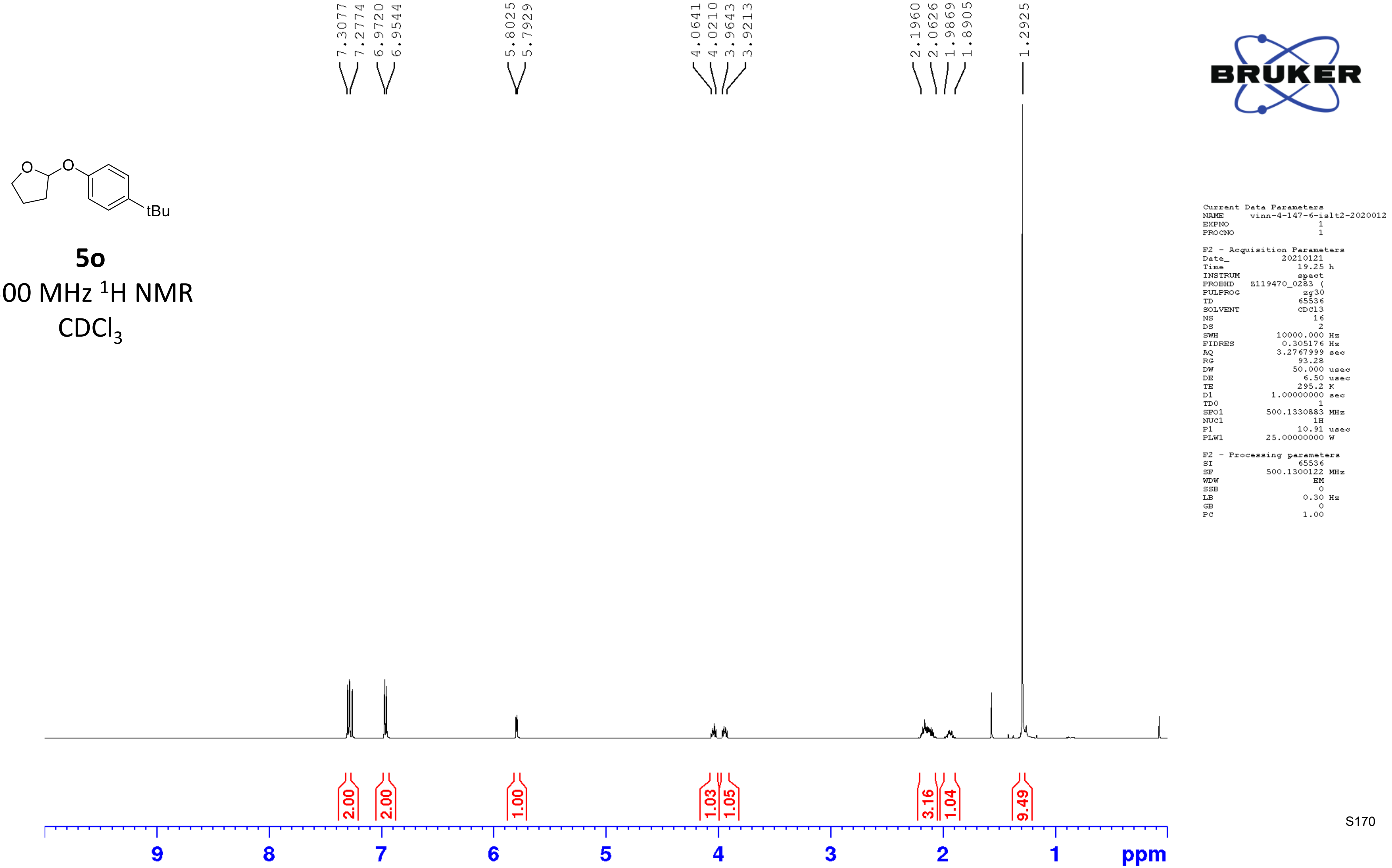

5o

500 MHz ^{1}H NMR

$CDCl_3$

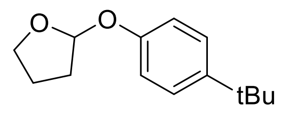

5o

125 MHz ^{13}C NMR

$CDCl_3$

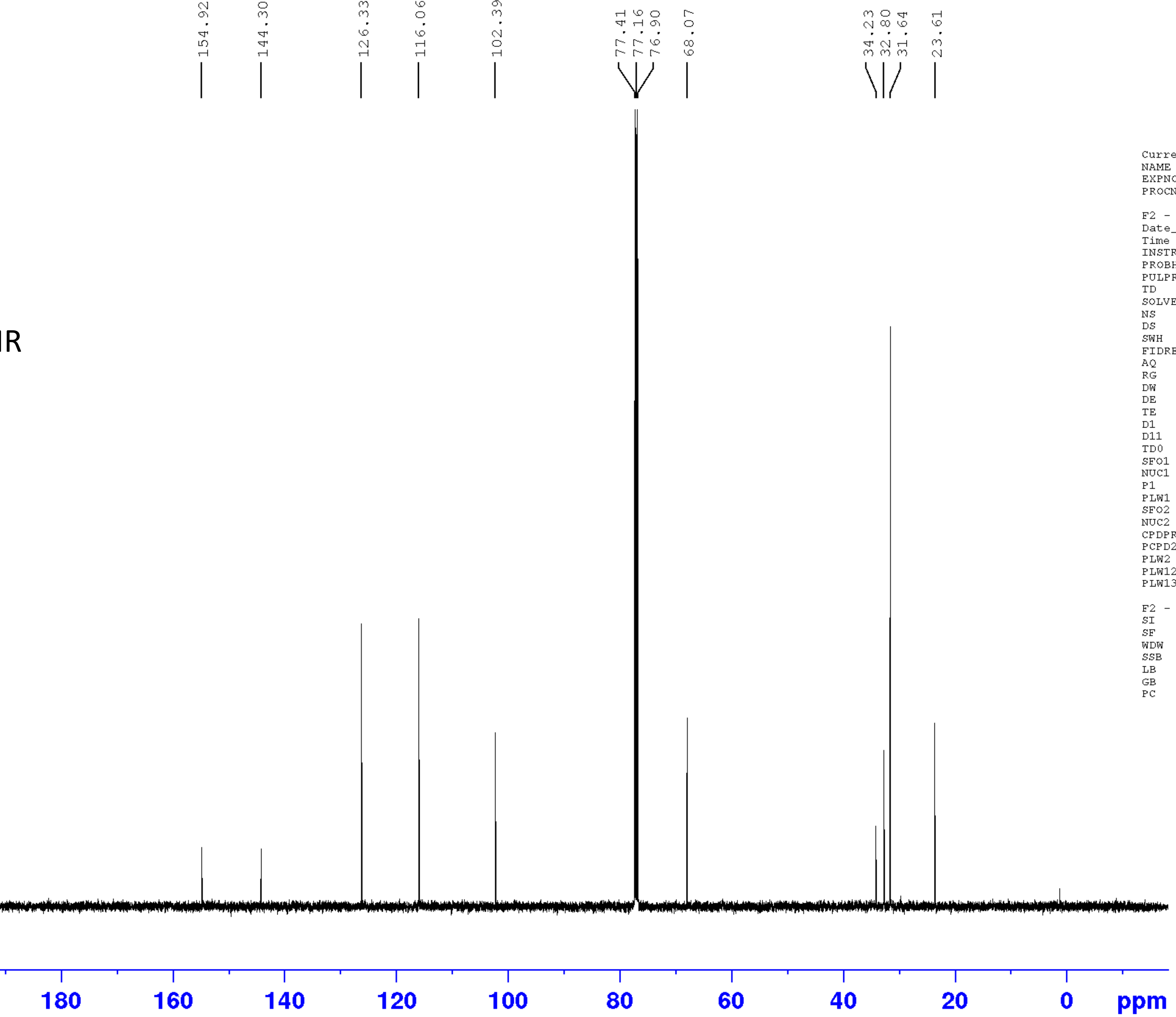


```
Current Data Parameters
NAME      vinn-4-147-6-islt2-20200121
EXPNO                    2
PROCNO                   1

F2 - Acquisition Parameters
Date_            20210121
Time                19.39 h
INSTRUM             spect
PROBHD   Z119470_0283 (
PULPROG            zgpg30
TD                  65536
SOLVENT             CDCl3
NS                    256
DS                      4
SWH            29761.904 Hz
FIDRES          0.908261 Hz
AQ             1.1010048 sec
RG                 206.72
DW                 16.800 usec
DE                   6.50 usec
TE                  295.2 K
D1            2.00000000 sec
D11           0.03000000 sec
TD0                     1
SFO1         125.7703643 MHz
NUC1                  13C
P1                   9.75 usec
PLW1         94.00000000 W
SFO2         500.1320005 MHz
NUC2                   1H
CPDPRG[2          waltz16
PCPD2               80.00 usec
PLW2         25.00000000 W
PLW12         0.46495000 W
PLW13         0.23387000 W

F2 - Processing parameters
SI                  32768
SF           125.7577731 MHz
WDW                    EM
SSB                     0
LB                   1.00 Hz
GB                      0
PC                   1.40
```

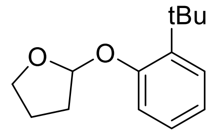

5p

400 MHz ^{1}H NMR

$CDCl_3$

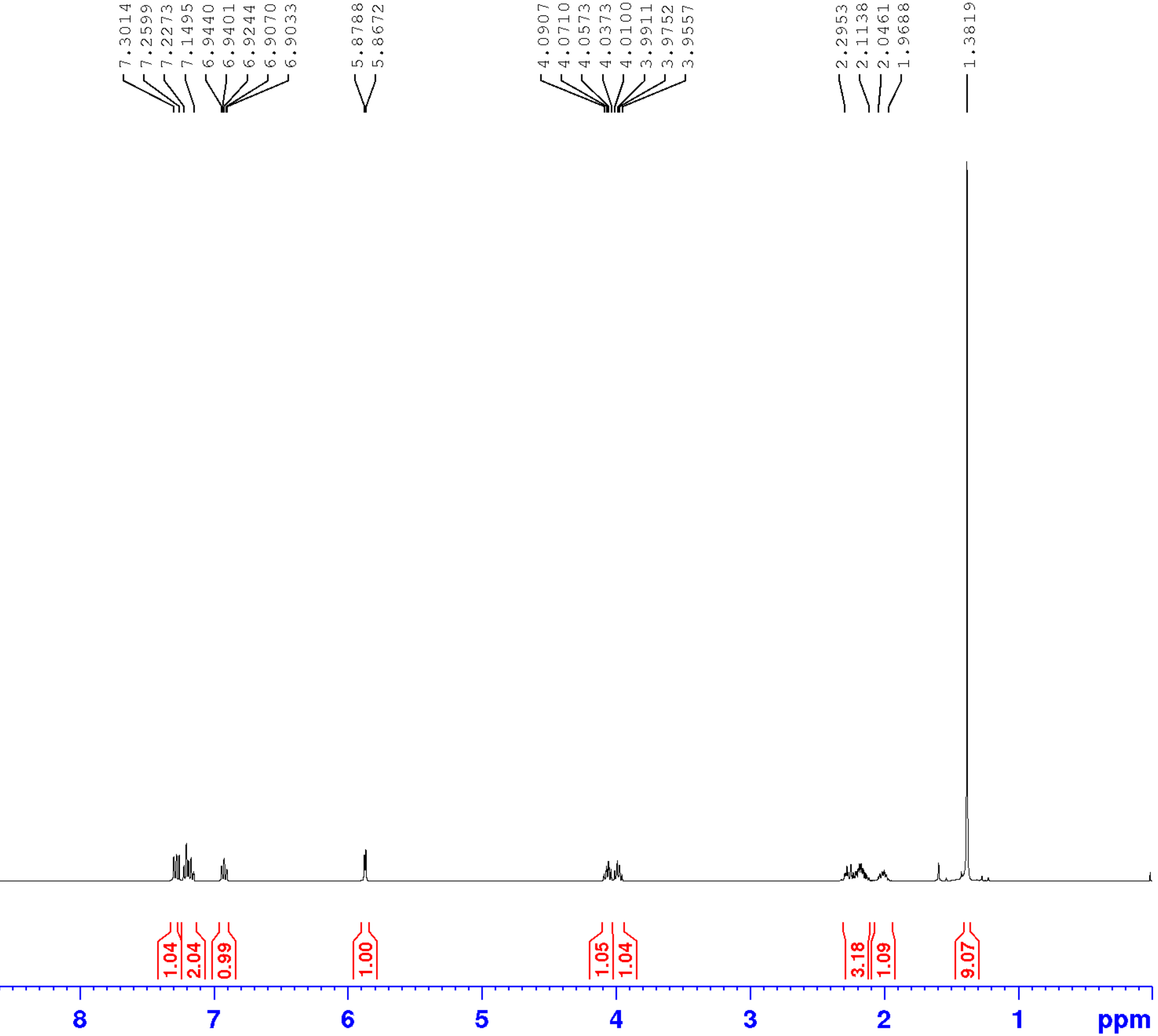


```
Current Data Parameters
NAME      vinn-4-183-4-islt-20230320
EXPNO                    1
PROCNO                   1

F2 - Acquisition Parameters
Date_             20230320
Time                 18.18 h
INSTRUM              spect
PROBHD     Z108618_0257 (
PULPROG               zg30
TD                   65536
SOLVENT              CDCl3
NS                      16
DS                       2
SWH              8012.820 Hz
FIDRES           0.244532 Hz
AQ              4.0894465 sec
RG                    80.6
DW                  62.400 usec
DE                    6.50 usec
TE                   295.4 K
D1             1.00000000 sec
TD0                      1
SFO1          400.1324708 MHz
NUC1                    1H
P1                   15.00 usec
PLW1           12.50000000 W

F2 - Processing parameters
SI                   65536
SF            400.1300101 MHz
WDW                     EM
SSB                      0
LB                    0.30 Hz
GB                       0
PC                    1.00
```

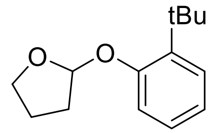

5p

100 MHz ^{13}C NMR

$CDCl_3$

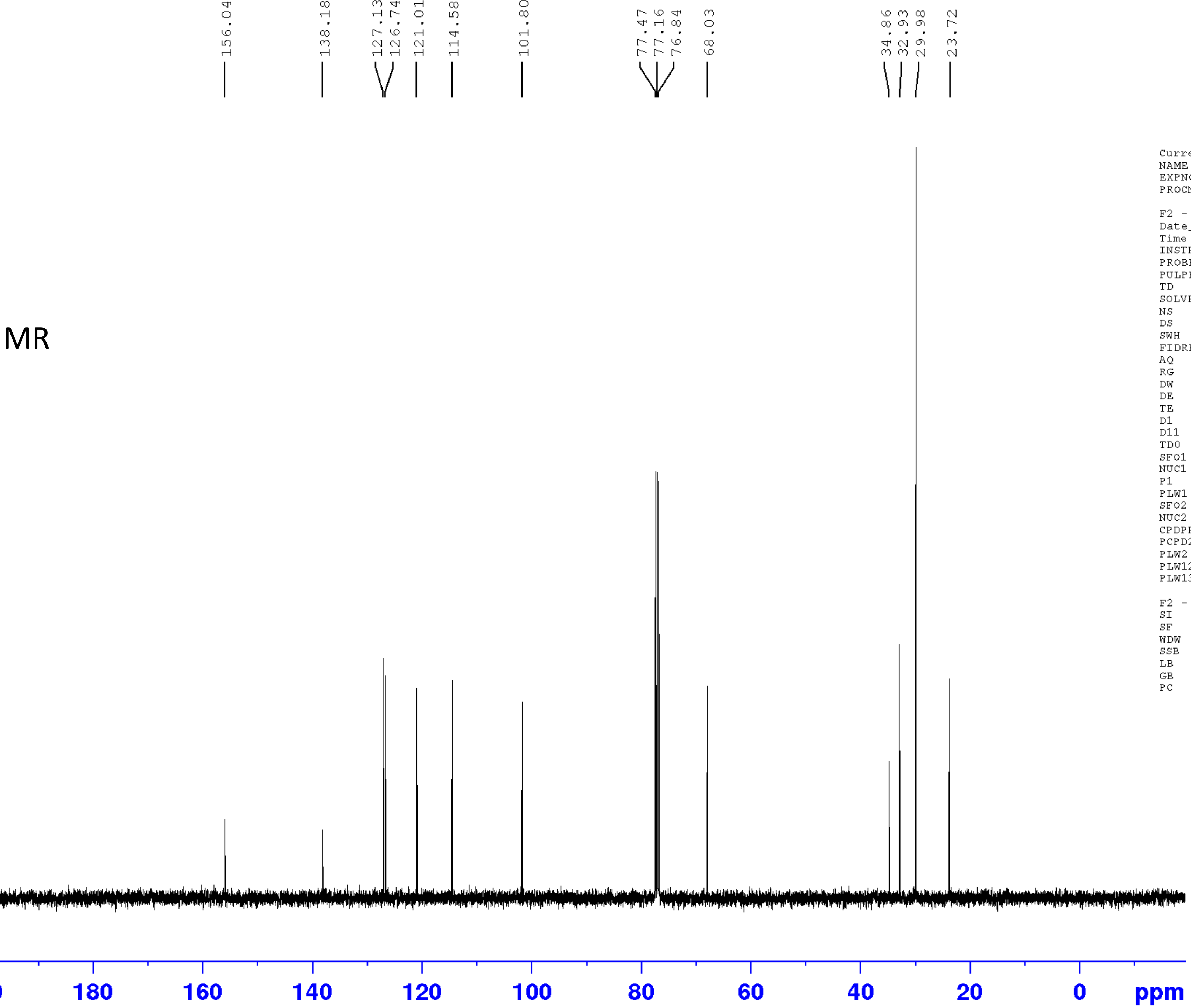

Current Data Parameters
NAME vinn-4-183-4-islt-20230320
EXPNO 2
PROCNO 1

F2 - Acquisition Parameters
Date_ 20230320
Time 18.22 h
INSTRUM spect
PROBHD Z108618_0257 (
PULPROG zgpg30
TD 65536
SOLVENT CDCl3
NS 40
DS 4
SWH 24038.461 Hz
FIDRES 0.733596 Hz
AQ 1.3631488 sec
RG 203
DW 20.800 usec
DE 6.50 usec
TE 295.8 K
D1 2.00000000 sec
D11 0.03000000 sec
TD0 1
SFO1 100.6228298 MHz
NUC1 13C
P1 10.00 usec
PLW1 51.00000000 W
SFO2 400.1316005 MHz
NUC2 1H
CPDPRG[2 waltz16
PCPD2 90.00 usec
PLW2 12.50000000 W
PLW12 0.34722000 W
PLW13 0.17465000 W

F2 - Processing parameters
SI 32768
SF 100.6127584 MHz
WDW EM
SSB 0
LB 1.00 Hz
GB 0
PC 1.40

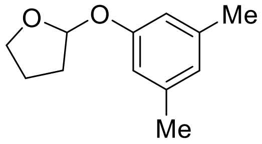

5q

500 MHz ^{1}H NMR

$CDCl_3$

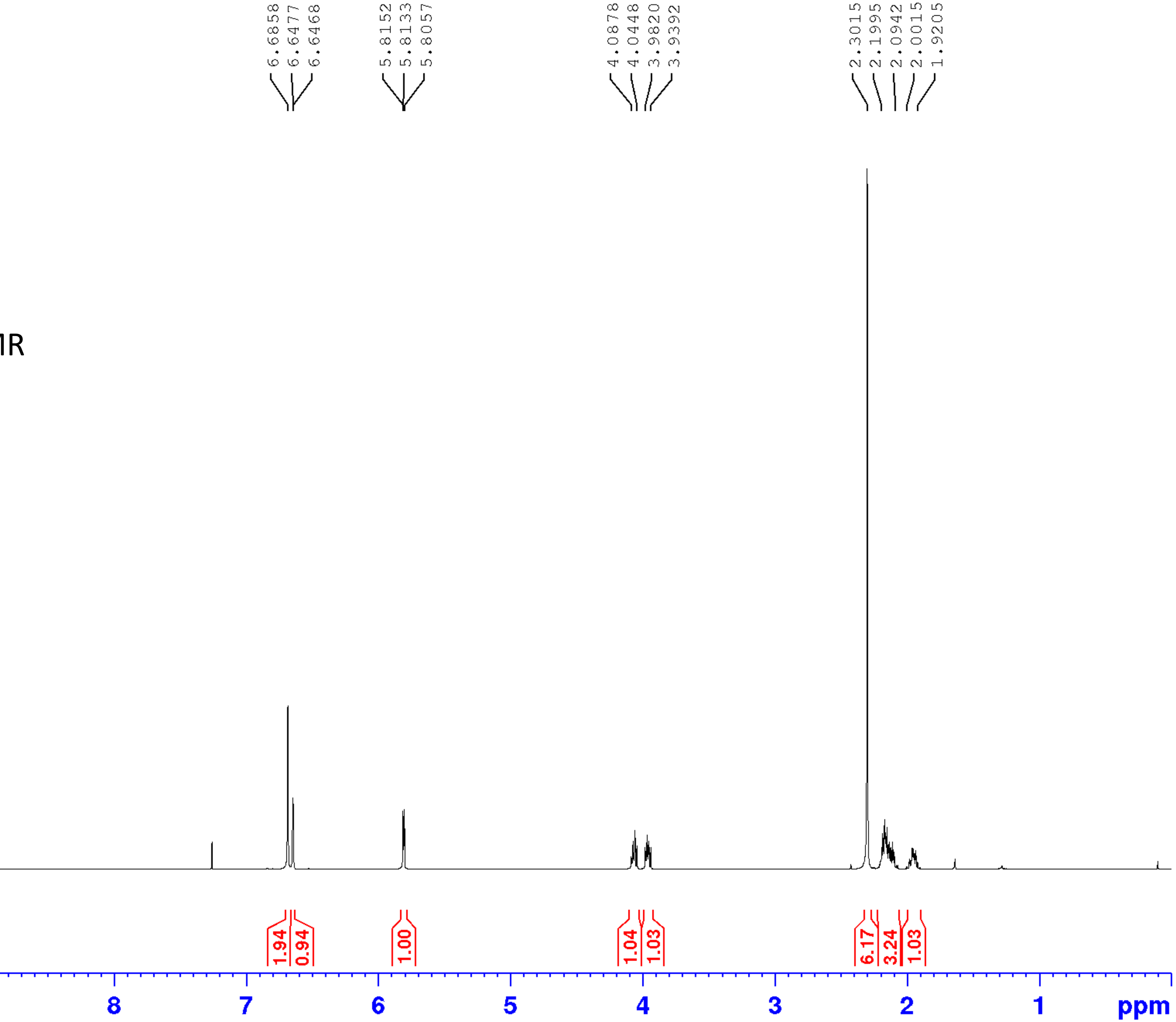

Current Data Parameters
NAME vinn-4-147-7-islt-20200120
EXPNO 3
PROCNO 1

F2 - Acquisition Parameters
Date_ 20210121
Time 18.38 h
INSTRUM spect
PROBHD Z119470_0283 (
PULPROG zg30
TD 65536
SOLVENT CDCl3
NS 16
DS 2
SWH 10000.000 Hz
FIDRES 0.305176 Hz
AQ 3.2767999 sec
RG 30.85
DW 50.000 usec
DE 6.50 usec
TE 295.1 K
D1 1.00000000 sec
TD0 1
SFO1 500.1330883 MHz
NUC1 1H
P1 10.91 usec
PLW1 25.00000000 W

F2 - Processing parameters
SI 65536
SF 500.1300122 MHz
WDW EM
SSB 0
LB 0.30 Hz
GB 0
PC 1.00

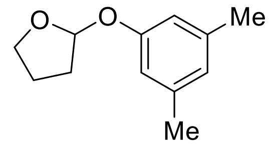

5q

125 MHz ^{13}C NMR

$CDCl_3$

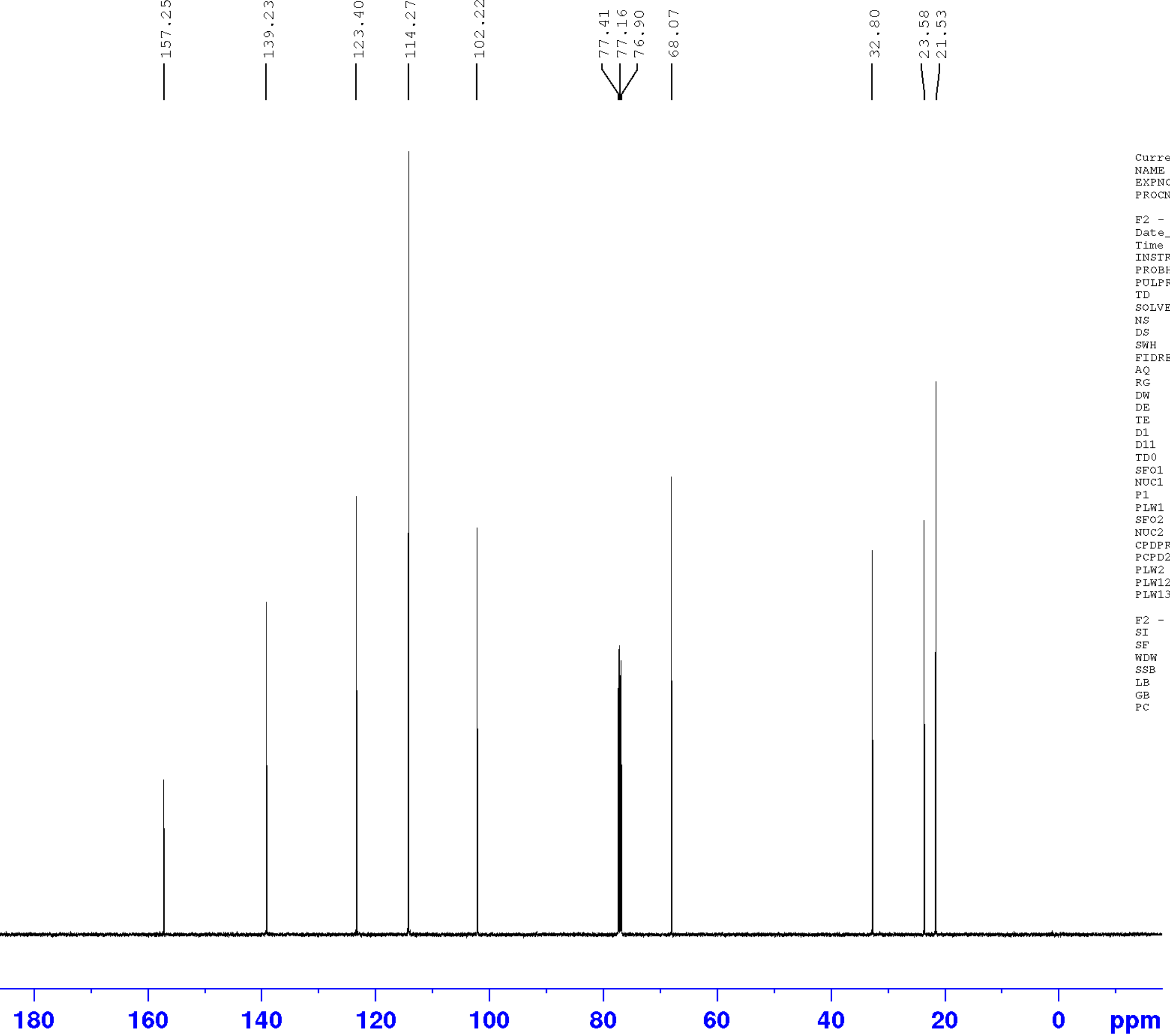


```
Current Data Parameters
NAME     vinn-4-147-7-islt-20200120
EXPNO                 4
PROCNO                1

F2 - Acquisition Parameters
Date_          20210121
Time              18.52 h
INSTRUM           spect
PROBHD   Z119470_0283 (
PULPROG          zgpg30
TD                65536
SOLVENT           CDCl3
NS                  256
DS                    4
SWH           29761.904 Hz
FIDRES         0.908261 Hz
AQ            1.1010048 sec
RG               206.72
DW               16.800 usec
DE                 6.50 usec
TE                295.2 K
D1           2.00000000 sec
D11          0.03000000 sec
TD0                   1
SFO1        125.7703643 MHz
NUC1                13C
P1                 9.75 usec
PLW1        94.00000000 W
SFO2        500.1320005 MHz
NUC2                 1H
CPDPRG[2        waltz16
PCPD2             80.00 usec
PLW2        25.00000000 W
PLW12        0.46495000 W
PLW13        0.23387000 W

F2 - Processing parameters
SI                32768
SF          125.7577797 MHz
WDW                  EM
SSB                   0
LB                 1.00 Hz
GB                    0
PC                 1.40
```

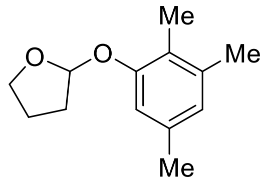

5r

500 MHz ^{1}H NMR

$CDCl_3$

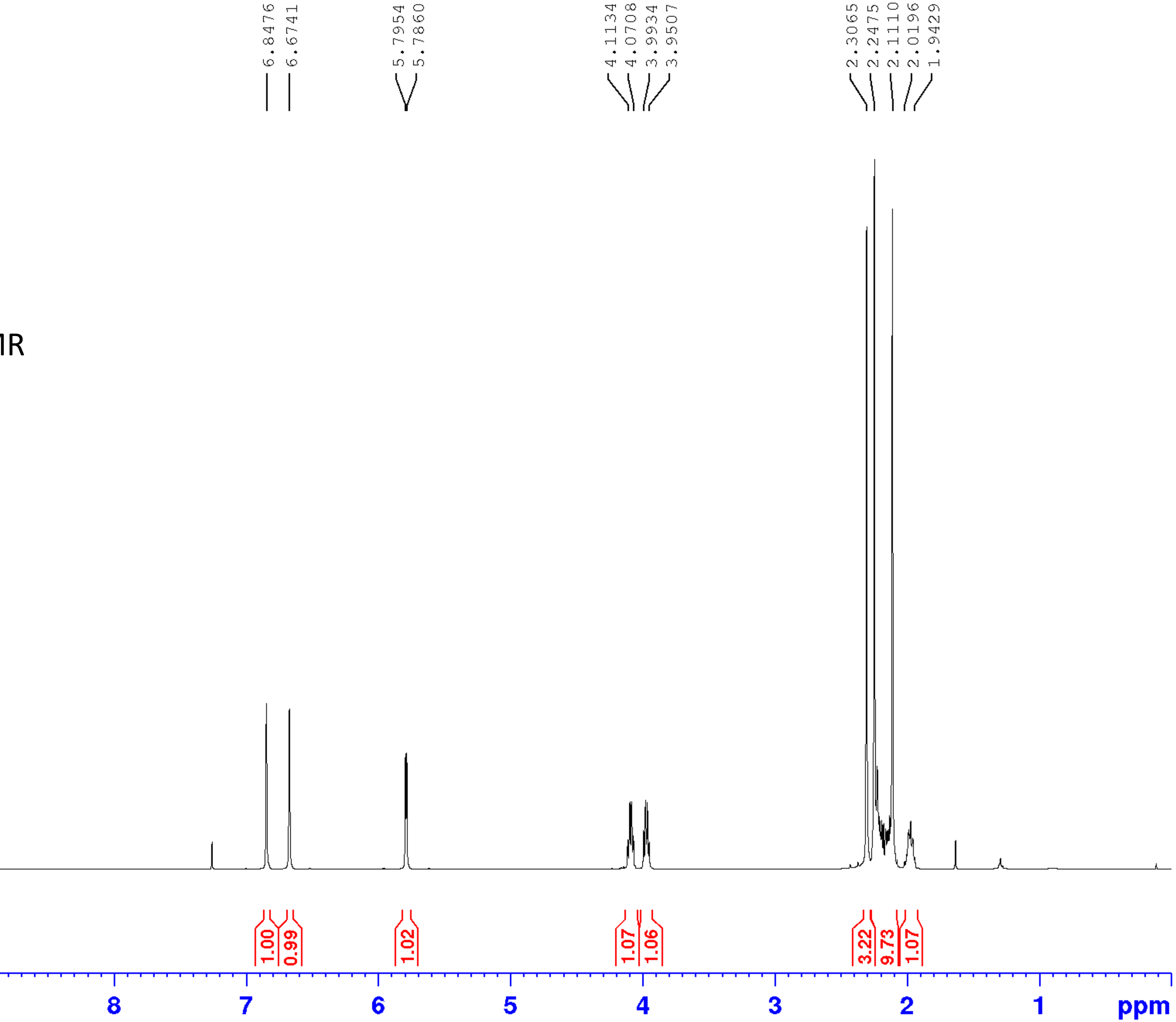


```
Current Data Parameters
NAME      vinn-4-147-8-islt-20200120
EXPNO                  1
PROCNO                 1

F2 - Acquisition Parameters
Date_           20210121
Time                7.22 h
INSTRUM            spect
PROBHD   Z119470_0283 (
PULPROG             zg30
TD                 65536
SOLVENT            CDCl3
NS                    16
DS                     2
SWH            10000.000 Hz
FIDRES          0.305176 Hz
AQ             3.2767999 sec
RG                 30.85
DW                50.000 usec
DE                  6.50 usec
TE                 295.1 K
D1            1.00000000 sec
TD0                    1
SFO1         500.1330883 MHz
NUC1                  1H
P1                 10.91 usec
PLW1         25.00000000 W

F2 - Processing parameters
SI                 65536
SF           500.1300125 MHz
WDW                   EM
SSB                    0
LB                  0.30 Hz
GB                     0
PC                  1.00
```

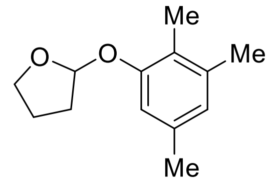

5r

125 MHz ^{13}C NMR

$CDCl_3$

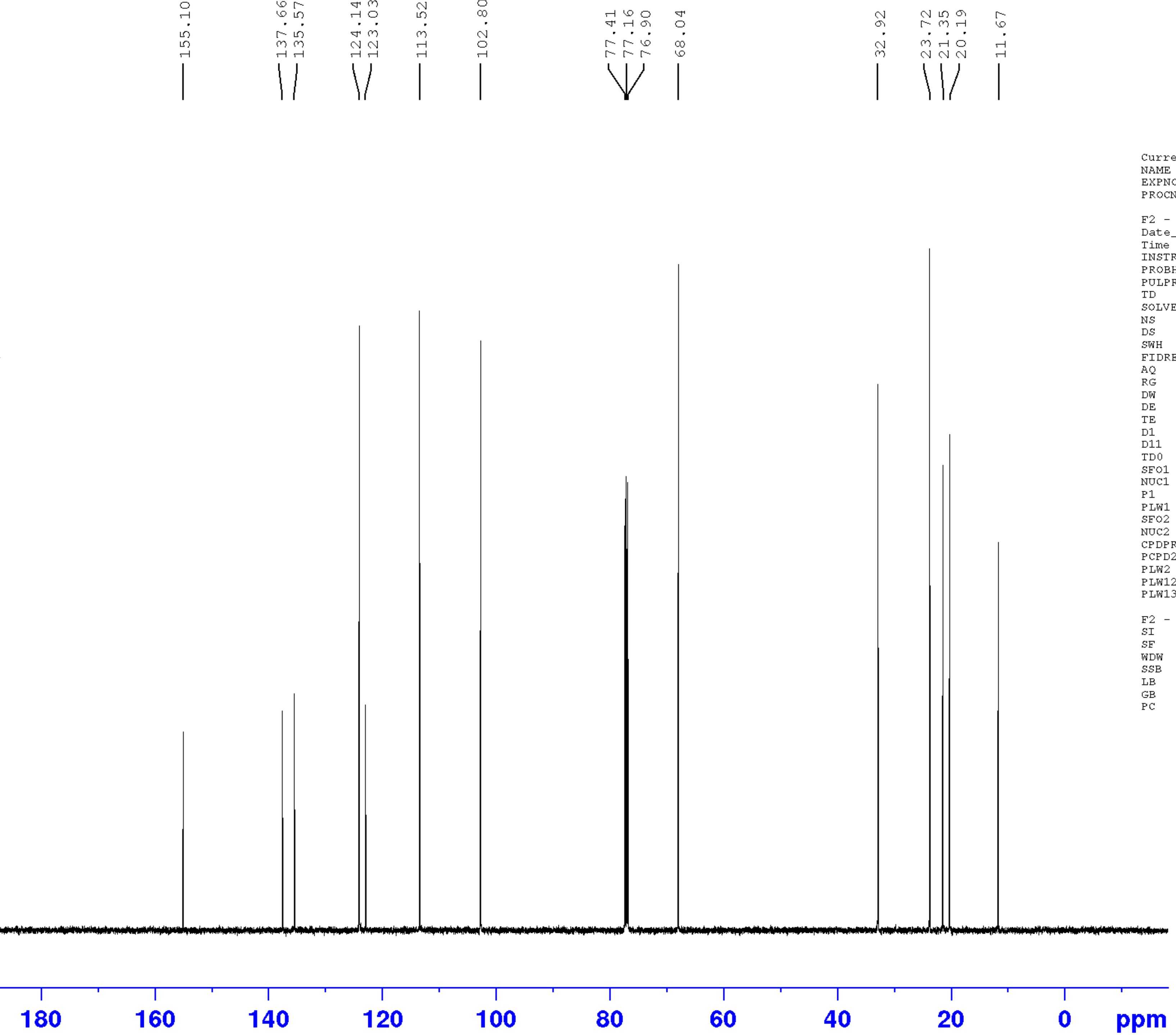


```
Current Data Parameters
NAME      vinn-4-147-8-islt-20200120
EXPNO                2
PROCNO               1

F2 - Acquisition Parameters
Date_           20210121
Time                7.36 h
INSTRUM            spect
PROBHD   Z119470_0283 (
PULPROG          zgpg30
TD                65536
SOLVENT           CDCl3
NS                  256
DS                    4
SWH           29761.904 Hz
FIDRES         0.908261 Hz
AQ            1.1010048 sec
RG               206.72
DW               16.800 usec
DE                 6.50 usec
TE                295.2 K
D1           2.00000000 sec
D11          0.03000000 sec
TD0                   1
SFO1        125.7703643 MHz
NUC1                13C
P1                 9.75 usec
PLW1        94.00000000 W
SFO2        500.1320005 MHz
NUC2                 1H
CPDPRG[2        waltz16
PCPD2             80.00 usec
PLW2        25.00000000 W
PLW12        0.46495000 W
PLW13        0.23387000 W

F2 - Processing parameters
SI                32768
SF          125.7577807 MHz
WDW                  EM
SSB                   0
LB                 1.00 Hz
GB                    0
PC                 1.40
```

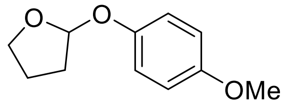

5s

500 MHz ^{1}H NMR

$CDCl_3$

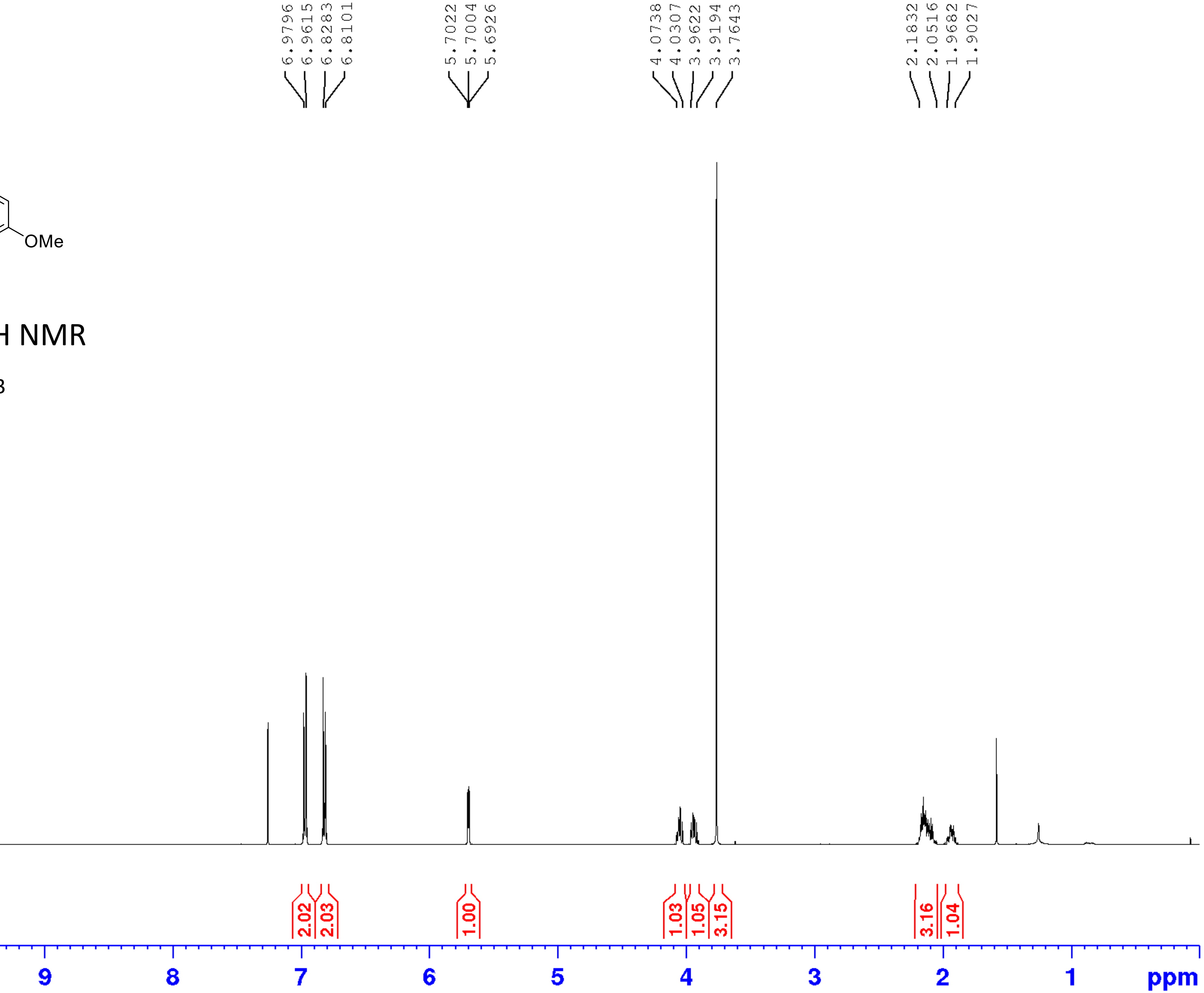


```
Current Data Parameters
NAME      vinn-4-147-4-islt-20200120
EXPNO                      1
PROCNO                     1

F2 - Acquisition Parameters
Date_               20210121
Time                    5.27 h
INSTRUM                spect
PROBHD    Z119470_0283 (
PULPROG                 zg30
TD                     65536
SOLVENT                CDCl3
NS                        16
DS                         2
SWH                10000.000 Hz
FIDRES              0.305176 Hz
AQ                 3.2767999 sec
RG                     102.6
DW                    50.000 usec
DE                      6.50 usec
TE                     295.1 K
D1                1.00000000 sec
TD0                        1
SFO1             500.1330883 MHz
NUC1                      1H
P1                     10.91 usec
PLW1             25.00000000 W

F2 - Processing parameters
SI                     65536
SF               500.1300123 MHz
WDW                       EM
SSB                        0
LB                      0.30 Hz
GB                         0
PC                      1.00
```

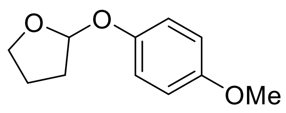

5s

125 MHz ^{13}C NMR

$CDCl_3$

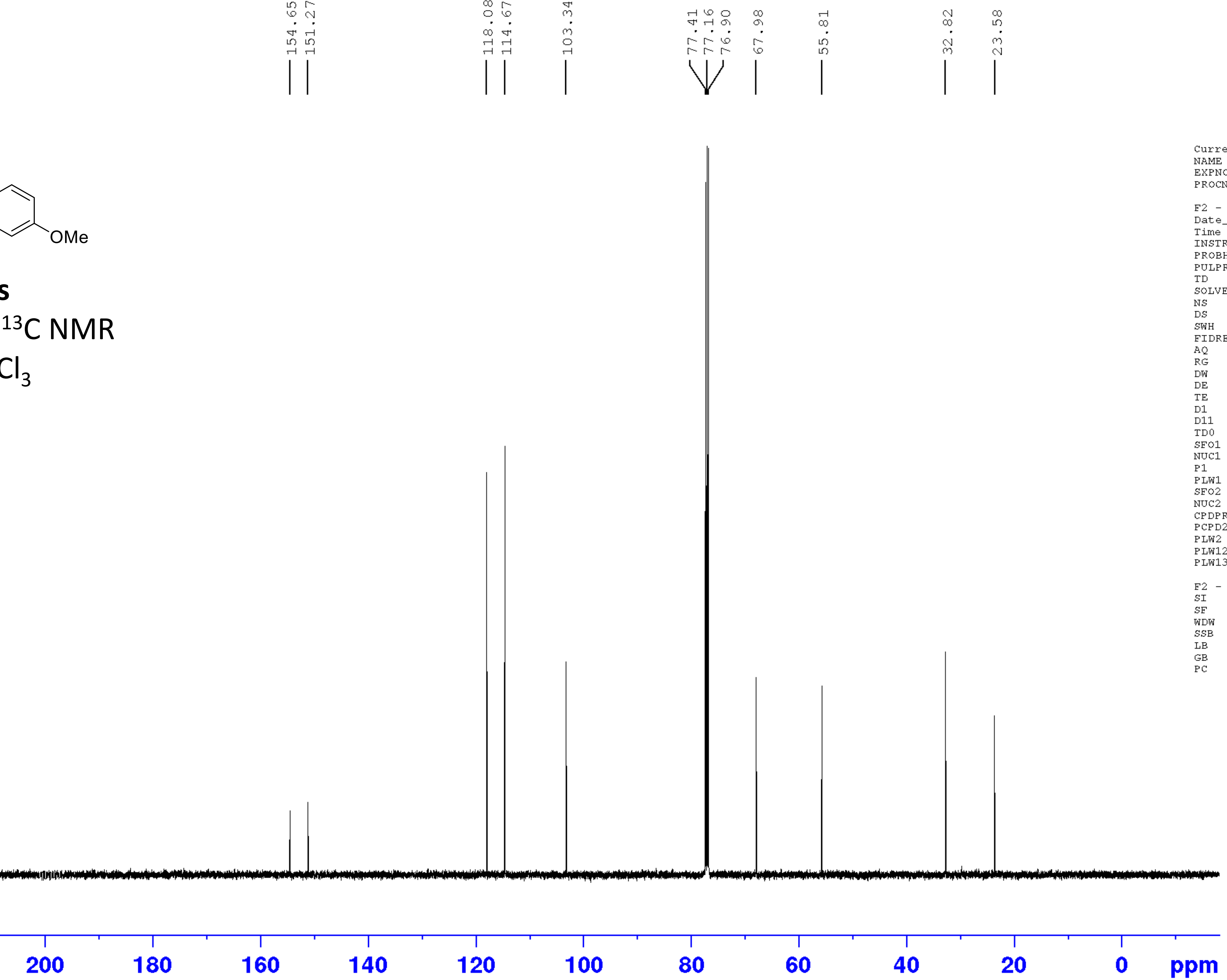


```
Current Data Parameters
NAME      vinn-4-147-4-islt-20200120
EXPNO                    2
PROCNO                   1

F2 - Acquisition Parameters
Date_            20210121
Time                 5.41 h
INSTRUM             spect
PROBHD   Z119470_0283 (
PULPROG            zgpg30
TD                  65536
SOLVENT             CDCl3
NS                    256
DS                      4
SWH             29761.904 Hz
FIDRES           0.908261 Hz
AQ              1.1010048 sec
RG                 206.72
DW                 16.800 usec
DE                   6.50 usec
TE                  295.1 K
D1             2.00000000 sec
D11            0.03000000 sec
TD0                     1
SFO1          125.7703643 MHz
NUC1                  13C
P1                   9.75 usec
PLW1          94.00000000 W
SFO2          500.1320005 MHz
NUC2                   1H
CPDPRG[2          waltz16
PCPD2               80.00 usec
PLW2          25.00000000 W
PLW12          0.46495000 W
PLW13          0.23387000 W

F2 - Processing parameters
SI                  32768
SF            125.7577736 MHz
WDW                    EM
SSB                     0
LB                   1.00 Hz
GB                      0
PC                   1.40
```

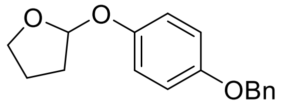

5t

500 MHz ^{1}H NMR

$CDCl_3$

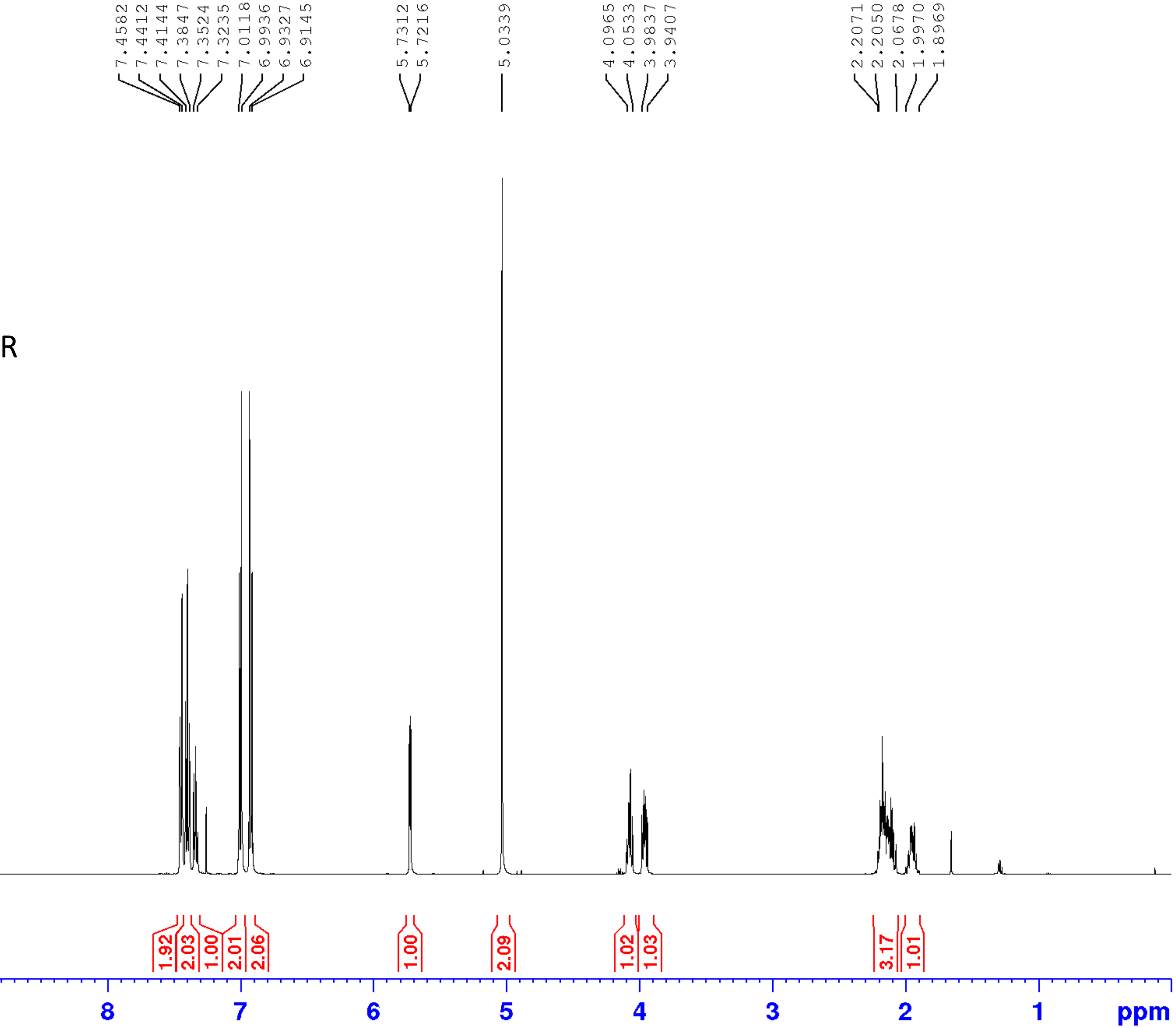

BRUKER

```
Current Data Parameters
NAME     vinn-4-147-5-islt-20200120
EXPNO                        1
PROCNO                       1

F2 - Acquisition Parameters
Date_                 20210121
Time                    5.45 h
INSTRUM                  spect
PROBHD       Z119470_0283 (
PULPROG                   zg30
TD                       65536
SOLVENT                  CDCl3
NS                          16
DS                           2
SWH              10000.000 Hz
FIDRES            0.305176 Hz
AQ               3.2767999 sec
RG                       30.85
DW                  50.000 usec
DE                    6.50 usec
TE                     295.2 K
D1              1.00000000 sec
TD0                          1
SFO1           500.1330883 MHz
NUC1                        1H
P1                   10.91 usec
PLW1           25.00000000 W

F2 - Processing parameters
SI                       65536
SF             500.1300121 MHz
WDW                         EM
SSB                          0
LB                     0.30 Hz
GB                           0
PC                        1.00
```

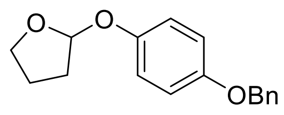

5t

125 MHz ^{13}C NMR

$CDCl_3$

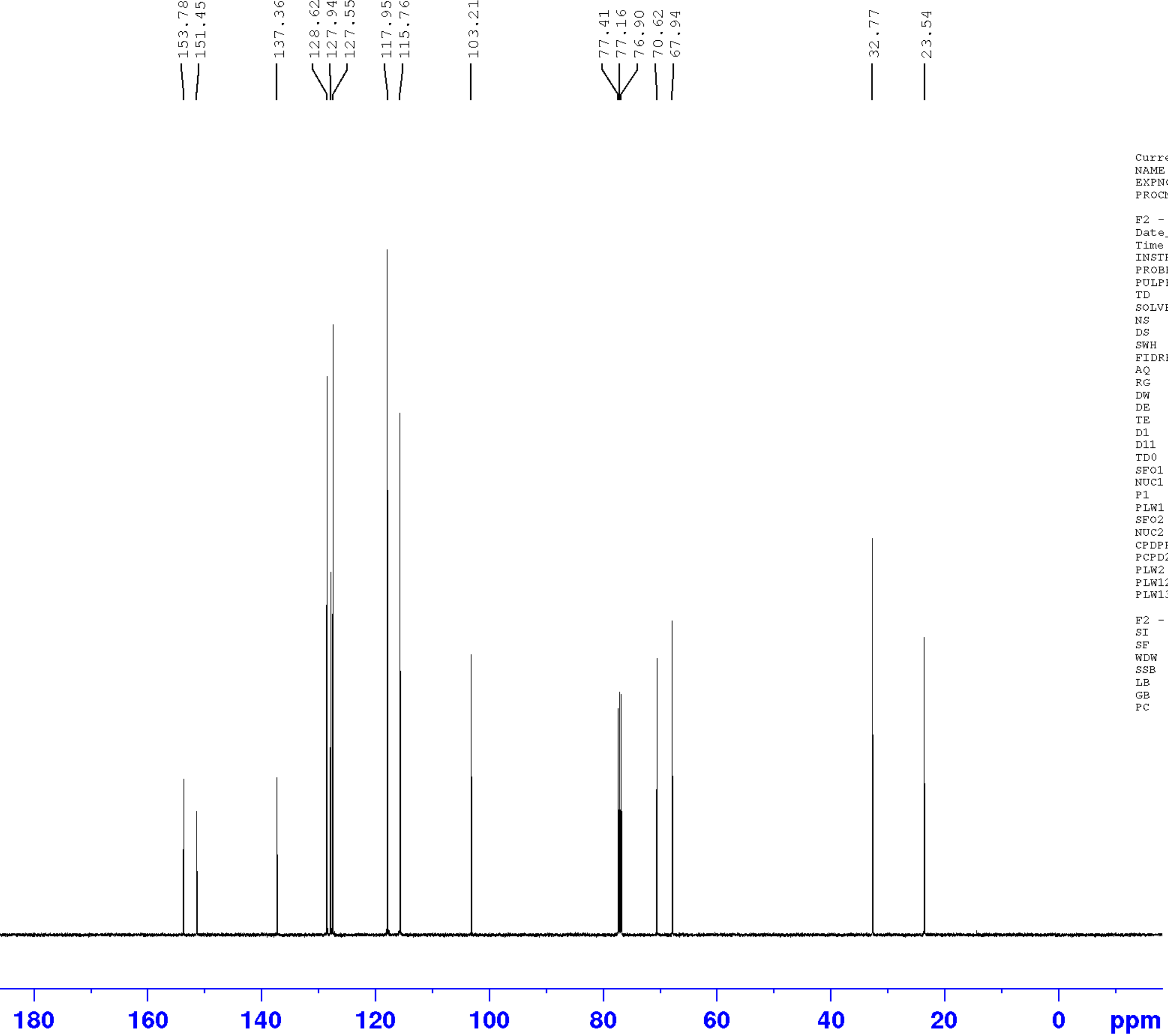

Current Data Parameters
NAME vinn-4-147-5-islt-20200120
EXPNO 2
PROCNO 1

F2 - Acquisition Parameters
Date_ 20210121
Time 5.59 h
INSTRUM spect
PROBHD Z119470_0283 (
PULPROG zgpg30
TD 65536
SOLVENT CDCl3
NS 256
DS 4
SWH 29761.904 Hz
FIDRES 0.908261 Hz
AQ 1.1010048 sec
RG 206.72
DW 16.800 usec
DE 6.50 usec
TE 295.2 K
D1 2.00000000 sec
D11 0.03000000 sec
TD0 1
SFO1 125.7703643 MHz
NUC1 13C
P1 9.75 usec
PLW1 94.00000000 W
SFO2 500.1320005 MHz
NUC2 1H
CPDPRG[2 waltz16
PCPD2 80.00 usec
PLW2 25.00000000 W
PLW12 0.46495000 W
PLW13 0.23387000 W

F2 - Processing parameters
SI 32768
SF 125.7577846 MHz
WDW EM
SSB 0
LB 1.00 Hz
GB 0
PC 1.40

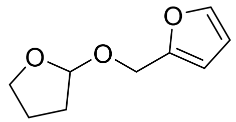

5u

500 MHz ^{1}H NMR

$CDCl_3$

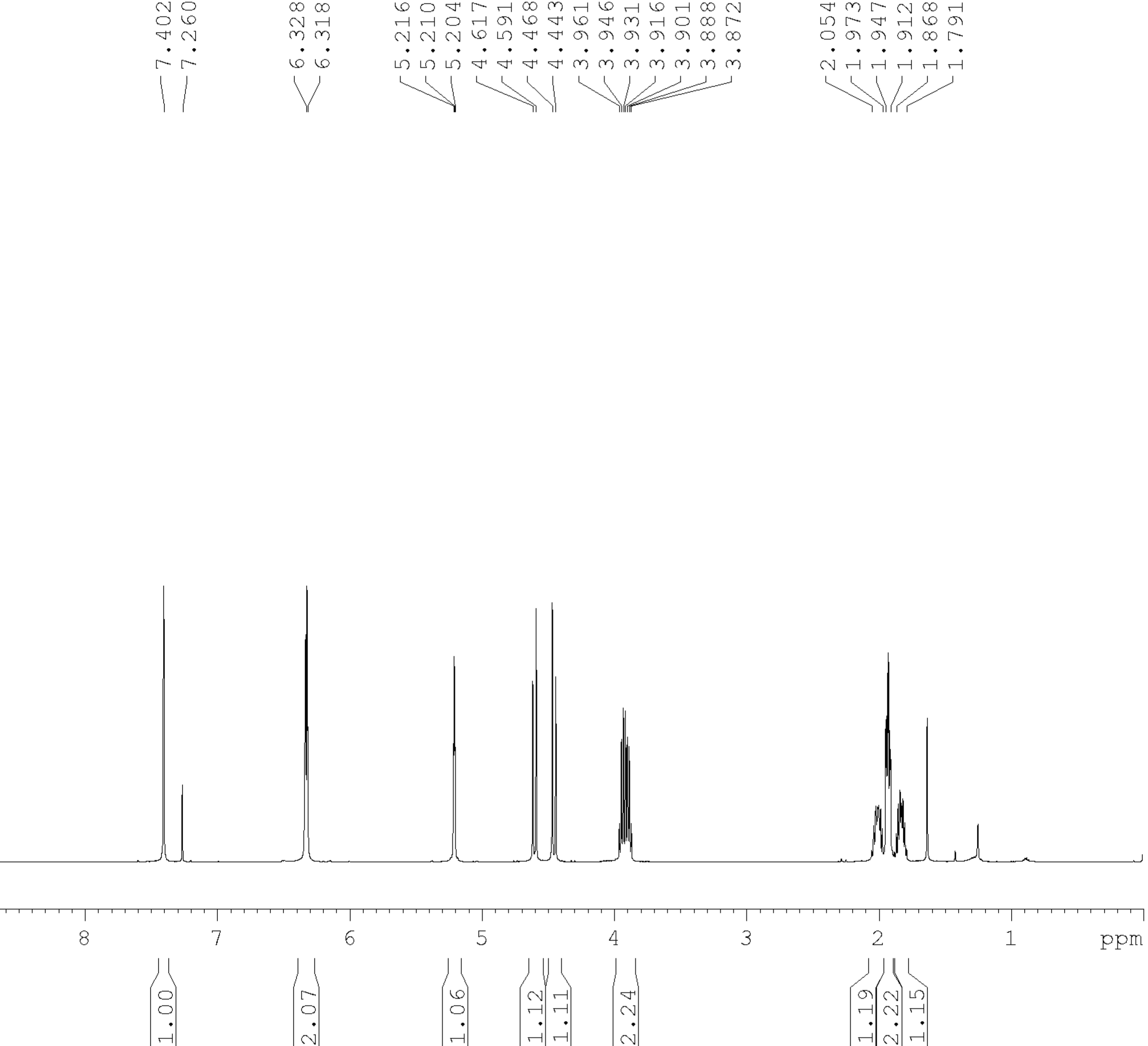

Current Data Parameters
NAME vinn-7-105-2-islt-20230807
EXPNO 1
PROCNO 1

F2 - Acquisition Parameters
Date_ 20230807
Time 17.51 h
INSTRUM spect
PROBHD Z149001_0010 (
PULPROG zg30
TD 65536
SOLVENT CDCl3
NS 16
DS 2
SWH 10000.000 Hz
FIDRES 0.305176 Hz
AQ 3.2767999 sec
RG 30.85
DW 50.000 usec
DE 10.00 usec
TE 296.1 K
D1 1.00000000 sec
TD0 1
SFO1 500.1330883 MHz
NUC1 1H
P1 11.25 usec
PLW1 17.35199928 W

F2 - Processing parameters
SI 65536
SF 500.1300134 MHz
WDW EM
SSB 0
LB 0.30 Hz
GB 0
PC 1.00

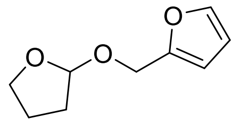

5u
125 MHz ^{13}C NMR
$CDCl_3$

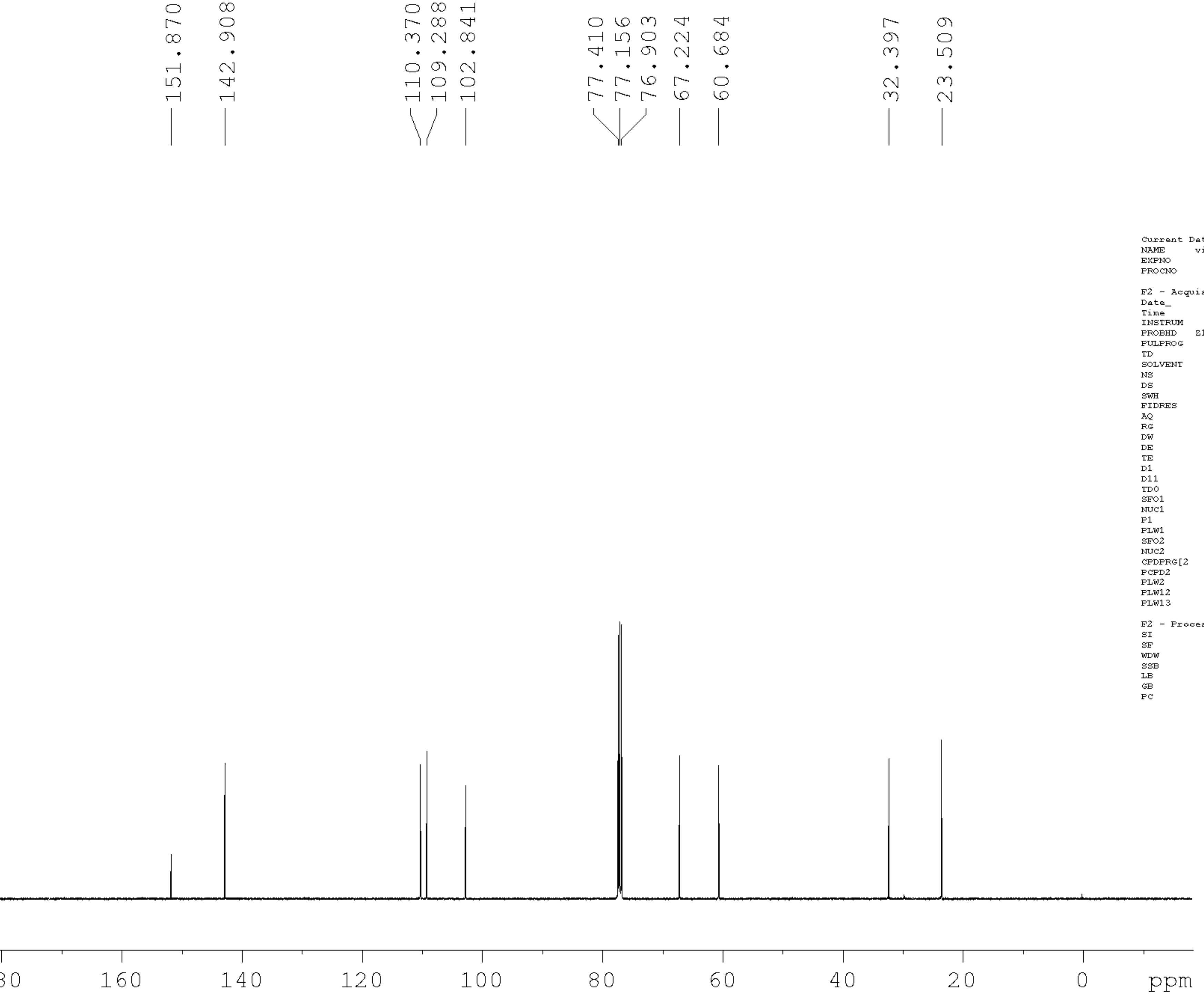


```
Current Data Parameters
NAME        vinn-7-105-2-islt-20230807
EXPNO                    2
PROCNO                   1

F2 - Acquisition Parameters
Date_             20230807
Time                 18.02 h
INSTRUM              spect
PROBHD   Z149001_0010 (
PULPROG            zgpg30
TD                   65536
SOLVENT              CDCl3
NS                     200
DS                       4
SWH              29761.904 Hz
FIDRES            0.908261 Hz
AQ               1.1010048 sec
RG                  206.72
DW                  16.800 usec
DE                   18.00 usec
TE                   296.2 K
D1              2.00000000 sec
D11             0.03000000 sec
TD0                      1
SFO1           125.7703643 MHz
NUC1                   13C
P1                   10.00 usec
PLW1           61.00000000 W
SFO2           500.1320005 MHz
NUC2                    1H
CPDPRG[2           waltz16
PCPD2                80.00 usec
PLW2           17.35199928 W
PLW12           0.34314999 W
PLW13           0.17260000 W

F2 - Processing parameters
SI                   32768
SF             125.7577745 MHz
WDW                     EM
SSB                      0
LB                    1.00 Hz
GB                       0
PC                    1.40
```

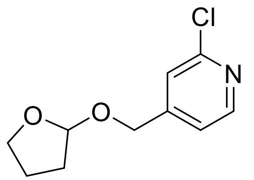

5v

400 MHz ^{1}H NMR

$CDCl_3$

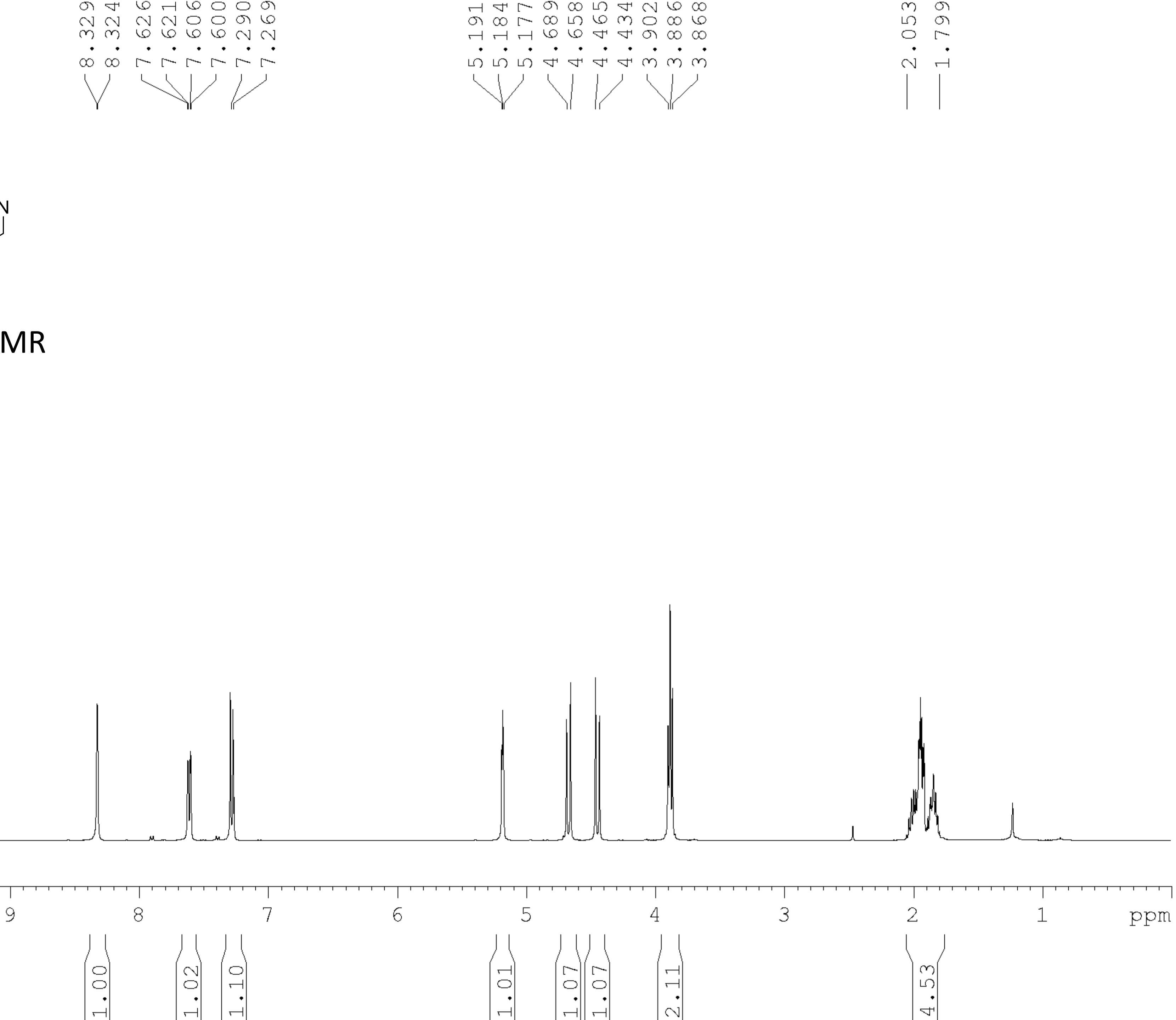


```
Current Data Parameters
NAME      vinn-7-110-2-islt-20230809
EXPNO                1
PROCNO               1

F2 - Acquisition Parameters
Date_          20230809
Time              18.00 h
INSTRUM           spect
PROBHD   Z108618_0257 (
PULPROG            zg30
TD                65536
SOLVENT           CDCl3
NS                   16
DS                    2
SWH            8012.820 Hz
FIDRES         0.244532 Hz
AQ            4.0894465 sec
RG                 90.5
DW               62.400 usec
DE                 6.50 usec
TE                295.9 K
D1           1.00000000 sec
TD0                   1
SFO1        400.1324708 MHz
NUC1                 1H
P1                15.00 usec
PLW1        12.50000000 W

F2 - Processing parameters
SI                65536
SF          400.1300096 MHz
WDW                  EM
SSB                   0
LB                 0.30 Hz
GB                    0
PC                 1.00
```

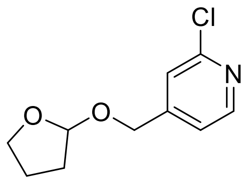

5v

100 MHz ^{13}C NMR

$CDCl_3$

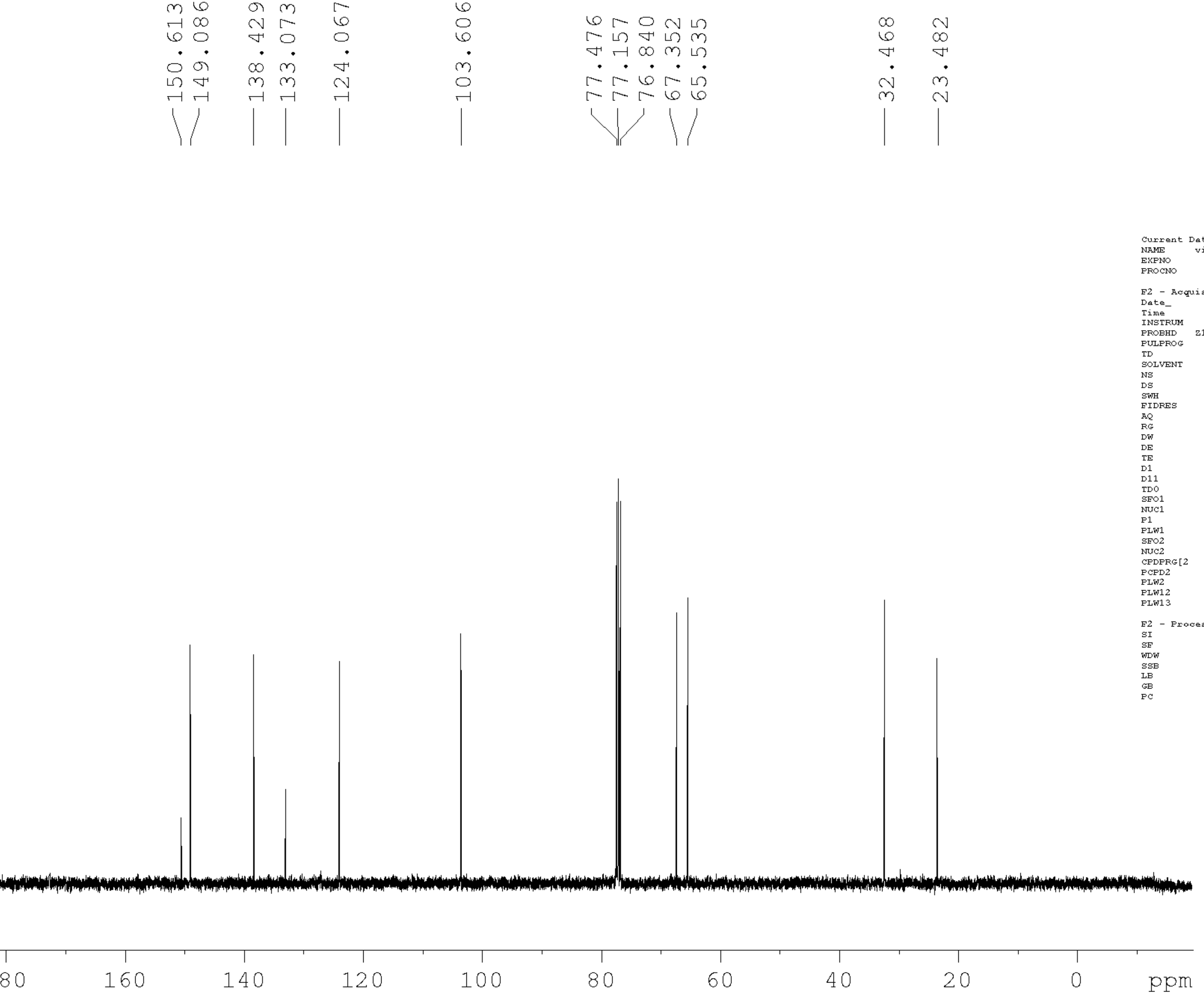


```
Current Data Parameters
NAME      vinn-7-110-2-islt-20230809
EXPNO                    2
PROCNO                   1

F2 - Acquisition Parameters
Date_             20230809
Time                 18.04 h
INSTRUM              spect
PROBHD   Z108618_0257 (
PULPROG            zgpg30
TD                  65536
SOLVENT             CDCl3
NS                     45
DS                      4
SWH             24038.461 Hz
FIDRES           0.733596 Hz
AQ              1.3631488 sec
RG                    203
DW                 20.800 usec
DE                   6.50 usec
TE                  296.2 K
D1             2.00000000 sec
D11            0.03000000 sec
TD0                     1
SFO1          100.6228298 MHz
NUC1                  13C
P1                  10.00 usec
PLW1          51.00000000 W
SFO2          400.1316005 MHz
NUC2                   1H
CPDPRG[2          waltz16
PCPD2               90.00 usec
PLW2          12.50000000 W
PLW12          0.34722000 W
PLW13          0.17465000 W

F2 - Processing parameters
SI                  32768
SF            100.6127594 MHz
WDW                    EM
SSB                     0
LB                   1.00 Hz
GB                      0
PC                   1.40
```

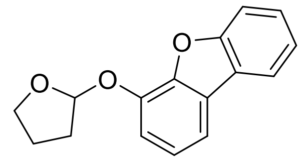

5w

400 MHz ^{1}H NMR

$CDCl_3$

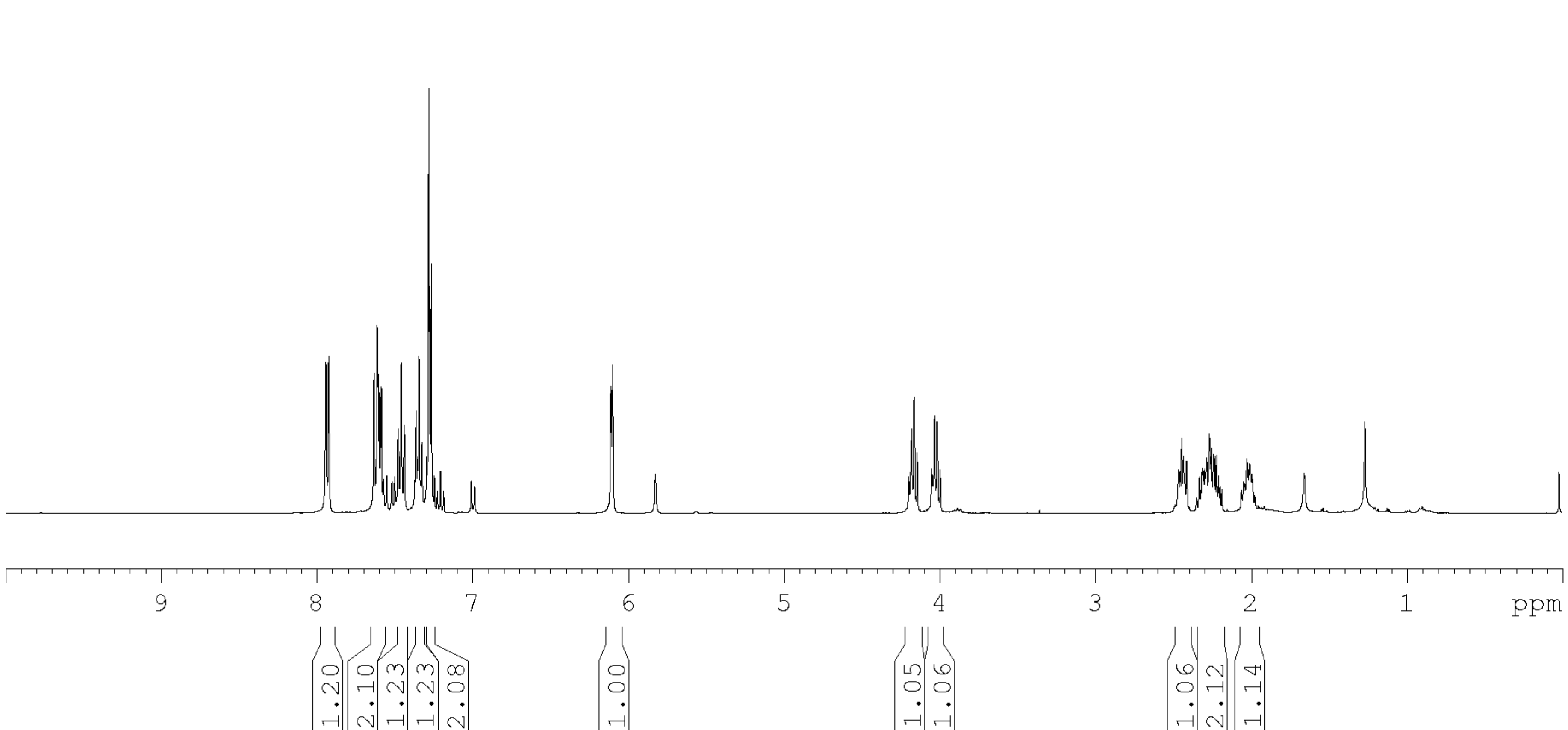


```
Current Data Parameters
NAME     vinn-7-110-4-islt-20230809
EXPNO                 1
PROCNO                1

F2 - Acquisition Parameters
Date_          20230809
Time              18.09 h
INSTRUM           spect
PROBHD   Z108618_0257 (
PULPROG            zg30
TD                65536
SOLVENT           CDCl3
NS                   16
DS                    2
SWH            8012.820 Hz
FIDRES         0.244532 Hz
AQ            4.0894465 sec
RG                 90.5
DW               62.400 usec
DE                 6.50 usec
TE                296.1 K
D1           1.00000000 sec
TD0                   1
SFO1        400.1324708 MHz
NUC1                 1H
P1                15.00 usec
PLW1        12.50000000 W

F2 - Processing parameters
SI                65536
SF          400.1300099 MHz
WDW                  EM
SSB                   0
LB                 0.30 Hz
GB                    0
PC                 1.00
```

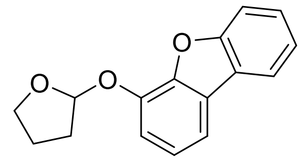

5w

100 MHz ^{13}C NMR

$CDCl_3$

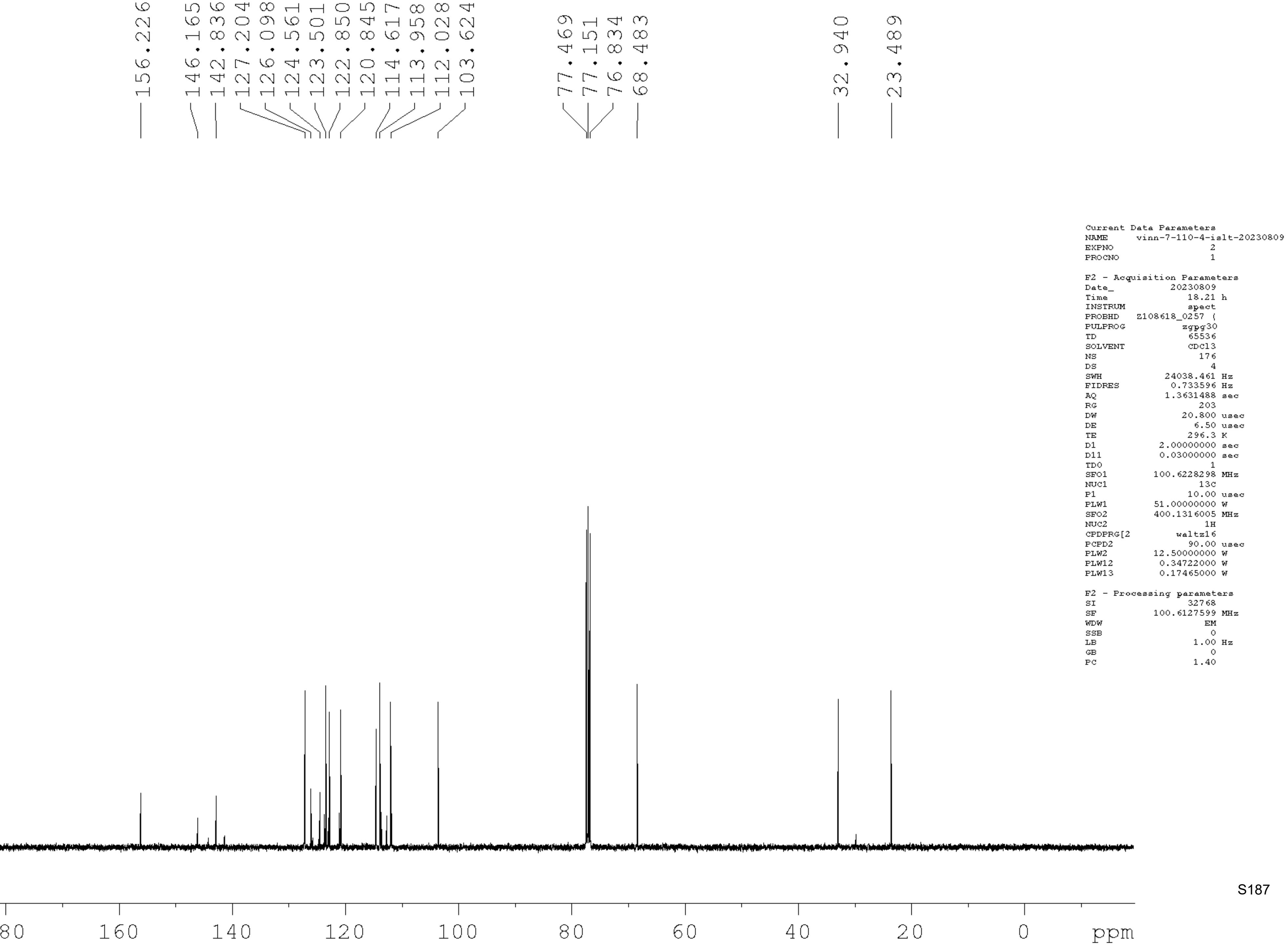

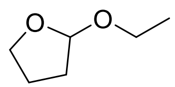

5x

500 MHz ¹H NMR

$CDCl_3$

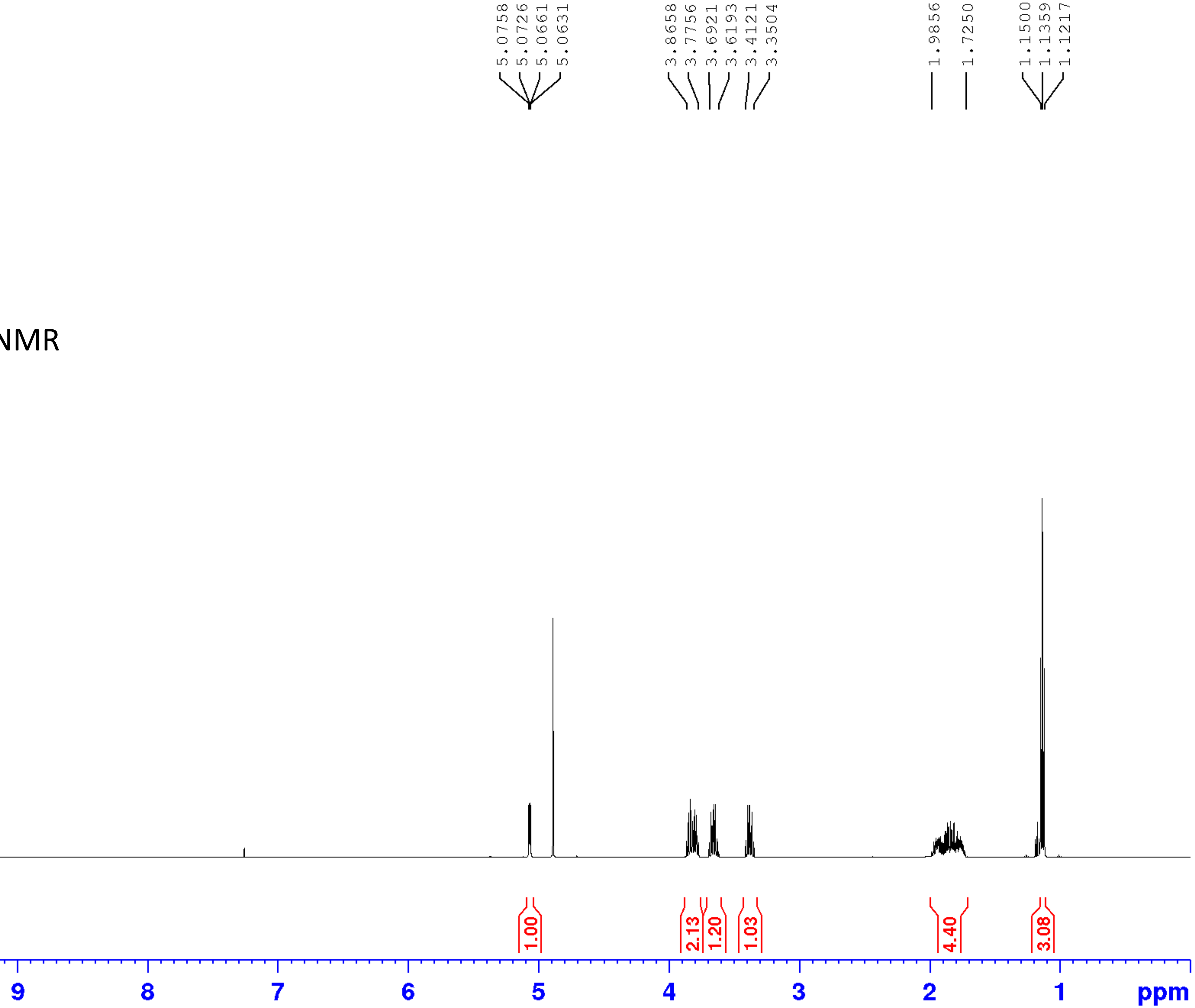


```
Current Data Parameters
NAME     vinn-4-183-6-20h-20230318
EXPNO                    1
PROCNO                   1

F2 - Acquisition Parameters
Date_             20230318
Time                 15.34 h
INSTRUM              spect
PROBHD   Z149001_0010 (
PULPROG               zg30
TD                   65536
SOLVENT              CDCl3
NS                      16
DS                       2
SWH              10000.000 Hz
FIDRES            0.305176 Hz
AQ               3.2767999 sec
RG                   13.21
DW                  50.000 usec
DE                   10.00 usec
TE                   296.1 K
D1              1.00000000 sec
TD0                      1
SFO1           500.1330883 MHz
NUC1                    1H
P1                   11.25 usec
PLW1           17.35199928 W

F2 - Processing parameters
SI                   65536
SF             500.1300121 MHz
WDW                     EM
SSB                      0
LB                    0.30 Hz
GB                       0
PC                    1.00
```

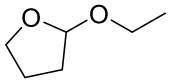

5x
100 MHz ^{13}C NMR
$CDCl_3$

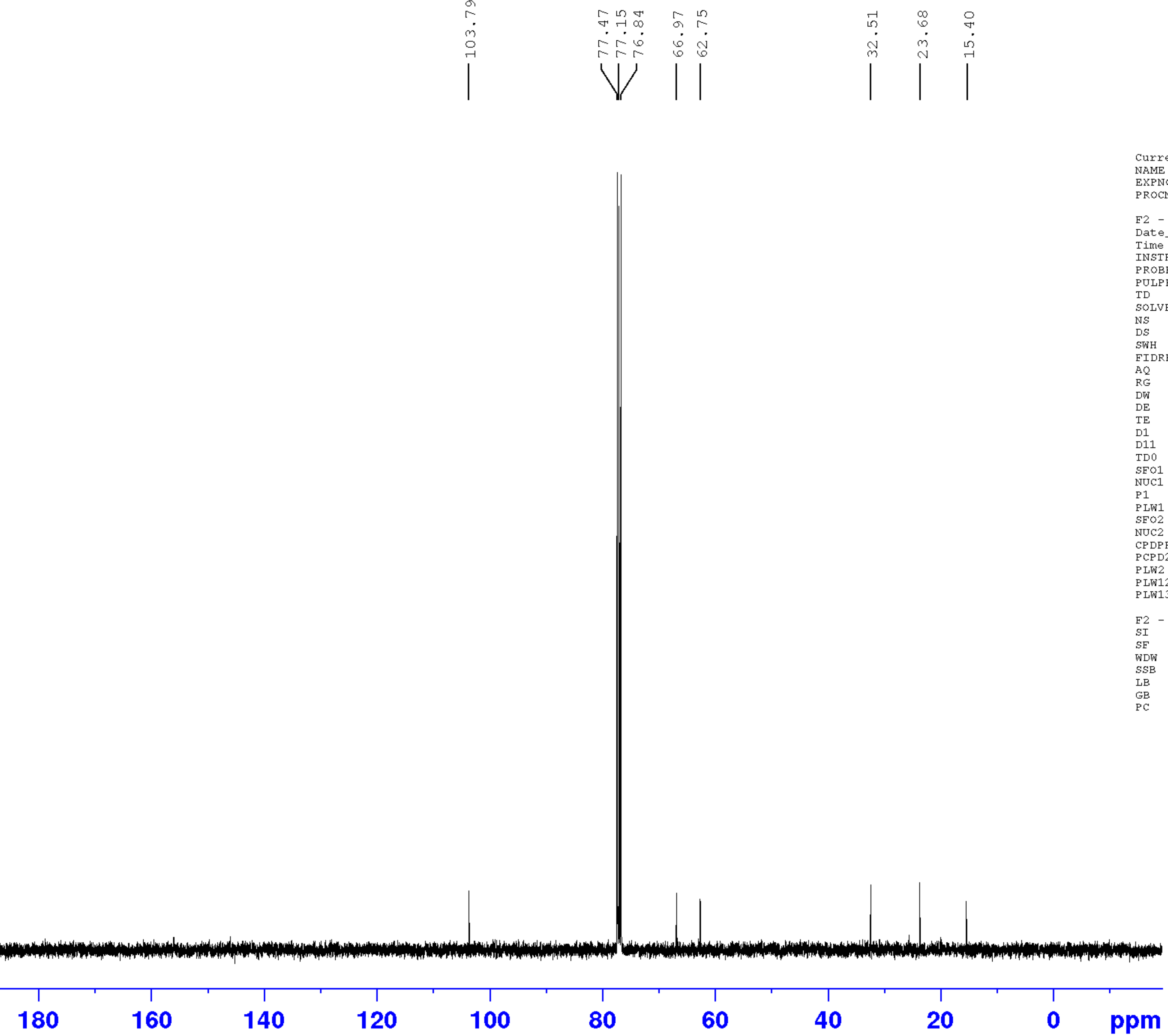


```
Current Data Parameters
NAME     vinn-4-183-6-islt-20230320
EXPNO                     2
PROCNO                    1

F2 - Acquisition Parameters
Date_              20230320
Time                  12.48 h
INSTRUM               spect
PROBHD    Z108618_0257 (
PULPROG             zgpg30
TD                    65536
SOLVENT               CDCl3
NS                      150
DS                        4
SWH               24038.461 Hz
FIDRES             0.733596 Hz
AQ                1.3631488 sec
RG                      203
DW                   20.800 usec
DE                     6.50 usec
TE                    295.9 K
D1               2.00000000 sec
D11              0.03000000 sec
TD0                       1
SFO1            100.6228298 MHz
NUC1                    13C
P1                    10.00 usec
PLW1            51.00000000 W
SFO2            400.1316005 MHz
NUC2                     1H
CPDPRG[2            waltz16
PCPD2                 90.00 usec
PLW2            12.50000000 W
PLW12            0.34722000 W
PLW13            0.17465000 W

F2 - Processing parameters
SI                    32768
SF              100.6127556 MHz
WDW                      EM
SSB                       0
LB                     1.00 Hz
GB                        0
PC                     1.40
```

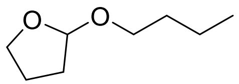

5y

500 MHz ^{1}H NMR

$CDCl_3$

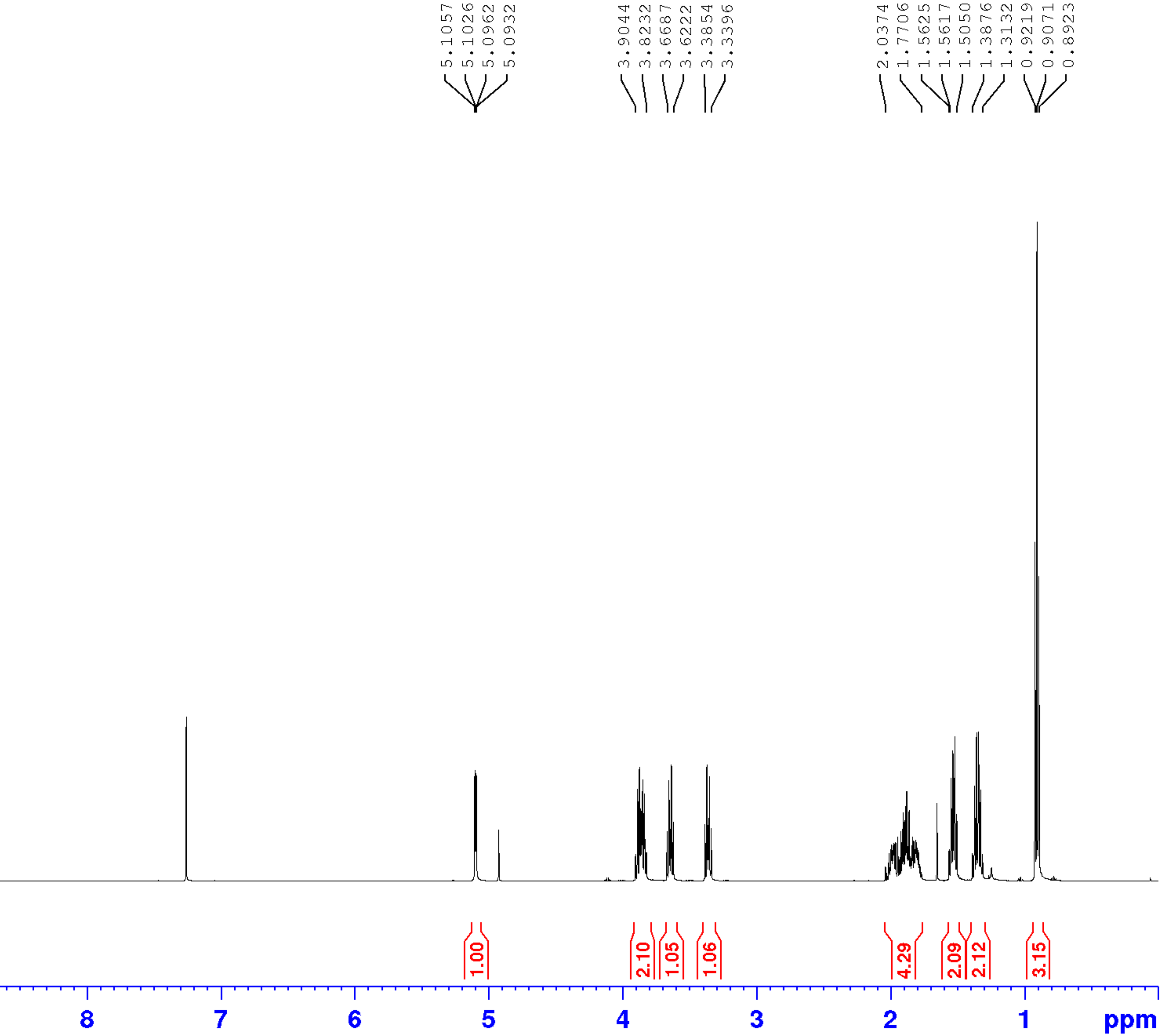


```
Current Data Parameters
NAME      vinn-4-149-4-islt-20200119
EXPNO                    1
PROCNO                   1

F2 - Acquisition Parameters
Date_             20210119
Time                 21.09 h
INSTRUM              spect
PROBHD    Z119470_0283 (
PULPROG               zg30
TD                   65536
SOLVENT              CDCl3
NS                      16
DS                       2
SWH              10000.000 Hz
FIDRES            0.305176 Hz
AQ               3.2767999 sec
RG                   63.76
DW                  50.000 usec
DE                    6.50 usec
TE                   295.1 K
D1              1.00000000 sec
TD0                      1
SFO1           500.1330883 MHz
NUC1                    1H
P1                   10.91 usec
PLW1           25.00000000 W

F2 - Processing parameters
SI                   65536
SF             500.1300121 MHz
WDW                     EM
SSB                      0
LB                    0.30 Hz
GB                       0
PC                    1.00
```

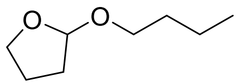

5y

125 MHz ^{13}C NMR

$CDCl_3$

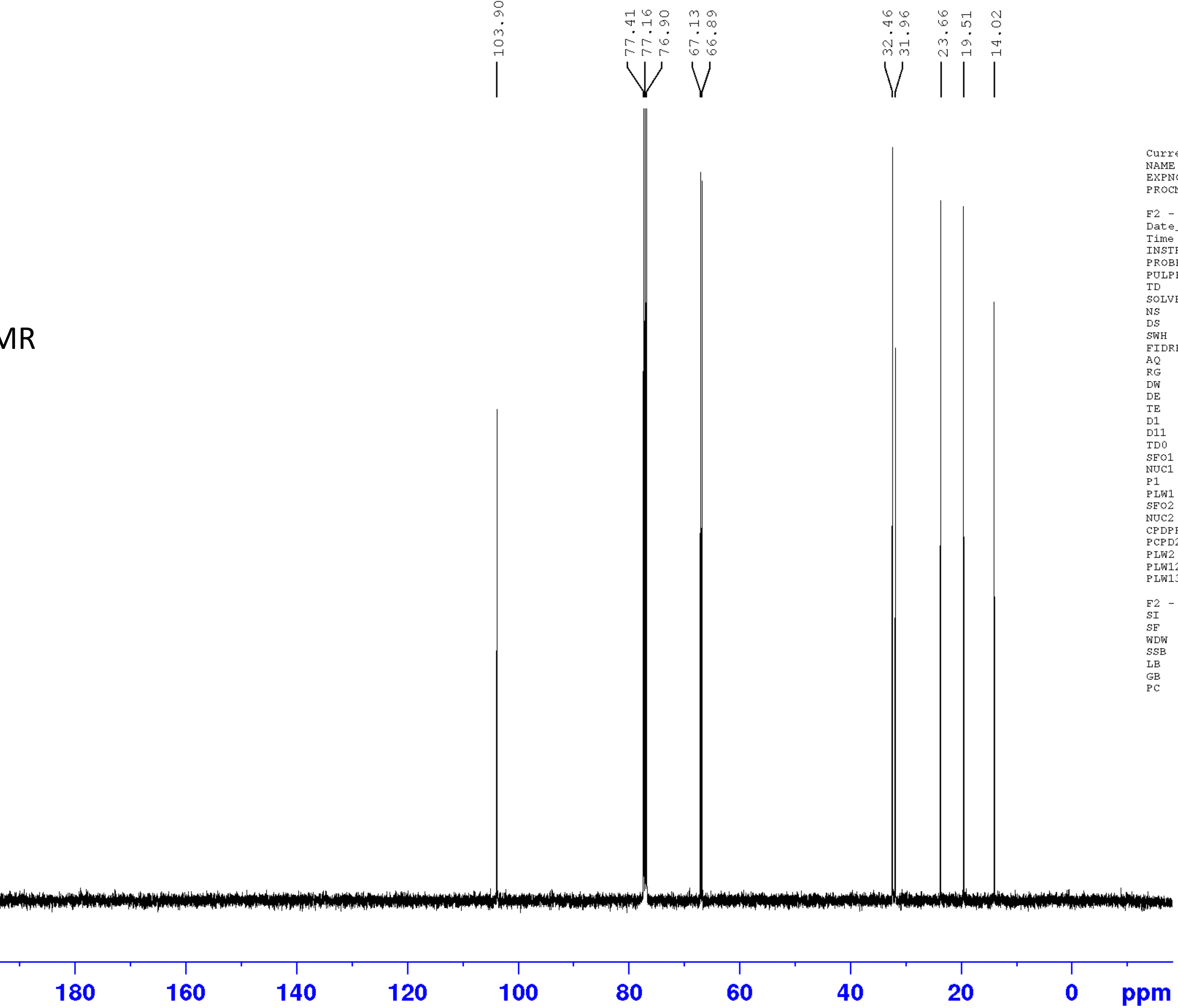


```
Current Data Parameters
NAME      vinn-4-149-4-islt-20210119
EXPNO                  2
PROCNO                 1

F2 - Acquisition Parameters
Date_           20210119
Time               21.23 h
INSTRUM            spect
PROBHD   Z119470_0283 (
PULPROG           zgpg30
TD                 65536
SOLVENT            CDCl3
NS                   256
DS                     4
SWH            29761.904 Hz
FIDRES          0.908261 Hz
AQ             1.1010048 sec
RG                206.72
DW                16.800 usec
DE                  6.50 usec
TE                 295.2 K
D1            2.00000000 sec
D11           0.03000000 sec
TD0                    1
SFO1         125.7703643 MHz
NUC1                 13C
P1                  9.75 usec
PLW1         94.00000000 W
SFO2         500.1320005 MHz
NUC2                  1H
CPDPRG[2         waltz16
PCPD2              80.00 usec
PLW2         25.00000000 W
PLW12         0.46495000 W
PLW13         0.23387000 W

F2 - Processing parameters
SI                 32768
SF           125.7577728 MHz
WDW                   EM
SSB                    0
LB                  1.00 Hz
GB                     0
PC                  1.40
```

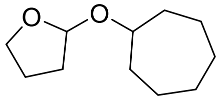

5z

500 MHz ^1H NMR

$CDCl_3$

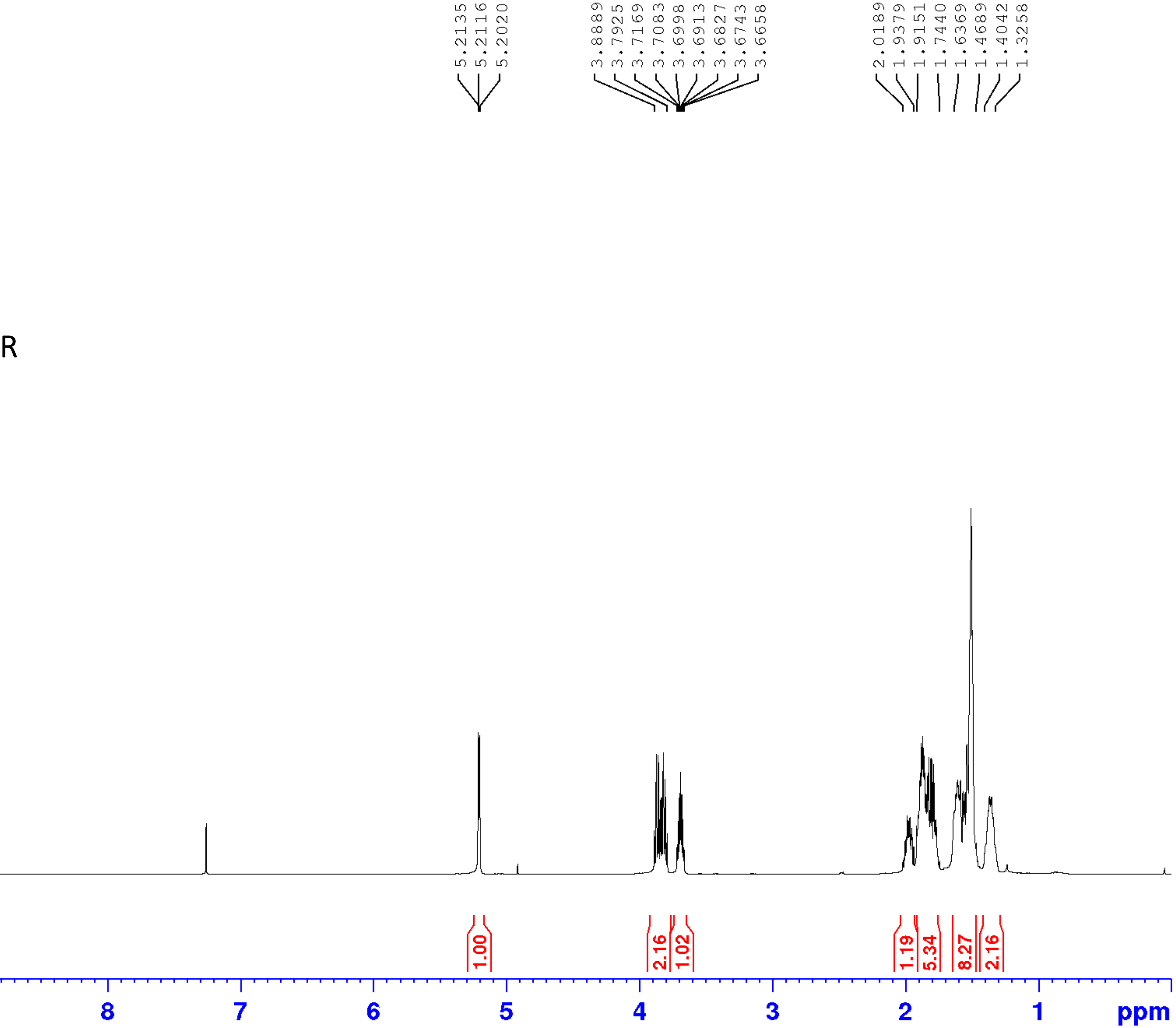

Current Data Parameters
NAME vinn-4-149-8-islt-20200119
EXPNO 1
PROCNO 1

F2 - Acquisition Parameters
Date_ 20210119
Time 22.02 h
INSTRUM spect
PROBHD Z119470_0283 (
PULPROG zg30
TD 65536
SOLVENT CDCl3
NS 16
DS 2
SWH 10000.000 Hz
FIDRES 0.305176 Hz
AQ 3.2767999 sec
RG 30.85
DW 50.000 usec
DE 6.50 usec
TE 295.1 K
D1 1.00000000 sec
TD0 1
SFO1 500.1330883 MHz
NUC1 1H
P1 10.91 usec
PLW1 25.00000000 W

F2 - Processing parameters
SI 65536
SF 500.1300125 MHz
WDW EM
SSB 0
LB 0.30 Hz
GB 0
PC 1.00

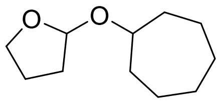

5z

125 MHz ^{13}C NMR

$CDCl_3$

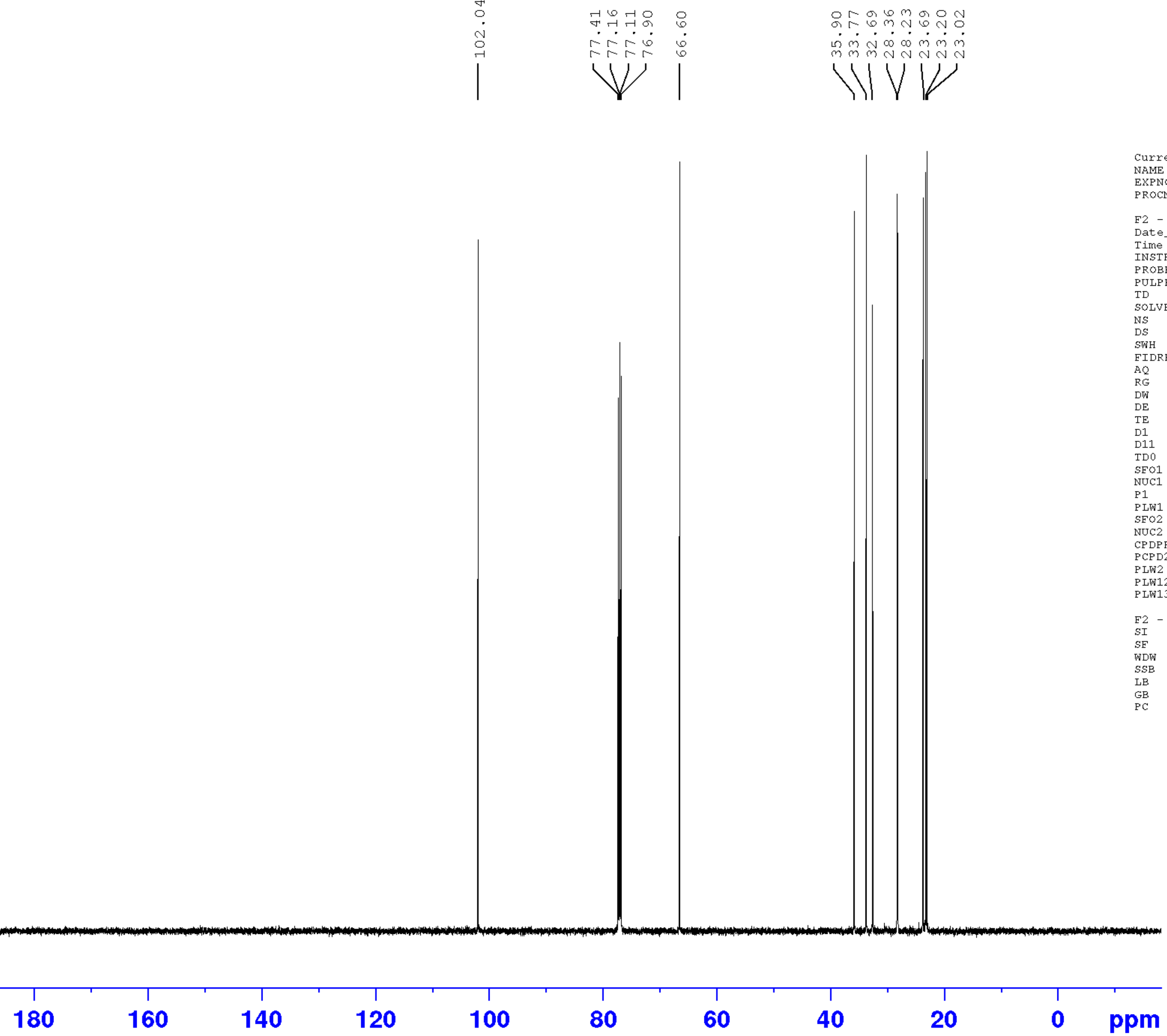


```
Current Data Parameters
NAME       vinn-4-149-8-islt-20200119
EXPNO                      2
PROCNO                     1

F2 - Acquisition Parameters
Date_               20210119
Time                   22.16 h
INSTRUM                spect
PROBHD    Z119470_0283 (
PULPROG              zgpg30
TD                    65536
SOLVENT               CDCl3
NS                      256
DS                        4
SWH               29761.904 Hz
FIDRES             0.908261 Hz
AQ                1.1010048 sec
RG                   206.72
DW                   16.800 usec
DE                     6.50 usec
TE                    295.1 K
D1               2.00000000 sec
D11              0.03000000 sec
TD0                       1
SFO1            125.7703643 MHz
NUC1                    13C
P1                     9.75 usec
PLW1            94.00000000 W
SFO2            500.1320005 MHz
NUC2                     1H
CPDPRG[2            waltz16
PCPD2                 80.00 usec
PLW2            25.00000000 W
PLW12            0.46495000 W
PLW13            0.23387000 W

F2 - Processing parameters
SI                    32768
SF              125.7577754 MHz
WDW                      EM
SSB                       0
LB                     1.00 Hz
GB                        0
PC                     1.40
```

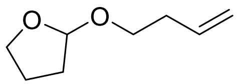

5aa

500 MHz ^{1}H NMR

$CDCl_3$

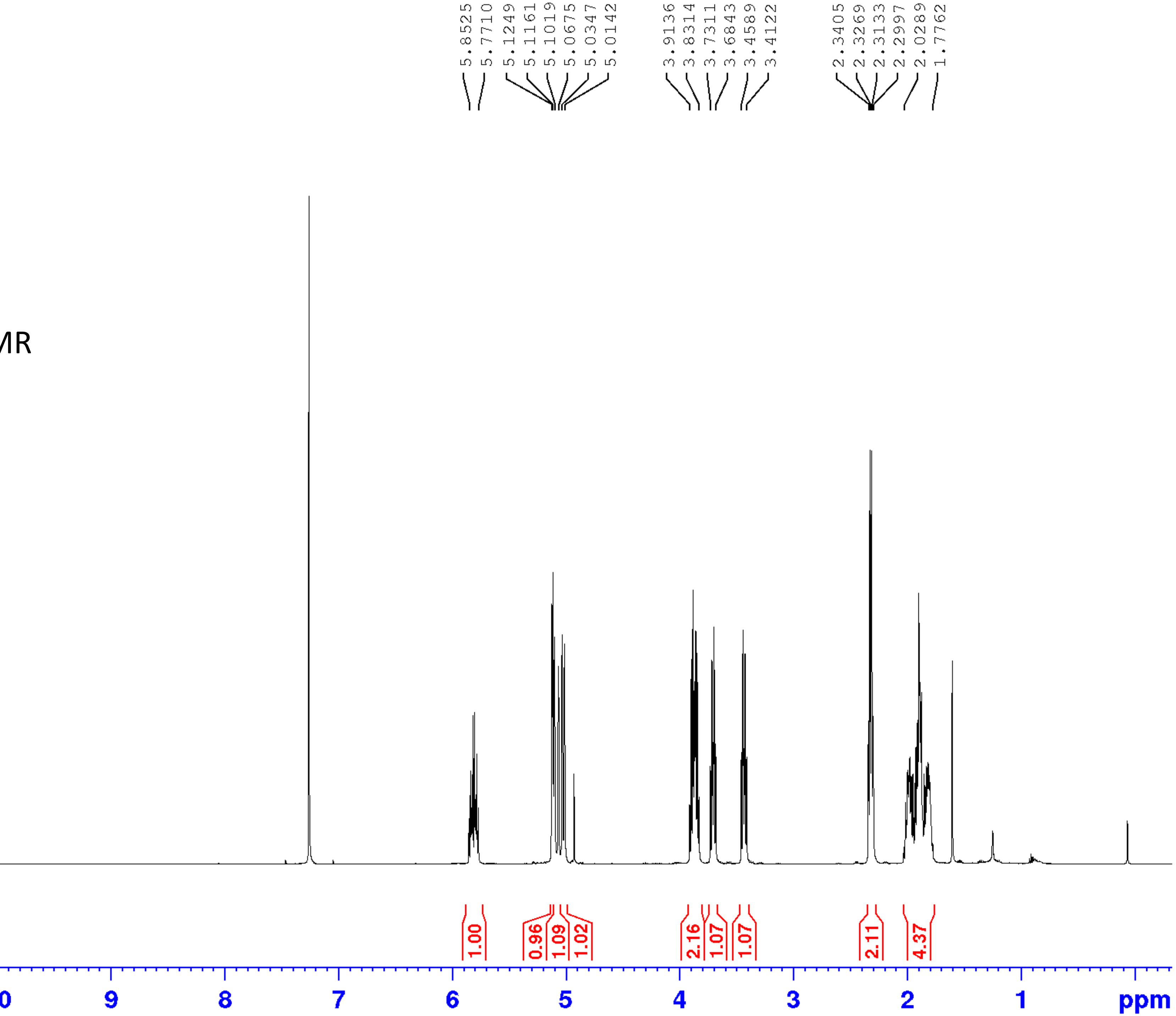


```
Current Data Parameters
NAME     vinn-4-149-13-islt-2020013
EXPNO                     1
PROCNO                    1

F2 - Acquisition Parameters
Date_              20210130
Time                  18.53 h
INSTRUM               spect
PROBHD    Z119470_0283 (
PULPROG                zg30
TD                    65536
SOLVENT               CDCl3
NS                       16
DS                        2
SWH           10000.000 Hz
FIDRES         0.305176 Hz
AQ            3.2767999 sec
RG                    93.28
DW               50.000 usec
DE                 6.50 usec
TE                295.1 K
D1           1.00000000 sec
TD0                       1
SFO1        500.1330883 MHz
NUC1                     1H
P1                10.91 usec
PLW1        25.00000000 W

F2 - Processing parameters
SI                    65536
SF          500.1300122 MHz
WDW                      EM
SSB                       0
LB                     0.30 Hz
GB                        0
PC                     1.00
```

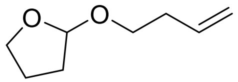

5aa

125 MHz ^{13}C NMR

$CDCl_3$

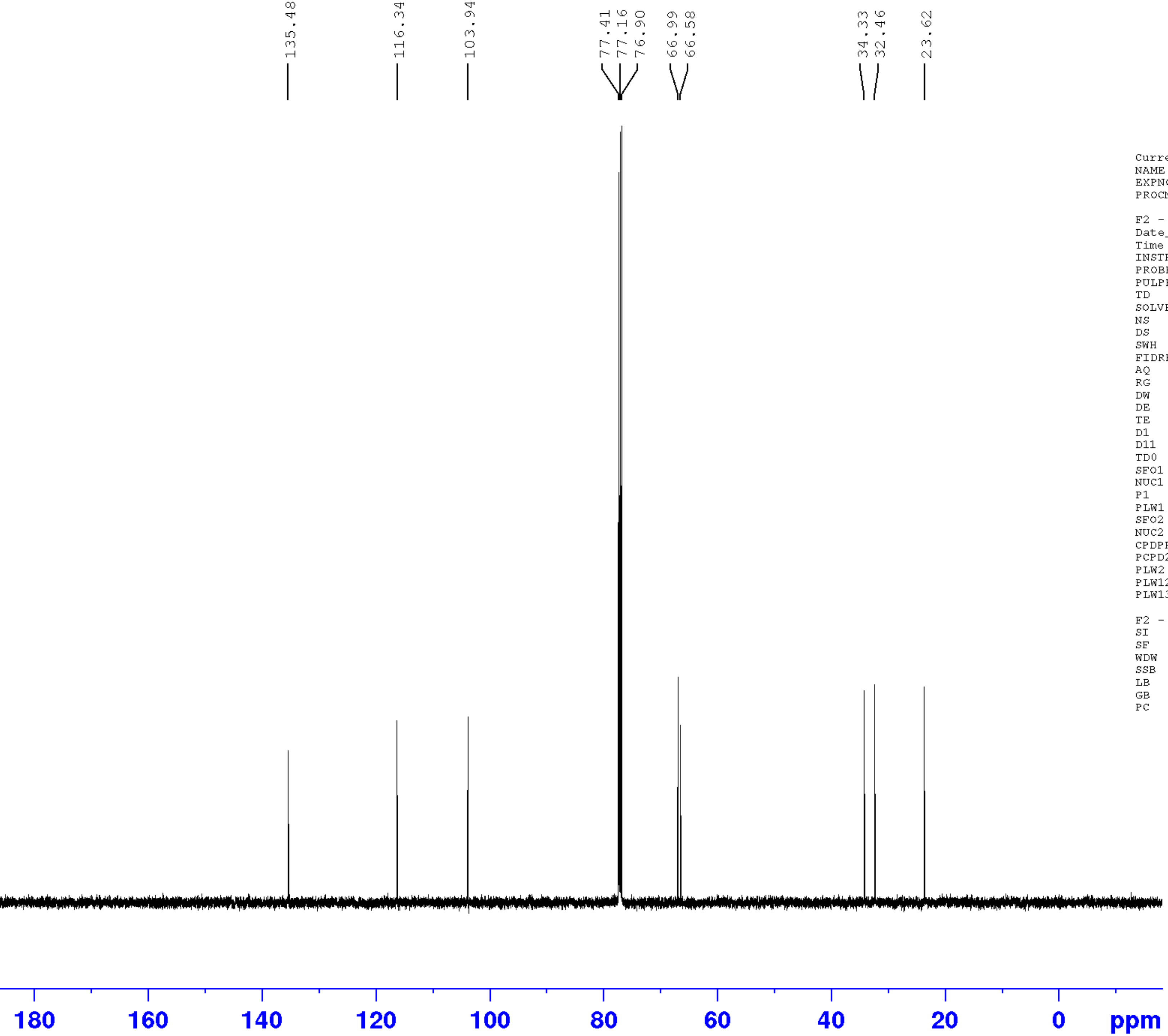


```
Current Data Parameters
NAME      vinn-4-149-13-islt-20200130
EXPNO                    2
PROCNO                   1

F2 - Acquisition Parameters
Date_             20210130
Time               19.00 h
INSTRUM              spect
PROBHD   Z119470_0283 (
PULPROG            zgpg30
TD                  65536
SOLVENT             CDCl3
NS                    128
DS                      4
SWH             29761.904 Hz
FIDRES           0.908261 Hz
AQ              1.1010048 sec
RG                 206.72
DW                 16.800 usec
DE                   6.50 usec
TE                  295.2 K
D1             2.00000000 sec
D11            0.03000000 sec
TD0                     1
SFO1          125.7703643 MHz
NUC1                  13C
P1                   9.75 usec
PLW1          94.00000000 W
SFO2          500.1320005 MHz
NUC2                   1H
CPDPRG[2          waltz16
PCPD2               80.00 usec
PLW2          25.00000000 W
PLW12          0.46495000 W
PLW13          0.23387000 W

F2 - Processing parameters
SI                  32768
SF            125.7577727 MHz
WDW                    EM
SSB                     0
LB                   1.00 Hz
GB                      0
PC                   1.40
```

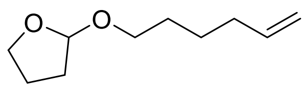

5ab

500 MHz ^{1}H NMR

$CDCl_3$

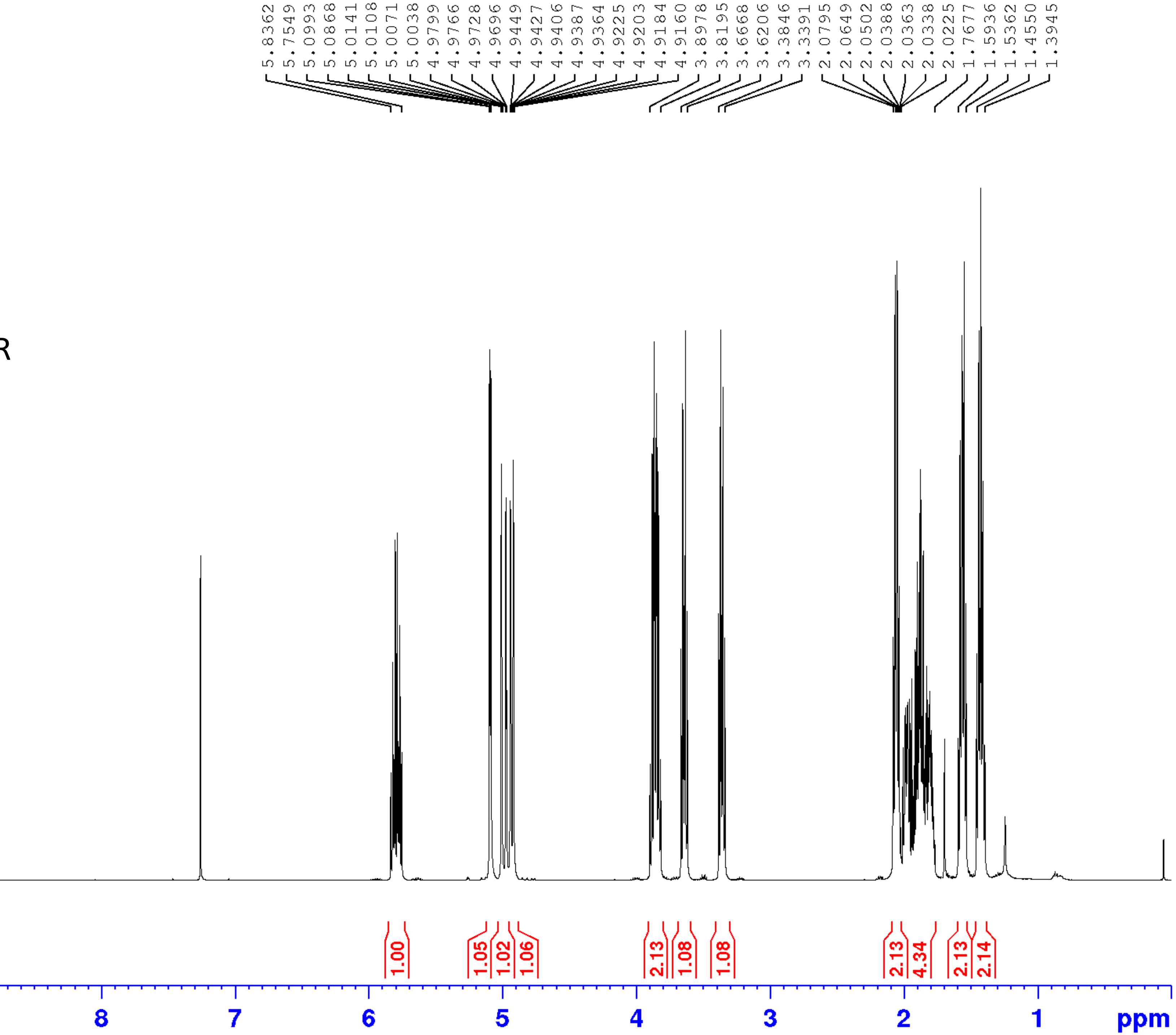


```
Current Data Parameters
NAME     vinn-4-149-9-islt-20200119
EXPNO                     1
PROCNO                    1

F2 - Acquisition Parameters
Date_              20210119
Time                  22.20 h
INSTRUM               spect
PROBHD   Z119470_0283 (
PULPROG                zg30
TD                    65536
SOLVENT               CDCl3
NS                       16
DS                        2
SWH            10000.000 Hz
FIDRES          0.305176 Hz
AQ             3.2767999 sec
RG                    30.85
DW                50.000 usec
DE                  6.50 usec
TE                  295.1 K
D1            1.00000000 sec
TD0                       1
SFO1         500.1330883 MHz
NUC1                     1H
P1                 10.91 usec
PLW1         25.00000000 W

F2 - Processing parameters
SI                    65536
SF           500.1300123 MHz
WDW                      EM
SSB                       0
LB                     0.30 Hz
GB                        0
PC                     1.00
```

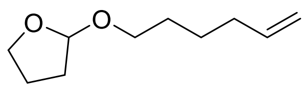

5ab

125 MHz ^{13}C NMR

$CDCl_3$

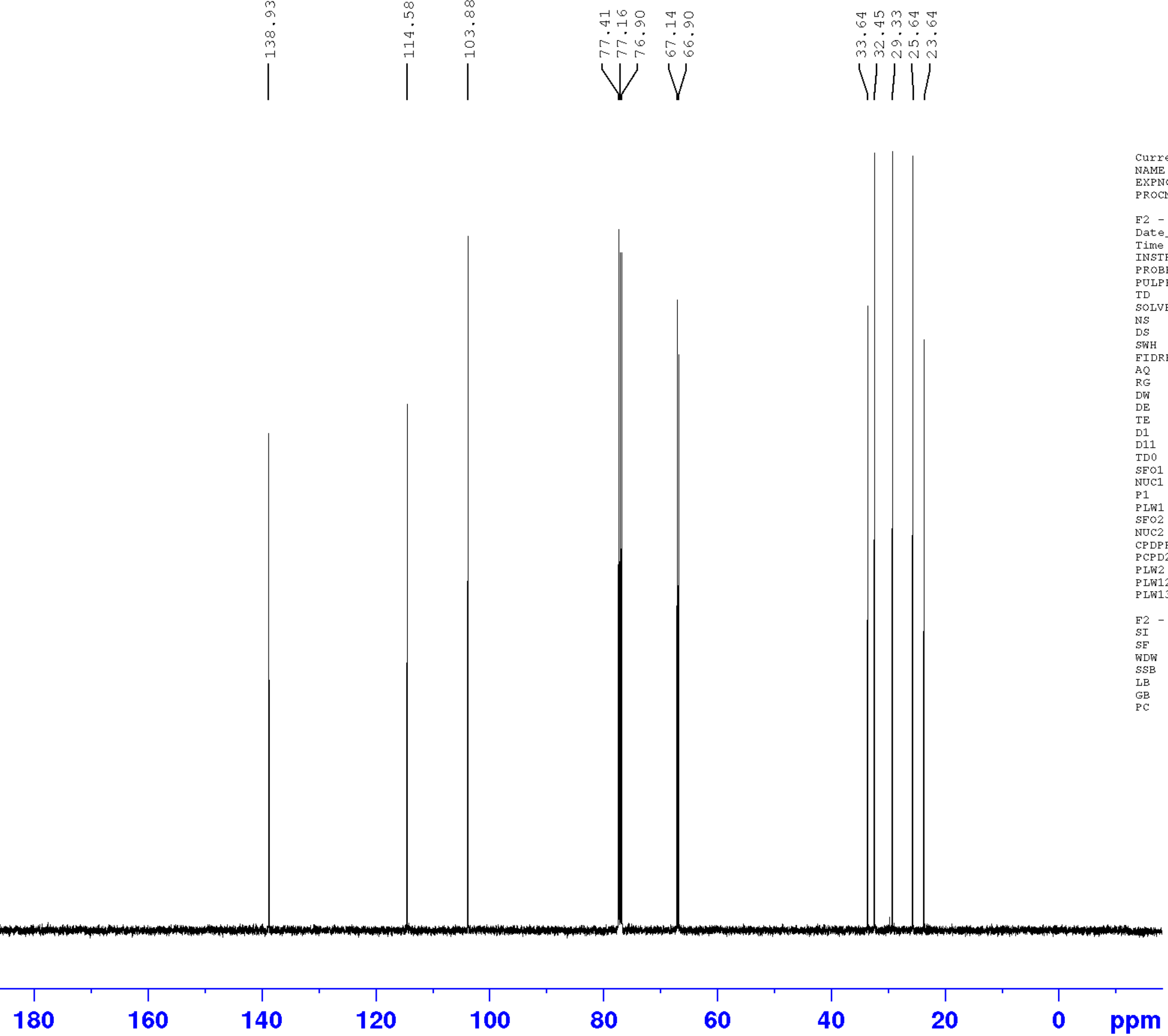


```
Current Data Parameters
NAME     vinn-4-149-9-islt-20200119
EXPNO                     2
PROCNO                    1

F2 - Acquisition Parameters
Date_              20210119
Time                  22.34 h
INSTRUM               spect
PROBHD   Z119470_0283 (
PULPROG              zgpg30
TD                    65536
SOLVENT               CDCl3
NS                      256
DS                        4
SWH              29761.904 Hz
FIDRES            0.908261 Hz
AQ               1.1010048 sec
RG                   206.72
DW                   16.800 usec
DE                     6.50 usec
TE                    295.1 K
D1              2.00000000 sec
D11             0.03000000 sec
TD0                       1
SFO1           125.7703643 MHz
NUC1                    13C
P1                     9.75 usec
PLW1           94.00000000 W
SFO2           500.1320005 MHz
NUC2                     1H
CPDPRG[2            waltz16
PCPD2                 80.00 usec
PLW2           25.00000000 W
PLW12           0.46495000 W
PLW13           0.23387000 W

F2 - Processing parameters
SI                    32768
SF             125.7577740 MHz
WDW                      EM
SSB                       0
LB                     1.00 Hz
GB                        0
PC                     1.40
```

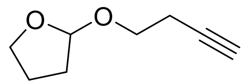

5ac

500 MHz ^{1}H NMR

$CDCl_3$

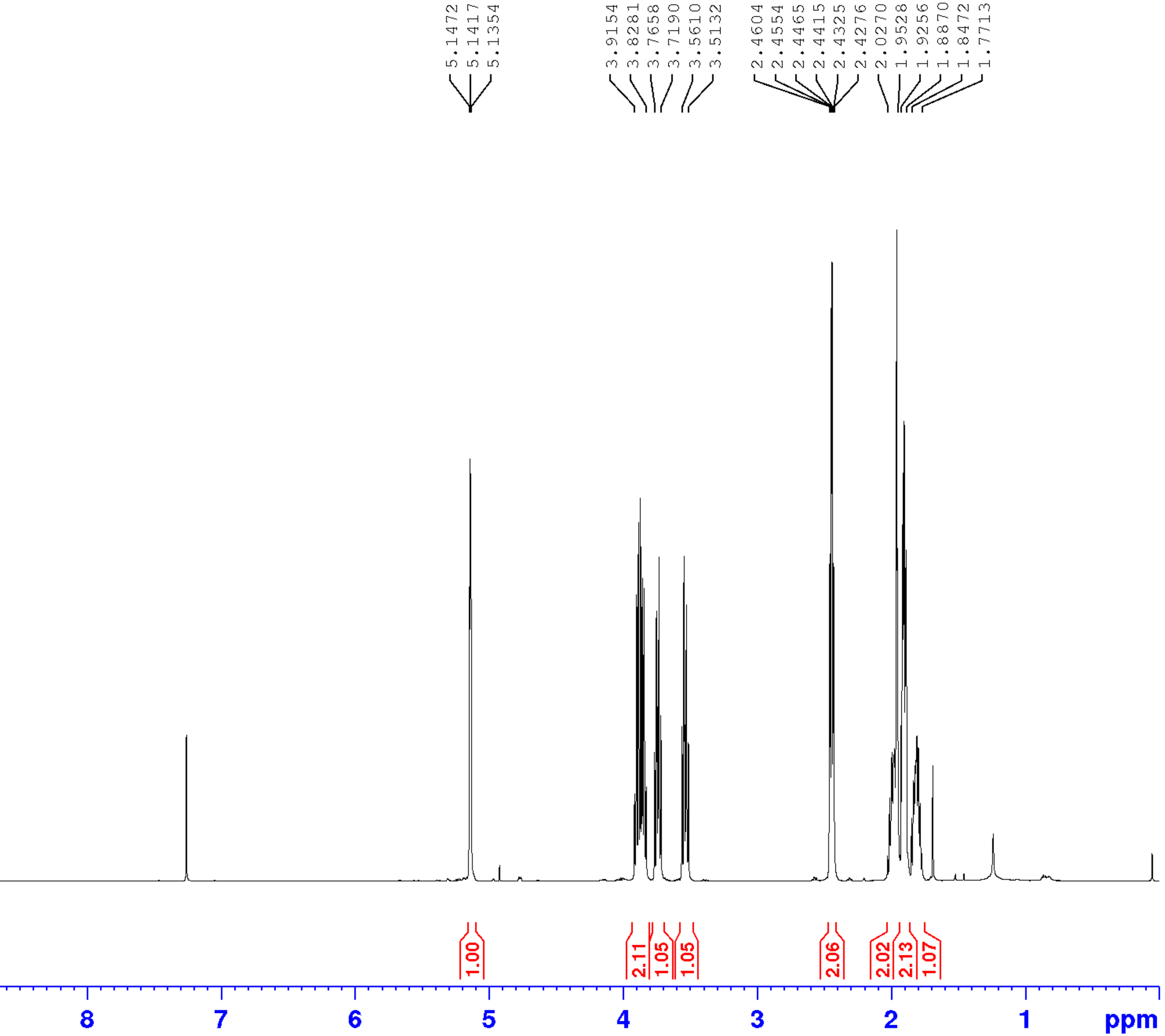


```
Current Data Parameters
NAME      vinn-4-149-11-islt-2020013
EXPNO                 1
PROCNO                1

F2 - Acquisition Parameters
Date_          20210130
Time              18.31 h
INSTRUM           spect
PROBHD    Z119470_0283 (
PULPROG            zg30
TD                65536
SOLVENT           CDCl3
NS                   16
DS                    2
SWH           10000.000 Hz
FIDRES         0.305176 Hz
AQ            3.2767999 sec
RG                56.83
DW               50.000 usec
DE                 6.50 usec
TE                295.2 K
D1           1.00000000 sec
TD0                   1
SFO1        500.1330883 MHz
NUC1                 1H
P1                10.91 usec
PLW1        25.00000000 W

F2 - Processing parameters
SI                65536
SF          500.1300123 MHz
WDW                  EM
SSB                   0
LB                 0.30 Hz
GB                    0
PC                 1.00
```

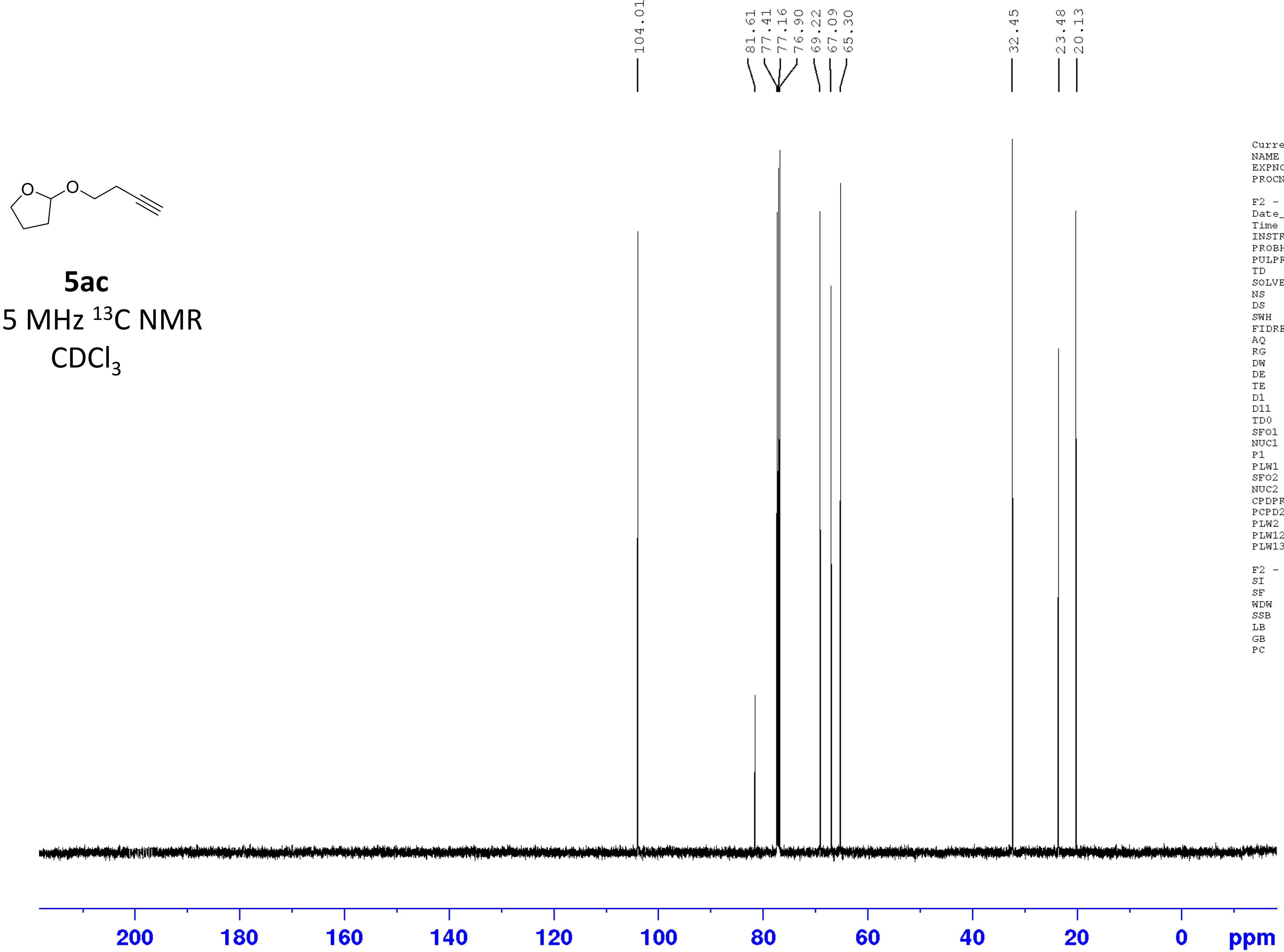

5ac

125 MHz ^{13}C NMR

$CDCl_3$

```
Current Data Parameters
NAME      vinn-4-149-11-islt-20200130
EXPNO                    2
PROCNO                   1

F2 - Acquisition Parameters
Date_             20210130
Time                 18.38 h
INSTRUM              spect
PROBHD   Z119470_0283 (
PULPROG            zgpg30
TD                  65536
SOLVENT             CDCl3
NS                    128
DS                      4
SWH             29761.904 Hz
FIDRES           0.908261 Hz
AQ              1.1010048 sec
RG                 206.72
DW                 16.800 usec
DE                   6.50 usec
TE                  295.2 K
D1             2.00000000 sec
D11            0.03000000 sec
TD0                     1
SFO1          125.7703643 MHz
NUC1                  13C
P1                   9.75 usec
PLW1          94.00000000 W
SFO2          500.1320005 MHz
NUC2                   1H
CPDPRG[2          waltz16
PCPD2               80.00 usec
PLW2          25.00000000 W
PLW12          0.46495000 W
PLW13          0.23387000 W

F2 - Processing parameters
SI                  32768
SF            125.7577747 MHz
WDW                    EM
SSB                     0
LB                   1.00 Hz
GB                      0
PC                   1.40
```

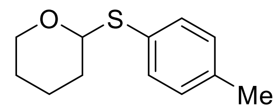

6a

400 MHz ^{1}H NMR

$CDCl_3$

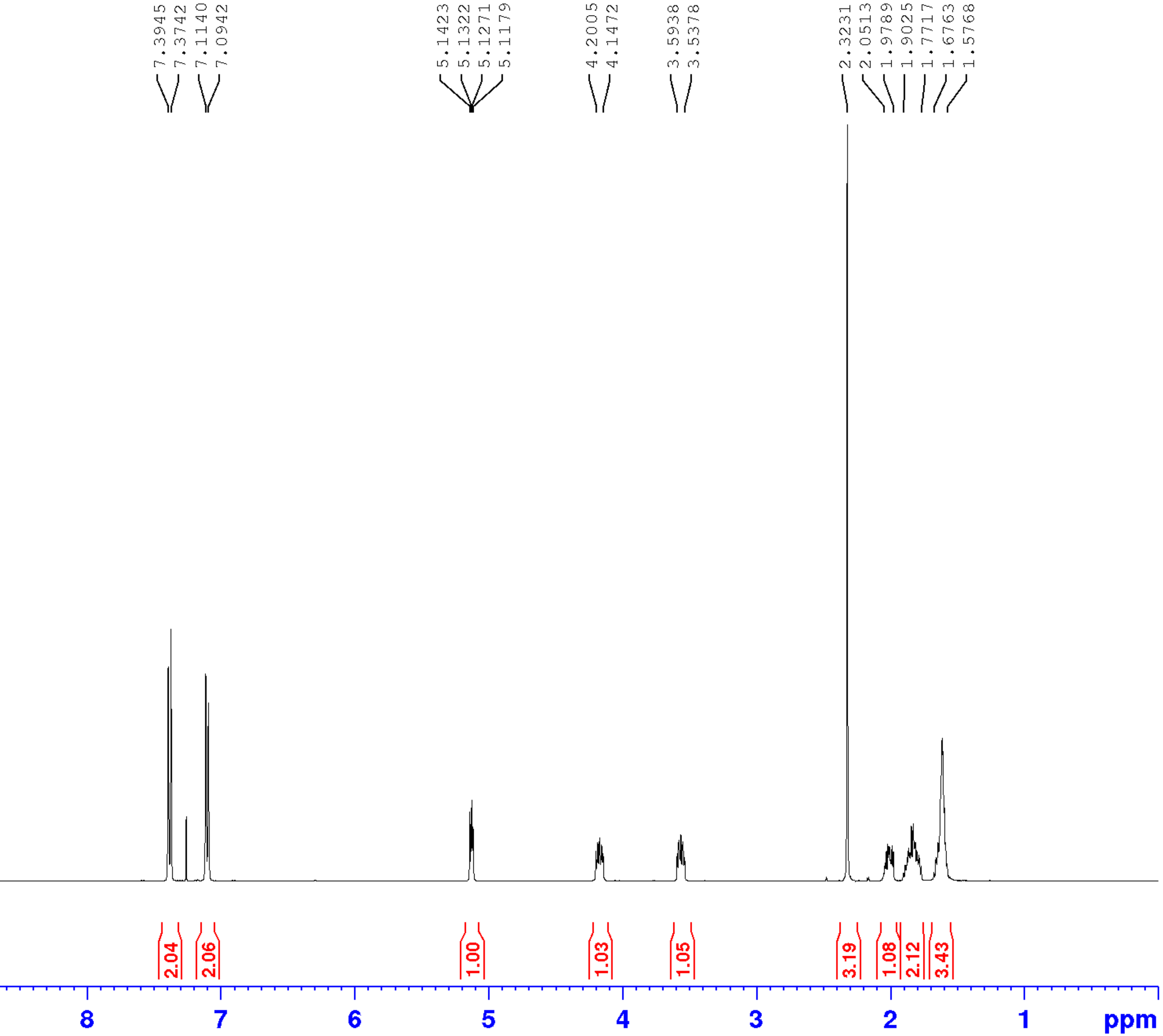

Current Data Parameters
NAME Andy-1-181-1-islt-2-20220812
EXPNO 4
PROCNO 1

F2 - Acquisition Parameters
Date_ 20220812
Time 18.56 h
INSTRUM spect
PROBHD Z108618_0257 (
PULPROG zg30
TD 65536
SOLVENT CDCl3
NS 16
DS 2
SWH 8012.820 Hz
FIDRES 0.244532 Hz
AQ 4.0894465 sec
RG 114
DW 62.400 usec
DE 6.50 usec
TE 295.6 K
D1 1.00000000 sec
TD0 1
SFO1 400.1324708 MHz
NUC1 1H
P1 15.00 usec
PLW1 12.50000000 W

F2 - Processing parameters
SI 65536
SF 400.1300098 MHz
WDW EM
SSB 0
LB 0.30 Hz
GB 0
PC 1.00

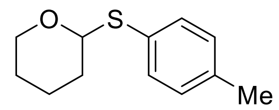

6a

100 MHz ^{13}C NMR

$CDCl_3$

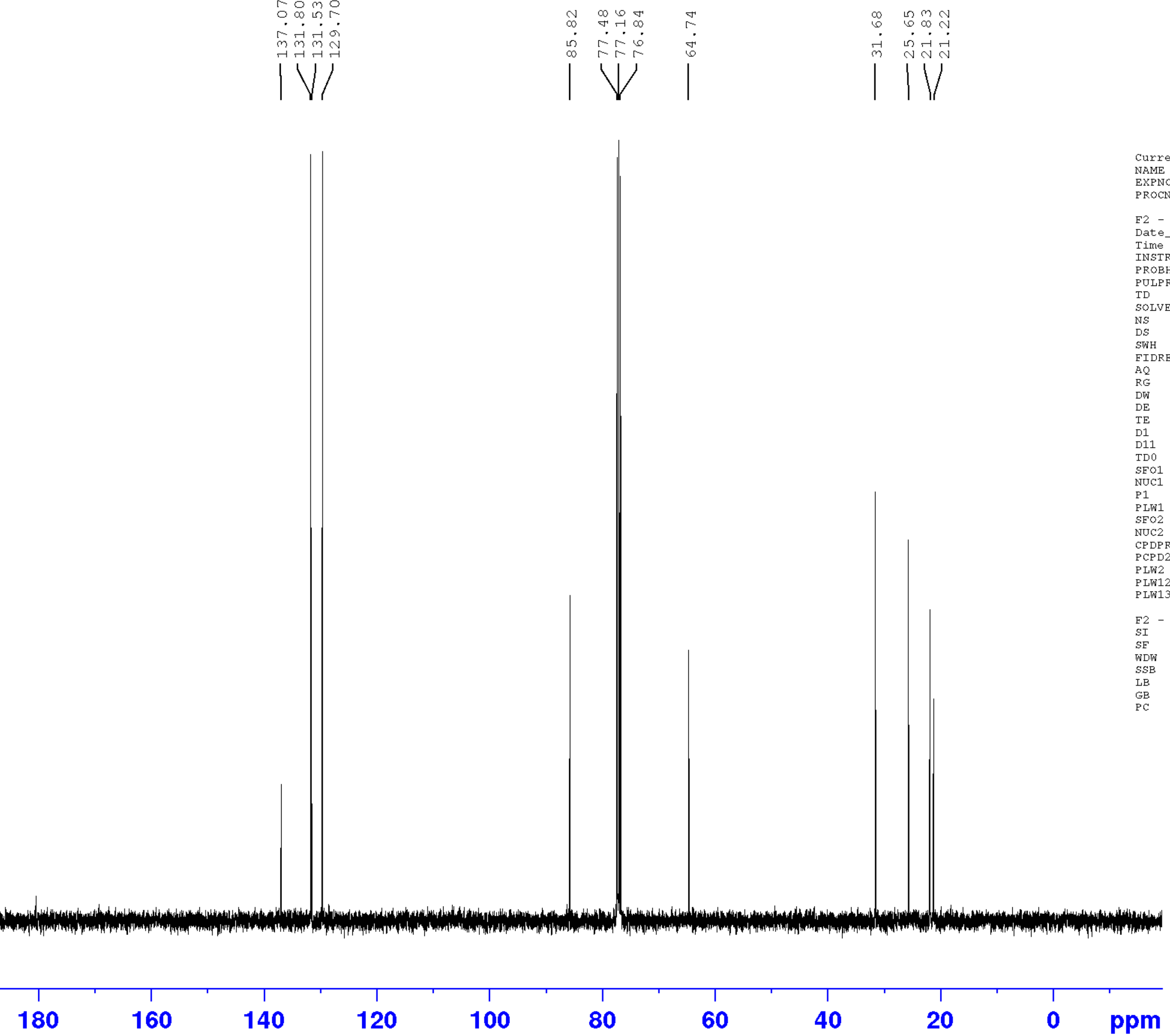


```
Current Data Parameters
NAME     Andy-1-181-1-islt-2-20220812
EXPNO                   5
PROCNO                  1

F2 - Acquisition Parameters
Date_           20220812
Time               19.04 h
INSTRUM            spect
PROBHD   Z108618_0257 (
PULPROG           zgpg30
TD                 65536
SOLVENT            CDCl3
NS                   102
DS                     4
SWH            24038.461 Hz
FIDRES          0.733596 Hz
AQ             1.3631488 sec
RG                   203
DW                20.800 usec
DE                  6.50 usec
TE                 296.0 K
D1            2.00000000 sec
D11           0.03000000 sec
TD0                    1
SFO1         100.6228298 MHz
NUC1                 13C
P1                 10.00 usec
PLW1         51.00000000 W
SFO2         400.1316005 MHz
NUC2                  1H
CPDPRG[2         waltz16
PCPD2              90.00 usec
PLW2         12.50000000 W
PLW12         0.34722000 W
PLW13         0.17465000 W

F2 - Processing parameters
SI                 32768
SF           100.6127579 MHz
WDW                   EM
SSB                    0
LB                  1.00 Hz
GB                     0
PC                  1.40
```

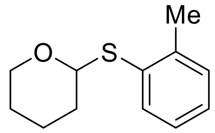

6b

400 MHz ^{1}H NMR

$CDCl_3$

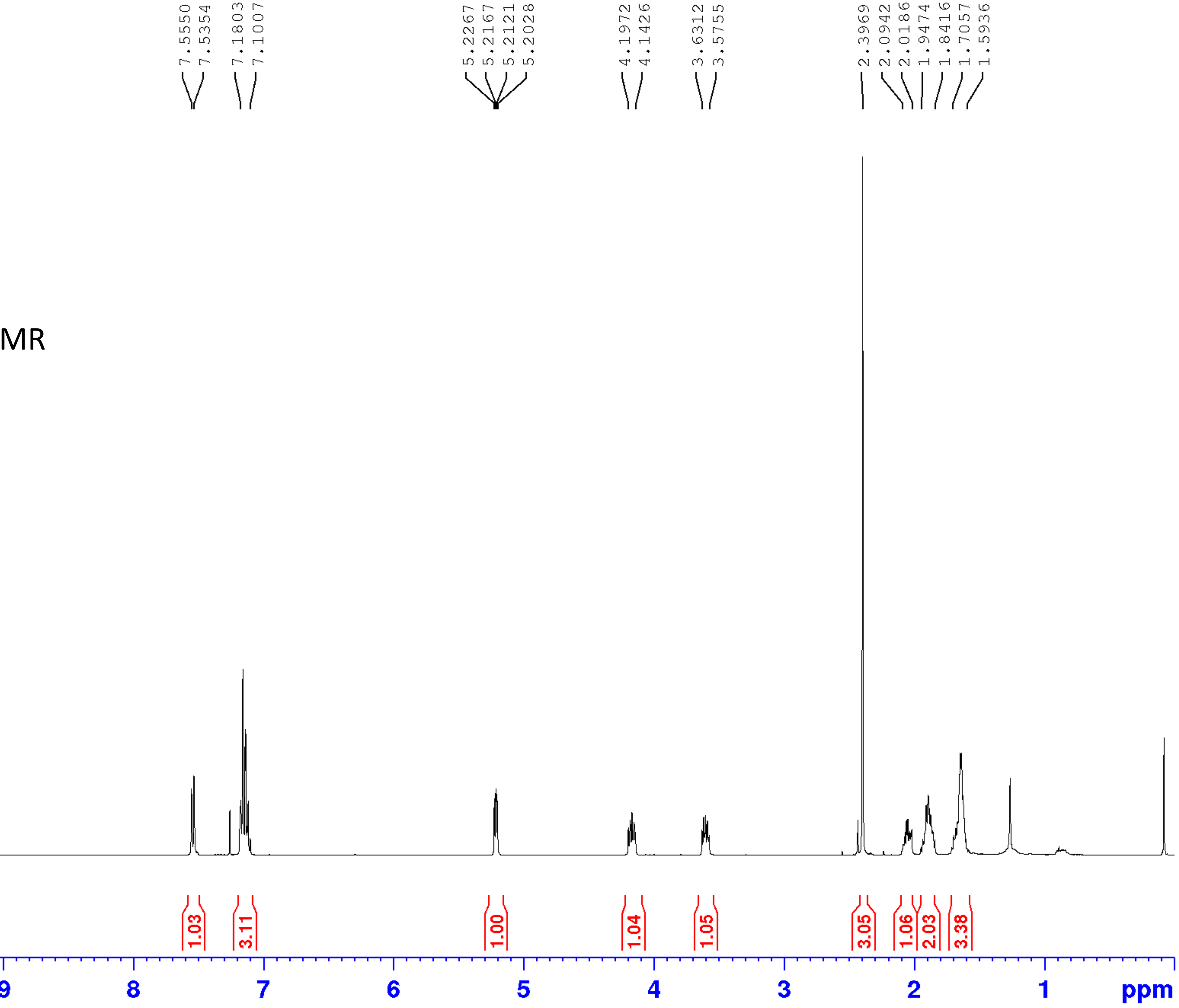


```
Current Data Parameters
NAME     Andy-181-4-islt-20220817
EXPNO                   4
PROCNO                  1

F2 - Acquisition Parameters
Date_          20220817
Time              19.08 h
INSTRUM           spect
PROBHD   Z108618_0257 (
PULPROG            zg30
TD                65536
SOLVENT           CDCl3
NS                   16
DS                    2
SWH            8012.820 Hz
FIDRES         0.244532 Hz
AQ            4.0894465 sec
RG                 90.5
DW               62.400 usec
DE                 6.50 usec
TE                295.6 K
D1          1.00000000 sec
TD0                   1
SFO1        400.1324708 MHz
NUC1                 1H
P1                15.00 usec
PLW1        12.50000000 W

F2 - Processing parameters
SI                65536
SF          400.1300108 MHz
WDW                  EM
SSB                   0
LB                 0.30 Hz
GB                    0
PC                 1.00
```

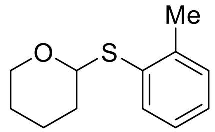

6b

100 MHz ^{13}C NMR

$CDCl_3$

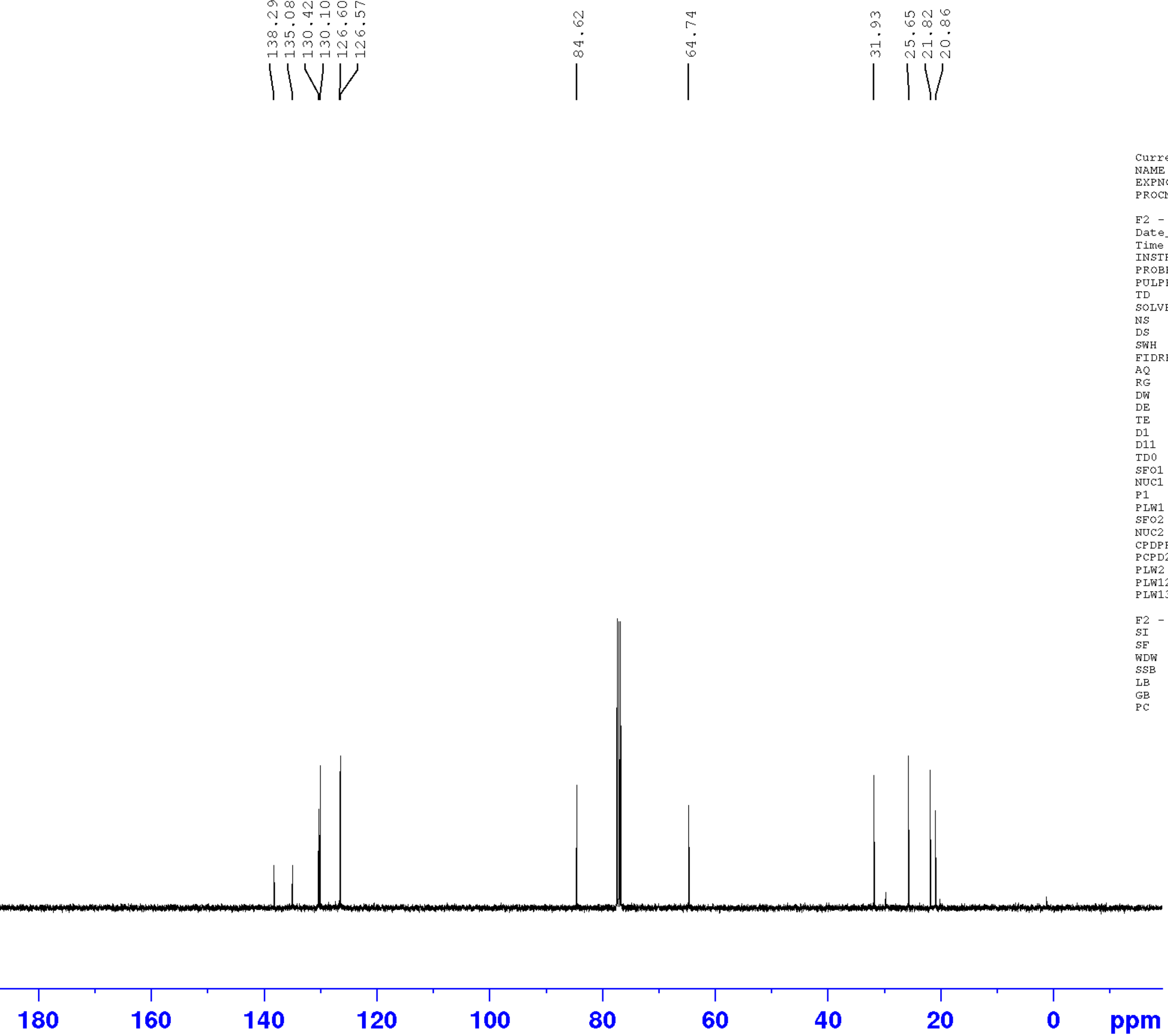


```
Current Data Parameters
NAME     Andy-181-4-islt-20220817
EXPNO                    5
PROCNO                   1

F2 - Acquisition Parameters
Date_           20220817
Time              19.17 h
INSTRUM           spect
PROBHD   Z108618_0257 (
PULPROG          zgpg30
TD                65536
SOLVENT           CDCl3
NS                  106
DS                    4
SWH           24038.461 Hz
FIDRES         0.733596 Hz
AQ            1.3631488 sec
RG                  203
DW               20.800 usec
DE                 6.50 usec
TE                296.3 K
D1           2.00000000 sec
D11          0.03000000 sec
TD0                   1
SFO1        100.6228298 MHz
NUC1                13C
P1                10.00 usec
PLW1        51.00000000 W
SFO2        400.1316005 MHz
NUC2                 1H
CPDPRG[2        waltz16
PCPD2             90.00 usec
PLW2        12.50000000 W
PLW12        0.34722000 W
PLW13        0.17465000 W

F2 - Processing parameters
SI                32768
SF          100.6127582 MHz
WDW                  EM
SSB                   0
LB                 1.00 Hz
GB                    0
PC                 1.40
```

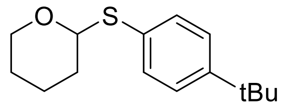

6c

400 MHz ^{1}H NMR

$CDCl_3$

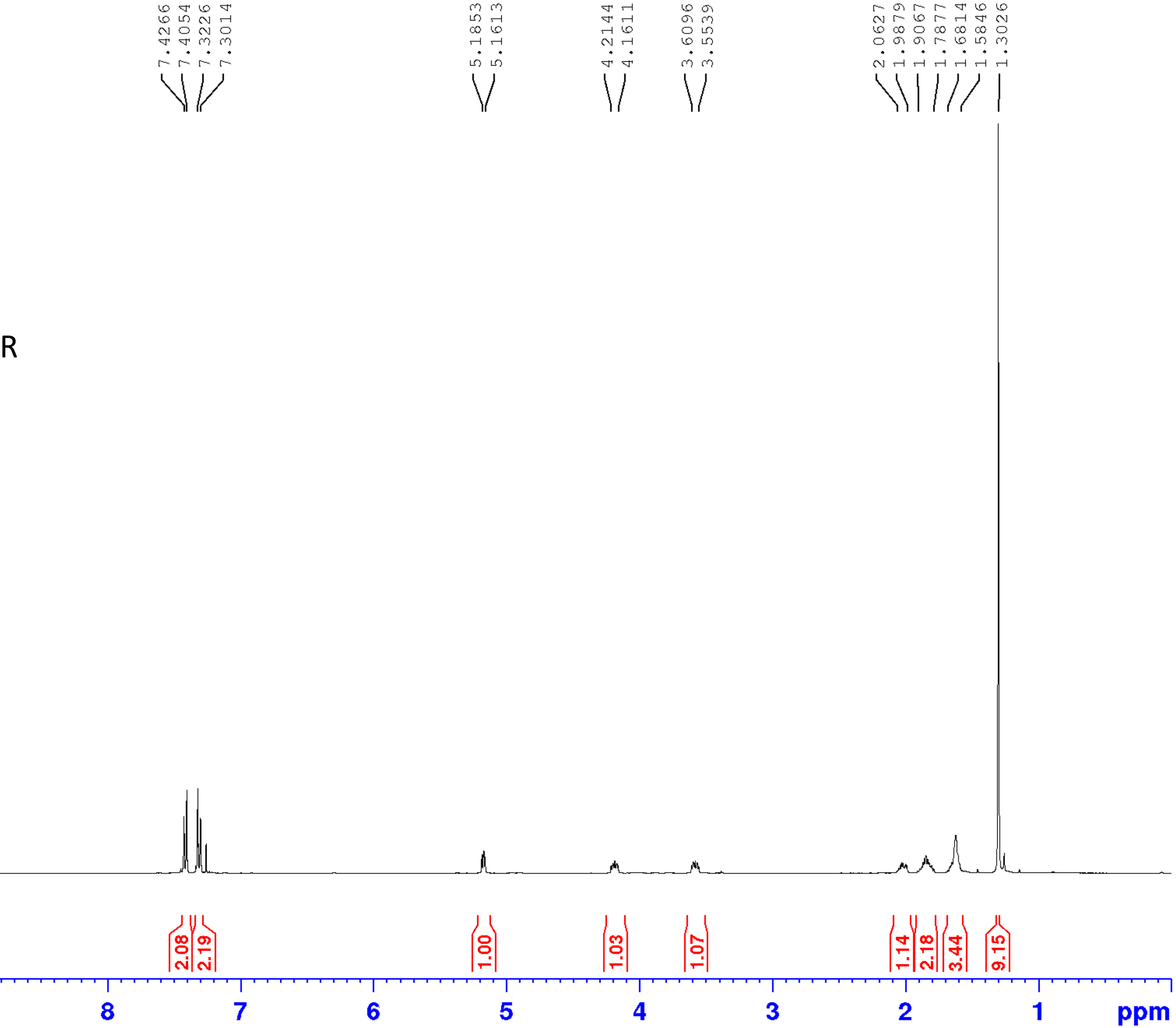


```
Current Data Parameters
NAME       Andy-181-3-islt-20220817
EXPNO                     2
PROCNO                    1

F2 - Acquisition Parameters
Date_           20220817
Time               18.55 h
INSTRUM             spect
PROBHD   Z108618_0257 (
PULPROG              zg30
TD                  65536
SOLVENT             CDCl3
NS                     16
DS                      2
SWH              8012.820 Hz
FIDRES           0.244532 Hz
AQ              4.0894465 sec
RG                    114
DW                 62.400 usec
DE                   6.50 usec
TE                  295.5 K
D1             1.00000000 sec
TD0                     1
SFO1          400.1324708 MHz
NUC1                   1H
P1                  15.00 usec
PLW1          12.50000000 W

F2 - Processing parameters
SI                  65536
SF            400.1300108 MHz
WDW                    EM
SSB                     0
LB                   0.30 Hz
GB                      0
PC                   1.00
```

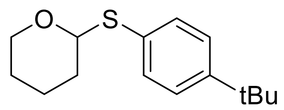

6c

100 MHz ^{13}C NMR

$CDCl_3$

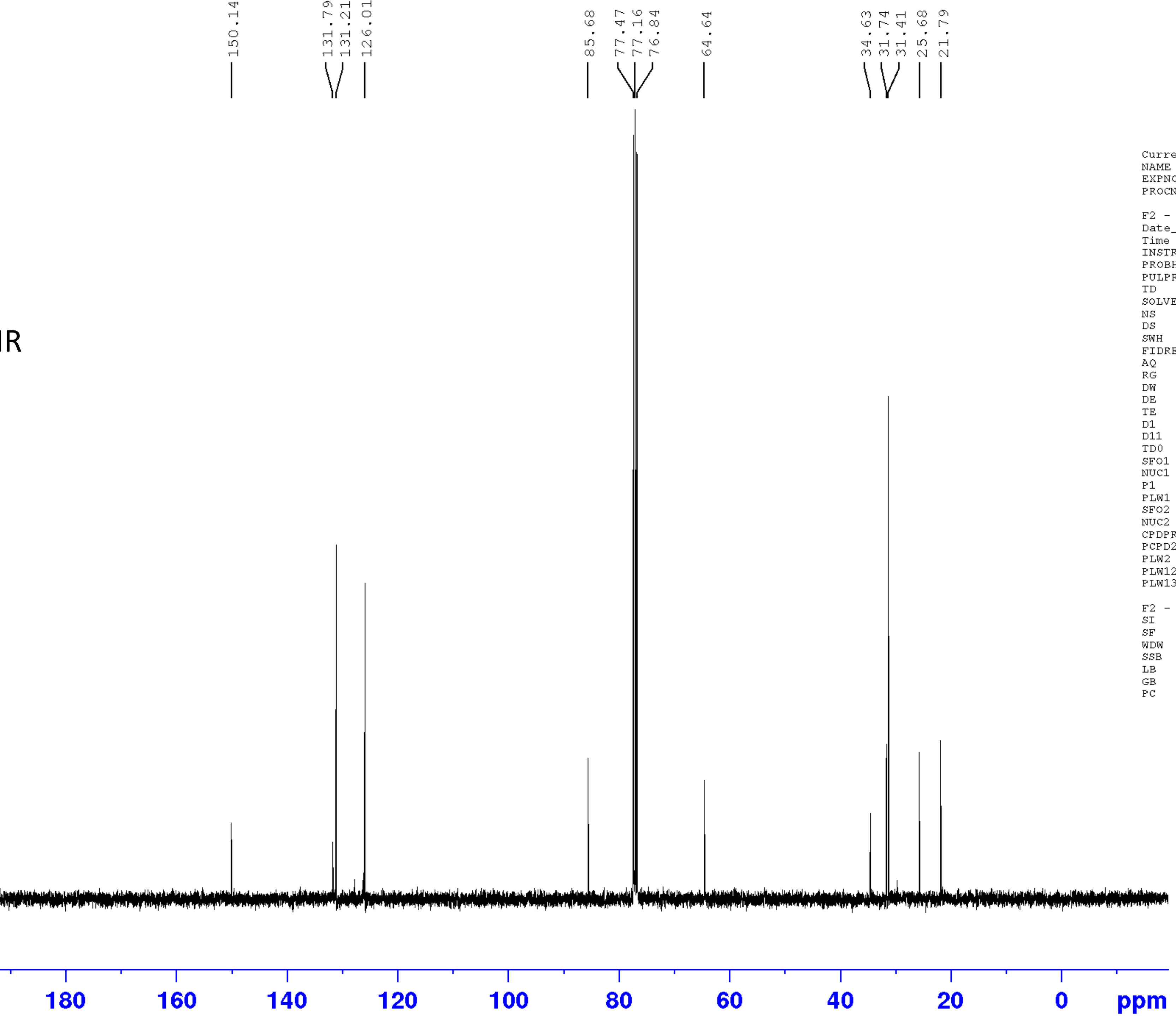


```
Current Data Parameters
NAME      Andy-181-3-islt-20220817
EXPNO                    3
PROCNO                   1

F2 - Acquisition Parameters
Date_             20220817
Time                 19.03 h
INSTRUM              spect
PROBHD     Z108618_0257 (
PULPROG             zgpg30
TD                   65536
SOLVENT              CDCl3
NS                     104
DS                       4
SWH              24038.461 Hz
FIDRES            0.733596 Hz
AQ               1.3631488 sec
RG                     203
DW                  20.800 usec
DE                    6.50 usec
TE                   296.2 K
D1              2.00000000 sec
D11             0.03000000 sec
TD0                      1
SFO1           100.6228298 MHz
NUC1                   13C
P1                   10.00 usec
PLW1           51.00000000 W
SFO2           400.1316005 MHz
NUC2                    1H
CPDPRG[2           waltz16
PCPD2                90.00 usec
PLW2           12.50000000 W
PLW12           0.34722000 W
PLW13           0.17465000 W

F2 - Processing parameters
SI                   32768
SF             100.6127564 MHz
WDW                     EM
SSB                      0
LB                    1.00 Hz
GB                       0
PC                    1.40
```

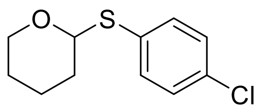

6d

500 MHz ^{1}H NMR

$CDCl_3$

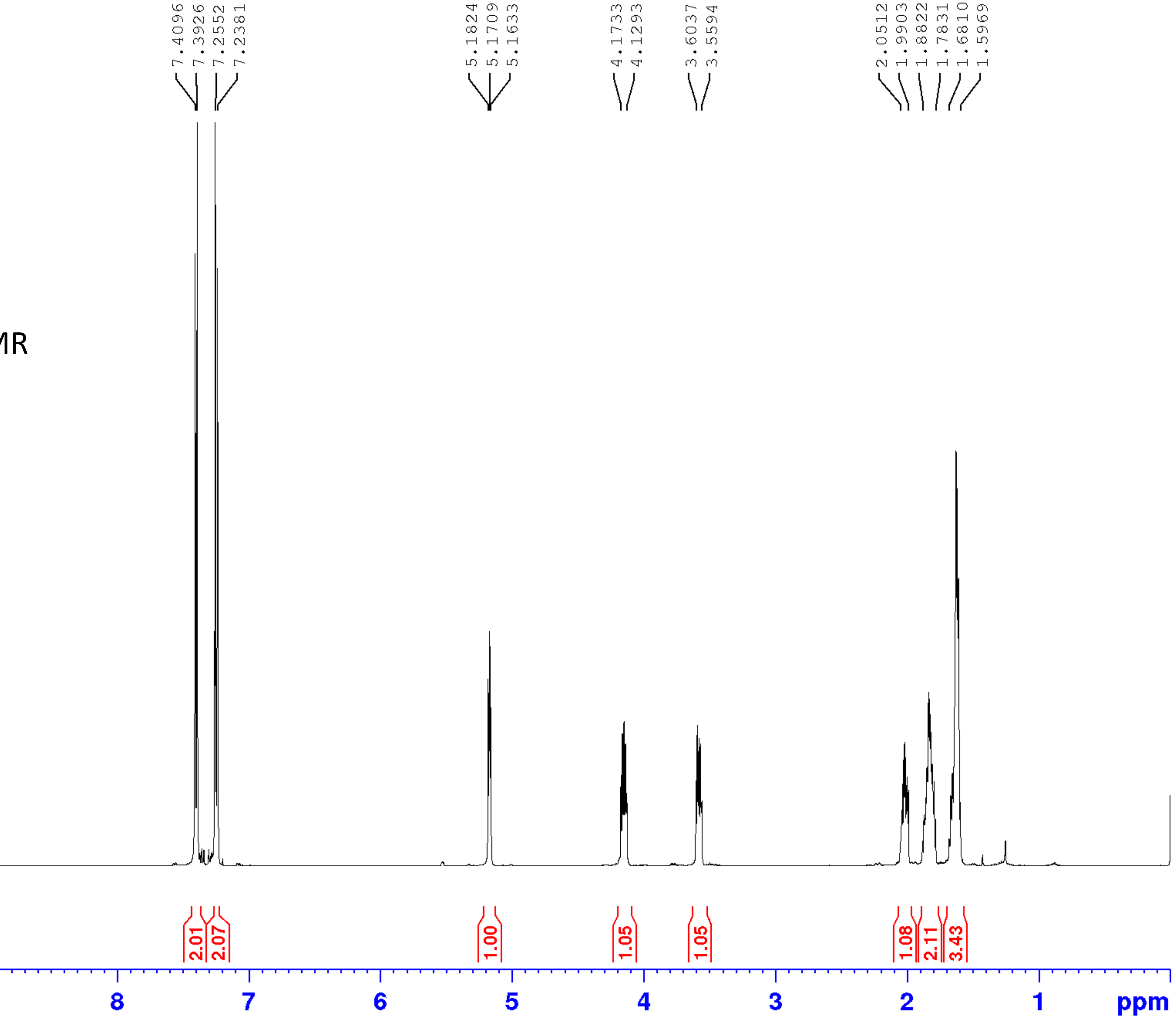

BRUKER

```
Current Data Parameters
NAME      vinn-4-190-4-islt-20230104
EXPNO                          1
PROCNO                         1

F2 - Acquisition Parameters
Date_              20230104
Time                  15.31 h
INSTRUM               spect
PROBHD     Z119470_0283 (
PULPROG                zg30
TD                    65536
SOLVENT               CDCl3
NS                       16
DS                        2
SWH               10000.000 Hz
FIDRES             0.305176 Hz
AQ                3.2767999 sec
RG                    63.76
DW                   50.000 usec
DE                     6.50 usec
TE                    295.1 K
D1               1.00000000 sec
TD0                       1
SFO1            500.1330883 MHz
NUC1                     1H
P1                    10.91 usec
PLW1            25.00000000 W

F2 - Processing parameters
SI                    65536
SF              500.1300120 MHz
WDW                      EM
SSB                       0
LB                     0.30 Hz
GB                        0
PC                     1.00
```

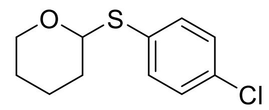

6d

125 MHz ^{13}C NMR

$CDCl_3$

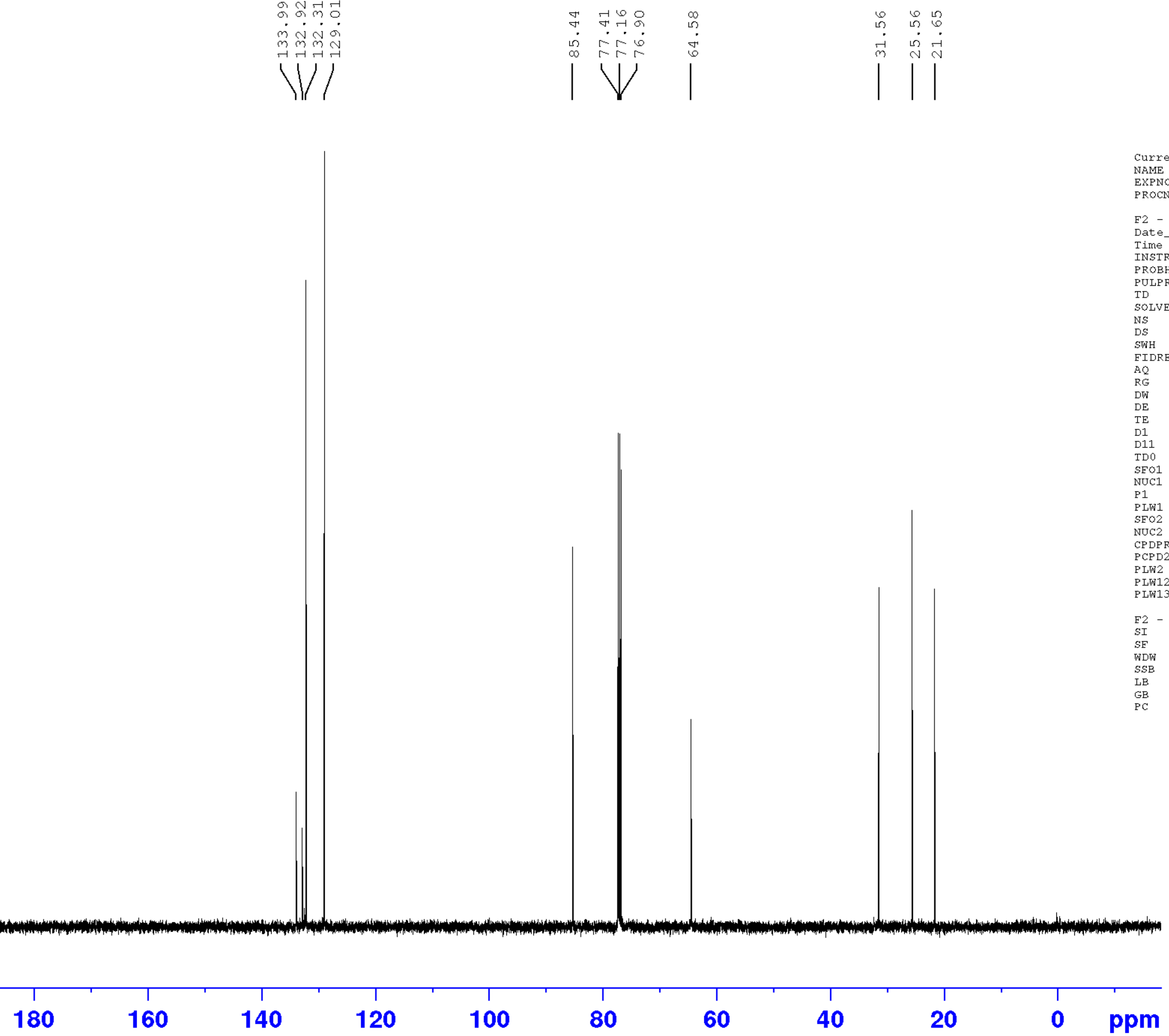


```
Current Data Parameters
NAME     vinn-4-190-4-islt-20230104
EXPNO                 2
PROCNO                1

F2 - Acquisition Parameters
Date_          20230104
Time              15.34 h
INSTRUM           spect
PROBHD   Z119470_0283 (
PULPROG          zgpg30
TD                65536
SOLVENT           CDCl3
NS                   50
DS                    4
SWH          29761.904 Hz
FIDRES        0.908261 Hz
AQ           1.1010048 sec
RG               186.15
DW               16.800 usec
DE                 6.50 usec
TE                295.1 K
D1          2.00000000 sec
D11         0.03000000 sec
TD0                   1
SFO1       125.7703643 MHz
NUC1                13C
P1                 9.75 usec
PLW1       94.00000000 W
SFO2       500.1320005 MHz
NUC2                 1H
CPDPRG[2        waltz16
PCPD2             80.00 usec
PLW2       25.00000000 W
PLW12       0.46495000 W
PLW13       0.23387000 W

F2 - Processing parameters
SI                32768
SF         125.7577768 MHz
WDW                  EM
SSB                   0
LB                 1.00 Hz
GB                    0
PC                 1.40
```

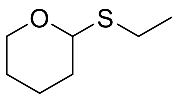

6e

500 MHz ^{1}H NMR

$CDCl_3$

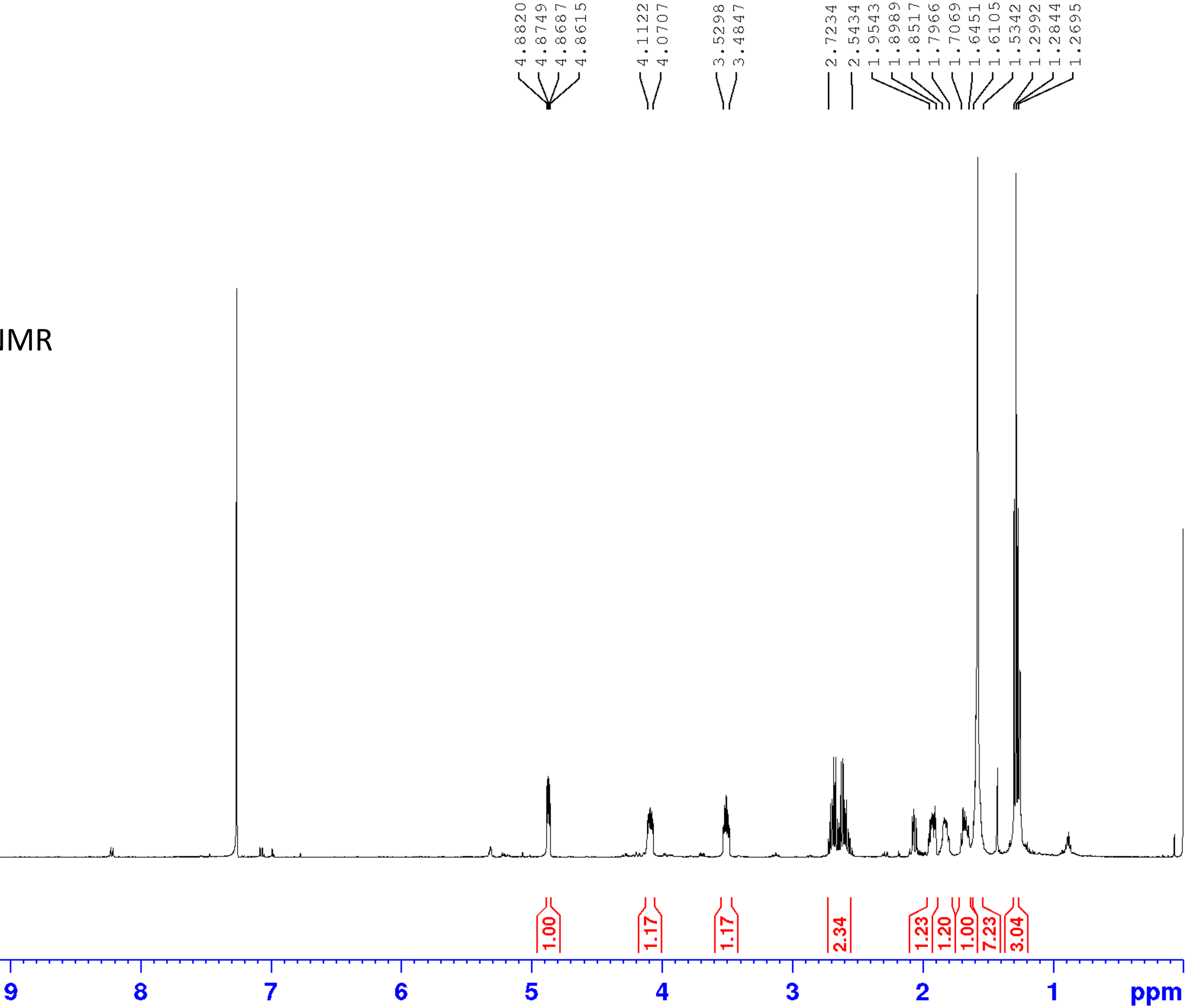


```
Current Data Parameters
NAME     vinn-4-190-5-islt-20230104
EXPNO                    1
PROCNO                   1

F2 - Acquisition Parameters
Date_            20230104
Time                15.38 h
INSTRUM             spect
PROBHD   Z119470_0283 (
PULPROG              zg30
TD                  65536
SOLVENT             CDCl3
NS                     16
DS                      2
SWH             10000.000 Hz
FIDRES           0.305176 Hz
AQ              3.2767999 sec
RG                 163.99
DW                 50.000 usec
DE                   6.50 usec
TE                  295.2 K
D1             1.00000000 sec
TD0                     1
SFO1          500.1330883 MHz
NUC1                   1H
P1                  10.91 usec
PLW1          25.00000000 W

F2 - Processing parameters
SI                  65536
SF            500.1300102 MHz
WDW                    EM
SSB                     0
LB                   0.30 Hz
GB                      0
PC                   1.00
```

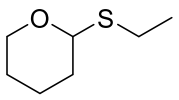

6e

125 MHz ^{13}C NMR

$CDCl_3$

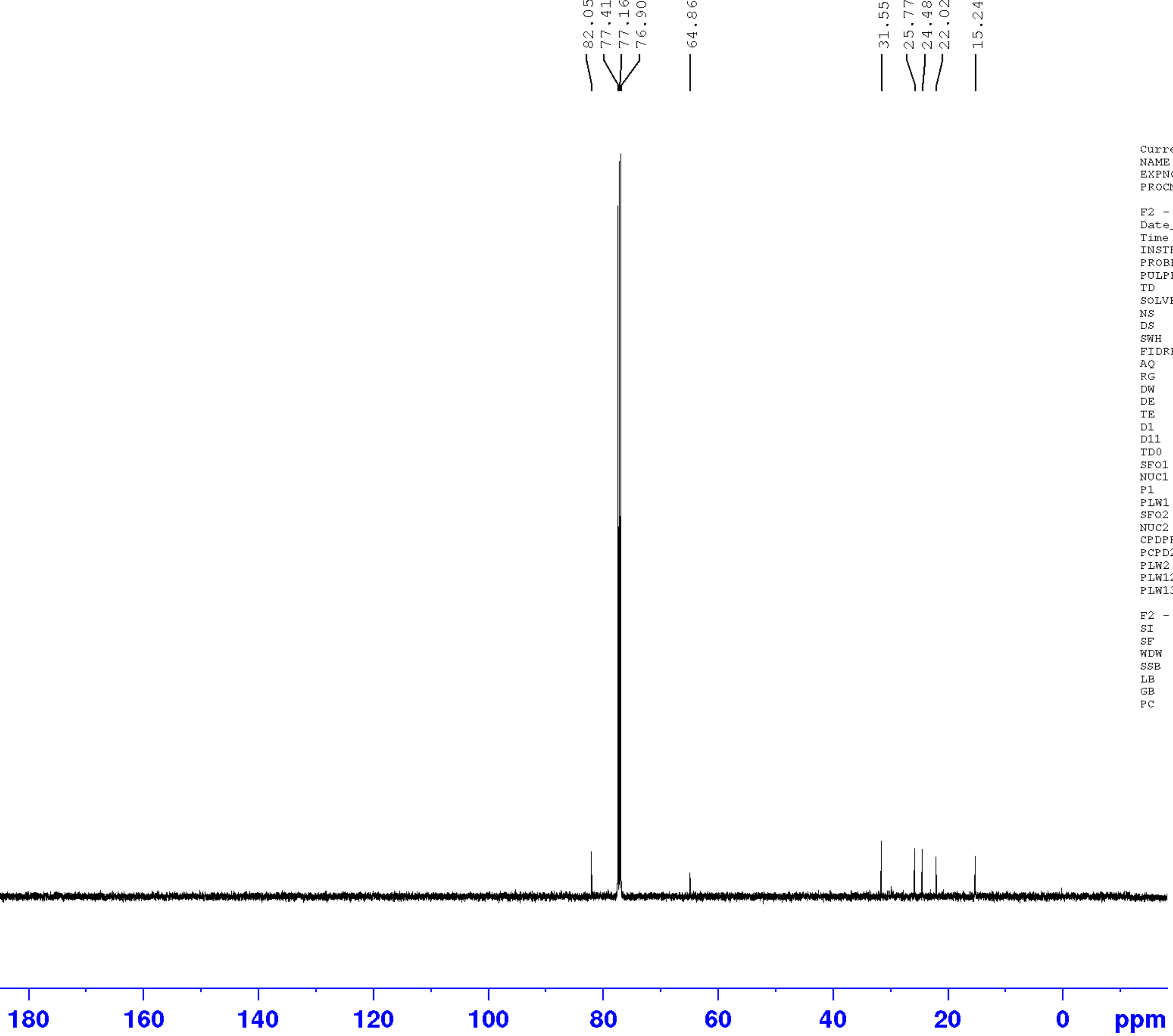

Current Data Parameters
NAME vinn-4-190-5-islt-20230104
EXPNO 2
PROCNO 1

F2 - Acquisition Parameters
Date_ 20230104
Time 15.52 h
INSTRUM spect
PROBHD Z119470_0283 (
PULPROG zgpg30
TD 65536
SOLVENT CDCl3
NS 256
DS 4
SWH 29761.904 Hz
FIDRES 0.908261 Hz
AQ 1.1010048 sec
RG 186.15
DW 16.800 usec
DE 6.50 usec
TE 295.2 K
D1 2.00000000 sec
D11 0.03000000 sec
TD0 1
SFO1 125.7703643 MHz
NUC1 13C
P1 9.75 usec
PLW1 94.00000000 W
SFO2 500.1320005 MHz
NUC2 1H
CPDPRG[2 waltz16
PCPD2 80.00 usec
PLW2 25.00000000 W
PLW12 0.46495000 W
PLW13 0.23387000 W

F2 - Processing parameters
SI 32768
SF 125.7577726 MHz
WDW EM
SSB 0
LB 1.00 Hz
GB 0
PC 1.40

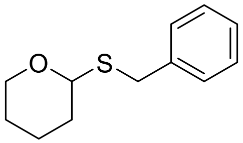

6f

400 MHz ^{1}H NMR

$CDCl_3$

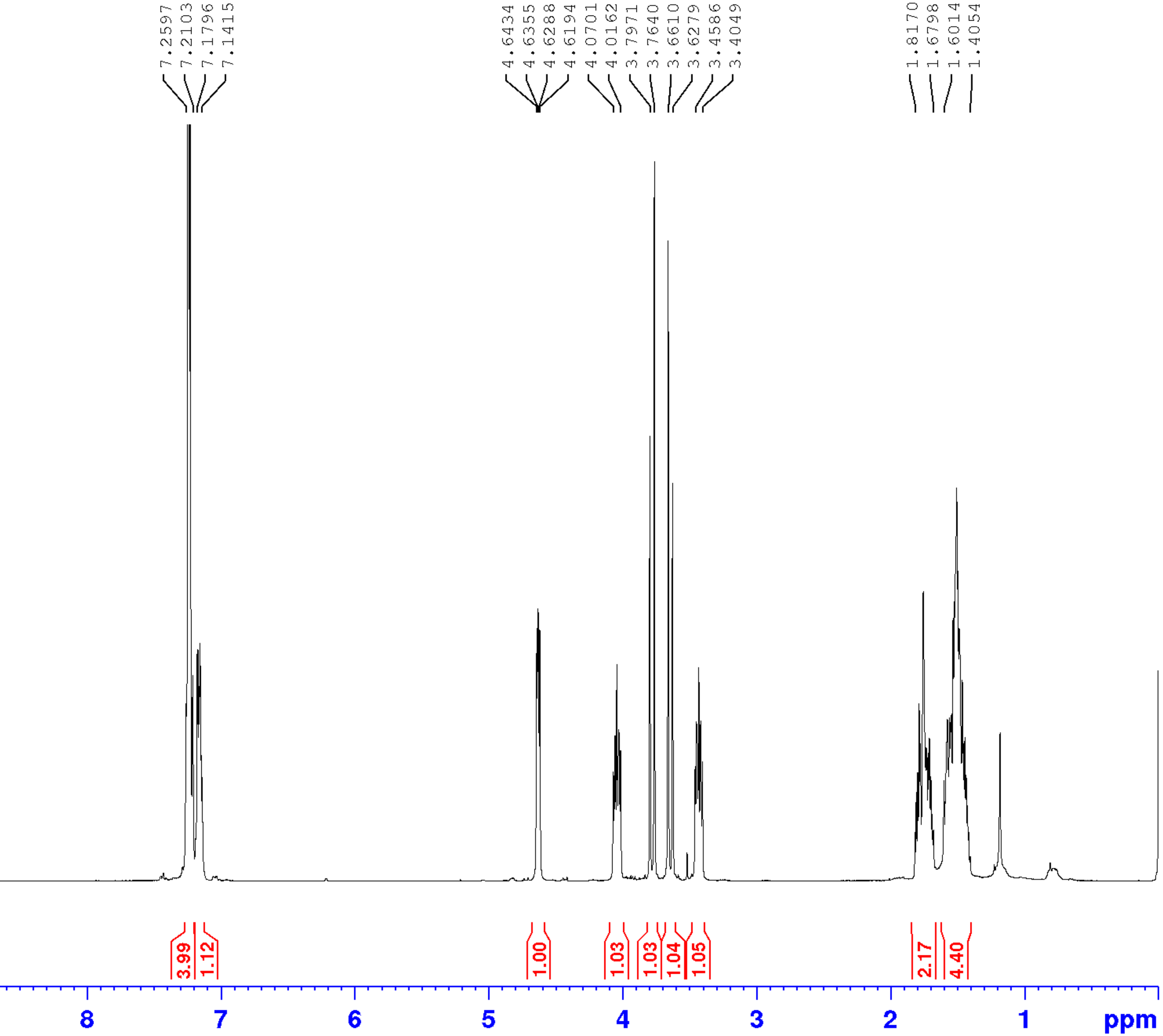

BRUKER

```
Current Data Parameters
NAME       Andy-181-5-islt-20220817
EXPNO                     6
PROCNO                    1

F2 - Acquisition Parameters
Date_              20220817
Time                  19.22 h
INSTRUM               spect
PROBHD    Z108618_0257 (
PULPROG                zg30
TD                    65536
SOLVENT               CDCl3
NS                       16
DS                        2
SWH               8012.820 Hz
FIDRES            0.244532 Hz
AQ               4.0894465 sec
RG                    80.6
DW                  62.400 usec
DE                    6.50 usec
TE                   295.6 K
D1              1.00000000 sec
TD0                       1
SFO1           400.1324708 MHz
NUC1                     1H
P1                   15.00 usec
PLW1           12.50000000 W

F2 - Processing parameters
SI                    65536
SF             400.1300429 MHz
WDW                      EM
SSB                       0
LB                     0.30 Hz
GB                        0
PC                     1.00
```

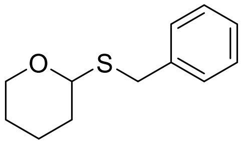

6f

100 MHz ^{13}C NMR

$CDCl_3$

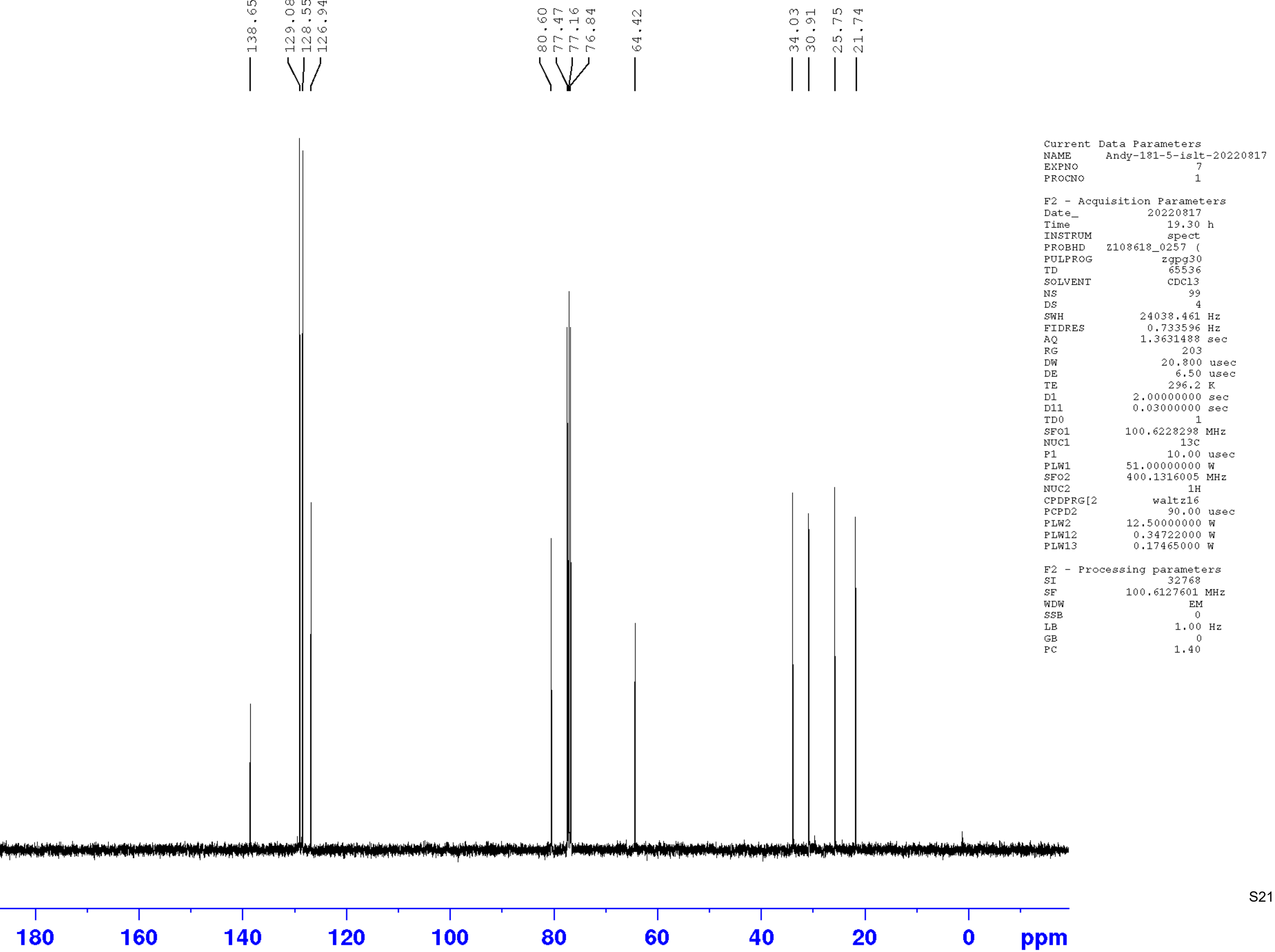

Supplement: SC-014-D3SC03081D-s001 [file SC-014-D3SC03081D-s001.pdf]
